# MTA-Cooperative PRMT5 Inhibitors: Mechanism Switching Through Structure-Based Design

**DOI:** 10.1021/acs.jmedchem.4c01998

**Published:** 2025-02-07

**Authors:** Kevin M. Cottrell, Douglas A. Whittington, Kimberly J. Briggs, Haris Jahic, Janid A. Ali, Alvaro J. Amor, Deepali Gotur, Matthew R. Tonini, Wenhai Zhang, Alan Huang, John P. Maxwell

**Affiliations:** Tango Therapeutics, 201 Brookline Ave, Boston, Massachusetts 02215, United States

## Abstract

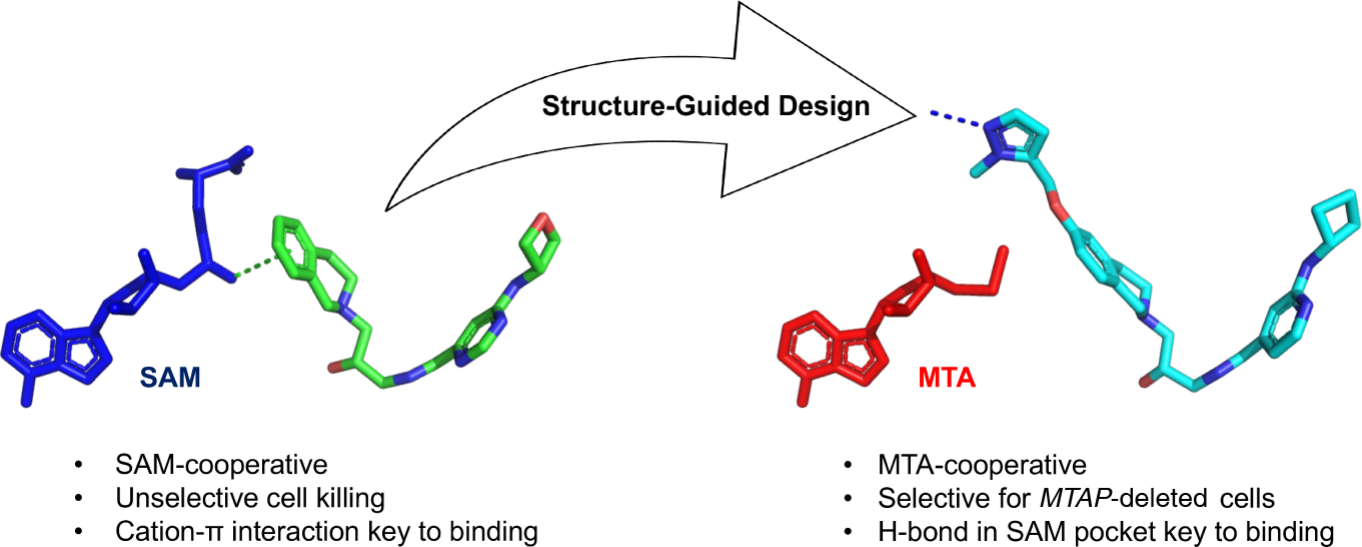

Deletion of the *MTAP* gene leads to accumulation of the substrate of the MTAP protein, methylthioadenosine (MTA). MTA binds PRMT5 competitively with S-adenosyl-l-methionine (SAM), and selective inhibition of the PRMT5•MTA complex relative to the PRMT5•SAM complex can lead to selective killing of cancer cells with *MTAP* deletion. Herein, we describe the discovery of novel compounds using structure-based drug design to switch the mechanism of binding of known, SAM-cooperative PRMT5 inhibitors to an MTA-cooperative binding mechanism by occupying the portion of the SAM binding pocket in PRMT5 that is unoccupied when MTA is bound and hydrogen bonding to Arg368, thereby allowing them to selectively target *MTAP*-deleted cancer cells.

Protein arginine methyltransferase 5 (PRMT5) is a type II arginine methyltransferase that regulates essential cellular functions including transcription, splicing, and cellular homeostasis by catalyzing methyl group transfer from SAM (S-adenosyl-l-methionine) to its substrate proteins as symmetric dimethylation (SDMA) marks.^1−3^ The first generation of PRMT5 inhibitors for the treatment of cancer were either SAM-cooperative (SAM-uncompetitive) or SAM-competitive.^4−6^ As these mechanisms of binding inhibit PRMT5 equally in both normal and cancer cells, these compounds led to on-target toxicities and were discontinued in the clinic due to poor tolerability.^7−9^

*MTAP* deletions occur in 10–15% of human cancers, providing one of the largest precision oncology patient populations.^10−12^ As MTAP is the only enzyme that catabolizes MTA (methylthioadenosine), an intermediate in the methionine salvage pathway, there is an accumulation of MTA in cells with *MTAP* deletion, and it acts as a weak, endogenous inhibitor of PRMT5 by binding competitively with SAM.^13,14^ MTA-cooperative PRMT5 inhibitors, i.e., compounds that can bind in the substrate binding pocket preferentially when MTA is in the SAM cofactor pocket, including clinical-stage compounds TNG908, TNG462, MRTX1719 (BMS-986504), and AMG 193 (Figure 1),^15−18^ differ from the first-generation inhibitors because they leverage the elevated levels of MTA in *MTAP*-deleted (MTAP-null) cells to bind to and inhibit the PRMT5•MTA complex and selectively kill MTAP-null cells while sparing normal tissue, resulting in a broader therapeutic index.

**Figure 1:**
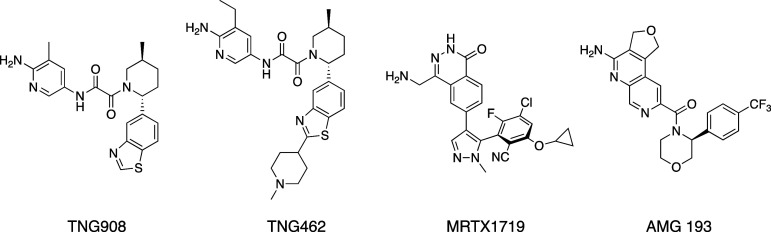
Chemical structures of the clinical MTA-cooperative PRMT5 inhibitors TNG908, TNG462, MRTX1719 (BMS-986504), and AMG 193.

While performing a high-throughput screen (HTS) that identified the hits that led to the discovery of TNG908 and TNG462, we embarked on a structure-based design effort to switch the mechanism of publicly disclosed first-generation PRMT5 inhibitors from SAM-cooperative to MTA-cooperative inhibition. This effort led to the first publicly disclosed compounds that bind to and inhibit PRMT5 preferentially as the PRMT5•MTA complex and not with the PRMT5•SAM complex, and that selectively kill MTAP-null cells relative to MTAP WT cells,^19,20^ and provided important chemical tools for the advancement of our understanding of the biology in the MTA-cooperative PRMT5 space.

## Results and Discussion

**Figure 2:**
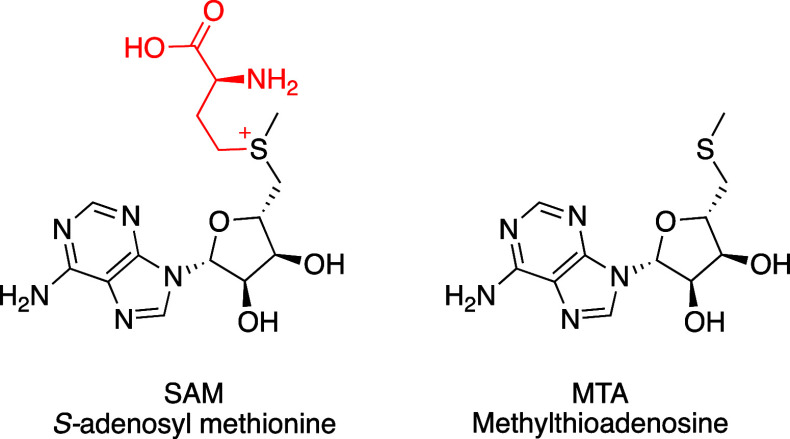
Chemical structures of S-adenosyl methionine (SAM) and methylthioadenosine (MTA).

A key difference in the structure of MTA relative to SAM, in addition to the lack of a sulfur cation, is the absence of the −CH_2_CH_2_CH(COOH)NH_2_ group (Figure 2). Analysis of the crystal structures of MTA and SAM bound to PRMT5 revealed a small pocket created by the absence of this SAM amino-carboxy terminus. We set out to design molecules that would exploit this structural difference through a combination of steric and polar interactions with the residues in that pocket. The resulting inhibitors would bind efficiently with MTA but would compete with SAM for occupancy of the cofactor site. The net result would be a mechanism switch, whereby a SAM-cooperative PRMT5 inhibitor scaffold is converted into a SAM-competitive and MTA-cooperative PRMT5 inhibitor scaffold.

**Figure 3:**
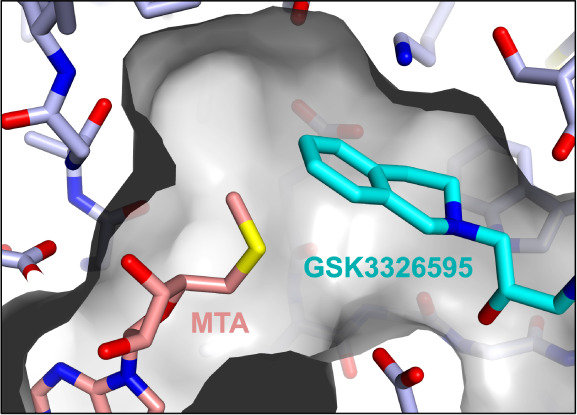
X-ray crystal structure of GSK3326595 (pemrametostat; blue, PDB9MGL) with PRMT5•MTA reveals a small pocket adjacent to the 6-position of the tetrahydroisoquinoline relative to the structure with SAM.

As a starting point for design, we focused on Compound **1** (GSK3203591/EPZ015866),^21^ an analog of the MTAP-unselective PRMT5 inhibitor GSK3326595 (pemrametostat) that was in a Phase 1 clinical trial at the time.^22^**1** is a substrate-competitive, SAM-cooperative inhibitor of PRMT5, and its binding is characterized by a unique cation−π interaction between the positively charged sulfur atom of SAM and the phenyl ring of the tetrahydroisoquinoline (THIQ) group. A crystal structure of GSK3326595 with PRMT5•MTA highlighted the proximity of the THIQ to the methionine region of the SAM binding pocket (Figure 3) which offered an opportunity to grow into this space and potentially achieve our mechanism switch goals.

Due to its orientation in the pocket, we biased our exploration at the 6-position of the THIQ ring. We did, however, explore all positions around the aromatic ring to account for potential movement in the binding site and made modifications to the ring itself, but none of those efforts were fruitful. (The data for substitution other than the 6-position and ring modifications are not disclosed here). We utilized a combination of structure-guided design and systematic exploration, starting with small substituents containing N, O, and C atom connections to the ring. The potency of the compounds was measured in a fluorescence anisotropy (FA) assay to detect displacement of a TAMRA (tetramethylrhodamine)-labeled histone H4 peptide from PRMT5 in the presence of 50 μM MTA to monitor desired activity, in comparison to activity in the presence of 50 μM SAM to monitor selectivity. A subset of compounds representing that exploration is shown in Table 1.

**Table 1:**
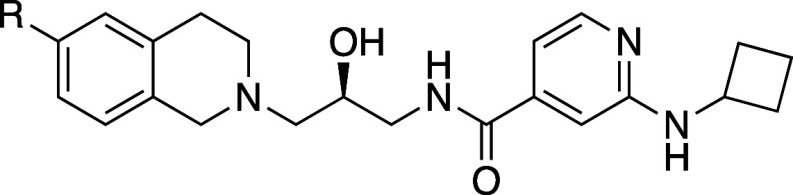
Biochemical Assay Data for Compounds 1–12

		PRMT5 biochemical *K*_i, app_ (μM)^a^	
Compound	R	+ MTA	+ SAM
1	H	3	<0.03
2	OH	4	<0.03
3	OMe	0.6	<0.03
4	NH_2_	>19	0.04
5	NHMe	13	0.15
6	CN	4	0.5
7	COOH	>19	>95
8	CONH_2_	>19	47
9	CONHMe	>19	>95
10	NHCOMe	>19	66
11	CH_2_CO_2_H	>19	13
12	3-THF^b^	>19	>95

a Detection of displacement of a TAMRA-labeled peptide by fluorescence anisotropy. *K*_i,app_ calculated from IC_50_ values using the Cheng–Prusoff equation.

b Racemic.

The baseline compound (**1**) had excellent potency with SAM (*K*_i,app, SAM_ = 0.03 μM**)**, reaching the detection limit of the assay, and weak activity with MTA (*K*_i,app MTA_ = 3 μM**)**. At the 6-position, N, C=O, and C linked analogs (**5**, **7**–**12**) led to significant loss of activity with both MTA and SAM, the NH_2_ analog (**4**) retained potency with SAM but lost activity with MTA, and the cyano analog (**6**) was equipotent to **1** with MTA but 18-fold less potent with SAM. O-linked substitutions showed the most promise; while phenol (**2**) was equipotent to **1** with both MTA and SAM, methoxy substitution (**3**) had a 5-fold improvement in potency with MTA. Despite the maintenance of potency with SAM, the improvement in potency of **3** with MTA suggested that the exploration of the O-linked analog could be fruitful.

To further explore the O-linked series, a selection of extended ethers was prepared to probe deeper into the SAM pocket with the goal of improving binding with MTA and increasing desired potency while competing with SAM to increase selectivity. We varied the length and rigidity of the linker, and the number and location of heteroatoms in the substituent to scan the space for productive interactions. A subset of compounds representing that exploration is shown in Table 2.

**Table 2:**
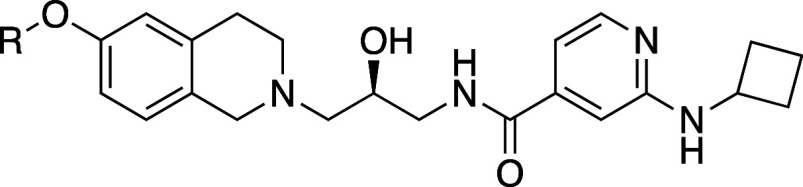

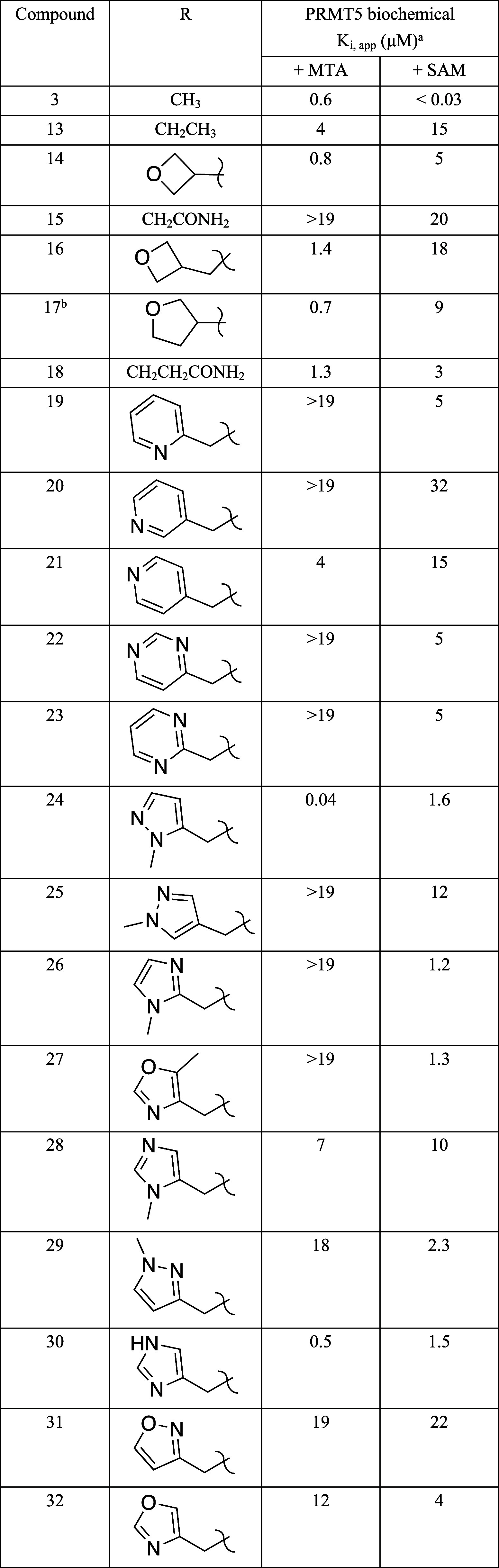
Biochemical Assay Data for Compounds 13–32

a Detection of displacement of a TAMRA-labeled peptide by fluorescence anisotropy. *K*_i, app_ calculated from IC_50_ values using the Cheng–Prusoff equation.

b Racemic.

These larger substituents led to reduced potency with SAM, while most substituents either did not show improvement in potency with MTA (**14**, **16**–**18**, **30**) or were worse (**13**, **15**, **19**–**23**, **25**–**29**, **31**, **32**). A standout compound was methyl pyrazole **24**, which had a 15-fold improvement in potency with MTA (*K*_i,app, MTA_ = 0.04 vs 0.6 μM) and a 53-fold reduction in potency with SAM (*K*_i,app, SAM_ = 1.6 vs 0.03 μM) relative to methyl ether compound **3**. This resulted in a selectivity of 40-fold for PRMT5•MTA versus PRMT5•SAM and was our first proof of concept that (1) selective biochemical binding with PRMT5•MTA versus PRMT5•SAM could be achieved, and (2) a rationally designed mechanism switch was feasible. Biophysical binding experiments using SPR on compound 24 and a subset of additional compounds confirmed this behavior (Figure SI-1 and Table SI-1).

**Figure 4:**
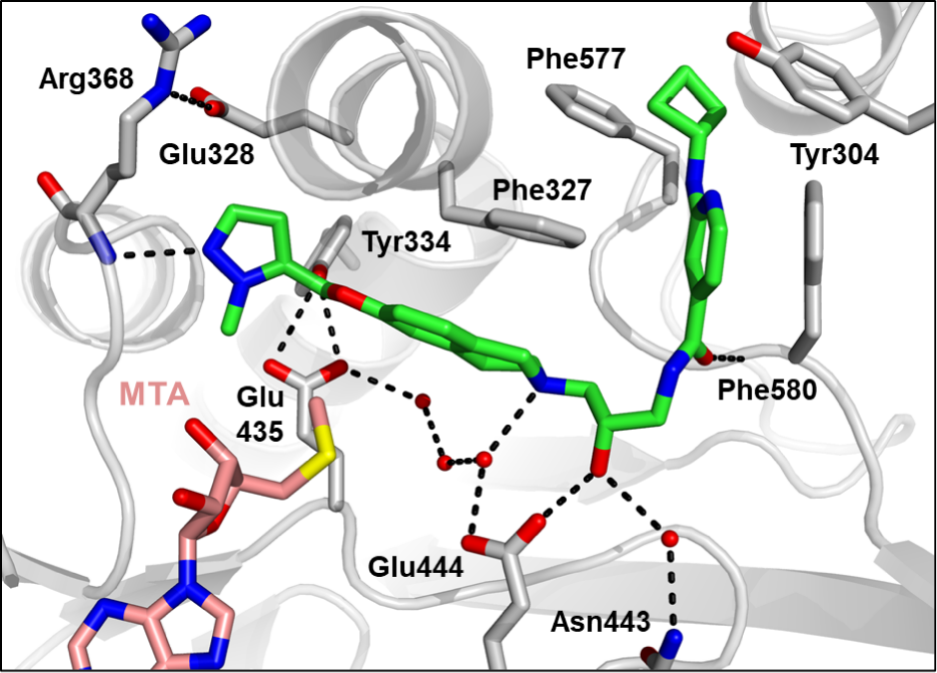
X-ray crystal structure of **24** (green, PDB entry 9MGM) with PRMT5•MTA. The crystal structure shows the ether extension reaching into the SAM binding pocket and making a hydrogen bond from the pyrazole N to the backbone NH of Arg368.

A crystal structure of **24** (Figure 4) revealed that the pyrazole forms a hydrogen bond with the backbone NH of Arg368 in the SAM binding pocket. While this compound retained the THIQ ring system which could form the same cation−π interaction with the sulfur cation of SAM, by extending into the SAM binding pocket and forming a hydrogen bond with Arg368 the molecule competes with SAM, increasing the selectivity for PRMT5•MTA versus PRMT5•SAM.

Looking to improve potency further, since there did not appear to be space in the pocket for significant growth of the substituent, we prepared a focused series of compounds that maintained a hydrogen bond acceptor in the appropriate position to further develop the SAR. A subset of these compounds is shown in Table 3.

**Table 3:**
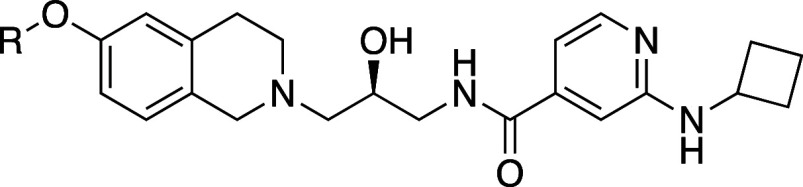

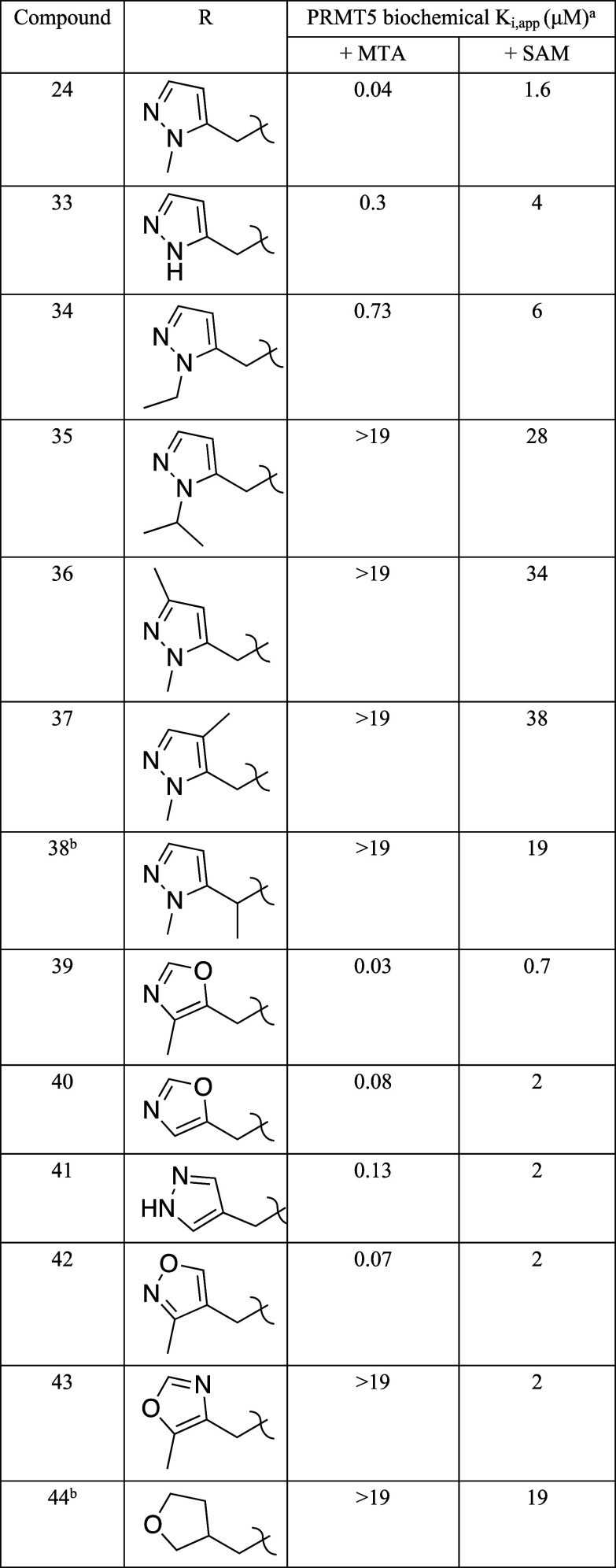
Biochemical Assay Data for Compounds 33–44^a^^b^

a Detection of displacement of a TAMRA-labeled peptide by fluorescence anisotropy. *K*_i, app_ calculated from IC_50_ values using the Cheng–Prusoff equation.

b Racemic.

**Figure 5:**
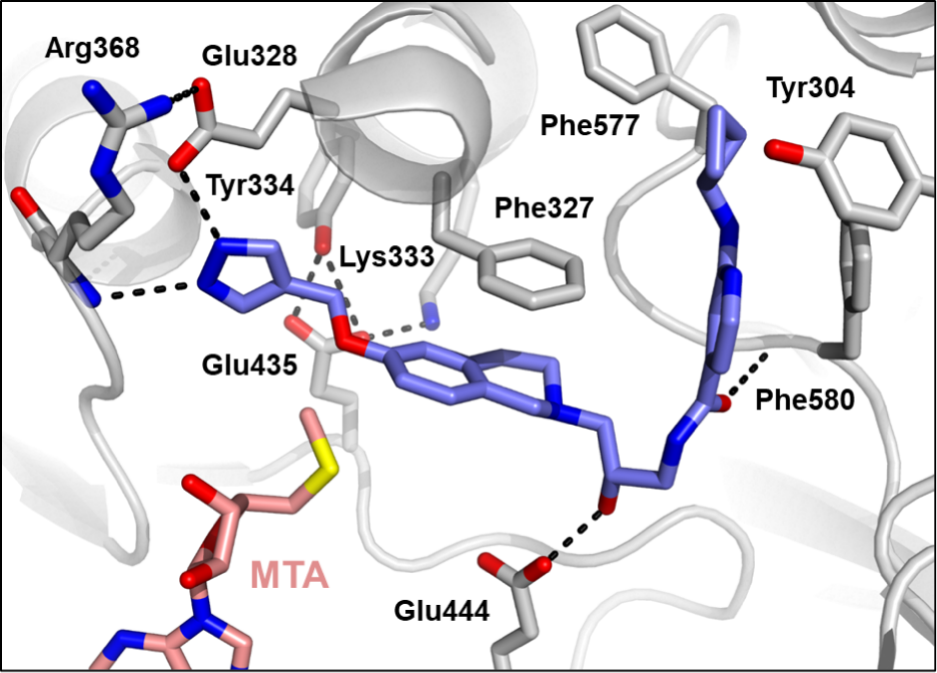
X-ray crystal structure of **41** (purple, PDB entry 9MGN) bound to PRMT5•MTA with pyrazole forming two hydrogen bonds to Arg368 and Glu328.

Removal of *N*-methyl (**33**) resulted in an 8-fold loss of potency with MTA (*K*_i,app,MTA_ = 0.3 μM). Increasing the size of the N-substituent also reduced potency with MTA (**34**: ethyl, *K*_i,app,MTA_ = 0.73 μM and **35**: isopropyl, *K*_i,app,MTA_ > 19 μM). A methyl walk (**36**–**38**) around the pyrazole ring and the connecting chain also led to a loss of potency in all cases. Oxazoles (**39** and **40**) and isoxazole (**42**) had potency similar to that of **24** (**39**: *K*_i,app,MTA_ = 0.03 μM, **40**: *K*_i,app,MTA_ 0.08 μM, **42**: *K*_i,app,MTA_ = 0.07 μM). Regioisomeric pyrazole (**41**), despite making an additional hydrogen bond with the side chain acid of Glu328 (Figure 5), lost about 3-fold in potency (*K*_i,app,MTA_ = 0.13 μM). Regioisomeric oxazole (**43**), in which the oxygen atom is the hydrogen bond acceptor to Arg368, and the saturated THF compound (**44**) showed no activity in the assay. In all, with this set of compounds, we had a series of PRMT5 inhibitors with selective potency for the PRMT5•MTA complex; however, these compounds did not show selective cellular potency. We hypothesized that additional potency with PRMT5•MTA was needed to demonstrate significant selective cellular activity in MTAP-null cells.

**Figure 6:**
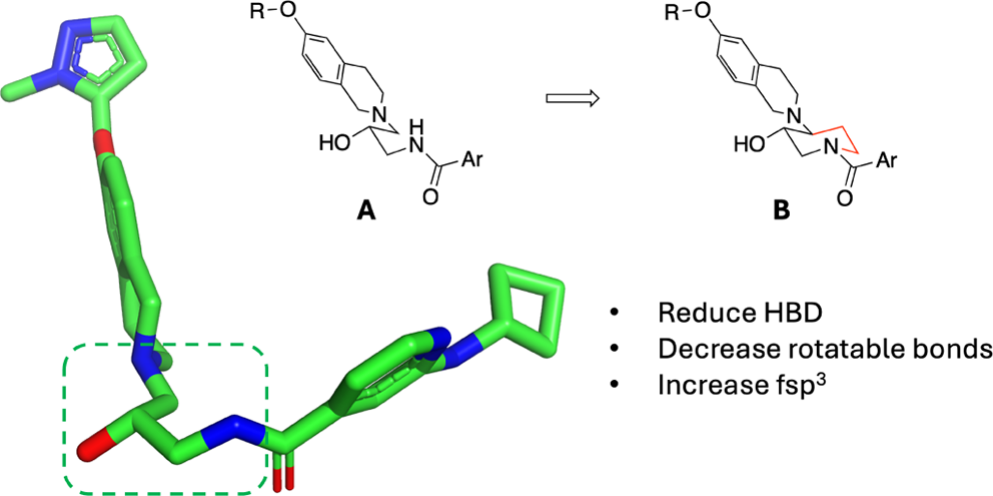
Cyclization of the central chain of A leads to B as a hypothesis for design that led to 46a.

Some efforts were made to additionally substitute around the THIQ ring, to change the ring system entirely, or to remove aromaticity to disrupt the cation-π interaction with SAM, but none of those efforts were successful (not shown). One modification to modify the molecule that was fruitful was rigidification of the bioactive conformation by cyclization of the chain connecting the THIQ to the amide (Figure 6).

Guided by crystal structure, we hypothesized that we could conformationally restrict the central chain of the molecule by connecting the α carbon on the chain to the THIQ of **24** by cyclizing it onto the amide nitrogen, generating a 3,4-disubstituted piperidine with *trans* orientation of the alcohol and THIQ. Based on prior experience with these molecules, we recognized that subtle changes around the THIQ system such as placement of the OH group, could shift how the ring sits in the pocket and potentially affect the ability of the extension to bind effectively in the SAM pocket. Based on molecular models, we noticed two things: (1) it appeared that there was enough flexibility in the space that either *trans* isomer of the piperidine could potentially work if the THIQ ring moved to accommodate it, and (2) the two-atom linker from the THIQ ring to the pyrazole could be too long for the size of the pocket with the piperidine core in place. To account for these possibilities, we prepared both *trans* enantiomers of the ether-linked compound (**45a** and **45b**) as well as both *trans* enantiomers of a shortened variant where we removed the O atom from the chain (**46a** and **46b**).

**Figure 7:**
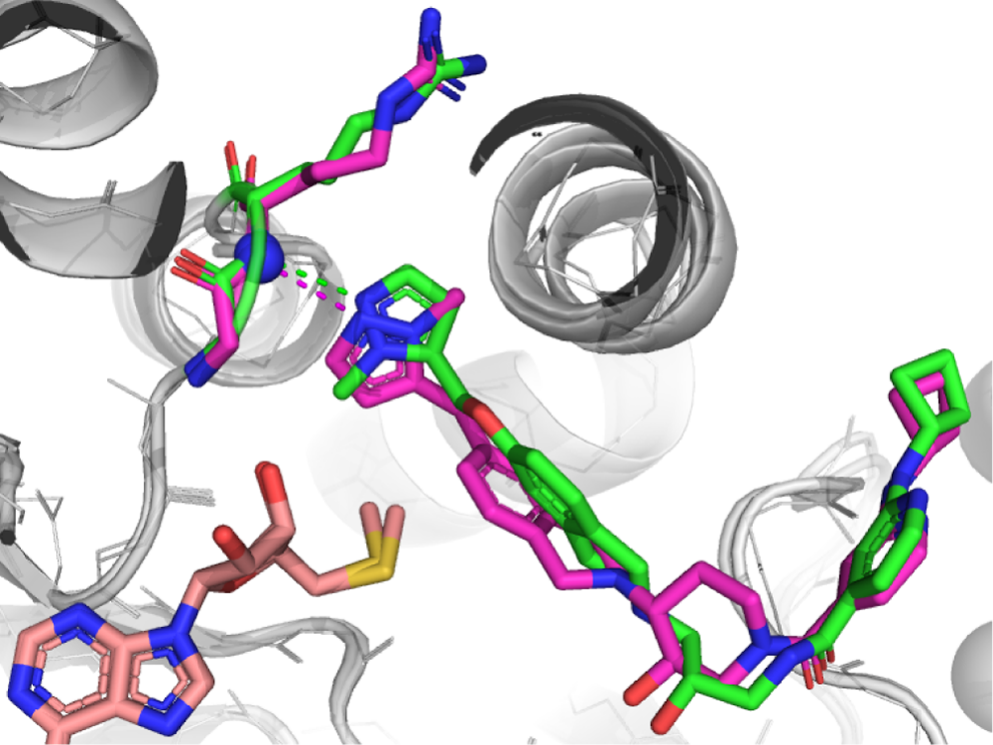
X-ray crystal structures of **46a** (magenta, PDB 9MGP) overlaid on **24** (green) showing the methylpyrazole subgroup flipped in the binding pocket but each still engaging Arg368 in a hydrogen bond.

These compounds were prepared as racemates, which were separated by SFC chromatography, with the stereochemistry around the piperidine ring initially assigned arbitrarily. Both cyclized ether compounds lost potency compared to the uncyclized (**45a**: *K*_i,app,MTA_ = 2.3 μM and **45b**: >19 μΜ, vs **24**: 0.04 μM), but one of the isomers with the truncated chain showed good potency (**46a**: K_i,app,MTA_ = 0.2 μM), while the other compound (**46b**) was inactive in the assay. A crystal structure of **46a** showed that the THIQ ring system did indeed rotate somewhat and the *R,R* enantiomer of the piperidine was preferred. The effect of removing the oxygen from the chain reduced conformational flexibility and led to a 180° flip of the pyrazole which still maintained a hydrogen bond with the backbone nitrogen of Arg368 (Figure 7). While giving no apparent advantages in potency, this core morph reduced the number of rotatable bonds, hydrogen bond donor and acceptor count, and led to increased permeability and reduced efflux compared to **24** (MDCK-Mdr1 A-B **46a** = 2.6 × 10^–6^ cm/s vs **24** = 0.8 × 10^–6^ cm/s and MDCK Mdr1 efflux ratio = 9 vs 34), knowledge which could later prove useful to achieving more drug-like properties in the series. These data are summarized in Table 4.

**Table 4:**
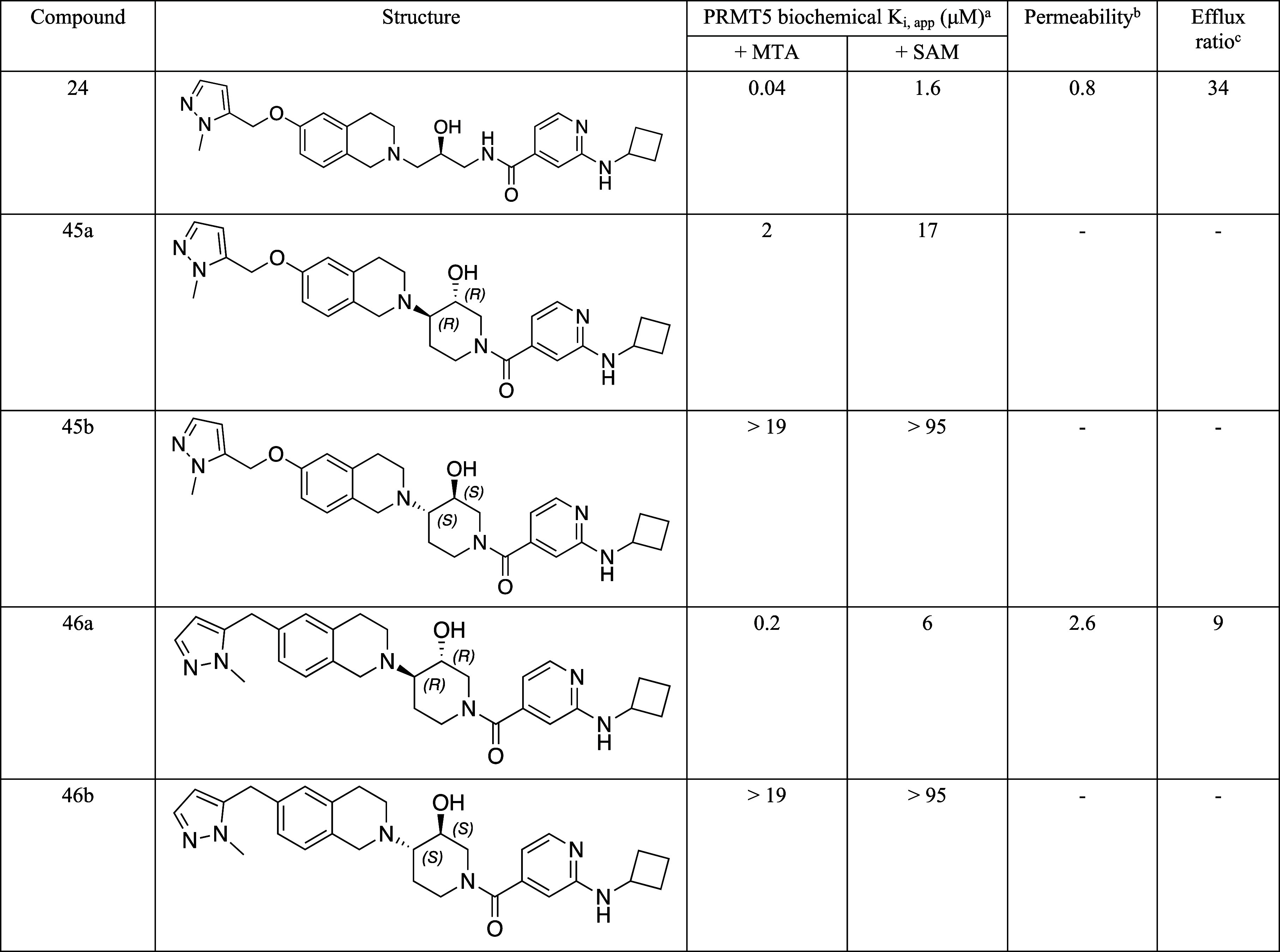
Biochemical, Permeability, and Efflux Assay Data for Compounds 45a, 45b, 46a, and 46b^a^^b^

a Detection of displacement of a TAMRA-labeled peptide by fluorescence anisotropy. *K*_i,app_ calculated from IC_50_ values using the Cheng–Prusoff equation.

b MDCKII-WT A–B (10^–6^ cm/s). ^c^MDCKII-Mdr1 cells (A–B)/(B–A).

**Figure 8:**
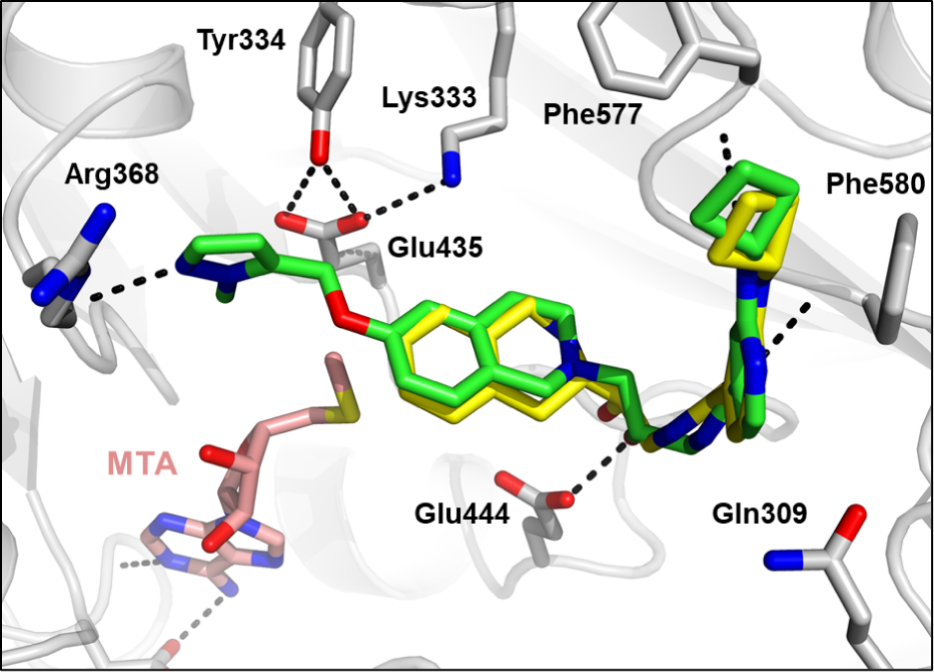
X-ray crystal structure overlays of 47 (yellow, PDB entry 9MGQ) in PRMT5•sinefungin and 24 (green) in PRMT5•MTA suggest that 7-substitution on 47 provides the best opportunity to access the SAM pocket to achieve MTA cooperativity. Only the protein and MTA when bound with 24 is depicted.

A series of C-linked THIQ analogs had also been reported as SAM cooperative PRMT5 inhibitors^23,24^ (represented by **47**). An overlay of the crystal structures of **24** and **47** (Figure 8) suggested that the SAM binding pocket of PRMT5 could be accessed through substitution at position 7 in the C-linked series. A selection of 7-ether-linked compounds was prepared, and representative matched pairs derived from the N-linked series are shown in Table 5. Compounds **48**–**50** achieved potency that reached the lower detection limit of the peptide displacement assay, and at this point, we observed cellular activity with an MTAP-null IC_50_ as low as 500 nM and greater than 20-fold cellular selectivity for **49** as measured by an SDMA in-cell western (ICW) pharmacodynamic assay in *MTAP*-isogenic HAP1 cells. While encouraging, this potency level was insufficient to drive a significant effect in a viability assay, suggesting more potency was needed.

**Table 5:**
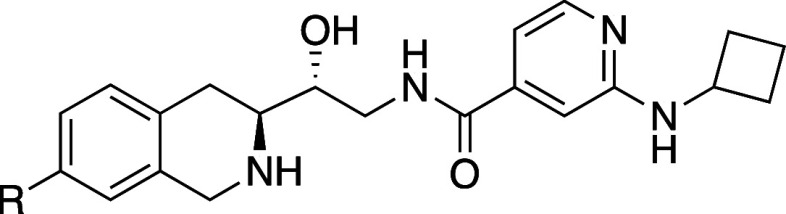

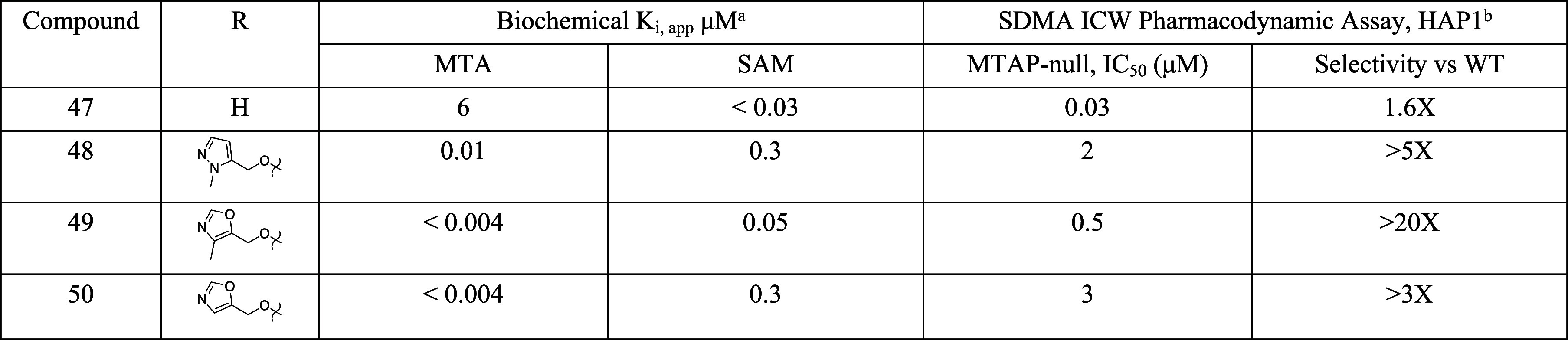
Biochemical and Cellular PD Assay Data for Compounds 47–50^a^^b^

a Detection of displacement of a TAMRA-labeled peptide by fluorescence anisotropy. *K*_i,app_ calculated from IC_50_ values using the Cheng–Prusoff equation.

b Inhibition of PRMT5 determined by an SDMA in-cell western assay in the HAP1 *MTAP*-isogenic cell line pair following 24 h compound treatment.

We believed the SAR of changes made in the substrate binding pocket far from the SAM binding pocket should be similar in both the newly discovered MTA-cooperative series as in the SAM-cooperative series, so we kept the oxymethyl-4-methyloxazole THIQ fixed, and matched molecular pair^25^ substitutions on the aromatic ring attached to the amide were explored, guided by published SAR from a SAM-cooperative series.^26,27^ A broad exploration of that SAR proved that hypothesis to be correct. Many of these compounds also reached the detection limit of the biochemical assay, showed improved potency in the SDMA ICW assay, and ultimately achieved selective activity in a cellular viability assay. SAR for this work was followed by cellular activity, and a selection of these compounds is shown in Table 6.

**Table 6:**
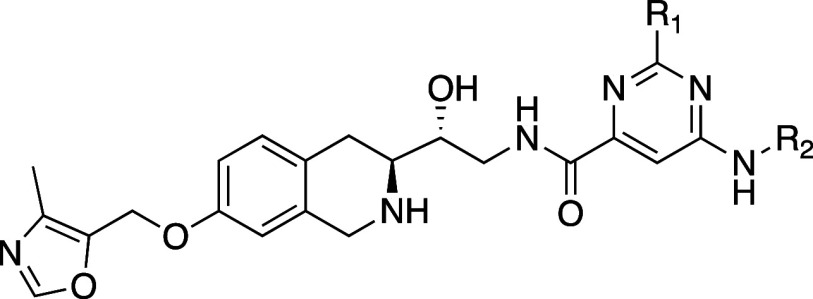

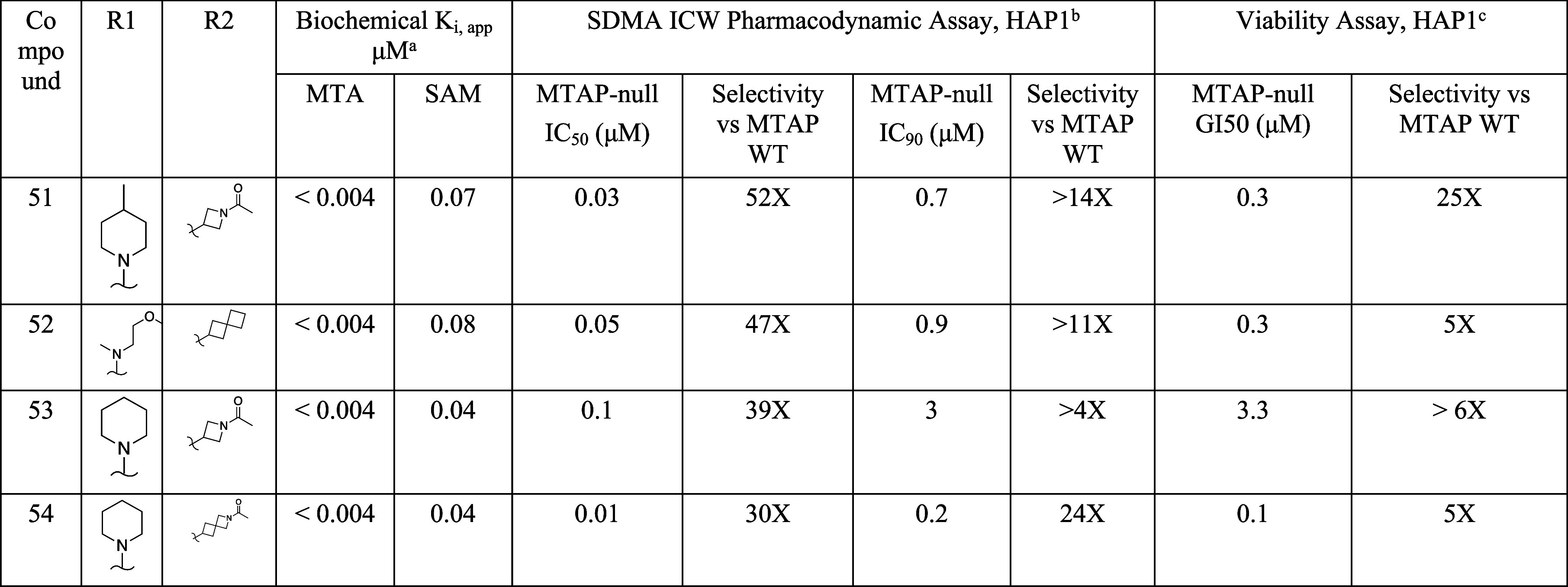
Biochemical, Cellular PD, and Viability Assay Data for Compounds 51–54^a^^b^^c^

a Detection of displacement of a TAMRA-labeled peptide by fluorescence anisotropy. *K*_i,app_ calculated from IC_50_ values using the Cheng–Prusoff equation.

b Inhibition of PRMT5 determined by an SDMA in-cell western assay in the HAP1 *MTAP*-isogenic cell line pair following 24 h compound treatment.

c Viability growth inhibition assessed after 7 days of compound treatment using a CellTiter-Glo luminescence-based assay in the same HAP1 *MTAP*-isogenic cells.

**Figure 9:**
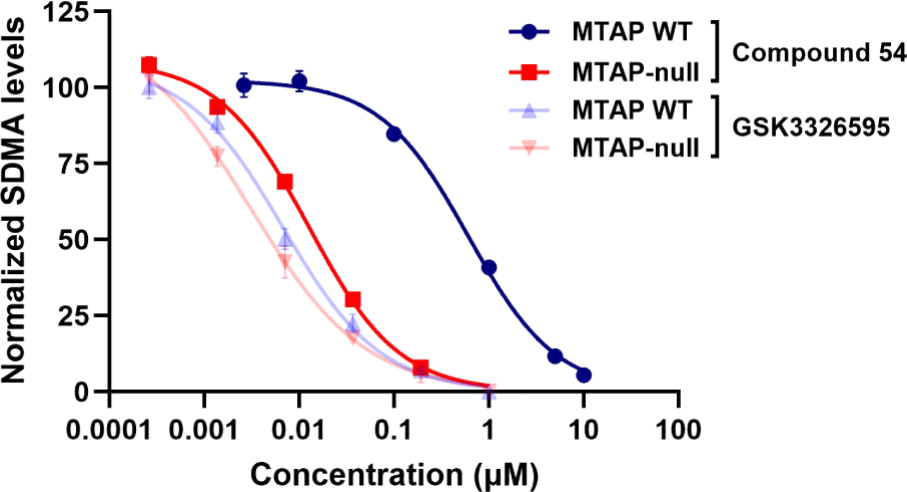
In cell western SDMA assay data for 54 are compared to GSK3226595 in *MTAP*-del and MTAP WT cells. 54 is equipotent to GSK3226595 in *MTAP*-del cells but is selective relative to MTAP WT cells.

**Compounds 51–54** represent compounds in the series that achieved good SDMA ICW potency and selectivity (MTAP-null ICW IC_50_ = 0.01–0.1 μM with >30-fold selectivity over MTAP WT cells). The ICW potency of **54** in MTAP-null cells approached that of SAM-cooperative clinical compound GSK3326595 (pemrametostat) and the ICW dose-response curves in MTAP-null vs MTAP WT cells for these two compounds demonstrate the on-target, on-mechanism selectivity of **54** (Figure 9).

The selective pharmacodynamic activity of these compounds translated into selective viability potency in MTAP-null cells as well (MTAP-null viability GI_50_ = 0.1–3.3 μM with ≥5× selectivity over MTAP WT cells). As we have observed previously, the viability GI_50_ correlated with the PD IC_90_, suggesting that >90% PRMT5 inhibition is required to achieve cellular viability effects. Compounds **52** and **54** demonstrated significant selectivity at the PD IC_90_ (>11-fold and 24-fold, respectively) but each only demonstrated 5-fold selectivity in the viability assay suggesting the presence of off-target activity at higher concentrations. In comparison, compounds **51** and **53** maintained similar selectivity over MTAP WT cells in both the PD and viability assays, suggesting the cellular viability effects in MTAP-null cells were on target.

**Figure 10:**
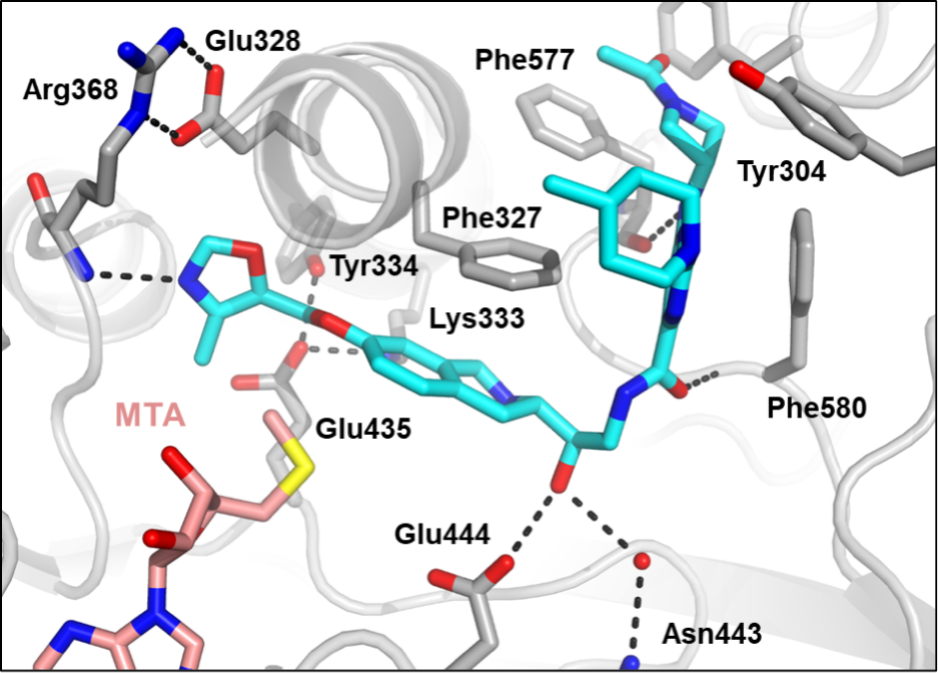
X-ray crystal structure of **51** (blue, PDB entry 9MGR) in PRMT5•MTA.

**Figure 11:**
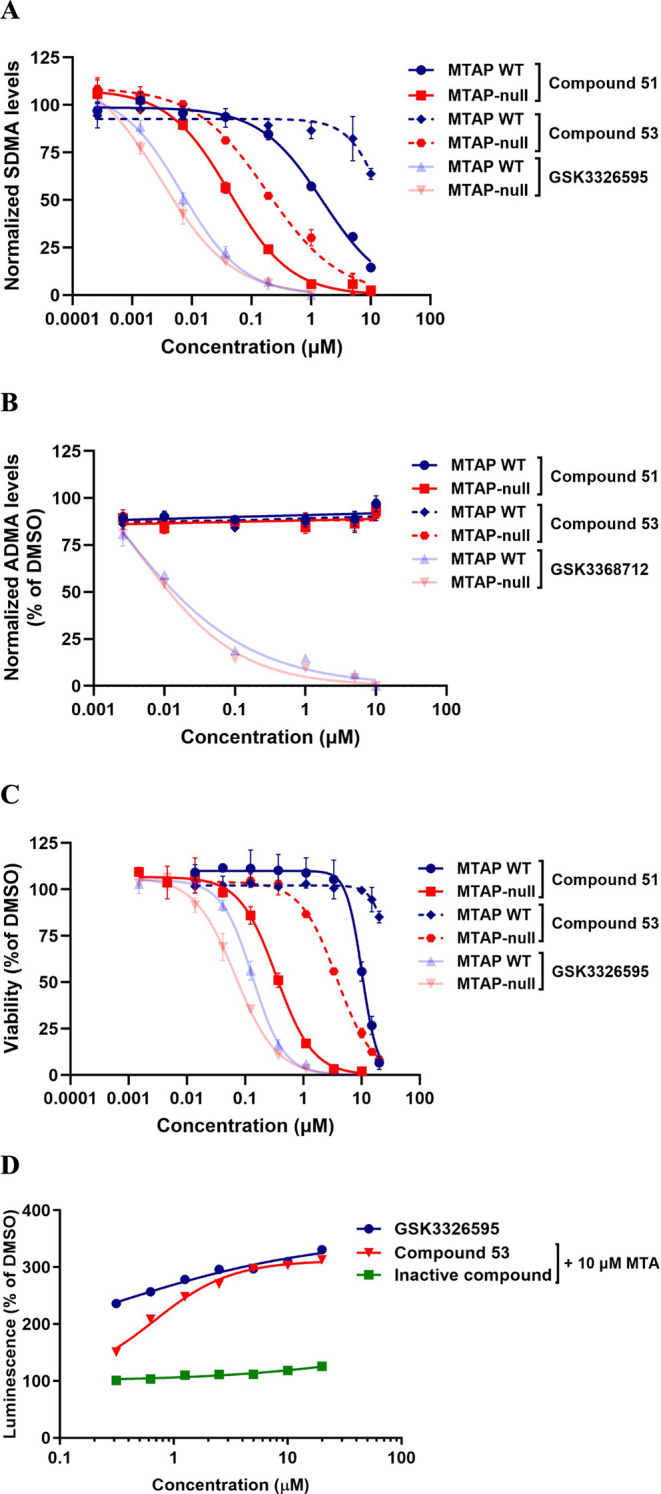
*In vitro* data: (A) SDMA in cell western (ICW) PD data for HAP1MTAP-null vs MTAP WT cells for 51 and 53. and (B) ADMA ICW PD data for HAP1MTAP-null vs MTAP WT cells for 51 and 53. GSK3368712 is a type I PRMT inhibitor and included as an assay control. (C) Cellular viability data in HAP1 MTAP-null vs MTAP WT cells for 51 and 53. (D) Thermostability target engagement in HAP1 MTAP-null cells engineered to stably express an exogenous NanoLuc-PRMT5 protein for 53.

A crystal structure of **51** was obtained (Figure 10), confirming that the C-linked THIQ compounds bind in the same manner as the N-linked compounds and the oxazole extension achieves a hydrogen bond with Arg368. Key assay data for **51** and **53** are shown in Figure 11, including selective PD activity (Figure 11A), selectivity against Type I PRMTs in an ADMA ICW assay, using GSK3368712, a Type I PRMT inhibitor as a control (Figure 11B), selective viability effects in *MTAP*-isogenic cells (Figure 11C), and confirmation of target engagement with a NanoLUC thermostability assay for **53** (Figure 11D). These data collectively support the conclusion that these compounds achieve selective on-target activity in MTAP-null cells and that potency and selectivity are maintained in all assays in comparison to GSK3326595, a SAM-uncompetitive PRMT5 inhibitor.

To further illustrate MTA-cooperativity in the series, we compared the activity with PRMT5•MTA relative to apo-PRMT5 in the peptide displacement assay. The data for several examples are summarized in Table 7.

**Table 7:** Biochemical Data with PRMT5•MTA and apo-PRMT5 Demonstrating MTA-cooperativity^a^^b^

	PRMT5 Peptide Displacement *K*_i,app_ (μM)^a^		
Compound	+ MTA	APO	Degree of cooperativity
24	0.04	11.3	280×
40	0.08	5.5	69×
49	<0.004	0.02	>5×
51	<0.004	0.03	>7×
52	<0.004	0.05	>12×
53	<0.004	0.04	>10×
54	<0.004	0.02	>5×

a Detection of displacement of a TAMRA-labeled peptide by fluorescence anisotropy. *K*_i, app_ calculated from IC_50_ values using the Cheng–Prusoff equation.

b Racemic.

The series consistently showed greater activity with PRMT5•MTA versus apoPRMT5. Earlier compounds, represented by **24** and **40**, showed strong cooperativity with a ratio of activity between apoPRMT5 and PRMT5•MTA of 280- and 60-fold, respectively. Significant selectivity for PRMT5•MTA versus apoprotein was also observed in SPR binding experiments with these compounds (Figure SI-1 and Table SI-1). As the molecules were made more potent, exemplified by **49**–**54**, the detection limit of the PRMT5•MTA assay was reached, and absolute ratios could not be calculated. SPR experiments similarly encountered limitations in *K*_d_ determination for the most potent compounds owing to the fact that *k*_off_ values could not be measured accurately (Figure SI-2). Overall, considering the cellular activity and selectivity, we believe that the degree of cooperativity is significant.

In general, the drug-like properties of these compounds were poor, with high MW, PSA, hydrogen bond donors, rotatable bonds, etc. A selection of compounds, as exemplified by **53**, was evaluated further, and these compounds showed poor metabolic stability with high intrinsic clearance (CL_int,mic_ = 408 μL/min/mg) in a human liver microsomal preparation. Permeability (MDCKII Mdr1 A-B < 0.2 × 10^–6^ cm/s) and efflux (MDCKII Mdr1 efflux ratio >16) were also poor. Further effort was made, including incorporation of the cyclized chain modification exemplified in **46a**, to improve the potency and properties of these compounds by modifying, morphing, and/or truncating the molecules, but no significant improvement was achieved. Nevertheless, these compounds became useful tools to establish proof of concept that selective, MTA-cooperative inhibition of PRMT5 could be achieved in cells and that this inhibition leads to selective killing of MTAP-null cells while sparing normal cells. They also proved useful in advancing early understanding of biology in our internal program that led to the discovery of TNG908 and TNG462, which are both in Phase 1/2 clinical studies.^28,29^

## Synthesis

**Scheme 1:**
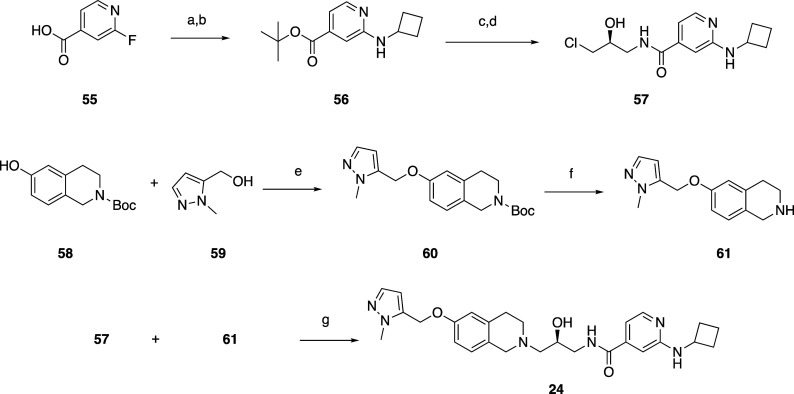
Synthesis of Compound 24 Reagents and conditions: (a) Boc_2_O, DMAP, MTBE. (b) Cyclobutylamine, K_2_CO_3_, DMF, 100 °C. (c) HCl, EtOAc, 50 °C. (d) (*S*)-1-Amino-3-chloropropan-2-ol hydrochloride, HATU, DIPEA, DCM, DMF. (e) PPh_3_, DIAD, toluene, 20–90 °C. (f) 4 M HCl, dioxane. (g) DIPEA, NaI, DBU, ACN.

A robust synthesis of C6 ether-substituted N-linked tetrahydroisoquinolines was developed, and a representative example for **24** is shown in Scheme 1. Analogs with other connectivity could be made similarly, after formation of the appropriately substituted THIQ. 2-Fluoroisonicotinic acid **55** was converted to its *t*-butyl ester with Boc_2_O and DMAP in MTBE, followed by displacement of the fluorine with cyclobutylamine to give aminopyridine **56**. Following ester hydrolysis with HCl in ethyl acetate, the acid was coupled to (*S*)-1-amino-3-chloropropan-2-ol hydrochloride in dichloromethane and dimethylformamide with HATU and DIPEA to give chloride **57**. Meanwhile, Boc-protected 1,2,3,4-tetrahydroisoquinolin-6-ol **58** was reacted with alcohol **59**, DIAD, and triphenylphosphine in toluene to yield ether **60**. Boc removal with HCl yielded amine **61**, which was coupled to chloride **57** in the presence of DIPEA, NaI, and DBU in acetonitrile, yielding the final compound **24**. Other ether analogs were prepared similarly or through the coupling of the appropriate chloride with cesium carbonate in DMF.

**Scheme 2:**
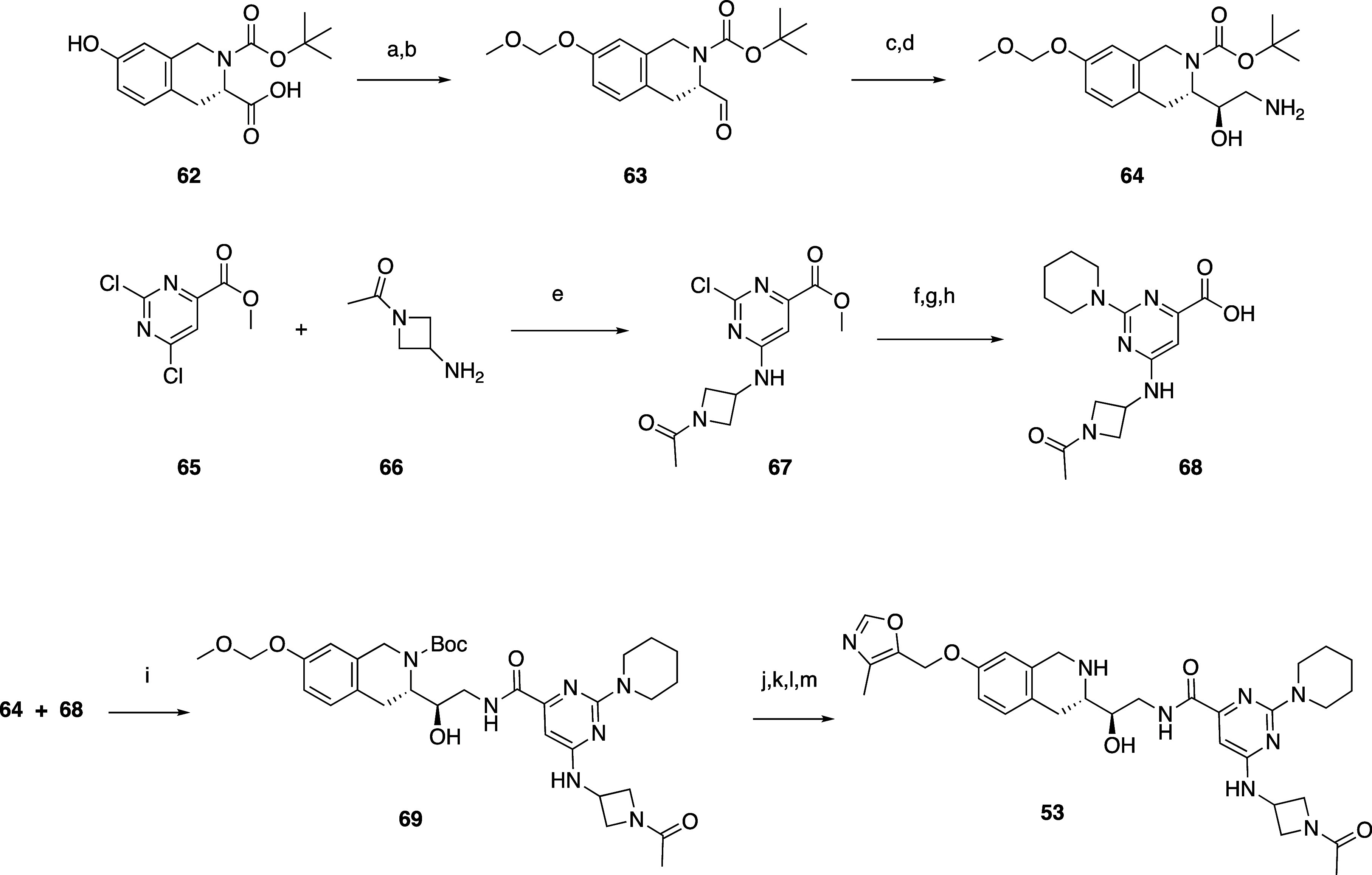
Synthesis of Compound 53 Reagents and conditions: (a) methoxymethyl chloride, cesium carbonate, DMF. (b) DIBAL-H, MeOH, DCM. (c) MeNO_2_, (1*R*, 2*R*)-*N*1,*N*2-bis[(4-chlorophenyl)methyl]cyclohexane-1,2-diamine copper(II) acetate hydrate, EtOH, MTBE. (d) 10% Pd/C, MeOH. (e) TEA, DCM. (f) piperidine, TEA, ACN. (g) LiOH•H_2_O, MeOH, THF, H_2_O. (h) AcCl, TEA, ACN. (i) HATU, TEA, DMF. (j) 4 M HCl, dioxane. (k) NaHCO_3_, Boc_2_O, THF, H_2_O. (l) 5-(chloromethyl)-4-methyloxazole, Cs_2_CO_3_, DMF. (m) 4 M HCl, dioxane.

A representative synthesis from the 7-substituted C-linked THIQ series is shown in Scheme 2 (**53**). Boc-protected (*S*)-7-hydroxy-1,2,3,4-tetrahydroisoquinoline-3-carboxylic acid **62** was MOM-protected followed by reduction of the resultant ester to aldehyde **63** with DIBAL-H in methanol and dichloromethane. Homologation with MeNO_2_ and reduction with 10% Pd/C gave **64**. Substituted pyrimidine acid **68** was prepared by 6-chloro displacement of methyl 2,6-dichloropyrimidine-4-carboxylate **65** with 1-(3-aminoazetidin-1-yl)ethan-1-one **66** in the presence of triethylamine in dichloromethane to give **67**, followed by 2-chloro displacement with piperidine. Hydrolysis of the ester with LiOH•H_2_O led to partial removal of the acetyl group, and this was reinstalled with acetyl chloride in the presence of triethylamine in acetonitrile. Coupling of amine **64** and acid **68** with HATU and triethylamine in dimethylformamide led to **69** that was deprotected with HCl. The amine was protected with Boc_2_O, the phenol was reacted with 5-(chloromethyl)-4-methyloxazole, and the Boc group was removed to give final ether compound **53**.

**Scheme 3:**
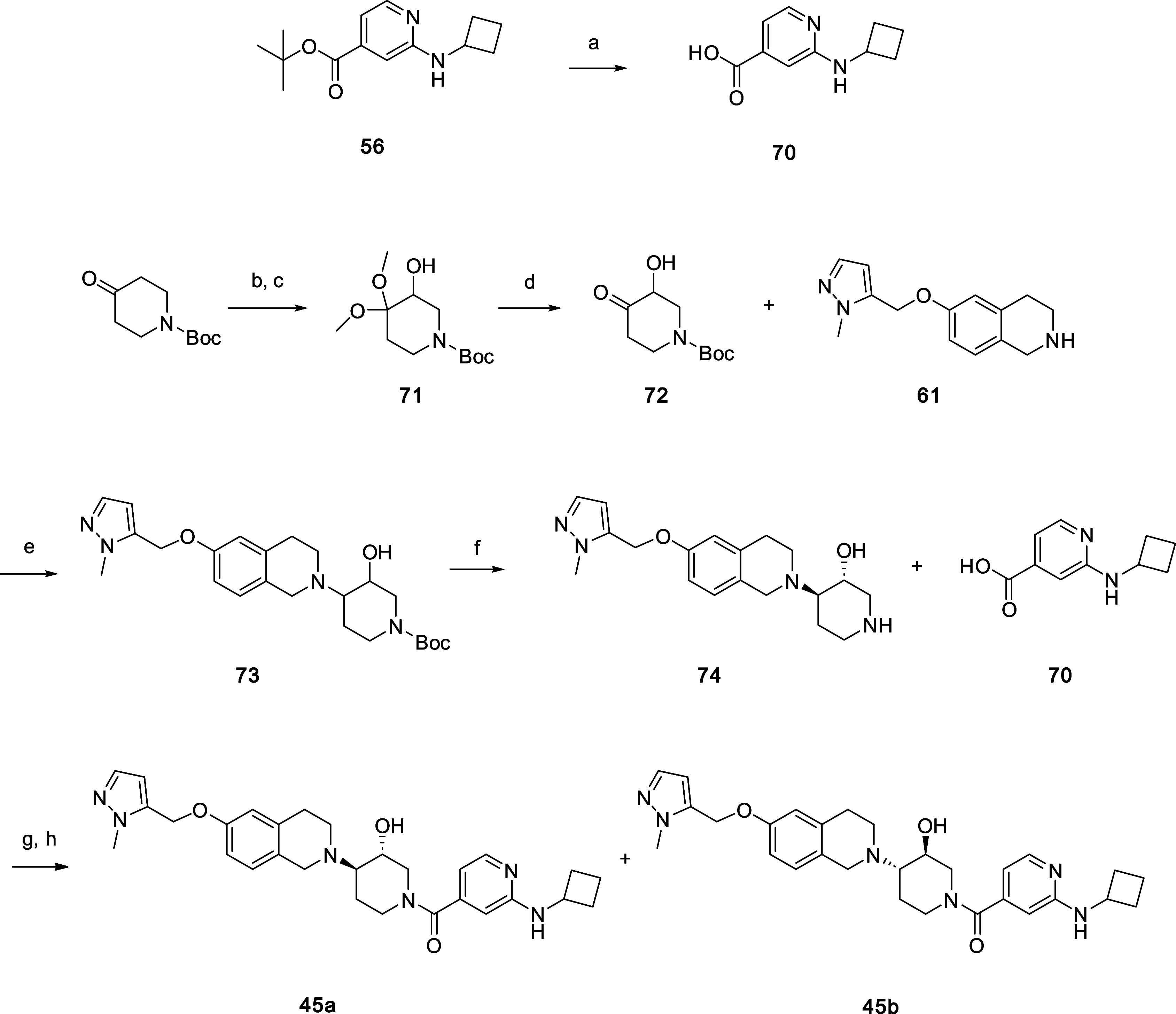
Synthesis of Compounds 45a and 45b Reagents and conditions: (a) HCl, EtOAc, 50 °C. (b) KOH, MeOH, 0 °C. (c) I_2_, MeOH, 0–20 °C. (d) p-TsOH•H_2_O, toluene. (e) NaBH(OAc)_3_, THF. (f) 4 N HCl, EtOAc, DCM. (g) HATU, DIPEA, DCM, DMF. (h) Chiral SFC separation.

Compounds **45a** and **45b** were prepared starting with *tert*-butyl 4-oxopiperidine-1-carboxylate which was protected as the ketal and hydroxylated to **71**, followed by deprotection with *p*-TsOH in toluene to yield hydroxyketone **72**. Reductive amination with **61** provided **73**, which was deprotected with HCl to give amine **74**. Meanwhile **56**, was deprotected with HCl to **70** which was coupled with **74** in the presence of HATU and DIPEA in DCM and DMF to yield racemic amide which was separated by chiral SFC chromatography to give **45a** and **45b (**Scheme 3).

**Scheme 4:**
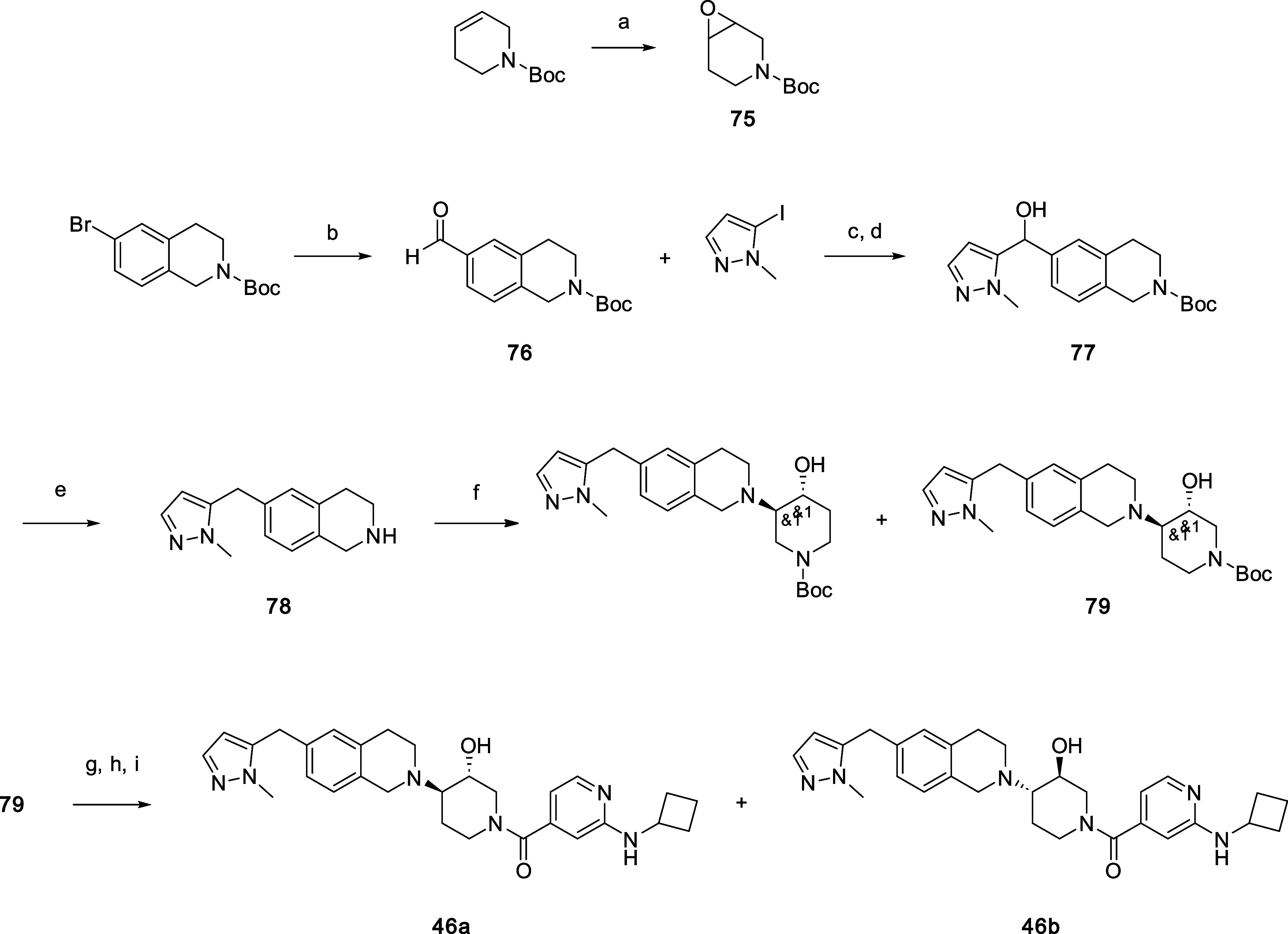
Synthesis of Compounds 46a and 46b Reagents and conditions: (a) mCPBA, DCM. (b) CO (50 psi), Pd(dppf)Cl_2_, TEA, TES, DMF, 80 °C. (c) nBuLi, hexanes, THF, −78 °C. (d) THF, −78 °C–20 °C. (e) Pd/C, H_2_ (15 psi), HCl/H_2_O, tBuOH, 70 °C. (f) 75, TEA, EtOH, 100 °C. (g) TFA, DCM. (h) 70, HATU, DIPEA, DCM, DMF. (i) Chiral SFC separation.

Compounds **46a** and **46b** were prepared starting from *tert*-butyl 6-bromo-3,4-dihydroisoquinoline-2(1*H*)-carboxylate which was formylated with CO in the presence of Pd(dppf)Cl_2_, TEA, and TES in DMF at 80 °C and at 50 psi pressure to give aldehyde **76**. 5-Iodo-1-methyl-1*H*-pyrazole was treated with *n*BuLi-hexanes in THF at −78 °C to generate the lithiated species to which **76** was added in THF at −78 °C and warmed to −20 °C to give alcohol **77** which was reduced with Pd/C under H_2_ atmosphere in the presence of HCl/H_2_O in tBuOH at 70 °C to yield **78**. Meanwhile, *tert*-butyl 3,6-dihydropyridine-1(2*H*)-carboxylate was converted to the corresponding epoxide, **75**, with *m*CPBA, and this was opened with **78** to yield Boc-protected amino-alcohol **79** and the regioisosteric side product. After separation, **79** was deprotected with TFA in DCM, and then reacted with **75** via HATU coupling to give the racemic amide, which was separated into enantiomers **46a** and **46b** by chiral SFC chromatography (Scheme 4).

## Conclusion

In summary, a rationally designed mechanism switch was achieved in a series of compounds that selectively inhibit the PRMT5•MTA complex relative to the PRMT5•SAM complex and selectively kill *MTAP*-deleted cancer cells relative to MTAP WT cells. These compounds were designed to achieve this profile by modifying SAM-cooperative PRMT5 inhibitors to enable them to fill the portion of the SAM cofactor binding pocket that normally accommodates the methionine side chain of SAM and is vacant when MTA is bound. By growing the inhibitors into this pocket and achieving a hydrogen bond with Arg368, potency against the PRMT5•MTA was improved, and competition with SAM led to switching of selectivity. These compounds provided a proof of concept that *MTAP*-deleted cancers could be targeted with MTA-cooperative PRMT5 inhibitors. Furthermore, they were also important chemical tools for the advancement of biological understanding as we transitioned to hit finding that eventually led to the discovery of TNG908 and TNG462.

## Experimental Section

### General Procedures

All chemicals were provided by Enamine Ltd., WuXi AppTec, or other commercial suppliers and used as received unless otherwise indicated. All solvents were treated according to standard methods. All reactions were monitored by LC-MS analysis using Agilent 1260 LC/MSD instruments, with an Agilent Poroshell 120 SB-C18 4.6 × 30 mm, 2.7 μm column; column temperature: 60 °C; mobile phase: Α—water (0.1% formic acid), Β—acetonitrile (0.1% formic acid), flow rate: 1.5 mL/min, gradient: 0.01 min −1% B, 5.00 min −100% B, 5.99 min −100% B, MS ionization mode: electrospray ionization (ESI), MS scan range: 83–1000 *m*/*z*, UV detection: 215 nm, 254 nm, 280 nm unless otherwise specified. Thin-layer chromatography (TLC) with precoated silica gel GF254 (0.2 mm) was used, and the results were visualized using either UV light or KMnO_4_ stain. Proton nuclear magnetic resonance (1H-NMR) spectra were recorded at 400, 500, or 600 MHz on Varian or Bruker instrumentation; chemical shifts were calibrated using residual nondeuterated solvents CDCl_3_ (δ = 7.26 ppm), DMSO-*d*_*6*_ (δ = 2.50 ppm), or MeOH-*d*_*4*_ (δ = 3.31 ppm) and expressed in δ ppm. Coupling constants (J), when given, are reported in hertz (Hz). Multiplicities are reported using the following abbreviations: s = singlet, d = doublet, dd = doublet of doublets, *t* = triplet, q = quartet, *m* = multiplet (range of multiplets is given), br = broad signal, and dt = doublet of triplets. All final compounds were purified by reverse-phase high-performance liquid chromatography (HPLC) or supercritical fluid chromatography (SFC) or silica gel chromatography (100–200 mesh). HPLC was done with an Agilent 1260 HPLC instrument (Agilent Technologies, Germany) equipped with a G7161A Preparative Binary Pump, a G7157A Prep Autosampler, a G7115A DAD WR, and a G7159B Preparative Fraction Collector unless otherwise noted. The Open Lab CDS software (version C.01.10) was used for instrument control, data acquisition, and data handling. SFC was done with a Waters 100q Prep SFC System unless otherwise noted. Chiral HPLC analytical analysis was done with an Agilent 1200 HPLC instrument (Agilent Technologies, Germany) equipped with a G1379B degasser, a G1312A Binary Pump, a G1329A ALS autosampler, and a G1315A diode array detector unless otherwise noted. Chiral SFC analytical analysis was done with an Agilent 1260 SFC instrument (Agilent Technologies, Germany) equipped with a G1379B degasser, a G1312B Binary Pump, a G1313A ALS autosampler, a G1316A thermostated column compartment, a G1315D Diode Array Detector, and an Aurora SFC system unless otherwise noted. Optical rotation was measured with a Polarimeter Anton Paar GmbH MCP 300 (accuracy: ± 0.003°), which is used to measure the angle of optical rotation. Standard conditions for analysis: solution concentration 0.25 g/100 mL (methanol solvent), wavelength 589 nm, temperature 21 °C. All compounds are >95% pure by HPLC.

### Biochemical Fluorescence Anisotropy Peptide Displacement Assay

A fluorescence anisotropy (FA) assay was established to measure binding of C-terminal 5′-TAMRA-labeled histone H4 peptide (1–21) with PRMT5/MEP50. The test compound competes with the peptide to bind to the PRMT5/MEP50 protein and thus would act to disrupt binding by the labeled histone H4 peptide. The assay buffer consisted of 30 mM Bicine (pH 8.0), 150 mM NaCl, 1.5 mM DTT, and 0.003% Tween-20. The two peptides were utilized for these studies were Me_0_:Ac-SGRGKGGKGLGKGGAKRHRKV-K(5-TAMRA)-NH_2_ and Me_2_:Ac-SGR(Sym Me_2_)GKGGKGLGKGGAKRHRKV-K(5-TAMRA)-NH_2_. Me_0_ peptide is not methylated and used to determine the compound potency in the presence of 50 μM 5′-methylthioadenosine (MTA). Its *K*_D_ with PRMT5•MTA = 4.6 nM. Me_2_ peptide is symmetrically methylated at Arginine 3 and used to determine the compound potency in the presence of 50 μM S-adenosyl methionine (SAM) and its *K*_D_ with PRMT5•SAM = 79 nM. Inhibitor potency was assessed at equilibrium by measuring the dose-dependent displacement of a fixed concentration of the peptide from PRMT5/MEP50. Following incubation at room temperature for 30 min, the plate was read on an Envision plate reader. For data analysis, fluorescence anisotropy (FA) detected equals 1000 ∗ (S – G ∗ P)/(S + G ∗ 2 ∗ P) where S = detector 2 or channel 2 signal, *p* = detector 1 or channel 1 signal, and G = G-factor. Fluorescence anisotropy is normalized to %inhibition using %inhibition = (Signal – MinAVG)/(MaxAVG – MinAVG) ∗ 100, where MinAVG = the average value of Min value and MaxAVG = the average value of Max value. Curves are fitted by XL-Fit as %inhibition vs log [compound concentration] using a 4-parameter logistic equation with fixed 0% and 100% inhibition limits. The Cheng–Prusoff equation for a competitive inhibitor *K*_i,app_ = IC_50_/(1 + [Peptide probe]/*K*_D_) was applied, where [Peptide probe] = the concentration of the H4 peptide probe used in the assay. *K*_D_ is the equilibrium dissociation constant of peptide probe, representing the peptide probe concentration at which 50% of the proteins are bound to the probes at equilibrium.

### HAP1 MTAP WT and MTAP-null In-Cell Western Assays

A HAP1 *MTAP*-isogenic cell line pair was acquired from Horizon Discovery (HZGHC004894c005) and maintained in DMEM (high glucose) + 10% FBS in a humidified, 10% CO_2_ tissue culture incubator. The SAM-cooperative PRMT5 inhibitor, GSK3326595, or the type I PRMT inhibitor, GSK3368712, were sourced from Selleck Chemicals and maintained as 10 mM DMSO stocks. All test compounds are maintained as 10 mM DMSO stocks.

On Day 0, MTAP WT or MTAP-null cells are seeded in a 384-well plate and incubated in a humidified, 5% CO_2_ tissue culture incubator for 16–24 h. On Day 1, the test compounds are dispensed to wells at defined concentrations using a Tecan D300e digital dispenser (*n* = 4), and the volume of DMSO is normalized to the highest class volume. Each plate includes wells dosed with defined concentrations of GSK3326595 in the SDMA ICW assay or GSK3368712 in the ADMA ICW assay as a plate control. The compounds are incubated with cells for 24 h in a humidified, 5% CO_2_ tissue culture incubator.

On Day 2, the compound-treated cells are fixed with a final concentration of 4% formaldehyde. The cells are then washed/permeabilized with 1× PBS + 0.1% Triton X-100, and then blocked with 5% goat serum/1× TBS. The fixed cells are then incubated overnight at 4 °C with a primary SDMA antibody cocktail in the SDMA ICW assay (Cell Signaling 13222) or a primary ADMA antibody cocktail in the ADMA ICW assay (Cell Signaling 13522).

On Day 3, the cells are washed with 1× PBS + 0.1% Triton X-100 and then incubated at room temperature for 1 h with a NIR fluorescent secondary antibody cocktail that also contained DRAQ5 (LiCor 926-32211 and VWR 10761-508). The cells are washed with 1× PBS + 0.1% Triton X-100 and then washed again with ddH_2_O. The plates are then imaged using a NIR fluorescent imager (LiCor Odyssey).

For data analysis, the SDMA or ADMA signal is normalized to the DRAQ5 signal. Assay background is determined by the signal from wells treated with 1 μM GSK3326595 or GSK3368712 and subtracted from every well. The data are plotted as % of the DMSO control wells for the MTAP WT and the MTAP-null cell lines independently and fitted to the four-parameter logistic (4-PL) Hill equation with maximal effect constrained to 0. The fit was performed using GraphPad Prism or the default IC_50_ fitting procedure in Dotmatics Studies 5.4 as part of a customized data analysis protocol.

### HAP1 MTAP WT and MTAP-null Viability Assay

A HAP1 *MTAP*-isogenic cell line pair was acquired from Horizon Discovery (HZGHC004894c005) and maintained in DMEM (high glucose) + 10% FBS in a humidified, 5 or 10% CO_2_ tissue culture incubator. All test compounds are maintained as 10 mM DMSO stocks.

On Day 0, MTAP WT and MTAP-null cells are seeded in a 96-well plate and incubated in a humidified, 5 or 10% CO_2_ tissue culture incubator for 16–24 h. On Day 1, the test compounds are dispensed to wells at defined concentrations using a Tecan D300e digital dispenser (*n* = 3), and the volume of DMSO is normalized to highest class volume (0.2%). The compound-treated plates are incubated for 7 days in a humidified, 5 or 10% CO_2_ tissue culture incubator.

On Day 7, the plates are removed from the tissue culture incubator and allowed to equilibrate to room temperature. Then either a 1/2 volume CellTiter-Glo Luminescent Cell Viability Assay reagent (Promega G7572) is added to each well, or the media is removed from every well and a 1:3 dilution of CellTiter-Glo 2.0 Cell Viability Assay reagent (Promega G9241) in 1× PBS is added. Ten minutes after addition, the luminescent signal is detected by an Envision plate reader. The data are plotted as % of the DMSO control wells for the MTAP WT and the MTAP-null cell lines independently and fitted to the 4-parameter logistic (4-PL) Hill equation with maximal effect constrained to 0. The fit was performed using GraphPad Prism or the default IC50 fitting procedure in Dotmatics Studies 5.4 as part of a customized data analysis protocol.

### Cellular Luciferase-Based Thermostability Assay

HAP1 *MTAP*-deleted cells were engineered to express exogenous PRMT5 fused to an N-terminal NanoLuc luciferase construct, and the endogenous PRMT5 gene was knocked out using CRISPR-based editing. The stable cell line was harvested by trypsinization, washed with 1× PBS, and resuspended in PRMT5 Activity Buffer (Bicine at 30 mM, Tween-20 at 0.003%, NaCl at 150 mM, Q.S. w/Water) supplemented with 1× Halt protease inhibitor cocktail (ThermoFisher #78446). Cells were counted and aliquoted into separated PCR tubes at 1.0 × 10^6^ cells/mL and were lysed by repeated freeze–thaw cycles (liquid nitrogen for 3 min followed by 15 min in a room temperature water bath × 3 cycles). Lysates were centrifuged for 20 min, the supernatants were transferred to PCR tubes, and compounds were dosed as indicated for 30 min. Lysates were supplemented with 10 μM MTA when profiling MTA-cooperative PRMT5 inhibitors. Supplementation with exogenous SAM was deemed unnecessary for GSK3326595 as PRMT5 stability was ∼300% at 10 μM GSK3326595 + 10 μM SAM in an independent study, which is the equivalent maximum effect determined with the conditions used here. Samples were then heated for 3 min and cooled to RT using a Bio-Rad T100 PCR thermal cycler. Individual wells were transferred to a 96 well white clear bottom plate at 25 μL per well and mixed with 25 μL of RT equilibrated Nano-Glo Luciferase substrate (#N1120) for 5 min. Plates were read on an Envision luminometer.

#### *tert*-butyl 2-(cyclobutylamino)isonicotinate (**56**)

To a solution of 2-fluoropyridine-4-carboxylic acid (20 g, 141.74 mmol, 1 *eq*) and DMAP (3.46 g, 28.35 mmol, 0.2 *eq*) in THF (150 mL) was added (Boc)_2_O (68.0 g, 311.57 mmol, 2.2 *eq*) at 0 °C. The mixture was stirred at 25 °C for 12 h. The resulting mixture was quenched by addition of NaHCO_3_ (20 mL) and extracted with EtOAc (150 mL × 3). The combined organic layer was dried over anhydrous Na_2_SO_4_, filtered, and concentrated under reduced pressure to afford *tert*-butyl 2-fluoropyridine-4-carboxylate (37 g, crude), which was used in the next step without further purification. ^1^H NMR (400 MHz, CDCl_3_) *δ* 8.30 (d, *J* = 5.0 Hz, 1 H), 7.67 (dt, *J* = 5.0, 1.5 Hz, 1 H), 7.40 (d, *J* = 1.0 Hz, 1 H), 1.58 (s, 9 H); LCMS (M+H^+^) *m*/*z*: calc'd 198.1; found 198.1. To a mixture of *tert*-butyl 2-fluoropyridine-4-carboxylate (70.87 mmol, crude product, 1 *eq*), cyclobutanamine (15 mL, 175.05 mmol, 2.5 *eq*) in DMF (150 mL) was added K_2_CO_3_ (29.38 g, 212.61 mmol, 3 *eq*). The mixture was stirred at 110 °C for 12 h (2 batches in parallel). The resulting mixture was filtered and diluted with EtOAc (100 mL × 3). The organic layers were washed with brine (150 mL × 3), dried over anhydrous Na_2_SO_4_, filtered, and concentrated under reduced pressure. The residue was purified by column chromatography (silica, petroleum ether/EtOAc = 30:1 to 10:1) to afford **56** (12 g, 31% in two steps) as a yellow oil. ^1^H NMR (400 MHz, CDCl_3_) *δ* 8.15 (d, *J* = 5.0 Hz, 1 H), 7.03 (dd, *J* = 5.3, 1.3 Hz, 1 H), 6.84 (s, 1 H), 4.85 (d, *J* = 6.0 Hz, 1 H), 4.10–4.23 (m, 1 H), 2.41–2.51 (m, 2 H), 1.69–1.94 (m, 4 H), 1.59 (s, 9 H); LCMS (M+H^+^) *m*/*z*: calc'd 249.2; found 249.0.

#### (S)-*N*-(3-chloro-2-hydroxypropyl)-2-(cyclobutylamino)isonicotinamide (**57**)

A solution of **56** (12 g, 43.49 mmol, 1 *eq*) in HCl/EtOAc (250 mL, 4 M) was stirred at 50 °C for 12 h. The precipitate was collected by filtration, washed with EtOAc (30 mL × 3), and dried under high vacuum to afford 2-(cyclobutylamino)isonicotinate (9.9 g, HCl salt, 99% yield) as a yellow solid. ^1^H NMR (400 MHz, DMSO-*d*_*6*_) *δ* 9.27 (br s, 1 H), 7.99 (d, J = 6.3 Hz, 1 H), 7.41 (s, 1 H), 7.06 (dd, J = 6.5, 1.3 Hz, 1 H), 4.29 (t, J = 7.5 Hz, 1 H), 2.37–2.46 (m, 2 H), 1.94–2.07 (m, 2 H), 1.65–1.85 (m, 2 H); LCMS (M+H+) *m*/*z*: calc'd 193.1; found 193.4. To a mixture of 2-(cyclobutylamino)isonicotinate (8.5 g, 37.17 mmol, 1 equiv, HCl) in DCM (130 mL) and DMF (35 mL) were added HATU (21.20 g, 55.76 mmol, 1.5 equiv) and DIPEA (185.85 mmol, 32.37 mL, 5 equiv) sequentially. After stirring for 15 min, (*S*)-1-amino-3-chloropropan-2-ol (5.97 g, 40.89 mmol, 1.1 eq, HCl) was added, and the mixture was stirred for 12 h at 25 °C. The reaction mixture was concentrated under reduced pressure to remove DCM. The residue was diluted with H_2_O (150 mL) and extracted with EtOAc (70 mL × 4). The combined organic layers were washed with saturated NH_4_Cl aqueous solution (100 mL), brine (100 mL), dried over anhydrous Na_2_SO_4_, filtered, and concentrated under reduced pressure. The residue was purified by flash chromatography (ISCO; 40 g SepaFlash Silica Flash Column, petroleum ether/EtOAc with EtOAc from 0–95%, flow rate = 35 mL/min) to afford **57** (12.1 g, 92% yield) as a yellow solid. ^1^H NMR (400 MHz, MeOH-*d*_*4*_) *δ* 7.98–8.05 (m, 1 H), 6.79–6.88 (m, 2 H), 4.25 (quin, J = 7.8 Hz, 1 H), 3.95–4.04 (m, 1 H), 3.53–3.70 (m, 3 H), 3.41–3.48 (m, 1 H), 2.35–2.45 (m, 2 H), 1.89–1.98 (m, 2 H), 1.76–1.85 (m, 2 H); LCMS (ESI) [M + H]+ calc'd 284.1, found 284.4.

#### *tert*-butyl 6-((1-methyl-1*H*-pyrazol-5-yl)methoxy)-3,4-dihydroisoquinoline-2(1*H*)-carboxylate (**60**)

To a solution of *tert*-butyl 6-hydroxy-3,4-dihydro-1H-isoquinoline-2-carboxylate (500 mg, 2.01 mmol), (2-methylpyrazol-3-yl)methanol (340 mg, 3.03 mmol), and PPh_3_ (800 mg, 3.05 mmol) in toluene (10 mL) was added a solution of DIAD (610 mg, 3.02 mmol) in toluene (4 mL) slowly at 20 °C. After addition was complete, the mixture was warmed to 90 °C and stirred for 12 h under nitrogen. The resulting mixture was concentrated under reduced pressure, and the residue was purified by flash chromatography (ISCO; 40 g flash silica column, petroleum ether/EtOAc with EtOAc from 0–50%, flow rate: 40 mL/min) to afford **60** (640 mg, 93% yield) as a yellow oil. LCMS (ESI) [M+H-56]+ *m*/*z* calc'd 288.2, found 288.1.

#### 6-((1-methyl-1*H*-pyrazol-5-yl)methoxy)-1,2,3,4-tetrahydroisoquinoline (**61**)

To a solution of **60** (640 mg, 1.86 mmol) in DCM (10 mL) was added TFA (13.0 mmol, 1 mL), and the mixture was stirred for 2 h at 20 °C. The resulting mixture was concentrated under reduced pressure and dissolved in MeOH (5 mL), followed by the addition of K_2_CO_3_ solid (∼1 g). The mixture was stirred overnight at 20 °C. The solvent was removed under reduced pressure, and the residue was dissolved in brine (15 mL). The aqueous layer was extracted with DCM (30 mL × 15), and the combined organic phase was dried over anhydrous Na_2_SO_4_, filtered, and concentrated in a vacuum to afford **61** (420 mg, 93% yield) as a red oil which was used in the next step directly. ^1^H NMR (400 MHz, MeOH-*d*_4_) *δ* 7.40 (d, *J* = 1.9 Hz, 1 H), 6.98 (d, *J* = 8.4 Hz, 1 H), 6.77–6.86 (m, 2 H), 6.36 (d, *J* = 2.0 Hz, 1 H), 5.10 (s, 2 H), 3.93 (s, 2 H), 3.88 (s, 3 H), 3.35 (s, 2 H), 3.09 (t, *J* = 6.1 Hz, 2 H), 2.80–2.86 (m, 2 H); LCMS (ESI) [M + H]^+^*m*/*z* calc'd 244.1, found 244.1; HPLC: 84.2% @ 220 nm.

#### (S)-2-(cyclobutylamino)-*N*-(2-hydroxy-3-(6-((1-methyl-1*H*-pyrazol-5-yl)methoxy)-3,4-dihydroisoquinolin-2(1*H*)-yl)propyl)isonicotinamide (**24**)

To a sealed tube were added **61** (40 mg, 0.164 mmol), **57** (45.0 mg, 0.159 mmol), TEA (0.502 mmol, 70 uL), NaI (36.0 mg, 0.240 mmol), and MeCN (2 mL). The mixture was sealed and stirred for 12 h at 100 °C. The resulting mixture was filtered, washed with MeOH (10 mL), and concentrated under reduced pressure. The residue was purified by preparative HPLC (Instrument: Gilson GX-281 Liquid Handler, Gilson 322 Pump, Gilson 156 UV Detector; Column: Waters XBridge 150 × 25 mm × 5 μm; Mobile phase A: H_2_O with 0.05% NH_3_–H_2_O (v%); Mobile phase B: MeCN; Gradient: B from 28% to 58% in 7.8 min, hold 100% B for 2.5 min; Flow Rate: 25 mL/min; Column Temperature: 30 °C; Wavelength: 220 nm, 254 nm) to afford **24** (25 mg, 32% yield) as an off-white solid. ^1^H NMR (400 MHz, MeOH-*d*_*4*_) *δ* 7.90 (d, J = 5.3 Hz, 1 H), 7.41 (d, J = 1.8 Hz, 1 H), 6.98 (d, J = 8.0 Hz, 1 H), 6.77–6.84 (m, 3 H), 6.70–6.76 (m, 1 H), 6.37 (d, J = 2.0 Hz, 1 H), 5.11 (s, 2 H), 4.16–4.33 (m, 1 H), 4.01–4.14 (m, 1 H), 3.89 (s, 3 H), 3.68 (s, 2 H), 3.39–3.55 (m, 2 H), 2.77–2.94 (m, 4 H), 2.57–2.71 (m, 2 H), 2.34–2.45 (m, 2 H), 1.84–1.99 (m, 2 H), 1.70–1.83 (m, 2 H). ^1^H NMR (DMSO-*d*_*6*_, 500 MHz): *δ* 1.64 (m, 2H), 1.83 (m, 2H), 2.25 (m, 2H), 2.44 (m, 2H), 2.68 (m, 2H), 2.76 (m, 2H), 3.18 (m, 1H), 3.37 (m, 1H), 3.54 (m, 2H), 3.80 (s, 3H), 3.87 (m, 1H), 4.24 (h, 1H), 4.80 (d, 1H), 5.09 (s, 2H), 6.33 (d, 1H), 6.73 (m, 2H), 6.78 (m, 2H), 6.91 (d, 1H), 6.94 (d, 1H), 7.34 (d, 1H), 7.94 (d, 1H), 8.45 (t, 1H). ^13^C NMR (DMSO-*d*_*6*_, 126 MHz): *δ* 14.69, 28.87, 30.56, 36.40, 44.74, 46.06, 51.00, 55.49, 59.99, 62.33, 66.66, 105.69, 106.86, 108.72, 112.77, 114.15, 127.29, 127.66, 135.31, 137.30, 137.54, 142.61, 148.06, 156.03, 158.37, 165.56. LCMS (ESI) [M + H]+ *m*/*z* calc'd 491.3, found 491.2; HPLC: 100% -at 254 nm; 100% ee. [α]^21^ D = −4.40° (*c* = 0.25 g/100 mL, MeOH). Elemental analysis [calculated/found] C[66.10/64.97] H[6.99/6.56] N[17.13/17.90] and O[9.78]. HRMS (ESI, + vw ion) *m*/*z* calc'd for C_27_H_34_N_6_O_3_ [M+H^+^] 490.26924, found 490.2685.

#### *tert*-butyl (*S*)-3-Formyl-7-(methoxymethoxy)-3,4-dihydroisoquinoline-2(1*H*)-carboxylate (**63**)

(*S*)-2-*tert*-butoxycarbonyl-7-hydroxy-3,4-dihydro-1*H*-isoquinoline-3-carboxylic acid (25 g, 85.23 mmol) and cesium carbonate (83.31 g, 255.70 mmol) were mixed with DMF (250 mL). Then, methoxymethyl chloride (20.59 g, 255.70 mmol) was added dropwise while stirring. The mixture was stirred at 20 °C for 10 h. After the completion of the reaction, MTBE (1 L) was added, and the mixture was extracted with H2O (5 × 300 mL). The organic phase was separated, dried with Na_2_SO_4_ and evaporated in vacuo at 35 °C to give (*S*)-2-*tert*-butyl 3-(methoxymethyl) 7-(methoxymethoxy)-3,4-dihydroisoquinoline-2,3(1*H*)-dicarboxylate (31 g, 81.28 mmol, 95% yield), which was used without further purification in the next step. ^1^H NMR (500 MHz, CDCl_3_) *δ* 1.47 (s, 9H), 3.20 (s, 2H), 3.24 (d, 2H), 3.38 (t, 1H), 3.45 (s, 3H), 3.51 (s, 3H), 5.12 (s, 2H), 5.27 (s, 2H), 6.79 (s, 1H), 6.84 (d, 1H), 7.05 (d, 1H). LCMS (ESI): [M-Boc]+ *m*/*z*: calc'd 281.4; found 282.2; Rt = 1.48 min. (*S*)-2-*tert*-butyl 3-(methoxymethyl)-7-(methoxymethoxy)-3,4-dihydroisoquinoline-2,3(1*H*)-dicarboxylate (31 g, 81.28 mmol) was dissolved in DCM (1000 mL) and cooled to −78 °C. Then, DIBAL-H (23.12 g, 162.55 mmol, 32.98 mL) was added dropwise at the same temperature while stirring. The mixture was stirred at −78 °C for 1 h, followed by the dropwise addition of the solution of methanol (26.04 g, 812.75 mmol, 32.92 mL) in DCM (100 mL). The mixture was warmed to room temperature and carefully poured into a vigorously stirred aqueous solution of citric acid. After 15 min of vigorous stirring, the organic layer was separated, dried with Na_2_SO_4_, and evaporated in vacuo at 35 °C to give crude **63** (26 g, 80.90 mmol, 100% yield) which was used as is in the next step. ^1^H NMR (500 MHz, CDCl_3_) *δ* 1.46 (m, 9H), 3.03 (m, 2H), 3.48 (s, 3H), 4.82 (m, 3H), 5.13 (s, 2H), 6.79 (m, 2H), 7.07 (d, 1H), 9.49 (d, 1H). LCMS(ESI): [M-Boc]+ *m*/*z*: calc'd 221.4; found 222.2; Rt = 1.38 min.

#### (*S*)-*tert*-butyl 3-((*R*)-2-Amino-1-hydroxyethyl)-7-(methoxymethoxy)-3,4-dihydroisoquinoline-2(1*H*)-carboxylate (**64**)

(*1R*,*2R*)-*N*^*1*^,*N*^*2*^-bis[(4-chlorophenyl)methyl]cyclohexane-1,2-diamine (3.66 g, 10.08 mmol) and copper(II) acetate hydrate (1.68 g, 8.40 mmol) were mixed together in ethanol (150 mL) and stirred for 15 min at 20 °C. Then, the mixture was cooled to 0 °C, and a solution of **63** (27 g, 84 mmol) in ethanol (150 mL) was added. After that, nitromethane (102.57 g, 1.68 mol, 91 mL) was added in one portion. The mixture was stirred at 20 °C for 10 h. Then, the solvent was evaporated in vacuo at 35 °C. The residue was dissolved in MTBE (500 mL), and the mixture was extracted with aqueous NH_3_ (3 × 100 mL) and aqueous citric acid (3 x 100 mL). The organic phase was separated, dried over Na_2_SO_4_, and evaporated in vacuo at 35 °C. The crude product was purified by column chromatography (Interchim, 330 g SiO_2_, petroleum ether/MTBE with MTBE from 0–30%, flow rate = 127 mL/min, Rt = 35 min) to give *tert*-butyl (*3S*)-3-[(*1R*)-1-hydroxy-2-nitro-ethyl]-7-(methoxymethoxy)-3,4-dihydro-*1H*-isoquinoline-2-carboxylate (10 g, 26.15 mmol, 31% yield). ^1^H NMR (400 MHz, CDCl_3_) *δ* 1.50 (s, 9H), 2.92 (d, 1H), 3.15 (m, 1H), 3.26 (m, 1H), 3.48 (s, 3H), 4.08 (m, 1H), 4.26 (m, 2H), 4.44 (m, 2H), 4.82 (m, 1H), 5.16 (s, 2H), 6.83 (s, 1H), 6.91 (d, 1H), 7.09 (d, 1H). LCMS (ESI): [M-Boc]^+^*m*/*z*: calc'd 282.4; found 283.2; Rt = 1.38 min. *tert*-butyl (3*S*)-3-[(1*R*)-1-hydroxy-2-nitro-ethyl]-7-(methoxymethoxy)-3,4-dihydro-*1H*-isoquinoline-2-carboxylate (9 g, 23.54 mmol) was dissolved in methanol (500 mL) and palladium; 10% on carbon (1 g, 2.09 mmol) was added. The mixture was hydrogenated in an autoclave at 50 °C, 50 atm (H_2_) for 10 h. The catalyst was filtered off, and the solvent was removed in vacuo at 35 °C to give **64** (8.2 g, 23.27 mmol, 99% yield). ^1^H NMR (400 MHz, DMSO-*d*_*6*_) *δ* 1.42 (s, 9H), 2.39 (m, 1H), 2.71 (m, 1H), 3.03 (m, 1H), 3.16 (m, 2H), 3.36 (s, 3H), 4.09 (m, 2H), 4.70 (m, 1H), 5.14 (s,2H), 6.82 (m, 2H), 7.06 (d, 1H). LCMS(ESI): [M + H]+ *m*/*z*: calc'd 352.4; found 353.2; Rt = 1.07 min.

#### Methyl 6-((1-acetylazetidin-3-yl)amino)-2-chloropyrimidine-4-carboxylate (**67**)

To a solution of methyl 2,6-dichloropyrimidine-4-carboxylate **65** (3 g, 14.49 mmol) in DCM (50 mL) at 0 °C was added TEA (28.98 mmol, 4 mL) followed by 1-(3-aminoazetidin-1-yl)ethanone **66** (3.31 g, 14.49 mmol, CF_3_CO_2_H) and the resulting reaction mixture was stirred at 0 °C for 30 min and allowed to warm to room temperature. After 12 h, the reaction mixture was triturated with water (30 mL). The layers were separated, and the aqueous layer was extracted with DCM (30 mL). The combined organic layers were washed with water (25 mL) and brine, dried over Na_2_SO_4_, and concentrated in vacuo to give **67** (2.95 g, crude). ^1^H NMR (500 MHz, DMSO-*d*_*6*_) *δ* 1.75 (s, 3H), 3.85 (s, 3H), 3.93 (m, 2H), 4.17 (m, 1H), 4.56 (m, 2H), 7.07 (s, 1H), 8.97 (bds, 1H). LCMS (ESI): [M-Boc]^+^*m*/*z*: calc'd 284.7; found 286.0; Rt = 0.817 min.

#### 6-((1-acetylazetidin-3-yl)amino)-2-(piperidin-1-yl)pyrimidine-4-carboxylic acid (**68**)

To a solution of **67** (1.5 g, 5.27 mmol) in ACN (20 mL) at room temperature was added TEA (5.80 mmol, 0.808 mL) followed by piperidine (5.80 mmol, 0.572 mL) and the resulting reaction mixture was stirred at 80 °C for 32 h, then it was diluted with water (40 mL). The aqueous layer was extracted with DCM (40 mL × 2). The combined organic layers were washed with water (15 mL) and brine, dried over Na_2_SO_4_, and concentrated in vacuo to give methyl 6-[(1-acetylazetidin-3-yl)amino]-2-(1-piperidyl)pyrimidine-4-carboxylate (1.1 g, crude). ^1^H NMR (500 MHz, DMSO-*d*_*6*_) *δ* 1.49 (m, 6H), 1.75 (s, 3H), 3.58 (m, 3H), 3.68 (m, 5H), 3.95 (m, 1H), 4.12 (m, 1H), 4.39 (m, 1H), 4.53 (m, 1H), 6.53 (s, 1H), 8.31 (bds, 1H). LCMS (ESI): [M-Boc]+ *m*/*z*: calc'd 333.4; found 334.2; Rt = 0.951 min. A mixture of methyl 6-[(1-acetylazetidin-3-yl)amino]-2-(1-piperidyl)pyrimidine-4-carboxylate (1.1 g, 3.30 mmol) and lithium hydroxide, hydrate (304.58 mg, 7.26 mmol) in a mixture of THF (10 mL)–methanol (10 mL)–water (15 mL) was stirred at room temperature for 12 h, at which point volatile organics were removed under reduced pressure. The aqueous phase was washed with DCM (10 mL), then acidified (NaHSO_4_, monohydrate) to pH 5, and the mixture was concentrated in vacuo. The residue was suspended in hot ethanol (100 mL) and filtered. The filter cake was washed with hot ethanol (2 × 50 mL), and the filtrate was discarded. The filtrate was evaporated in vacuo to leave the residue 6-(azetidin-3-ylamino)-2-(piperidin-1-yl)pyrimidine-4-carboxylic acid (1 g, crude, contains 37% of deacylated byproduct). ^1^H NMR (400 MHz, DMSO-*d*_*6*_) *δ* 1.60 (m, 6H), 3.69 (m, 4H), 4.18 (m, 2H), 4.39 (m, 1H), 4.53 (m, 1H), 4.78 (m, 1H), 6.32 (s, 1H), 8.02 (bds, 1H), 8.73 (bds, 1H), 10.45 (bds, 1H). LCMS (ESI): [M-Boc]+ *m*/*z*: calc'd 277.3; found 278.2; Rt = 0.644 min. To a solution of the crude material from previous step which contained 6-(azetidin-3-ylamino)-2-(1-piperidyl)pyrimidine-4-carboxylic acid (1 g, 1.33 mmol) and TEA (54.00 mg, 533.68 μmol, 74 μL) in ACN (25 mL) was added acetyl chloride (533.68 μmol, 32 μL) dropwise at 0 °C. The reaction mixture was then stirred at room temperature, and after 24 h, 70% conversion was observed. TEA (533.68 μmol, 74 μL) and acetyl chloride (533.68 μmol, 32 μL) were added again, and the reaction mixture was then stirred at 35 °C another 24 h. After full consumption of the starting material, the reaction mixture was concentrated in vacuo. Then Na_2_CO_3_ (15 mL, 5% aqueous solution) was added, and the aqueous phase was washed with DCM (2 × 10 mL), then it was acidified (NaHSO_4_, monohydrate) to pH 5 and the mixture was concentrated in vacuo. The residue was suspended in hot ethanol (100 mL) and filtered. The filter cake was washed with hot ethanol (2 × 50 mL), filtered, and discarded. The filtrate was evaporated in vacuo to provide **68** (0.68 g, crude). ^1^H NMR (400 MHz, DMSO-*d*_*6*_) *δ* 1.47 (m, 4H), 1.56 (m, 2H), 1.75 (s, 3H), 3.62 (m, 4H), 3.74 (m, 1H), 3.97 (m, 1H), 4.10 (m, 1H), 4.38 (m, 1H), 4.51 (m, 1H), 6.32 (s, 1H), 7.85 (bds, 1H), 11.45 (bds, 1H). LCMS (ESI): [M-Boc]+ *m*/*z*: calc'd 319.4; found 320.2; Rt = 0.866 min.

#### *tert*-Butyl (*S)*-3-((*R*)-2-(6-((1-acetylazetidin-3-yl)amino)-2-(piperidin-1-yl)pyrimidine-4-carboxamido)-1-hydroxyethyl)-7-(methoxymethoxy)-3,4-dihydroisoquinoline-2(1*H*)-carboxylate (**69**)

**68** (151 mg, 425.62 μmol, HCl) and TEA (4.26 mmol, 593 μL) were dissolved in DMF (3 mL) and cooled to 0 °C, HATU (243 mg, 638 μmol) was added, and the mixture was stirred for 15 min at 0 °C. **64** (0.15 g, 425 μmol) was added, and the mixture was warmed to room temperature and stirred overnight. Ethyl acetate (10 mL) was added, and the organic phase was washed with brine three times. The organic phase was dried over Na_2_SO_4_, filtered, and concentrated in vacuo at 45 °C to give crude product, which was purified by HPLC (45–60% water–acetonitrile, 2–10 min, flow: 30 mL/min (loading pump 4 mL/min acetonitrile) column: SunFire C18 100 mm × 19 mm) to give **69** (48 mg, 73.42 μmol, 17% yield). ^1^H NMR (CDCl_3_, 400 MHz) *δ* 1.49 (m, 11H), 1.62 (m, 3H), 1.69 (s, 3H), 2.83 (m, 1H), 2.96 (m, 1H), 3.11 (m, 1H), 3.43 (s, 3H), 3.69 (m, 2H), 3.79 (m, 4H), 3.99 (m, 3H), 4.21 (m, 1H), 4.31 (m, 4H), 4.65 (m, 2H), 5.10 (s, 2H), 5.84 (m, 1H), 6.46 (s, 1H), 6.76 (s, 1H), 6.85 (d, 1H), 7.06 (d, 1H), 9.12 (m, 1H). LCMS (ESI): [M + H]+ *m*/*z*: calc'd 653.3; found 654.4; Rt = 1.46 min.

#### 6-((1-acetylazetidin-3-yl)amino)-*N*-((*R*)-2-hydroxy-2-((*S*)-7-((4-methyloxazol-5-yl)methoxy)-1,2,3,4-tetrahydroisoquinolin-3-yl)ethyl)-2-(piperidin-1-yl)pyrimidine-4-carboxamide (**53**)

**69** (48 mg, 73.42 μmol) was dissolved in a mixture of Et_2_O (1 mL) and MeOH (2 mL). 4.0 M hydrogen chloride solution in dioxane (5.51 mmol, 250.97 μL) was added. The mixture was stirred for 12 h at 20 °C. Solvent was removed in vacuo at 35 °C to give 6-[(1-acetylazetidin-3-yl)amino]-*N*-[(2*R*)-2-hydroxy-2-[(3*S*)-7-hydroxy-1,2,3,4-tetrahydroisoquinolin-3-yl]ethyl]-2-(1-piperidyl)pyrimidine-4-carboxamide (32 μg, 54.93 μmol, 75% yield, 2HCl) which was used in the next step without further purification. ^1^H NMR (CDCl_3_, 500 MHz) *δ* 1.73 (m, 6H), 1.90 (s, 3H), 3.10 (m, 2H), 3.18 (m, 1H), 3.51 (m, 3H), 3.67 (m, 4H), 3.79 (m, 4H), 4.00 (m, 1H), 4.27 (m, 2H), 4.60 (m, 1H), 4.78 (m, 1H), 6.63 (s, 1H), 6.68 (s, 1H), 6.75 (d, 1H), 7.12 (d, 1H), OH and NH is not observed. LCMS (ESI): [M + H]+ *m*/*z*: calcd 509.2; found 508.2; Rt = 2.47 min. The material was dissolved in a mixture of water (1 mL) and THF (1 mL) and then sodium hydrogen carbonate; 99% (14 mg, 164.80 μmol) was added in one portion, followed by a solution of di-*tert*-butyl dicarbonate (12 mg, 54.93 μmol) in THF (0.2 mL). The reaction mixture was stirred overnight at room temperature. Ethyl acetate (15 mL) was added to the reaction mixture, the organic phase was separated, and water was extracted with ethyl acetate (2 × 15 mL). The organic phase was washed with brine, dried over Na_2_SO_4_, filtered and evaporated in vacuo at 40 °C to give *tert*-butyl (*S*)-3-[(*R*)-2-[[6-[(1-acetylazetidin-3-yl)amino]-2-(1-piperidyl)pyrimidine-4-carbonyl]amino]-1-hydroxy-ethyl]-7-hydroxy-3,4-dihydro-1H-isoquinoline-2-carboxylate (30 mg, 49.20 μmol, 90% yield) which was used in the next step without purification. ^1^H NMR (CDCl_3_, 500 MHz) *δ* 1.45 (m, 10H), 1.67 (m, 6H), 1.88 (s, 3H), 2.82 (m, 3H), 3.76 (m, 6H), 3.83 (m, 2H), 4.13 (m, 2H), 4.27 (m, 2H), 4.52 (m, 1H), 4.65 (m, 1H), 6.41 (s, 1H), 6.56 (s, 1H), 6.64 (d, 1H), 6.98 (d, 1H), OH and NH is not observed. LCMS (ESI): [M + H]^+^*m*/*z*: calc'd 609.3; found 610.4; Rt = 1.38 min. The material, 5-(chloromethyl)-4-methyl-oxazole (10 mg, 59.04 μmol, HCl) and cesium carbonate (48 mg, 147.61 μmol) was dissolved in DMF (2 mL) and heated to 50 °C overnight. The reaction mixture was filtered, the solid was washed with DMF (2 mL), and the filtrate was concentrated in vacuo at 60 °C to give *tert*-butyl (*S*)-3-[(*R*)-2-[[6-[(1-acetylazetidin-3-yl)amino]-2-(1-piperidyl)pyrimidine-4-carbonyl]amino]-1-hydroxy-ethyl]-7-[(4-methyloxazol-5-yl)methoxy]-3,4-dihydro-1H-isoquinoline-2-carboxylate (34 mg, 48.24 μmol, 98% yield), which was used in the next step without further purification. ^1^H NMR (CDCl_3_, 400 MHz) *δ* 1.50 (m, 19H), 1.86 (m, 2H), 2.21 (s, 3H), 3.12 (m, 1H), 3.69 (m, 6H), 4.00 (m, 2H), 4.21 (m, 4H), 4.62 (m, 2H), 4.96 (m, 2H), 6.41 (s, 1H), 6.68 (s, 1H), 6.79 (d, 1H), 7.12 (d, 1H), 7.80 (s, 1H), 8.00 (s, 1H), 9.07 (m, 1H). LCMS (ESI): [M + H]^+^*m*/*z*: calc'd 704.3; found 705.2; Rt = 4.29 min. The material was dissolved in a mixture of Et_2_O (1 mL) and MeOH (0.2 mL). 4.0 M hydrogen chloride solution in dioxane (3.62 mmol, 165 μL) was added. The solvent was removed in vacuo at 45 °C. The residue was dissolved in 5 mL of methanol and 10 mg of scavenger (SiliaMetS© Dimercaptotriazine(DMT)) was added and the resulting suspension was stirred for 12 h. The suspension was filtered, the filtrate was evaporated under reduced pressure, and the residue was purified by HPLC (25–60% water–methanol, 10 min, flow 30 mL/min (loading pump 4 mL/min methanol), column: SUNFIRE C18 100 mm × 29 mm) to give **53** (10.5 mg, 14.70 μmol, 30% yield, 3HCl). ^1^H NMR (MeOH-*d*_*4*_, 400 MHz) *δ* 1.73 (m, 6H), 1.91 (s, 3H), 2.23 (s, 3H), 2.66 (s, 2H), 3.16 (m, 2H), 3.59 (m, 3H), 3.79 (m, 3H), 4.24 (m, 5H), 4.57 (m, 2H), 4.80 (m, 1H), 5.12 (s, 2H), 6.74 (s, 1H), 6.89 (s, 1H), 6.96 (d, 1H), 7.23 (d, 1H), 8.44 (s, 1H). ^1^H NMR (DMSO-*d*_*6*_, 500 MHz) *δ* 1.48 (m, 4H), 1.59 (m, 2H), 1.74 (s, 3H), 2.13 (s, 3H), 2.23 (m, 1H), 2.50 (m, 1H), 2.71 (m, 2H), 3.35 (m, 1H), 3.51 (m, 2H), 3.71 (m, 5H), 3.84 (m, 2H), 3.94 (dd, 1H), 4.10 (t, 1H), 4.37 (t, 1H), 4.52 (m, 1H), 5.05 (s, 2H), 5.06 (m, 1H), 6.35 (s, 1H), 6.67 (d, 1H), 6.74 (dd, 1H), 6.98 (d, 1H), 7.88 (s, 1H), 8.26 (s, 1H), 8.77 (m, 1H). ^13^C NMR (DMSO-*d*_*6*_, 126 MHz) *δ* 10.92, 18.74, 24.40, 25.32, 29.50, 43.31, 44.13, 47.79, 53.97, 57.10, 57.28, 58.63, 71.73, 111.46, 113.12, 127.58, 130.01, 134.25, 137.38, 142.24, 151.46, 155.59, 160.51, 163.66, 169.62. LCMS (ESI): [M + H]^+^*m*/*z*: calc'd 604.3; found 605.3; Rt = 1.01 min. [α]^21^ D = −44.69° (*c* = 0.25 g/100 mL, MeOH). Elemental analysis [calc'd/found] C[61.57/60.85] H[6.67/6.82] N[18.53/18.38] O[13.23]. HRMS (ESI, + vw ion) *m*/*z* calcd for C_31_H_40_N_8_O_5_ [M+H^+^] 604.31217, found 604.31084

#### 2-(cyclobutylamino)isonicotinic Acid (**70**)

A solution of *tert*-butyl 2-(cyclobutylamino) pyridine-4-carboxylate, **56** (12 g, 43.49 mmol, 1 equiv) in HCl/EtOAc (250 mL, 4M) was stirred at 50 °C for 12 h. The precipitate was collected by filtration, washed with EtOAc (30 mL × 3), and dried under high vacuum to afford 2-(cyclobutylamino)pyridine-4-carboxylic acid, **70** (9.9 g, HCl salt, 99% yield) as a yellow solid. ^1^H NMR (400 MHz, DMSO-*d*_*6*_) *δ* 9.27 (br s, 1 H), 7.99 (d, J = 6.3 Hz, 1 H), 7.41 (s, 1 H), 7.06 (dd, J = 6.5, 1.3 Hz, 1 H), 4.29 (t, J = 7.5 Hz, 1 H), 2.37–2.46 (m, 2 H), 1.94–2.07 (m, 2 H), 1.65–1.85 (m, 2 H); LCMS (M+H+) *m*/*z*: calc'd 193.1; found 193.4.

#### *tert*-butyl 3-Hydroxy-4,4-dimethoxypiperidine-1-carboxylate (**71**)

A solution of KOH (3.52 g, 62.74 mmol) in MeOH (75 mL) was cooled to 0 °C then *tert*-butyl 4-oxopiperidine-1-carboxylate (5 g, 25.09 mmol) was added portion wise. After stirring for 20 min, a solution of iodine (7.64 g, 30.10 mmol) in MeOH (100 mL) was added dropwise at 0 °C. After the addition was complete, the mixture was stirred for 1 h at 0 °C, then warmed to 20 °C and stirred for another 12 h under N_2_. The resulting mixture was concentrated under reduced pressure and the residue was diluted with toluene (200 mL) and stirred for 1 h at room temperature. The solid was removed by filtration and washed with toluene (50 mL), and the filtrate was concentrated in a vacuum to afford *tert*-butyl 3-hydroxy-4,4-dimethoxy-piperidine-1-carboxylate, **71** (6.56 g, crude) as a yellow oil which was used in next step directly. ^1^H NMR (400 MHz, DMSO-*d_6_*) *δ* 4.76 (d, J = 3.9 Hz, 1 H), 3.67–3.91 (m, 2 H), 3.56 (s, 1 H), 3.32 (s, 1 H), 3.11 (s, 6 H), 2.83–3.05 (m, 1 H), 2.57–2.80 (m, 1 H), 1.56–1.73 (m, 2 H), 1.33–1.47 (s, 9H).

#### *tert*-butyl 3-hydroxy-4-oxopiperidine-1-carboxylate (**72**)

To a solution of **71** (6.56 g, 25.10 mmol) in acetone (100 mL) was added p-toluenesulfonic acid monohydrate (250 mg, 1.31 mmol) and the mixture was stirred for 72 h at 20 °C under N_2_. The resulting mixture was concentrated under reduced pressure, and the residue was dissolved in MTBE (150 mL). The organic layer was washed with saturated NaHCO_3_ aqueous solution (100 mL x 2), brine (100 mL), dried over anhydrous Na_2_SO_4_ and filtered. The solvent was removed in vacuum to afford *tert*-butyl 3-hydroxy-4-oxo-piperidine-1-carboxylate, **72** (5 g, crude) as a yellow oil. LCMS (ESI) [M+H-56]+ *m*/*z* calc'd 160.1, found 160.1.

#### *tert*-butyl 3-hydroxy-4-(6-((1-methyl-1*H*-pyrazol-5-yl)methoxy)-3,4-dihydroisoquinolin-2(1*H*)-yl)piperidine-1-carboxylate (**73**)

To a solution of **72** (1.46 g, crude) and **61** (300 mg, 1.23 mmol) in THF (15 mL) was added NaBH(OAc)_3_ (780 mg, 3.7 mmol). The mixture was stirred at 20 °C for 12 h. The resulting mixture was concentrated under reduced pressure, diluted with H_2_O (20 mL) and extracted with EtOAc (25 mL × 3). The combined organic layers were dried over anhydrous Na_2_SO_4_, filtered, and concentrated under reduced pressure. The residue was purified by flash chromatography (ISCO; 20 g of AgelaFlash Silica Flash Column, DCM/MeOH with MeOH from 0–10%, flow rate = 30 mL/min) to afford a crude product (mixture of two isomers), which was purified by preparative HPLC purification (Instrument: Gilson GX-281 Liquid Handler, Gilson 322 Pump, Gilson 156 UV Detector; Column: Waters Xbridge 150 × 25 mm × 5 μm; Mobile phase A: H_2_O with 0.05% NH_3_–H_2_O (v%); Mobile phase B: MeCN; Gradient: B from 40% to 70% in 7.8 min, hold 100% B for 1 min; Flow Rate: 25 mL/min; Column Temperature: 30 °C; Wavelength: 220 nm, 254 nm) to afford *tert*-butyl 3-hydroxy-4-[6-[(2-methylpyrazol-3-yl)methoxy]-3,4-dihydro-1*H*-isoquinolin-2-yl]piperidine-1-carboxylate (58 mg, 11%, cis) as light-yellow solid and *tert*-butyl 3-hydroxy-4-[6-[(2-methylpyrazol-3-yl)methoxy]-3,4-dihydro-1*H*-isoquinolin-2-yl]piperidine-1-carboxylate (33 mg, 6%, trans) as light-yellow solid. *tert*-butyl 3-hydroxy-4-[6-[(2-methylpyrazol-3-yl)methoxy]-3,4-dihydro-1*H*-isoquinolin-2-yl]piperidine-1-carboxylate (58 mg, 11%, cis): ^1^H NMR (400 MHz, MeOH-*d*_*4*_) *δ* 7.42 (d, J = 1.8 Hz, 1H), 7.01 (d, J = 8.3 Hz, 1H), 6.79–6.84 (m, 2H), 6.38 (d, J = 1.8 Hz, 1H), 5.12 (s, 2H), 4.18 (br s, 3H), 3.90 (s, 3H), 3.84 (s, 2H), 2.20–3.03 (m, 6H), 2.50 (d, J = 11.3 Hz, 1H), 1.87–1.98 (m, 1H), 1.77–1.84 (m, 1H), 1.48 (s, 9H); LCMS (ESI) [M + H]+ *m*/*z*: calc'd 443.3, found 443.2. *tert*-butyl 3-hydroxy-4-[6-[(2-methylpyrazol-3-yl)methoxy]-3,4-dihydro-1*H*-isoquinolin-2-yl]piperidine-1-carboxylate, **73** (33 mg, 6%, trans): ^1^H NMR (400 MHz, MeOH-*d*_*4*_) *δ* 7.42 (d, J = 1.8 Hz, 1H), 7.00 (d, J = 8.3 Hz, 1H), 6.78–6.82 (m, 2H), 6.37 (d, J = 2.0 Hz, 1H), 5.12 (s, 2H), 4.24 (d, J = 10.5 Hz, 1H), 4.12 (d, J = 13.6 Hz, 1H), 3.90 (s, 3H), 3.76–3.88 (m, 2H), 3.65 (dd, J = 9.8, 5.0 Hz, 1H), 2.98–3.02 (m, 1H), 2.76–2.92 (m, 4H), 2.58–2.69 (m, 2H), 1.89 (d, J = 13.3 Hz, 1H), 1.51–1.58 (m, 1H), 1.49 (s, 9H); LCMS (ESI) [M + H]+ *m*/*z*: calc'd 443.3, found 443.2.

#### 4-[6-[(2-methylpyrazol-3-yl)methoxy]-3,4-dihydro-1*H*-isoquinolin-2-yl]piperidin-3-ol (**74**)

To a solution of **73** (30.0 mg, 0.07 mmol, *trans*) in DCM (3 mL) was added 4 M HCl/EtOAc (1 mL, 4 mmol). The mixture was stirred at 20 °C for 2 h. The resulting mixture was concentrated under reduced pressure to afford 4-[6-[(2-methylpyrazol-3-yl)methoxy]-3,4-dihydro-1*H*-isoquinolin-2-yl]piperidin-3-ol, **74** (45 mg, crude, HCl salt, trans) as white solid which was used in the next step reaction without purification.

(2-(Cyclobutylamino)pyridin-4-yl)((3*R*,4*R*)-3-hydroxy-4-(6-((1-methyl-1*H*-pyrazol-5-yl)methoxy)-3,4-dihydroisoquinolin-2(1*H*)-yl)piperidin-1-yl)methanone (**45a**) and (2-(cyclobutylamino)pyridin-4-yl)((3*S*,4*S*)-3-hydroxy-4-(6-((1-methyl-1*H*-pyrazol-5-yl)methoxy)-3,4-dihydroisoquinolin-2(1*H*)-yl)piperidin-1-yl)methanone (**45b**)

A mixture of **70** (14.5 mg, 0.06 mmol, HCl salt, *trans*), HATU (31.6 mg, 0.08 mmol), and DIPEA (0.3 mmol, 50 uL) in DCM (4 mL) was stirred at 20 °C for 30 min. Then a solution of **74** (20.0 mg, 0.05 mmol, HCl salt) in DMF (1 mL) was added, and the mixture was stirred for 12 h at 20 °C. The mixture was concentrated under reduced pressure to remove DCM, and the residue was preparative HPLC (Instrument: Gilson GX-281 Liquid Handler, Gilson 322 Pump, Gilson 156 UV Detector; Column: Waters Xbridge 150 × 25 mm × 5 μm; Mobile phase A: H_2_O with 0.05% NH_3_–H_2_O (v%); Mobile phase B: MeCN; Gradient: B from 23% to 53% in 9.5 min, hold 100% B for 2 min; Flow Rate: 25 mL/min; Column Temperature: 30 °C; Wavelength: 220 nm, 254 nm) to afford [2-(cyclobutylamino)-4-pyridyl]-[3-hydroxy-4-[6-[(2-methylpyrazol-3-yl)methoxy]-3,4-dihydro-1*H*-isoquinolin-2-yl]-1-piperidyl]methanone (15 mg, 55%, *trans*, 2.1 mg was delivered to Evotec) as a light-yellow solid. ^1^H NMR (400 MHz, methanol-*d_4_*) *δ* 8.00 (t, *J* = 5.7 Hz, 1H), 7.40 (d, *J* = 1.8 Hz, 1H), 6.99 (d, *J* = 8.3 Hz, 1H), 6.75–6.82 (m, 2H), 6.49–6.56 (m, 1H), 6.44 (d, *J* = 8.8 Hz, 1H), 6.36 (d, *J* = 2.2 Hz, 1H), 5.10 (s, 2H), 4.50–4.73 (m, 2H), 4.27 (br s, 1H), 3.85–3.92 (m, 3H), 3.67–3.85 (m, 3H), 3.07–3.16 (m, 1H), 2.93–3.03 (m, 2H), 2.83–2.93 (m, 3H), 2.68–2.80 (m, 1H), 2.39 (br s, 2H), 1.86–2.05 (m, 3H), 1.79 (d, *J* = 7.9 Hz, 2H), 1.51–1.68 (m, 1H); LCMS (ESI) [M + H]^+^*m*/*z*: calc'd 517.3, found 517.0; HPLC: 94.54% at 254 nm; racemic. The material (10.0 mg, trans) was separated by chiral SFC (Instrument: Berger, MULTIGR AM-II; Column: DAICEL CHIRALPAK AS-H 250 × 30 mm I.D. Five μm; Mobile phase: supercritical CO_2_/EtOH (0.1% NH_3_•H_2_O, v%) = 60/40; Flow Rate: 50 mL/min; Column Temperature: 38 °C; Nozzle Pressure: 100 bar; Nozzle Temperature: 60 °C; Evaporator Temperature: 20 °C; Trimmer Temperature: 25 °C; Wavelength: 220 nm) to afford **45a** and **45b**. **45a** (peak 2, retention time: 4.516 min): (4.4 mg, light-yellow solid). ^1^H NMR (400 MHz, methanol-*d*_*4*_) *δ* 7.97–8.03 (m, 1H), 7.42 (d, J = 1.9 Hz, 1H), 7.02 (d, J = 8.1 Hz, 1H), 6.79–6.86 (m, 2H), 6.47–6.60 (m, 2H), 6.38 (d, J = 1.9 Hz, 1H), 5.13 (s, 2H), 4.74 (br d, J = 12.9 Hz, 1H), 4.62 (s, 1H), 4.36 (br s, 1H), 4.21–4.30 (m, 1H), 4.14 (br s, 1H), 3.85–3.93 (m, 5H), 3.79 (br d, J = 13.9 Hz, 1H), 3.25 (br d, J = 13.4 Hz, 1H), 3.14 (br t, J = 11.9 Hz, 1H), 3.03 (br d, J = 5.1 Hz, 2H), 2.89–2.96 (m, 2H), 2.79–2.88 (m, 1H), 2.68 (br s, 1H), 2.37–2.47 (m, 2H), 1.87–2.04 (m, 4H), 1.74–1.85 (m, 2H); LCMS (ESI) [M + H]+ *m*/*z*: calc'd 517.3, found 517.2; HPLC: 100% at 254 nm; 99.9% ee. **45b** (peak 1, retention time: 4.280 min): (2.9 mg, light-yellow solid). ^1^H NMR (400 MHz, methanol-*d*_*4*_) *δ* 8.02 (t, J = 5.6 Hz, 1H), 7.42 (d, J = 1.9 Hz, 1H), 7.02 (d, J = 8.4 Hz, 1H), 6.79–6.86 (m, 2H), 6.54 (dd, J = 8.2, 5.7 Hz, 1H), 6.46 (d, J = 8.5 Hz, 1H), 6.38 (d, J = 1.9 Hz, 1H), 5.13 (s, 2H), 4.72 (br d, J = 9.3 Hz, 1H), 4.62 (s, 1H), 4.58 (br d, J = 14.0 Hz, 1H), 4.28 (br t, J = 6.6 Hz, 1H), 3.87–3.99 (m, 5H), 3.70–3.86 (m, 2H), 2.98–3.19 (m, 2H), 2.94 (br s, 3H), 2.83 (br s, 1H), 2.70–2.79 (m, 1H), 2.43 (br d, J = 4.5 Hz, 2H), 1.89–2.09 (m, 3H), 1.76–1.86 (m, 2H), 1.57–1.74 (m, 1H); LCMS (ESI) [M + H]+ *m*/*z*: calc'd 517.3, found 517.2; HPLC: 100% at 254 nm; 99.3% ee.

#### *tert*-butyl 7-oxa-3-azabicyclo[4.1.0]heptane-3-carboxylate (**75**)

To a solution of *tert*-butyl 3,6-dihydro-2*H*-pyridine-1-carboxylate (2 g, 10.9 mmol) in DCM (10 mL) was added 3-chlorobenzenecarboperoxoic acid (2.60 g, 12.1 mmol, 80 wt %) at 0 °C. The mixture was stirred for 16 h at 20 °C. The reaction mixture was filtered, and the filtrate was concentrated under reduced pressure. The residue was purified with flash chromatography (ISCO; 20 g AgelaFlash Silica Flash Column, petroleum ether/EtOAc with EtOAc from 0–30%, flow rate = 35 mL/min) to afford *tert*-butyl 7-oxa-4-azabicyclo[4.1.0]heptane-4-carboxylate, **75** (1.68 g, 77% yield) as colorless oil. ^1^H NMR (400 MHz, MeOH-*d*_*4*_) *δ* 3.76 (br d, J = 17.1 Hz, 2H), 3.15–3.30 (m, 4H), 1.98 (br s, 1H), 1.84–1.93 (m, 1H), 1.45 (s, 9H).

#### *tert*-butyl 6-formyl-3,4-dihydroisoquinoline-2(1*H*)-carboxylate (**76**)

To a mixture of *tert*-butyl 6-bromo-3,4-dihydro-1*H*-isoquinoline-2-carboxylate (2.8 g, 8.97 mmol) in DMF (15 mL) were added Pd(dppf)Cl_2_ (1.2 g, 1.64 mmol), TES (5.2 mL, 32.6 mmol), and TEA (5.5 mL, 39.5 mmol). The mixture was degassed and backfilled with Ar 3 times, then repeated with CO 3 times, and stirred for 24 h at 80 °C under CO (50 psi). The mixture was diluted with EtOAc (100 mL) and filtered, and the filtrate was washed with brine (50 mL x 5), dried over anhydrous Na_2_SO_4_, filtered, and concentrated under reduced pressure. The residue was purified by flash chromatography (ISCO; 40 g AgelaFlash Silica Flash Column, petroleum ether/EtOAc with EtOAc from 0–20%, flow rate = 40 mL/min) to afford *tert*-butyl 6-formyl-3,4-dihydro-1*H*-isoquinoline-2-carboxylate, **76** (1.84 g, 79% yield) as light-yellow solid. ^1^H NMR (400 MHz, MeOH-*d*_*4*_) *δ* 9.93 (s, 1H), 7.71–7.75 (m, 2H), 7.35 (d, J = 7.8 Hz, 1H), 4.65 (br s, 2H), 3.68 (t, J = 5.7 Hz, 2H), 2.93 (t, J = 5.9 Hz, 2H), 1.50 (s, 9H); LCMS (ESI) [M+H-56]+*m*/*z*: calc'd 206.1; found 206.0.

#### *tert*-butyl 6-(Hydroxy(1-methyl-1*H*-pyrazol-5-yl)methyl)-3,4-dihydroisoquinoline-2(1*H*)-carboxylate (**77**)

A mixture of 5-iodo-1-methyl-pyrazole (2.20 g, 10.6 mmol) in THF (6 mL) was degassed under vacuum and purged with N_2_ several times. *n*-BuLi (9 mL, 22.5 mmol, 2.5 M in hexane) was added dropwise into the mixture under −78 °C. When the addition was complete, the mixture was stirred at −78 °C for 30 min. A solution of **76** (2.3 g, 8.80 mmol) in THF (6 mL) was added into the mixture under −78 °C and the mixture was allowed to warm to 20 °C and stirred for 12 h. The reaction mixture was quenched by addition of a saturated aqueous solution of NH_4_Cl (10 mL), and then extracted with EtOAc (15 mL x 3). The combined organic layers were washed dried over anhydrous Na_2_SO_4_, filtered, and concentrated under reduced pressure to give a residue which was purified with flash chromatography (ISCO; 20 g AgelaFlash Silica Flash Column, petroleum ether/EtOAc with EtOAc from 0–50%, flow rate = 35 mL/min) to afford *tert*-butyl 6-[hydroxy-(2-methylpyrazol-3-yl)methyl]-3,4-dihydro-1*H*-isoquinoline-2-carboxylate, **77** (2.8 g, 93% yield) as yellow oil. ^1^H NMR (400 MHz, MeOH-*d*_*4*_) *δ* 7.34 (d, J = 1.9 Hz, 1H), 7.18–7.22 (m, 2H), 7.12–7.16 (m, 1H), 6.03 (d, J = 1.8 Hz, 1H), 5.88 (s, 1H), 4.56 (br s, 2H), 3.77 (s, 3H), 3.60–3.68 (m, 2H), 2.83 (t, J = 5.9 Hz, 2H), 1.49 (s, 9H); LCMS (ESI) [M + H]+*m*/*z*: calc'd 344.2, found 344.2.

#### 6-((1-methyl-1*H*-pyrazol-5-yl)methyl)-1,2,3,4-tetrahydroisoquinoline (**78**)

To a mixture of **77** (2.8 g, 8.15 mmol) in *t*-BuOH (8 mL) were added Pd/C (200 mg, 10 wt % of Pd with 50 wt % of H_2_O) and HCl/H_2_O (1 mL, 38 wt %). The suspension was degassed under a vacuum and purged with H_2_ several times. The mixture was stirred under H_2_ (∼15 psi) at 70 °C for 48 h. The reaction mixture was filtered, and the filtrate was concentrated under reduced pressure to afford a crude product which was diluted with H_2_O (10 mL), adjusted to pH = 8 with a saturated aqueous solution of NaHCO_3_. The aqueous solution was concentrated under reduced pressure and the residue was triturated with DCM/MeOH (20 mL, v/v = 10:1). After stirring at 20 °C for 30 min, the solid was removed by filtration and the filtrate was concentrated under reduced pressure to afford 6-[(2-methylpyrazol-3-yl)methyl]-1,2,3,4-tetrahydroisoquinoline, **78** (700 mg, crude) as light-yellow oil. ^1^H NMR (400 MHz, MeOH-*d*_*4*_) *δ* 7.35 (d, J = 1.9 Hz, 1H), 6.97–7.01 (m, 1H), 6.91–6.96 (m, 2H), 6.04 (d, J = 1.9 Hz, 1H), 3.98 (s, 2H), 3.93 (s, 2H), 3.68 (s, 3H), 3.07 (t, J = 6.0 Hz, 2H), 2.79 (t, J = 5.9 Hz, 2H); LCMS (ESI) [M + H]+ *m*/*z*: calc'd 228.1, found 228.1.

#### *Rac*-*tert*-butyl (3*R*,4*R*)-3-hydroxy-4-(6-((1-methyl-1*H*-pyrazol-5-yl)methyl)-3,4-dihydroisoquinolin-2(1*H*)-yl)piperidine-1-carboxylate (**79**)

To a mixture of **75** (1.1 g, 5.52 mmol) and **78** (700 mg, 3.08 mmol) in EtOH (10 mL) was added TEA (1.4 mL, 10.0 mmol). The mixture was stirred at 100 °C for 12 h. The reaction mixture was concentrated under reduced pressure, and the residue was purified with preparative HPLC (Instrument: Gilson GX-281 Liquid Handler, Gilson 322 Pump, Gilson 156 UV Detector; YMC-Triart Prep C18 150 × 40 mm × 7 μm; Mobile phase A: H_2_O with 0.04% NH_3_–H_2_O (v %)+10 mM NH_4_HCO_4_; Mobile phase B: MeCN; Gradient: B from 50% to 50% in 10 min, hold 100% B for 4 min; Flow Rate: 60 mL/min; Column Temperature: 30 °C; Wavelength: 220 nm, 254 nm) to afford *rac*-*tert*-butyl (3*R*,4*R*)-4-hydroxy-3-(6-((1-methyl-1*H*-pyrazol-5-yl)methyl)-3,4-dihydroisoquinolin-2(1*H*)-yl)piperidine-1-carboxylate (540 mg, 43%, racemic, trans, light-yellow oil). ^1^H NMR (400 MHz, MeOH-*d*_*4*_) *δ* 7.35 (d, J = 2.0 Hz, 1H), 6.98–7.02 (m, 1H), 6.90–6.95 (m, 2H), 6.04 (d, J = 1.8 Hz, 1H), 4.12 (br s, 1H), 3.84–4.02 (m, 7H), 3.68 (s, 3H), 3.08 (br s, 1H), 2.81–2.93 (m, 5H), 2.46 (td, J = 9.9, 3.9 Hz, 1H), 1.95–2.03 (m, 1H), 1.46 (s, 9H); LCMS (ESI) [M + H]+*m*/*z*: calc'd 427.3, found 427.2 and *rac-tert-*butyl (3*R*,4*R*)-3-hydroxy-4-(6-((1-methyl-1*H*-pyrazol-5-yl)methyl)-3,4-dihydroisoquinolin-2(1*H*)-yl)piperidine-1-carboxylate, **79** (210 mg, 16% yield, racemic, trans, light-yellow oil). ^1^H NMR (400 MHz, MeOH-*d*_*4*_) *δ* 7.35 (d, J = 2.0 Hz, 1H), 7.00 (d, J = 8.3 Hz, 1H), 6.90–6.94 (m, 2H), 6.03 (d, J = 2.0 Hz, 1H), 4.22 (br d, J = 10.0 Hz, 1H), 4.10 (br d, J = 12.8 Hz, 1H), 3.97 (s, 2H), 3.87–3.93 (m, 1H), 3.78–3.84 (m, 1H), 3.67 (s, 3H), 3.62 (dt, J = 9.7, 5.0 Hz, 1H), 2.95–3.01 (m, 1H), 2.77–2.87 (m, 4H), 2.55–2.66 (m, 2H), 1.87 (br dd, J = 13.3, 3.3 Hz, 1H), 1.48–1.56 (m, 2H), 1.47 (s, 9H);LCMS (ESI) [M + H]+*m*/*z*: calc'd 427.3; found 427.2.

#### (2-(cyclobutylamino)pyridin-4-yl)((3*R*,4*R*)-3-hydroxy-4-(6-((1-methyl-1*H*-pyrazol-5-yl)methyl)-3,4-dihydroisoquinolin-2(1*H*)-yl)piperidin-1-yl)methanone (**46a**) and [2-(cyclobutylamino)-4-pyridyl]-[(3*S*,4*S*)-3-hydroxy-4-[6-[(2-methylpyrazol-3-yl)methyl]-3,4-dihydro-1*H*-isoquinolin-2-yl]-1-piperidyl]methanone (**46b**)

To a mixture of *tert*-butyl 3-hydroxy-4-[6-[(2-methylpyrazol-3-yl)methyl]-3,4-dihydro-1*H*-isoquinolin-2-yl]piperidine-1-carboxylate (60 mg, 0.141 mmol) in DCM (3 mL) was added TFA (160 μL, 2.08 mmol). The mixture was stirred at 20 °C for 12 h. The mixture was diluted with H_2_O (8 mL), adjusted to pH = 8 with a saturated aqueous solution of NaHCO_3_, and then extracted with DCM (10 mL × 2). The combined organic layers were dried over anhydrous Na_2_SO_4_, filtered, and concentrated under reduced pressure to afford 4-[6-[(2-methylpyrazol-3-yl)methyl]-3,4-dihydro-1*H*-isoquinolin-2-yl]piperidin-3-ol (40 mg, crude) as light-yellow oil, which was used into the next step directly. A mixture of 2-(cyclobutylamino)pyridine-4-carboxylic acid (40 mg, 0.175 mmol, HCl salt), HATU (70 mg, 0.184 mmol), DIPEA (100 μL, 0.574 mmol) in DCM (3 mL) and DMF (1 mL) was stirred at 20 °C for 30 min. Then 4-[6-[(2-methylpyrazol-3-yl)methyl]-3,4-dihydro-1*H*-isoquinolin-2-yl]piperidin-3-ol (40 mg, 0.123 mmol) was added, and the mixture was stirred for 12 h at 20 °C. The reaction mixture was concentrated under reduced pressure, and the residue was purified with preparative HPLC (Instrument: Gilson GX-281 Liquid Handler, Gilson 322 Pump, Gilson 156 UV Detector; Column: Waters Xbridge BEH C18 100 × 25 mm × 5 μm; Mobile phase A: H_2_O with 0.05% NH_3_–H_2_O (v%); Mobile phase B: MeCN; Gradient: B from 40% to 80% in 7.8 min, hold 100% B for 2 min; Flow Rate: 25 mL/min; Column Temperature: 30 °C; Wavelength: 220 nm, 254 nm) to afford [2-(cyclobutylamino)-4-pyridyl]-[3-hydroxy-4-[6-[(2-methylpyrazol-3-yl)methyl]-3,4-dihydro-1*H*-isoquinolin-2-yl]-1-piperidyl]methanone (40 mg, 65% yield) as white solid which was separated by chiral SFC (Instrument: Thar 80; Column: Daicel Chiralpak AD 250 × 30 mm I.D. Ten μm; Mobile phase: supercritical CO2/EtOH (0.1% NH_3_–H_2_O, v%) = 60/40; Flow Rate: 80 mL/min; Column Temperature: 38 °C; Nozzle Pressure: 100 bar; Nozzle Temperature: 60 °C; Evaporator Temperature: 20 °C; Trimmer Temperature: 25 °C; Wavelength: 220 nm) to afford **46a**, [2-(cyclobutylamino)-4-pyridyl]-[(3*R*,4*R*)-3-hydroxy-4-[6-[(2-methylpyrazol-3-yl)methyl]-3,4-dihydro-1*H*-isoquinolin-2-yl]-1-piperidyl]methanone (15 mg, 38% yield, peak 1, retention time = 3.753 min, white solid). ^1^H NMR (400 MHz, MeOH-*d*_*4*_) *δ* 8.00 (t, J = 5.6 Hz, 1H), 7.35 (d, J = 1.9 Hz, 1H), 6.98–7.03 (m, 1H), 6.89–6.95 (m, 2H), 6.52 (dd, J = 8.0, 5.6 Hz, 1H), 6.44 (d, J = 8.5 Hz, 1H), 6.03 (d, J = 1.9 Hz, 1H), 4.69 (br dd, J = 12.4, 3.7 Hz, 1H), 4.52–4.71 (m, 1H), 3.98 (s, 2H), 3.88 (q, J = 14.8 Hz, 2H), 3.68–3.81 (m, 2H), 3.67 (s, 3H), 2.68–3.16 (m, 2H), 2.91–3.04 (m, 2H), 2.81–2.90 (m, 3H), 2.35–2.45 (m, 2H), 1.84–2.05 (m, 3H), 1.74–1.82 (m, 2H), 1.51–1.73 (m, 1H); LCMS (ESI) [M + H]+ *m*/*z*: calc'd 501.3, found 501.2; HPLC: 100% at 254 nm; 99.7% ee and **46b**, [2-(cyclobutylamino)-4-pyridyl]-[(3*S*,4*S*)-3-hydroxy-4-[6-[(2-methylpyrazol-3-yl)methyl]-3,4-dihydro-1*H*-isoquinolin-2-yl]-1-piperidyl]methanone (15 mg, 38% yield, retention time = 4.259 min, white solid). ^1^H NMR (400 MHz, MeOH-*d*_*4*_) *δ* 8.00 (t, J = 5.6 Hz, 1H), 7.35 (d, J = 1.9 Hz, 1H), 6.99–7.03 (m, 1H), 6.90–6.95 (m, 2H), 6.52 (dd, J = 7.9, 5.4 Hz, 1H), 6.44 (d, J = 8.6 Hz, 1H), 6.03 (d, J = 1.9 Hz, 1H), 4.50–4.73 (m, 1H), 4.21–4.30 (m, 1H), 3.98 (s, 2H), 3.82–3.95 (m, 2H), 3.69–3.81 (m, 2H), 3.67 (s, 3H), 2.68–3.16 (m, 2H), 2.90–3.05 (m, 2H), 2.83–2.90 (m, 3H), 2.36–2.45 (m, 2H), 1.84–2.04 (m, 3H), 1.72–1.83 (m, 2H), 1.53–1.71 (m, 1H); LCMS (ESI) [M + H]+ *m*/*z*: calc'd 501.3, found 501.2; HPLC: 99.32% at 254 nm; 99.9% ee.

## Supporting Information Available

General experimental and chemical procedures, NMR spectra, HPLC/LCMS traces, and other analytical data of final compounds, biochemical fluorescence anisotropy peptide displacement assay, HAP1 MTAP WT and MTAP-null in-cell western assay, HAP1 MTAP WT and MTAP-null viability assay, nanoluciferase thermal shift in cell lysates, MDR1-MDCKII assay, human liver microsomes assay, kinetic solubility assay, PRMT5:MEP50 expression and purification, and PRMT5:MEP50 crystallography (PDF)
Molecular formula strings (CSV)

## Abbreviations

MDCK: Madin-Darby Canine Kidney cell
Mdr1: multidrug resistance 1
MTA: methylthioadenosine
MTAP: methylthioadenosine phosphorylase
MW: molecular weight
PRMT5: protein arginine methyltransferase
PSA: polar surface area
TAMRA: tetramethylrhodamine
