# Supplementary material for: MTA-Cooperative PRMT5 Inhibitors: Mechanism Switching Through Structure-Based Design
Source: J Med Chem. 2025 Feb 7;68(4):4217–36. doi: 10.1021/acs.jmedchem.4c01998 (PMC11874000; doi:10.1021/acs.jmedchem.4c01998)
Supplement: Supplementary file 1 — jm4c01998_si_001.pdf [file jm4c01998_si_001.pdf]

SUPPORTING INFORMATION

MTA COOPERATIVE PRMT5 INHIBITORS: MECHANISM SWITCHING THROUGH STRUCTURE-BASED DESIGN

Kevin M. Cottrell*‡, Douglas A. Whittington‡, Kimberly J. Briggs‡, Haris Jahic‡, Janid A. Ali‡, Alvaro J. Amor‡, Deepali Gotur‡, Matthew R. Tonini‡, Wenhai Zhang‡, Alan Huang‡, and John P. Maxwell‡

*Corresponding Author kcottrell@tangotx.com
‡Tango Therapeutics, Boston, MA 02215, United States

Table of Contents

General experimental and chemical procedures……S1
NMR Spectra, HPLC/LCMS traces, and other analytical data of final compounds……S57
Biochemical fluorescence anisotropy peptide displacement assay……S240
HAP1 MTAP WT and MTAP-null In-Cell Western Assay……S240
HAP1 MTAP WT and MTAP-null Viability Assay……S240
Nano-luciferase Thermal Shift in Cell Lysates……S241
MDR1-MDCKII assay……S241
Human liver microsomes assay……S241
Kinetic solubility assay……S241
PRMT5:MEP50 expression and purification……S242
PRMT5:MEP50 SPR studies……S243
PRMT5:MEP50 crystallography……S244

General experimental and chemical procedures

All chemicals were provided by Enamine Ltd., WuXi Apptech, or other commercial suppliers and used as received unless otherwise indicated. All solvents were treated according to standard methods. All reactions were monitored and analysis of final compounds performed by LC-MS using Agilent 1260 LC/MSD instruments, with an Agilent Poroshell 120 SB-C18 4.6 x 30mm 2.7 µm column, column Temperature: 60 °C, mobile phase: A – H_2O (0.1% formic acid), B – ACN (0.1% formic acid), flow rate: 1.5 mL/min, gradient: 0.01 min – 1% B, 5.00 min – 100% B, 5.99 min – 100% B, MS Ionization mode: Electrospray ionization (ESI), MS Scan range: 83 – 1000 m/z, UV detection: 215 nm, 254 nm, 280 nm unless otherwise specified. Thin-layer chromatography (TLC) with pre-coated silica gel GF254 (0.2 mm) was used and the results were visualized using either UV light or $KMnO_4$ stain. Proton nuclear magnetic resonance (^{1}H-NMR) spectra were recorded at 400, 500 or 600 MHz on Varian or Bruker instrumentation; chemical shifts were calibrated using residual non-deuterated solvents $CDCl_3$ (δ = 7.26 ppm), DMSO-d_6 (δ = 2.50 ppm) or MeOH-d_4 (δ = 3.31 ppm) and expressed in δ ppm. Coupling constants (J), when given, are reported in hertz. Multiplicities are reported using the following abbreviations: s = singlet, d = doublet, dd = doublet of doublets, t = triplet, q =quartet, m = multiplet (range of multiplets is given), br = broad signal, dt = doublet of triplets. ^{13}C NMR spectra were recorded at 101, 126 or 151 MHz (Varian). ^{13}C NMR chemical shifts are reported relative to the central $CDCl_3$ (δ = 77.16 ppm), DMSO-d_6 (δ = 39.52 ppm) or MeOH-d_4 (δ = 49.00 ppm) and chemical shifts are reported in parts per million (ppm). All final compounds were purified by reverse phase high-performance liquid chromatography (HPLC) or supercritical fluid chromatography (SFC) or silica gel chromatography (100-200 mesh) unless otherwise specified. HPLC was done with an Agilent 1260 HPLC instrument (Agilent Technologies, Germany) equipped with a G7161A Preparative Binary Pump, a G7157A Prep Autosampler, a G7115A DAD WR, and a G7159B Preparative Fraction Collector unless otherwise specified. The Open Lab CDS software (version C.01.10) was used for instrument control, data acquisition, and data handling. SFC was done with a Waters 100q Prep SFC System unless otherwise specified. Chiral HPLC analytical analysis was done with an Agilent 1200 HPLC instrument (Agilent Technologies, Germany) equipped with a G1379B degasser, a G1312A Binary Pump, a G1329A ALS autosampler, a G1315A Diode Array Detector unless otherwise specified. Chiral SFC analytical analysis was done with an Agilent 1260 SFC instrument (Agilent Technologies, Germany) equipped with a G1379B degasser, a G1312B Binary Pump, a G1313A ALS autosampler, a G1316A thermostatted column compartment, a G1315D Diode Array Detector and an Aurora SFC systems unless otherwise specified. Melting points were taken using OptiMelt Automated Melting Point System Digital Image Processing Technology SRS Stanford Pesearch Systems, 2 ^{o}C/min (5 ^{o}C/min at high melting point). Optical rotation was measured with Polarimeter Anton Paar GmbH MCP 300 (Accuracy: ±0.003°) used to measure the

angle of optical rotation. Standard conditions for analysis: solution concentration 0.5 g/100 mL (solvent: MeOH), wavelength 589 nm, temperature 21 °C. All final compounds are > 95% pure by HPLC. Compound 1 (GSK3203591/EPZ015866) purchased from MedChemExpress.

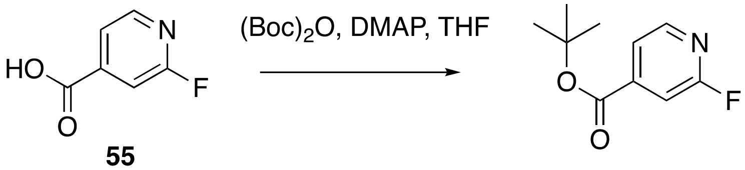

55

tert-butyl 2-fluoroisonicotinate

To a solution of 2-fluoropyridine-4-carboxylic acid **55** (20 g, 141.74 mmol, 1 eq) and DMAP (3.46 g, 28.35 mmol, 0.2 eq) in THF (150 mL) was added $(Boc)_2O$ (68.0 g, 311.57 mmol, 2.2 eq) at 0 °C. The mixture was stirred at 25 °C for 12 h. The resulting mixture was quenched by addition of $NaHCO_3$ (20 mL) and extracted with EtOAc (150 mL x 3). The combined organic layer was dried over anhydrous Na_2SO_4, filtered, and concentrated under reduced pressure to afford *tert*-butyl 2-fluoropyridine-4-carboxylate (37 g, crude) which was used in the next step without further purification. ^{1}H NMR (400 MHz, CDCl3) δ ppm 8.30 (d, J = 5.0 Hz, 1 H), 7.67 (dt, J = 5.0, 1.5 Hz, 1 H), 7.40 (d, J = 1.0 Hz, 1 H), 1.58 (s, 9 H); LCMS (M+H$^+$) m/z: calcd 198.1; found 198.1.

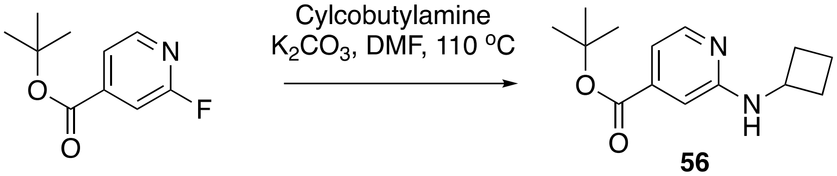

56

tert-butyl 2-(cyclobutylamino)isonicotinate (56)

To a mixture of *tert*-butyl 2-fluoropyridine-4-carboxylate (70.87 mmol, crude product, 1 eq) and cyclobutanamine (15 mL, 175.05 mmol, 2.5 eq) in DMF (150 mL) was added K_2CO_3 (29.38 g, 212.61 mmol, 3 eq). The mixture was stirred at 110 °C for 12 h. The resulting mixture was filtered and diluted with EtOAc (100 mL x 3). The organic layers were washed with brine (150 mL x 3), dried over anhydrous Na_2SO_4, filtered, and concentrated under reduced pressure. The residue was purified by column chromatography (silica, petroleum ether/EtOAc = 30:1 to 10:1) to afford *tert*-butyl 2-(cyclobutylamino) pyridine-4-carboxylate **56** (12 g, 31% for two steps) as a yellow oil. ^{1}H NMR (400 MHz, $CDCl_3$) δ ppm 8.15 (d, J = 5.0 Hz, 1 H), 7.03 (dd, J = 5.3, 1.3 Hz, 1 H), 6.84 (s, 1 H), 4.85 (d, J = 6.0 Hz, 1 H), 4.10 - 4.23 (m, 1 H), 2.41 - 2.51 (m, 2 H), 1.69 - 1.94 (m, 4 H), 1.59 (s, 9 H); LCMS (M+H$^+$) m/z: calcd 249.2; found 249.0.

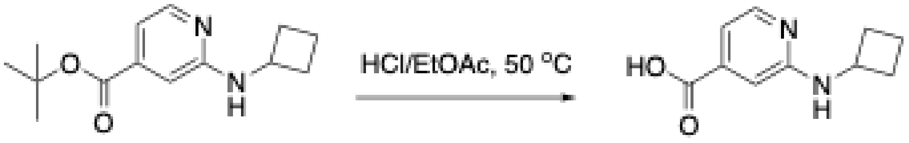

56 **70**

2-(cyclobutylamino)isonicotinic acid (**70**)

A solution of *tert*-butyl 2-(cyclobutylamino) pyridine-4-carboxylate, **56** (12 g, 43.49 mmol, 1 eq) in HCl/EtOAc (250 mL, 4M) was stirred at 50 °C for 12 h. The precipitate was collected by filtration, washed with EtOAc (30 mL x 3) and dried under high vacuum to afford 2-(cyclobutylamino)pyridine-4-carboxylic acid, **70** (9.9 g, HCl salt, 99% yield) as a yellow solid. ^{1}H NMR (400 MHz, DMSO-d_6) δ ppm 9.27 (br s, 1 H), 7.99 (d, J = 6.3 Hz, 1 H), 7.41 (s, 1 H), 7.06 (dd, J = 6.5, 1.3 Hz, 1 H), 4.29 (t, J = 7.5 Hz, 1 H), 2.37 - 2.46 (m, 2 H), 1.94 - 2.07 (m, 2 H), 1.65 - 1.85 (m, 2 H); LCMS (M+H$^+$) m/z: calcd 193.1; found 193.4.

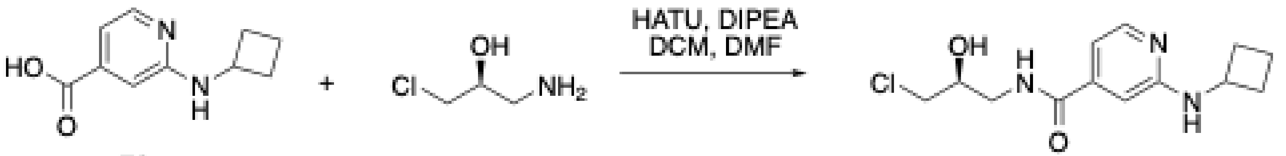

70

57

(S)-N-(3-chloro-2-hydroxypropyl)-2-(cyclobutylamino)isonicotinamide (57)
To a mixture of **70** (8.5 g, 37.17 mmol, 1 eq, HCl) in DCM (130 mL) and DMF (35 mL) were added HATU (21.20 g, 55.76 mmol, 1.5 eq) and DIPEA (24.02 g, 185.85 mmol, 32.37 mL, 5 eq) sequentially. After stirring 15 minutes, (2S)-1-amino-3-chloro-propan-2-ol (5.97 g, 40.89 mmol, 1.1 eq, HCl) was added and the mixture was stirred for 12 h at 25 °C. The reaction mixture was concentrated under reduced pressure to remove DCM. The residue was diluted with H_2O (150 mL) and extracted with EtOAc (70 mL x 4). The combined organic layers were washed with saturated NH_4Cl aqueous solution (100 mL), brine (100 mL), dried over anhydrous Na_2SO_4, filtered and concentrated under reduced pressure. The residue was purified by flash chromatography (ISCO®; 40 g SepaFlash® Silica Flash Column, petroleum ether/EtOAc with EtOAc from 0~95%, flow rate = 35 mL/min) to afford **57** N-[(2S)-3-chloro-2-hydroxy-propyl]-2-(cyclobutylamino)pyridine-4-carboxamide (12.1 g, 92 % yield) as yellow solid. ^{1}H NMR (400 MHz, MeOH-d_4) δ ppm 7.98 - 8.05 (m, 1 H), 6.79 - 6.88 (m, 2 H), 4.25 (quin, J = 7.8 Hz, 1 H), 3.95 - 4.04 (m, 1 H), 3.53 - 3.70 (m, 3 H), 3.41 - 3.48 (m, 1 H), 2.35 - 2.45 (m, 2 H), 1.89 - 1.98 (m, 2 H), 1.76 – 1.85 (m, 2 H); LCMS (ESI) [M+H]$^+$ calcd 284.1, found 284.4.

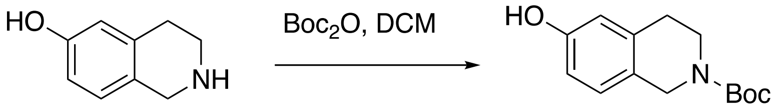

58

tert-butyl 6-hydroxy-3,4-dihydroisoquinoline-2(1H)-carboxylate (**58**)
To a suspension of 1,2,3,4-tetrahydroisoquinolin-6-ol (19.86 g, 133.12 mmol) in dichloromethane (300 mL), cooled to 0 °C was added dropwise di-*tert*-butyl dicarbonate (29.63 g, 135.78 mmol) in dichloromethane (80 mL), the reaction mixture was stirred for 12 h at room temperature. The reaction mixture was evaporated, poured into water, extracted with dichloromethane (300 mL), dried over Na_2SO_4, and evaporated to afford the *tert*-butyl 6-hydroxy-3,4-dihydro-1H-isoquinoline-2-carboxylate **58** (29.8 g, 119.53 mmol, 90 % yield).
^{1}H NMR (400 MHz, DMSO-d_6) δ (ppm) 1.41 (s, 9H), 2.66 (t, 2H), 3.48 (t, 2H), 4.35 (s, 2H), 6.53 (s, 1H), 6.58 (d, 1H), 6.92 (d, 1H), 9.21 (s, 1H). LCMS(ESI): [M+H]$^+$ m/z: calcd 250.1; found 250.1; Rt = 0.75 min.

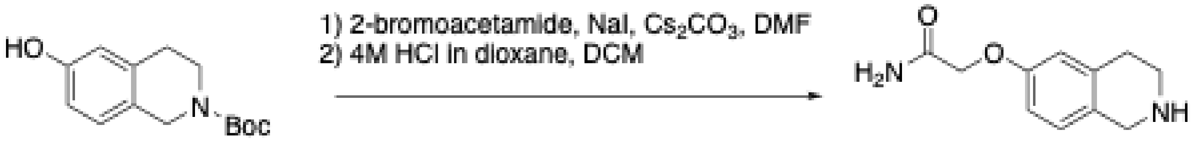

58

Intermediate 15a

2-((1,2,3,4-tetrahydroisoquinolin-6-yl)oxy)acetamide, **intermediate 15a**
tert-butyl 6-hydroxy-3,4-dihydro-1H-isoquinoline-2-carboxylate (1.5 g, 6.02 mmol), sodium iodide (180.37 mg, 1.20 mmol), and cesium carbonate (3.92 g, 12.03 mmol) were mixed together in DMF (20 mL). The resulting suspension was stirred at 25 °C for 30 min, then 2-bromoacetamide (1.04 g, 7.52 mmol) was added, and the reaction mixture was stirred at 50 °C for 24 h, cooled, diluted with water (100 mL), and stirred for 1 h. The precipitate was isolated by filtration, washed with water (2 x 10 mL), and dried in vacuo to afford *tert*-butyl 6-(2-amino-2-oxo-ethoxy)-3,4-dihydro-1H-isoquinoline-2-carboxylate (1.3 g, 4.24 mmol, 71% yield) as a white solid. ^{1}H NMR (500 MHz, DMSO-d_6) δ (ppm) 1.41 (s, 9H), 2.72 (m, 2H), 3.50 (m, 2H), 4.37 (s, 2H), 4.40 (s, 2H), 6.74 (s, 1H), 6.78 (dd, 1H), 7.07 (d, 1H), 7.35 (s, 1H), 7.46 (s, 1H). LCMS(ESI): [M+H]+ m/z: calcd 306.1; found 307.1; Rt = 1.22 min. 4.0M hydrogen chloride solution in dioxane (84.87 mmol, 20.18 mL, 15%) was added to a solution of *tert*-butyl 6-(2-amino-2-oxo-ethoxy)-3,4-dihydro-1H-isoquinoline-2-carboxylate (1.3 g, 4.24 mmol) in dichloromethane (30 mL). The resulting mixture was stirred at 25 °C for 12 h. The precipitate was isolated by

filtration, washed with dichloromethane (20 mL), and dried in vacuo to afford 2-(1,2,3,4-tetrahydroisoquinolin-6-yloxy)acetamide, **intermediate 15a** (1 g, 4.12 mmol, 97% yield, HCl) as a white solid. ^{1}H NMR (400 MHz, DMSO-*d$_6$*) *δ* (ppm) 2.96 (m, 2H), 3.30 (m, 2H), 4.14 (s, 2H), 4.40 (s, 2H), 6.79 (s, 1H), 6.83 (dd, 1H), 7.13 (d, 1H), 7.37 (s, 1H), 7.52 (s, 1H). LCMS(ESI): [M+H]+ m/z: calcd 206.1; found 207.1; Rt = 0.37 min.

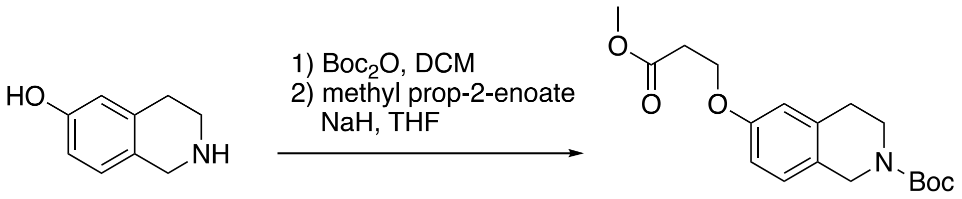

tert-butyl 6-(3-methoxy-3-oxopropoxy)-3,4-dihydroisoquinoline-2(1*H*)-carboxylate
To a suspension of 1,2,3,4-tetrahydroisoquinolin-6-ol (8 g, 53.62 mmol) in dichloromethane (150 mL), which was cooled to 0 °C, was added dropwise di-*tert*-butyl dicarbonate (13.11 g, 60.06 mmol) in dichloromethane (50 mL), the reaction mixture was stirred for 12 h at room temperature, after which time it was concentrated in vacuo. The residue was triturated with n-hexane (70 mL). The resulting precipitate was filtered and the filtrate concentrated in vacuo to provide *tert*-butyl 6-hydroxy-3,4-dihydro-1*H*-isoquinoline-2-carboxylate (11.5 g, 46.13 mmol, 86% yield). ^{1}H NMR (400 MHz, DMSO-*d$_6$*) *δ* (ppm) 1.41 (s, 9H), 2.66 (t, 2H), 3.48 (t, 2H), 4.35 (s, 2H), 6.53 (s, 1H), 6.58 (d, 1H), 6.92 (d, 1H), 9.21 (s, 1H). LCMS(ESI): [M+H]+ m/z: calcd 250.1; found 250.1; Rt = 0.75 min. To a solution of the *tert*-butyl 6-hydroxy-3,4-dihydro-1*H*-isoquinoline-2-carboxylate (4 g, 16.04 mmol) and methyl prop-2-enoate (20.72 g, 240.67 mmol, 21.67 mL) in THF (15 mL) was added sodium hydride (60% dispersion in mineral oil) (19.25 mg, 802.23 umol) at room temperature. The reaction mixture was stirred for 24 h at 70 °C, then the reaction mixture was concentrated in vacuo to give 4.7 g of crude product which was purified by column chromatography on silica gel (hexane-MTBE as eluents) to give *tert*-butyl 6-(3-methoxy-3-oxo-propoxy)-3,4-dihydro-1*H*-isoquinoline-2-carboxylate (3 g, 8.94 mmol, 56 % yield). ^{1}H NMR (400 MHz, DMSO-d6) δ (ppm) 1.43 (s, 9H), 2.75 (m, 4H), 3.53 (t, 2H), 3.65 (s, 3H), 4.15 (t, 2H), 4.39 (s, 2H), 6.68 (m, 2H), 6.98 (d, 1H).

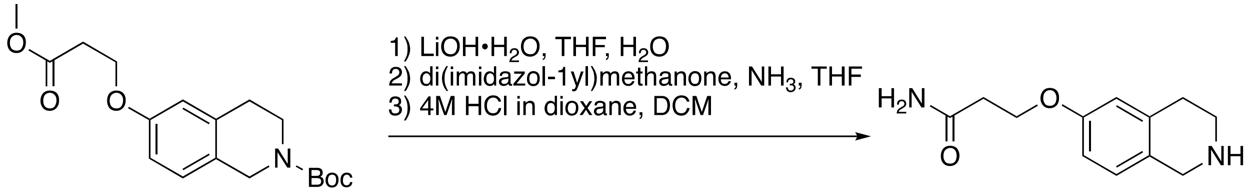

Intermediate 18a

3-((1,2,3,4-tetrahydroisoquinolin-6-yl)oxy)propenamide, **intermediate 18a**
A mixture of *tert*-butyl 6-(3-methoxy-3-oxo-propoxy)-3,4-dihydro-1*H*-isoquinoline-2-carboxylate (3.4 g, 10.14 mmol) and lithium hydroxide, monohydrate (446.67 mg, 10.64 mmol) in THF (40 mL) – water (40 mL) was stirred at room temperature for 24 h. The mixture was concentrated under reduced pressure and H_2O (100 mL) was added. The aqueous layer was washed with MTBE (50 mL), then acidified ($NaHSO_4$, aq., 5 %) to pH 3 and the product was extracted with DCM (2 x 50 mL). The combined organic layers were dried over Na_2SO_4 and concentrated in vacuo to give the 3-[(2-*tert*-butoxycarbonyl-3,4-dihydro-1*H*-isoquinolin-6-yl)oxy]propanoic acid (1.8 g, 5.60 mmol, 55% yield) as a yellow solid. ^{1}H NMR (400 MHz, $CDCl_3$) *δ* (ppm) 1.47 (s, 9H), 2.81 (m, 4H), 3.62 (m, 2H), 4.22 (t, 2H), 4.49 (s, 2H), 6.67 (s, 1H), 6.75 (d, 1H), 7.00 (d, 1H). LCMS(ESI): [M-Boc]+ m/z: calcd 321.1; found 222.2; Rt = 1.35 min. To a solution of 3-[(2-*tert*-butoxycarbonyl-3,4-dihydro-1*H*-isoquinolin-6-yl)oxy]propanoic acid (1.7 g, 5.29 mmol) in THF (80 mL) was added di(imidazol-1-yl)methanone (1.03 g, 6.35 mmol), and the resulting mixture was stirred at room temperature for 4 h. NH_3 was then carefully bubbled through the stirred, cooled (0 °C) reaction solution for 5 min. The reaction mixture was then stirred for 12 h at room temperature. The solvent was evaporated in vacuo and the residue was taken up with DCM (100 mL). The organic layer was washed successively with $NaHCO_3$ aq., 5% aqueous (30 mL), and $NaHSO_4$ aq., 5% aqueous (30 mL), dried over Na_2SO_4, and concentrated in vacuo to give *tert*-butyl 6-(3-amino-3-oxo-propoxy)-3,4-dihydro-1*H*-isoquinoline-2-carboxylate (1.5 g, 4.68 mmol, 89% yield). ^{1}H NMR (400 MHz, $CDCl_3$) *δ* (ppm) 1.46 (s, 9H), 2.68 (t, 2H), 2.77 (m, 2H), 3.59 (m, 2H), 4.22 (t, 2H), 4.48 (s, 2H), 5.62 (s, 1H), 5.75 (s, 1H), 6.67 (s, 1H), 6.73 (d, 1H), 7.01 (d, 1H). LCMS(ESI): [M-Boc]+ m/z: calcd 320.1; found 221.2; Rt = 1.28 min.
4.0M hydrogen chloride solution in dioxane (48.38 mmol, 12.00 mL, 14% purity) was carefully added to a solution of *tert*-butyl 6-(3-amino-3-oxo-propoxy)-3,4-dihydro-1*H*-isoquinoline-2-carboxylate (1.55 g, 4.84 mmol) at room

temperature in DCM (100 mL). The reaction mixture was then stirred for 12 h. The solvents were evaporated in vacuo to give 3-(1,2,3,4-tetrahydroisoquinolin-6-yloxy)propanamide, **intermediate 18a** (1.4 g, crude, HCl) as a white solid. ^{1}H NMR (400 MHz, DMSO-*d*$_6$) *δ* (ppm) 2.49 (s, 2H), 2.65 (t, 2H), 3.31 (m, 2H), 4.14 (m, 4H), 6.78 (s, 1H), 6.81 (d, 1H), 6.88 (m, 1H), 7.10 (d, 1H), 7.42 (s, 1H), 9.29 (s, 2H). LCMS(ESI): [M+H]+ m/z: calcd 220.1; found 221.2; Rt = 0.48 min.

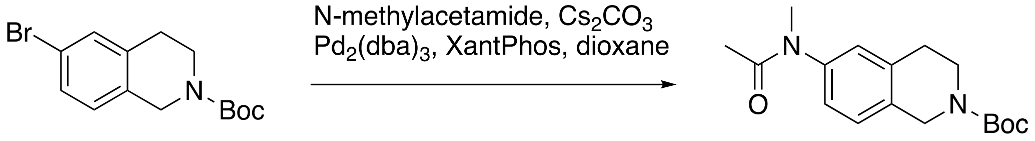

tert-butyl 6-(*N*-methylacetamido)-3,4-dihydroisoquinoline-2(1*H*)-carboxylate
tert-butyl 6-bromo-3,4-dihydro-1*H*-isoquinoline-2-carboxylate (4 g, 12.81 mmol), *N*-methylacetamide (1.12 g, 15.37 mmol), and cesium carbonate (6.26 g, 19.22 mmol) were placed in the Schlenk flask, and dioxane (40 mL) was added. The flask was evacuated, degassed in an ulatrsonic bath, and purged with argon. Tris(dibenzylideneacetone)dipalladium(0) (234.65 mg, 256.24 umol) and 4,5-bis(diphenylphosphino)-9,9-dimethyl xanthene (593.07 mg, 1.02 mmol) were added under the stream of Ar, the reaction mixture was heated to 100 °C and stirred for 18 h. After cooling to 20 °C, H_2O (50 mL) was added. The aqueous layer was separated and extracted with ethyl acetate (50 mL), organic layers were combined and washed with brine (100 mL), dried over Na_2SO_4, and concentrated under reduced pressure, affording crude product as a light-brown oil. The product was purified by column chromatograph to give *tert*-butyl 6-[acetyl(methyl)amino]-3,4-dihydro-1*H*-isoquinoline-2-carboxylate (1 g, 3.29 mmol, 26 % yield). ^{1}H NMR (400 MHz, $CDCl_3$) *δ* (ppm) 1.47 (s, 9H), 1.85 (s, 3H), 2.81 (s, 2H), 3.21 (s, 3H), 3.64 (s, 2H), 4.56 (s, 2H), 6.96 (m, 2H), 7.13 (m, 1H). LCMS(ESI): $[M+H]^+$ m/z: calcd 304.3; found 305.1; Rt = 1.29 min.

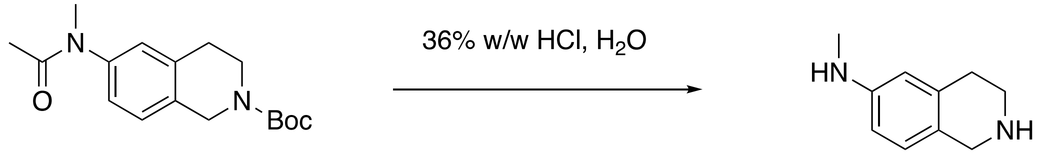

intermediate 5a

N-methyl-1,2,3,4-tetrahydroisoquinolin-6-amine, **intermediate 5a**
tert-butyl 6-[acetyl(methyl)amino]-3,4-dihydro-1*H*-isoquinoline-2-carboxylate (0.75 g, 2.46 mmol) was suspended in water (10 mL) and hydrochloric acid, 36% w/w aq. soln. (8.00 g, 219.41 mmol, 10 mL) was added. The suspension was heated to 80 °C and stirred at this temperture for 18 h. The mixture was cooled to 20 °C, water (20 mL) was added, then it was washed with ethyl acetate (2 x 20 mL). KOH, 50 % aq. solution (30 mL) was added to the aqueous layer and obtained emulsion was extracted with DCM (2 x 50 mL). The organic layers were combined and washed with brine (50 mL), dried over Na_2SO_4, and concentrated under reduced pressure affording *N*-methyl-1,2,3,4-tetrahydroisoquinolin-6-amine, **intermediate 5a** (0.27 g, 1.66 mmol, 68 % yield). ^{1}H NMR (400 MHz, $CDCl_3$) *δ* (ppm) 2.09 (s, 1H), 2.73 (m, 2H), 2.81 (s, 3H), 3.11 (m, 2H), 3.55 (s, 1H), 3.92 (s, 2H), 6.34 (s, 1H), 6.45 (d, 1H), 6.84 (d, 1H). LCMS(ESI): $[M+H]^+$ m/z: calcd 162.1; found 163.2; Rt = 0.18 min.

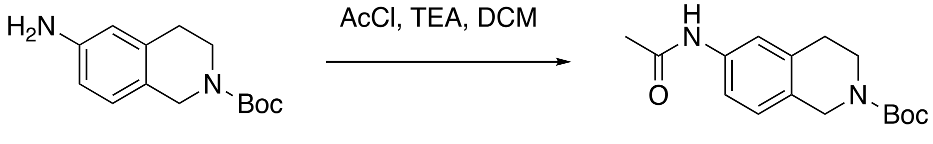

tert-butyl 6-acetamido-3,4-dihydroisoquinoline-2(1*H*)-carboxylate
To a solution of *tert*-butyl 6-amino-3,4-dihydro-1*H*-isoquinoline-2-carboxylate (0.8 g, 3.22 mmol) and triethylamine (977.99 mg, 9.66 mmol, 1.35 mL) in DCM (60 mL), acetyl chloride (303.48 mg, 3.87 mmol, 235.26 uL) was added dropwise at 0 °C. The resulting mixture was stirred at room temperature for 12 h and then washed with a solution of $NaHSO_4$ (2 x 30 mL) and brine (2 x 30mL). The organic layer was dried over Na_2SO_4 and evaporated in vacuo to give *tert*-butyl 6-acetamido-3,4-dihydro-1*H*-isoquinoline-2-carboxylate (0.9 g, 3.10 mmol, 96% yield). ^{1}H NMR

(500 MHz, DMSO-d_6) δ (ppm) 1.39 (s, 9H), 1.99 (s, 3H), 2.70 (m, 2H), 3.49 (m, 2H), 4.39 (s, 2H), 7.04 (d, 1H), 7.30 (d, 1H), 7.40 (s, 1H), 9.83 (s, 1H). LCMS(ESI): $[M+H]^+$ m/z: calcd 290.1; found 291.1; Rt = 1.19 min.

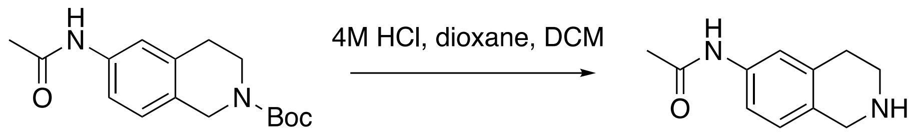

intermediate 10a

N-(1,2,3,4-tetrahydroisoquinolin-6-yl)acetamide, **intermediate 10a**
To a solution of *tert*-butyl 6-acetamido-3,4-dihydro-1*H*-isoquinoline-2-carboxylate (0.9 g, 3.10 mmol) in DCM (50 mL), 4.0 M hydrogen chloride solution in dioxane (7 g, 31.00 mmol) was added. The resulting mixture was stirred at 25 °C for 24 h. The solvent was evaporated in vacuo and the residue was triturated with CH_3CN (20 mL). The precipitate was filtered and washed with CH_3CN (10 m), MTBE (2 x 20 mL) and dried in vacuo to give N-(1,2,3,4-tetrahydroisoquinolin-6-yl)acetamide, **intermediate 10a** (0.66 g, 2.91 mmol, 94 % yield, HCl). ^1H NMR (500 MHz, DMSO-d_6) δ (ppm) 2.03 (s, 3H), 2.95 (m, 2H), 3.31 (m, 2H), 4.16 (s, 2H), 7.11 (d, 1H), 7.38 (d, 1H), 7.50 (s, 1H), 9.46 (s, 2H), 10.07 (s, 1H). LCMS(ESI): $[M+H]^+$ m/z: calcd 190.1; found 191.2; Rt = 0.34 min.

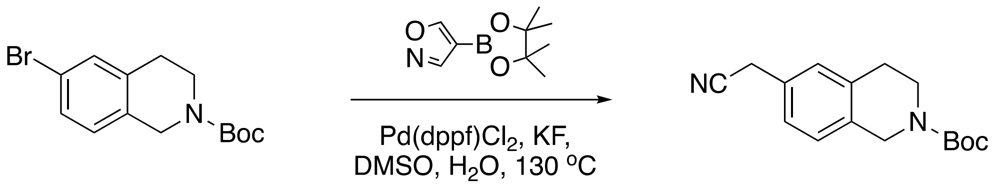

tert-butyl 6-(cyanomethyl)-3,4-dihydroisoquinoline-2(1*H*)-carboxylate
tert-butyl 6-bromo-3,4-dihydro-1*H*-isoquinoline-2-carboxylate (350 mg, 1.12 mmol), KF (195.4 mg, 3.36 mmol), Pd(dppf)Cl_2 (82.0 mg, 0.11 mmol), 4-(4,4,5,5-tetramethyl-1,3,2-dioxaborolan-2-yl) isoxazole (546.6 mg, 2.80 mmol), H_2O (2 mL) and DMSO (20 mL) were added to a round bottom flask. The mixture was degassed and backfilled with N_2 three times and then stirred for 12 h at 130 °C under N_2. The resulting mixture was diluted with EtOAc (250 mL) and the organic layer was washed with water (60 mL x 2), then brine (60 mL x 3), then dried over anhydrous Na_2SO_4, filtered, and concentrated under reduced pressure. The residue was purified by flash chromatography (ISCO®; 20 g of SepaFlash® Silica Flash Column, petroleum ether/EtOAc with EtOAc from 0 ~ 35 %, Flow rate: 35 mL / min) to afford *tert*-butyl 6-(cyanomethyl)-3,4-dihydro-1*H*-isoquinoline-2-carboxylate (250 mg, 74 % yield) as a yellow oil. ^1H NMR (400 MHz, DMSO-d_6) δ ppm 7.13 - 7.21 (m, 3 H), 4.48 (s, 2 H), 3.98 (s, 2 H), 3.54 (t, J = 5.9 Hz, 2 H), 2.78 (t, J = 5.9 Hz, 2 H), 1.39 - 1.45 (m, 9 H).

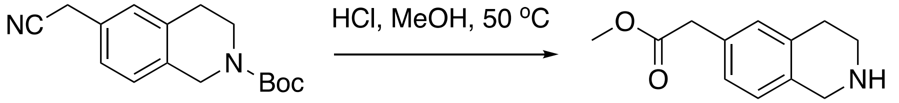

intermediate 11a

methyl 2-(1,2,3,4-tetrahydroisoquinolin-6-yl)acetate, **intermediate 11a**
To a solution of *tert*-butyl 6-(cyanomethyl)-3,4-dihydro-1*H*-isoquinoline-2-carboxylate (220 mg, 0.73 mmol) in MeOH (10 mL) was added 4M HCl/MeOH (10 mL). The mixture was stirred at 50 °C for 12 h. The resulting mixture was concentrated under reduced pressure and dissolved in H_2O (5 mL). The aqueous was adjusted to pH = 7 by addition of saturated $NaHCO_3$ anqueous solution and then extracted with DCM (100 mL x 4). The combined organic layer was dried over anhydrous Na_2SO_4, filtered, and concentrated under reduced pressure to afford methyl 2-(1,2,3,4-tetrahydroisoquinolin-6-yl)acetate, **intermediate 11a** (120 mg, 72 % yield) as a red oil. ^1H NMR (400 MHz, MeOH-d_4) δ ppm 6.98 - 7.08 (m, 3 H), 3.95 (s, 2 H), 3.68 (s, 3 H), 3.59 (s, 2 H), 3.09 (t, J = 6.0 Hz, 2 H), 2.83 (t, J = 6.0 Hz, 2 H); LCMS (ESI) [M+H]+ m/z: calcd 206.1; found 206.1.

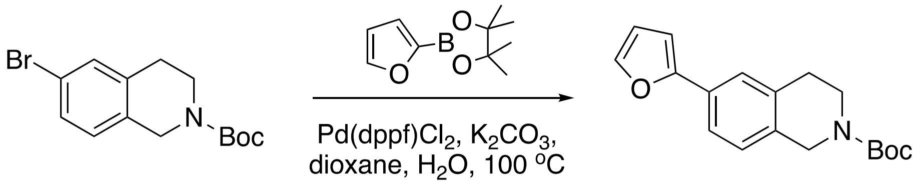

tert-butyl 6-(furan-2-yl)-3,4-dihydroisoquinoline-2(1*H*)-carboxylate
To a solution of *tert*-butyl 6-bromo-3,4-dihydro-1*H*-isoquinoline-2-carboxylate (500 mg, 1.60 mmol, 1 eq) and 2-(2-furyl)-4,4,5,5-tetramethyl-1,3,2-dioxaborolane (375 mg, 1.90 mmol, 1.21 eq) in dioxane (10 mL) and H_2O (0.5 mL) was added Pd(dppf)Cl_2 (100 mg, 0.14 mmol) and K_2CO_3 (663 mg, 4.8 mmol, 3.0 eq), and the mixture was stirred at 100 °C for 12 h. The mixture was diluted with water (20 mL) and then extracted with EtOAc (20 mL x 3). The combined organic layers were washed with saturated NH_4Cl aqueous solution (20 mL x 2), brine (20 mL), dried over anhydrous Na_2SO_4, filtered and concentrated under reduced pressure. The residue was purified by flash chromatography (ISCO®; 24 g SepaFlash® Silica Flash Column, petroleum ether/EtOAc with EtOAc from 0 ~ 10 %, flow rate: 30 mL / min) to afford *tert*-butyl 6-(2-furyl)-3,4-dihydro-1*H*-isoquinoline-2-carboxylate (350 mg, 69 % yield) as light yellow oil. ^{1}H NMR (400 MHz, MeOH-*d$_4$*) δ ppm 7.47 - 7.58 (m, 3 H), 7.16 (d, *J* = 7.8 Hz, 1 H), 6.74 (d, *J* = 3.3 Hz, 1 H), 6.51 (dd, *J* = 3.3, 1.8 Hz, 1 H), 4.57 (s, 2 H), 3.67 (t, *J* = 5.8 Hz, 2 H), 2.88 (t, *J* = 5.9 Hz, 2 H), 1.52 (s, 9 H); LCMS (ESI) $[M+H-56]^+$ m/z calcd 244.2, found 244.0.

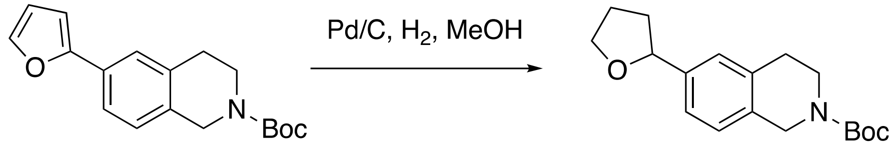

tert-butyl 6-(tetrahydrofuran-2-yl)-3,4-dihydroisoquinoline-2(1*H*)-carboxylate
To a solution of *tert*-butyl 6-(2-furyl)-3,4-dihydro-1*H*-isoquinoline-2-carboxylate (300 mg, 1.0 mmol, 1 eq) in MeOH (5 mL) was added Pd/C (30 mg, 10 % of Pd with 50 % of water, wt %). The mixture was degassed and backfilled with N_2 three times and then repeated with H_2 three times. The reaction mixture was stirred at 25 °C under H_2 balloon for 12 h. The resulting mixture was filtered through celite, washed with MeOH (10 mL x 2), and concentrated under reduced pressure. The residue was purified by flash chromatography (ISCO®; 12 g of SepaFlash® Silica Flash Column, petroleum ether/EtOAc with EtOAc from 0 ~ 10 %, flow rate: 30 mL / min) to afford *tert*-butyl 6-tetrahydrofuran-2-yl-3,4-dihydro-1*H*-isoquinoline-2-carboxylate (240 mg, 75 % yield) as colorless oil. ^{1}H NMR (400 MHz, MeOH-*d$_4$*) δ ppm 6.99 - 7.29 (m, 3 H), 4.84 (t, J = 7.2 Hz, 1 H), 4.55 (br s, 2 H), 4.05 - 4.19 (m, 1 H), 3.84 - 3.97 (m, 1 H), 3.63 (br d, J = 5.3 Hz, 2 H), 2.84 (t, J = 5.9 Hz, 2 H), 2.23 - 2.43 (m, 1 H), 1.95 - 2.12 (m, 2 H), 1.68 - 1.84 (m, 1 H), 1.51 (s, 9 H); LCMS (ESI) [M+H-56]+ calcd 248.2, found 248.1.

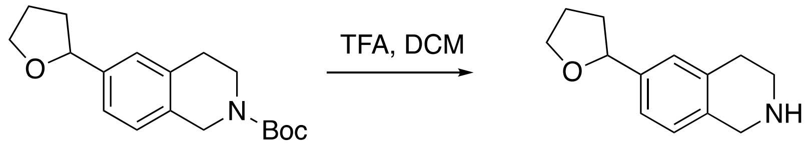

intermediate 12a

6-(tetrahydrofuran-2-yl)-1,2,3,4-tetrahydroisoquinoline, **intermediate 12a**
To a solution of *tert*-butyl 6-tetrahydrofuran-2-yl-3,4-dihydro-1*H*-isoquinoline-2-carboxylate (235 mg, 0.77 mmol, 1 eq) in DCM (6 mL) was added TFA (0.8 mL). The mixture was stirred at 25 °C for 1 h. The reaction mixture was cooled to 0 °C and adjust to pH = 8 with saturated $NaHCO_3$ aqueous solution. The aqueous mixture was extracted with DCM (10 mL x 3) and the combined organic layers were washed with brine (20 mL), dried over anhydrous Na_2SO_4, filtered, and concentrated under reduced pressure to afford 6-tetrahydrofuran-2-yl-1,2,3,4-tetrahydroisoquinoline, **intermediate 12a** (140 mg, crude) as yellow oil which was used in next step without purification. LCMS (ESI) $[M+H]^+$ m/z calcd 204.1, found 204.1.

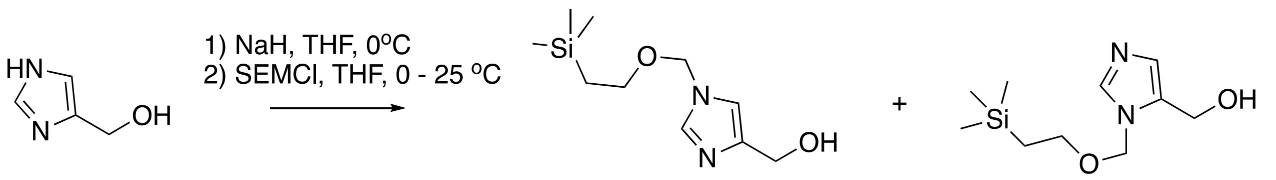

mixture, intermediate 30a'

(1-((2-(trimethylsilyl)ethoxy)methyl)-1*H*-imidazol-4-yl)methanol and (1-((2-(trimethylsilyl)ethoxy)methyl)-1*H*-imidazol-5-yl)methanol, **intermediate 30a'**

To a solution of 1*H*-imidazol-4-ylmethanol (1.7 g, 17.0 mmol) in THF (15 mL) was added NaH (1.04 g, 26.0 mmol, 60% in mineral oil, wt%) and the mixture was stirred at 0 °C for 0.5 h. A solution of SEMCl (3.03 g, 18.0 mmol) in THF (5 mL) was added dropwise over 5 minutes. Then the mixture was stirred at 25 °C for 12 h. The resulting mixture was quenched by addition of water (20 mL) and extracted with EtOAc (20 mL x 3). The combined organic layer was dried over anhydrous Na_2SO_4, filtered, and concentrated under reduced pressure to give a crude product, which was purified by flash chromatography (ISCO®; 12 g AgelaFlash® Silica Flash Column, petroleum ether/EtOAc with EtOAc from 0~70%, 30 mL/min) to afford a mixture (1.4 g, 35% yield) of (1-((2-(trimethylsilyl)ethoxy)methyl)-1*H*-imidazol-4-yl)methanol and (1-((2-(trimethylsilyl)ethoxy)methyl)-1*H*-imidazol-5-yl)methanol, **intermediate 30a'**, as a yellow oil. LCMS (ESI) [M+H]+ m/z: calcd 229.1, found 229.1.

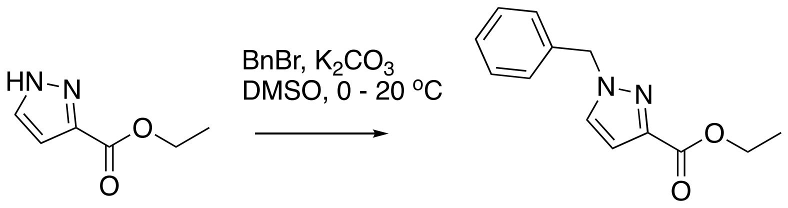

ethyl 1-benzyl-1*H*-pyrazole-3-carboxylate

A mixture of ethyl 1*H*-pyrazole-3-carboxylate (4 g, 28.54 mmol) and K_2CO_3 (4.73 g, 34.25 mmol) in DMSO (50 mL) was stirred at 0 °C, and then bromomethylbenzene (31.99 mmol, 3.8 mL) was added slowly. After addition was complete, the mixture was allowed to warm to 20 °C and stirred for 16 h. The resulting mixture was quenched by addition of water (100 mL) and extracted with EtOAc (100 mL x 3). The combined organic layer was washed with saturated LiCl aqueous solution (100 mL x 2), brine (100 mL), dried over anhydrous Na_2SO_4, filtered, and concentrated under reduced pressure. The residue was purified by flash chromatography (ISCO®; 40 g AgelaFlash® Silica Flash Column, petroleum ether/EtOAc with EtOAc from 0 ~ 30 %, Flow Rate: 30 mL / min) to afford ethyl 1-benzylpyrazole-3-carboxylate (4.2 g, 64 % yield, colorless oil). ^{1}H NMR (400 MHz, MeOH-d_4) δ ppm 7.71 (d, J = 2.3 Hz, 1 H), 7.24 - 7.37 (m, 5 H), 6.81 (d, J = 2.5 Hz, 1 H), 5.38 (s, 2 H), 4.34 (q, J = 7.1 Hz, 2 H), 1.35 (t, J = 7.2 Hz, 3 H); LCMS (ESI) [M+H]+ m/z: calcd 231.1, found 231.1. ethyl 2-benzylpyrazole-3-carboxylate (1.5 g, 23 % yield, colorless oil). ^{1}H NMR (400 MHz, MeOH-d_4) δ ppm 7.56 (d, J = 2.0 Hz, 1 H), 7.11 - 7.31 (m, 5 H), 6.91 (d, J = 2.0 Hz, 1 H), 5.74 (s, 2 H), 4.27 (q, J = 7.1 Hz, 2 H), 1.28 (t, J = 7.2 Hz, 3 H); LCMS (ESI) [M+H]+ m/z: calcd 231.1, found 231.1.

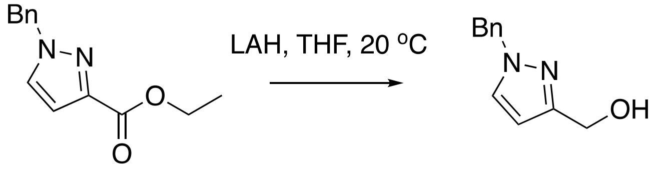

(1-benzyl-1*H*-pyrazol-3-yl)methanol

A mixture of ethyl 1-benzylpyrazole-3-carboxylate (2 g, 8.69 mmol) and LAH (396 mg, 10.42 mmol), THF (20 mL) was stirred at 20 °C for 16 h. The mixture was quenched with water (1 mL) slowly, dried over Na_2SO_4, filtered, and concentrated under reduced pressure to afford (1-benzylpyrazol-3-yl)methanol, intermediate 33a' (1.6 g, 98 % yield) as a colorless oil. ^{1}H NMR (400 MHz, MeOH-d_4) δ ppm 7.60 (d, J = 2.3 Hz, 1 H), 7.25 - 7.36 (m, 5 H), 6.33 (d, J = 2.3 Hz, 1 H), 5.26 (s, 2 H), 4.57 (s, 2 H); LCMS (ESI) [M+H]+ m/z: calcd 189.1, found 189.1.

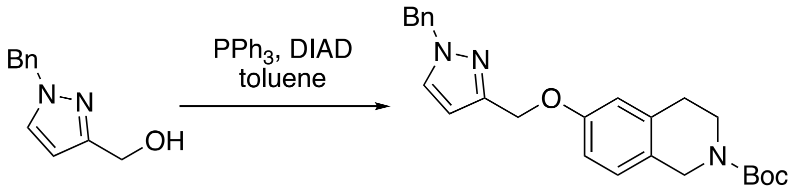

6-((1-benzyl-1*H*-pyrazol-3-yl)methoxy)-1,2,3,4-tetrahydroisoquinoline
To a solution of (1-benzylpyrazol-3-yl)methanol (189 mg, 1.00 mmol), *tert*-butyl 6-hydroxy-3,4-dihydro-1*H*-isoquinoline-2-carboxylate (250 mg, 1.00 mmol), triphenylphosphane (396 mg, 1.51 mmol) in toluene (1 mL) was added a solution of isopropyl (NE)-N-isopropoxycarbonyliminocarbamate (302 mg, 1.49 mmol) in toluene (2 mL) slowly at 20 °C. The mixture was stirred at 90 °C for 16 h. The mixture was filtered and concentrated under reduced pressure. The residue was purified by flash chromatography (ISCO®; 20 g AgelaFlash® Silica Flash Column, petroleum ether/EtOAc with EtOAc from 0 ~ 30 %, Flow Rate: 30 mL / min) to afford *tert*-butyl 6-[(1-benzylpyrazol-3-yl)methoxy]-3,4-dihydro-1*H*-isoquinoline-2-carboxylate (400 mg, crude) as a white solid.^{1}H NMR (400 MHz, MeOH-d_4) δ ppm 7.66 (d, *J* = 2.4 Hz, 1 H), 7.27 - 7.37 (m, 3 H), 7.18 - 7.24 (m, 2 H), 7.02 (d, *J* = 8.4 Hz, 1 H), 6.77 - 6.86 (m, 2 H), 6.40 (d, *J* = 2.3 Hz, 1 H), 5.34 (s, 2 H), 5.04 (s, 2 H), 3.59 (t, *J* = 5.3 Hz, 2 H), 2.78 (t, J = 5.4 Hz, 2H), 1.50 (s, 9 H); LCMS (ESI) $[M+H-56]^+$ m/z: calcd 364.2, found 364.1.

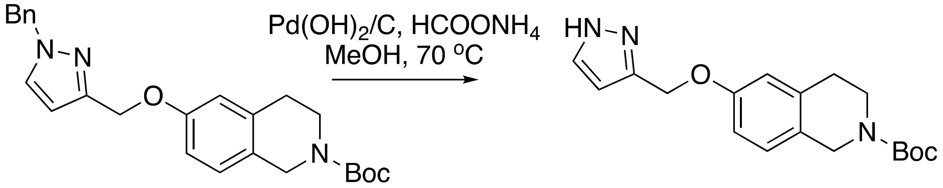

tert-butyl 6-((1*H*-pyrazol-3-yl)methoxy)-3,4-dihydroisoquinoline-2(1*H*)-carboxylate
To a solution of *tert*-butyl 6-[(1-benzylpyrazol-3-yl)methoxy]-3,4-dihydro-1*H*-isoquinoline-2-carboxylate (600 mg, 1.43 mmol) in MeOH (3 mL) was added $HCOONH_4$ (541.1 mg, 8.58 mmol) and $Pd(OH)_2/C$ (50 mg, 20% of $Pd(OH)_2$ with 50% of water; wt%). The mixture was stirred at 70 °C for 26. The mixture was filtered, followed by addition of $HCOONH_4$ (60 mg) and $Pd(OH)_2/C$ (50 mg, 20 % of $Pd(OH)_2$ of 50 % with water; wt %) and stirred at 70 °C for 32 h. The mixture was filtered and concentrated under reduced pressure to afford *tert*-butyl 6-(1*H*-pyrazol-3-ylmethoxy)-3,4-dihydro-1*H*-isoquinoline-2-carboxylate (365 mg, crude) as a yellow oil. LCMS (ESI) [M+H-100]+ m/z: calcd 230.2, found 230.1.

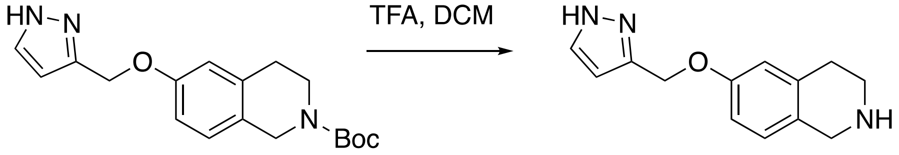

intermediate 33a

6-((1*H*-pyrazol-3-yl)methoxy)-1,2,3,4-tetrahydroisoquinoline, **intermediate 33a**
The solution of *tert*-butyl 6-(1*H*-pyrazol-3-ylmethoxy)-3,4-dihydro-1*H*-isoquinoline-2-carboxylate (365 mg, 1.11 mmol) and TFA (11.68 mmol, 0.9 mL) in DCM (10 mL) was stirred for 16 h at 20 °C. The mixture was filtered and concentrated under reduced pressure. The residue was quenched by addition of water (20 mL) and washed with DCM (10 mL x 2). The aqueous layer was adjusted to pH = 7~8 by saturated $NaHCO_3$ aqueous solution, extracted with DCM (20 mL x 3), and the combined organic layer was dried over anhydrous Na_2SO_4, filtered and concentrated under reduced pressure to give 6-(1*H*-pyrazol-3-ylmethoxy)-1,2,3,4-tetrahydroisoquinoline, **intermediate 33a** (180 mg, crude) as a yellow oil. LCMS (ESI) $[M+H]^+$ m/z: calcd 230.1, found 230.1.

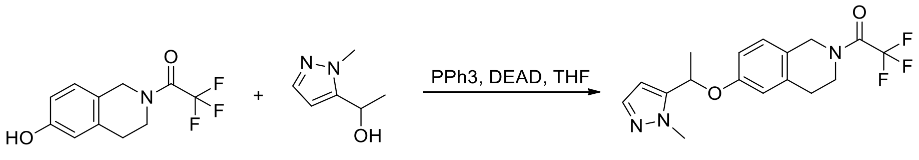

2,2,2-trifluoro-1-[6-[1-(2-methylpyrazol-3-yl)ethoxy]-3,4-dihydro-1*H*-isoquinolin-2-yl]ethenone
2,2,2-Trifluoro-1-(6-hydroxy-3,4-dihydro-1*H*-isoquinolin-2-yl)ethanone (3.3 g, 13.46 mmol), 1-(2-methylpyrazol-3-yl)ethanol (1.70 g, 13.46 mmol) and triphenylphosphine (4.24 g, 16.15 mmol) were mixed in THF (30 mL) and cooled with an ice-bath, then diisopropyl azodicarboxylate (3.27 g, 16.15 mmol, 3.17 mL) was added. The obtained mixture was stirred at 0 °C for 30 min then the ice-bath was removed and the reaction was stirred for 16 h at 20 °C. The obtained solution was evaporated in vacuo and the residue was subjected to flash-chromatography (SiO_2, hexane-MTBE) to give 2.1 g of crude product which was further purified by HPLC (C18 column, ACN-water) to yield 2,2,2-trifluoro-1-[6-[1-(2-methylpyrazol-3-yl)ethoxy]-3,4-dihydro-1*H*-isoquinolin-2-yl]ethanone (1.51 g, 4.27 mmol, 32 % yield) as yellowish viscous oil.^{1}H NMR (400 MHz, DMSO-d_6) δ (ppm) 1.57 (m, 3H), 2.81 (m, 2H), 3.78 (m, 5H), 4.66 (m, 2H), 5.72 (m, 1H), 6.28 (s, 1H), 6.87 (m, 2H), 7.16 (m, 1H), 7.32 (s, 1H). LCMS(ESI): $[M+H]^+$ m/z: calcd 353.1; found 354.0; Rt = 1.14 min.

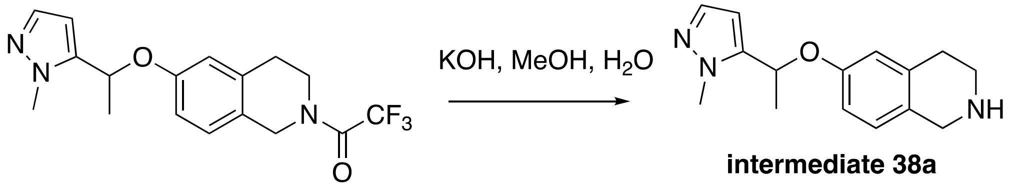

intermediate 38a

6-(1-(1-methyl-1*H*-pyrazol-5-yl)ethoxy)-1,2,3,4-tetrahydroisoquinoline, **intermediate 38a**
2,2,2-trifluoro-1-[6-[1-(2-methylpyrazol-3-yl)ethoxy]-3,4-dihydro-1*H*-isoquinolin-2-yl]ethanone (0.36 g, 1.02 mmol) was dissolved in MeOH (6 mL) then potassium hydroxide (68.60 mg, 1.22 mmol, 33.63 uL) dissolved in water (2 mL) was added and the obtained mixture was stirred at 70 °C for 2 h. The volatiles were evaporated in vacuo and the residue was partitioned between water (10 mL) and DCM (10 mL). The water layer was additionally extracted with DCM (10 mL x 3) and the combined organic fractions were washed with brine (20 mL), dried over Na_2SO_4 and evaporated in vacuo to give 6-[1-(2-methylpyrazol-3-yl)ethoxy]-1,2,3,4-tetrahydroisoquinoline, **intermediate 38a** (190 mg, 738.35 umol, 72 % yield) as a colorless viscous oil which was used as is.

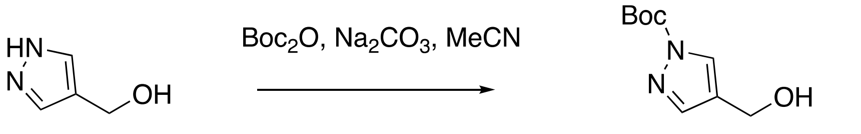

intermediate 41a'

tert-butyl 4-(hydroxymethyl)-1*H*-pyrazole-1-carboxylate, **intermedite 41a'**
To a solution of 1*H*-pyrazol-4-ylmethanol (950 mg, 9.68 mmol) in H_2O (5 mL) and MeCN (5 mL) was added Na_2CO_3 (1.14 g, 10.76 mmol), followed by *tert*-butoxycarbonyl *tert*-butyl carbonate (10.02 mmol, 2.3 mL). The mixture was stirred at 25 °C for 12 h. The resulting mixture was diluted with water (5 mL) and the aqueous solution was extracted with DCM (30 mL x 3). The organic layer was dried over anhydrous Na_2SO_4, filtered, and concentrated under reduced pressure to afford *tert*-butyl 4-(hydroxymethyl)pyrazole-1-carboxylate, **intermediate 41a'** (1.2 g, 56% yield) as a yellow oil.^{1}H NMR (400 MHz, MeOH-d_4) δ ppm 8.04 (s, 1 H), 7.70 (s, 1 H), 4.61 (s, 2 H), 1.63 - 1.68 (m, 9 H); LCMS (ESI) $[2M+Na]^+$ m/z: calcd 419.2, found 419.1.

Intermediates 16a, 17a, 19a-26a, 28a-30a, 32a, 33a, 39a-41a, 44a

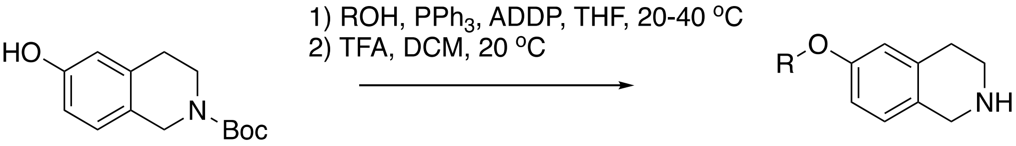

Intermediate General Procedure
To a mixture of *tert*-butyl 6-hydroxy-3,4-dihydro-1*H*-isoquinoline-2-carboxylate (1 eq), the appropriate alcohol (1.3 eq), and PPh_3 (1.5 eq) in THF (3 mL) was added a solution of (NE)-N-(piperidine-1-carbonylimino)piperidine-1-carboxamide (1.5 eq) in THF (1 mL) at 20 °C. After addition was complete, the mixture was stirred at 40 °C for 12 h. The resulting mixture was concentrated under reduced pressure and the residue was purified by flash chromatography. To this material was added TFA and the mixture was stirred at 25 °C for 2 h. The mixture was diluted with H_2O and the pH was adjusted to 9 with saturated K_2CO_3 aqueous solution. The mixture was extracted with DCM (3X) and the combined organic layer was dried over anhydrous Na_2SO_4, filtered, and concentrated under reduced pressure to give the desired amine.

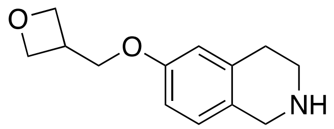

6-(oxetan-3-ylmethoxy)-1,2,3,4-tetrahydroisoquinoline, **intermediate 16a**
Alcohol: oxetan-3-ylmethanol. Colorless gum, 74% yield over 2 steps. ^{1}H NMR (400 MHz, MeOH-d_4) δ ppm 6.99 (d, J = 8.3 Hz, 1H), 6.71 - 6.80 (m, 2H), 4.82 - 4.87 (m, 2H), 4.59 (t, J = 6.0 Hz, 2H), 4.17 (d, J = 6.3 Hz, 2H), 3.98 (s, 2H), 3.38 - 3.49 (m, 1H), 3.11 - 3.18 (m, 2H), 2.87 (t, J = 6.0 Hz, 2 H).

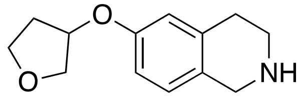

6-((tetrahydrofuran-3-yl)oxy)-1,2,3,4-tetrahydroisoquinoline, **intermediate 17a**
Alcohol: tetrahydrofuran-3-ol. Brown oil, 95% yield. ^{1}H NMR (400 MHz, $CDCl_3$) δ ppm 6.96 (d, J = 8.5 Hz, 1H), 6.71 (dd, J = 8.4, 2.6 Hz, 1H), 6.62 (s, 1H), 4.91 (t, J = 5.0 Hz, 1H), 4.06 (s, 2H), 3.88 - 4.03 (m, 4H), 3.23 (t, J = 6.1 Hz, 2H), 2.88 (t, J = 6.0 Hz, 2H), 2.12 - 2.24 (m, 2H); LCMS (ESI) $[M+H]^+$ m/z: calcd 220.3; found 220.1.

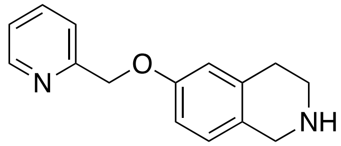

6-(pyridin-2-ylmethoxy)-1,2,3,4-tetrahydroisoquinoline, **intermediate 19a**
Alcohol: pyridin-2-ylmethanol. Yellow oil, 60% yield over two steps. LCMS (ESI) [M+H]+ calcd 241.1, found 241.1.

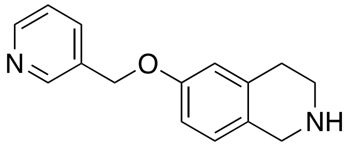

6-(pyridin-3-ylmethoxy)-1,2,3,4-tetrahydroisoquinoline, **intermediate 20a**
Alcohol: pyridin-3-ylmethanol. Colorless oil which was used without further purification, 68% yield over two steps. LCMS (ESI) [M+H]+ m/z: calcd 241.2; found 241.1.

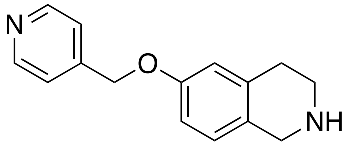

6-(pyridin-4-ylmethoxy)-1,2,3,4-tetrahydroisoquinoline, **intermediate 21a**

Alcohol: pyridin-4-ylmethanol. Light-yellow solid, 71% yield over 2 steps. ^{1}H NMR (400 MHz, MeOH-d_4) δ ppm 8.52 - 8.57 (m, 2H), 7.52 (d, J = 6.0 Hz, 2H), 7.12 (d, J = 8.5 Hz, 1H), 6.93 (dd, J = 8.4, 2.4 Hz, 1H), 6.89 (s, 1H), 5.20 (s, 2H), 4.18 (s, 2H), 3.35 - 3.39 (m, 2H), 3.02 (t, J = 6.1 Hz, 2H); LCMS (ESI) [M+H]+ m/z: calcd 241.1; found 241.1.

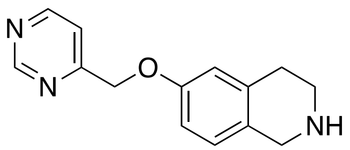

6-(pyrimidin-4-ylmethoxy)-1,2,3,4-tetrahydroisoquinoline, **intermediate 22a**
Alcohol: pyrimidin-4-ylmethanol. Yellow solid, 57% yield over 2 steps. ^{1}H NMR (400 MHz, MeOH-*d4*) δ ppm 9.13 (s, 1H), 8.78 (d, J = 5.3 Hz, 1H), 7.68 (d, J = 5.3 Hz, 1H), 7.07 (d, J = 8.4 Hz, 1H), 6.82 - 6.90 (m, 2H), 5.19 (s, 2H), 4.48 (s, 2H), 3.60 (t, J = 5.4 Hz, 2H), 2.80 (t, J = 5.9 Hz, 2H), 1.49 (s, 9H); LCMS (ESI) [M+H-56]+ m/z: calcd 286.2; found 286.1.

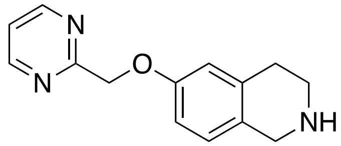

6-(pyrimidin-2-ylmethoxy)-1,2,3,4-tetrahydroisoquinoline, **intermediate 23a**
Alcohol: pyrimidin-2-ylmethanol. Colorless oil, 73% yield over 2 steps. ^{1}H NMR (400 MHz, MeOH-d_4) δ ppm 8.71 (d, J = 5.0 Hz, 2 H), 7.34 (t, J = 4.9 Hz, 1 H), 6.85 (d, J = 8.4 Hz, 1 H), 6.70 (d, J = 8.8 Hz, 1 H), 6.66 (s, 1 H), 5.13 (s, 2 H), 3.80 (s, 2 H), 2.96 (t, J = 6.0 Hz, 2 H), 2.70 (t, J = 5.9 Hz, 2 H); LCMS (ESI) [M+H]+ m/z: calcd 242.1; found 242.1.

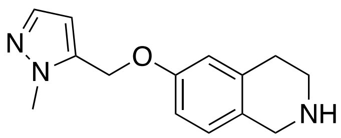

6-((1-methyl-1*H*-pyrazol-5-yl)methoxy)-1,2,3,4-tetrahydroisoquinoline, **61**
Alcohol: (1-methyl-1*H*-pyrazol-5-yl)methanol. Red oil which was used in next step directly, 87% yield over 2 steps. ^{1}H NMR (400 MHz, MeOH-d_4) δ ppm 7.40 (d, J = 1.9 Hz, 1 H), 6.98 (d, J = 8.4 Hz, 1 H), 6.77 - 6.86 (m, 2 H), 6.36 (d, J = 2.0 Hz, 1 H), 5.10 (s, 2 H), 3.93 (s, 2 H), 3.88 (s, 3 H), 3.35 (s, 2 H), 3.09 (t, J = 6.1 Hz, 2 H), 2.80 - 2.86 (m, 2 H); LCMS (ESI) [M+H]+ m/z calcd 244.1, found 244.1; HPLC: 84.2% @ 220nm.

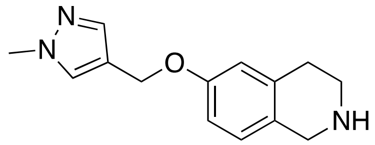

6-((1-methyl-1*H*-pyrazol-4-yl)methoxy)-1,2,3,4-tetrahydroisoquinoline, **intermediate 25a**
Alcohol: (1-methyl-1*H*-pyrazol-4-yl)methanol. Yellow oil 26% yield over 2 steps. LCMS (ESI) [M+H]+ m/z: calcd 244.1; found 244.1.

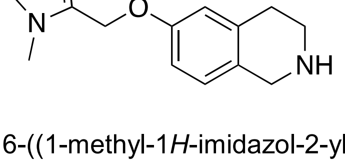

6-((1-methyl-1*H*-imidazol-2-yl)methoxy)-1,2,3,4-tetrahydroisoquinoline, **intermediate 26a**
Alcohol: (1-methyl-1*H*-imidazol-2-yl)methanol. Yellow solid, 54% over 2 steps. LCMS (ESI) $[M+H]^+$ m/z: calcd 244.1; found 244.1.

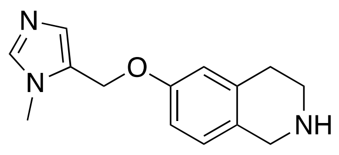

6-((1-methyl-1*H*-imidazol-5-yl)methoxy)-1,2,3,4-tetrahydroisoquinoline, **intermediate 28a**
Alcohol: (1-methyl-1*H*-imidazol-5-yl)methanol. Yellow oil which was directly used without further purification, 54% yield over 2 steps.

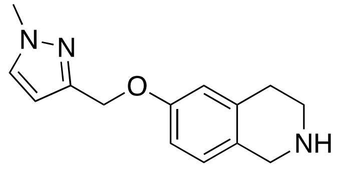

6-((1-methyl-1*H*-pyrazol-3-yl)methoxy)-1,2,3,4-tetrahydroisoquinoline, **intermediate 29a**
Alcohol: (1-methyl-1*H*-pyrazol-3-yl)methanol. Yellow solid, 69% yield over 2 steps. LCMS (ESI) [M+H]+ m/z: calcd 244.1; found 244.1.

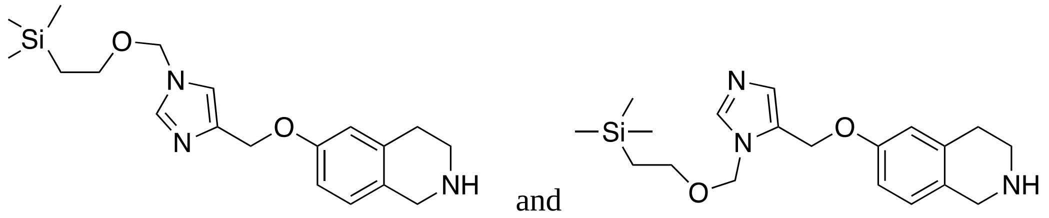

Mixture of 6-((1-((2-(trimethylsilyl)ethoxy)methyl)-1*H*-imidazol-4-yl)methoxy)-1,2,3,4-tetrahydroisoquinoline and 6-((1-((2-(trimethylsilyl)ethoxy)methyl)-1*H*-imidazol-5-yl)methoxy)-1,2,3,4-tetrahydroisoquinoline, intermediate 30a
Alcohol: intermediate 30a'. Brown oil which was directly used without further purification. LCMS (ESI) $[M+H]^+$ m/z: calcd 360.2, found 360.2.

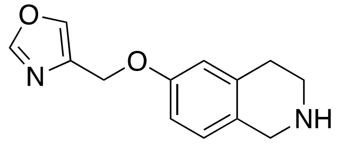

4-(((1,2,3,4-tetrahydroisoquinolin-6-yl)oxy)methyl)oxazole, **intermediate 32a**
Alcohol: oxazol-4-ylmethanol. Yellow oil, 86% yield. ^{1}H NMR (400 MHz, MeOH-d_4) δ ppm 8.23 (s, 1 H), 7.98 (s, 1 H), 6.95 (d, J = 8.4 Hz, 1 H), 6.72 - 6.80 (m, 2 H), 4.96 - 5.03 (m, 2 H), 3.89 (s, 2 H), 3.05 (t, J = 6.0 Hz, 2 H), 2.81 (t, J = 6.0 Hz, 2 H); LCMS (ESI) [M+H]+ m/z: calcd 231.1, found 231.1.

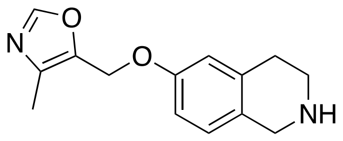

4-methyl-5-(((1,2,3,4-tetrahydroisoquinolin-6-yl)oxy)methyl)oxazole, **intermediate 39a**
Alcohol: (4-methyloxazol-5-yl)methanol. Colorless oil used as is. LCMS (ESI) $[M+H]^+$ m/z: calcd 245.1, found 245.1.

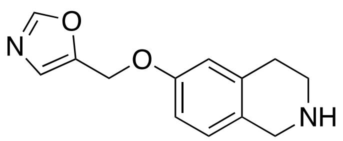

5-(((1,2,3,4-tetrahydroisoquinolin-6-yl)oxy)methyl)oxazole, **intermediate 40a**
Alcohol: oxazol-5-ylmethanol. Yellow oil, 96% yield. ^{1}H NMR (400 MHz, MeOH-d_4) δ ppm 8.13 (s, 1 H), 7.12 (s, 1 H), 6.87 (d, *J* = 8.3 Hz, 1 H), 6.64 - 6.71 (m, 2 H), 5.00 (s, 2 H), 3.79 (s, 2 H), 2.96 (t, *J* = 6.0 Hz, 2 H), 2.71 (t, *J* = 5.9 Hz, 2 H); LCMS (ESI) $[M+H]^+$ m/z: calcd 231.1; found 231.1.

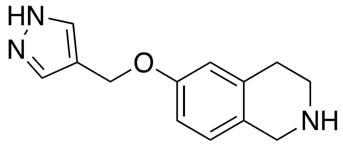

6-((1*H*-pyrazol-4-yl)methoxy)-1,2,3,4-tetrahydroisoquinoline, **intermediate 41a**
Alcohol: intermediate 41a'. Yellow oil, 44% yield over 2 steps. ^{1}H NMR (400 MHz, MeOH-d_4) δ ppm 7.66 (s, 2 H), 6.94 (d, *J* = 8.5 Hz, 1 H), 6.74 - 6.78 (m, 1 H), 6.72 (s, 1 H), 4.98 (s, 2 H), 3.88 (s, 2 H), 3.02 - 3.07 (m, 2 H), 2.80 (t, *J* = 6.0 Hz, 2 H); LCMS (ESI) $[M+H]^+$ m/z: calcd 230.1, found 230.1.

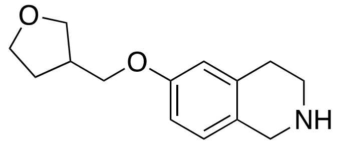

6-((tetrahydrofuran-3-yl)methoxy)-1,2,3,4-tetrahydroisoquinoline, **intermediate 44a**
Alcohol: (tetrahydrofuran-3-yl)methanol. Light yellow oil, 36% yield over 2 steps. ^{1}H NMR (400 MHz, MeOH-d_4) δ ppm 6.99 (d, *J* = 8.5 Hz, 1 H), 6.65 - 6.85 (m, 2 H), 3.98 (s, 2 H), 3.85 - 3.96 (m, 4 H), 3.75 - 3.82 (m, 1 H), 3.69 (dd, *J* = 8.8, 5.5 Hz, 1 H), 3.15 (t, *J* = 6.1 Hz, 2 H), 2.87 (t, *J* = 6.1 Hz, 2 H), 2.67 - 2.79 (m, 1 H), 2.08 - 2.24 (m, 1 H), 1.72 - 1.84 (m, 1 H); LCMS (ESI) $[M+H]^+$ m/z: calcd 234.1, found 234.1.

Intermediates 27a, 31a, 34a-37a, 42a, 43a.

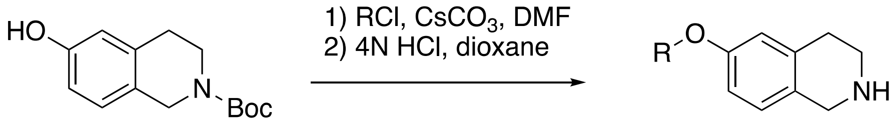

Intermediate General Procedure
To a solution of *tert*-butyl 6-hydroxy-3,4-dihydro-1*H*-isoquinoline-2-carboxylate (1 eq) in DMF (0.2 M) was added cesium carbonate (3.3 eq), followed by the appropriate chloride (3 eq). The reaction mixture was stirred at 100 °C for 24 h, then cooled to room temperature, poured into water, and the product was extracted with EtOAc (3x20 mL). The combined organic extracts were washed with brine (2 times), dried over sodium sulphate and evaporated in vacuo to afford desired Boc protected ether which was stirred in 4.0M hydrogen chloride solution in dioxane (0.2 M) and the resulting mixture was stirred at 25 °C for 2 h. The solid that formed was filtered, washed with EtOAc, and dried to give the HCl salt of the desired amine.

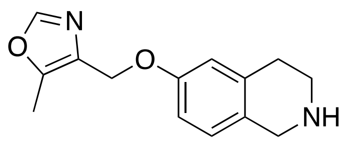

5-methyl-4-(((1,2,3,4-tetrahydroisoquinolin-6-yl)oxy)methyl)oxazole, **intermediate 27a**
Chloride: 4-(chloromethyl)-5-methyloxazole. White solid, 55% yield over 2 steps. ^{1}H NMR (400 MHz, DMSO-d_6) δ (ppm) 2.33 (s, 3H), 2.96 (m, 2H), 3.26 (m, 2H), 4.11 (m, 2H), 4.90 (s, 2H), 6.86 (m, 2H), 7.10 (d, 1H), 8.21 (s, 1H), 9.78 (s, 1H). LCMS(ESI): $[M+H]^+$ m/z: calcd 244.1; found 245.1; Rt = 0.75 min.

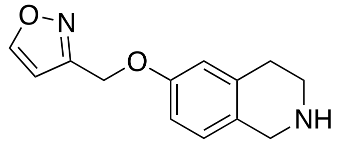

3-(((1,2,3,4-tetrahydroisoquinolin-6-yl)oxy)methyl)isoxazole, **intermediate 31a**
Chloride: 3-(chloromethyl)isoxazole. White solid, 71% yield over 2 steps. ^{1}H NMR (400 MHz, DMSO-d_6) δ (ppm) 2.95 (t, 2H), 3.27 (t, 2H), 4.13 (s, 2H), 5.20 (s, 2H), 6.65 (s, 1H), 6.91 (m, 2H), 7.13 (d, 1H), 8.93 (s, 1H), 9.62 (brs, 2H). LCMS(ESI): $[M+H]^+$ m/z: calcd 230.1; found 231.2; Rt = 0.78 min.

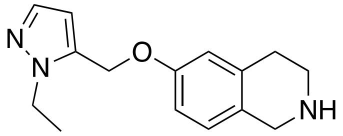

6-((1-ethyl-1*H*-pyrazol-5-yl)methoxy)-1,2,3,4-tetrahydroisoquinoline, **intermediate 34a**
Chloride: 5-(chloromethyl)-1-ethyl-1*H*-pyrazole. White solid, 67% yield over 2 steps. ^{1}H NMR (400 MHz, DMSO-d_6) δ (ppm) 1.32 (t, 3H), 2.97 (m, 2H), 3.29 (m, 2H), 4.13 (m, 4H), 5.15 (s, 2H), 6.36 (s, 1H), 6.91 (m, 2H), 7.13 (d, 1H), 7.40 (s, 1H), 9.63 (s, 2H). LCMS(ESI): $[M+H]^+$ m/z: calcd 257.1; found 258.1; Rt = 0.74 min.

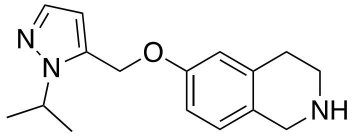

6-((1-isopropyl-1*H*-pyrazol-5-yl)methoxy)-1,2,3,4-tetrahydroisoquinoline, **intermediate 35a**
Chloride: 5-(chloromethyl)-1-isopropyl-1*H*-pyrazole. White solid, 65% yield over 2 steps. ^{1}H NMR (400 MHz, DMSO-d_6) δ (ppm) 1.38 (m, 6H), 2.97 (m, 2H), 3.29 (m, 2H), 4.14 (s, 2H), 4.57 (m, 1H), 5.15 (s, 2H), 6.33 (s, 1H), 6.90 (s, 2H), 7.12 (d, 1H), 7.41 (s, 1H), 9.59 (s, 2H). LCMS(ESI): $[M+H]^+$ m/z: calcd 271.1; found 272.2; Rt = 0.84 min.

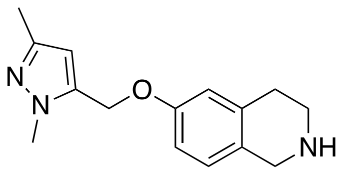

6-((1,3-dimethyl-1*H*-pyrazol-5-yl)methoxy)-1,2,3,4-tetrahydroisoquinoline, **intermediate 36a**
Chloride: 5-(chloromethyl)-1,3-dimethyl-1*H*-pyrazole. White solid, 82% yield over 2 steps. ^{1}H NMR (400 MHz, DMSO-d_6) δ (ppm) 2.12 (s, 3H), 2.97 (m, 2H), 3.28 (m, 2H), 3.74 (s, 3H), 4.13 (m, 2H), 2.10 (s, 2H), 6.17 (s, 1H), 6.91 (m, 2H), 7.13 (d, 1H), 9.68 (s, 2H). LCMS(ESI): $[M+H]^+$ m/z: calcd 257.2; found 258.2; Rt = 0.83 min.

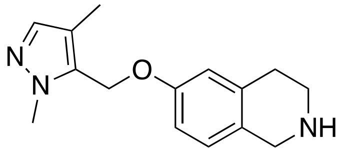

6-((1,4-dimethyl-1*H*-pyrazol-5-yl)methoxy)-1,2,3,4-tetrahydroisoquinoline, **intermediate 37a**
Chloride: 5-(chloromethyl)-1,4-dimethyl-1*H*-pyrazole. White solid, 64% yield over 2 steps. ^{1}H NMR (500 MHz, DMSO-d_6) δ (ppm) 2.01 (s, 3H), 2.98 (m, 2H), 3.29 (m, 2H), 3.78 (s, 3H), 4.14 (s, 2H), 5.08 (s, 2H), 6.91 (m, 2H), 7.14 (d, 1H), 7.26 (s, 1H), 8.47 (s, 2H), 9.74 (s, 2H). LCMS(ESI): $[M+H]^+$ m/z: calcd 257.1; found 258.2; Rt = 0.84 min.

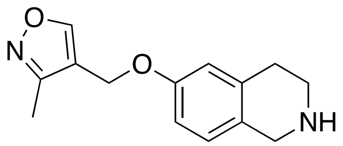

3-methyl-4-(((1,2,3,4-tetrahydroisoquinolin-6-yl)oxy)methyl)isoxazole, **intermediate 42a**
Chloride: 4-(chloromethyl)-3-methylisoxazole. White solid, 50% yield over 2 steps. ^{1}H NMR (500 MHz, DMSO-d_6) δ (ppm) 2.24 (s, 3H), 2.94 (m, 2H), 3.31 (m, 2H), 4.15 (m, 2H), 4.97 (s, 2H), 6.88 (m, 2H), 7.11 (d, 1H), 8.88 (m, 1H), 9.08 (s, 2H). LCMS(ESI): $[M+H]^+$ m/z: calcd 244.1; found 245.2; Rt = 0.81 min.

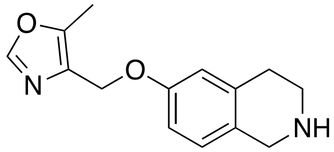

5-methyl-4-(((1,2,3,4-tetrahydroisoquinolin-6-yl)oxy)methyl)oxazole, **intermediate 43a**
Chloride: 4-(chloromethyl)-5-methyloxazole. White solid, 55% yield over 2 steps. ^{1}H NMR (400 MHz, DMSO-d_6) δ (ppm) 2.33 (s, 3H), 2.96 (m, 2H), 3.26 (m, 2H), 4.11 (m, 2H), 4.90 (s, 2H), 6.86 (m, 2H), 7.10 (d, 1H), 8.21 (s, 1H), 9.78 (s, 1H). LCMS(ESI): $[M+H]^+$ m/z: calcd 244.1; found 245.1; Rt = 0.75 min.

Compounds Intermediate 4a, 2, 3, 5, 10 - 44

General Procedure A. Substituted tetrahydroisoquinoline (1.03 eq), N-[(2S)-3-chloro-2-hydroxy-propyl]-2-(cyclobutylamino)pyridine-4-carboxamide (1 eq), triethylamine (3 eq), sodium iodide (q.5 eq) and acetonitrile (0.1 M) were added to a sealed tube. The mixture was stirred for 12 h at 100 °C. The resulting mixture was filtered, washed with MeOH (10 mL) and concentrated under reduced pressure. The residue was purified by preparative HPLC (Instrument: Gilson GX-281 Liquid Handler, Gilson 322 Pump, Gilson 156 UV Detector; Column: Waters Xbridge 150 × 25 mm × 5 µm; Mobile phase A: H_2O with 0.05% NH_3-H_2O (v%); Mobile phase B: MeCN; Gradient: B from 28% to 58% in 7.8 min, hold 100% B for 2.5 min; Flow Rate: 25 mL/min; Column Temperature: 30 ^oC; Wavelength: 220 nm, 254 nm) to afford the desired compound.

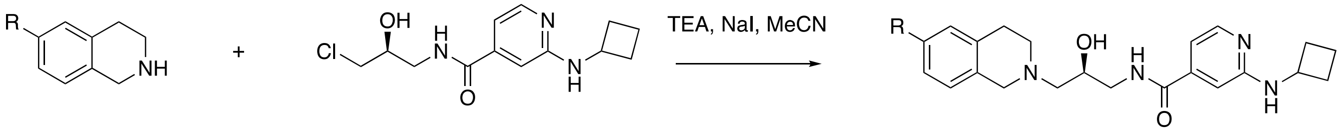

The following compounds were prepared by general procedure A.

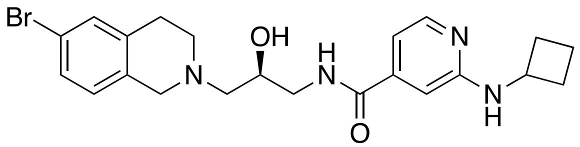

Intermediate 4a

(*S*)-*N*-(3-(6-bromo-3,4-dihydroisoquinolin-2(1*H*)-yl)-2-hydroxypropyl)-2-(cyclobutylamino)isonicotinamide, **intermediate 4a**.
Amine: 6-bromo-1,2,3,4-tetrahydroisoquinoline. Yellow solid, 51% yield. ^{1}H NMR (400 MHz, MeOH-d_4) δ ppm 7.94 (d, J = 5.4 Hz, 1H), 7.26 - 7.33 (m, 2H), 7.00 (d, J = 8.3 Hz, 1H), 6.81 (s, 1H), 6.77 (d, J = 5.5 Hz, 1H), 4.21 - 4.30 (m, 1H), 4.09 - 4.15 (m, 1H), 3.79 (s, 2H), 3.50 - 3.57 (m, 1H), 3.41 - 3.48 (m, 1 H), 2.94 (s, 4H), 2.69 - 2.79

(m, 2H), 2.39 - 2.47 (m, 2H), 1.89 - 2.00 (m, 2H), 1.74 - 1.85 (m, 2H); LCMS (ESI) $[M+H]^+$ m/z: calcd 459.1; found 459.1.

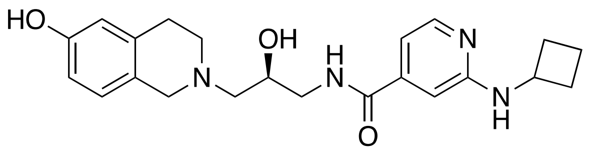

2

(*S*)-2-(cyclobutylamino)-*N*-(2-hydroxy-3-(6-hydroxy-3,4-dihydroisoquinolin-2(1*H*)-yl)propyl)isonicotinamide (**2**). Amine: 1,2,3,4-tetrahydroisoquinolin-6-ol. Yellow solid, 45% yield. ^{1}H NMR (400 MHz, MeOH-d_4) δ ppm 7.89 (d, J = 5.3 Hz, 1 H), 6.85 (d, J = 8.3 Hz, 1 H), 6.77 (s, 1 H), 6.72 (dd, J = 5.5, 1.3 Hz, 1 H), 6.57 (dd, J = 8.3, 2.5 Hz, 1 H), 6.53 (d, J = 2.0 Hz, 1 H), 4.15 - 4.27 (m, 1 H), 4.00-4.14 (m, 1 H), 3.63 (s, 2 H), 3.40 - 3.52 (m, 2 H), 2.81 (br d, J = 4.0 Hz, 4 H), 2.54 - 2.70 (m, 2 H), 2.32 - 2.45 (m, 2 H), 1.84 - 1.98 (m, 2 H), 1.67 - 1.81 (m, 2 H); LCMS (ESI) $[M+H]^+$ calcd 397.2, found 397.1; HPLC, 100% @254nm; 100%ee.

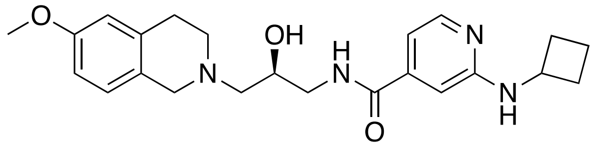

3

(*S*)-2-(cyclobutylamino)-*N*-(2-hydroxy-3-(6-methoxy-3,4-dihydroisoquinolin-2(1*H*)-yl)propyl)isonicotinamide (**3**). Amine: 6-methoxy-1,2,3,4-tetrahydroisoquinoline. White solid, 22% yield. ^{1}H NMR (400 MHz, MeOH-d_4) δ ppm 7.88 (d, J = 5.5 Hz, 1H), 6.95 (d, J = 8.5 Hz, 1H), 6.78 (s, 1H), 6.65 - 6.74 (m, 3H), 4.17 - 4.27 (m, 1H), 4.04 - 4.12 (m, 1H), 3.75 (s, 3H), 3.67 (s, 2H), 3.41 - 3.53 (m, 2H), 2.80 - 2.91 (m, 4H), 2.60 - 2.71 (m, 2H), 2.35 - 2.44 (m, 2H), 1.86 - 1.97 (m, 2H), 1.73 - 1.82 (m, 2H); LCMS (ESI) $[M+H]^+$ m/z: calcd 411.2; found 411.2; HPLC: 98.22% @254 nm; 99.6%ee.

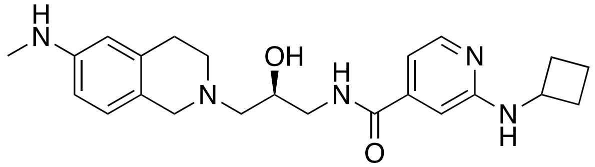

5

(*S*)-2-(cyclobutylamino)-*N*-(2-hydroxy-3-(6-(methylamino)-3,4-dihydroisoquinolin-2(1*H*)-yl)propyl)isonicotinamide (**5**).

Amine: **Intermediate 5a**. Yellow solid, 14% yield. ^{1}H NMR(DMSO-d_6, 500 MHz): δ 1.67 (m, 2H), 1.85 (m, 2H), 2.24 (m, 2H), 2.44 (m, 2H), 2.65 (m, 7H), 3.21 (m, 1H), 3.38 (m, 1H), 3.46 (m, 2H), 3.87 (m, 1H), 4.26 (m, 1H), 4.80 (d, 1H), 5.35 (d, 1H), 6.23 (s, 1H), 6.34 (d, 1H), 6.73 (m, 3H), 6.91 (d, 1H), 7.93 (d, 1H), 8.51 (t, 1H). LCMS(ESI): $[M+H]^+$ m/z: calcd 409.2; found 410.2; Rt = 0.64 min.

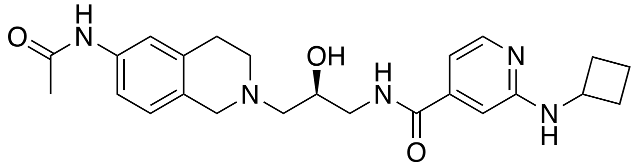

10

(*S*)-N-(3-(6-acetamido-3,4-dihydroisoquinolin-2(1*H*)-yl)-2-hydroxypropyl)-2-(cyclobutylamino)isonicotinamide (**10**).

Amine: **Intermediate 10a**. Yellow solid, 14% yield. ^{1}H NMR (400 MHz, DMSO-d_6) δ 1.67 (m, 2H), 1.88 (m, 2H), 2.03 (s, 3H), 2.29 (m, 2H), 2.47 (m, 1H), 2.72 (m, 2H), 2.78 (m, 2H), 3.23 (m, 1H), 3.41 (m, 2H), 3.58 (m, 2H), 3.91 (m, 1H), 4.28 (m, 1H), 4.85 (d, 1H), 6.76 (m, 2H), 6.95 (t, 2H), 7.29 (m, 1H), 7.38 (s, 1H), 7.97 (d, 1H), 8.50 (t, 1H), 9.81 (s, 1H). LCMS(ESI): [M+H]+ m/z: calcd 437.2; found 438.2; Rt = 0.68 min.

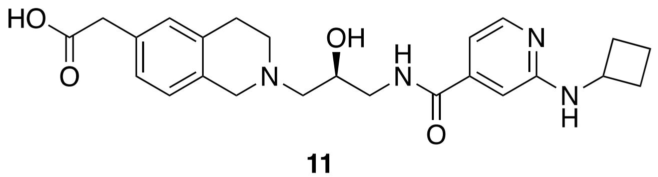

11

(*S*)-2-(2-(3-(2-(cyclobutylamino)isonicotinamido)-2-hydroxypropyl)-1,2,3,4-tetrahydroisoquinolin-6-yl)acetic acid (**11**).
Amine: **Intermediate 11a**, followed by the following procedure: To a solution of methyl 2-[2-[(2S)-3-[[2-(cyclobutylamino)pyridine-4-carbonyl]amino]-2-hydroxy-propyl]-3,4-dihydro-1*H*-isoquinolin-6-yl]acetate (50 mg, 0.11 mmol) in MeOH (2.5 mL) was added LiOH-H_2O (14 mg, 0.33 mmol) in H_2O (2.5 mL). Then the mixture was stirred at 20 °C for 12 h. The mixture was concentrated under reduced pressure and diluted with H_2O (10 mL), adjusted to pH = 6~7 with 2 M HCl aqueous solution. The resulting mixture was filtered and the filtrate was purified by preparative HPLC purification (Instrument: Gilson GX-281 Liquid Handler, Gilson 322 Pump, Gilson 156 UV Detector; Column: Durashell 150 × 25 mm × 5 μm; Mobile phase A: H2O with 0.05% NH_3-H_2O (v%); Mobile phase B: MeCN; Gradient: B from 10% to 40% in 6.5 min, hold 100% B for 2.5 min; Flow Rate: 25 mL/min; Column Temperature: 30 °C; Wavelength: 220 nm, 254 nm) to afford 2-[2-[(2S)-3-[[2-(cyclobutylamino)pyridine-4-carbonyl]amino]-2-hydroxy-propyl]-3,4-dihydro-1*H*-isoquinolin-6-yl]acetic acid **11** (20 mg, 41%) as a white solid. ^{1}H NMR (400 MHz, MeOH-d_4) δ ppm 7.98 (d, *J* = 5.3 Hz, 1H), 7.12 - 7.17 (m, 2H), 7.05 (d, *J* = 7.5 Hz, 1H), 6.80 - 6.85 (m, 2H), 4.19 - 4.31 (m, 2H), 4.13 (s, 2H), 3.43 - 3.56 (m, 4H), 3.28 (t, *J* = 6.5 Hz, 2H), 2.96 - 3.09 (m, 4H), 2.39 - 2.48 (m, 2H), 1.90 - 2.00 (m, 2H), 1.76 - 1.84 (m, 2H); LCMS (ESI) $[M+H]^+$ m/z: calcd 439.2; found 439.1; HPLC, 100%@254nm; 99.0%ee.

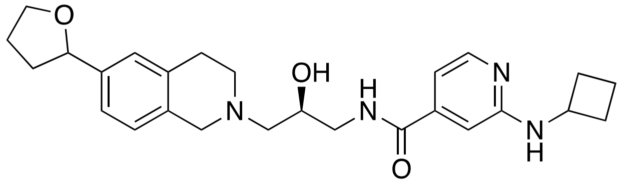

12

2-(cyclobutylamino)-*N*-((2*S*)-2-hydroxy-3-(6-(tetrahydrofuran-2-yl)-3,4-dihydroisoquinolin-2(1*H*)-yl)propyl)isonicotinamide (**12**).
Amine: **intermediate 12a**. Yellow solid, 44% yield. ^{1}H NMR (400 MHz, MeOH-d_4) δ ppm 7.91 (d, *J* = 5.5 Hz, 1 H), 7.10 (s, 2 H), 7.01 - 7.05 (m, 1 H), 6.82 (s, 1 H), 6.75 (d, *J* = 5.4 Hz, 1 H), 4.23 - 4.30 (m, 1 H), 4.06 - 4.14 (m, 2 H), 3.88 - 3.96 (m, 1 H), 3.75 (br s, 2 H), 3.41 - 3.48 (m, 2 H), 2.90 (d, *J* = 13.5 Hz, 4 H), 2.68 (br s, 2 H), 2.29 - 2.47 (m, 3 H), 2.01 - 2.09 (m, 2 H), 1.93 (br d, *J* = 11.0 Hz, 2 H), 1.72 - 1.84 (m, 3 H); LCMS (ESI) $[M+H]^+$ m/z calcd 451.3, found 451.3; HPLC 100%@254 nm.

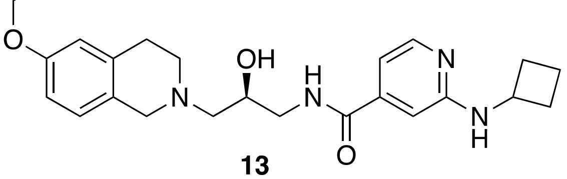

13

(*S*)-2-(cyclobutylamino)-*N*-(3-(6-ethoxy-3,4-dihydroisoquinolin-2(1*H*)-yl)-2-hydroxypropyl)isonicotinamide (**13**).

Amine: 6-ethoxy-1,2,3,4-tetrahydroisoquinoline. Yellow solid, 18% yield. ^{1}H NMR (400 MHz, MeOH-d_4) δ ppm 7.90 (d, J = 5.3 Hz, 1H), 6.95 (d, J = 8.3 Hz, 1H), 6.79 (s, 1H), 6.65 - 6.75 (m, 3H), 4.19 - 4.29 (m, 1H), 4.06 - 4.13 (m, 1H), 4.01 (q, J = 7.0 Hz, 2H), 3.69 (s, 2H), 3.43 - 3.54 (m, 2H), 2.81 - 2.92 (m, 4H), 2.62 - 2.72 (m, 2H), 2.38 - 2.46 (m, 2H), 1.88 - 1.99 (m, 2H), 1.74 - 1.84 (m, 2H), 1.38 (t, J = 7.0 Hz, 3 H); LCMS (ESI) $[M+H]^+$ m/z: calcd 425.2; found 425.2; HPLC: 99.76% @ 254 nm; 100%ee.

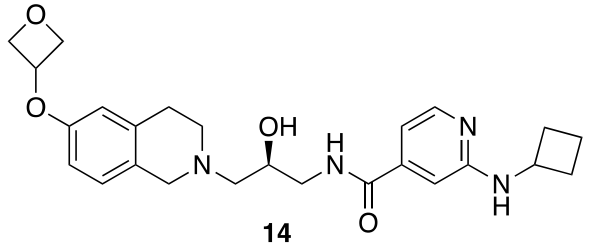

14

(*S*)-2-(cyclobutylamino)-*N*-(2-hydroxy-3-(6-(oxetan-3-yloxy)-3,4-dihydroisoquinolin-2(1*H*)-yl)propyl)isonicotinamide (**14**).
Amine: 6-(oxetan-3-yloxy)-1,2,3,4-tetrahydroisoquinoline. Yellow solid, 24% yield. ^{1}H NMR (400 MHz, MeOH-d_4) δ ppm 7.88 (d, J = 5.5 Hz, 1H), 6.96 (d, J = 8.5 Hz, 1H), 6.78 (s, 1H), 6.72 (dd, J = 5.3, 1.5 Hz, 1H), 6.56 (dd, J = 8.3, 2.5 Hz, 1H), 6.48 (d, J = 2.3 Hz, 1H), 5.19 - 5.26 (m, 1H), 4.99 (t, J = 6.7 Hz, 2H), 4.66 (dd, J = 7.0, 5.0 Hz, 2H), 4.18 - 4.27 (m, 1H), 4.04 - 4.11 (m, 1H), 3.66 (s, 2H), 3.40 - 3.53 (m, 2H), 2.79 - 2.89 (m, 4H), 2.59 - 2.69 (m, 2H), 2.36 - 2.44 (m, 2H), 1.87 - 1.97 (m, 2H), 1.72 - 1.82 (m, 2H); LCMS (ESI) $[M+H]^+$ m/z: calcd 453.2; found 453.2; HPLC: 97.17% @254nm; 99.5%ee.

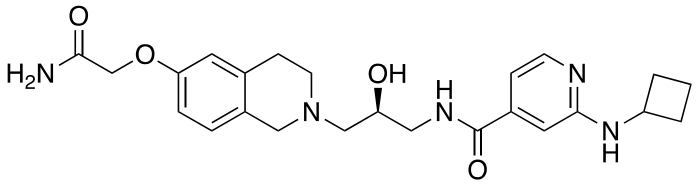

15

(*S*)-*N*-(3-(6-(2-amino-2-oxoethoxy)-3,4-dihydroisoquinolin-2(1*H*)-yl)-2-hydroxypropyl)-2-(cyclobutylamino)isonicotinamide (**15**).
Amine: **intermediate 15a**. Yellow solid, 14% yield. ^{1}H NMR (400 MHz, DMSO-d_6) δ (ppm) 1.66 (m, 2H), 1.85 (m, 2H), 2.26 (m, 2H), 2.69 (m, 3H), 2.76 (m, 2H), 3.20 (m, 1H), 3.39 (m, 2H), 3.54 (m, 2H), 3.88 (m, 1H), 4.25 (m, 1H), 4.35 (s, 2H), 4.81 (m, 1H), 6.67 (m, 1H), 6.75 (m, 3H), 6.94 (m, 2H), 7.35 (s, 1H), 7.45 (s, 1H), 7.95 (d, 1H), 8.46 (t, 1H). LCMS(ESI): $[M+H]^+$ m/z: calcd 453.2; found 454.2; Rt = 0.71 min.

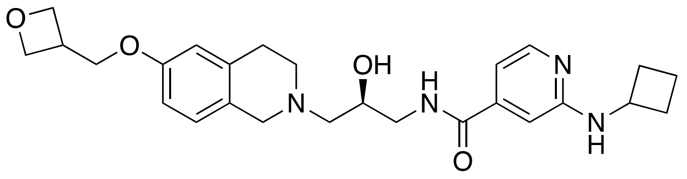

16

(*S*)-2-(cyclobutylamino)-*N*-(2-hydroxy-3-(6-(oxetan-3-ylmethoxy)-3,4-dihydroisoquinolin-2(1*H*)-yl)propyl)isonicotinamide (**16**).
Amine: **intermediate 16a**. Yellow solid, 39% yield. ^{1}H NMR (400 MHz, MeOH-d_4) δ ppm 7.89 (d, J = 5.5 Hz, 1H), 6.96 (d, J = 8.3 Hz, 1H), 6.78 (s, 1H), 6.70 - 6.76 (m, 3H), 4.84 - 4.87 (m, 2H), 4.59 (t, J = 6.0 Hz, 2H), 4.19 - 4.27 (m, 1H), 4.16 (d, J = 6.5 Hz, 2H), 4.04 - 4.11 (m, 1H), 3.68 (s, 2H), 3.39 - 3.53 (m, 3H), 2.79 - 2.92 (m, 4H), 2.60 - 2.70 (m, 2H), 2.36 - 2.44 (m, 2H), 1.87 - 1.97 (m, 2H), 1.72 - 1.82 (m, 2H); LCMS (ESI) $[M+H]^+$ m/z: calcd 467.2; found 467.3; HPLC: 98.87% @254nm; 99.4%ee.

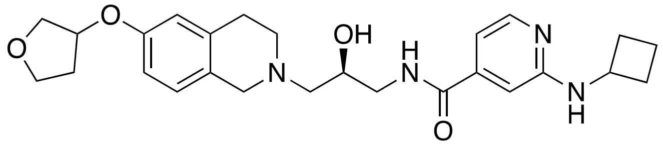

17

2-(cyclobutylamino)-*N*-((2*S*)-2-hydroxy-3-(6-((tetrahydrofuran-3-yl)oxy)-3,4-dihydroisoquinolin-2(1*H*)-yl)propyl)isonicotinamide (**17**).
Amine: **intermediate 17a**. Yellow solid, 65% yield. ^{1}H NMR (400 MHz, MeOH-d_4) δ ppm 7.89 (d, *J* = 5.4 Hz, 1H), 6.96 (d, *J* = 8.4 Hz, 1H), 6.79 (s, 1H), 6.73 (dd, *J* = 5.4, 1.4 Hz, 1H), 6.66 - 6.70 (m, 1H), 6.65 (d, *J* = 2.3 Hz, 1H), 4.17 - 4.30 (m, 1H), 4.04 - 4.13 (m, 1H), 3.81 - 3.99 (m, 4H), 3.67 (s, 2H), 3.39 - 3.55 (m, 2H), 2.72 - 2.93 (m, 4H), 2.56 - 2.70 (m, 2H), 2.33 - 2.47 (m, 2H), 2.15 - 2.27 (m, 1H), 1.99 - 2.12 (m, 1H), 1.85 - 1.98 (m, 2H), 1.72 - 1.82 (m, 2H); LCMS (ESI) [M+H]$^+$ m/z: calcd 467.3; found 467.2; HPLC: 100%@220nm; racemic.

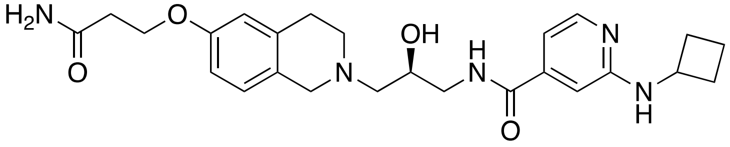

18

(*S*)-*N*-(3-(6-(3-amino-3-oxopropoxy)-3,4-dihydroisoquinolin-2(1*H*)-yl)-2-hydroxypropyl)-2-(cyclobutylamino)isonicotinamide (**18**).
Amine: **intermediate 18a**. White solid, 14% yield. ^{1}H NMR(DMSO-d_6, 500 MHz): δ (ppm) 1.70 (m, 2H), 1.90 (m, 2H), 2.32 (m, 2H), 2.59 (m, 4H), 2.84 (m, 4H), 3.23 (m, 4H), 3.41 (m, 1H), 4.12 (m, 3H), 4.32 (m, 2H), 6.68 (m, 5H), 6.97 (s, 1H), 7.32 (d, 1H), 7.92 (d, 1H), 8.42 (s, 1H).
LCMS(ESI): [M+H]+ m/z: calcd 468.2; found 468.2; Rt = 0.67 min.

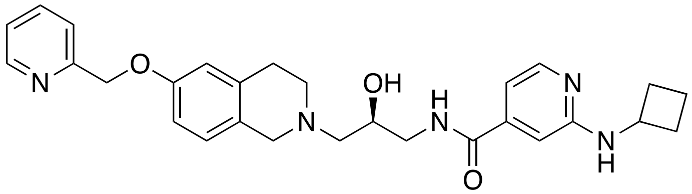

19

(*S*)-2-(cyclobutylamino)-*N*-(2-hydroxy-3-(6-(pyridin-2-ylmethoxy)-3,4-dihydroisoquinolin-2(1*H*)-yl)propyl)isonicotinamide (**19**).
Amine: **intermediate 19a**. Yellow solid, 36% yield. ^{1}H NMR (400 MHz, MeOH-d_4) δ ppm 8.53 (d, *J* = 4.6 Hz, 1 H), 7.89 (d, *J* = 5.3 Hz, 1 H), 7.83 - 7.87 (m, 1 H), 7.60 (d, *J* = 7.9 Hz, 1 H), 7.36 (dd, *J* = 7.1, 5.3 Hz, 1 H), 6.97 (d, *J* = 8.4 Hz, 1 H), 6.75 - 6.82 (m, 3 H), 6.73 (dd, *J* = 5.4, 1.3 Hz, 1 H), 5.14 (s, 2 H), 4.22 (quin, *J* = 7.8 Hz, 1 H), 4.07 (quin, *J* = 6.0 Hz, 1 H), 3.67 (s, 2 H), 3.38 - 3.56 (m, 2 H), 2.85 (dd, *J* = 15.4, 4.7 Hz, 4 H), 2.58 - 2.71 (m, 2 H), 2.34 - 2.46 (m, 2 H), 1.86 - 1.99 (m, 2 H), 1.71 - 1.82 (m, 2 H); LCMS (ESI) [M+H]$^+$ calcd 488.3, found 488.3; HPLC, 100%@254nm; 99.5%ee.

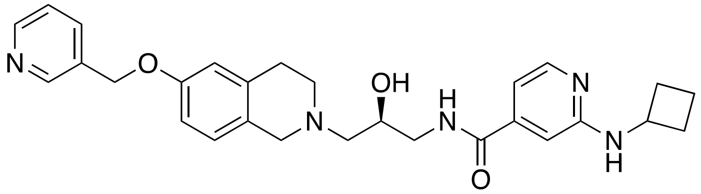

20

(*S*)-2-(cyclobutylamino)-*N*-(2-hydroxy-3-(6-(pyridin-3-ylmethoxy)-3,4-dihydroisoquinolin-2(1*H*)-yl)propyl)isonicotinamide (**20**).
Amine: **intermediate 20a**. Yellow solid, 15% yield. ^{1}H NMR (400 MHz, MeOH-d_4) δ ppm 8.64 (s, 1H), 8.51 (dd, *J* = 4.8, 1.5 Hz, 1H), 7.96 (d, *J* = 8.0 Hz, 1H), 7.91 (d, *J* = 5.5 Hz, 1H), 7.48 (dd, *J* = 7.8, 5.0 Hz, 1H), 6.99 (d, *J* = 8.3 Hz, 1H), 6.79 - 6.85 (m, 3H), 6.75 (dd, *J* = 5.5, 1.5 Hz, 1H), 5.14 (s, 2H), 4.19 - 4.29 (m, 1H), 4.06 - 4.14 (m, 1H), 3.70 (s, 2H), 3.42 - 3.56 (m, 2H), 2.81 - 2.94 (m, 4H), 2.61 - 2.72 (m, 2H), 2.37 - 2.46 (m, 2H), 1.88 - 1.99 (m, 2H), 1.73 - 1.84 (m, 2H); LCMS $[M+H^{+}]$ m/z: calcd 488.3; found 488.2; HPLC: 98.77% @254nm; 100%ee.

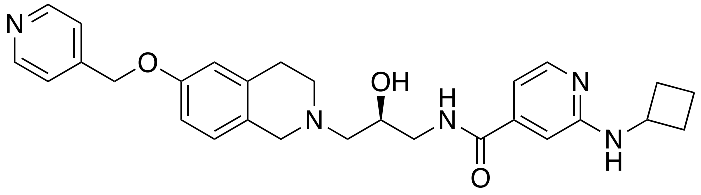

21

(*S*)-2-(cyclobutylamino)-*N*-(2-hydroxy-3-(6-(pyridin-4-ylmethoxy)-3,4-dihydroisoquinolin-2(1*H*)-yl)propyl)isonicotinamide (**21**).
Amine: **intermediate 21a**. Yellow solid, 30% yield. ^{1}H NMR (400 MHz, MeOH-d_4) δ ppm 8.54 (d, *J* = 6.0 Hz, 2H), 7.90 (d, *J* = 5.5 Hz, 1H), 7.53 (d, *J* = 6.0 Hz, 2H), 7.00 (d, *J* = 8.3 Hz, 1H), 6.77 - 6.84 (m, 3H), 6.74 (d, *J* = 5.5, 1.5 Hz, 1H), 5.18 (s, 2H), 4.20 - 4.28 (m, 1H), 4.07 - 4.13 (m, 1H), 3.70 (s, 2H), 3.43 - 3.54 (m, 2H), 2.9 - 2.80 (m, 4H), 2.62 - 2.71 (m, 2H), 2.38 - 2.46 (m, 2H), 1.88 - 1.99 (m, 2H), 1.73 - 1.84 (m, 2H); LCMS (ESI) $[M+H]^{+}$m/z: calcd 488.3; found 488.2; HPLC, 100%@254 nm;100%ee.

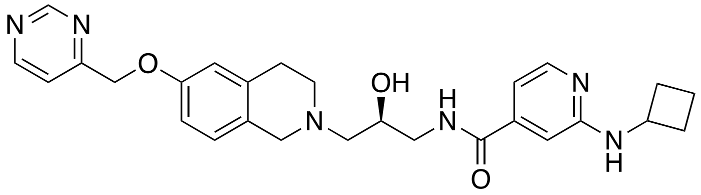

22

(*S*)-2-(cyclobutylamino)-*N*-(2-hydroxy-3-(6-(pyrimidin-4-ylmethoxy)-3,4-dihydroisoquinolin-2(1*H*)-yl)propyl)isonicotinamide (**22**).
Amine: **intermediate 22a**. Yellow solid, 18% yield. ^{1}H NMR (400 MHz, MeOH-d_4) δ ppm 9.13 (d, *J* = 1.3 Hz, 1H), 8.79 (d, *J* = 5.3 Hz, 1H), 7.89 (d, *J* = 5.3 Hz, 1H), 7.69 (d, *J* = 5.3 Hz, 1H), 6.99 (d, *J* = 8.3 Hz, 1H), 6.77 - 6.83 (m, 3H), 6.73 (dd, *J* = 5.3, 1.5 Hz, 1H), 5.18 (s, 2H), 4.18 - 4.27 (m, 1H), 4.04 - 4.11 (m, 1H), 3.68 (s, 2H), 3.40 - 3.53 (m, 2H), 2.80 - 2.91 (m, 4H), 2.60 - 2.69 (m, 2H), 2.36 - 2.44 (m, 2H), 1.87 - 1.97 (m, 2H), 1.71 - 1.82 (m, 2H); LCMS (ESI) $[M+H]^{+}$ m/z: calcd 489.3; found 489.2; HPLC: 100% @254nm; 99.2%ee.

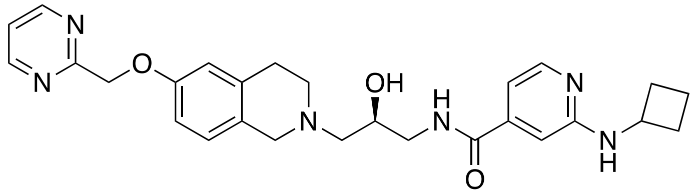

23

(*S*)-2-(cyclobutylamino)-*N*-(2-hydroxy-3-(6-(pyrimidin-2-ylmethoxy)-3,4-dihydroisoquinolin-2(1*H*)-yl)propyl)isonicotinamide (**23**).
Amine: **intermediate 23a**. Yellow solid, 29% yield. ^{1}H NMR (400 MHz, MeOH-d_4) δ ppm 8.84 (d, *J* = 4.9 Hz, 2 H), 7.91 (d, *J* = 5.4 Hz, 1 H), 7.47 (t, *J* = 4.9 Hz, 1 H), 6.97 (d, *J* = 8.4 Hz, 1 H), 6.77 - 6.84 (m, 3 H), 6.74 (dd, *J* = 5.4, 1.3 Hz, 1 H), 5.26 (s, 2 H), 4.20 - 4.29 (m, 1 H), 4.09 (t, *J* = 5.9 Hz, 1 H), 3.69 (s, 2 H), 3.43 - 3.53 (m, 2 H), 2.82 - 2.92 (m, 4 H), 2.60 - 2.72 (m, 2 H), 2.38 - 2.48 (m, 2 H), 1.89 - 2.00 (m, 2 H), 1.74 - 1.84 (m, 2 H); LCMS (ESI) $[M+H]^{+}$ m/z: calcd 489.2; found 489.2, HPLC 98.76%@ 220 nm; 100%ee.

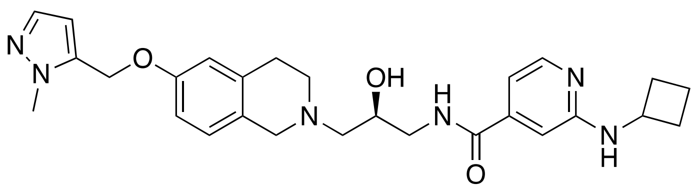

24

(*S*)-2-(cyclobutylamino)-*N*-(2-hydroxy-3-(6-((1-methyl-1*H*-pyrazol-5-yl)methoxy)-3,4-dihydroisoquinolin-2(1*H*)-yl)propyl)isonicotinamide (**24**).
Amine: **61**. Off-white solid, 32% yield. ^{1}H NMR (400 MHz, MeOH-*d$_4$*) *δ* ppm 7.90 (d, J = 5.3 Hz, 1 H), 7.41 (d, J = 1.8 Hz, 1 H), 6.98 (d, J = 8.0 Hz, 1 H), 6.77 - 6.84 (m, 3 H), 6.70 - 6.76 (m, 1 H), 6.37 (d, J = 2.0 Hz, 1 H), 5.11 (s, 2 H), 4.16 - 4.33 (m, 1 H), 4.01 - 4.14 (m, 1 H), 3.89 (s, 3 H), 3.68 (s, 2 H), 3.39 - 3.55 (m, 2 H), 2.77 - 2.94 (m, 4 H), 2.57 - 2.71 (m, 2 H), 2.34 - 2.45 (m, 2 H), 1.84 - 1.99 (m, 2 H), 1.70 - 1.83 (m, 2 H). ^{1}H NMR (DMSO *d$_6$*, 500 MHz): δ (ppm) 1.64 (m, 2H), 1.83 (m, 2H), 2.25 (m, 2H), 2.44 (m, 2H), 2.68 (m, 2H), 2.76 (m, 2H), 3.18 (m, 1H), 3.37 (m, 1H), 3.54 (m, 2H), 3.80 (s, 3H), 3.87 (m, 1H), 4.24 (h, 1H), 4.80 (d, 1H), 5.09 (s, 2H), 6.33 (d, 1H), 6.73 (m, 2H), 6.78 (m, 2H), 6.91 (d, 1H), 6.94 (d, 1H), 7.34 (d, 1H), 7.94 (d, 1H), 8.45 (t, 1H). ^{13}C NMR (DMSO-*d$_6$*, 126 MHz): δ (ppm) 14.69, 28.87, 30.56, 36.40, 44.74, 46.06, 51.00, 55.49, 59.99, 62.33, 66.66, 105.69, 106.86, 108.72, 112.77, 114.15, 127.29, 127.66, 135.31, 137.30, 137.54, 142.61, 148.06, 156.03, 158.37, 165.56. LCMS (ESI) [M+H]+ m/z calcd 491.3, found 491.2; HPLC: 100% @ 254nm; 100%ee. $[\alpha]^{21}_{D}$ = -4.40° (*c* = 0.25 g/100 mL, MeOH). Elemental analysis [calculated/found] C[66.10/64.97] H[6.99/6.56] N[17.13/17.90] O[9.78]. HRMS (ESI, + vw ion) m/z calcd for $C_{27}H_{34}N_6O_3$ [M+H$^+$] 490.26924, found 490.2685.

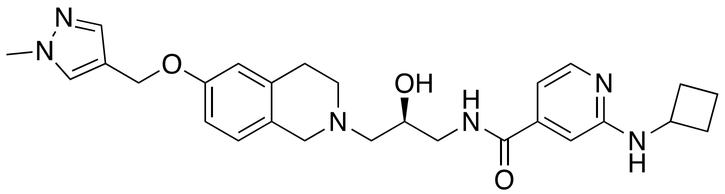

25

(*S*)-2-(cyclobutylamino)-*N*-(2-hydroxy-3-(6-((1-methyl-1*H*-pyrazol-4-yl)methoxy)-3,4-dihydroisoquinolin-2(1*H*)-yl)propyl)isonicotinamide (**25**).
Amine: **intermediate 25a**. White solid, 23% yield. ^{1}H NMR (400 MHz, MeOH-*d$_4$*) *δ* ppm 7.90 (d, *J* = 5.8 Hz, 1 H), 7.68 (s, 1 H), 7.53 (s, 1 H), 6.97 (d, *J* = 8.5 Hz, 1 H), 6.72 - 6.81 (m, 4 H), 4.96 (s, 2H), 4.24 (t, *J* = 7.7 Hz, 1 H), 4.05 - 4.14 (m, 1 H), 3.90 (s, 3 H), 3.69 (s, 2 H), 3.45 - 3.55 (m, 2 H), 2.80 - 2.95 (m, 4 H), 2.67 (t, *J* = 5.9 Hz, 2 H), 2.38 - 2.46 (m, 2 H), 1.89 - 1.99 (m, 2 H), 1.72 - 1.89 (m, 2 H); LCMS (ESI) [M+H]$^+$ m/z: calcd 491.3; found 491.2; HPLC, 100%@254nm; 99.1%ee.

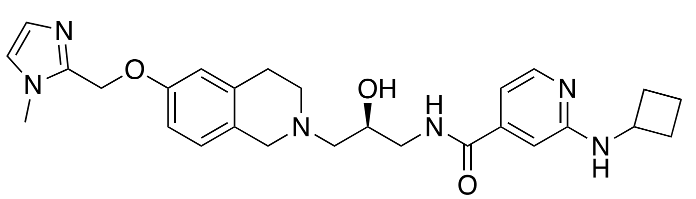

26

(*S*)-2-(cyclobutylamino)-N-(2-hydroxy-3-(6-((1-methyl-1*H*-imidazol-2-yl)methoxy)-3,4-dihydroisoquinolin-2(1*H*)-yl)propyl)isonicotinamide (**26**).
Amine: **intermediate 26a**. Brown solid, 5% yield. ^{1}H NMR (400 MHz, MeOH-*d$_4$*) *δ* ppm 7.90 (d, J = 5.5 Hz, 1H), 7.11 (s, 1H), 6.97 (d, J = 9.3 Hz, 1H), 6.93 (s, 1H), 6.78 - 6.84 (m, 3H), 6.74 (dd, J = 5.5, 1.3 Hz, 1H), 5.11 (s, 2H), 4.18 - 4.27 (m, 1H), 4.04 - 4.12 (m, 1H), 3.75 (s, 3H), 3.67 (s, 2H), 3.47 - 3.54 (m, 1H), 3.39 - 3.46 (m, 1H), 2.79 -

2.93 (m, 4H), 2.58 - 2.70 (m, 2H), 2.36 - 2.44 (m, 2H), 1.87 - 1.96 (m, 2H), 1.74 - 1.82 (m, 2H); LCMS (ESI) [M+H]+ m/z: calcd 491.2; found 491.3; HPLC: 92.85% @254nm; 99.2%ee.

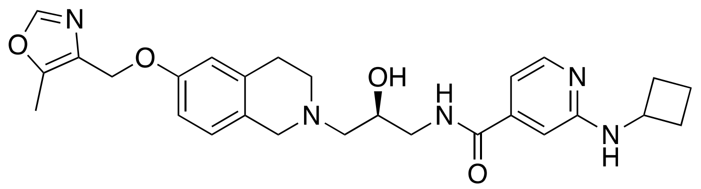

27

(*S*)-2-(cyclobutylamino)-*N*-(2-hydroxy-3-(6-((5-methyloxazol-4-yl)methoxy)-3,4-dihydroisoquinolin-2(1*H*)-yl)propyl)isonicotinamide (**27**).
Amine: **intermediate 27a**. Yellow solid, 2% yield. ^{1}H NMR (500 MHz, DMSO-d_6) δ (ppm) 1.70 (m, 2H), 1.88 (m, 2H), 2.29 (m, 2H), 2.37 (s, 3H), 2.46 (m, 1H), 2.70 (m, 2H), 2.80 (m, 2H), 3.23 (m, 1H), 3.41 (m, 2H), 3.57 (m, 2H), 3.91 (m, 1H), 4.28 (m, 1H), 4.84 (m, 1H), 4.91 (s, 2H), 6.76 (m, 4H), 6.96 (m, 2H), 7.97 (d, 1H), 8.24 (s, 1H), 8.49 (t, 1H). LCMS(ESI): [M+H]$^+$ m/z: calcd 491.2; found 492.0; Rt = 0.85 min.

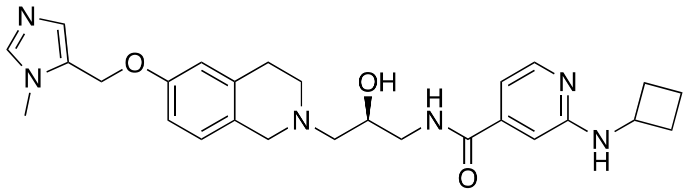

28

(*S*)-2-(cyclobutylamino)-*N*-(2-hydroxy-3-(6-((1-methyl-1*H*-imidazol-5-yl)methoxy)-3,4-dihydroisoquinolin-2(1*H*)-yl)propyl)isonicotinamide (**28**).
Amine: **intermediate 28a**. Light yellow solid, 10% yield. ^{1}H NMR (400 MHz, methnaol-d_4) δ ppm 7.91 (d, J = 5.4 Hz, 1 H), 7.67 (s, 1 H), 7.06 (s, 1 H), 7.00 (d, J = 8.3 Hz, 1 H), 6.79 - 6.85 (m, 3 H), 6.75 (dd, J = 5.4, 1.4 Hz, 1 H), 5.08 (s, 2 H), 4.25 (t, J = 8.1 Hz, 1 H), 4.04 - 4.14 (m, 1 H), 3.75 (s, 3 H), 3.70 (s, 2 H), 3.45 - 3.56 (m, 2 H), 2.82 - 2.94 (m, 4 H), 2.62 - 2.71 (m, 2 H), 2.37 - 2.47 (m, 2 H), 1.89 - 2.01 (m, 2 H), 1.74 - 1.85 (m, 2 H); LCMS (ESI) [M+H]$^+$ calcd 491.3, found 491.2; HPLC 96.14% @ 220nm; 99.8%ee.

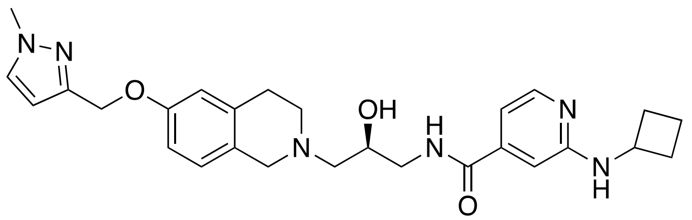

29

(*S*)-2-(cyclobutylamino)-*N*-(2-hydroxy-3-(6-((1-methyl-1*H*-pyrazol-3-yl)methoxy)-3,4-dihydroisoquinolin-2(1*H*)-yl)propyl)isonicotinamide (**29**).
Amine: **intermediate 29a**. Light yellow solid, 29% yield. ^{1}H NMR (400 MHz, methanol-*d*) δ ppm 7.91 (d, J = 5.4 Hz, 1H), 7.57 (d, J = 2.0 Hz, 1H), 6.97 (d, J = 8.4 Hz, 1H), 6.73 - 6.81 (m, 4H), 6.35 (d, J = 2.0 Hz, 1H), 5.01 (s, 2H), 4.21 - 4.29 (m, 1H), 4.06 - 4.12 (m, 1H), 3.90 (s, 3H), 3.69 (s, 2H), 3.46 - 3.51 (m, 2H), 2.89 (d, J = 5.0 Hz, 2H), 2.85 (m, 2H), 2.61 - 2.69 (m, 2H), 2.42 (d, J = 7.6 Hz, 2H), 1.89 - 1.99 (m, 2H), 1.73 - 1.83 (m, 2H); LCMS (ESI) [M+H]$^+$ m/z: calcd 491.3, found 491.2; HPLC: 94.61%@254nm; 99.6%ee.

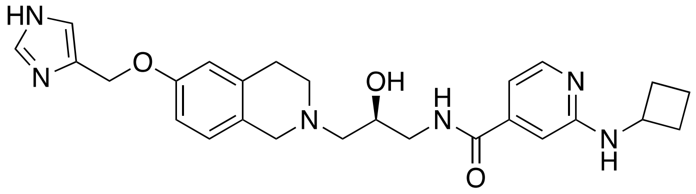

30

(*S*)-*N*-(3-(6-((1*H*-imidazol-4-yl)methoxy)-3,4-dihydroisoquinolin-2(1*H*)-yl)-2-hydroxypropyl)-2-(cyclobutylamino)isonicotinamide (**30**).

Amine: **intermediate 30a**, followed by the following procedure: To a solution of 2-(cyclobutylamino)-N-[(2*S*)-2-hydroxy-3-[6-[[1-(2-trimethylsilylethoxymethyl)imidazol-4-yl]methoxy]-3,4-dihydro-1*H*-isoquinolin-2-yl]propyl]pyridine-4-carboxamide and 2-(cyclobutylamino)-N-[(2*S*)-2-hydroxy-3-[6-[[3-(2-trimethylsilylethoxymethyl)imidazol-4-yl]methoxy]-3,4-dihydro-1*H*-isoquinolin-2-yl]propyl]pyridine-4-carboxamide (mixture, 200 mg, 0.33 mmol) in DCM (15 mL) was added TFA (3 mL) dropwise at 25 °C. The mixture was stirred at 25 °C for 3 h then concentrated under reduced pressure to give crude product, which was purified by preparative HPLC (Instrument: Gilson GX-281 Liquid Handler, Gilson 322 Pump, Gilson 156 UV Detector; Column: Xtimate C18 150 × 25 mm × 5 µm; Mobile phase A: H_2O with 0.225% FA (v%); Mobile phase B: MeCN; Gradient: B from 5% to 20% in 7 min, hold 100% B for 2 min; Flow Rate: 25 mL/min; Column Temperature: 30 °C; Wavelength: 220 nm, 254 nm) to give 2-(cyclobutylamino)-N-[(2*S*)-2-hydroxy-3-[6-(1*H*-imidazol-4-ylmethoxy)-3,4-dihydro-1*H*-isoquinolin-2-yl]propyl]pyridine-4-carboxamide, **30** (10 mg, 6% yield as a white hygroscopic solid. ^{1}H NMR (400 MHz, MeOH-d_4) δ ppm 8.91 (s, 1H), 7.92 (d, *J* = 6.6 Hz, 1H), 7.66 (s, 1H), 7.35 (s, 1H), 7.09 - 7.20 (m, 2H), 6.93 - 7.01 (m, 2H), 5.20 (s, 2H), 4.31 - 4.52 (m, 3H), 4.22 (quin, *J* = 7.8 Hz, 1H), 3.66 (s, 1H), 3.37 - 3.62 (m, 4H), 3.12 - 3.30 (m, 3H), 2.47 - 2.57 (m, 2H), 2.04 - 2.18 (m, 2H), 1.82 - 1.95 (m, 2H); LCMS (ESI) $[M+H]^+$ m/z: calcd 477.3, found 477.2; HPLC: 98.73%@254nm; 99.1%ee.

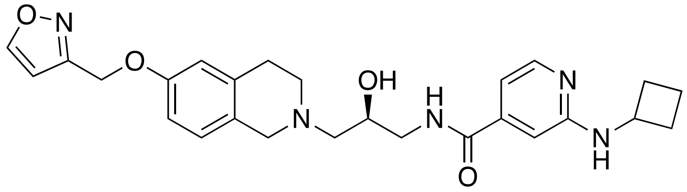

31

(*S*)-2-(cyclobutylamino)-N-(2-hydroxy-3-(6-(isoxazol-3-ylmethoxy)-3,4-dihydroisoquinolin-2(1*H*)-yl)propyl)isonicotinamide (**31**).

Amine: **intermediate 31a**. Yellow solid, 19% yield. ^{1}H NMR (400 MHz, $CDCl_3$) δ (ppm) 1.84 (m, 4H), 2.42 (m, 2H), 2.58 (m, 2H), 2.72 (m, 1H), 2.87 (m, 3H), 3.41 (m, 1H), 3.55 (d, 1H), 3.73 (m, 3H), 4.00 (m, 1H), 4.16 (h, 1H), 4.89 (d, 1H), 5.16 (s, 2H), 6.47 (d, 1H), 6.69 (s, 1H), 6.73 (m, 2H), 6.77 (dd, 1H), 6.88 (m, 1H), 6.93 (d, 1H), 8.08 (d, 1H), 8.40 (s, 1H). LCMS(ESI): $[M+H]^+$ m/z: calcd 477.2; found 478.2; Rt = 0.85 min.

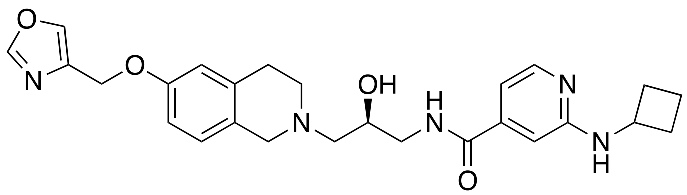

32

(*S*)-2-(cyclobutylamino)-N-(2-hydroxy-3-(6-(oxazol-4-ylmethoxy)-3,4-dihydroisoquinolin-2(1*H*)-yl)propyl)isonicotinamide (**32**).

Amine: **intermediate 32a**. Yellow solid, 27% yield. ^{1}H NMR (400 MHz, MeOH-d_4) δ ppm 8.23 (s, 1 H), 7.99 (s, 1 H), 7.89 (d, *J* = 5.5 Hz, 1 H), 6.97 (d, *J* = 8.3 Hz, 1 H), 6.76 - 6.81 (m, 3 H), 6.73 (dd, *J* = 5.4, 1.4 Hz, 1 H), 4.99 (s, 2 H), 4.23 (quin, *J* = 7.8 Hz, 1 H), 4.08 (quin, *J* = 6.0 Hz, 1 H), 3.68 (s, 2 H), 3.47 (qd, *J* = 13.7, 5.9 Hz, 2 H), 2.80 - 2.92 (m, 4 H), 2.60 - 2.70 (m, 2 H), 2.36 - 2.44 (m, 2 H), 1.87 - 1.97 (m, 2 H), 1.71 - 1.82 (m, 2 H); LCMS (ESI) $[M+H]^+$ m/z: calcd 478.2, found 478.2; HPLC: 100%@ 220 nm; 100%ee.

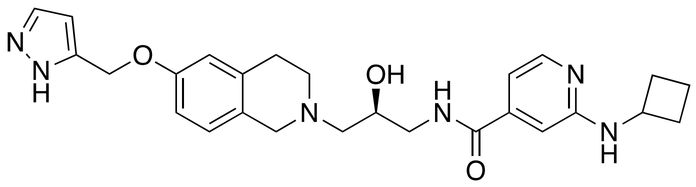

33

(*S*)-N-(3-(6-((1*H*-pyrazol-5-yl)methoxy)-3,4-dihydroisoquinolin-2(1*H*)-yl)-2-hydroxypropyl)-2-(cyclobutylamino)isonicotinamide (**33**).
Amine: **intermediate 33a**. White solid, 6% yield. ^{1}H NMR (400 MHz, MeOH-d_4) δ ppm 7.90 (d, *J* = 5.5 Hz, 1 H), 7.64 (br s, 1 H), 6.96 (d, *J* = 8.3 Hz, 1 H), 6.76 - 6.81 (m, 3 H), 6.74 (d, *J* = 5.5 Hz, 1 H), 6.39 (br s, 1 H), 5.06 (br s, 2 H), 4.23 (quin, *J* = 7.8 Hz, 1 H), 4.09 (quin, *J* = 6.0 Hz, 1 H), 3.70 (s, 2 H), 3.42 - 3.55 (m, 2 H), 2.81 - 2.92 (m, 4 H), 2.62 - 2.73 (m, 2 H), 2.35 - 2.44 (m, 2 H), 1.87 - 1.98 (m, 2 H), 1.72 - 1.82 (m, 2 H); LCMS (ESI) [M+H]+ m/z: calcd 477.2, found 477.2; HPLC: 100%@ 254 nm; 99.0%ee

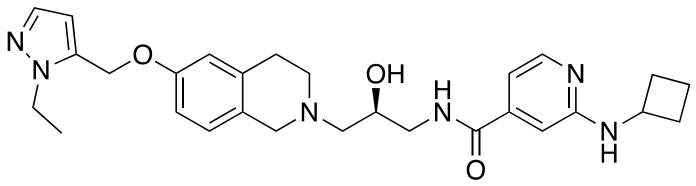

34

(*S*)-2-(cyclobutylamino)-N-(3-(6-((1-ethyl-1*H*-pyrazol-5-yl)methoxy)-3,4-dihydroisoquinolin-2(1*H*)-yl)-2-hydroxypropyl)isonicotinamide (**34**).
Amine: **intermediate 34a**. Yellow solid, 16% yield. ^{1}H NMR (500 MHz, DMSO-d_6) δ (ppm) 1.33 (t, 3H), 1.66 (m, 2H), 1.85 (m, 2H), 2.25 (m, 2H), 2.45 (m, 2H), 2.70 (m, 2H), 2.77 (m, 2H), 3.20 (m, 1H), 3.39 (m, 1H), 3.54 (m, 2H), 3.88 (h, 1H), 4.12 (q, 2H), 4.25 (h, 1H), 4.81 (d, 1H), 5.10 (s, 2H), 6.34 (d, 1H), 6.74 (m, 2H), 6.78 (m, 2H), 6.94 (dd, 2H), 7.39 (d, 1H), 7.95 (d, 1H), 8.47 (t, 1H). LCMS(ESI): $[M+H]^+$ m/z: calcd 504.3; found 505.4; Rt = 0.91 min.

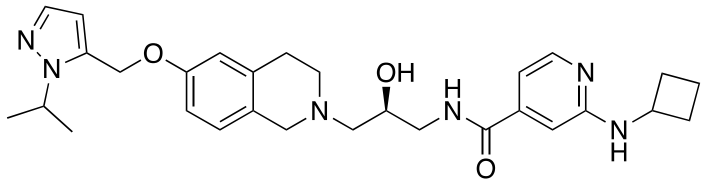

35

(*S*)-2-(cyclobutylamino)-N-(2-hydroxy-3-(6-((1-isopropyl-1*H*-pyrazol-5-yl)methoxy)-3,4-dihydroisoquinolin-2(1*H*)-yl)propyl)isonicotinamide (**35**).
Amine: **intermediate 35a**. Yellow solid. 15% yield. ^{1}H NMR (500 MHz, DMSO-d_6) δ (ppm) 1.38 (d, 6H), 1.65 (m, 2H), 1.88 (m, 2H), 2.26 (m, 2H), 2.43 (m, 2H), 2.67 (m, 2H), 2.77 (m, 2H), 3.22 (m, 1H), 3.37 (m, 1H), 3.56 (m, 2H), 3.88 (m, 1H), 4.25 (h, 1H), 4.57 (hept, 1H), 4.81 (d, 1H), 5.11 (m, 2H), 6.31 (m, 1H), 6.73 (dt, 1H), 6.77 (m, 3H), 6.94 (m, 2H), 7.40 (s, 1H), 7.95 (d, 1H), 8.47 (t, 1H). LCMS(ESI): $[M+H]^+$ m/z: calcd 518.3; found 519.4; Rt = 0.94 min.

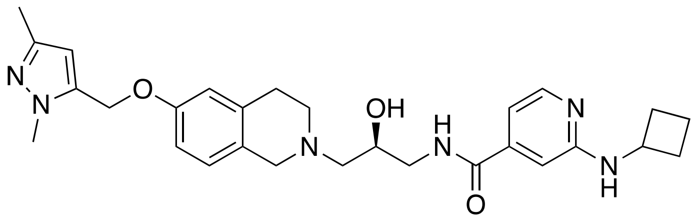

36

(*S*)-2-(cyclobutylamino)-N-(3-(6-((1,3-dimethyl-1*H*-pyrazol-5-yl)methoxy)-3,4-dihydroisoquinolin-2(1*H*)-yl)-2-hydroxypropyl)isonicotinamide (**36**).
Amine: **intermediate 36a**. Yellow solid, 24% yield. ^{1}H NMR (400 MHz, DMSO-d_6) δ (ppm) 1.64 (m, 3H), 1.86 (m, 2H), 2.27 (s, 3H), 2.67 (m, 3H), 2.69 (m, 1H), 2.77 (m, 2H), 3.18 (m, 2H), 3.20 (m, 1H), 3.39 (m, 1H), 3.55 (m, 2H), 3.71 (s, 3H), 4.25 (m, 1H), 4.81 (d, 1H), 5.03 (s, 2H), 6.10 (s, 1H), 6.74 (m, 3H), 6.94 (m, 2H), 7.94 (dd, 1H), 8.46 (t, 1H). LCMS(ESI): $[M+H]^+$ m/z: calcd 504.2; found 505.2; Rt = 0.88 min.

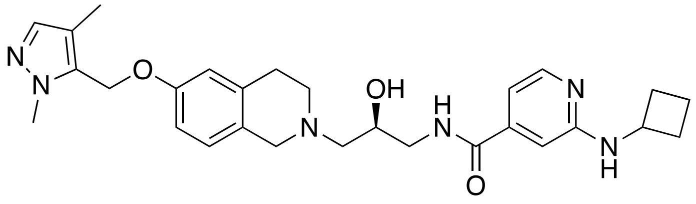

37

(*S*)-2-(cyclobutylamino)-N-(3-(6-((1,4-dimethyl-1*H*-pyrazol-5-yl)methoxy)-3,4-dihydroisoquinolin-2(1*H*)-yl)-2-hydroxypropyl)isonicotinamide (**37**).
Amine: **intermediate 37a**. Yellow solid, 13% yield. ^{1}H NMR (500 MHz, DMSO-d_6) δ (ppm) 1.64 (m, 2H), 1.85 (m, 2H), 2.01 (s, 3H), 2.26 (m, 2H), 2.44 (m, 1H), 2.69 (m, 2H), 2.78 (m, 2H), 3.19 (m, 1H), 3.37 (m, 2H), 3.56 (m, 2H), 3.77 (s, 3H), 3.89 (m, 1H), 4.25 (m, 1H), 4.80 (m, 1H), 5.02 (s, 2H), 6.74 (m, 2H), 6.78 (m, 2H), 6.94 (m, 2H), 7.21 (s, 1H), 7.96 (d, 1H), 8.46 (t, 1H). LCMS(ESI): $[M+H]^+$ m/z: calcd 504.2; found 505.2; Rt = 0.88 min.

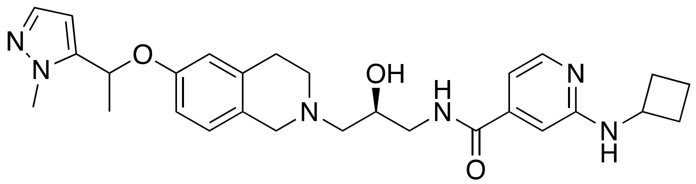

38

2-(cyclobutylamino)-N-((2*S*)-2-hydroxy-3-(6-(1-(1-methyl-1*H*-pyrazol-5-yl)ethoxy)-3,4-dihydroisoquinolin-2(1*H*)-yl)propyl)isonicotinamide (**38**).
Amine: **intermediate 38a**. Yellow solid, 17% yield. ^{1}H NMR($CDCl_3$, 400 MHz) δ (ppm) 1.68 (d, 3H), 1.82 (m, 4H), 2.44 (m, 2H), 2.59 (m, 2H), 2.74 (m, 1H), 2.89 (m, 3H), 3.41 (m, 2H), 3.57 (d, 1H), 3.70 (m, 1H), 3.78 (d, 1H), 3.86 (s, 3H), 4.02 (m, 1H), 4.17 (m, 1H), 4.90 (d, 1H), 5.40 (q, 1H), 6.24 (d, 1H), 6.67 (d, 2H), 6.74 (m, 1H), 6.86 (t, 1H), 6.90 (d, 1H), 7.39 (d, 1H), 8.09 (d, 1H). LCMS(ESI): $[M+H]^+$ m/z: calcd 504.3; found 505.2; Rt = 0.84 min.

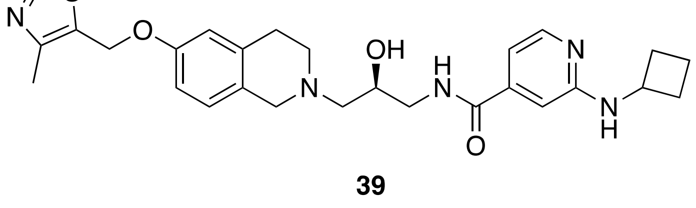

39

(*S*)-2-(cyclobutylamino)-N-(2-hydroxy-3-(6-((4-methyloxazol-5-yl)methoxy)-3,4-dihydroisoquinolin-2(1*H*)-yl)propyl)isonicotinamide (**39**).
Amine: **intermediate 39a**. White solid, 31% yield. ^{1}H NMR (400 MHz, MeOH-d_4) δ ppm 8.14 (s, 1 H), 7.90 (d, *J* = 5.5 Hz, 1 H), 6.98 (d, *J* = 8.0 Hz, 1 H), 6.71 - 6.81 (m, 4 H), 5.06 (s, 2 H), 4.23 (t, *J* = 7.9 Hz, 1 H), 4.09 (quin, *J* = 5.9 Hz, 1 H), 3.69 (s, 2 H), 3.43 - 3.54 (m, 2 H), 2.89 (br d, *J* = 5.8 Hz, 2 H), 2.80 - 2.88 (m, 2 H), 2.60 - 2.70 (m, 2 H), 2.36 - 2.46 (m, 2 H), 2.21 (s, 3 H), 1.85 - 1.99 (m, 2 H), 1.71 - 1.84 (m, 2 H); LCMS (ESI) $[M+H]^+$ m/z: calcd 492.3, found 492.2; HPLC: 100%@254nm; 100%ee.

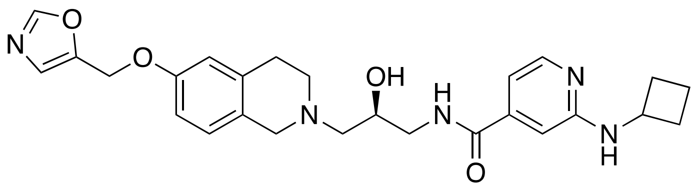

40

(*S*)-2-(cyclobutylamino)-N-(2-hydroxy-3-(6-(oxazol-5-ylmethoxy)-3,4-dihydroisoquinolin-2(1*H*)-yl)propyl)isonicotinamide (**40**).
Amine: **intermediate 40a**. Light yellow solid, 35% yield. ^{1}H NMR (400 MHz, methanol-*d*) δ ppm 8.26 (s, 1H), 7.91 (d, *J* = 5.5 Hz, 1H), 7.25 (s, 1H), 6.99 (d, *J* = 8.0 Hz, 1H), 6.80 (d, *J* = 8.0 Hz, 3H), 6.75 (dd, *J* = 5.5, 1.3 Hz, 1H), 5.12 (s, 2H), 4.20 - 4.28 (m, 1H), 4.07 - 4.13 (m, 1H), 3.69 (s, 2H), 3.13 - 3.54 (m, 2H), 2.90 (d, *J* = 5.8 Hz, 2H), 2.80 - 2.87 (m, 2H), 2.61 - 2.71 (m, 2H), 2.37 - 2.46 (m, 2H), 1.88 - 1.99 (m, 2H), 1.73 - 1.83 (m, 2H); LCMS (ESI) $[M+H]^+$ m/z: calcd 478.2, found 478.2; HPLC: 100%@254nm; 99.7%ee.

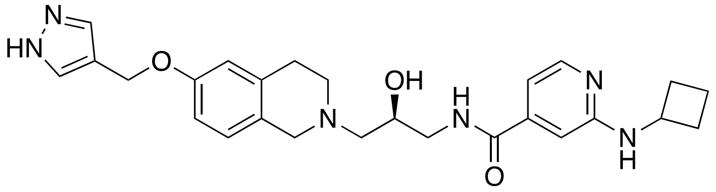

41

(*S*)-N-(3-(6-((1*H*-pyrazol-4-yl)methoxy)-3,4-dihydroisoquinolin-2(1*H*)-yl)-2-hydroxypropyl)-2-(cyclobutylamino)isonicotinamide (**41**).
Amine: **intermediate 41a**. White solid, 31% yield. ^{1}H NMR (400 MHz, MeOH-d_4) δ ppm 7.89 (d, *J* = 5.4 Hz, 1 H), 7.67 (br s, 2 H), 6.96 (d, *J* = 8.3 Hz, 1 H), 6.71 - 6.80 (m, 4 H), 4.99 (s, 2 H), 4.22 (quin, *J* = 7.9 Hz, 1 H), 4.08 (quin, *J* = 6.0 Hz, 1 H), 3.68 (s, 2 H), 3.41 - 3.53 (m, 2 H), 2.80 - 2.92 (m, 4 H), 2.60 - 2.70 (m, 2 H), 2.35 - 2.45 (m, 2 H), 1.86 - 1.99 (m, 2 H), 1.72 - 1.82 (m, 2 H); LCMS (ESI) $[M+H]^+$ m/z: calcd 477.3, found 477.2; HPLC: 98.42%@ 254 nm; 99.2%ee.

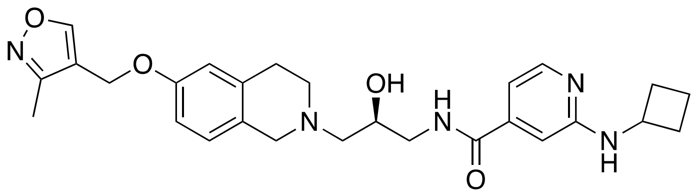

42

(*S*)-2-(cyclobutylamino)-N-(2-hydroxy-3-(6-((3-methylisoxazol-4-yl)methoxy)-3,4-dihydroisoquinolin-2(1*H*)-yl)propyl)isonicotinamide (**42**).
Amine: **intermediate 42a**. Yellow solid, 13% yield. LCMS(ESI): $[M+H]^+$ m/z: calcd 491.2; found 492.2; Rt = 0.81 min. ^{1}H NMR (400 MHz, $CDCl_3$) δ (ppm) 1.83 (m, 4H), 2.34 (s, 3H), 2.43 (m, 2H), 2.56 (m, 2H), 2.72 (m, 1H), 2.88 (m, 3H), 3.41 (m, 2H), 3.56 (d, 1H), 3.74 (m, 2H), 4.01 (m, 1H), 4.17 (h, 1H), 4.86 (s, 2H), 4.89 (m, 1H), 6.69 (m, 2H), 6.74 (m, 2H), 6.88 (m, 1H), 6.94 (d, 1H), 8.08 (d, 1H), 8.35 (s, 1H).

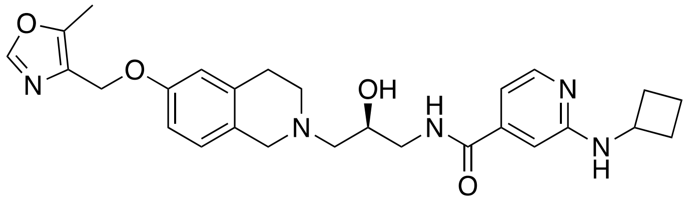

43

(*S*)-2-(cyclobutylamino)-N-(2-hydroxy-3-(6-((5-methyloxazol-4-yl)methoxy)-3,4-dihydroisoquinolin-2(1*H*)-yl)propyl)isonicotinamide (**43**).
Amine: **intermediate 43a**. Yellow soid, 2% yield. ^{1}H NMR (500 MHz, DMSO-d_6) δ (ppm) 1.70 (m, 2H), 1.88 (m, 2H), 2.29 (m, 2H), 2.37 (s, 3H), 2.46 (m, 1H), 2.70 (m, 2H), 2.80 (m, 2H), 3.23 (m, 1H), 3.41 (m, 2H), 3.57 (m, 2H), 3.91 (m, 1H), 4.28 (m, 1H), 4.84 (m, 1H), 4.91 (s, 2H), 6.76 (m, 4H), 6.96 (m, 2H), 7.97 (d, 1H), 8.24 (s, 1H), 8.49 (t, 1H). LCMS(ESI): $[M+H]^+$ m/z: calcd 491.2; found 492.0; Rt = 0.85 min.

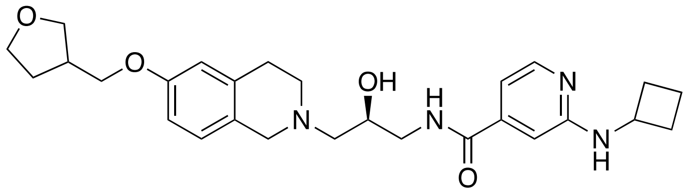

44

2-(cyclobutylamino)-N-((2*S*)-2-hydroxy-3-(6-((tetrahydrofuran-3-yl)methoxy)-3,4-dihydroisoquinolin-2(1*H*)-yl)propyl)isonicotinamide (**44**).
Amine: **intermediate 44a**. Off-white solid, 59% yield. ^{1}H NMR (400 MHz, MeOH-d_4) δ ppm 7.89 (d, J = 5.5 Hz, 1 H), 6.94 (d, J = 8.3 Hz, 1 H), 6.78 (s, 1 H), 6.65 - 6.74 (m, 3 H), 4.15 - 4.29 (m, 1 H), 4.03 - 4.12 (m, 1 H), 3.82 - 3.97 (m, 4 H), 3.77 (q, J = 7.7 Hz, 1 H), 3.69 (d, J = 5.6 Hz, 1 H), 3.67 (s, 2 H), 3.39 - 3.56 (m, 2 H), 2.78 - 2.93 (m, 4 H), 2.57 - 2.75 (m, 3 H), 2.34 - 2.46 (m, 2 H), 2.05 - 2.18 (m, 1 H), 1.86 - 1.98 (m, 2 H), 1.70 - 1.82 (m, 3 H); LCMS (ESI) $[M+H]^+$ m/z calcd 481.3, found 481.2; HPLC, 100%@254nm; racemic.

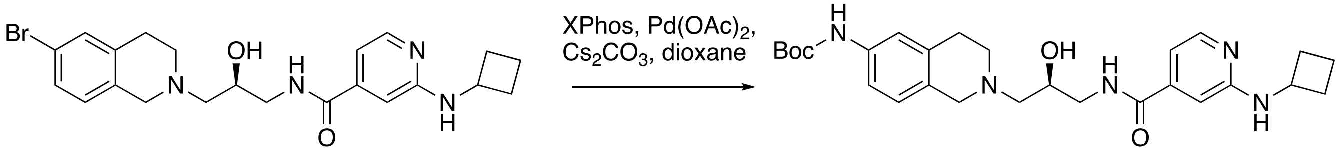

tert-butyl (*S*)-(2-(3-(2-(cyclobutylamino)isonicotinamido)-2-hydroxypropyl)-1,2,3,4-tetrahydroisoquinolin-6-yl)carbamate.
To a sealed tube were added N-[(2*S*)-3-(6-bromo-3,4-dihydro-1*H*-isoquinolin-2-yl)-2-hydroxy-propyl]-2-(cyclobutylamino)pyridine-4-carboxamide (100 mg, 0.22 mmol, 1 eq), XPhos (20.8 mg, 0.05 mmol, 0.2 eq), Pd(OAc)$_2$ (10 mg, 0.05 mmol, 0.2 eq), Cs_2CO_3 (70.9 mg, 0.22 mmol, 1 eq) and dioxane (5 mL). Then the mixture was degassed, sealed and stirred for 16 h at 120 °C. The mixture was concentrated under reduced pressure and the residue was purified by flash chromatography (ISCO®; 4 g of SepaFlash® Silica Flash Column, petroleum ether/EtOAc with EtOAc from 50~100%, flow rate = 20 mL/min) to afford *tert*-butyl (*S*)-(2-(3-(2-(cyclobutylamino)isonicotinamido)-2-hydroxypropyl)-1,2,3,4-tetrahydroisoquinolin-6-yl)carbamate (55 mg, 51%) as yellow oil which was used in next step.

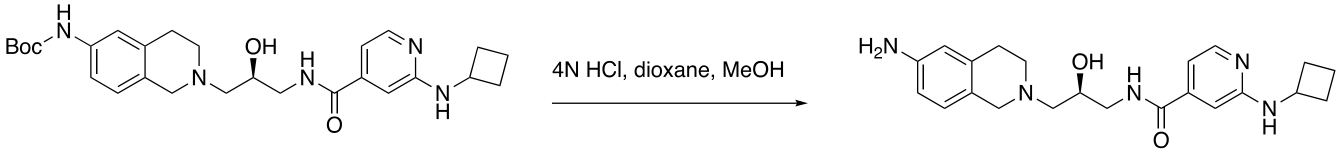

4

(*S*)-N-(3-(6-amino-3,4-dihydroisoquinolin-2(1*H*)-yl)-2-hydroxypropyl)-2-(cyclobutylamino)isonicotinamide (4). A mixture of *tert*-butyl (*S*)-(2-(3-(2-(cyclobutylamino)isonicotinamido)-2-hydroxypropyl)-1,2,3,4-tetrahydroisoquinolin-6-yl)carbamate (55 mg, 0.12 mmol, 1 eq) in MeOH (2 mL) was added 4M HCl/dioxane (2 mL, 8 mmol, 72.1 eq) and the mixture was stirred at 25 °C for 3 h. The mixture was concentrated under reduced pressure to give an oil which was purified by preparative HPLC (Instrument: Gilson GX-281 Liquid Handler, Gilson 322 Pump, Gilson 156 UV Detector; Column: Durashell 150 × 25 mm × 5 µm; Mobile phase A: H_2O with 0.05% NH_3-H_2O (v%); Mobile phase B: MeCN; Gradient: B from 20% to 50% in 7.8 min, hold 100% B for 2.5 minutes; Flow Rate: 25 mL/min; Column Temperature: 30 °C; Wavelength: 220 nm, 254 nm) to afford N-[(2*S*)-3-(6-amino-3,4-dihydro-1*H*-isoquinolin-2-yl)-2-hydroxy-propyl]-2-(cyclobutylamino)pyridine-4-carboxamide, **4** (12 mg, 26%) as a white solid. ^{1}H NMR (400 MHz, MeOH-*d*$_4$) δ ppm 7.91 - 7.94 (m, 1H), 6.79 - 6.84 (m, 2H), 6.74 - 6.76 (m, 1H), 6.55 - 6.58 (m, 1H), 6.52 (d, *J* = 2.3 Hz, 1H), 4.21 - 4.39 (m, 1H), 4.06 - 4.13 (m, 1H), 3.64 (s, 2H), 3.43 - 3.53 (m, 2H), 2.81 - 2.85 (m, 4H), 2.60 - 2.70 (m, 2H), 2.38 - 2.46 (m, 2H), 1.90 - 2.00 (m, 2H), 1.75 - 1.83 (m, 2H); LCMS (ESI) [M+H]$^+$ m/z: calcd 396.2; found 396.2; HPLC, 96.0%@220 nm; 93.9 %ee.

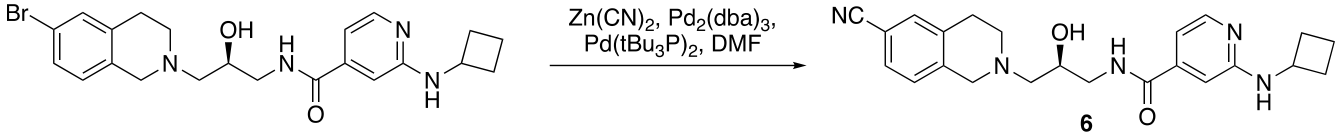

6

(*S*)-N-(3-(6-cyano-3,4-dihydroisoquinolin-2(1*H*)-yl)-2-hydroxypropyl)-2-(cyclobutylamino)isonicotinamide (6). To a solution of intermediate 4a (50 mg, 0.11 mmmol, 1 eq) in DMF (5 mL) was added $Zn(CN)_2$ (19.2 mg, 0.16 mmol, 1.5 eq), $Pd(^tBu_3P)_2$ (8.3 mg, 0.02 mmol, 0.15 eq), and $Pd_2(dba)_3$ (15.0 mg, 0.02 mmol, 0.15 eq). The mixture was degassed, sealed, and stirred at 100 °C for 16 h. The mixture was diluted with water (15 mL) and extracted with EtOAc (10 mL x 3). The combined organic layers were washed with saturated NH_4Cl aqueous solution (10 mL x 2), brine (10 mL x 2), dried over anhydrous Na_2SO_4, filtered, and concentrated under reduced pressure. The residue was purified by flash chromatography (ISCO®; 4 g SepaFlash® Silica Flash Column, DCM/MeOH with MeOH from 0~5%, Flow Rate = 20 mL/min) to afford crude product. This material was further purified by preparative HPLC (Instrument: Gilson GX-281 Liquid Handler, Gilson 322 Pump, Gilson 156 UV Detector; Column: Durashell 150 × 25 mm × 5 µm; Mobile phase A: H_2O with 0.05% NH_3-H_2O (v%); Mobile phase B: MeCN; Gradient: B from 40% to 70% in 6.5 minutes, hold 100% B for 2.5 min; Flow Rate: 25 mL/min; Column Temperature: 30 °C; Wavelength: 220 nm, 254 nm) then purified further by preparative HPLC (Instrument: Gilson GX-281 Liquid Handler, Gilson 322 Pump, Gilson 156 UV Detector; Column: Durashell 150 × 25 mm × 5 µm; Mobile phase A: H_2O with 0.05% NH_3-H_2O (v%); Mobile phase B: MeCN; Gradient: B from 40% to 70% in 6.5 min, hold 100% B for 2.5 min; Flow Rate: 25 mL/min; Column Temperature: 30 °C; Wavelength: 220 nm, 254 nm) to afford N-[(2S)-3-(6-cyano-3,4-dihydro-1*H*-isoquinolin-2-yl)-2-hydroxy-propyl]-2-(cyclobutylamino)pyridine-4-carboxamide **6** (20 mg, 45%) as a yellow solid. ^{1}H NMR (400 MHz, MeOH-*d*$_4$) δ ppm 7.95 (d, *J* = 5.4 Hz, H), 7.45 - 7.52 (m, 2H), 7.24 (d, *J* = 8.1 Hz, 1H), 6.77 - 6.82 (m, 2H), 4.20 - 4.24 (m, 1H), 4.06 - 4.13 (m, 1H), 3.81 (s, 2H), 3.56 (dd, *J* = 13.6, 5.3 Hz, 1H), 3.42 (dd, *J* = 13.6, 6.5 Hz, 1H), 2.92 - 3.01 (m, 2H), 2.86 - 2.91 (m, 2H), 2.62 - 2.72 (m, 2H), 2.43 (br d, *J* = 7.1 Hz, 2H), 1.89 - 2.00 (m, 2H), 1.74 - 1.85 (m, 2H); LCMS (ESI) [M+H]$^+$ m/z: calcd 406.2; found 406.2; HPLC, 99.47% @220 nm; 99.0%ee.

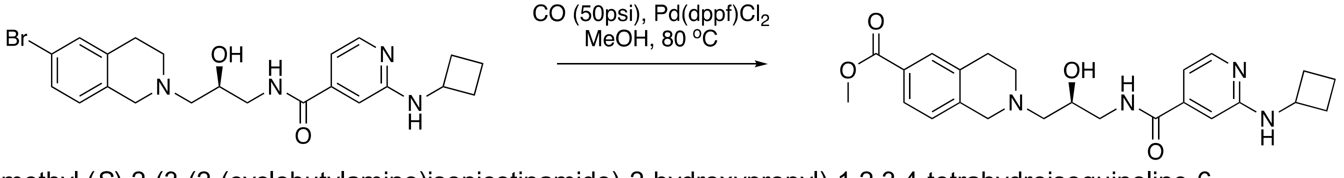

methyl (*S*)-2-(3-(2-(cyclobutylamino)isonicotinamido)-2-hydroxypropyl)-1,2,3,4-tetrahydroisoquinoline-6-carboxylate.

To a solution of intermediate 4a (2.5 g, 5.4 mmol, 1 eq) in MeOH (20 mL) was added Pd(dppf)Cl_2 (597.3 mg, 0.82 mmol, 0.15 eq), TEA (2.3 mL, 16.3 mmol, 3 eq). The suspension was degassed under vacuum and purged with Ar several times, then with CO gas 3 times. The mixture was stirred under CO (50 psi) at 80 °C for 96 h. The mixture was filtered, and the filtrate was concentrated under reduced pressure. The residue was diluted with EtOAc (50 mL) and washed with saturated NH_4Cl aqueous solution (50 mL x 3). The organic layer was dried over anhydrous Na_2SO_4, filtered and concentrated under reduced pressure to give methyl 2-[(2S)-3-[[2-(cyclobutylamino)pyridine-4-carbonyl]amino]-2-hydroxy-propyl]-3,4-dihydro-1*H*-isoquinoline-6-carboxylate (2.84 g, crude) as brown solid which was used into next step without further purification. ^{1}H NMR (400 MHz, $CDCl_3$) δ ppm 8.11 (d, *J* = 5.1 Hz, 1H), 7.78 - 7.83 (m, 2H), 7.08 (d, *J* = 7.8 Hz, 1H), 6.82 (br s, 1H), 6.76 (br d, *J* = 5.0 Hz, 1H), 6.71 (s, 1H), 4.92 (br d, *J* = 6.8 Hz, 1H), 4.15 - 4.23 (m, 1H), 4.05 (br d, *J* = 4.1 Hz, 1H), 3.91 (s, 3H), 3.70 - 3.77 (m, 2H), 3.40 - 3.48 (m, 1H), 2.97 (br s, 2H), 2.75 - 2.82 (m, 2H), 2.55 - 2.69 (m, 2H), 2.42 - 2.49 (m, 2H), 1.73 - 1.94 (m, 6H); LCMS (ESI) $[M+H]^+$m/z: calcd 439.2; found 439.2.

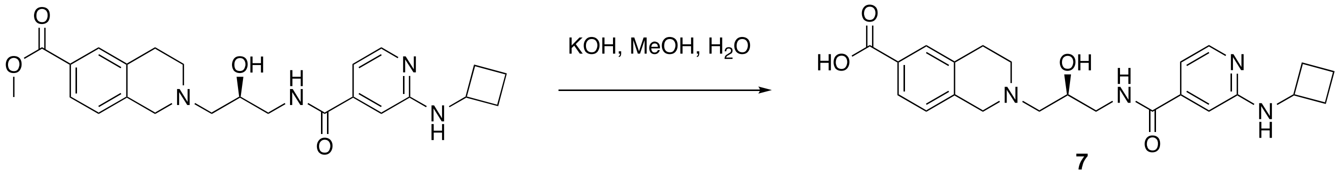

(*S*)-2-(3-(2-(cyclobutylamino)isonicotinamido)-2-hydroxypropyl)-1,2,3,4-tetrahydroisoquinoline-6-carboxylic acid (**7**). To a solution of methyl 2-[(2S)-3-[[2-(cyclobutylamino)pyridine-4-carbonyl]amino]-2-hydroxy-propyl]-3,4-dihydro-1*H*-isoquinoline-6-carboxylate (2.75 g, 6.27 mmol, 1 eq) in MeOH (15 mL) was added KOH (1.06 g, 18.8 mmol, 3 eq) in H_2O (15 mL), then the mixture was stirred at 25 °C for 12 h. The mixture was concentrated under reduced pressure to remove the MeOH and acidified with 2 N HCl/H_2O to pH = 5. The mixture was concentrated under vacuum and the residue was purified by preparative HPLC (Instrument: Phenomenex luna C18; Column: Durashell 250 × 50 mm × 10 µm; Mobile phase A: H_2O with 0.05% HCl (v%); Mobile phase B: MeCN; Gradient: B from 0% to 15% in 12 min, hold 100% B for 10 min; Flow Rate: 120 mL/min; Column Temperature: 30 °C; Wavelength: 220 nm, 254 nm) to afford 2-[(2S)-3-[[2-(cyclobutylamino)pyridine-4-carbonyl]amino]-2-hydroxy-propyl]-3,4-dihydro-1*H*-isoquinoline-6-carboxylic acid, **7** (1.22 g, 39% yield, HCl salt) as light yellow solid. ^{1}H NMR (400 MHz, DMSO-*d*6) δ ppm 10.85 (br s, 1 H), 9.90 (br s, 1 H), 9.39 (br s, 1 H), 8.00 - 8.18 (m, 1 H), 7.82 - 7.93 (m, 2 H), 7.59 (br s, 1 H), 7.36 - 7.44 (m, 1 H), 7.32 (d, *J* = 6.5 Hz, 1 H), 4.67 - 4.80 (m, 1 H), 4.54 (br dd, *J* = 15.9, 7.9 Hz, 2 H), 4.39 - 4.47 (m, 2 H), 3.91 (br d, *J* = 7.0 Hz, 1 H), 3.74 - 3.84 (m, 1 H), 3.25 - 3.53 (m, 6 H), 3.14 - 3.24 (m, 1 H), 2.48 - 2.55 (m, 2 H), 2.06 - 2.16 (m, 2 H), 1.74 - 1.89 (m, 2 H); HPLC, 92.6%; LCMS (ESI) $[M+H]^+$ m/z: calcd 425.2; found 425.2; 100%ee.

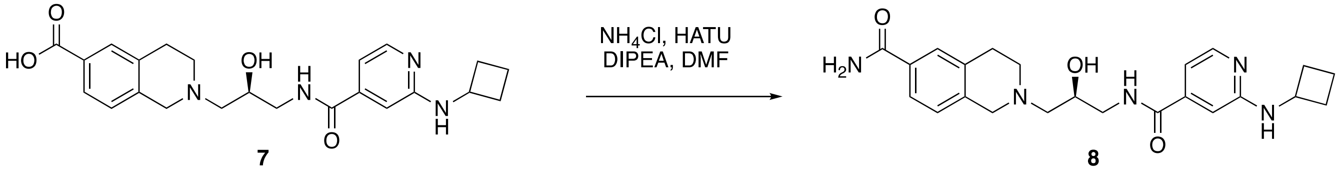

(*S*)-2-(3-(2-(cyclobutylamino)isonicotinamido)-2-hydroxypropyl)-1,2,3,4-tetrahydroisoquinoline-6-carboxamide (**8**). A mixture of 2-[(2*S*)-3-[[2-(cyclobutylamino)pyridine-4-carbonyl]amino]-2-hydroxy-propyl]-3,4-dihydro-1*H*-isoquinoline-6-carboxylic acid (75 mg, 0.16 mmol, HCl salt), NH_4Cl (13 mg, 0.24 mmol), HATU (75 mg, 0.20 mmol), DIPEA (105 mg, 0.81 mmol) and DCM (5 mL) was stirred at 25 °C for 12 h. The mixture was concentrated under reduced pressure and the residue was purified by preparative HPLC (Instrument: Gilson GX-281 Liquid Handler, Gilson 322 Pump, Gilson 156 UV Detector; Column: Waters Xbridge 150 × 25 mm × 5 µm; Mobile phase A: H_2O with 0.05% NH_3-H_2O (v%); Mobile phase B: MeCN; Gradient: B from 14% to 44% in 9.5 min, hold 100% B for 2.5 min; Flow Rate: 25 mL/min; Column Temperature: 30 °C; Wavelength: 220 nm, 254 nm) to give 2-[(2S)-3-[[2-(cyclobutylamino)pyridine-4-carbonyl]amino]-2-hydroxy-propyl]-3,4-dihydro-1*H*-isoquinoline-6-carboxamide, **8** (25 mg, 36% yield) as a white solid. ^{1}H NMR (400 MHz, MeOH-*d₄*) δ ppm 7.93 (d, *J* = 5.3 Hz, 1H), 7.58 - 7.72 (m, 2H), 7.16 (d, *J* = 8.0 Hz, 1H), 6.72 - 6.86 (m, 2H), 4.19 - 4.29 (m, 1H), 4.06 - 4.16 (m, 1H), 3.81 (s, 2H), 3.39 - 3.60 (m, 2H), 2.95 - 3.02 (m, 2H), 2.85 - 2.94 (m, 2H), 2.62 - 2.75 (m, 2H), 2.37 - 2.46 (m, 2H), 1.88 - 2.00 (m, 2H), 1.72 - 1.84 (m, 2H); LCMS $[M+H^+]$ m/z: calcd 424.2; found 424.2; HPLC: 99.7% @254nm; 99.6%ee.

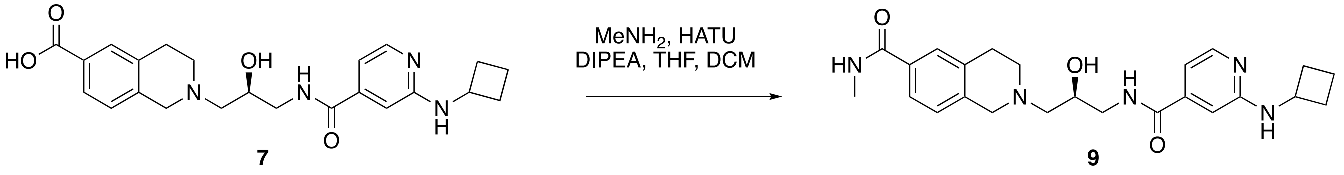

(*S*)-2-(3-(2-(cyclobutylamino)isonicotinamido)-2-hydroxypropyl)-*N*-methyl-1,2,3,4-tetrahydroisoquinoline-6-carboxamide (**9**). To a solution of **7** (75 mg, 0.16 mmol, HCl) in DCM (5 mL) was added HATU (92.8 mg, 244.06 umol) and DIPEA (61.5 mg, 0.48 mmol). After stirring for 15 minutes, methanamine/THF (0.1 mL, 0.2 mmol, 2M) was added and the mixture was stirred at 20 °C for 16 h. The resulting mixture was washed with saturated NH_4Cl aqueous solution (15 mL) and extracted with DCM (10 mL x 3). The combined organic layers were dried over anhydrous Na_2SO_4, filtered, and concentrated under reduced pressure. The residue was purified by preparative HPLC purification (Instrument: Gilson GX-281 Liquid Handler, Gilson 322 Pump, Gilson 156 UV Detector; Column: Durashell 150 × 25 mm × 5 µm; Mobile phase A: H_2O with 0.05% NH_3-H_2O (v%); Mobile phase B: MeCN; Gradient: B from 22% to 42% in 6.5 min, hold 100% B for 2.5 min; Flow Rate: 25 mL/min; Column Temperature: 30 °C; Wavelength: 220 nm, 254 nm) to afford 2-[(2S)-3-[[2-(cyclobutylamino)pyridine-4-carbonyl]amino]-2-hydroxy-propyl]-N-methyl-3,4-dihydro-1*H*-isoquinoline-6-carboxamide, **9** (33 mg, 46%) was obtained as white solid. ^1H NMR (400 MHz, MeOH-d_4) δ ppm 7.92 (d, J = 5.4 Hz, 1H), 7.56 - 7.61 (m, 2H), 7.15 (d, J = 7.8 Hz, 1H), 6.81 (s, 1H), 6.77 (dd, J = 5.4, 1.5 Hz, 1H), 4.20 -4.28 (m, 1H), 4.07 - 4.15 (m, 1H), 3.80 (s, 2H), 3.51 - 3.58 (m, 1H), 3.41 - 3.48 (m, 1H), 2.94 - 3.01 (m, 2H), 2.92 (s, 3H), 2.86 - 2.91 (m, 2H), 2.63 - 2.72 (m, 2H), 2.37 - 2.46 (m, 2H), 1.88 - 1.99 (m, 2H), 1.74 - 1.84 (m, 2H); HPLC: 100% @254 nm; LCMS (ESI) $[M+H]^+$ m/z: calcd 438.2; found 438.2; 98.9%ee.

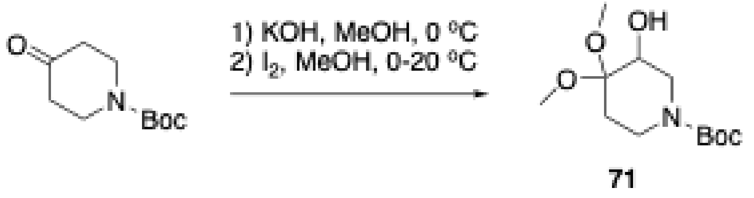

tert-butyl 3-hydroxy-4,4-dimethoxypiperidine-1-carboxylate (**71**)

A solution of KOH (3.52 g, 62.74 mmol) in MeOH (75 mL) was cooled to 0 °C then *tert*-butyl 4-oxopiperidine-1-carboxylate (5 g, 25.09 mmol) was added portion wise. After stirring for 20 minutes, a solution of iodine (7.64 g, 30.10 mmol) in MeOH (100 mL) was added dropwise at 0 °C. After the addition was complete, the mixture was stirred for 1 h at 0 °C, then warmed to 20 °C and stirred for another 12 h under N_2. The resulting mixture was concentrated under reduced pressure and the residue was diluted with toluene (200 mL) and stirred for 1 h at room temperature. The solid was removed by filtration, washed with toluene (50 mL), and the filtrate was concentrated in vacuum to afford *tert*-butyl 3-hydroxy-4,4-dimethoxy-piperidine-1-carboxylate, **71** (6.56 g, crude) as yellow oil which was used in next step directly. ^1H NMR (400 MHz, DMSO-d_6) δ ppm 4.76 (d, J = 3.9 Hz, 1 H), 3.67 - 3.91 (m, 2 H), 3.56 (s, 1 H), 3.32 (s, 1 H), 3.11 (s, 6 H), 2.83 - 3.05 (m, 1 H), 2.57 - 2.80 (m, 1 H), 1.56 - 1.73 (m, 2 H), 1.33 - 1.47 (s, 9H).

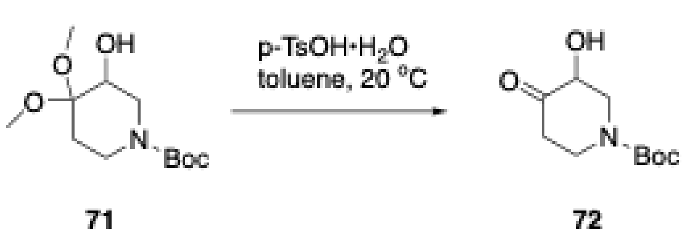

tert-butyl 3-hydroxy-4-oxopiperidine-1-carboxylate (**72**)

To a solution of **71** (6.56 g, 25.10 mmol) in acetone (100 mL) was added p-toluenesulfonic acid monohydrate (250 mg, 1.31 mmol) and the mixture was stirred for 72 h at 20 °C under N_2. The resulting mixture was concentrated under reduced pressure and the residue was dissolved in MTBE (150 mL). The organic layer was washed with saturated $NaHCO_3$ aqueous solution (100 mL x 2), brine (100 mL), dried over anhydrous Na_2SO_4 and filtered. The

solvent was removed in vacuum to afford *tert*-butyl 3-hydroxy-4-oxo-piperidine-1-carboxylate, **72** (5 g, crude) as yellow oil. LCMS (ESI) $[M+H-56]^+$ m/z calcd 160.1, found 160.1.

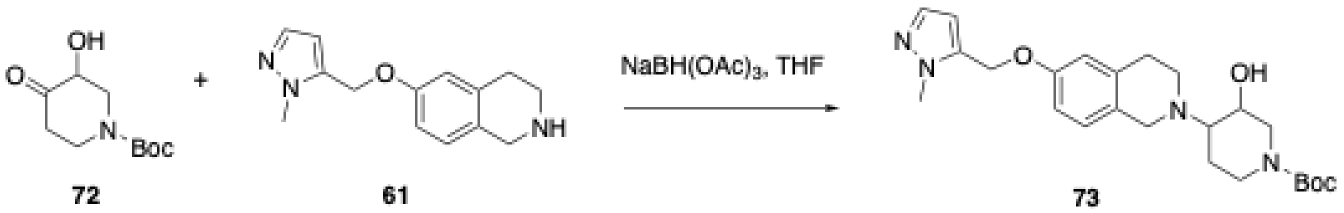

tert-butyl 3-hydroxy-4-(6-((1-methyl-1*H*-pyrazol-5-yl)methoxy)-3,4-dihydroisoquinolin-2(1*H*)-yl)piperidine-1-carboxylate (**73**)

To a solution of **72** (1.46 g, crude), and **61** (300 mg, 1.23 mmol) in THF (15 mL) was added $NaBH(OAc)_3$ (780 mg, 3.7 mmol). The mixture was stirred at 20 °C for 12 h. The resulting mixture was concentrated under reduced pressure, diluted with H_2O (20 mL) and extracted with EtOAc (25 mL x 3). The combined organic layers were dried over anhydrous Na_2SO_4, filtered, and concentrated under reduced pressure. The residue was purified by flash chromatography (ISCO®; 20 g of AgelaFlash® Silica Flash Column, DCM/MeOH with MeOH from 0~10%, flow rate = 30 mL/min) to afford a crude product (mixture of two isomers) which was purified by preparative HPLC purification (Instrument: Gilson GX-281 Liquid Handler, Gilson 322 Pump, Gilson 156 UV Detector; Column: Waters Xbridge 150 × 25 mm × 5 μm; Mobile phase A: H_2O with 0.05% NH_3-H_2O (v%); Mobile phase B: MeCN; Gradient: B from 40% to 70% in 7.8 min, hold 100% B for 1 min; Flow Rate: 25 mL/min; Column Temperature: 30 °C; Wavelength: 220 nm, 254 nm) to afford *tert*-butyl 3-hydroxy-4-[6-[(2-methylpyrazol-3-yl)methoxy]-3,4-dihydro-1*H*-isoquinolin-2-yl]piperidine-1-carboxylate (58 mg, 11%, *cis*) as light-yellow solid and *tert*-butyl 3-hydroxy-4-[6-[(2-methylpyrazol-3-yl)methoxy]-3,4-dihydro-1*H*-isoquinolin-2-yl]piperidine-1-carboxylate (33 mg, 6%, *trans*) as light-yellow solid.

tert-butyl 3-hydroxy-4-[6-[(2-methylpyrazol-3-yl)methoxy]-3,4-dihydro-1*H*-isoquinolin-2-yl]piperidine-1-carboxylate (58 mg, 11%, *cis*): ^{1}H NMR (400 MHz, MeOH-d_4) δ 7.42 (d, J = 1.8 Hz, 1H), 7.01 (d, J = 8.3 Hz, 1H), 6.79 - 6.84 (m, 2H), 6.38 (d, J = 1.8 Hz, 1H), 5.12 (s, 2H), 4.18 (br s, 3H), 3.90 (s, 3H), 3.84 (s, 2H), 2.20 - 3.03 (m, 6H), 2.50 (d, J = 11.3 Hz, 1H), 1.87 - 1.98 (m, 1H), 1.77 - 1.84 (m, 1H), 1.48 (s, 9H); LCMS (ESI) $[M+H]^+$ m/z: calcd 443.3, found 443.2. *tert*-butyl 3-hydroxy-4-[6-[(2-methylpyrazol-3-yl)methoxy]-3,4-dihydro-1*H*-isoquinolin-2-yl]piperidine-1-carboxylate, **73** (33 mg, 6%, *trans*): ^{1}H NMR (400 MHz, MeOH-d_4) δ 7.42 (d, J = 1.8 Hz, 1H), 7.00 (d, J = 8.3 Hz, 1H), 6.78 - 6.82 (m, 2H), 6.37 (d, J = 2.0 Hz, 1H), 5.12 (s, 2H), 4.24 (d, J = 10.5 Hz, 1H), 4.12 (d, J = 13.6 Hz, 1H), 3.90 (s, 3H), 3.76 - 3.88 (m, 2H), 3.65 (dd, J = 9.8, 5.0 Hz, 1H), 2.98 - 3.02 (m, 1H), 2.76 - 2.92 (m, 4H), 2.58 - 2.69 (m, 2H), 1.89 (d, J = 13.3 Hz, 1H), 1.51 - 1.58 (m, 1H), 1.49 (s, 9H); LCMS (ESI) $[M+H]^+$ m/z: calcd 443.3, found 443.2.

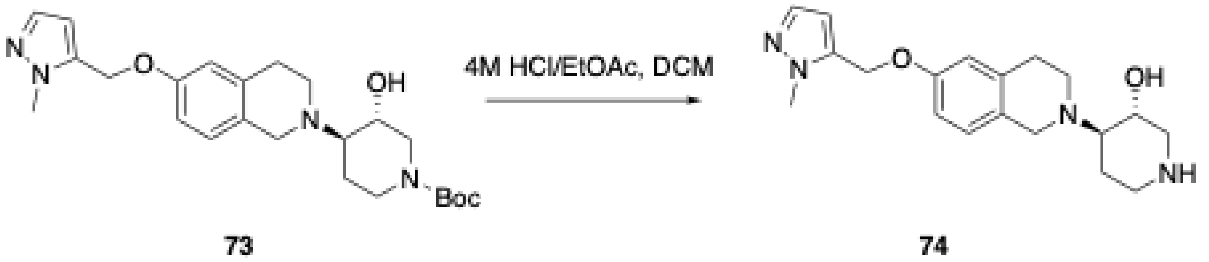

4-[6-[(2-methylpyrazol-3-yl)methoxy]-3,4-dihydro-1*H*-isoquinolin-2-yl]piperidin-3-ol (**74**)

To a solution of **73** (30.0 mg, 0.07 mmol, *trans*) in DCM (3 mL) was added 4M HCl/EtOAc (1 mL, 4 mmol). The mixture was stirred at 20 °C for 2 h. The resulting mixture was concentrated under reduced pressure to afford 4-[6-[(2-methylpyrazol-3-yl)methoxy]-3,4-dihydro-1*H*-isoquinolin-2-yl]piperidin-3-ol, **74** (45 mg, crude, HCl salt, trans) as white solid which was used in next step reaction without purification.

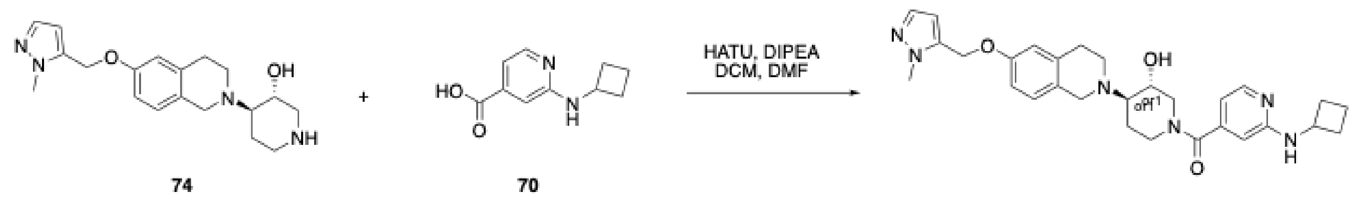

[2-(cyclobutylamino)-4-pyridyl]-[3-hydroxy-4-[6-[(2-methylpyrazol-3-yl)methoxy]-3,4-dihydro-1*H*-isoquinolin-2-yl]-1-piperidyl]methanone

A mixture of **70** (14.5 mg, 0.06 mmol, HCl salt, *trans*), HATU (31.6 mg, 0.08 mmol), DIPEA (0.3 mmol, 50 uL) in DCM (4 mL) was stirred at 20 °C for 30 minutes. Then a solution of **74** (20.0 mg, 0.05 mmol, HCl salt) in DMF (1 mL) was added and the mixture was stirred for 12 h at 20 °C. The mixture was concentrated under reduced pressure to remove DCM and the residue was preparative HPLC (Instrument: Gilson GX-281 Liquid Handler, Gilson 322 Pump, Gilson 156 UV Detector; Column: Waters Xbridge 150 × 25 mm × 5 µm; Mobile phase A: H_2O with 0.05% NH_3-H_2O (v%); Mobile phase B: MeCN; Gradient: B from 23% to 53% in 9.5 min, hold 100% B for 2 min; Flow Rate: 25 mL/min; Column Temperature: 30 °C; Wavelength: 220 nm, 254 nm) to afford [2-(cyclobutylamino)-4-pyridyl]-[3-hydroxy-4-[6-[(2-methylpyrazol-3-yl)methoxy]-3,4-dihydro-1*H*-isoquinolin-2-yl]-1-piperidyl]methanone (15 mg, 55%, *trans*, 2.1 mg was delivered to Evotec) as light-yellow solid. ^{1}H NMR (400 MHz, methanol-*d*) *δ* ppm 8.00 (t, *J* = 5.7 Hz, 1H), 7.40 (d, *J* = 1.8 Hz, 1H), 6.99 (d, *J* = 8.3 Hz, 1H), 6.75 - 6.82 (m, 2H), 6.49 - 6.56 (m, 1H), 6.44 (d, *J* = 8.8 Hz, 1H), 6.36 (d, *J* = 2.2 Hz, 1H), 5.10 (s, 2H), 4.50 - 4.73 (m, 2H), 4.27 (br s, 1H), 3.85 - 3.92 (m, 3H), 3.67 - 3.85 (m, 3H), 3.07 - 3.16 (m, 1H), 2.93 - 3.03 (m, 2H), 2.83 - 2.93 (m, 3H), 2.68 - 2.80 (m, 1H), 2.39 (br s, 2H), 1.86 - 2.05 (m, 3H), 1.79 (d, *J* = 7.9 Hz, 2H), 1.51 - 1.68 (m, 1H); LCMS (ESI) $[M+H]^+$ m/z: calcd 517.3, found 517.0; HPLC: 94.54%@254nm; racemic.

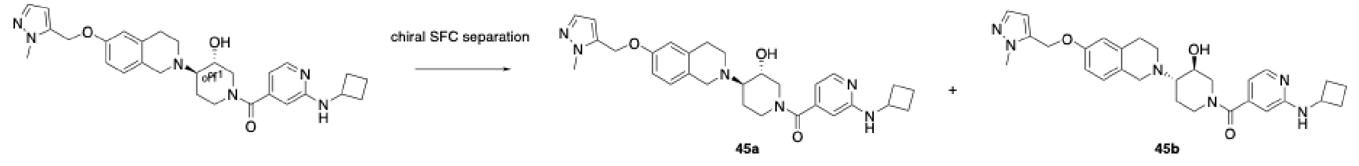

(2-(cyclobutylamino)pyridin-4-yl)((3*R*,4*R*)-3-hydroxy-4-(6-((1-methyl-1*H*-pyrazol-5-yl)methoxy)-3,4-dihydroisoquinolin-2(1*H*)-yl)piperidin-1-yl)methanone (**45a**) and (2-(cyclobutylamino)pyridin-4-yl)((3*S*,4*S*)-3-hydroxy-4-(6-((1-methyl-1*H*-pyrazol-5-yl)methoxy)-3,4-dihydroisoquinolin-2(1*H*)-yl)piperidin-1-yl)methanone (**45b**). [2-(cyclobutylamino)-4-pyridyl]-[3-hydroxy-4-[6-[(2-methylpyrazol-3-yl)methoxy]-3,4-dihydro-1*H*-isoquinolin-2-yl]-1-piperidyl]methanone (10.0 mg, *trans*) was separated by chiral SFC (Instrument: Berger, MULTIGR AM-II; Column: DAICEL CHIRALPAK AS-H 250 × 30 mm I.D. 5 µm; Mobile phase: supercritical CO_2/EtOH (0.1% $NH_3 \cdot H_2O$, v%) = 60/40; Flow Rate: 50 mL/min; Column Temperature: 38 °C; Nozzle Pressure: 100 bar; Nozzle Temperature: 60 °C; Evaporator Temperature: 20 °C; Trimmer Temperature: 25 °C; Wavelength: 220 nm) to afford **45a** and **45b**.

45a (peak 2, retention time: 4.516 minutes): (4.4 mg, light-yellow solid). ^{1}H NMR (400 MHz, methanol-*d₄*) *δ* 7.97 - 8.03 (m, 1H), 7.42 (d, *J* = 1.9 Hz, 1H), 7.02 (d, *J* = 8.1 Hz, 1H), 6.79 - 6.86 (m, 2H), 6.47 - 6.60 (m, 2H), 6.38 (d, *J* = 1.9 Hz, 1H), 5.13 (s, 2H), 4.74 (br d, J = 12.9 Hz, 1H), 4.62 (s, 1H), 4.36 (br s, 1H), 4.21 - 4.30 (m, 1H), 4.14 (br s, 1H), 3.85 - 3.93 (m, 5H), 3.79 (br d, J = 13.9 Hz, 1H), 3.25 (br d, J = 13.4 Hz, 1H), 3.14 (br t, J = 11.9 Hz, 1H), 3.03 (br d, J = 5.1 Hz, 2H), 2.89 - 2.96 (m, 2H), 2.79 - 2.88 (m, 1H), 2.68 (br s, 1H), 2.37 - 2.47 (m, 2H), 1.87 - 2.04 (m, 4H), 1.74 - 1.85 (m, 2H); LCMS (ESI) [M+H]+ m/z: calcd 517.3, found 517.2; HPLC: 100%@254nm; 99.9%ee. **45b** (peak 1, retention time: 4.280 minutes): (2.9 mg, light-yellow solid). ^{1}H NMR (400 MHz, methanol-*d₄*) *δ* 8.02 (t, J = 5.6 Hz, 1H), 7.42 (d, J = 1.9 Hz, 1H), 7.02 (d, J = 8.4 Hz, 1H), 6.79 - 6.86 (m, 2H), 6.54 (dd, J = 8.2, 5.7 Hz, 1H), 6.46 (d, J = 8.5 Hz, 1H), 6.38 (d, J = 1.9 Hz, 1H), 5.13 (s, 2H), 4.72 (br d, J = 9.3 Hz, 1H), 4.62 (s, 1H), 4.58 (br d, J = 14.0 Hz, 1H), 4.28 (br t, J = 6.6 Hz, 1H), 3.87 - 3.99 (m, 5H), 3.70 - 3.86 (m, 2H), 2.98 - 3.19 (m, 2H), 2.94 (br s, 3H), 2.83 (br s, 1H), 2.70 - 2.79 (m, 1H), 2.43 (br d, J = 4.5 Hz, 2H), 1.89 - 2.09 (m, 3H), 1.76 - 1.86 (m, 2H), 1.57 - 1.74 (m, 1H); LCMS (ESI) [M+H]+ m/z: calcd 517.3, found 517.2; HPLC: 100%@254nm; 99.3%ee.

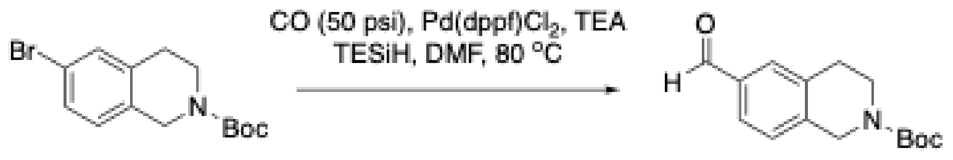

76

tert-butyl 6-formyl-3,4-dihydroisoquinoline-2(1*H*)-carboxylate (**76**)
To a mixture of *tert*-butyl 6-bromo-3,4-dihydro-1*H*-isoquinoline-2-carboxylate (2.8 g, 8.97 mmol) in DMF (15 mL) were added Pd(dppf)Cl_2 (1.2 g, 1.64 mmol), TES (5.2 mL, 32.6 mmol) and TEA (5.5 mL, 39.5 mmol). The mixture was degassed and backfilled with Ar 3 times, then repeated with CO 3 times, and stirred for 24 h at 80 °C under CO (50 psi). The mixture was diluted with EtOAc (100 mL), filtered, and the filtrate was washed with brine (50 mL x 5), dried over anhydrous Na_2SO_4, filtered, and concentrated under reduced pressure. The residue was purified by flash chromatography (ISCO®; 40 g AgelaFlash® Silica Flash Column, petroleum ether/EtOAc with EtOAc from 0~20%, flow rate = 40 mL/min) to afford *tert*-butyl 6-formyl-3,4-dihydro-1*H*-isoquinoline-2-carboxylate, **76** (1.84 g, 79 % yield) as light-yellow solid. ^{1}H NMR (400 MHz, MeOH-d_4) δ 9.93 (s, 1H), 7.71 - 7.75 (m, 2H), 7.35 (d, J = 7.8 Hz, 1H), 4.65 (br s, 2H), 3.68 (t, J = 5.7 Hz, 2H), 2.93 (t, J = 5.9 Hz, 2H), 1.50 (s, 9H); LCMS (ESI) [M+H-56]$^+$m/z: calcd 206.1; found 206.0.

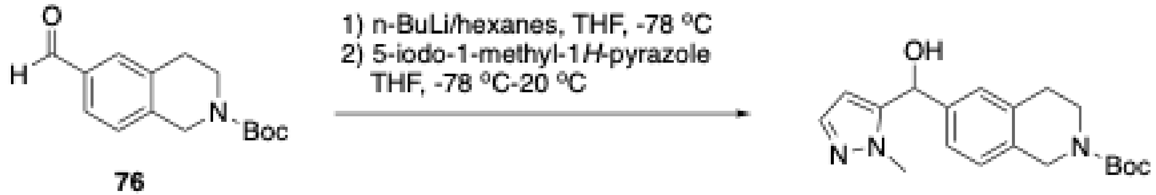

76 **77**

tert-butyl 6-(hydroxy(1-methyl-1*H*-pyrazol-5-yl)methyl)-3,4-dihydroisoquinoline-2(1*H*)-carboxylate (**77**)
A mixture of 5-iodo-1-methyl-pyrazole (2.20 g, 10.6 mmol) in THF (6 mL) was degassed under vacuum and purged with N_2 several times. n-BuLi (9 mL, 22.5 mmol, 2.5 M in hexane) was added dropwise into the mixture under -78 °C. When the addition was complete, the mixture was stirred at -78 °C for 30 minutes. A solution of **76** (2.3 g, 8.80 mmol) in THF (6 mL) was added into the mixture under -78 °C and the mixture was allowed to warm to 20 °C and stir for 12 h. The reaction mixture was quenched by addition of saturated aqueous solution of NH_4Cl (10 mL), and then extracted with EtOAc (15 mL x 3). The combined organic layers were washed dried over anhydrous Na_2SO_4, filtered, and concentrated under reduced pressure to give a residue which was purified with flash chromatography (ISCO®; 20 g AgelaFlash® Silica Flash Column, petroleum ether/EtOAc with EtOAc from 0~50%, flow rate = 35 mL/min) to afford *tert*-butyl 6-[hydroxy-(2-methylpyrazol-3-yl)methyl]-3,4-dihydro-1*H*-isoquinoline-2-carboxylate, **77** (2.8 g, 93 % yield) as yellow oil. ^{1}H NMR (400 MHz, MeOH-d_4) δ ppmppm 7.34 (d, J = 1.9 Hz, 1H), 7.18 - 7.22 (m, 2H), 7.12 - 7.16 (m, 1H), 6.03 (d, J = 1.8 Hz, 1H), 5.88 (s, 1H), 4.56 (br s, 2H), 3.77 (s, 3H), 3.60 - 3.68 (m, 2H), 2.83 (t, J = 5.9 Hz, 2H), 1.49 (s, 9H); LCMS (ESI) [M+H]+m/z: calcd 344.2, found 344.2.

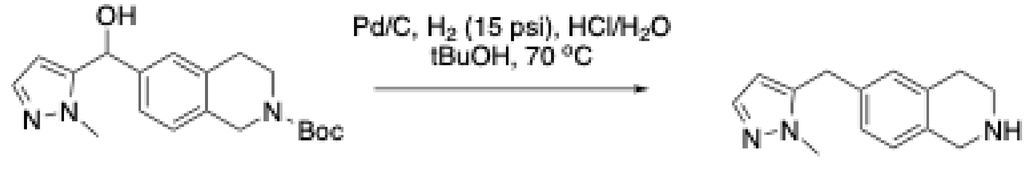

77 **78**

6-((1-methyl-1*H*-pyrazol-5-yl)methyl)-1,2,3,4-tetrahydroisoquinoline (**78**)
To a mixture of **77** (2.8 g, 8.15 mmol) in t-BuOH (8 mL) were added Pd/C (200 mg, 10 % wt of Pd with 50 % wt of H_2O) and HCl/H_2O (1 mL, 38%wt). The suspension was degassed under vacuum and purged with H_2 several times. The mixture was stirred under H_2 (~15 psi) at 70 °C for 48 h. The reaction mixture was filtered and the filtrate was concentrated under reduced pressure to afford a crude product which was diluted with H_2O (10 mL),

adjusted to pH = 8 with saturated aqueous solution of $NaHCO_3$. The aqueous solution was concentrated under reduced pressure and the residue was triturated with DCM/MeOH (20 mL, v/v = 10:1). After stirring at 20 °C for 30 minutes, the solid was removed by filtration and the filtrate was concentrated under reduced pressure to afford 6-[(2-methylpyrazol-3-yl)methyl]-1,2,3,4-tetrahydroisoquinoline, **78** (700 mg, crude) as light-yellow oil.
^{1}H NMR (400 MHz, MeOH-d_4) δ ppm 7.35 (d, J = 1.9 Hz, 1H), 6.97 - 7.01 (m, 1H), 6.91 - 6.96 (m, 2H), 6.04 (d, J = 1.9 Hz, 1H), 3.98 (s, 2H), 3.93 (s, 2H), 3.68 (s, 3H), 3.07 (t, J = 6.0 Hz, 2H), 2.79 (t, J = 5.9 Hz, 2H); LCMS (ESI) $[M+H]^+$ m/z: calcd 228.1, found 228.1.

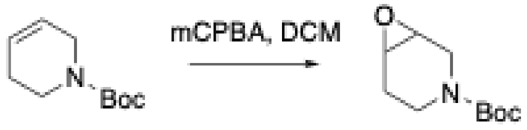

75

tert-butyl 7-oxa-3-azabicyclo[4.1.0]heptane-3-carboxylate, **75**
To a solution of *tert*-butyl 3,6-dihydro-2*H*-pyridine-1-carboxylate (2 g, 10.9 mmol) in DCM (10 mL) was added 3-chlorobenzenecarboperoxoic acid (2.60 g, 12.1 mmol, 80 % wt) at 0 °C. The mixture was stirred for 16 h at 20 °C. The reaction mixture was filtered, and the filtrate was concentrated under reduced pressure. The residue was purified with flash chromatography (ISCO®; 20 g AgelaFlash® Silica Flash Column, petroleum ether/EtOAc with EtOAc from 0~30 %, flow rate = 35 mL /min) to afford *tert*-butyl 7-oxa-4-azabicyclo[4.1.0]heptane-4-carboxylate, **75** (1.68 g, 77% yield) as colorless oil. ^{1}H NMR (400 MHz, MeOH-d_4) δ 3.76 (br d, J = 17.1 Hz, 2H), 3.15 - 3.30 (m, 4H), 1.98 (br s, 1H), 1.84 - 1.93 (m, 1H), 1.45 (s, 9H).

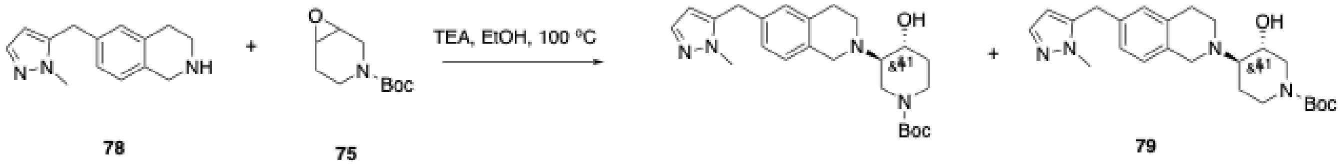

78 75 79

rac-tert-butyl (3*R*,4*R*)-3-hydroxy-4-(6-((1-methyl-1*H*-pyrazol-5-yl)methyl)-3,4-dihydroisoquinolin-2(1*H*)-yl)piperidine-1-carboxylate (**79**)
To a mixture of **75** (1.1 g, 5.52 mmol) and **78** (700 mg, 3.08 mmol) in EtOH (10 mL) was added TEA (1.4 mL, 10.0 mmol). The mixture was stirred at 100 °C for 12 h. The reaction mixture was concentrated under reduced pressure and the residue was purified with preparative HPLC (Instrument: Gilson GX-281 Liquid Handler, Gilson 322 Pump, Gilson 156 UV Detector; YMC-Triart Prep C18 150 × 40 mm × 7 µm; Mobile phase A: H2O with 0.04% NH_3-H_2O (v%)+10 mM NH_4HCO_4; Mobile phase B: MeCN; Gradient: B from 50% to 50% in 10 min, hold 100% B for 4 min; Flow Rate: 60 mL/min; Column Temperature: 30 °C; Wavelength: 220 nm, 254 nm) to afford *rac-tert*-butyl (3*R*,4*R*)-4-hydroxy-3-(6-((1-methyl-1*H*-pyrazol-5-yl)methyl)-3,4-dihydroisoquinolin-2(1*H*)-yl)piperidine-1-carboxylate (540 mg, 43%, racemic, trans, light-yellow oil). ^{1}H NMR (400 MHz, MeOH-d_4) δ ppm 7.35 (d, J = 2.0 Hz, 1H), 6.98 - 7.02 (m, 1H), 6.90 - 6.95 (m, 2H), 6.04 (d, J = 1.8 Hz, 1H), 4.12 (br s, 1H), 3.84 - 4.02 (m, 7H), 3.68 (s, 3H), 3.08 (br s, 1H), 2.81 - 2.93 (m, 5H), 2.46 (td, J = 9.9, 3.9 Hz, 1H), 1.95 - 2.03 (m, 1H), 1.46 (s, 9H); LCMS (ESI) [M+H]+m/z: calcd 427.3, found 427.2 and *rac-tert*-butyl (3*R*,4*R*)-3-hydroxy-4-(6-((1-methyl-1*H*-pyrazol-5-yl)methyl)-3,4-dihydroisoquinolin-2(1*H*)-yl)piperidine-1-carboxylate, **79** (210 mg, 16 % yield, racemic, trans, light-yellow oil). ^{1}H NMR (400 MHz, MeOH-d_4) δ ppm 7.35 (d, J = 2.0 Hz, 1H), 7.00 (d, J = 8.3 Hz, 1H), 6.90 - 6.94 (m, 2H), 6.03 (d, J = 2.0 Hz, 1H), 4.22 (br d, J = 10.0 Hz, 1H), 4.10 (br d, J = 12.8 Hz, 1H), 3.97 (s, 2H), 3.87 - 3.93 (m, 1H), 3.78 - 3.84 (m, 1H), 3.67 (s, 3H), 3.62 (dt, J = 9.7, 5.0 Hz, 1H), 2.95 - 3.01 (m, 1H), 2.77 - 2.87 (m, 4H), 2.55 - 2.66 (m, 2H), 1.87 (br dd, J = 13.3, 3.3 Hz, 1H), 1.48 - 1.56 (m, 2H), 1.47 (s, 9H);LCMS (ESI) [M+H]+m/z: calcd 427.3; found 427.2.

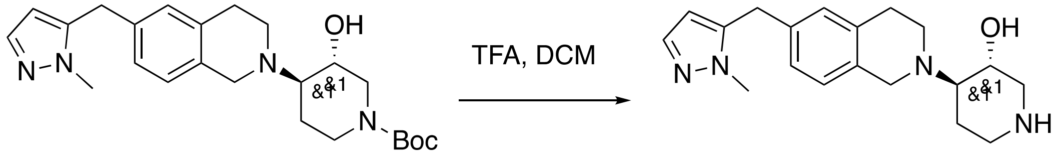

rac-(3*R*,4*R*)-4-(6-((1-methyl-1*H*-pyrazol-5-yl)methyl)-3,4-dihydroisoquinolin-2(1*H*)-yl)piperidin-3-ol
To a mixture of *tert*-butyl 3-hydroxy-4-[6-[(2-methylpyrazol-3-yl)methyl]-3,4-dihydro-1*H*-isoquinolin-2-yl]piperidine-1-carboxylate (60 mg, 0.141 mmol) in DCM (3 mL) was added TFA (160 µL, 2.08 mmol,). The mixture was stirred at 20 °C for 12 h. The mixture was diluted with H_2O (8 mL), adjusted to pH = 8 with saturated aqueous solution of $NaHCO_3$, and then extracted with DCM (10 mL x 2). The combined organic layers were dried over anhydrous Na_2SO_4, filtered, and concentrated under reduced pressure to afford 4-[6-[(2-methylpyrazol-3-yl)methyl]-3,4-dihydro-1*H*-isoquinolin-2-yl]piperidin-3-ol (40 mg, crude) as light-yellow oil which was used into next step directly.

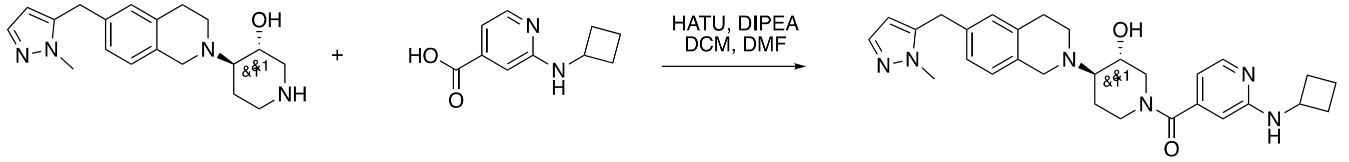

rac-(2-(cyclobutylamino)pyridin-4-yl)((3*R*,4*R*)-3-hydroxy-4-(6-((1-methyl-1*H*-pyrazol-5-yl)methyl)-3,4-dihydroisoquinolin-2(1*H*)-yl)piperidin-1-yl)methanone
A mixture of 2-(cyclobutylamino)pyridine-4-carboxylic acid (40 mg, 0.175 mmol, HCl salt), HATU (70 mg, 0.184 mmol), DIPEA (100 µL, 0.574 mmol) in DCM (3 mL) and DMF (1 mL) was stirred at 20 °C for 30 minutes. Then 4-[6-[(2-methylpyrazol-3-yl)methyl]-3,4-dihydro-1*H*-isoquinolin-2-yl]piperidin-3-ol (40 mg, 0.123 mmol) was added and the mixture was stirred for 12 h at 20 °C. The reaction mixture was concentrated under reduced pressure and the residue was purified with preparative HPLC (Instrument: Gilson GX-281 Liquid Handler, Gilson 322 Pump, Gilson 156 UV Detector; Column: Waters Xbridge BEH C18 100 × 25 mm × 5 µm; Mobile phase A: H_2O with 0.05% NH_3-H_2O (v%); Mobile phase B: MeCN; Gradient: B from 40% to 80% in 7.8 min, hold 100% B for 2 min; Flow Rate: 25 mL/min; Column Temperature: 30 °C; Wavelength: 220 nm, 254 nm) to afford [2-(cyclobutylamino)-4-pyridyl]-[3-hydroxy-4-[6-[(2-methylpyrazol-3-yl)methyl]-3,4-dihydro-1*H*-isoquinolin-2-yl]-1-piperidyl]methanone (40 mg, 65% yield) as white solid which was used into next step directly.

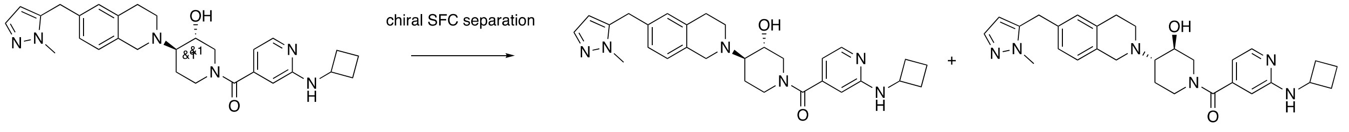

(2-(cyclobutylamino)pyridin-4-yl)((3*R*,4*R*)-3-hydroxy-4-(6-((1-methyl-1*H*-pyrazol-5-yl)methyl)-3,4-dihydroisoquinolin-2(1*H*)-yl)piperidin-1-yl)methanone (**46a**) and [2-(cyclobutylamino)-4-pyridyl]-[(3S,4S)-3-hydroxy-4-[6-[(2-methylpyrazol-3-yl)methyl]-3,4-dihydro-1*H*-isoquinolin-2-yl]-1-piperidyl]methanone (**46b**). [2-(cyclobutylamino)-4-pyridyl]-[3-hydroxy-4-[6-[(2-methylpyrazol-3-yl)methyl]-3,4-dihydro-1*H*-isoquinolin-2-yl]-1-piperidyl]methanone (40 mg, 0.080 mmol) was separated by chiral SFC (Instrument: Thar 80; Column: Daicel Chiralpak AD 250 × 30 mm I.D. 10 µm; Mobile phase: supercritical CO_2/EtOH (0.1% NH_3-H_2O, v%) = 60/40; Flow Rate: 80 mL/min; Column Temperature: 38 °C; Nozzle Pressure: 100 bar; Nozzle Temperature: 60 °C; Evaporator Temperature: 20 °C; Trimmer Temperature: 25 °C; Wavelength: 220 nm) to afford **46a,** [2-(cyclobutylamino)-4-pyridyl]-[(3R,4R)-3-hydroxy-4-[6-[(2-methylpyrazol-3-yl)methyl]-3,4-dihydro-1*H*-isoquinolin-2-yl]-1-piperidyl]methanone (15 mg, 38% yield, peak 1, retention time = 3.753 min, white solid). ^{1}H NMR (400 MHz, MeOH-*d₄*) δ ppm 8.00 (t, *J* = 5.6 Hz, 1H), 7.35 (d, *J* = 1.9 Hz, 1H), 6.98 - 7.03 (m, 1H), 6.89 - 6.95 (m, 2H), 6.52 (dd, *J* = 8.0, 5.6 Hz, 1H), 6.44 (d, *J* = 8.5 Hz, 1H), 6.03 (d, *J* = 1.9 Hz, 1H), 4.69 (br dd, *J* = 12.4, 3.7 Hz, 1H), 4.52 - 4.71 (m, 1H), 3.98 (s, 2H), 3.88 (q, *J* = 14.8 Hz, 2H), 3.68 - 3.81 (m, 2H), 3.67 (s, 3H), 2.68 - 3.16 (m, 2H), 2.91 - 3.04 (m, 2H), 2.81 - 2.90 (m, 3H), 2.35 - 2.45 (m, 2H), 1.84 - 2.05 (m, 3H), 1.74 - 1.82 (m, 2H), 1.51 - 1.73 (m, 1H); LCMS (ESI) $[M+H]^+$ m/z: calcd 501.3, found 501.2; HPLC: 100%@254nm; 99.7%ee and **46b**, [2-(cyclobutylamino)-4-pyridyl]-[(3S,4S)-3-hydroxy-4-[6-[(2-methylpyrazol-3-yl)methyl]-3,4-dihydro-1*H*-isoquinolin-2-yl]-1-piperidyl]methanone (15 mg, 38% yield, retention time = 4.259 min, white solid). ^{1}H NMR (400 MHz, MeOH-*d_4*) δ ppm 8.00 (t, *J* = 5.6 Hz, 1H), 7.35 (d, *J* = 1.9 Hz, 1H), 6.99 - 7.03 (m, 1H), 6.90 - 6.95 (m, 2H), 6.52 (dd, *J* = 7.9, 5.4 Hz, 1H), 6.44 (d, *J* = 8.6 Hz, 1H), 6.03 (d, *J* = 1.9 Hz, 1H), 4.50 - 4.73 (m, 1H), 4.21 - 4.30 (m, 1H), 3.98 (s, 2H), 3.82 - 3.95 (m, 2H), 3.69 - 3.81 (m, 2H), 3.67 (s, 3H), 2.68 - 3.16 (m, 2H), 2.90 - 3.05 (m, 2H), 2.83 - 2.90 (m, 3H), 2.36 - 2.45 (m, 2H), 1.84 - 2.04 (m, 3H), 1.72 - 1.83 (m, 2H), 1.53 - 1.71 (m, 1H); LCMS (ESI) $[M+H]^+$ m/z: calcd 501.3, found 501.2; HPLC: 99.32%@254nm; 99.9%ee.

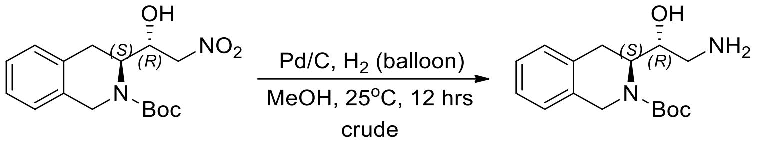

tert-butyl (3*S*)-3-[(1*R*)-2-amino-1-hydroxy-ethyl]-3,4-dihydro-1*H*-isoquinoline-2-carboxylate
To a solution of *tert*-butyl (3*S*)-3-[(1*R*)-1-hydroxy-2-nitro-ethyl]-3,4-dihydro-1*H*-isoquinoline-2-carboxylate (200 mg, 620.43 μmol, 1 eq) in MeOH (10 mL) was added Pd/C (20 mg, 10% of Pd with 50% of water, wt%). The mixture was degassed and backfilled with N_2 three times and then repeated with H_2 three times. The reaction mixture was stirred for 24 h at 25 °C under H_2 (balloon). The resulting mixture was filtered through celite, washed with MeOH (10 mL x 2), and concentrated under reduced pressure to afford *tert*-butyl (3*S*)-3-[(1*R*)-2-amino-1-hydroxy-ethyl]-3,4-dihydro-1*H*-isoquinoline-2-carboxylate (crude) as a yellow oil which was used in next step directly. LCMS ($M+H^+$) m/z: calcd 293.2; found 293.0.

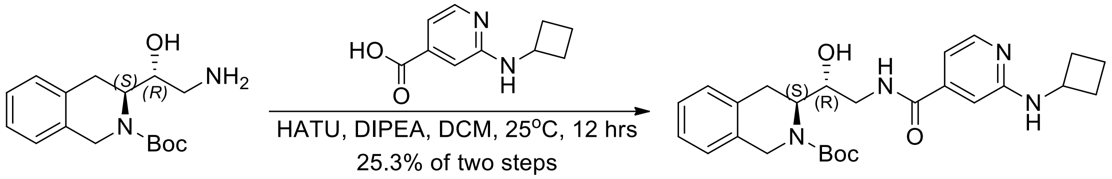

tert-butyl (3*S*)-3-[(1*R*)-2-[[2-(cyclobutylamino)pyridine-4-carbonyl]amino]-1-hydroxy-ethyl]-3,4-dihydro-1*H*-isoquinoline-2-carboxylate
To a mixture of *tert*-butyl (3*S*)-3-[(1*R*)-2-amino-1-hydroxy-ethyl]-3,4-dihydro-1*H*-isoquinoline-2-carboxylate (620.43 μmol, 1.2 eq) in DCM (10 mL) was added HATU (237 mg, 623.31 umol, 1.21 eq) and DIPEA (200 mg, 1.55 mmol, 3.00 eq). After stirring 15 minutes, 2-(cyclobutylamino)pyridine-4-carboxylic acid (118 mg, 516.02 umol, 1 eq, HCl) was added and the mixture was stirred for 12 h at 25 °C. The solvent was removed under reduced pressure and the residue was diluted with EtOAc (50 mL), washed with saturated NH_4Cl aqueous solution (30 mL x 2), brine (30 mL), dried over anhydrous Na_2SO_4, filtered and concentrated in vacuum. The residue was purified by flash chromatography (ISCO®; 40 g of Agela flash silica column, petroleum ether/EtOAc with EtOAc from 0~75 %, flow rate: 40 mL/min) to afford *tert*-butyl (3*S*)-3-[(1*R*)-2-[[2-(cyclobutylamino)pyridine-4-carbonyl]amino]-1-hydroxy-ethyl]-3,4-dihydro-1*H*-isoquinoline-2-carboxylate (65 mg, 25 % yield) as a yellow gum. ^1H NMR (400 MHz, MeOH-d_4) δ ppm 8.47 (br s, 1 H), 7.58 - 7.80 (m, 5 H), 7.22 - 7.41 (m, 2 H), 5.25 - 5.38 (m, 1 H), 4.66 - 4.97 (m, 2 H), 4.15 - 4.33 (m, 1 H), 4.07 (br s, 1 H), 3.70 (m, 1 H), 3.52 - 3.64 (m, 1 H), 3.44 (dd, J = 15.8, 5.0 Hz, 1 H), 2.90 (br s, 2 H), 2.34 - 2.48 (m, 2 H), 2.20 - 2.31 (m, 2 H), 1.93 - 2.07 (m, 9 H); LCMS (ESI) $[M+H]^+$ calcd 467.3, found 467.4.

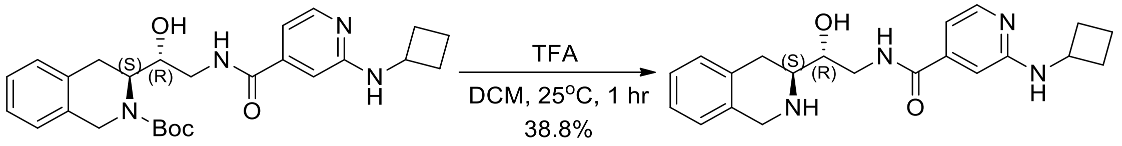

47

2-(cyclobutylamino)-N-[(2*R*)-2-hydroxy-2-[(3*S*)-1,2,3,4-tetrahydroisoquinolin-3-yl]ethyl]pyridine-4-carboxamide., **47**. To a solution of *tert*-butyl (3*S*)-3-[(1*R*)-2-[[2-(cyclobutylamino)pyridine-4-carbonyl]amino]-1-hydroxy-ethyl]-3,4-dihydro-1*H*-isoquinoline-2-carboxylate (65 mg, 139.31 umol, 1 eq) in DCM (3 mL) was added TFA (0.5 mL, 6.75 mmol, 48.47 eq). The mixture was stirred for 1 h at 25 °C. The solvent was removed under reduced pressure and the residue was purified by preparative HPLC (Instrument: Gilson GX-281 Liquid Handler, Gilson 322 Pump, Gilson 156 UV Detector; Column: Waters Xbridge 150 × 25 mm × 5 μm; Mobile phase A: H_2O with 0.05% NH_3-H_2O (v%); Mobile phase B: MeCN; Gradient: B from 40% to 70% in 6.5 min, hold 100% B for 2.5 min; Flow Rate: 25 mL/min; Column Temperature: 30 °C; Wavelength: 220 nm, 254 nm) to afford 2-(cyclobutylamino)-N-[(2*R*)-2-hydroxy-2-[(3*S*)-1,2,3,4-tetrahydroisoquinolin-3-yl]ethyl]pyridine-4-carboxamide, **47** (20 mg, 39% yield) as a white solid. ^1H NMR (400 MHz, MeOH-d_4) δ ppm 8.00 (dd, J = 5.3, 0.7 Hz, 1 H), 7.08 - 7.16 (m, 3 H), 7.01 - 7.07 (m, 1 H), 6.84 (dd, J = 5.3, 1.5 Hz, 1 H), 6.82 (d, J = 0.7 Hz, 1 H), 4.25 (quin, J = 7.9 Hz, 1 H), 3.96 - 4.09 (m, 2 H), 3.88 (dt, J = 6.9, 4.8 Hz, 1 H), 3.60 - 3.67 (m, 1 H), 3.48 - 3.58 (m, 1 H), 2.81 - 3.01 (m, 3 H), 2.33 - 2.47 (m, 2 H), 1.86

- 1.99 (m, 2 H), 1.70 - 1.83 (m, 2 H); LCMS (ESI) $[M+H]^+$ calcd 367.4, found 367.4; HPLC, 99.08% @254 nM; 52.6% ee.

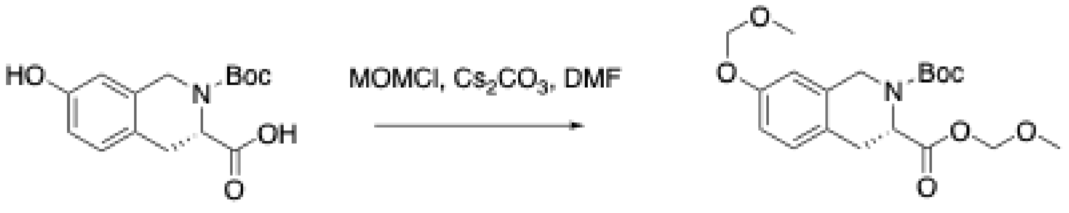

62

2-(*tert*-butyl) 3-(methoxymethyl) (*S*)-7-(methoxymethoxy)-3,4-dihydroisoquinoline-2,3(1*H*)-dicarboxylate.
62 (25 g, 85.23 mmol) and cesium carbonate (83.31 g, 255.70 mmol) were mixed in DMF (250 mL). Methoxymethyl chloride (20.59 g, 255.70 mmol) was added dropwise while stirring. The mixture was stirred at 20 °C for 10 h. After the completion of the reaction, MTBE (1 L) was added and the mixture was extracted with H_2O (5 x 300 mL). The organic phase was separated, dried with Na_2SO_4 and evaporated in vacuo at 35 °C to give (*S*)-2-*tert*-butyl 3-(methoxymethyl) 7-(methoxymethoxy)-3,4-dihydroisoquinoline-2,3(*1H*)-dicarboxylate (31 g, 81.28 mmol, 95% yield) which was used without further purification on the next step. ^1H NMR (500 MHz, $CDCl_3$) *δ* (ppm) 1.47 (s, 9H), 3.20 (s, 2H), 3.24 (d, 2H), 3.38 (t, 1H), 3.45 (s, 3H), 3.51 (s, 3H), 5.12 (s, 2H), 5.27 (s, 2H), 6.79 (s, 1H), 6.84 (d, 1H), 7.05 (d, 1H). LCMS(ESI): $[M\text{-}Boc]^+$ m/z: calcd 281.4; found 282.2; Rt = 1.48 min.

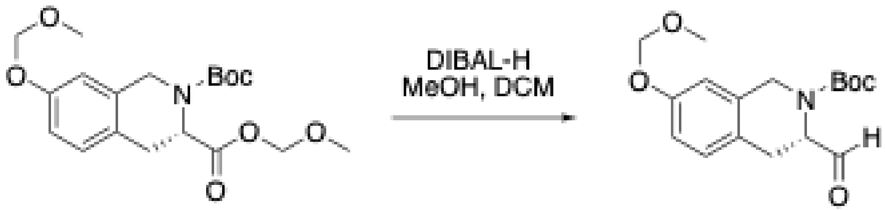

63

tert-butyl (*S*)-3-formyl-7-(methoxymethoxy)-3,4-dihydroisoquinoline-2(1*H*)-carboxylate (**63)**
(*S*)-2-*tert*-butyl 3-(Methoxymethyl) 7-(methoxymethoxy)-3,4-dihydroisoquinoline-2,3(1*H*)-dicarboxylate (31 g, 81.28 mmol) was dissolved in DCM (1000 mL) and cooled to -78 °C. DIBAL-H (23.12 g, 162.55 mmol, 32.98 mL) was added dropwise at the same temperature while stirring. The mixture was stirred at -78 °C for 1 h followed by the dropwise addition of the solution of methanol (26.04 g, 812.75 mmol, 32.92 mL) in DCM (100 mL). The mixture was warmed to room temperature and carefully poured into a vigorously stirred aqueous solution of citric acid. After 15 min of vigorous stirring the organic layer was separated, dried with Na_2SO_4 and evaporated in vacuo at 35 °C to give crude *tert*-butyl (*3S*)-3-formyl-7-(methoxymethoxy)-3,4-dihydro-1*H*-isoquinoline-2-carboxylate, **63** (26 g, 80.90 mmol, 100% yield) which was used as is in the next step. ^1H NMR (500 MHz, $CDCl_3$) δ (ppm) 1.46 (m, 9H), 3.03 (m, 2H), 3.48 (s, 3H), 4.82 (m, 3H), 5.13 (s, 2H), 6.79 (m, 2H), 7.07 (d, 1H), 9.49 (d, 1H). LCMS(ESI): $[M\text{-}Boc]^+$ m/z: calcd 221.4; found 222.2; Rt = 1.38 min.

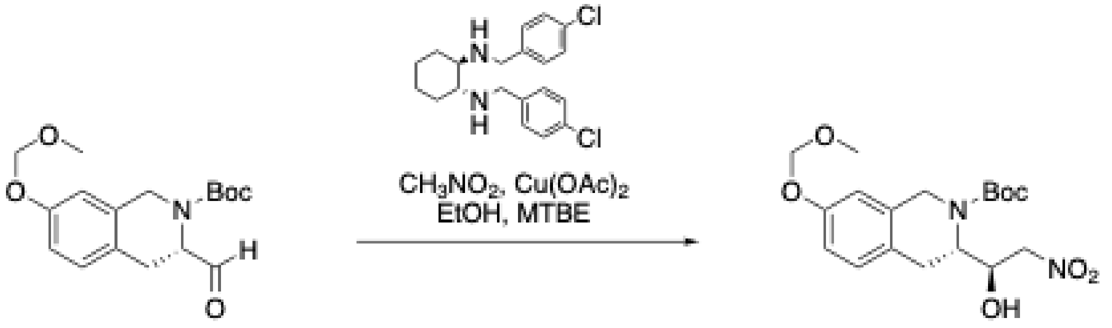

63

tert-butyl (*S*)-3-((*R*)-1-hydroxy-2-nitroethyl)-7-(methoxymethoxy)-3,4-dihydroisoquinoline-2(1*H*)-carboxylate
(*1R*,*2R*)-N^1,N^2-Bis[(4-chlorophenyl)methyl]cyclohexane-1,2-diamine (3.66 g, 10.08 mmol) and copper(II) acetate hydrate (1.68 g, 8.40 mmol, 892.22 uL) were mixed together in ethanol (150 mL) and stirred for 15 min at 20 °C. The mixture was cooled to 0 °C and a solution of **63** (27 g, 84.02 mmol) in ethanol (150 mL) was added. Nitromethane (102.57 g, 1.68 mol, 90.77 mL) was then added in one portion. The mixture was stirred at 20 °C for 10 h. The solvent was evaporated in vacuo at 35 °C. The residue was dissolved in MTBE (500 mL) and the mixture was extracted with aqueous NH_3 (3*100 mL) and aqueous citric acid (3 x 100 mL). The organic phase was separated, dried over Na_2SO_4 and evaporated in vacuo at 35 °C. The crude product was purified by column chromatography (Interchim, 330g SiO_2, petroleum ether/MTBE with MTBE from 0~30%,flow rate = 127 mL/min, Rt = 35min) to give *tert*-butyl (*3S*)-3-[(*1R*)-1-hydroxy-2-nitro-ethyl]-7-(methoxymethoxy)-3,4-dihydro-1*H*-isoquinoline-2-carboxylate (10 g, 26.15 mmol, 31% yield). ^{1}H NMR (400 MHz, $CDCl_3$) *δ* (ppm) 1.50 (s, 9H), 2.92 (d, 1H), 3.15 (m, 1H), 3.26 (m, 1H), 3.48 (s, 3H), 4.08 (m, 1H), 4.26 (m, 2H), 4.44 (m, 2H), 4.82 (m, 1H), 5.16 (s, 2H), 6.83 (s, 1H), 6.91 (d, 1H), 7.09 (d, 1H). LCMS(ESI): [M-Boc]$^+$ m/z: calcd 282.4; found 283.2; Rt = 1.38 min.

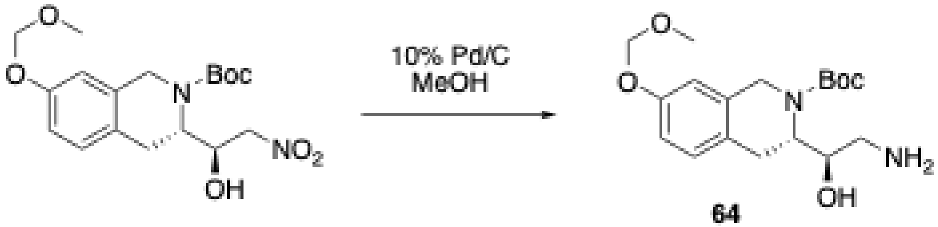

64

tert-butyl (*S*)-3-((*R*)-2-amino-1-hydroxyethyl)-7-(methoxymethoxy)-3,4-dihydroisoquinoline-2(1*H*)-carboxylate (**64**)
tert-Butyl (*3S*)-3-[(*1R*)-1-hydroxy-2-nitro-ethyl]-7-(methoxymethoxy)-3,4-dihydro-1*H*-isoquinoline-2-carboxylate (9 g, 23.54 mmol) was dissolved in methanol (500 mL) and palladium, 10% on carbon (1 g, 2.09 mmol) was added. The mixture was hydrogenated in autoclave at 50 °C, 50 atm (H_2) for 10 h. The catalyst was filtered off and the solvent was removed in vacuo at 35 °C to give *tert*-butyl (*3S*)-3-[(*1R*)-2-amino-1-hydroxy-ethyl]-7-(methoxymethoxy)-3,4-dihydro-1*H*-isoquinoline-2-carboxylate, **64** (8.2 g, 23.27 mmol, 99% yield). ^{1}H NMR (400 MHz, DMSO-*d*$_6$) *δ* (ppm) 1.42 (s, 9H), 2.39 (m, 1H), 2.71 (m, 1H), 3.03 (m, 1H), 3.16 (m, 2H), 3.36 (s, 3H), 4.09 (m, 2H), 4.70 (m, 1H), 5.14 (s,2H), 6.82 (m, 2H), 7.06 (d, 1H), OH and NH_2 are not observed. LCMS(ESI): [M+H]$^+$ m/z: calcd 352.4; found 353.2; Rt = 1.07 min.

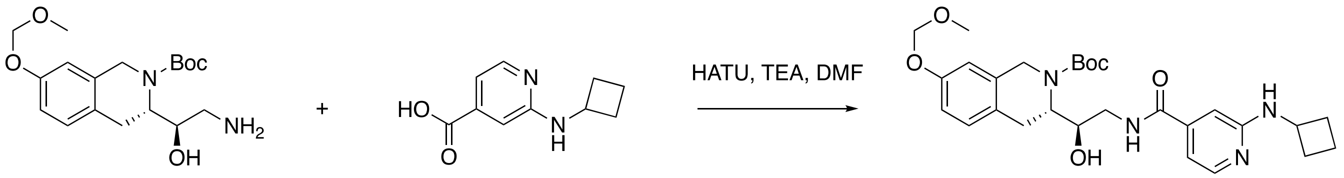

tert-butyl (*S*)-3-((*R*)-2-(2-(cyclobutylamino)isonicotinamido)-1-hydroxyethyl)-7-(methoxymethoxy)-3,4-dihydroisoquinoline-2(1*H*)-carboxylate
2-(Cyclobutylamino)pyridine-4-carboxylic acid (272.70 mg, 1.42 mmol) and TEA (1.44 g, 14.19 mmol, 1.98 mL) were mixed together in DMF (10 mL) and cooled to 0 °C. HATU (809.17 mg, 2.13 mmol) was added and the mixture was stirred for 15 min at 0 °C followed by the addition of the *tert*-butyl (3*S*)-3-[(1*R*)-2-amino-1-hydroxy-ethyl]-7-(methoxymethoxy)-3,4-dihydro-1*H*-isoquinoline-2-carboxylate (0.5 g, 1.42 mmol). The resulting mixture

was warmed to room temperature and stirred overnight. After all starting material was consumed, as was shown by LCMS, 10 mL of ethyl acetate was added and the organic phase was washed with brine three times. The organic layer was dried over Na_2SO_4, filtered, and concentrated in vacuo at 45 °C to give crude product which was purified by HPLC (40-55% water-acetonitrile, 2-10 min, flow: 30mL/min (loading pump 4mL/min acetonitrile) column: SunFire C18 100 x 19 mm) to give *tert*-butyl (*3S*)-3-[(*1R*)-2-[[2-(cyclobutylamino)pyridine-4-carbonyl]amino]-1-hydroxy-ethyl]-7-(methoxymethoxy)-3,4-dihydro-1*H*-isoquinoline-2-carboxylate (0.406 g, 54% yield). ^{1}H NMR (400 MHz, DMSO-*d*$_6$) *δ* (ppm) 1.40 (s, 9H), 1.44 (m, 2H), 1.66 (m, 2H), 2.25 (m, 2H), 2.77 (m, 1H), 3.02 (m, 2H), 3.32 (s, 3H), 3.46 (m, 1H), 3.60 (m, 1H), 4.16 (m, 3H), 4.77 (m, 1H), 5.15 (m, 3H), 6.73 (s, 1H), 6.80 (d, 1H), 6.85 (m, 2H), 6.91 (d, 1H), 7.08 (d, 1H), 7.99 (m, 1H), 8.36 (m, 1H). LCMS(ESI): $[M+H]^+$ m/z: calcd 526.6; found 527.4; Rt = 1.21 min.

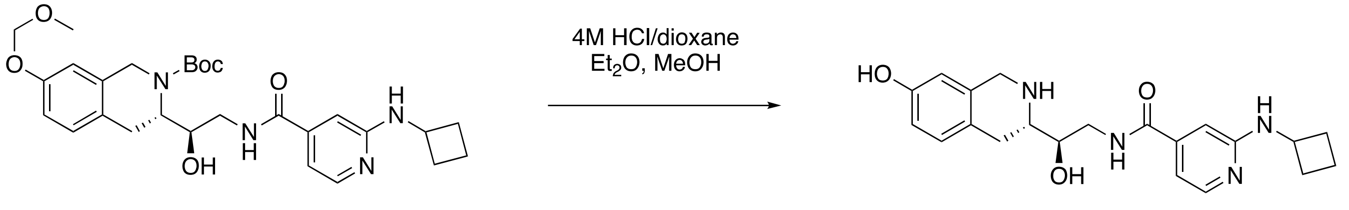

2-(cyclobutylamino)-*N*-((*R*)-2-hydroxy-2-((*S*)-7-hydroxy-1,2,3,4-tetrahydroisoquinolin-3-yl)ethyl)isonicotinamide
Hydrogen chloride solution 4.0M in dioxane (2.11 g, 57.82 mmol, 2.64 mL) was added to the solution of the *tert*-butyl (*3S*)-3-[(*1R*)-2-[[2-(cyclobutylamino)pyridine-4-carbonyl]amino]-1-hydroxy-ethyl]-7-(methoxymethoxy)-3,4-dihydro-1*H*-isoquinoline-2-carboxylate (0.406 g, 770.95 umol) in the mixture of Et_2O (4 mL) and MeOH (2 mL). The resulting mixture was stirred for 24 h at 20 °C. The formed solid was filtered off, washed with Et_2O (4 mL), and dried in vacuo at 35 °C to give 2-(cyclobutylamino)-*N*-[(*2R*)-2-hydroxy-2-[(*3S*)-7-hydroxy-1,2,3,4-tetrahydroisoquinolin-3-yl]ethyl]pyridine-4-carboxamide (0.302 g, 663.19 umol, 86.02% yield, 2HCl). ^{1}H NMR (500 MHz, DMSO-*d*$_6$) *δ* (ppm) 1.75 (m, 3H), 1.99 (m, 2H), 2.41 (m, 2H), 2.97 (m, 2H), 3.39 (m, 2H), 4.12 (m, 4H), 6.59 (m, 1H), 6.70 (s, 1H), 6.72 (d, 1H), 7.19 (m, 3H), 8.01 (d, 1H), 8.88 (m, 1H), 9.14 (m, 1H), 6.42 (m, 1H), 9.61 (m, 1H). LCMS(ESI): $[M+H]^+$ m/z: calcd 382.2; found 383.2; Rt = 0.67 min.

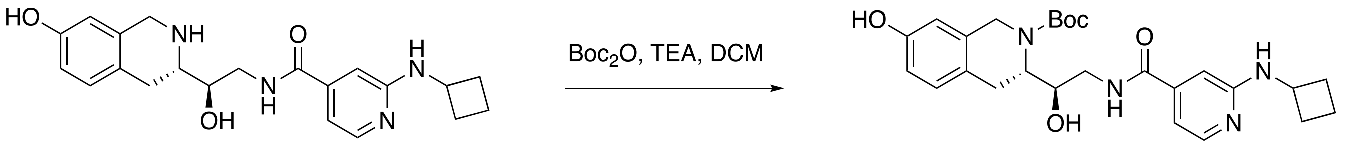

tert-butyl (*S*)-3-((*R*)-2-(2-(cyclobutylamino)isonicotinamido)-1-hydroxyethyl)-7-hydroxy-3,4-dihydroisoquinoline-2(1*H*)-carboxylate
Sodium hydrogen carbonate, 99% (169.35 mg, 2.02 mmol) was added in one portion to the solution of the 2-(cyclobutylamino)-*N*-[(*2R*)-2-hydroxy-2-[(*3S*)-7-hydroxy-1,2,3,4-tetrahydroisoquinolin-3-yl]ethyl]pyridine-4-carboxamide (0.306 g, 671.97 umol, 2HCl) in a mixture of water (8 mL) and THF (8 mL). The resulting mixture was stirred for 5 min at room temperature followed by the dropwise addition of the solution of di-*tert*-butyl dicarbonate (146.66 mg, 671.97 umol) in THF (2 mL). The reaction mixture was stirred overnight at room temperature. Ethyl acetate (15mL) was added to the reaction mixture. The organic phase was separated and washed with brine, dried over Na_2SO_4, filtered, and evaporated in vacuo at 40 °C to give *tert*-butyl (*3S*)-3-[(*1R*)-2-[[2-(cyclobutylamino)pyridine-4-carbonyl]amino]-1-hydroxy-ethyl]-7-hydroxy-3,4-dihydro-1*H*-isoquinoline-2-carboxylate (0.27 g, 559.50 umol, 83% yield) which was used in the next step without purification. ^{1}H NMR (500 MHz, DMSO-*d*$_6$) *δ* (ppm) 1.46 (m, 12H), 1.51 (m, 2H), 1.69 (m, 2H), 2.27 (m, 2H), 2.72 (m, 1H), 2.98 (m, 2H), 3.49 (m, 2H), 4.14 (m, 2H), 4.72 (m, 1H), 5.09 (m, 1H), 6.56 (m, 1H), 6.61 (s, 1H), 6.76 (d, 1H), 6.80 (d, 1H), 6.96 (d, 1H), 8.02 (s, 1H), 8.32 (m, 1H), 9.15 (s, 1H). LCMS(ESI): $[M+H]^+$ m/z: calcd 482.6; found 483.4; Rt = 1.03 min.

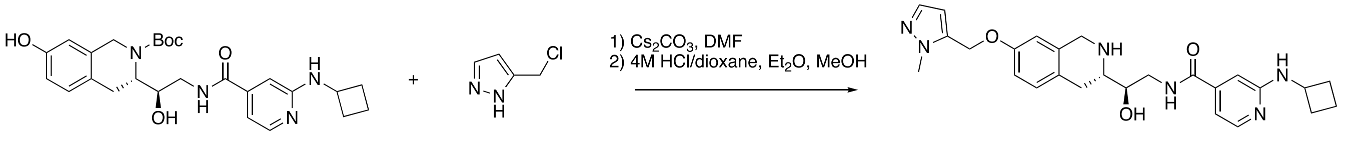

48

2-(cyclobutylamino)-*N*-((*R*)-2-hydroxy-2-((*S*)-7-((1-methyl-1*H*-pyrazol-5-yl)methoxy)-1,2,3,4-tetrahydroisoquinolin-3-yl)ethyl)isonicotinamide (**48**). (*S*)-*tert*-Butyl 3-((*R*)-2-(2-(cyclobutylamino)isonicotinamido)-1-hydroxyethyl)-7-hydroxy-3,4-dihydroisoquinoline-2(1*H*)-carboxylate (103.61 umol), 5-(chloromethyl)-1-methyl-1*H*-pyrazole (19.04 mg, 113.97 μmol, HCl) and cesium carbonate (101.28 mg, 310.84 μmol) were mixed together in DMF (2 mL) and stirred at 50 °C overnight. The reaction mixture was diluted with water end extracted three times with EtOAc. The organic phase was washed with brine, dried over Na_2SO_4, filtered on and evaporated at 40 °C to give *tert*-butyl (*S*)-3-((*R*)-2-(2-(cyclobutylamino)isonicotinamido)-1-hydroxyethyl)-7-((1-methyl-1*H*-pyrazol-5-yl)methoxy)-3,4-dihydroisoquinoline-2(1*H*)-carboxylate (0.048 g, 80% yield) which was used in the next step without further purification.^{1}H NMR (400 MHz, DMSO-d_6) δ (ppm) 1.40 (m, 9H), 1.65 (m, 4H), 1.84 (m, 2H), 2.24 (m, 2H), 2.77 (m, 2H), 3.46 (m, 2H), 3.81 (s, 3H), 4.03 (m, 1H), 4.23 (m, 2H), 4.81 (m, 1H), 5.11 (m, 3H), 6.36 (s, 1H), 6.72 (d, 1H), 6.88 (m, 2H), 7.07 (d, 1H), 7.34 (s, 1H), 7.99 (d, 1H), 8.33 (m, 1H). LCMS(ESI): [M+H]$^+$ m/z: calcd 576.3; found 577.2; Rt = 1.19 min. Hydrogen chloride solution 4.0M in dioxane (227.61 mg, 6.24 mmol, 284.51 μL) was added to the solution of the *tert*-butyl (*3S*)-3-[(*1R*)-2-[[2-(cyclobutylamino)pyridine-4-carbonyl]amino]-1-hydroxy-ethyl]-7-[(2-methylpyrazol-3-yl)methoxy]-3,4-dihydro-1*H*-isoquinoline-2-carboxylate (0.048 g, 83.23 μmol) in the mixture of Et_2O (1 mL) and MeOH (0.5 mL). The resulting mixture was stirred for 24 h at 20 °C. The formed solid was filtered, washed with Et_2O (1 mL) and dried in vacuo at 35 °C to give crude product which was purified by HPLC (15-40% water-acetonitrile+NH_3, 10min, flow30mL/min (loading pump 4mL/min acetonitrile) , column: SUNFIRE C18 100 x 19mm to give 2-(cyclobutylamino)-*N*-[(*2R*)-2-hydroxy-2-[(*3S*)-7-[(2-methylpyrazol-3-yl)methoxy]-1,2,3,4-tetrahydroisoquinolin-3-yl]ethyl]pyridine-4-carboxamide **48** (0.0166 g, 34.83 umol, 42% yield).^{1}H NMR($CDCl_3$, 400 MHz): δ (ppm) 1.83 (m, 7H), 2.44 (m, 2H), 2.80 (m, 2H), 3.04 (m, 2H), 3.89 (s, 3H), 4.02 (s, 2H), 4.17 (m, 2H), 4.88 (m, 1H), 4.98 (s, 2H), 6.28 (s, 1H), 6.61 (d, 1H), 6.69 (s, 1H), 6.74 (t, 2H), 7.06 (m, 2H), 7.42 (s, 1H), 8.11 (d, 1H). LCMS(ESI): [M+2H]$^+$ m/z: calcd 476.2; found 478.2; Rt = 0.85 min.

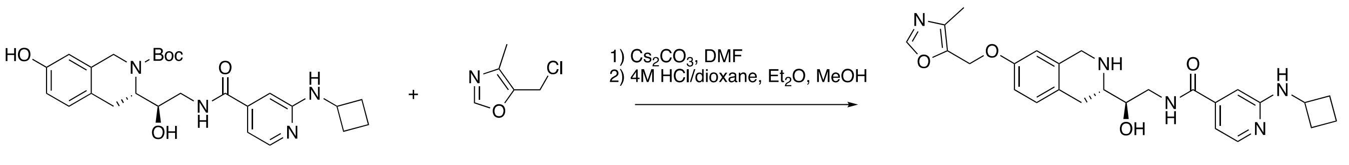

49

2-(cyclobutylamino)-*N*-((*R*)-2-hydroxy-2-((*S*)-7-((4-methyloxazol-5-yl)methoxy)-1,2,3,4-tetrahydroisoquinolin-3-yl)ethyl)isonicotinamide (**49**). *tert*-Butyl (3*S*)-3-[(1*R*)-2-[[2-(cyclobutylamino)pyridine-4-carbonyl]amino]-1-hydroxy-ethyl]-7-hydroxy-3,4-dihydro-1*H*-isoquinoline-2-carboxylate (0.06 g, 124.33 umol), 5-(chloromethyl)-4-methyl-oxazole (22.98 mg, 136.77 umol, HCl) and cesium carbonate (121.53 mg, 373.00 umol) were mixed together in DMF (2 mL) and stirred at 50 °C overnight. The reaction mixture was diluted with water and extracted three times with EtOAc. The organic phase was washed with brine, dried over Na2SO4, filtered and evaporated at 40 °C to give *tert*-butyl (3*S*)-3-[(1*R*)-2-[[2-(cyclobutylamino)pyridine-4-carbonyl]amino]-1-hydroxy-ethyl]-7-[(4-methyloxazol-5-yl)methoxy]-3,4-dihydro-1*H*-isoquinoline-2-carboxylate (0.045 g, 77.90 umol, 63% yield) which was used in the next step without further purification. ^{1}H NMR (400 MHz, DMSO-d_6) δ (ppm) 1.41 (m, 9H), 1.66 (m, 2H), 1.84 (m, 2H), 2.15 (s, 3H), 2.86 (m, 2H), 3.02 (m, 2H), 4.08 (m, 4H), 4.76 (m, 1H), 5.08 (m, 2H), 6.73 (s, 1H), 6.78 (m, 2H), 6.88 (m, 2H), 7.06 (d, 1H), 7.99 (m, 1H). LCMS(ESI): [M+H]+ m/z: calcd 577.6; found 578.4; Rt = 3.15 min. 4.0M hydrogen chloride solution in dioxane (213.02 mg, 5.84 mmol, 266.28 μL) was added to the solution of the *tert*-butyl (3*S*)-3-[(1*R*)-2-[[2-(cyclobutylamino)pyridine-4-carbonyl]amino]-1-hydroxy-ethyl]-7-[(4-methyloxazol-5-yl)methoxy]-3,4-dihydro-1*H*-isoquinoline-2-carboxylate (0.045 g, 77.90 μmol) in the mixture of Et_2O (1 mL) and MeOH (0.5 mL). The resulting mixture was stirred for 24 h at 20 °C. The formed solid was filtered off, washed with Et_2O (1 mL), and dried in vacuo at 35 °C to give crude product which was purified by HPLC (50-70% water-methanol+NH3, 10min, flow30mL/min (loading pump 5mL/min methano+NH3) , column: YMC ACTUS TRIART C18 100 x 20mm to give 2-(cyclobutylamino)-N-[(2*R*)-2-hydroxy-2-[(3*S*)-7-[(4-methyloxazol-5-yl)methoxy]-1,2,3,4-tetrahydroisoquinolin-3-yl]ethyl]-pyridine-4-carboxamide **49** (0.0179 g, 37.48 umol, 48% yield).

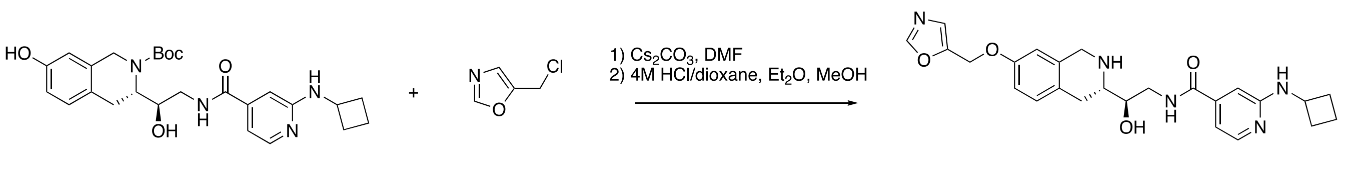

50

2-(cyclobutylamino)-*N*-((*R*)-2-hydroxy-2-((*S*)-7-(oxazol-5-ylmethoxy)-1,2,3,4-tetrahydroisoquinolin-3-yl)ethyl)isonicotinamide (**50**). *tert*-Butyl (3*S*)-3-[(1*R*)-2-[[2-(cyclobutylamino)pyridine-4-carbonyl]amino]-1-hydroxy-ethyl]-7-hydroxy-3,4-dihydro-1*H*-isoquinoline-2-carboxylate (0.06 g, 124.33 µmol), 5-(chloromethyl)oxazole (21.06 mg, 136.77 µmol, HCl) and cesium carbonate (121.53 mg, 373.00 µmol) were mixed together in DMF (2 mL) and stirred at 50 °C overnight. The reaction mixture was diluted with water end extracted three times with EtOAc. The organic phase was washed three times with brine, dried over Na_2SO_4, filtered off and evaporated at 40 °C to give *tert*-butyl (3*S*)-3-[(1*R*)-2-[[2-(cyclobutylamino)pyridine-4-carbonyl]amino]-1-hydroxy-ethyl]-7-(oxazol-5-ylmethoxy)-3,4-dihydro-1*H*-isoquinoline-2-carboxylate (0.052 g, 92.26 µmol, 74% yield) which was used in the next step without further purification. ^{1}H NMR (400 MHz, DMSO-d_6) δ (ppm) 1.41 (m, 9H), 1.67 (m, 3H), 1.87 (m, 2H), 2.25 (m, 3H), 3.04 (m, 2H), 3.51 (m, 2H), 4.13 (m, 4H), 5.13 (s, 2H), 6.73 (s, 1H), 6.84 (m, 4H), 7.32 (s, 1H), 7.99 (d, 1H), 8.40 (m, 1H). LCMS(ESI): $[M+H]^+$ m/z: calcd 563.6; found 564.4; Rt = 2.86 min. Hydrogen chloride solution 4.0M in dioxane (252.28 mg, 6.92 mmol, 315.35 µL) was added to the solution of the *tert*-butyl (3*S*)-3-[(*1R*)-2-[[2-(cyclobutylamino)pyridine-4-carbonyl]amino]-1-hydroxy-ethyl]-7-(oxazol-5-ylmethoxy)-3,4-dihydro-1*H*-isoquinoline-2-carboxylate (0.052 g, 92.26 umol) in the mixture of Et_2O (1 mL) and MeOH (0.5 mL). The resulting mixture was stirred for 24 h at 20 °C. The formed solid was filtered on, washed with Et_2O (1 mL) and dried in vacuo at 35 °C to give crude product which was purified by HPLC (40-55% water-acetonitrile+NH_3, 10min, flow30mL/min (loading pump 4mL/min acetonitrile), column: YMC-Actus Triart c18 to give 2-(cyclobutylamino)-*N*-[(*2R*)-2-hydroxy-2-[(*3S*)-7-(oxazol-5-ylmethoxy)-1,2,3,4-tetrahydroisoquinolin-3-yl]ethyl]pyridine-4-carboxamide, **50** (13.30 mg, 28.69 umol, 31% yield). ^{1}H NMR (400 MHz, DMSO-d_6) δ 1.67 (m, 2H), 1.86 (m, 2H), 2.26 (m, 2H), 2.43 (m, 1H), 2.59 (m, 2H), 2.69 (m, 2H), 3.26 (m, 1H), 3.52 (m, 1H), 3.64 (m, 1H), 3.88 (AB-system, 2H), 4.27 (m, 1H), 4.94 (d, 1H), 5.11 (s, 2H), 6.70 (s, 1H), 6.76 (s, 1H), 6.80 (d, 1H), 6.96 (d, 1H), 7.01 (d, 1H), 7.30 (s, 1H), 8.02 (d, 1H), 8.39 (s, 1H), 8.51 (t, 1H). LCMS(ESI): $[M+H]^+$ m/z: calcd 463.2; found 464.2; Rt = 0.77 min.

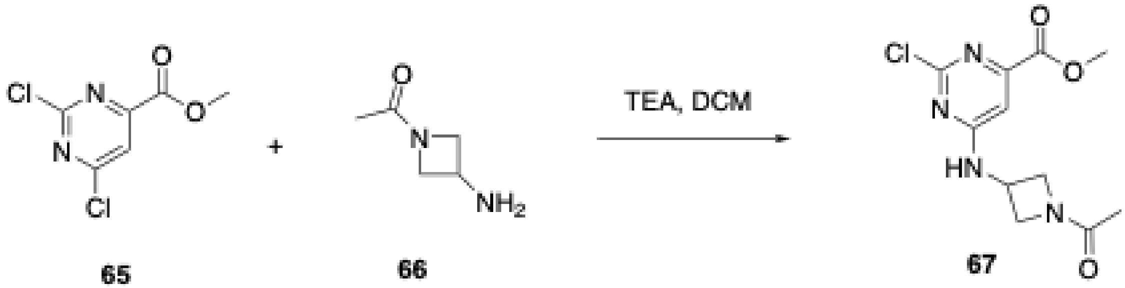

methyl 6-((3-acetylcyclobutyl)amino)-2-chloropyrimidine-4-carboxylate, **67**
To a solution of **65** (3 g, 14.49 mmol) in DCM (50 mL) at 0 °C was added TEA (2.93 g, 28.98 mmol, 4.04 mL) followed by **66** (3.31 g, 14.49 mmol, CF_3CO_2H) and the resulting reaction mixture was stirred at 0 °C for 30 min and allowed to warm to room temperature. After 12 h the reaction mixture was triturated with water (30 mL). The layers were separated and the aqueous layer was extracted with DCM (30 mL). The combined organic layers were washed with water (25 mL) and brine, dried over Na_2SO_4 and concentrated in vacuo to give methyl 6-[(1-acetylazetidin-3-yl)amino]-2-chloro-pyrimidine-4-carboxylate, **67** (2.95 g, crude).^{1}H NMR (500 MHz, DMSO-d_6) δ (ppm) 1.75 (s, 3H), 3.85 (s, 3H), 3.93 (m, 2H), 4.17 (m, 1H), 4.56 (m, 2H), 7.07 (s, 1H), 8.97 (bds, 1H). LCMS(ESI): $[M\text{-}Boc]^+$ m/z: calcd 284.7; found 286.0; Rt = 0.817 min

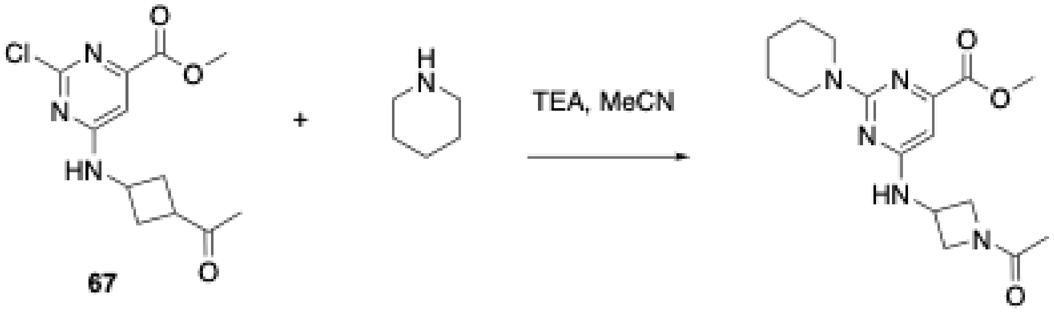

methyl 6-((3-acetylcyclobutyl)amino)-2-(piperidin-1-yl)pyrimidine-4-carboxylate
To a solution of **67** (1.5 g, 5.27 mmol) in ACN (20 mL) at room temperature was added TEA (586.46 mg, 5.80 mmol, 807.79 µL) followed by piperidine (493.48 mg, 5.80 mmol, 572.48 µL) and the resulting reaction mixture was stirred at 80 °C for 32 h, then it was diluted with water (40 mL). The aqueous layer was extracted with DCM (40 mL x 2). The combined organic layers were washed with water (15 mL) and brine, dried over Na_2SO_4 and

concentrated in vacuo to give methyl 6-[(1-acetylazetidin-3-yl)amino]-2-(1-piperidyl)pyrimidine-4-carboxylate (1.1 g, crude). ^{1}H NMR (500 MHz, DMSO-d_6) δ (ppm) 1.49 (m, 6H), 1.75 (s, 3H), 3.58 (m, 3H), 3.68 (m, 5H), 3.95 (m, 1H), 4.12 (m, 1H), 4.39 (m, 1H), 4.53 (m, 1H), 6.53 (s, 1H), 8.31 (bds, 1H). LCMS (ESI): [M-Boc]$^+$ m/z: calcd 333.4; found 334.2; Rt = 0.951 min.

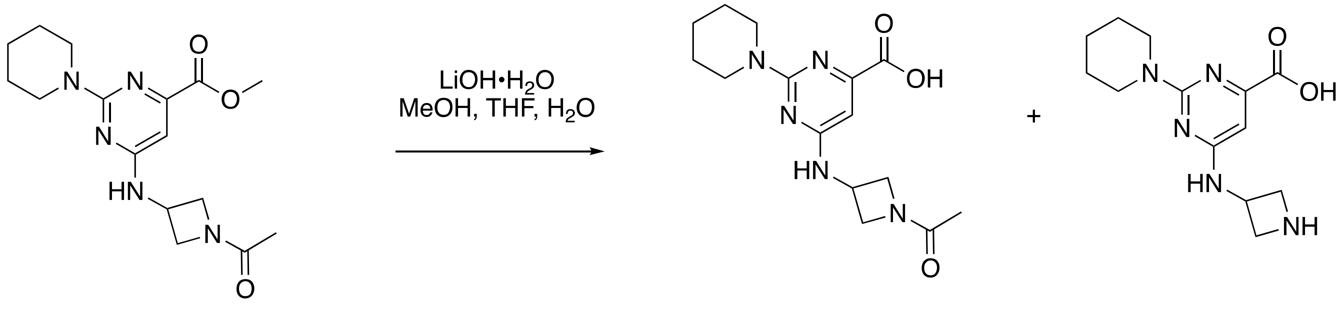

6-((1-acetylazetidin-3-yl)amino)-2-(piperidin-1-yl)pyrimidine-4-carboxylic acid
A mixture of methyl 6-[(1-acetylazetidin-3-yl)amino]-2-(1-piperidyl)pyrimidine-4-carboxylate (1.1 g, 3.30 mmol) and lithium hydroxide, hydrate (304.58 mg, 7.26 mmol) in THF (10 mL) - methanol (10 mL) - water (15 mL) was stirred at room temperature for 12 h. Then the volatile organic solvents were rotoevaporated. The aqueous phase was washed with DCM (10 mL), then acidified ($NaHSO_4$, monohydrate) to pH 5 and the mixture was concentrated in vacuo. The residue was suspended in hot ethanol (100 mL) and filtered. The filtercake was washed with hot ethanol (2 x 50 mL) and discarded. The filtrate was evaporated in vacuo to leave the residue 6-(azetidin-3-ylamino)-2-(piperidin-1-yl)pyrimidine-4-carboxylic acid (1 g, crude, contains 37% of deacylated byproduct).
^{1}H NMR (400 MHz, DMSO-d_6) δ (ppm) 1.60 (m, 6H), 3.69 (m, 4H), 4.18 (m, 2H), 4.39 (m, 1H), 4.53 (m, 1H), 4.78 (m, 1H), 6.32 (s, 1H), 8.02 (bds, 1H), 8.73 (bds, 1H), 10.45 (bds, 1H). LCMS(ESI): [M-Boc]+ m/z: calcd 277.3; found 278.2; Rt = 0.644 min.

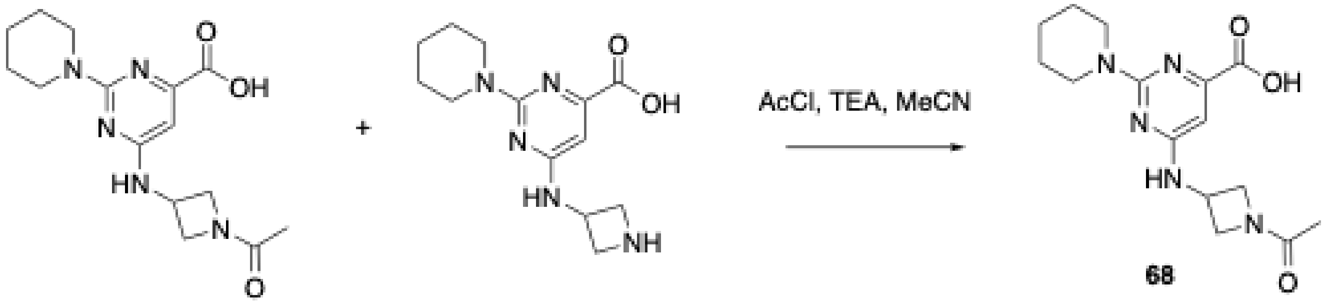

6-((1-acetylazetidin-3-yl)amino)-2-(piperidin-1-yl)pyrimidine-4-carboxylic acid (**68**)
To a solution of the crude material from previous step which contained 6-(azetidin-3-ylamino)-2-(1-piperidyl)pyrimidine-4-carboxylic acid (1 g, 1.33 mmol) as impurity (37% by LCMS) and TEA (54.00 mg, 533.68 umol, 74.38 uL) in ACN (25 mL) was added acetyl chloride (41.89 mg, 533.68 umol, 32.47 uL) dropwise at 0 °C. The reaction mixture was then stirred at room temperature. After 24 hr 70% conversion was observed. TEA (54.00 mg, 533.68 umol, 74.38 uL) and acetyl chloride (41.89 mg, 533.68 umol, 32.47 uL) was added again and the reaction mixture was then stirred at 35 °C another 24 h. After full consumption of the starting material (according to LCMS) the reaction mixture was concentrated in vacuo. Then Na_2CO_3 (15 mL, 5% aqueous solution) was added and the aqueous phase was washed with DCM (2 x 10 mL), then it was acidified ($NaHSO_4$, monohydrate) to pH 5 and the mixture was concentrated in vacuo. The residue was suspended in hot ethanol (100 mL) and filtered. The filtercake was washed with hot ethanol (2 x 50 mL) and discarded. The filtrate was evaporated in vacuo to provide the product 6-[(1-acetylazetidin-3-yl)amino]-2-(1-piperidyl)pyrimidine-4-carboxylic acid, **68** (0.68 g, crude). ^{1}H NMR (400 MHz, DMSO-d_6) δ (ppm) 1.47 (m, 4H), 1.56 (m, 2H), 1.75 (s, 3H), 3.62 (m,

4H), 3.74 (m, 1H), 3.97 (m, 1H), 4.10 (m, 1H), 4.38 (m, 1H), 4.51 (m, 1H), 6.32 (s, 1H), 7.85 (bds, 1H), 11.45 (bds, 1H). LCMS(ESI): [M-Boc]+ m/z: calcd 319.4; found 320.2; Rt = 0.866 min.

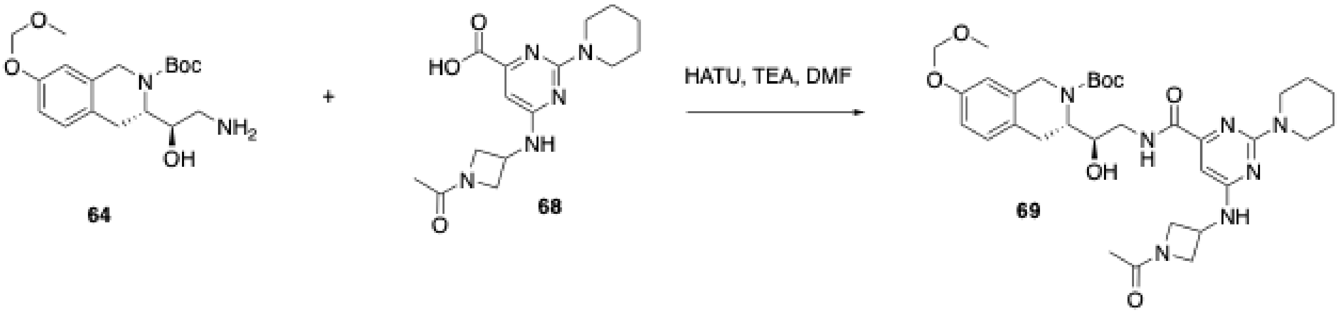

tert-butyl (*S*)-3-((*R*)-2-(6-((1-acetylazetidin-3-yl)amino)-2-(piperidin-1-yl)pyrimidine-4-carboxamido)-1-hydroxyethyl)-7-(methoxymethoxy)-3,4-dihydroisoquinoline-2(1*H*)-carboxylate (**69)**

68 (151.45 mg, 425.62 umol, HCl) and TEA (430.69 mg, 4.26 mmol, 593.23 µL) were dissolved in DMF (3 mL) and cooled to 0 °C, HATU (242.75 mg, 638.44 µmol) was added and the mixture was stirred for 15 min at 0 °C. **64** (0.15 g, 425.62 µmol) was added and the mixture was warmed to room temperature and stirred overnight. Ethyl acetate (10mL) was added and organic phase was washed with brine three times. Organic phase was dried over Na_2SO_4, filtered, and concentrated in vacuo at 45 °C to give crude product which was purified by HPLC (45-60 % water-acetonitrile, 2 - 10min, flow: 30 mL / min (loading pump 4 mL / min acetonitrile) column: SunFire C18 100 x 19 mm) to give *tert*-butyl (3*S*)-3-[(1*R*)-2-[[6-[(1-acetylazetidin-3-yl)amino]-2-(1-piperidyl)pyrimidine-4-carbonyl]amino]-1-hydroxy-ethyl]-7-(methoxymethoxy)-3,4-dihydro-1*H*-isoquinoline-2-carboxylate, **69** (0.048 g, 73.42 µmol, 17v% yield). ^{1}H NMR ($CDCl_3$, 400 MHz) *δ*: 1.49 (m, 11H), 1.62 (m, 3H), 1.69 (s, 3H), 2.83 (m, 1H), 2.96 (m, 1H), 3.11 (m, 1H), 3.43 (s, 3H), 3.69 (m, 2H), 3.79 (m, 4H), 3.99 (m, 3H), 4.21 (m, 1H), 4.31 (m, 4H), 4.65 (m, 2H), 5.10 (s, 2H), 5.84 (m, 1H), 6.46 (s, 1H), 6.76 (s, 1H), 6.85 (d, 1H), 7.06 (d, 1H), 9.12 (m, 1H). LCMS(ESI): [M+H]+ m/z: calcd 653.3; found 654.4; Rt = 1.46 min.

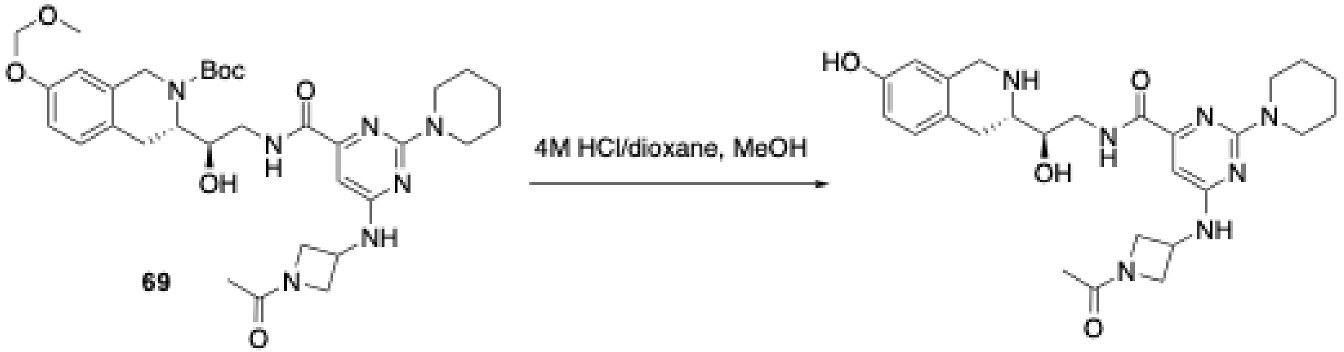

6-((1-acetylazetidin-3-yl)amino)-*N*-((*R*)-2-hydroxy-2-((*S*)-7-hydroxy-1,2,3,4-tetrahydroisoquinolin-3-yl)ethyl)-2-(piperidin-1-yl)pyrimidine-4-carboxamide

69 (0.048 g, 73.42 µmol) was dissolved in a mixture of MeOH (2 mL). Hydrogen chloride solution 4.0M in dioxane (200.77 mg, 5.51 mmol, 250.97 µL) was added. The mixture was stirred for 12 h at 20 °C . Solvent was removed in vacuo at 35 °C to give 6-[(1-acetylazetidin-3-yl)amino]-N-[(2*R*)-2-hydroxy-2-[(3*S*)-7-hydroxy-1,2,3,4-tetrahydroisoquinolin-3-yl]ethyl]-2-(1-piperidyl)pyrimidine-4-carboxamide (0.032 g, 75% yield, 2HCl) which was used in the next step without further purification. ^{1}H NMR($CDCl_3$, 500 MHz) *δ*: 1.73 (m, 6H), 1.90 (s, 3H), 3.10 (m, 2H), 3.18 (m, 1H), 3.51 (m, 3H), 3.67 (m, 4H), 3.79 (m, 4H), 4.00 (m, 1H), 4.27 (m, 2H), 4.60 (m, 1H), 4.78 (m, 1H), 6.63 (s, 1H), 6.68 (s, 1H), 6.75 (d, 1H), 7.12 (d, 1H), OH and NH isn't observed. LCMS(ESI): [M+H]+ m/z: calcd 509.2; found 508.2; Rt = 2.47 min.

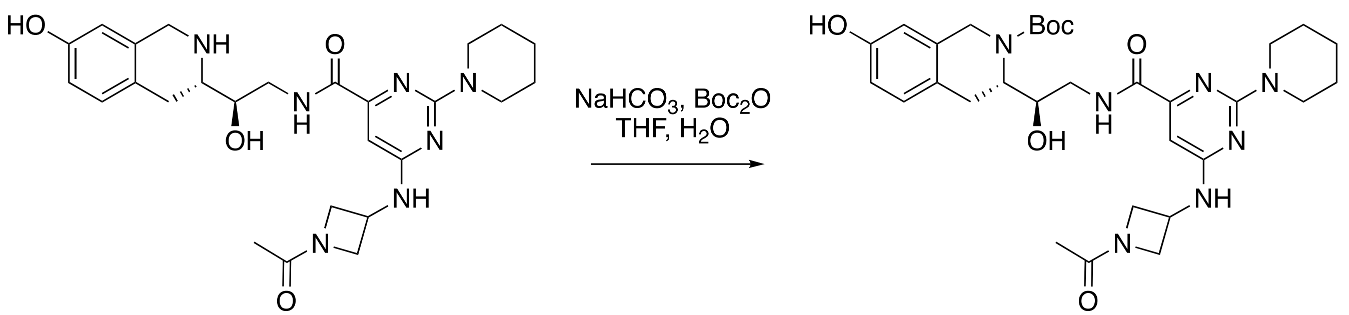

tert-butyl (*S*)-3-((*R*)-2-(6-((1-acetylazetidin-3-yl)amino)-2-(piperidin-1-yl)pyrimidine-4-carboxamido)-1-hydroxyethyl)-7-hydroxy-3,4-dihydroisoquinoline-2(1*H*)-carboxylate
6-[(1-acetylazetidin-3-yl)amino]-N-[(2*R*)-2-hydroxy-2-[(3*S*)-7-hydroxy-1,2,3,4-tetrahydroisoquinolin-3-yl]ethyl]-2-(1-piperidyl)pyrimidine-4-carboxamide (0.032 g, 54.93 µmol, 2HCl) as dissolved in a mixture of water (1 mL) and THF (1 mL) then sodium hydrogen carbonate, 99% (13.84 mg, 164.80 µmol) was added in one portion, after that solution of di-*tert*-butyl dicarbonate (11.99 mg, 54.93 µmol) in THF (0.2 mL) was added dropwise. The reaction mixture was stirred overnight at room temperature. Ethyl acetate (15mL) was added to the reaction mixture, organic phase was separated, and the aqueous phase was extracted with ethyl acetate (2X15mL). The organic phase was washed with brine, dried over Na_2SO_4, filtered and evaporated in vacuo at 40 °C to give *tert*-butyl (3*S*)-3-[(1*R*)-2-[[6-[(1-acetylazetidin-3-yl)amino]-2-(1-piperidyl)pyrimidine-4-carbonyl]amino]-1-hydroxy-ethyl]-7-hydroxy-3,4-dihydro-1*H*-isoquinoline-2-carboxylate (0.03 g, 49.20 umol, 90% yield) which was used in the next step without purification. ^{1}H NMR($CDCl_3$, 500 MHz) *δ*: 1.45 (m, 10H), 1.67 (m, 6H), 1.88 (s, 3H), 2.82 (m, 3H), 3.76 (m, 6H), 3.83 (m, 2H), 4.13 (m, 2H), 4.27 (m, 2H), 4.52 (m, 1H), 4.65 (m, 1H), 6.41 (s, 1H), 6.56 (s, 1H), 6.64 (d, 1H), 6.98 (d, 1H), OH and NH isn't observed. LCMS(ESI): [M+H]+ m/z: calcd 609.3; found 610.4; Rt = 1.38 min.

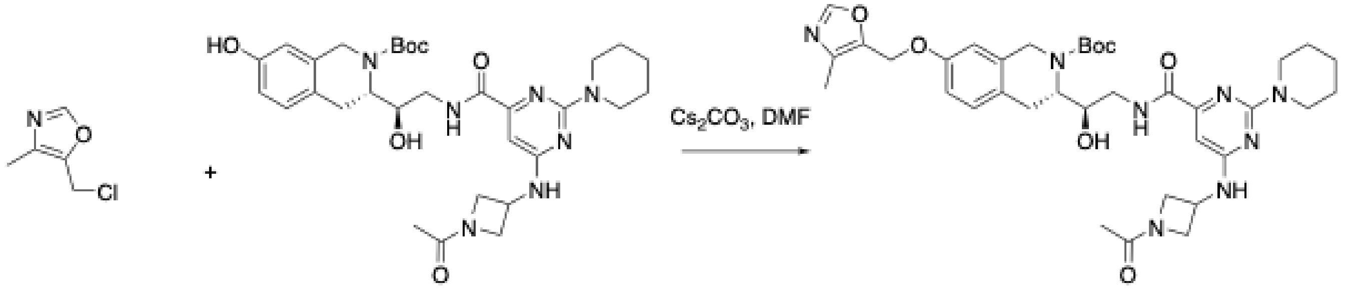

tert-butyl (*S*)-3-((*R*)-2-(6-((1-acetylazetidin-3-yl)amino)-2-(piperidin-1-yl)pyrimidine-4-carboxamido)-1-hydroxyethyl)-7-((4-methyloxazol-5-yl)methoxy)-3,4-dihydroisoquinoline-2(1*H*)-carboxylate.
tert-Butyl (3*S*)-3-[(1*R*)-2-[[6-[(1-acetylazetidin-3-yl)amino]-2-(1-piperidyl)pyrimidine-4-carbonyl]amino]-1-hydroxy-ethyl]-7-hydroxy-3,4-dihydro-1*H*-isoquinoline-2-carboxylate (0.03 g, 49.20 umol), 5-(chloromethyl)-4-methyl-oxazole (9.92 mg, 59.04 µmol, HCl) and cesium carbonate (48.09 mg, 147.61 µmol) was dissolved in DMF (2 mL) and heated at 50 °C overnight. The reaction mixture was filtered, the solid was washed with DMF (2mL), and the filtrate was concentrated on vacuo at 60 °C to give *tert*-butyl (3*S*)-3-[(1*R*)-2-[[6-[(1-acetylazetidin-3-yl)amino]-2-(1-piperidyl)pyrimidine-4-carbonyl]amino]-1-hydroxy-ethyl]-7-[(4-methyloxazol-5-yl)methoxy]-3,4-dihydro-1*H*-isoquinoline-2-carboxylate (0.034 g, 48.24 µmol, 98 % yield) which was used in the next step without further purification. ^{1}H NMR ($CDCl_3$, 400 MHz) *δ*: 1.50 (m, 19H), 1.86 (m, 2H), 2.21 (s, 3H), 3.12 (m, 1H), 3.69 (m, 6H), 4.00 (m, 2H), 4.21 (m, 4H), 4.62 (m, 2H), 4.96 (m, 2H), 6.41 (s, 1H) , 6.68 (s, 1H), 6.79 (d, 1H), 7.12 (d, 1H), 7.80 (s, 1H), 8.00 (s, 1H), 9.07 (m, 1H). LCMS(ESI): $[M+H]^+$ m/z: calcd 704.3; found 705.2; Rt = 4.29 min.

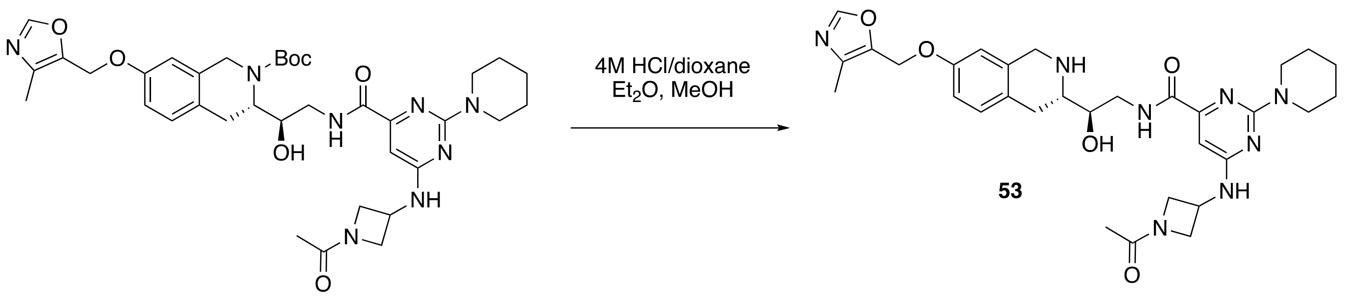

6-((1-acetylazetidin-3-yl)amino)-*N*-((*R*)-2-hydroxy-2-((*S*)-7-((4-methyloxazol-5-yl)methoxy)-1,2,3,4-tetrahydroisoquinolin-3-yl)ethyl)-2-(piperidin-1-yl)pyrimidine-4-carboxamide (**53**).
tert-Butyl (3*S*)-3-[(1*R*)-2-[[6-[(1-acetylazetidin-3-yl)amino]-2-(1-piperidyl)pyrimidine-4-carbonyl]amino]-1-hydroxy-ethyl]-7-[(4-methyloxazol-5-yl)methoxy]-3,4-dihydro-1*H*-isoquinoline-2-carboxylate (0.034 g, 48.24 µmol) was dissolved in a mixture of Et_2O (1 mL) and MeOH (0.2 mL). 4.0M hydrogen chloride solution in dioxane (131.91 mg, 3.62 mmol, 164.89 µL) was added. The solvent was removed on vacuo at 45 °C. The residue was dissolved in 5 mL of methanol and 10 mg of scavenger (SiliaMetS© Dimercaptotriazine(DMT) was added and the resulting suspension was stirred for 12 h. The suspension was filtered, the filtrate was evaporated under reduced pressure, and the residue was purified by HPLC (25-60% water-methanol, 10min, flow 30mL/min (loading pump 4mL/min methanol) , column: SUNFIRE C18 100 x 29mm to give 6-[(1-acetylazetidin-3-yl)amino]-N-[(2*R*)-2-hydroxy-2-[(3*S*)-7-[(4-methyloxazol-5-yl)methoxy]-1,2,3,4-tetrahydroisoquinolin-3-yl]ethyl]-2-(1-piperidyl)pyrimidine-4-carboxamide **53** (10.5 mg, 14.70 umol, 30% yield, 3HCl). ^{1}H NMR(MeOH-d_4 , 400 MHz): δ (ppm) 1.73 (m, 6H), 1.91 (s, 3H), 2.23 (s, 3H), 2.66 (s, 2H), 3.16 (m, 2H), 3.59 (m, 3H), 3.79 (m, 3H), 4.24 (m, 5H), 4.57 (m, 2H), 4.80 (m, 1H), 5.12 (s, 2H), 6.74 (s, 1H), 6.89 (s, 1H), 6.96 (d, 1H), 7.23 (d, 1H), 8.44 (s, 1H). ^{1}H NMR(DMSO-d_6, 500 MHz): δ (ppm) 1.48 (m, 4H), 1.59 (m, 2H), 1.74 (s, 3H), 2.13 (s, 3H), 2.23 (m, 1H), 2.50 (m, 1H), 2.71 (m, 2H), 3.35 (m, 1H), 3.51 (m, 2H), 3.71 (m, 5H), 3.84 (m, 2H), 3.94 (dd, 1H), 4.10 (t, 1H), 4.37 (t, 1H), 4.52 (m, 1H), 5.05 (s, 2H), 5.06 (m, 1H), 6.35 (s, 1H), 6.67 (d, 1H), 6.74 (dd, 1H), 6.98 (d, 1H), 7.88 (s, 1H), 8.26 (s, 1H), 8.77 (m, 1H). ^{13}C NMR (DMSO, 126 MHz): δ (ppm) 10.92, 18.74, 24.40, 25.32, 29.50, 43.31, 44.13, 47.79, 53.97, 57.10, 57.28, 58.63, 71.73, 111.46, 113.12, 127.58, 130.01, 134.25, 137.38, 142.24, 151.46, 155.59, 160.51, 163.66, 169.62. LCMS(ESI): [M+H]$^+$ m/z: calcd 604.3; found 605.3; Rt = 1.01 min. $[\alpha]^{21}_D$ = -44.69° (*c* = 0.25 g/100 mL, MeOH). Elemental analysis [calculated/found] C[61.57/60.85] H[6.67/6.82] N[18.53/18.38] O[13.23]. HRMS (ESI, + vw ion) m/z calcd for $C_{31}H_{40}N_8O_5$ [M+H$^+$] 604.31217, found 604.31084

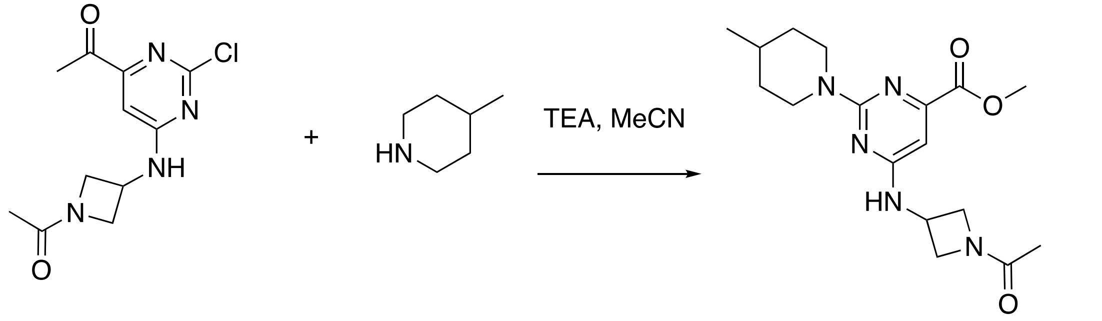

methyl 6-((3-acetylcyclobutyl)amino)-2-(4-methylpiperidin-1-yl)pyrimidine-4-carboxylate
To a solution of methyl 6-[(1-acetylazetidin-3-yl)amino]-2-chloro-pyrimidine-4-carboxylate (1.5 g, 5.27 mmol) in ACN (25 mL) at room temperature was added TEA (799.71 mg, 7.90 mmol, 1.10 mL) followed by 4-methylpiperidine (574.77 mg, 5.80 mmol, 685.89 µL) and the resulting reaction mixture was stirred at 80 °C for 12 h, then it was diluted with water (50 mL). The aqueous layer was extracted with DCM (50 mL). The combined organic layers were washed with water (25 mL) and brine, dried over Na_2SO_4 and concentrated in vacuo to give methyl 6-[(1-acetylazetidin-3-yl)amino]-2-(4-methyl-1-piperidyl)pyrimidine-4-carboxylate (1.6 g, 4.61 mmol, 87% yield). LCMS(ESI): [M+H]$^+$ m/z: calcd 347.2; found 348.2; Rt = 0.917 min.

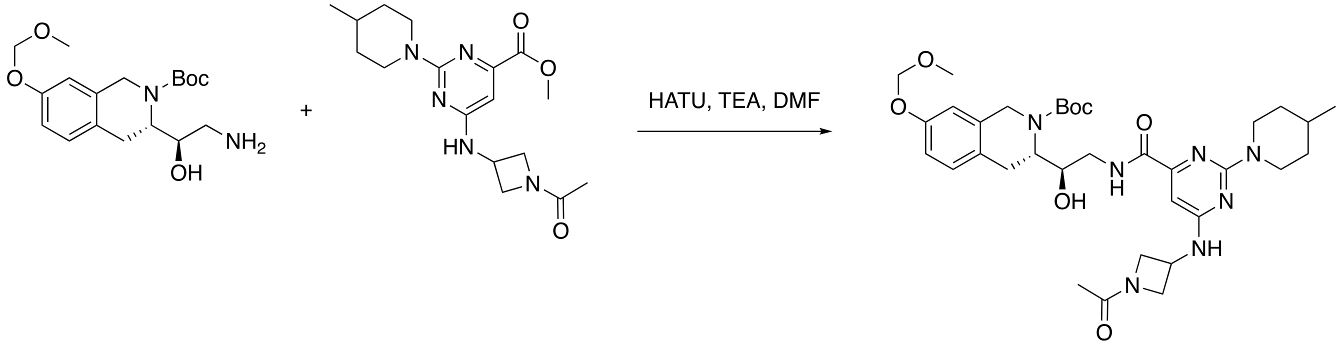

tert-butyl (*S*)-3-((*R*)-2-(6-((1-acetylazetidin-3-yl)amino)-2-(4-methylpiperidin-1-yl)pyrimidine-4-carboxamido)-1-hydroxyethyl)-7-(methoxymethoxy)-3,4-dihydroisoquinoline-2(1*H*)-carboxylate

Methyl 6-[(1-acetylazetidin-3-yl)amino]-2-(4-methyl-1-piperidyl)pyrimidine-4-carboxylate (197.16 mg, 567.50 μmol) and *tert*-butyl (3*S*)-3-[(1*R*)-2-amino-1-hydroxy-ethyl]-7-(methoxymethoxy)-3,4-dihydro-1*H*-isoquinoline-2-carboxylate (200 mg, 567.50 μmol) were mixed in methanol (2 mL) and heated at 75 °C for 72 h. After the completion of the reaction, the solution was evaporated under reduce pressure. The resulting crude product was purified by HPLC (70-95% water-acetonitrile, 2-10 min, flow: 30mL/min) to obtain *tert*-butyl (3*S*)-3-[(1*R*)-2-[[6-[(1-acetylazetidin-3-yl)amino]-2-(4-methyl-1-piperidyl)pyrimidine-4-carbonyl]amino]-1-hydroxy-ethyl]-7-(methoxymethoxy)-3,4-dihydro-1*H*-iso-quinoline-2-carboxylate (107.8 mg, 161.43 μmol, 28% yield). ^{1}H NMR($CDCl_3$, 400 MHz): *δ* 1.40 (m, 2H), 1.53 (m, 9H), 1.61 (m, 4H), 1.88 (s, 3H), 3.00 (m, 5H), 3.48 (m, 5H), 4.33 (m, 12H), 5.15 (s, 2H), 5.78 (m, 1H), 6.47 (s, 1H), 6.79 (s, 1H), 7.08 (d, 1H), 7.12 (d, 1H), 9.15 (m, 1H). LCMS(ESI): $[M+H]^+$ m/z: calcd 667.3; found 668.4; Rt = 1.58 min.

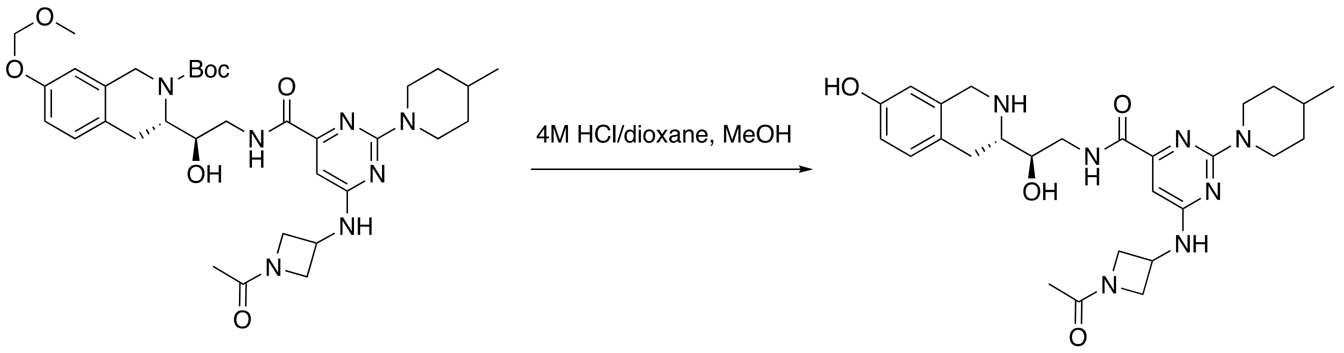

6-((1-acetylazetidin-3-yl)amino)-*N*-((*R*)-2-hydroxy-2-((*S*)-7-hydroxy-1,2,3,4-tetrahydroisoquinolin-3-yl)ethyl)-2-(4-methylpiperidin-1-yl)pyrimidine-4-carboxamide

The solution of *tert*-butyl (3*S*)-3-[(1*R*)-2-[[6-[(1-acetylazetidin-3-yl)amino]-2-(4-methyl-1-piperidyl)pyrimidine-4-carbonyl]amino]-1-hydroxy-ethyl]-7-(methoxymethoxy)-3,4-dihydro-1*H*-isoquinoline-2-carboxylate (107.8 mg, 161.43 μmol) in dioxane / HCl (2 mL) and methanol (2 mL) was stirred for 12 h at 25 °C. Then the solution was evaporated to obtain 6-[(1-acetylazetidin-3-yl)amino]-N-[(2*R*)-2-hydroxy-2-[(3*S*)-7-hydroxy-1,2,3,4-tetrahydroisoquinolin-3-yl]ethyl]-2-(4-methyl-1-piperidyl)-pyrimidine-4-carboxamide (100 mg, crude, 3HCl). ^{1}H NMR(MeOD, 400 MHz): *δ* 1.14 (m, 5H), 1.82 (m, 5H), 3.07 (m, 2H), 3.50 (m, 2H), 4.21 (m, 17H), 6.63 (s, 1H), 6.71 (s, 1H), 6.85 (d, 1H), 7.07 (d, 1H). LCMS(ESI): [M+H]+ m/z: calcd 523.3; found 524.2; Rt = 0.95 min.

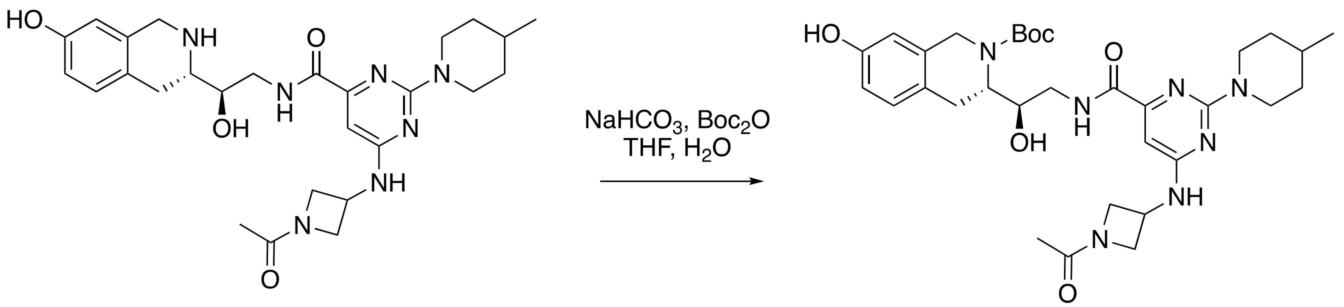

tert-butyl (*S*)-3-((*R*)-2-(6-((1-acetylazetidin-3-yl)amino)-2-(4-methylpiperidin-1-yl)pyrimidine-4-carboxamido)-1-hydroxyethyl)-7-hydroxy-3,4-dihydroisoquinoline-2(1*H*)-carboxylate

To a stirred solution of sodium hydrogen carbonate (66.36 mg, 789.88 µmol) in water (2 mL) the solution of 6-[(1-acetylazetidin-3-yl)amino]-N-[(2*R*)-2-hydroxy-2-[(3*S*)-7-hydroxy-1,2,3,4-tetrahydroisoquinolin-3-yl]ethyl]-2-(4-methyl-1-piperidyl)pyrimidine-4-carboxamide (100 mg, 157.98 µmol, 3HCl) in THF (1.5 mL) was added. The resulting mixture was stirred for 5 min at room temperature followed by the addition of di-*tert*-butyl dicarbonate (36.20 mg, 165.87 umol, 38.07 uL) in THF (1.5 mL). The resulting mixture was stirred at 25 °C for 12 h. After the completion of the reaction, EtOAc (10 mL) was added and the organic phase was separated and washed with brine (2 x 5 mL). Then the solvent was dried over sodium sulfate, filtered, and evaporated to obtain *tert*-butyl (3*S*)-3-[(1*R*)-2-[[6-[(1-acetylazetidin-3-yl)amino]-2-(4-methyl-1-piperidyl)pyrimidine-4-carbonyl]amino]-1-hydroxy-ethyl]-7-hydroxy-3,4-dihydro-1*H*-isoquinoline-2-carboxylate (85 mg, crude). LCMS(ESI): [M+H]+ m/z: calcd 623.3; found 624.4; Rt = 1.42 min.

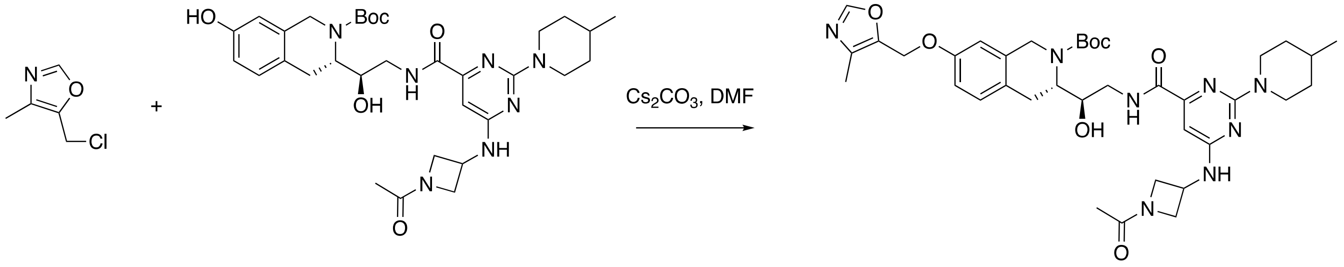

tert-butyl (*S*)-3-((*R*)-2-(6-((1-acetylazetidin-3-yl)amino)-2-(4-methylpiperidin-1-yl)pyrimidine-4-carboxamido)-1-hydroxyethyl)-7-((4-methyloxazol-5-yl)methoxy)-3,4-dihydroisoquinoline-2(1*H*)-carboxylate

To the solution of *tert*-butyl (3*S*)-3-[(1*R*)-2-[[6-[(1-acetylazetidin-3-yl)amino]-2-(4-methyl-1-piperidyl)pyrimidine-4-carbonyl]amino]-1-hydroxy-ethyl]-7-hydroxy-3,4-dihydro-1*H*-isoquinoline-2-carboxylate (85 mg, 136.27 µmol) and cesium carbonate (133.20 mg, 408.82 µmol) in DMF (3 mL), 5-(chloromethyl)-4-methyl-oxazole (27.48 mg, 163.53 µmol, HCl) was added. The resulting mixture was heated at 50 °C for 12 h. The mixture was filtered and evaporated to obtain *tert*-butyl (3*S*)-3-[(1*R*)-2-[[6-[(1-acetylazetidin-3-yl)amino]-2-(4-methyl-1-piperidyl)pyrimidine-4-carbonyl]amino]-1-hydroxy-ethyl]-7-[(4-methyloxazol-5-yl)methoxy]-3,4-dihydro-1*H*-isoquinoline-2-carboxylate (95 mg, crude) that was used without further purification. LCMS(ESI): $[M+H]^+$ m/z: calcd 718.4; found 719.4; Rt = 1.46 min.

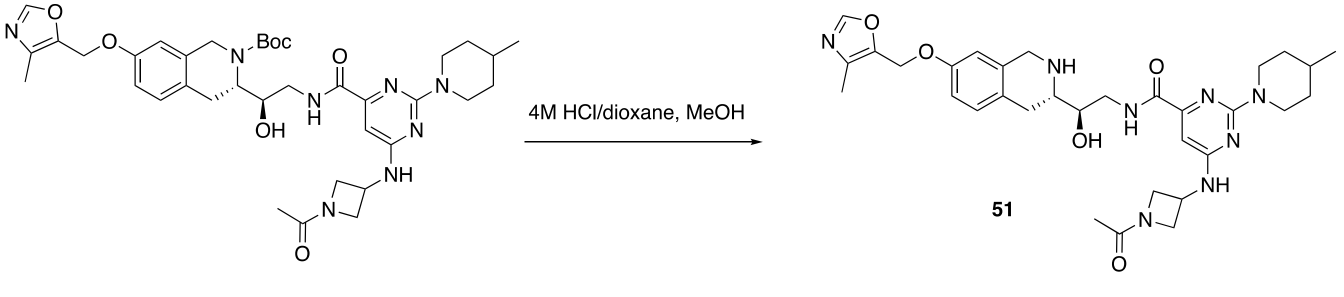

51

6-((1-acetylazetidin-3-yl)amino)-*N*-((*R*)-2-hydroxy-2-((*S*)-7-((4-methyloxazol-5-yl)methoxy)-1,2,3,4-tetrahydroisoquinolin-3-yl)ethyl)-2-(4-methylpiperidin-1-yl)pyrimidine-4-carboxamide (**51**).
The solution of *tert*-butyl (3*S*)-3-[(1*R*)-2-[[6-[(1-acetylazetidin-3-yl)amino]-2-(4-methyl-1-piperidyl)pyrimidine-4-carbonyl]amino]-1-hydroxy-ethyl]-7-[(4-methyloxazol-5-yl)methoxy]-3,4-dihydro-1*H*-isoquinoline-2-carboxylate (95 mg, 132.16 μmol) in dioxane / HCl (2 mL) and methanol (2 mL) was stirred for 3 h at 25 °C. The resulting mixture was stirred with SiliaMetS® DMT (30 mg) in methanol (1 mL) for 12 h. The obtained solution was filtered, evaporated, and purified by HPLC (05_methanol+NH_3) to obtain 6-[(1-acetylazetidin-3-yl)amino]-N-[(2*R*)-2-hydroxy-2-[(3*S*)-7-[(4-methyloxazol-5-yl)methoxy]-1,2,3,4-tetrahydroisoquinolin-3-yl]ethyl]-2-(4-methyl-1-piperidyl)pyrimidine-4-carboxamide **51** (10.4 mg, 16.81 μmol, 13% yield). ^{1}H NMR (500 MHz, DMSO-*d*$_6$) *δ* 0.97 (d, 3H), 1.09 (m, 3H), 1.66 (m, 3H), 1.77 (s, 3H), 2.18 (s, 3H), 2.60 (m, 1H), 2.78 (m, 4H), 3.39 (m, 1H), 3.56 (m, 2H), 3.79 (m, 1H), 3.92 (s, 2H), 4.00 (m, 1H), 4.13 (m, 1H), 4.38 (m, 1H), 4.59 (m, 1H), 4.69 (m, 2H), 4.94 (m, 1H), 4.98 (s, 2H), 6.37 (s, 1H), 6.61 (s, 1H), 6.70 (d, 1H), 6.98 (d, 1H), 7.74 (m, 1H), 8.02 (s, 1H), 8.56 (m, 1H). LCMS(ESI): $[M+H]^+$ m/z: calcd 618.3; found 619.4; Rt = 1.07 min.

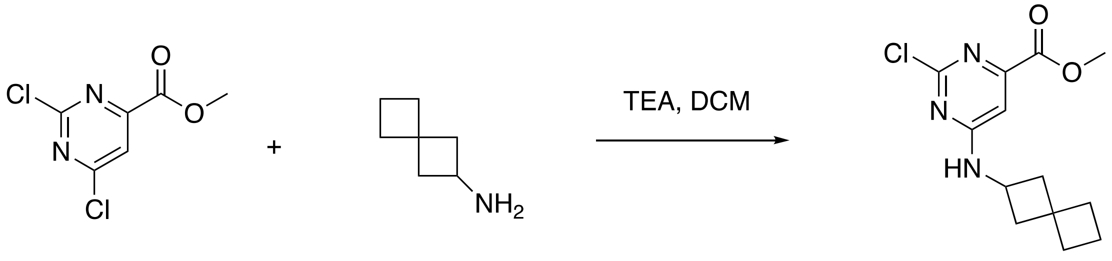

methyl 2-chloro-6-(spiro[3.3]heptan-2-ylamino)pyrimidine-4-carboxylate
To a solution of methyl 2,6-dichloropyrimidine-4-carboxylate (8 g, 38.64 mmol) in DCM (120 mL) at 0 °C was added TEA (79.22 mmol, 11.04 mL) followed by spiro[3.3]heptan-2-amine (5.71 g, 38.64 mmol, HCl) and the resulting reaction mixture was stirred at 0 °C for 30 min and then allowed to warm to room temperature. After 14 h the reaction mixture was washed with water (100 mL). The organic layer was dried over Na_2SO_4 and evaporated. The residue was recrystallized (hexane/MTBE) to give methyl 2-chloro-6-(spiro[3.3]heptan-2-ylamino)pyrimidine-4-carboxylate (4.35 g, 15.44 mmol, 40% yield). LCMS(ESI): $[M+H]^+$ m/z: calcd 281.74; found 282.2; Rt = 1.37 min.

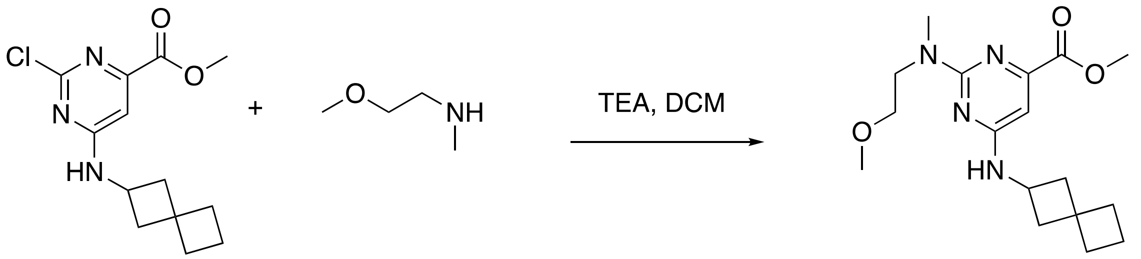

methyl 2-((2-methoxyethyl)(methyl)amino)-6-(spiro[3.3]heptan-2-ylamino)pyrimidine-4-carboxylate
To a solution of methyl 2-chloro-6-(spiro[3.3]heptan-2-ylamino)pyrimidine-4-carboxylate (1.4 g, 4.97 mmol) in MeCN (45 mL) at room temperature was added TEA (7.45 mmol, 1.04 mL) followed by 2-methoxy-N-methyl-ethanamine (4.97 mmol, 533.65 μL) and the resulting reaction mixture was stirred at 25 °C for 17 h then diluted with water (50 mL). The aqueous layer was extracted with DCM (50 mL) and the combined organic layers were washed with water (25 mL) and brine, dried over Na_2SO_4 and concentrated in vacuo to give methyl 2-[2-methoxyethyl(methyl)amino]-6-(spiro[3.3]heptan-2-ylamino)pyrimidine-4-carboxylate (1.52 g, 4.55 mmol, 91% yield). LCMS(ESI): $[M+H]^+$ m/z: calcd 334.42; found 335.2; Rt = 1.13 min.

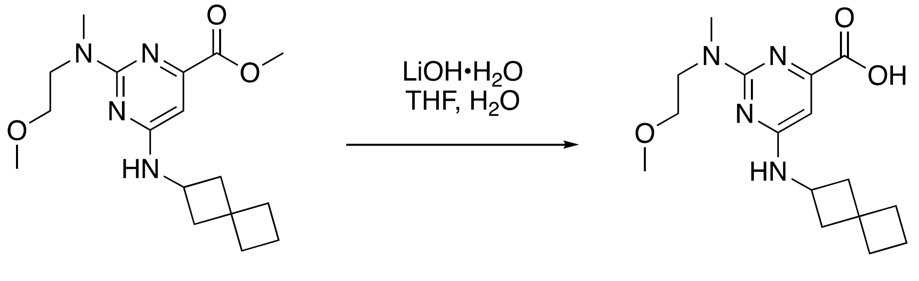

2-((2-methoxyethyl)(methyl)amino)-6-(spiro[3.3]heptan-2-ylamino)pyrimidine-4-carboxylic acid
To the solution of methyl 2-[2-methoxyethyl(methyl)amino]-6-(spiro[3.3]heptan-2-ylamino)pyrimidine-4-carboxylate (1.59 g, 4.74 mmol) in H_2O (15 mL) andTHF (20 mL), lithium hydroxide monohydrate, 98% (437.99 mg, 10.44 mmol) was added and the reaction mixture was stirred at 25 °C for 1.5 h after which the reaction mixture was evaporated to dryness. The residual water solution was acidified with sodium bisulfate to slightly acidic pH with NH_4Cl (aq). The desired product was extracted with DCM (3 x 25 mL) and dried over Na_2SO_4. The organic layer was evaporated to give 2-[2-methoxyethyl(methyl)amino]-6-(spiro[3.3]heptan-2-ylamino)pyrimidine-4-carboxylic acid (1.1 g, 3.43 mmol, 72% yield). LCMS(ESI): $[M+H]^+$ m/z: calcd 320.18; found 321.2; Rt = 1.15 min.

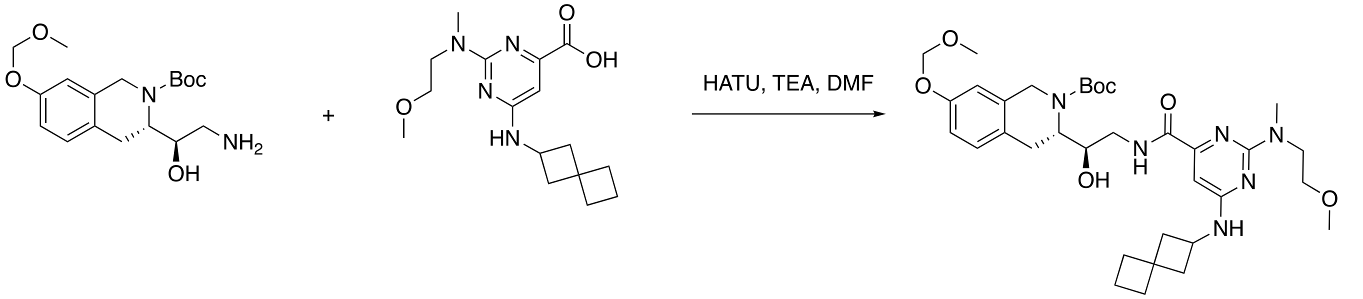

tert-butyl (*S*)-3-((*R*)-1-hydroxy-2-(2-((2-methoxyethyl)(methyl)amino)-6-(spiro[3.3]heptan-2-ylamino)pyrimidine-4-carboxamido)ethyl)-7-(methoxymethoxy)-3,4-dihydroisoquinoline-2(1*H*)-carboxylate
tert-Butyl (3*S*)-3-[(1*R*)-2-amino-1-hydroxy-ethyl]-7-(methoxymethoxy)-3,4-dihydro-1*H*-iso-quinoline-2-carboxylate (0.2 g, 567.50 µmol), 2-[2-methoxyethyl(methyl)amino]-6-(spiro[3.3]heptan-2-ylamino)pyrimidine-4-carboxylic acid (181.82 mg, 567.50 µmol) and triethylamine (791 µL) were mixed together in DMF (3 mL) and HATU (323.67 mg, 851.25 µmol) was added. The reaction mixture was stirred for 18 h. The reaction mixture was poured into water (15 mL) and the resulting mixture was extracted with EtOAc (2 x 20 mL). The combined organic layers were washed with water (3 x 15 mL), brine, dried over Na_2SO_4, filtered and evaporated to obtain *tert*-butyl (3*S*)-3-[(1*R*)-1-hydroxy-2-[[2-[2-methoxyethyl(methyl)amino]-6-(spiro[3.3]heptan-2-ylamino)pyrimidine-4-carbonyl]-amino]ethyl]-7-(methoxymethoxy)-3,4-dihydro-1*H*-isoquinoline-2-carboxylate (319.30 mg, 487.63 µmol, 86% yield) as a brown solid. ^{1}H NMR (400 MHz, DMSO-d_6) δ (ppm) 1.41 (m, 9H), 1.80 (m, 6H), 2.02 (m, 3H), 2.36 (m, 3H), 3.03 (m, 4H), 3.24 (s, 3H), 3.35 (s, 3H), 3.51 (m, 4H), 3.68 (m, 2H), 4.10 (m, 3H), 4.73 (m, 1H), 5.14 (m, 3H), 6.27 (s, 1H), 6.82 (s, 2H),7.05 (d, 1H), 7.44 (m, 1H), 8.37 (m, 1H). LCMS(ESI): $[M+H]^+$ m/z: calcd 655.44; found 655.4; Rt = 1.527 min.

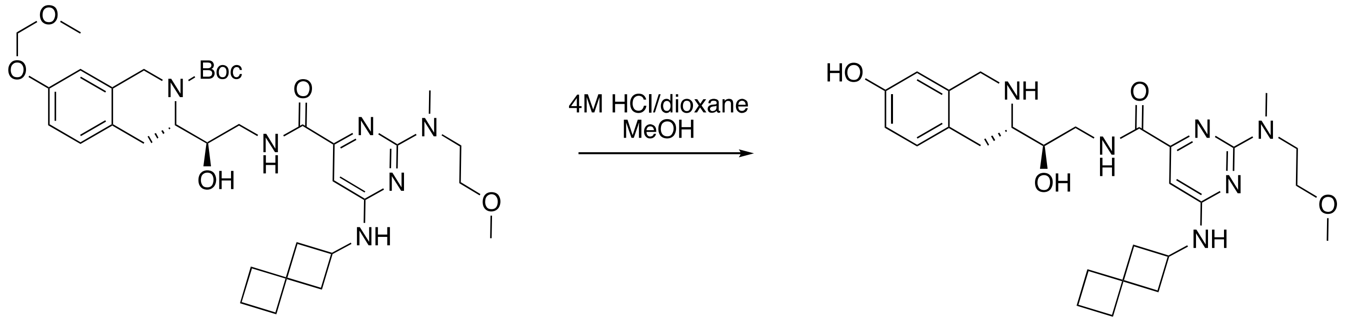

N-((*R*)-2-hydroxy-2-((*S*)-7-hydroxy-1,2,3,4-tetrahydroisoquinolin-3-yl)ethyl)-2-((2-methoxyethyl)(methyl)amino)-6-(spiro[3.3]heptan-2-ylamino)pyrimidine-4-carboxamide
tert-Butyl (3S)-3-[(1*R*)-1-hydroxy-2-[[2-[2-methoxyethyl(methyl)amino]-6-(spiro[3.3]heptan-2-ylamino)pyrimidine-4-carbonyl]amino]ethyl]-7-(methoxymethoxy)-3,4-dihydro-1*H*-isoquinoline-2-carboxylate (319.30 mg, 487.63 µmol)

was dissolved in MeOH (3 mL) and 4.0M hydrogen chloride solution in dioxane (3.90 mmol, 177.80 µL) was added. The resulting mixture was stirred for 3 h and evaporated to dryness to obtain N-[(2*R*)-2-hydroxy-2-[(3*S*)-7-hydroxy-1,2,3,4-tetrahydroisoquinolin-3-yl]ethyl]-2-[2-methoxyethyl(methyl)amino]-6-(spiro[3.3]heptan-2-ylamino)pyrimidine-4-carboxamide (0.2998 g, crude, 2HCl) as a light-yellow solid. ^{1}H NMR (500 MHz, DMSO-d_6) δ (ppm) 1.23 (m, 5H), 1.82 (m, 5H), 1.99 (m, 3H), 2.39 (m, 2H), 2.97 (m, 2H), 3.26 (s, 3H), 3.40 (m, 3H), 3.53 (m, 3H), 3.74 (m, 2H), 4.18 (m, 6H), 6.45 (s, 1H), 6.59 (s, 1H), 6.68 (d, 1H), 7.02 (d, 1H), 8.91 (m, 1H), 9.70 (m, 1H). LCMS(ESI): [M+2H]$^+$ m/z: calcd 512.36; found 512.4; Rt = 1.136 min.

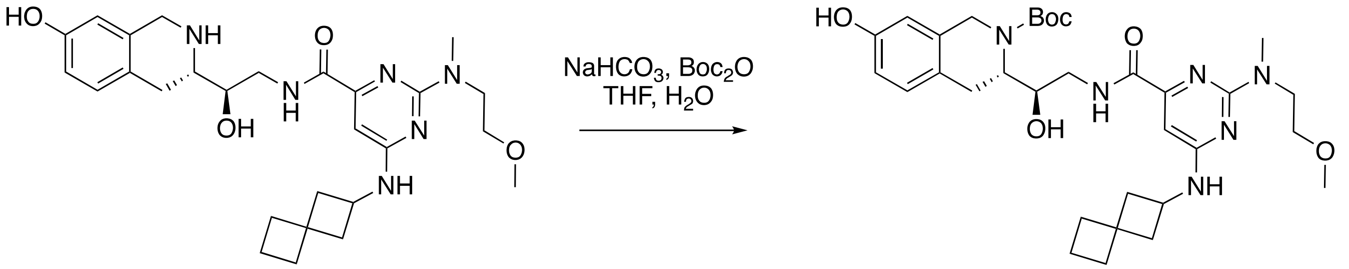

tert-butyl (*S*)-7-hydroxy-3-((*R*)-1-hydroxy-2-(2-((2-methoxyethyl)(methyl)amino)-6-(spiro[3.3]heptan-2-ylamino)pyrimidine-4-carboxamido)ethyl)-3,4-dihydroisoquinoline-2(1*H*)-carboxylate

N-[(2*R*)-2-Hydroxy-2-[(3*S*)-7-hydroxy-1,2,3,4-tetrahydroisoquinolin-3-yl]ethyl]-2-[2-methoxyethyl(methyl)amino]-6-(spiro[3.3]heptan-2-ylamino)pyrimidine-4-carboxamide (0.28456 g, 487.64 µmol, 2HCl) was dissolved in water (2 mL) and sodium bicarbonate (245.79 mg, 2.93 mmol) was added. The resulting mixture was diluted with THF (2 mL) and di-*tert*-butyl dicarbonate (106 mg, 488 µmol) was added. The reaction mixture was stirred for 18 h. The reaction mixture was extracted with EtOAc (2 x 25 mL) and the combined organic layers were washed with brine, dried over Na_2SO_4, filtered, and evaporated to obtain *tert*-butyl (3*S*)-7-hydroxy-3-[(1*R*)-1-hydroxy-2-[[2-[2-methoxyethyl(methyl)amino]-6-(spiro[3.3]heptan-2-ylamino)pyrimidine-4-carbonyl]amino]ethyl]-3,4-dihydro-1*H*-isoquinoline-2-carboxylate (0.306 g, 501.84 µmol, 89% yield) as a light-yellow solid. ^{1}H NMR (500 MHz, DMSO-d_6) δ (ppm) 1.40 (m, 9H), 1.91 (m, 8H), 2.37 (m, 3H), 2.95 (m, 5H), 3.24 (s, 3H), 3.50 (m, 3H), 3.70 (m, 3H), 4.06 (m, 1H), 4.18 (m, 2H), 4.62 (m, 1H), 5.11 (m, 1H), 6.28 (m, 1H), 6.51 (m, 1H), 6.58 (m, 1H), 6.92 (d, 1H), 4.76 (m, 1H), 8.33 (m, 1H), 9.18 (m, 1H). LCMS(ESI): [M+H]$^+$ m/z: calcd 611.41; found 611.4; Rt = 1.507 min.

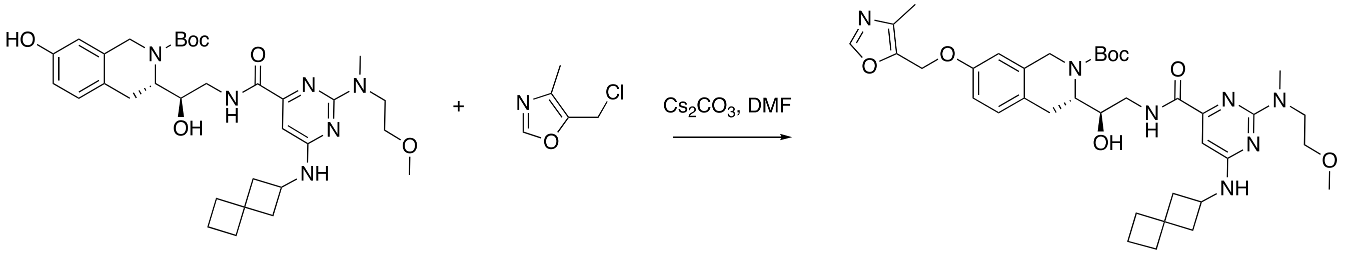

tert-butyl (*S*)-3-((*R*)-1-hydroxy-2-(2-((2-methoxyethyl)(methyl)amino)-6-(spiro[3.3]heptan-2-ylamino)pyrimidine-4-carboxamido)ethyl)-7-((4-methyloxazol-5-yl)methoxy)-3,4-dihydroisoquinoline-2(1*H*)-carboxylate

tert-Butyl (3*S*)-7-hydroxy-3-[(1*R*)-1-hydroxy-2-[[2-[2-methoxyethyl(methyl)amino]-6-(spiro[3.3]heptan-2-ylamino)pyrimidine-4-carbonyl]amino]ethyl]-3,4-dihydro-1*H*-isoquinoline-2-carboxylate (0.2548 g, 417.20 µmol), 5-(chloromethyl)-4-methyl-oxazole (98.14 mg, 584.08 µmol, HCl) and cesium carbonate (543.72 mg, 1.67 mmol) were mixed together in DMF (3 mL) and the resulting mixture was heated at 60 °C for 18 h. The reaction mixture was cooled to room temperature and poured into water (15 mL). The resulting mixture was extracted with EtOAc (2 x 20 mL) and the combined organic layers were washed with water (4 x 15 mL), brine, dried over Na_2SO_4, filtered, and evaporated to obtain *tert*-butyl (3*S*)-3-[(1*R*)-1-hydroxy-2-[[2-[2-methoxyethyl(methyl)amino]-6-(spiro[3.3]heptan-2-ylamino)pyrimidine-4-carbonyl]amino]ethyl]-7-[(4-methyloxazol-5-yl)methoxy]-3,4-dihydro-1*H*-isoquinoline-2-carboxylate (0.2525 g, 357.73 µmol, 86% yield) as a light-yellow solid. ^{1}H NMR (400 MHz, DMSO-d_6) δ (ppm) 1.41 (m, 9H), 1.80 (m, 7H), 1.99 (m, 3H), 2.14 (s, 3H), 2.35 (m, 3H), 3.04 (m, 5H), 3.11 (s, 4H), 3.49 (m, 2H), 3.69 (m, 2H), 4.15 (m, 3H), 4.18 (m, 1H), 5.07 (s, 2H), 5.30 (m, 1H), 6.27 (s, 1H), 6.84 (s, 1H), 7.07 (d, 1H), 7.45 (m, 1H), 8.27 (s, 1H), 8.44 (m, 1H). LCMS(ESI): [M+H]$^+$ m/z: calcd 706.45; found 706.4; Rt = 1.608 min.

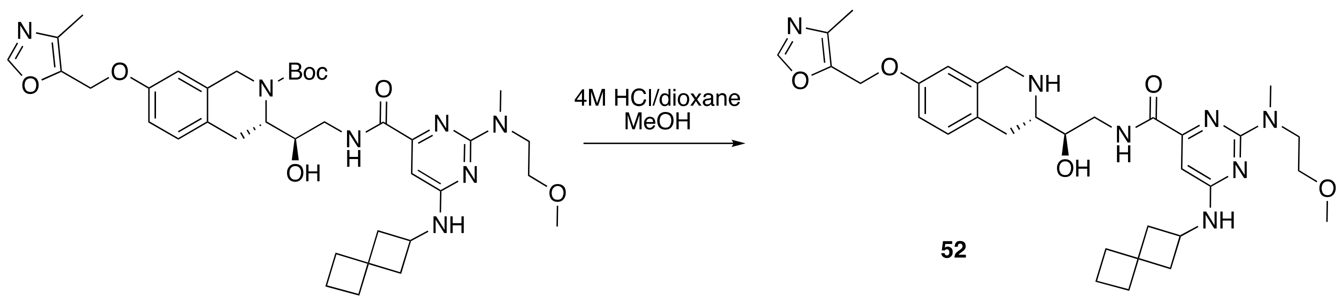

N-((*R*)-2-hydroxy-2-((*S*)-7-((4-methyloxazol-5-yl)methoxy)-1,2,3,4-tetrahydroisoquinolin-3-yl)ethyl)-2-((2-methoxyethyl)(methyl)amino)-6-(spiro[3.3]heptan-2-ylamino)pyrimidine-4-carboxamide (**52**). *tert*-Butyl (3*S*)-3-[(1*R*)-1-hydroxy-2-[[2-[2-methoxyethyl(methyl)amino]-6-(spiro[3.3]heptan-2-ylamino)pyrimidine-4-carbonyl]amino]ethyl]-7-[(4-methyloxazol-5-yl)methoxy]-3,4-dihydro-1*H*-isoquinoline-2-carboxylate (252.5 mg, 358 mmol) was dissolved in MeOH (3 mL) and 4.0M hydrogen chloride solution in dioxane (72.15 mmol, 97.8 µL) was added. The resulting mixture was stirred for 2 hr and evaporated to dryness. The residue was dissolved in 5 mL of MeOH and 30 mg of scavenger (SiliaMetS© Dimercaptotriazine(DMT)) was added and the resulting suspension was stirred for 12 h. The suspension was filtered and the filtrate was evaporated under reduced pressure. The residue was purified by HPLC (40-80%, 0-6.5 min water+NH_3-acetonitrile+NH_3, flow 30 mL/min (loading pump 4 mL/min ACN)) to obtain N-[(2*R*)-2-hydroxy-2-[(3*S*)-7-[(4-methyloxazol-5-yl)methoxy]-1,2,3,4-tetrahydroisoquinolin-3-yl]ethyl]-2-[2-methoxyethyl(methyl)amino]-6-(spiro[3.3]heptan-2-ylamino)pyrimidine-4-carboxamide, **52** (0.0719 g, 118.70 µmol, 33% yield) as a light-yellow gum. ^{1}H NMR (500 MHz, DMSO-d_6 + CCl_4) *δ* 1.79 (m, 2H), 1.90 (m, 4H), 2.02 (t, 2H), 2.15 (s, 4H), 2.37 (t, 2H), 2.72 (m, 2H), 3.10 (s, 3H), 3.24 (s, 3H), 3.36 (s, 2H), 3.51 (m, 4H), 3.71 (m, 2H), 3.85 (q, 2H), 4.18 (s, 1H), 5.06 (s, 3H), 6.32 (s, 1H), 6.69 (s, 1H), 6.75 (d, 1H), 7.00 (d, 1H), 7.47 (m, 1H), 8.27 (s, 1H), 8.65 (t, 1H). LCMS(ESI): $[M+H]^+$ m/z: calcd 607.4; found 607.4; Rt = 1.24 min.

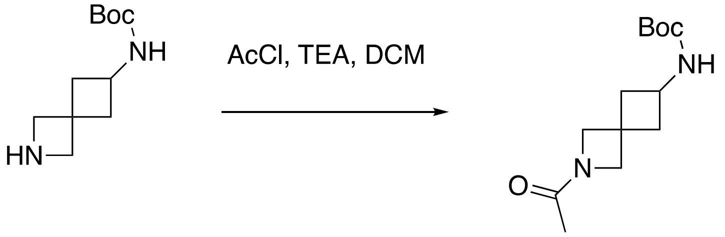

tert-butyl (2-acetyl-2-azaspiro[3.3]heptan-6-yl)carbamate
tert-butyl N-(2-azaspiro[3.3]heptan-6-yl)carbamate (1.5 g, 6.03 mmol, HCl) was suspended in dichloromethane (30 mL) with triethylamine (15.08 mmol, 2.10 mL). Acetyl chloride (7.24 mmol, 440.33 µL) was added dropwise over 5 minutes. The resulting mixture was stirred at 20 °C for 1 h then was quenched with water (10 ml). The organic layer was separated and washed with water (2x10 mL), dried over Na_2SO_4, and evaporated in vacuo, affording *tert*-butyl N-(2-acetyl-2-azaspiro[3.3]heptan-6-yl)carbamate (1.36 g, 5.35 mmol, 89% yield). LCMS(ESI): $[M+H]^+$ m/z: calcd 254.16; found 255.2; Rt = 1.003 min.

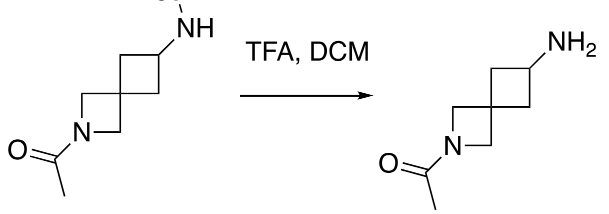

1-(6-amino-2-azaspiro[3.3]heptan-2-yl)ethan-1-one
Trifluoroacetic acid (55.05 mmol, 4.24 mL) was added to a solution of *tert*-butyl N-(2-acetyl-2-azaspiro[3.3]heptan-6-yl)carbamate (1.4 g, 5.50 mmol) in dichloromethane (15 mL). The resulting mixture was stirred at 20 °C for 12 h, then the volatiles were removed under reduced pressure, affording 1-(6-amino-2-azaspiro[3.3]heptan-2-yl)ethanone (3.5 g, crude, 4TFA). LCMS(ESI): $[M+H]^+$ m/z: calcd 154.21; found 155.2; Rt = 0.173 min.

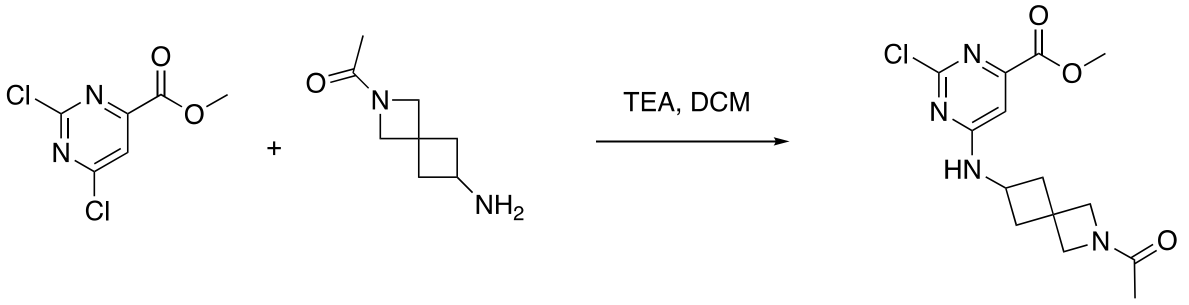

methyl 6-((2-acetyl-2-azaspiro[3.3]heptan-6-yl)amino)-2-chloropyrimidine-4-carboxylate
1-(6-amino-2-azaspiro[3.3]heptan-2-yl)ethanone (3.5 g, 5.77 mmol, 4TFA) and methyl 2,6-dichloropyrimidine-4-carboxylate (1.20 g, 5.77 mmol) were stirred in dichloromethane (20 mL). DIPEA (28.86 mmol, 5.03 mL) was added and the resulting mixture was stirred at 25 °C for 16 h, then it was concentrated under reduced pressure and the residue was purified by column chromatography (RediSep Column: 40 g ; MTBE-MeOH 0-25% ; Flow Rate: 40 mL/min) affording methyl 6-[(2-acetyl-2-azaspiro[3.3]heptan-6-yl)amino]-2-chloro-pyrimidine-4-carboxylate (827 mg, 2.55 mmol, 44% yield). LCMS(ESI): $[M+H]^+$ m/z: calcd 324.77; found 325.2; Rt = 1.003 min.

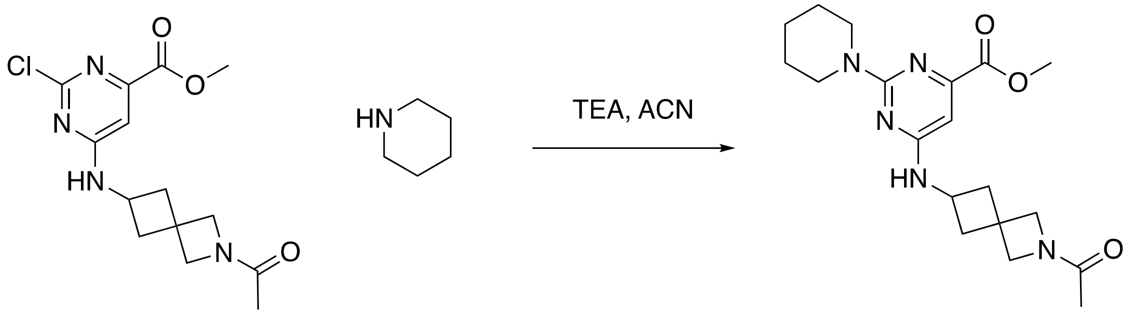

methyl 6-((2-acetyl-2-azaspiro[3.3]heptan-6-yl)amino)-2-(piperidin-1-yl)pyrimidine-4-carboxylate
TEA (5.09 mmol, 709.86 µL) and piperidine (3.06 mmol, 301.85 µL) were added to a solution of methyl 6-[(2-acetyl-2-azaspiro[3.3]heptan-6-yl)amino]-2-chloro-pyrimidine-4-carboxylate (827 mg, 2.55 mmol) in acetonitrile (15 mL). The resulting mixture was stirred at 82 °C for 16 h, then the solvent was removed under reduced pressure and the residue was triturated with water (10mL). The precipitate was filtered, rinsed with water, and dried, affording methyl 6-[(2-acetyl-2-azaspiro[3.3]heptan-6-yl)amino]-2-(1-piperidyl)pyrimidine-4-carboxylate (750 mg, 2.01 mmol, 79% yield). LCMS(ESI): $[M+H]^+$ m/z: calcd 373.46; found 374.2; Rt = 0.915 min.

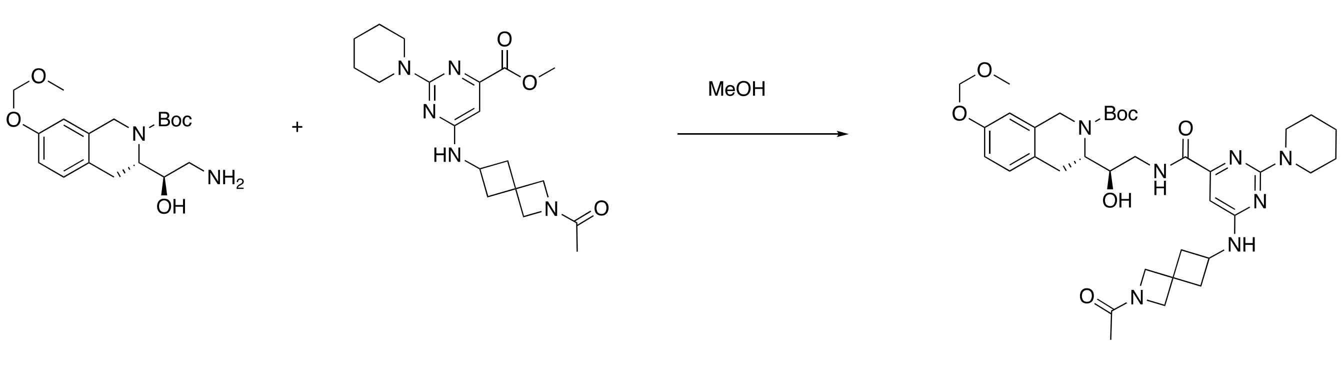

tert-butyl (*S*)-3-((*R*)-2-(6-((2-acetyl-2-azaspiro[3.3]heptan-6-yl)amino)-2-(piperidin-1-yl)pyrimidine-4-carboxamido)-1-hydroxyethyl)-7-(methoxymethoxy)-3,4-dihydroisoquinoline-2(1*H*)-carboxylate
tert-butyl (3*S*)-3-[(1*R*)-2-amino-1-hydroxy-ethyl]-7-(methoxymethoxy)-3,4-dihydro-1*H*-isoquinoline-2-carboxylate (0.2 g, 567.50 µmol) and methyl 6-[(2-acetyl-2-azaspiro[3.3]heptan-6-yl)amino]-2-(1-piperidyl)pyrimidine-4-carboxylate (211.93 mg, 567.50 µmol) were stirred in MeOH (3 mL) and the resulting mixture was heated at 75 °C for 65 hr. The reaction mixture was cooled to room temperature and evaporated to dryness. The residue was purified by HPLC (30-40% water-acetonitrile, 10 min, flow 30mL/min (loading pump 4 mL/min acetonitrile)) to

obtain *tert*-butyl (3*S*)-3-[(1*R*)-2-[[6-[(2-acetyl-2-azaspiro[3.3]heptan-6-yl)amino]-2-(1-piperidyl)pyrimidine-4-carbonyl]amino]-1-hydroxy-ethyl]-7-(methoxymethoxy)-3,4-dihydro-1*H*-isoquinoline-2-carboxylate (154.70 mg, 222.97 µmol, 39% yield). ^{1}H NMR(DMSO-d_6, 400 MHz): δ 1.39 (m, 15H), 1.45 (m, 2H), 1.72 (m, 3H), 2.10 (m, 2H), 2.87 (m, 4H), 3.36 (m, 4H), 3.73 (m, 6H), 4.10 (m, 6H), 4.70 (m, 1H), 5.14 (m, 3H), 6.83 (m, 2H), 7.08 (d, 1H), 7.51 (m, 1H), 8.49 (m, 1H). LCMS(ESI): $[M+H]^+$ m/z: calcd 693.85; found 694.4; Rt = 1.496 min.

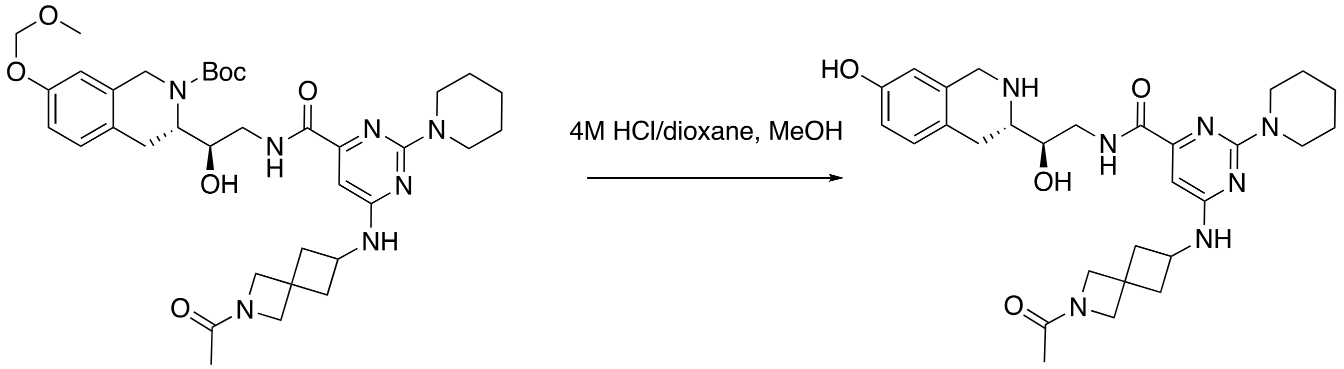

6-((2-acetyl-2-azaspiro[3.3]heptan-6-yl)amino)-*N*-((*R*)-2-hydroxy-2-((*S*)-7-hydroxy-1,2,3,4-tetrahydroisoquinolin-3-yl)ethyl)-2-(piperidin-1-yl)pyrimidine-4-carboxamide

tert-Butyl (3*S*)-3-[(1*R*)-2-[[6-[(2-acetyl-2-azaspiro[3.3]heptan-6-yl)amino]-2-(1-piperidyl)pyrimidine-4-carbonyl]amino]-1-hydroxy-ethyl]-7-(methoxymethoxy)-3,4-dihydro-1*H*-isoquinoline-2-carboxylate (154.70 mg, 222.97 µmol) was dissolved in MeOH (4 mL) and 4.0M hydrogen chloride solution in dioxane (2.68 mmol, 121.94 µL) was added. The resulting mixture was stirred for 3 h, then the reaction mixture was evaporated to dryness to obtain 6-[(2-acetyl-2-azaspiro[3.3]heptan-6-yl)amino]-N-[(2*R*)-2-hydroxy-2-[(3*S*)-7-hydroxy-1,2,3,4-tetrahydroisoquinolin-3-yl]ethyl]-2-(1-piperidyl)pyrimidine-4-carboxamide (0.155 g, 222.86 µmol, 100% yield, 4HCl). ^{1}H NMR(DMSO-d_6, 400 MHz): δ 1.54 (m, 6H), 1.72 (m, 3H), 2.16 (m, 2H), 2.56 (m, 2H), 2.97 (m, 2H), 3.40 (m, 3H), 3.73 (m, 5H), 4.03 (m, 6H), 6.40 (s, 1H), 6.59 (s, 1H), 6.70 (d, 1H), 7.02 (d, 1H), 8.90 (m, 2H), 9.64 (m, 1H). LCMS(ESI): $[M+2H]^+$ m/z: calcd 549.3; found 551.2; Rt = 0.98 min.

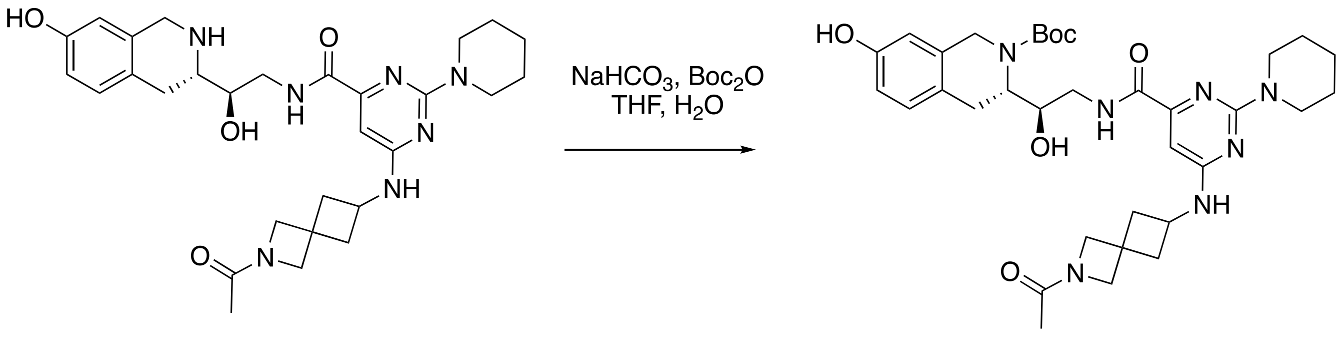

tert-butyl (*S*)-3-((*R*)-2-(6-((2-acetyl-2-azaspiro[3.3]heptan-6-yl)amino)-2-(piperidin-1-yl)pyrimidine-4-carboxamido)-1-hydroxyethyl)-7-hydroxy-3,4-dihydroisoquinoline-2(1*H*)-carboxylate

6-[(2-Acetyl-2-azaspiro[3.3]heptan-6-yl)amino]-N-[(2*R*)-2-hydroxy-2-[(3*S*)-7-hydroxy-1,2,3,4-tetrahydroisoquinolin-3-yl]ethyl]-2-(1-piperidyl)pyrimidine-4-carboxamide (0.155 g, 222.86 µmol, 4HCl) was dissolved in water (2 mL) and sodium bicarbonate (131.05 mg, 1.56 mmol) was added. The resulting mixture was diluted with THF (2 mL) and di-*tert*-butyl dicarbonate (48.64 mg, 222.86 µmol) was added. The reaction mixture was stirred for 18 h. The resulting mixture was extracted with EtOAc (2 x 15 mL) and combined organic layers were washed with brine, dried over Na_2SO_4, filtered, and evaporated to obtain *tert*-butyl (3*S*)-3-[(1*R*)-2-[[6-[(2-acetyl-2-azaspiro[3.3]heptan-6-yl)amino]-2-(1-piperidyl)pyrimidine-4-carbonyl]amino]-1-hydroxy-ethyl]-7-hydroxy-3,4-dihydro-1*H*-isoquinoline-2-carboxylate (0.0843 g, 129.74 µmol, 58% yield). ^{1}H NMR (DMSO-d_6, 400 MHz): δ 1.38 (m, 10H), 1.58 (m, 4H), 2.09 (m, 4H), 2.69 (m, 2H), 2.94 (m, 2H), 3.69 (m, 6H), 4.10 (m, 6H), 4.63 (m, 1H), 5.02 (m, 1H), 6.25 (s, 1H),

6.51 (s, 1H), 6.56 (d, 1H), 6.93 (d, 1H), 7.47 (m, 1H), 8.47 (m, 1H), 9.17 (m, 1H). LCMS(ESI): $[M+H]^+$ m/z: calcd 649.3; found 650.4; Rt = 1.37 min.

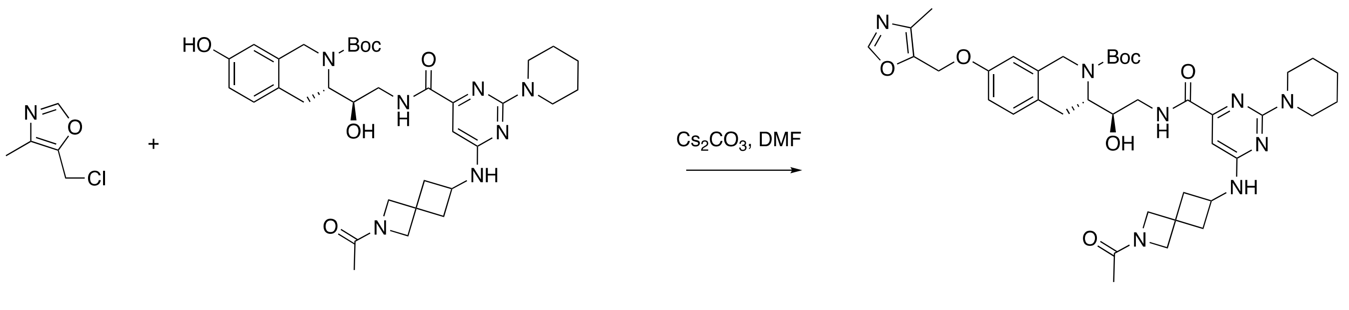

tert-butyl (*S*)-3-((*R*)-2-(6-((2-acetyl-2-azaspiro[3.3]heptan-6-yl)amino)-2-(piperidin-1-yl)pyrimidine-4-carboxamido)-1-hydroxyethyl)-7-((4-methyloxazol-5-yl)methoxy)-3,4-dihydroisoquinoline-2(1*H*)-carboxylate
tert-Butyl (3*S*)-3-[(1*R*)-2-[[6-[(2-acetyl-2-azaspiro[3.3]heptan-6-yl)amino]-2-(1-piperidyl)-pyrimidine-4-carbonyl]amino]-1-hydroxy-ethyl]-7-hydroxy-3,4-dihydro-1*H*-isoquinoline-2-carboxylate (0.0843 g, 129.74 µmol), 5-(chloromethyl)-4-methyl-oxazole (30.52 mg, 181.63 µmol, HCl) and cesium carbonate (169.08 mg, 518.95 µmol) were stirred in DMF (2 mL) and the resulting mixture was heated at 60 °C for 18 h. The resulting mixture was cooled to room temperature and diluted with water (10mL). The resulting mixture was extracted with EtOAc (2 x 15 mL) and the combined organic layers were washed with water (4 x 5 mL) and brine, dried over Na_2SO_4, filtered, and evaporated to obtain *tert*-butyl (3*S*)-3-[(1*R*)-2-[[6-[(2-acetyl-2-azaspiro[3.3]heptan-6-yl)amino]-2-(1-piperidyl)pyrimidine-4-carbonyl]amino]-1-hydroxy-ethyl]-7-[(4-methyloxazol-5-yl)methoxy]-3,4-dihydro-1*H*-isoquinoline-2-carboxylate (65.70 mg, 88.20 µmol, 68% yield). ^{1}H NMR(DMSO-d_6, 500 MHz): δ 1.39 (m, 9H), 1.47 (m, 3H), 1.70 (m, 4H), 1.98 (s, 3H), 2.09 (m, 3H), 2.72 (m, 2H), 3.04 (m, 1H), 3.71 (m, 8H), 4.02 (m, 4H), 4.14 (m, 4H), 4.68 (m, 1H), 5.32 (m, 3H), 6.27 (s, 1H), 6.53 (d, 1H), 6.81 (m, 2H), 7.05 (d, 1H), 7.48 (m, 1H), 8.37 (m, 2H). LCMS(ESI): $[M+H]^+$ m/z: calcd 744.4; found 745.2; Rt = 1.37 min.

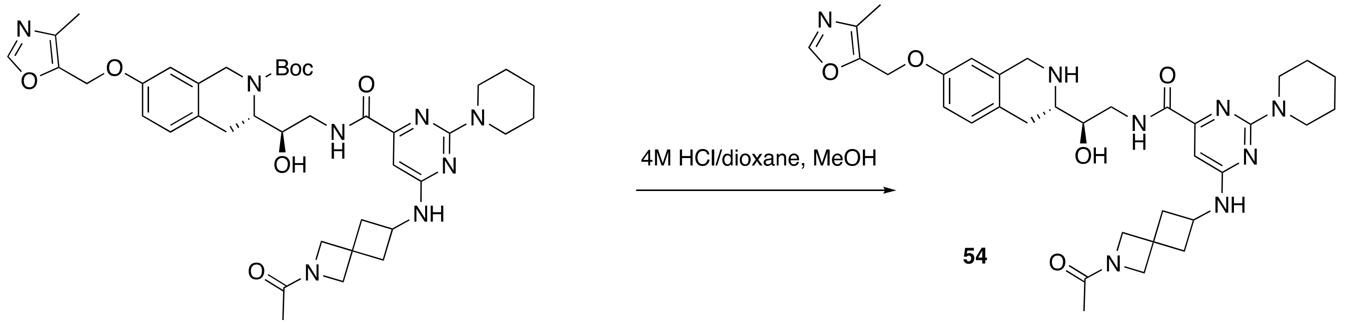

54

6-((2-acetyl-2-azaspiro[3.3]heptan-6-yl)amino)-*N*-((*R*)-2-hydroxy-2-((*S*)-7-((4-methyloxazol-5-yl)methoxy)-1,2,3,4-tetrahydroisoquinolin-3-yl)ethyl)-2-(piperidin-1-yl)pyrimidine-4-carboxamide (**54**). *tert*-Butyl (3*S*)-3-[(1*R*)-2-[[6-[(2-acetyl-2-azaspiro[3.3]heptan-6-yl)amino]-2-(1-piperidyl)-pyrimidine-4-carbonyl]amino]-1-hydroxy-ethyl]-7-[(4-methyloxazol-5-yl)methoxy]-3,4-dihydro-1*H*-isoquinoline-2-carboxylate (65.70 mg, 88.20 µmol) was dissolved in MeOH (2 mL) and 4.0M hydrogen chloride solution in dioxane (529.21 µmol, 24.12 µL) was added thereto. The resulting mixture was stirred for 2 h and then evaporated to dryness. The residue was dissolved in 3 mL of MeOH and 15 mg of scavenger (SiliaMetS Dimercaptotriazine(DMT)) was added and the resulting suspension was stirred for 12 h. The suspension was filtered off and the filtrate was evaporated under reduced pressure. The residue was purified by HPLC (40-90% 0-5 min, water+NH_3-methanole+ NH_3, flow 30 mL/min (loading pump 4 mL/min) to obtain 6-[(2-acetyl-2-azaspiro[3.3]heptan-6-yl)amino]-N-[(2*R*)-2-hydroxy-2-[(3*S*)-7-[(4-methyloxazol-5-yl)methoxy]-1,2,3,4-tetrahydroisoquinolin-3-yl]ethyl]-2-(1-piperidyl)pyrimidine-4-carboxamide, **54** (0.0251 g, 38.93 µmol, 44% yield). ^{1}H NMR (500 MHz, DMSO-d_6) δ 1.56 (m, 4H), 1.65 (m, 2H), 1.72 (m, 3H), 2.01 (m, 1H), 2.17 (m, 5H), 2.57 (m, 3H), 2.75 (m, 2H), 3.38 (m, 1H), 3.55 (m, 2H), 3.72 (m, 4H), 3.79 (m, 1H), 3.90 (m, 3H), 4.05 (m, 1H), 4.17 (m, 1H), 4.23 (m, 1H), 4.88 (d, 1H), 4.97 (s, 2H), 6.30 (s, 1H), 6.60 (s, 1H), 6.69 (d, 1H), 6.96 (d, 1H), 7.31 (m, 1H), 8.02 (s, 1H), 8.54 (m, 1H). LCMS(ESI): $[M+H]^+$ m/z: calcd 644.3; found 645.4; Rt = 1.03 min. HRMS (ESI, + vw ion) m/z calcd for $C_{34}H_{44}N_8O_5$ $[M+H^+]$ 644.34347, found 644.3424.

Compound 2

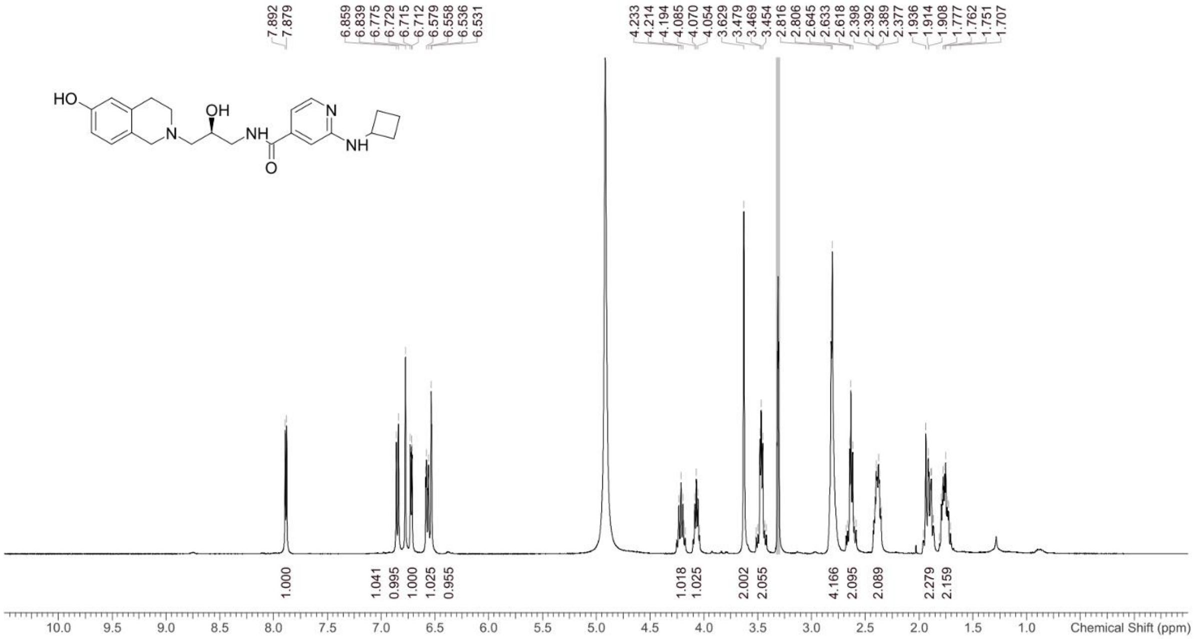

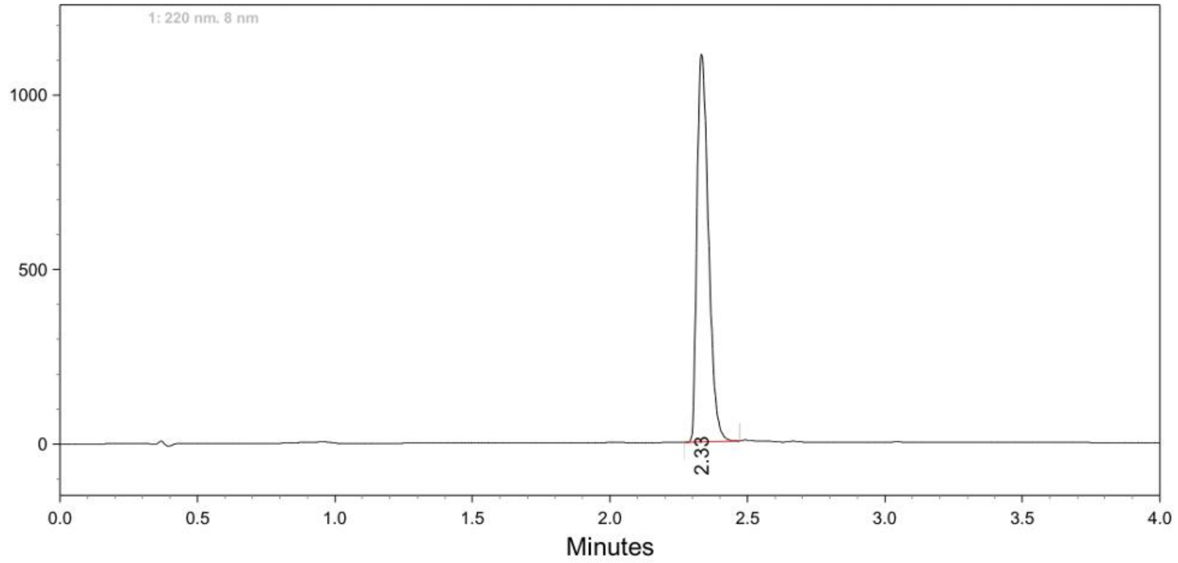

1: 220 nm, 8 nm

Retention Time	*Height*	*Area*	*Area Percent*
2.33	1104650	3218577	100.00

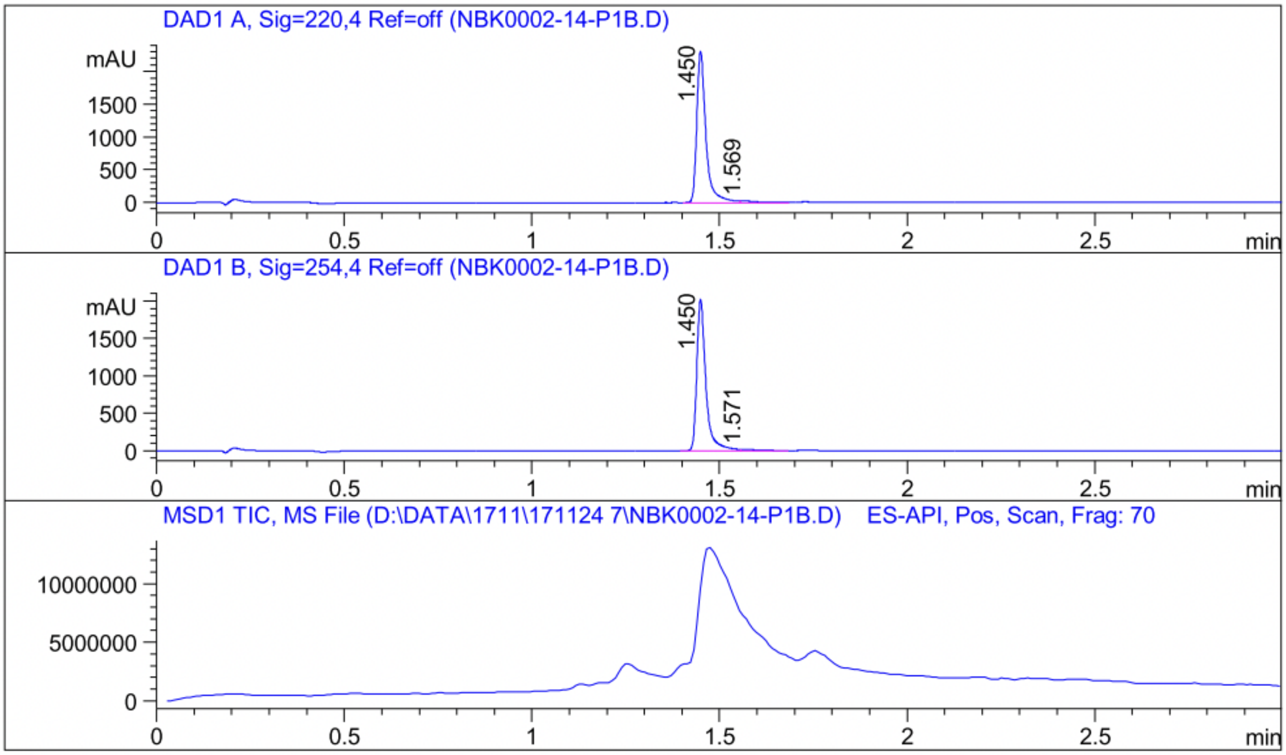

Report

===

Signal ->: DAD1 A, Sig=220,4 Ref=off

#	Meas. Ret.	Height	Width	Area	Area %
1	1.450	2287.748	0.026	3934.717	97.957
2	1.569	25.390	0.044	82.064	2.043

Signal ->: DAD1 B, Sig=254,4 Ref=off

#	Meas. Ret.	Height	Width	Area	Area %
1	1.450	2002.132	0.026	3428.679	97.872
2	1.571	21.167	0.047	74.548	2.128

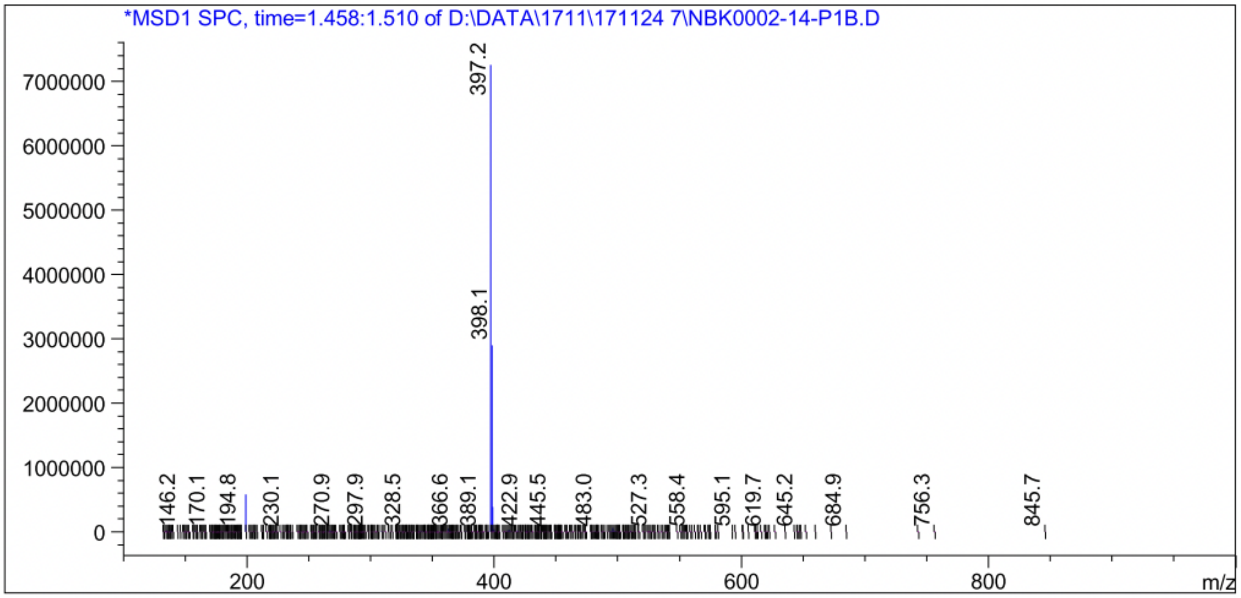
*MSD1 SPC, time=1.458:1.510 of D:\DATA\1711\171124 7\NBK0002-14-P1B.D
7000000
6000000
5000000
4000000
3000000
2000000
1000000
0
397.2
398.1
146.2
170.1
194.8
230.1
270.9
297.9
328.5
366.6
389.1
422.9
445.5
483.0
527.3
558.4
595.1
619.7
645.2
684.9
756.3
845.7
200
400
600
800
m/z

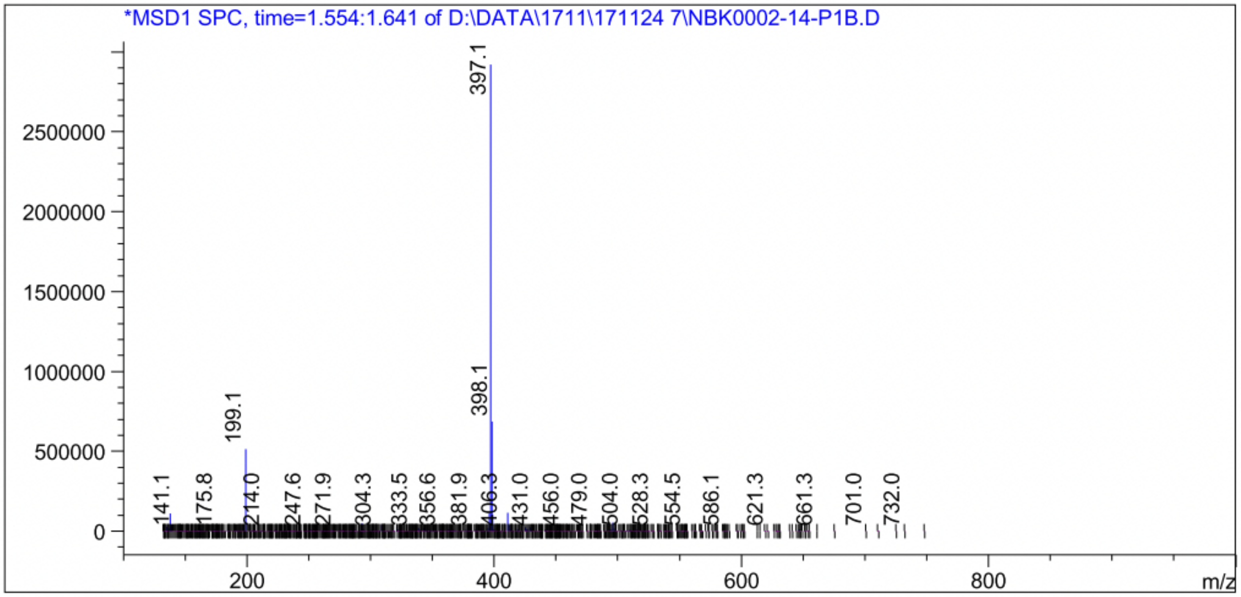
*MSD1 SPC, time=1.554:1.641 of D:\DATA\1711\171124 7\NBK0002-14-P1B.D
2500000
2000000
1500000
1000000
500000
0
397.1
398.1
199.1
141.1
175.8
214.0
247.6
271.9
304.3
333.5
356.6
381.9
406.3
431.0
456.0
479.0
504.0
528.3
554.5
586.1
621.3
661.3
701.0
732.0
200
400
600
800
m/z

Method : Column: Chiralcel OJ-3 100×4.6mm I.D., 3μm
Mobile phase: A: CO_2 B:ethanol (0.1%ethanolamine)
Gradient: from 5% to 40% of B in 4.5min and hold 40% for 2.5 min, then 5% of B for 1min
Flow rate: 2.8mL/min Column temperature:40 C

===

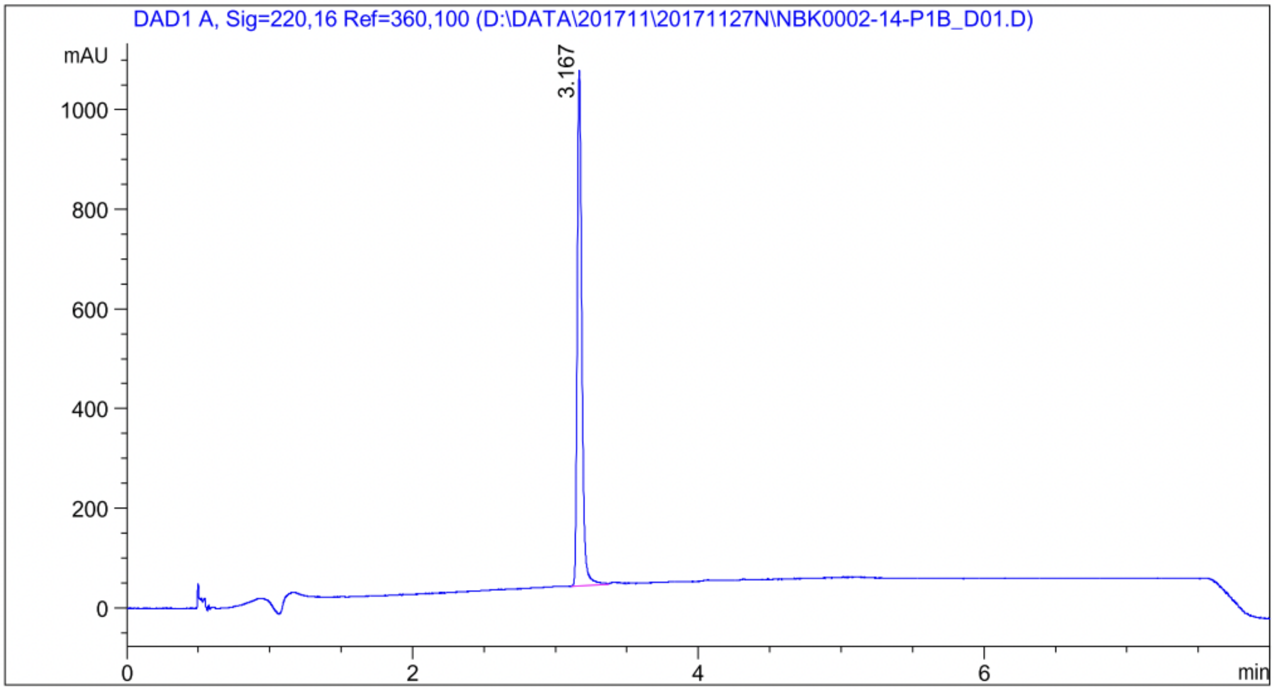

===

DAD1 A, Sig=220,16 Ref=360,100

#	Meas. Ret. Time	Height	Height %	Width	Area	Area %
1	3.167	1037.742	100.000	0.037	2324.470	100.000

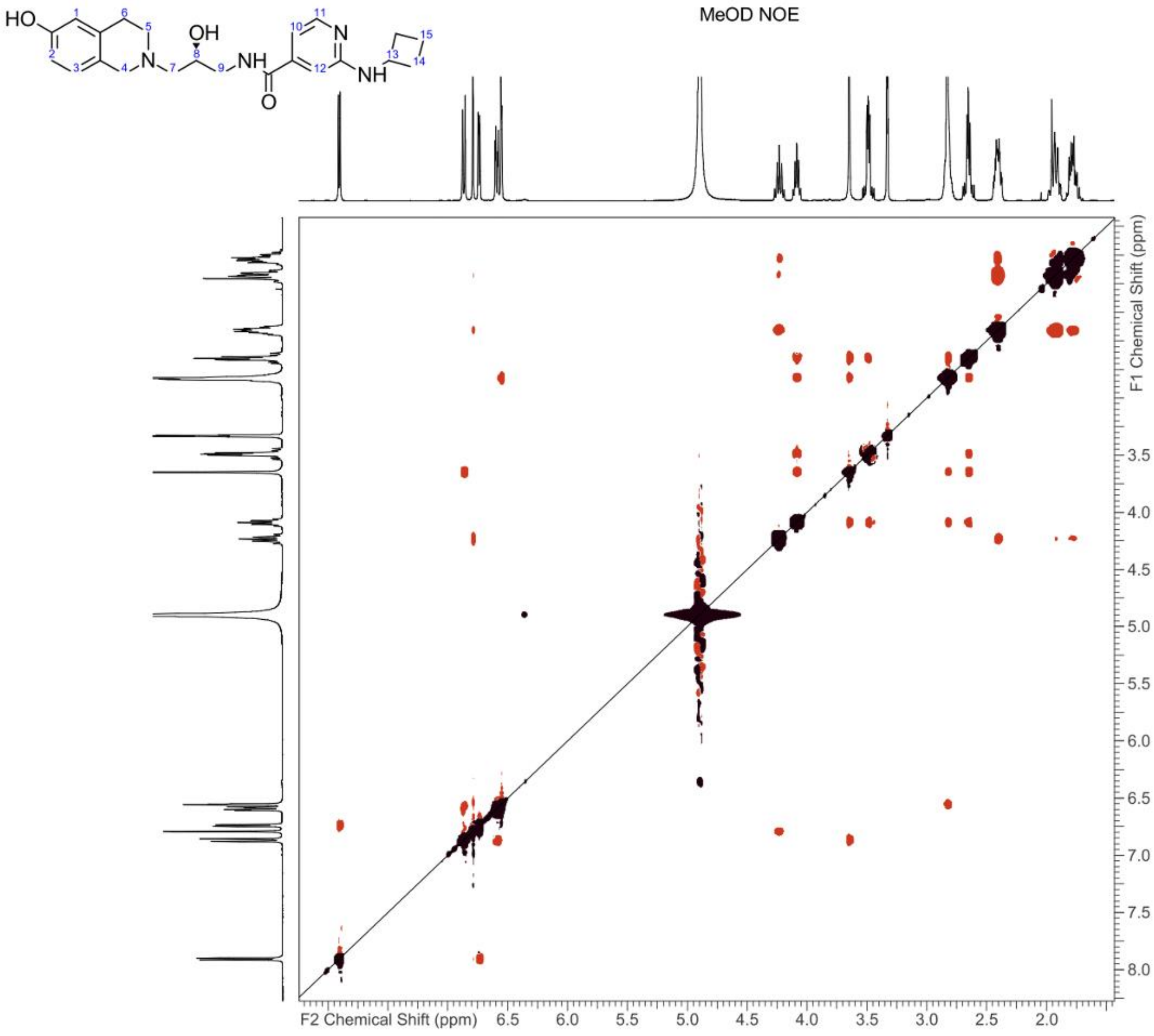

HO
OH
NH
N
NH
O
1
2
3
4
5
6
7
8
9
10
11
12
13
14
15
MeOD NOE
F1 Chemical Shift (ppm)
F2 Chemical Shift (ppm)
6.5
6.0
5.5
5.0
4.5
4.0
3.5
3.0
2.5
2.0
7.0
7.5
8.0

Compound 3

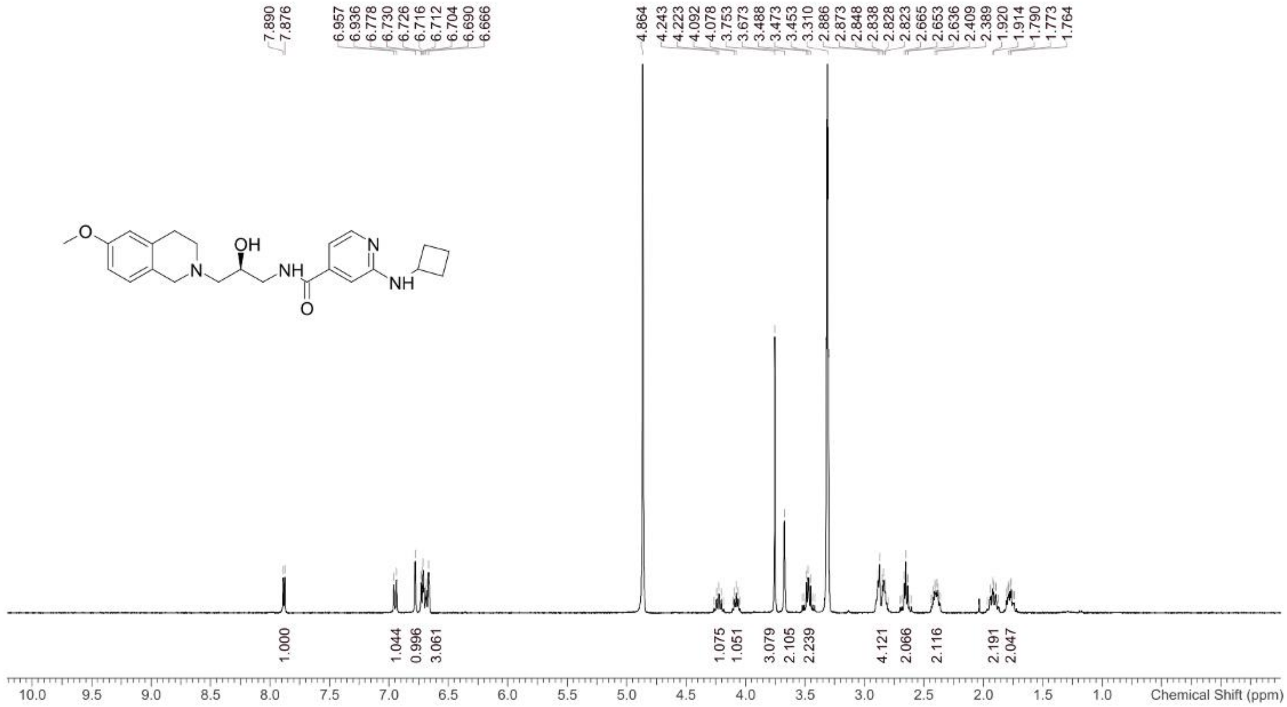

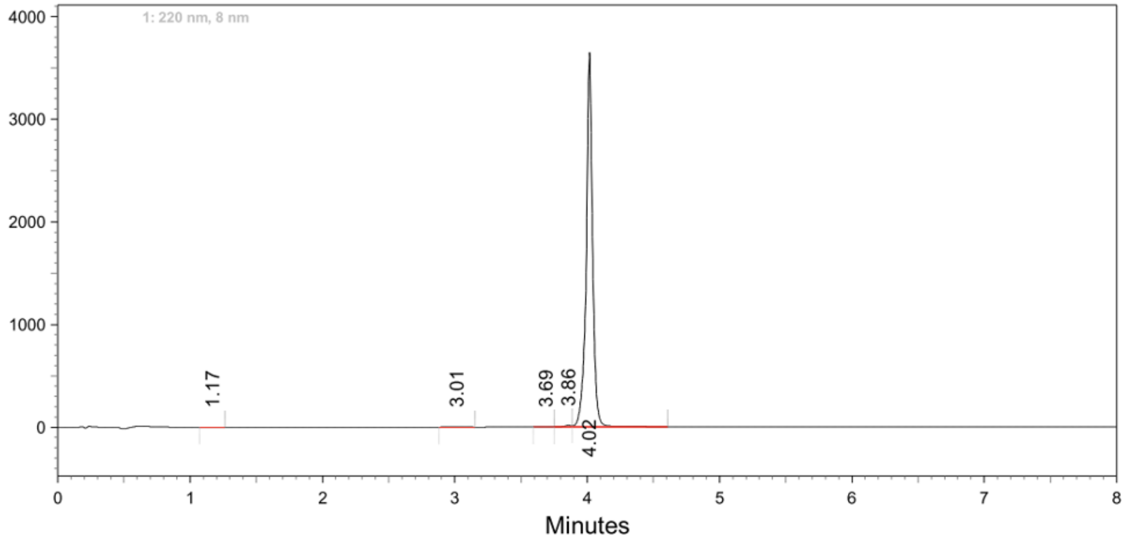

1: 220 nm, 8 nm

Retention Time	***Height***	***Area***	***Area Percent***
1.17	1970	10831	0.09
3.01	4160	28235	0.23
3.69	2892	11131	0.09
3.86	15591	57177	0.46
4.02	3554900	12314523	99.14

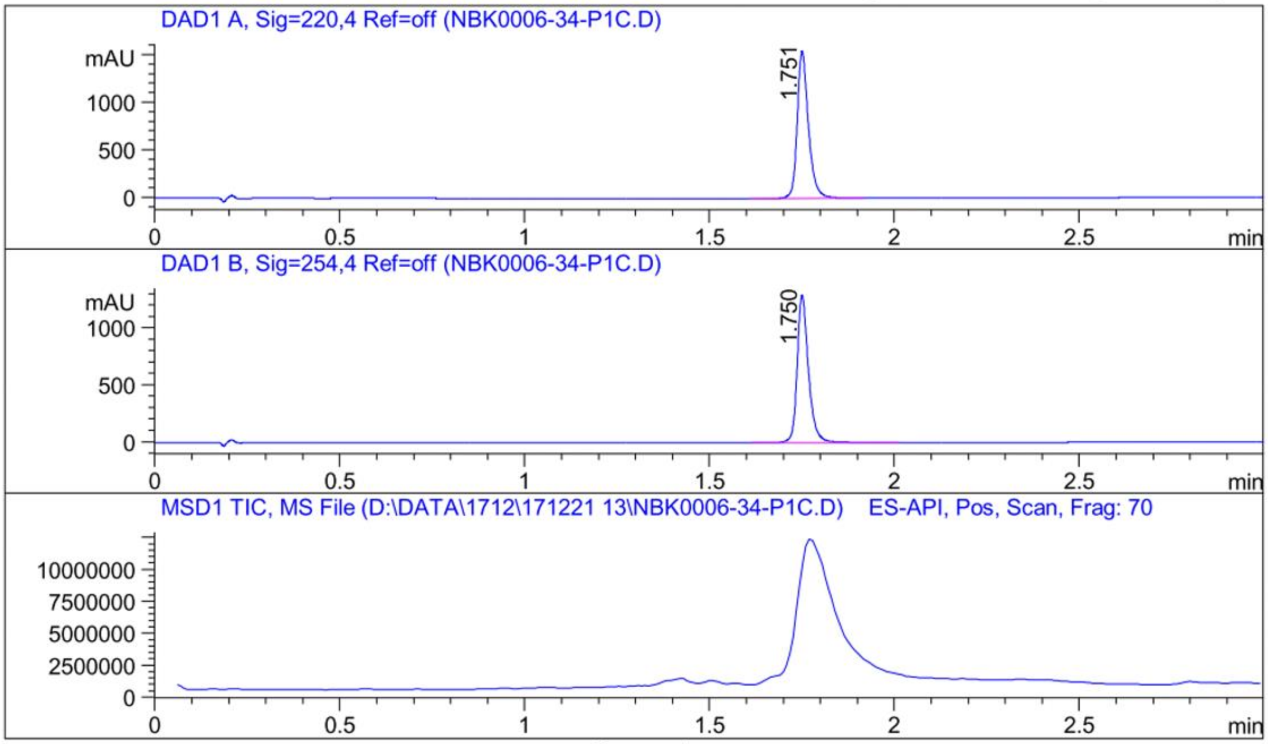

Report

==

Signal ->: DAD1 A, Sig=220,4 Ref=off

#	Meas. Ret.	Height	Width	Area	Area %
1	1.751	1537.892	0.032	3276.935	100.000

Signal ->: DAD1 B, Sig=254,4 Ref=off

#	Meas. Ret.	Height	Width	Area	Area %
1	1.750	1288.838	0.032	2761.055	100.000

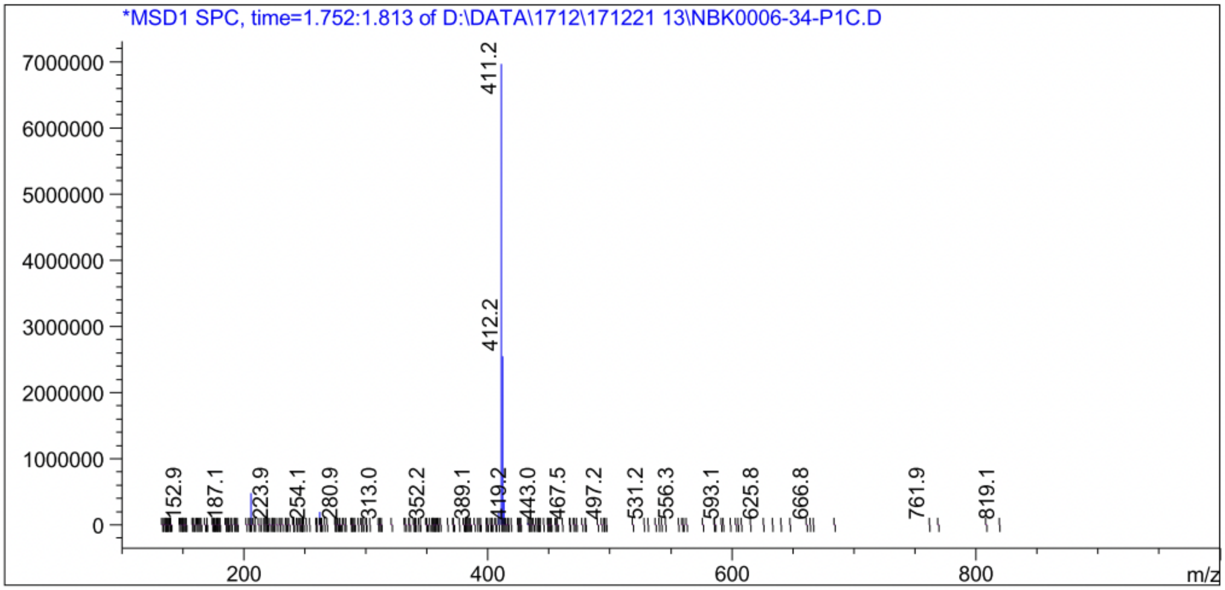


```
Method                : Column: Chiralpak AD-3 50×3mm I.D., 3um
                        Mobile phase: A: CO2  B:ethanol (0.05% DEA)
                        Gradient:from 5% to 40% of B in 2.5 min and hold 40%
                        for 0.35 min, then from 40% to 5% of B for 0.15 min
                        Flow rate: 2.5mL/min    Column temperature:40 C
```

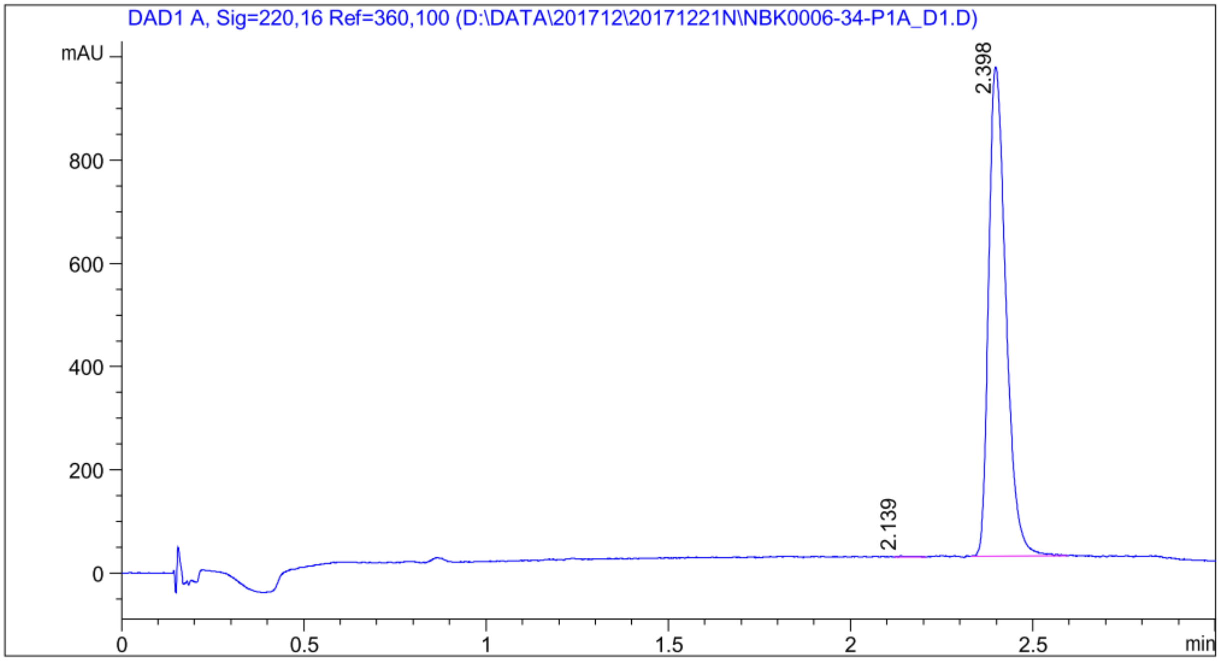

DAD1 A, Sig=220,16 Ref=360,100

#	Meas. Ret. Time	Height	Height %	Width	Area	Area %
1	2.139	2.586	0.272	0.039	6.030	0.192
2	2.398	949.078	99.728	0.055	3126.936	99.808

Compound 4

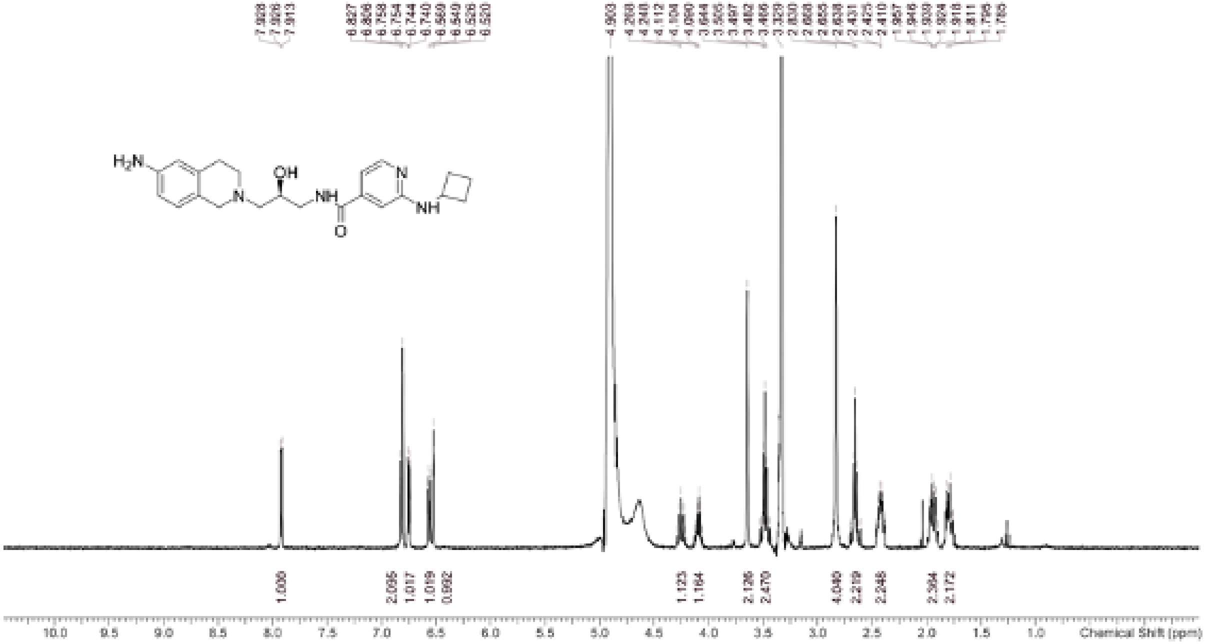

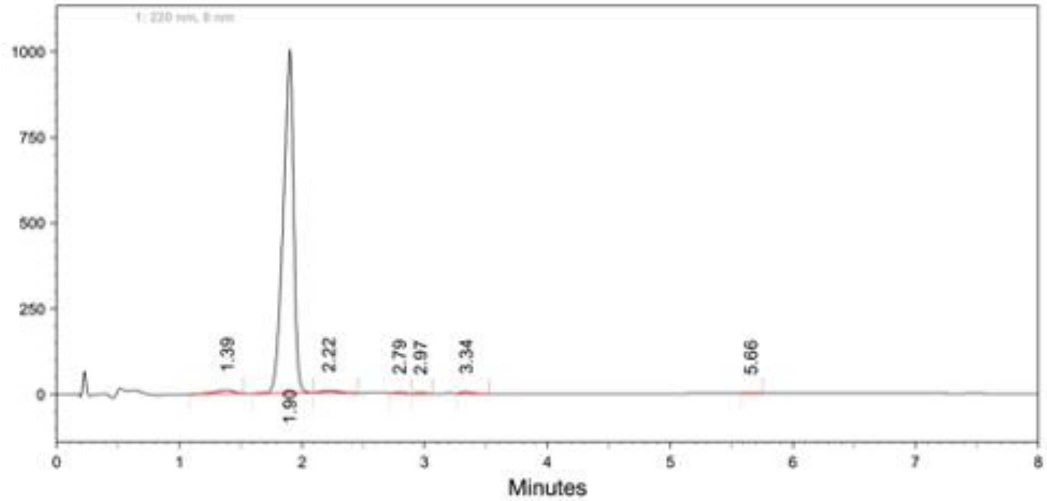

1: 220 nm, 8 nm

Retention Time	*Height*	*Area*	*Area Percent*
1.39	11231	120396	1.99
1.90	1000549	5797304	95.98
2.22	7173	67327	1.11
2.79	3613	14384	0.24
2.97	2768	11151	0.18
3.34	5090	23948	0.40
5.66	1326	5305	0.09

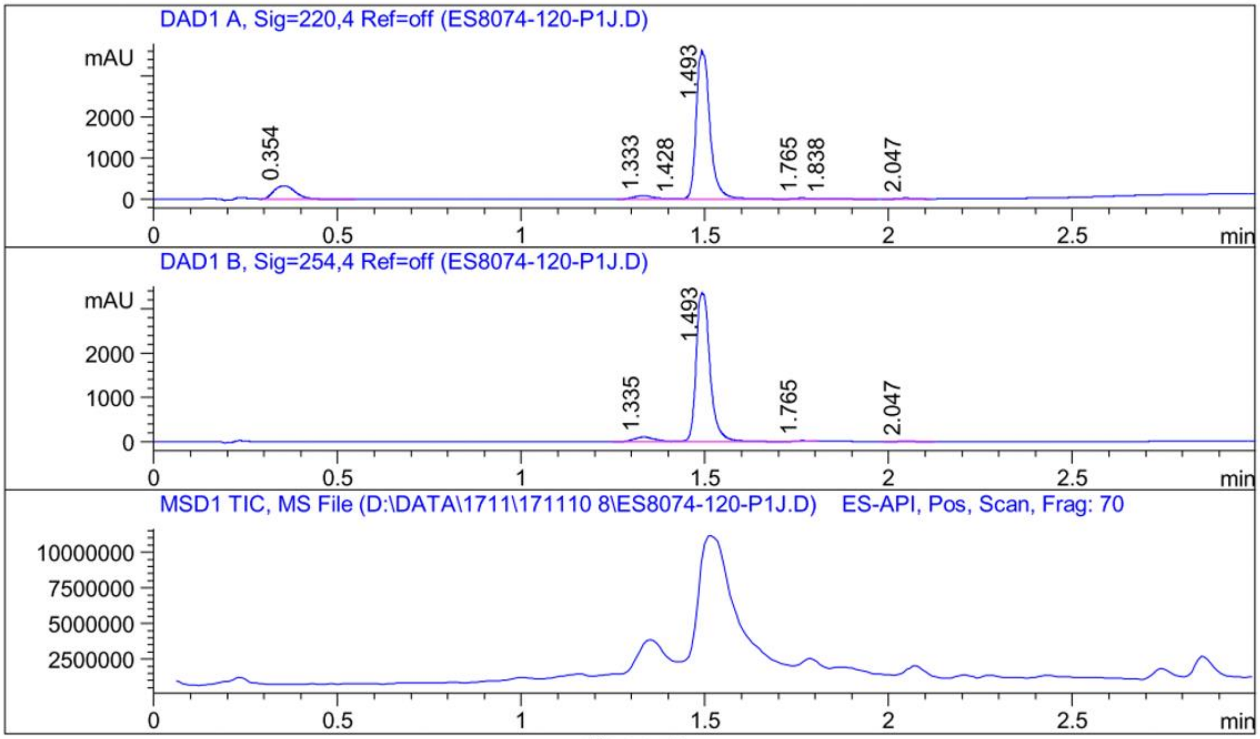

Report

Signal ->: DAD1 A, Sig=220,4 Ref=off

#	Meas. Ret.	Height	Width	Area	Area %
1	0.354	317.864	0.066	1285.646	11.511
2	1.333	86.496	0.057	323.657	2.898
3	1.428	20.786	0.030	45.320	0.406
4	1.493	3607.507	0.040	9359.929	83.801
5	1.765	21.261	0.036	53.233	0.477
6	1.838	15.029	0.049	52.215	0.467
7	2.047	20.751	0.036	49.225	0.441

Signal ->: DAD1 B, Sig=254,4 Ref=off

#	Meas. Ret.	Height	Width	Area	Area %
1	1.335	109.062	0.060	436.837	4.655
2	1.493	3339.704	0.041	8854.619	94.352
3	1.765	17.811	0.032	38.898	0.414
4	2.047	22.207	0.037	54.266	0.578

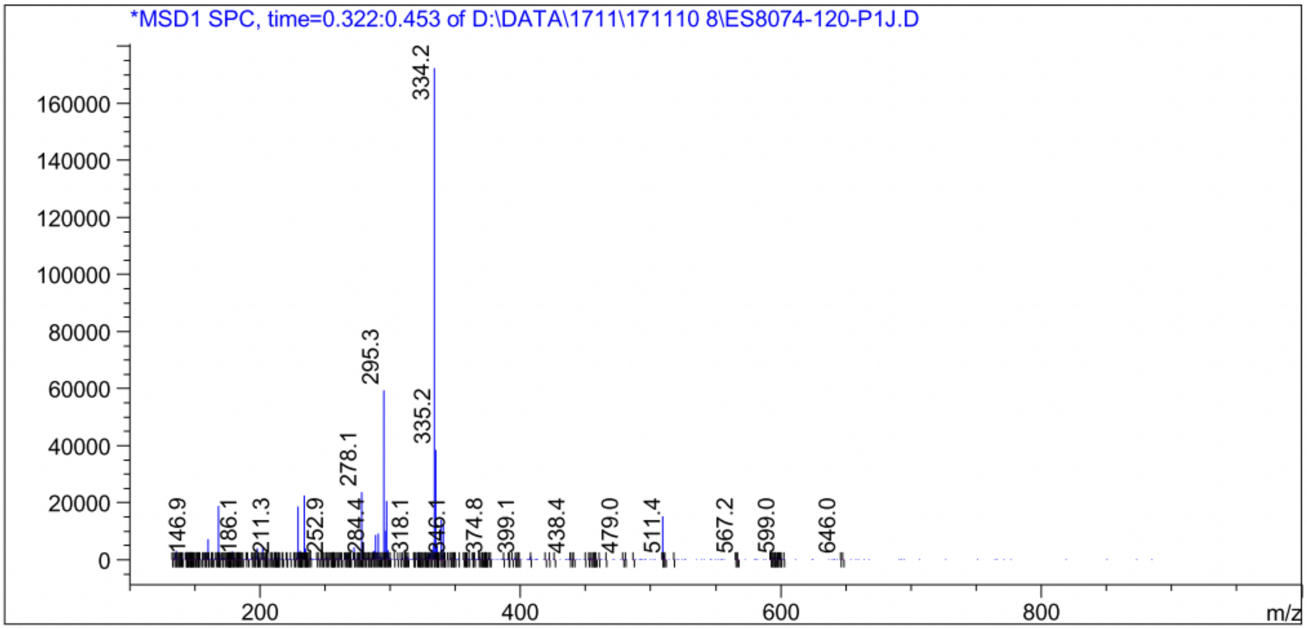
*MSD1 SPC, time=0.322:0.453 of D:\DATA\1711\171110 8\ES8074-120-P1J.D
334.2
295.3
335.2
278.1
146.9
186.1
211.3
252.9
284.4
318.1
346.1
374.8
399.1
438.4
479.0
511.4
567.2
599.0
646.0
m/z

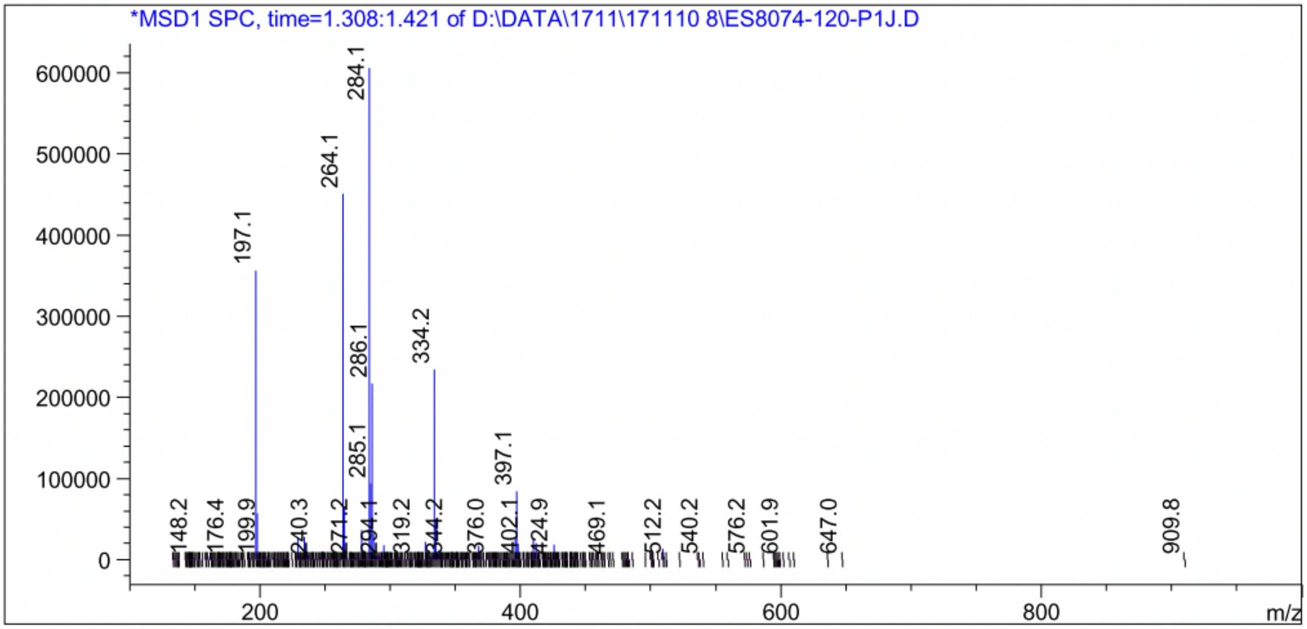
*MSD1 SPC, time=1.308:1.421 of D:\DATA\1711\171110 8\ES8074-120-P1J.D
284.1
264.1
197.1
334.2
286.1
285.1
397.1
148.2
176.4
199.9
240.3
271.2
294.1
319.2
344.2
376.0
402.1
424.9
469.1
512.2
540.2
576.2
601.9
647.0
909.8
m/z

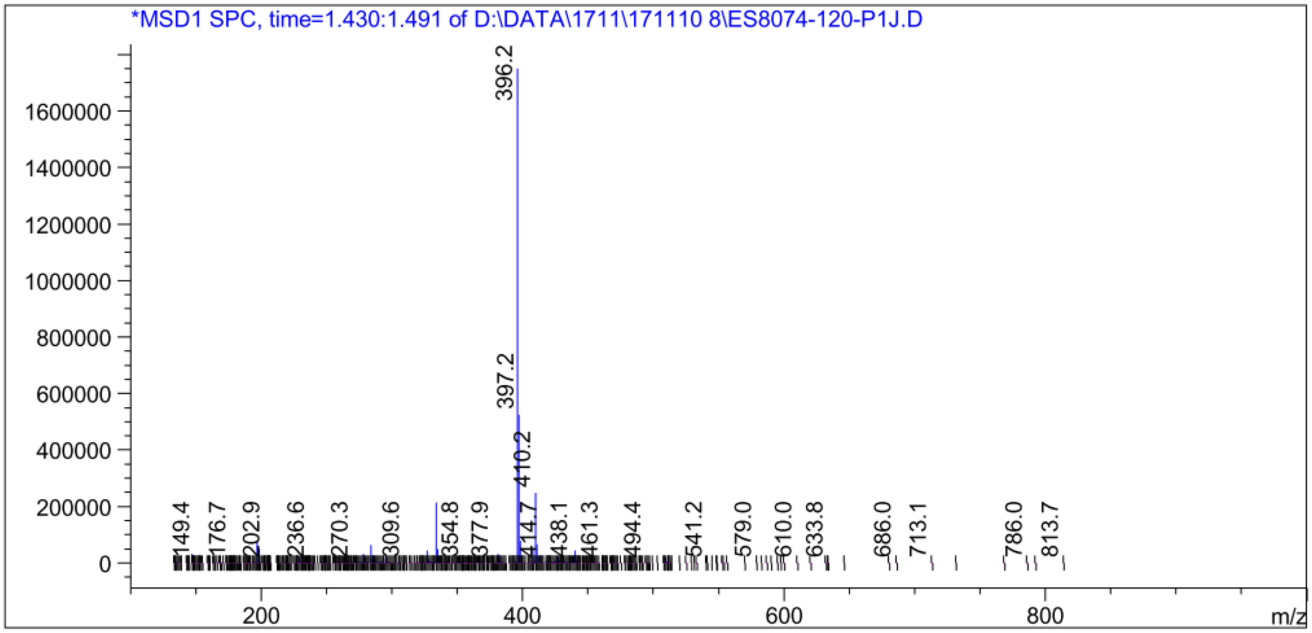
*MSD1 SPC, time=1.430:1.491 of D:\DATA\1711\171110 8\ES8074-120-P1J.D
1600000
1400000
1200000
1000000
800000
600000
400000
200000
0
396.2
397.2
410.2
149.4
176.7
202.9
236.6
270.3
309.6
354.8
377.9
414.7
438.1
461.3
494.4
541.2
579.0
610.0
633.8
686.0
713.1
786.0
813.7
200
400
600
800
m/z

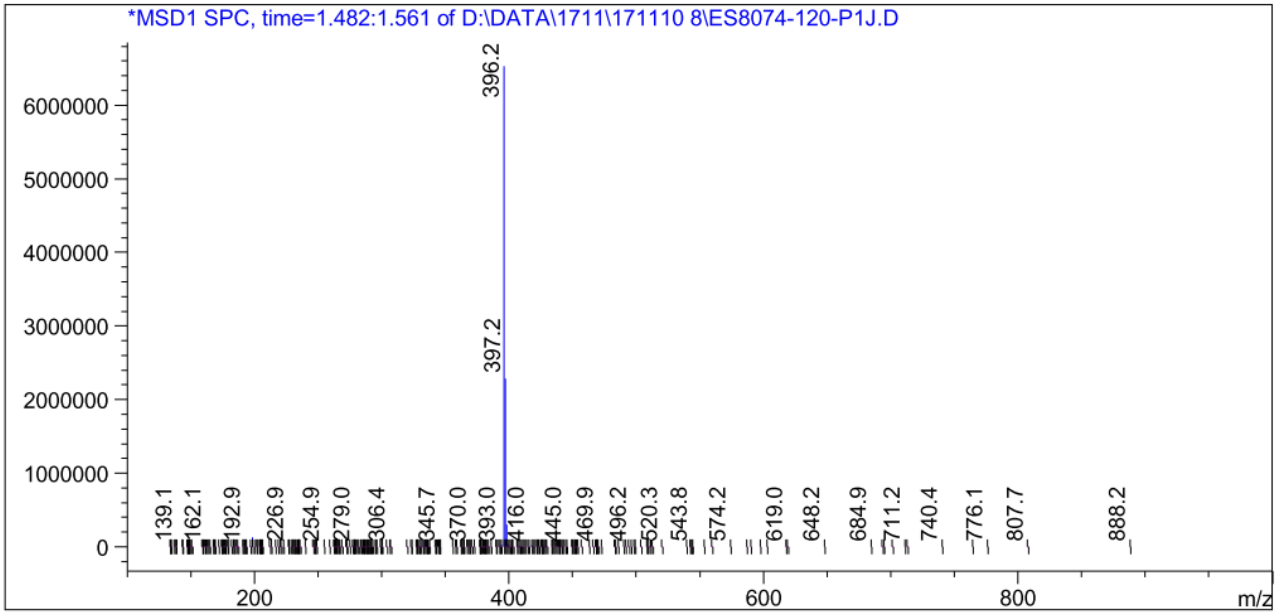
*MSD1 SPC, time=1.482:1.561 of D:\DATA\1711\171110 8\ES8074-120-P1J.D
396.2
397.2
139.1
162.1
192.9
226.9
254.9
279.0
306.4
345.7
370.0
393.0
416.0
445.0
469.9
496.2
520.3
543.8
574.2
619.0
648.2
684.9
711.2
740.4
776.1
807.7
888.2
m/z

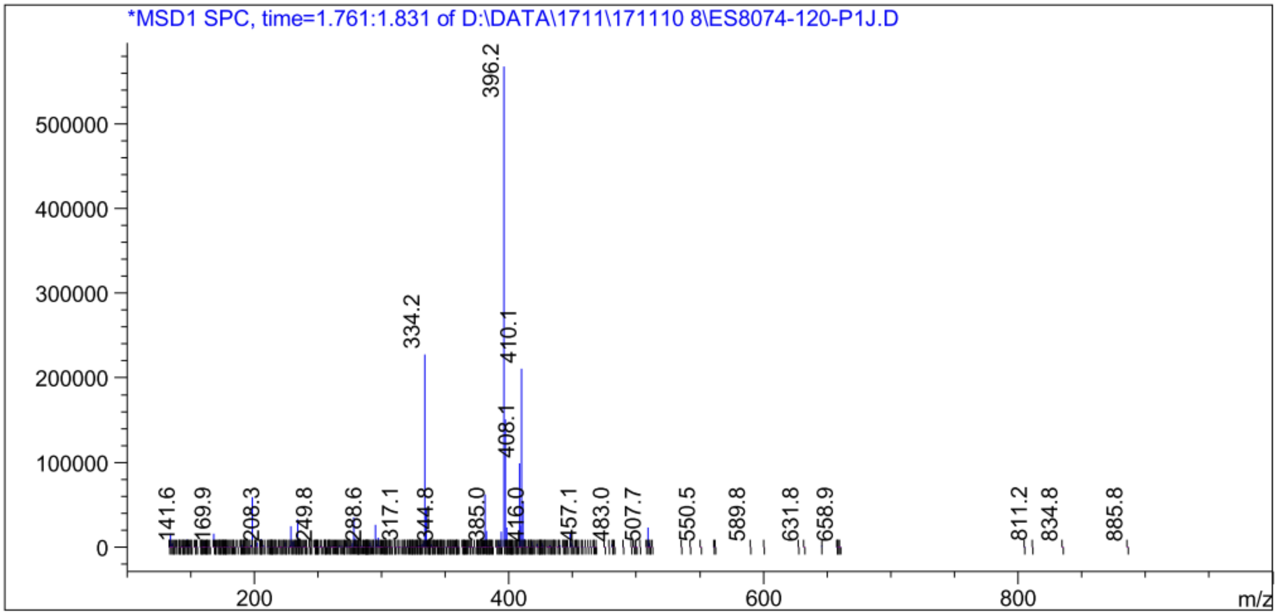
*MSD1 SPC, time=1.761:1.831 of D:\DATA\1711\171110 8\ES8074-120-P1J.D
396.2
334.2
410.1
408.1
141.6
169.9
208.3
249.8
288.6
317.1
344.8
385.0
416.0
457.1
483.0
507.7
550.5
589.8
631.8
658.9
811.2
834.8
885.8
m/z

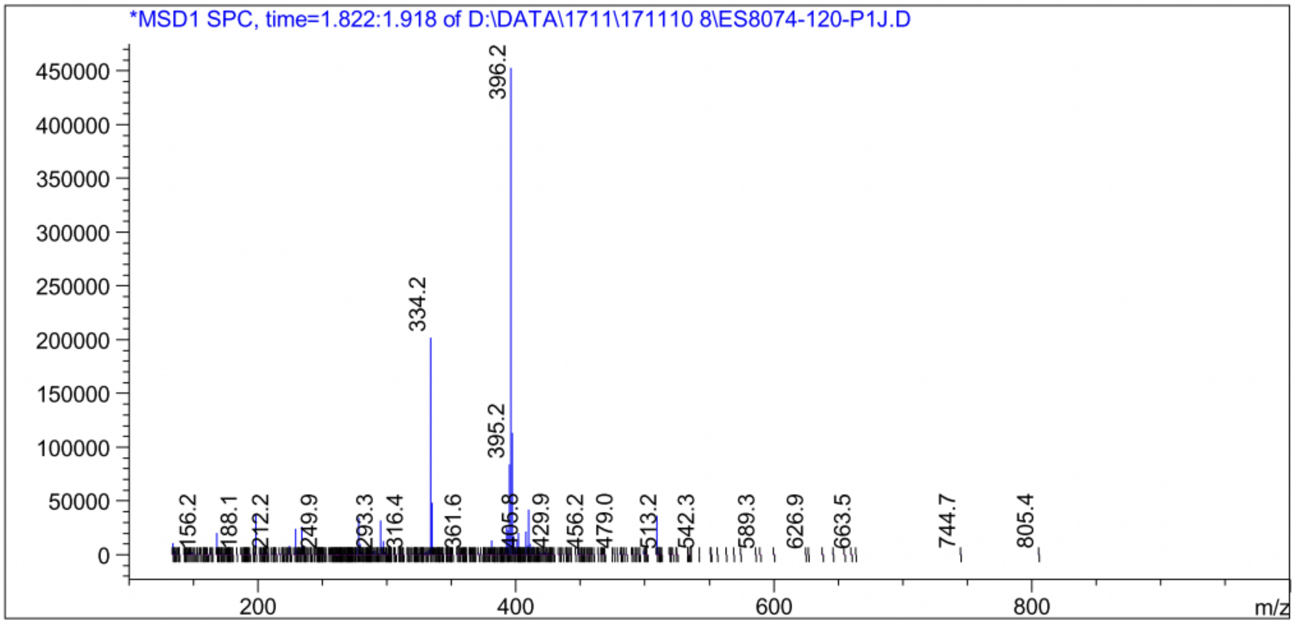
*MSD1 SPC, time=1.822:1.918 of D:\DATA\1711\171110 8\ES8074-120-P1J.D
396.2
334.2
395.2
156.2
188.1
212.2
249.9
293.3
316.4
361.6
405.8
429.9
456.2
479.0
513.2
542.3
589.3
626.9
663.5
744.7
805.4
450000
400000
350000
300000
250000
200000
150000
100000
50000
0
200
400
600
800
m/z

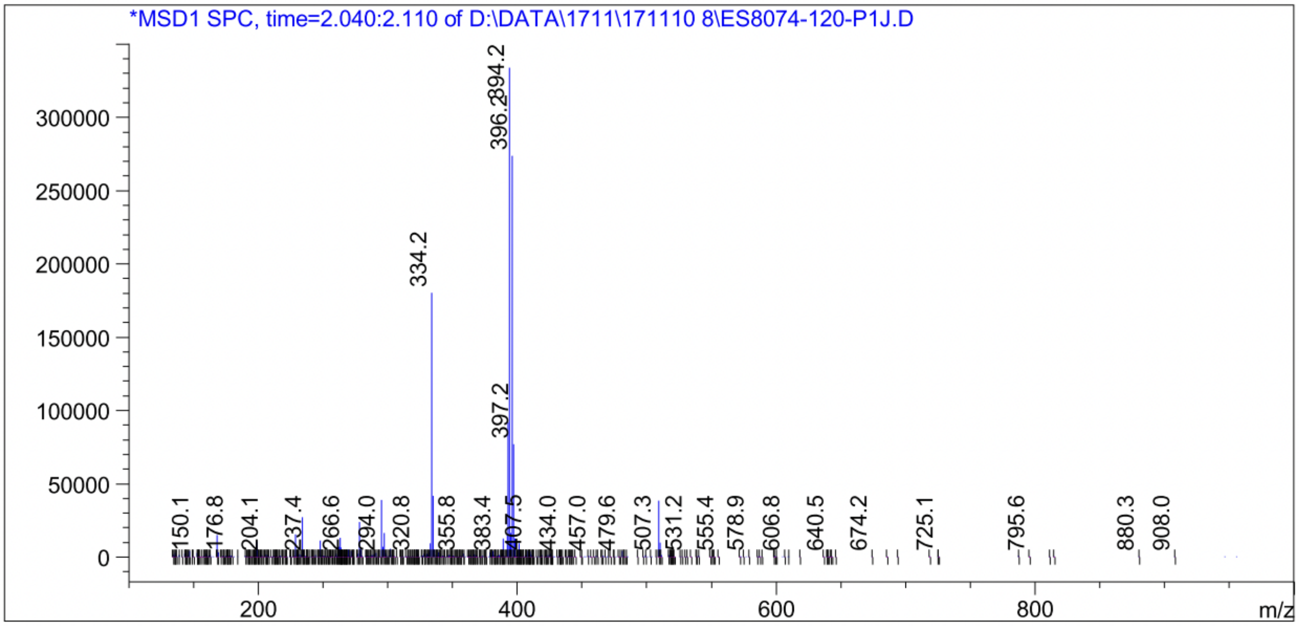
*MSD1 SPC, time=2.040:2.110 of D:\DATA\1711\171110 8\ES8074-120-P1J.D
394.2
396.2
334.2
397.2
150.1
176.8
204.1
237.4
266.6
294.0
320.8
355.8
383.4
407.5
434.0
457.0
479.6
507.3
531.2
555.4
578.9
606.8
640.5
674.2
725.1
795.6
880.3
908.0
300000
250000
200000
150000
100000
50000
0
200
400
600
800
m/z

```
Method             :Column: Chiralpak AD-3 50*4.6mm I.D., 3um
                    Mobile phase: A:CO2  B:iso-propanol (0.05% DEA)
                    Gradient: hold 5% for 0.2 min,then from 5% to 40% of B
                    in 1.4 min and hold 40% for 1.05 min,  then 5% of B for
                    0.35 min
                    Flow rate: 4mL/min   Column temp:40 C
```

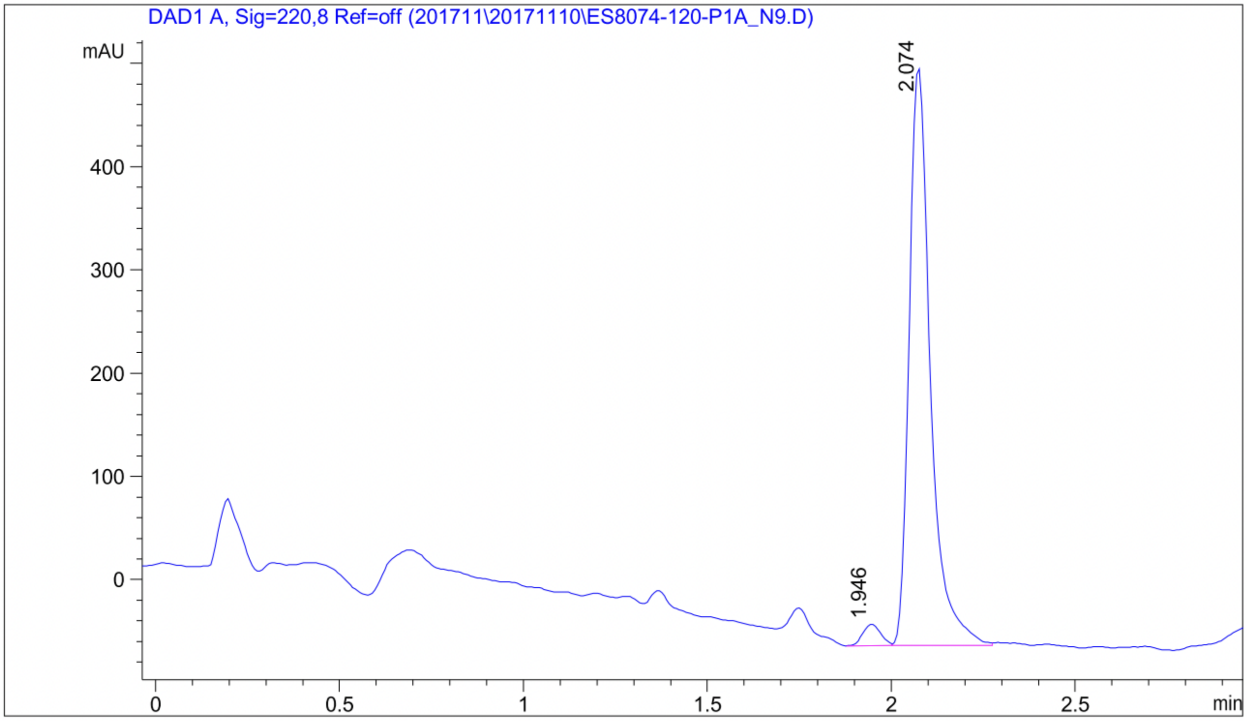

Signal 1 : DAD1 A, Sig=220,8 Ref=off

Peak	Meas. Ret. Time	Height	Height %	Width	Area	Area %
1	1.946	20.828	3.578	0.056	70.609	3.031
2	2.074	561.280	96.422	0.063	2258.720	96.969

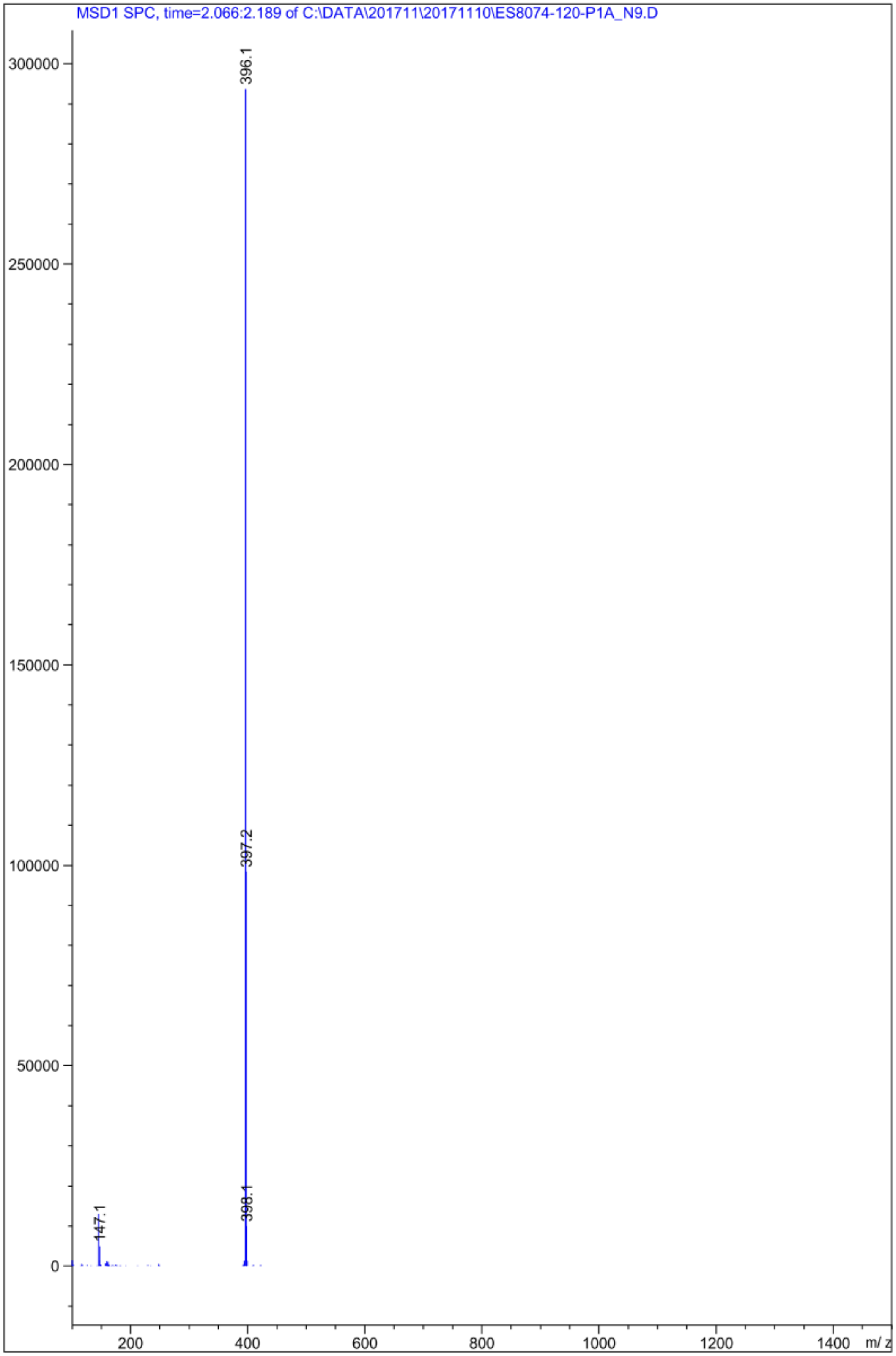

MSD1 SPC, time=2.066:2.189 of C:\DATA\201711\20171110\ES8074-120-P1A_N9.D
300000
250000
200000
150000
100000
50000
0
396.1
397.2
398.1
147.1
200
400
600
800
1000
1200
1400
m/z

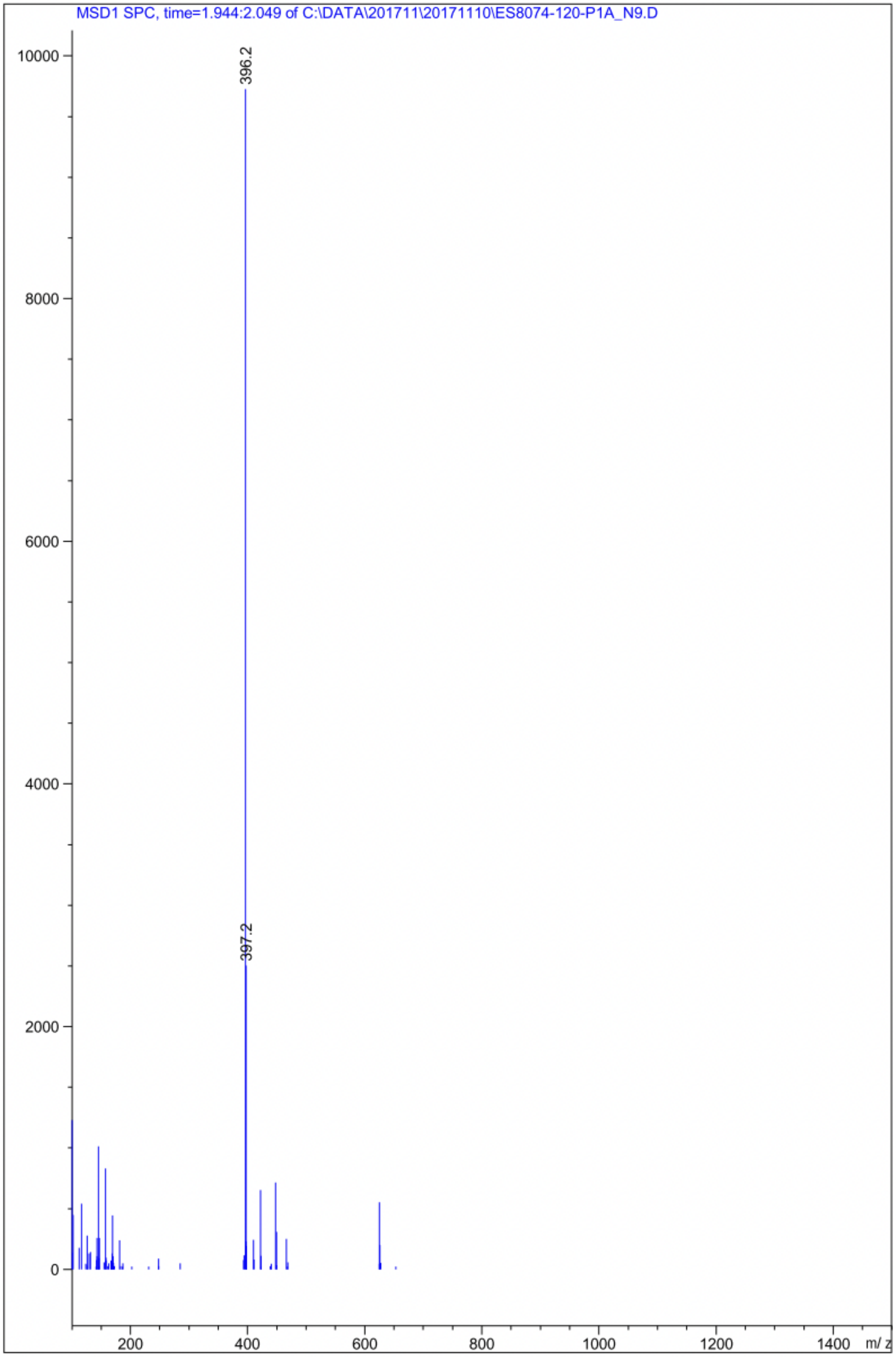

MSD1 SPC, time=1.944:2.049 of C:\DATA\201711\20171110\ES8074-120-P1A_N9.D
396.2
397.2
10000
8000
6000
4000
2000
0
200
400
600
800
1000
1200
1400
m/z

Compound 5

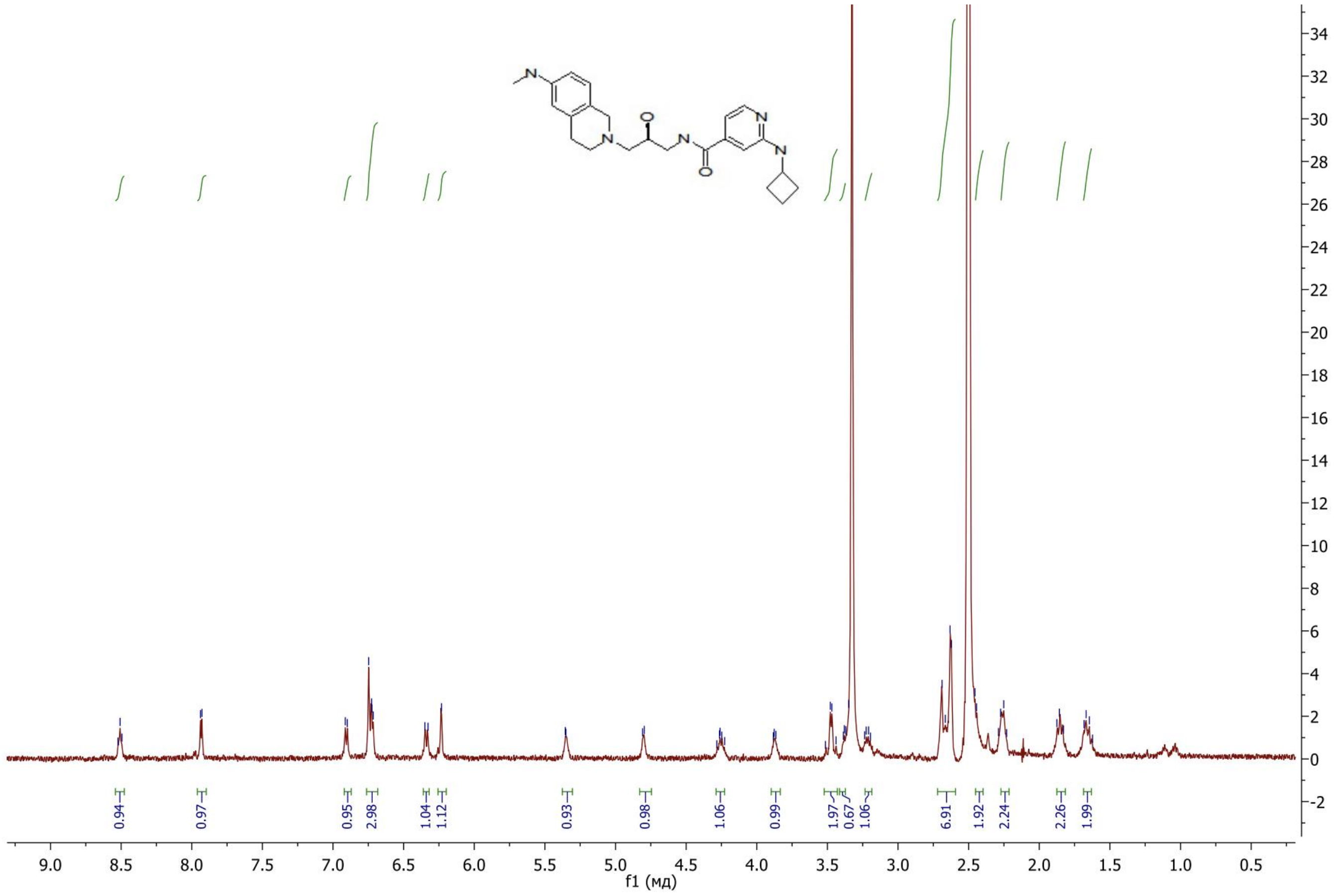
f1 (мд)

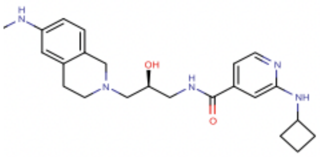

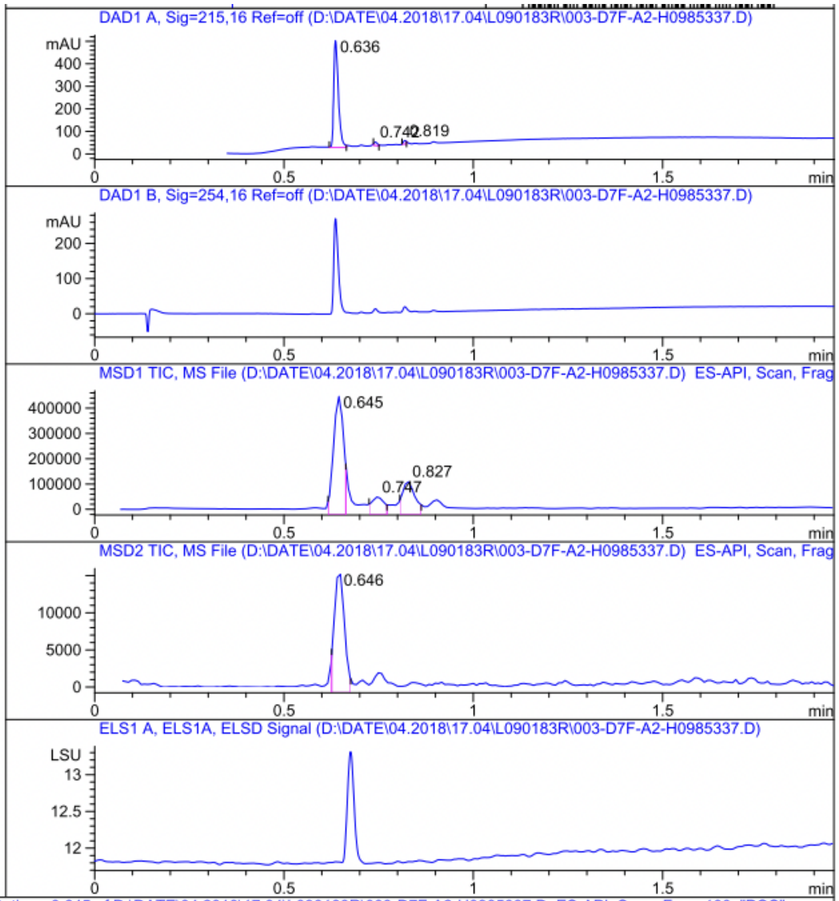

Mol Wt 409.52

Exact Mass 409.29

#	Time	Area%
1	0.636	95.72
2	0.742	2.28
3	0.819	2.00

RT 0.645

RT 0.747

RT 0.827

RT 0.646

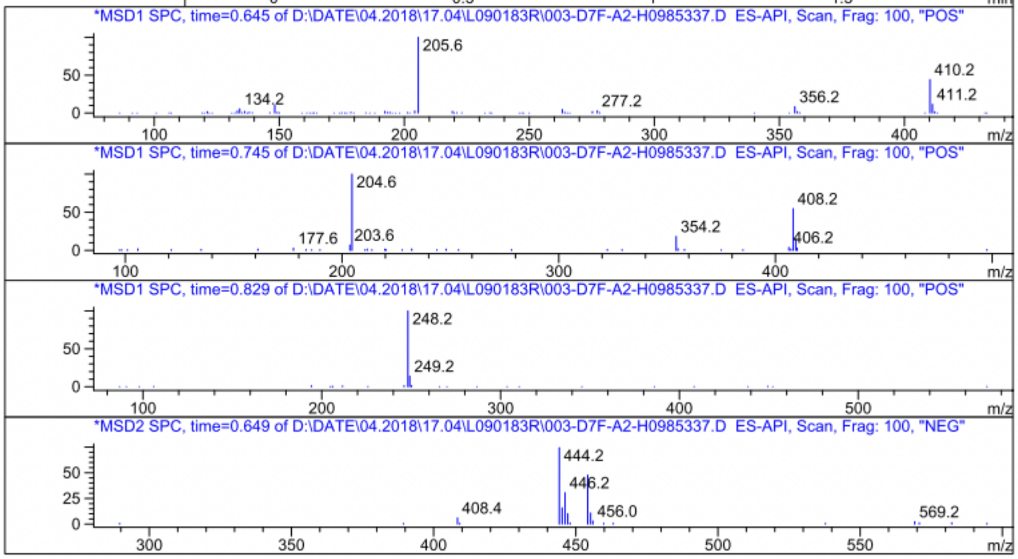

Compound 6

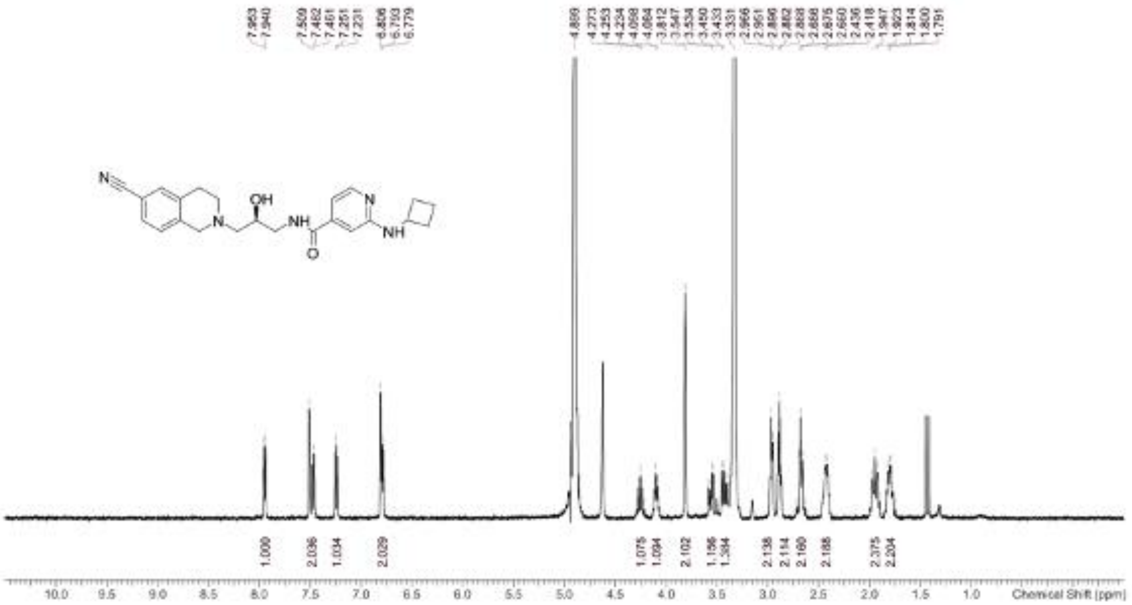

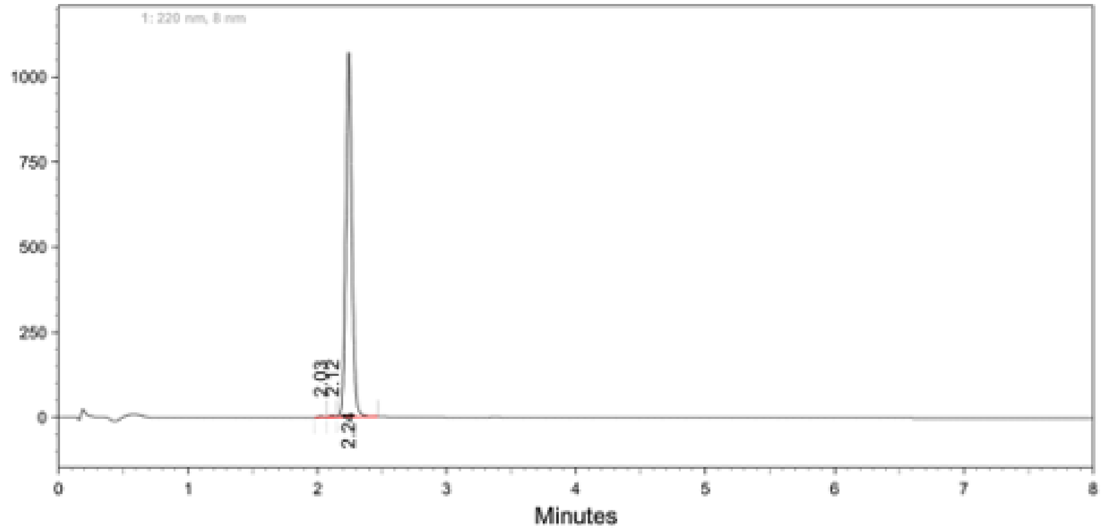

1: 220 nm, 8 nm

Retention Time	*Height*	*Area*	*Area Percent*
2.03	2870	7655	0.22
2.12	3726	11184	0.32
2.24	1061415	3518809	99.47

Instrument : LCMS AR
A:,Xtimate,2.1*30mm,3um
B:XBridge Shield, 2.1*50mm,5um
Confidential. For research only NOT for regulatory filing

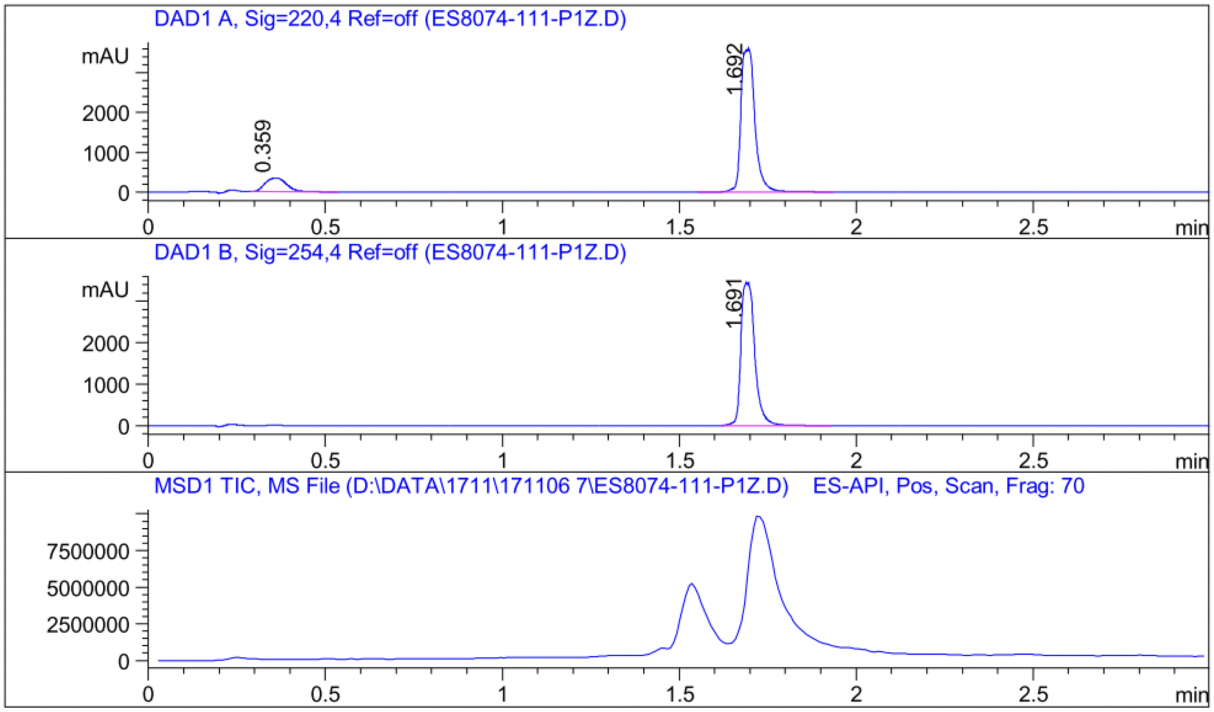

Report

===

Signal ->: DAD1 A, Sig=220,4 Ref=off

#	Meas. Ret.	Height	Width	Area	Area %
1	0.359	347.155	0.069	1449.431	12.709
2	1.692	3579.499	0.044	9955.270	87.291

Signal ->: DAD1 B, Sig=254,4 Ref=off

#	Meas. Ret.	Height	Width	Area	Area %
1	1.691	3419.409	0.043	9445.609	100.000

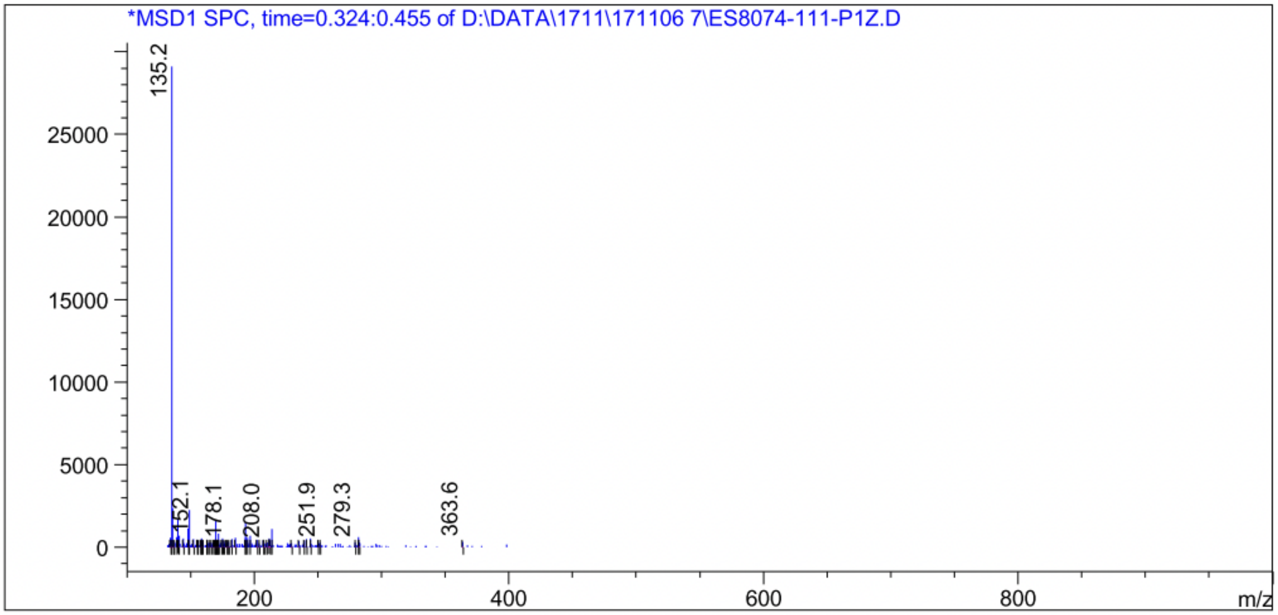
*MSD1 SPC, time=0.324:0.455 of D:\DATA\1711\171106 7\ES8074-111-P1Z.D
135.2
152.1
178.1
208.0
251.9
279.3
363.6
25000
20000
15000
10000
5000
0
200
400
600
800
m/z

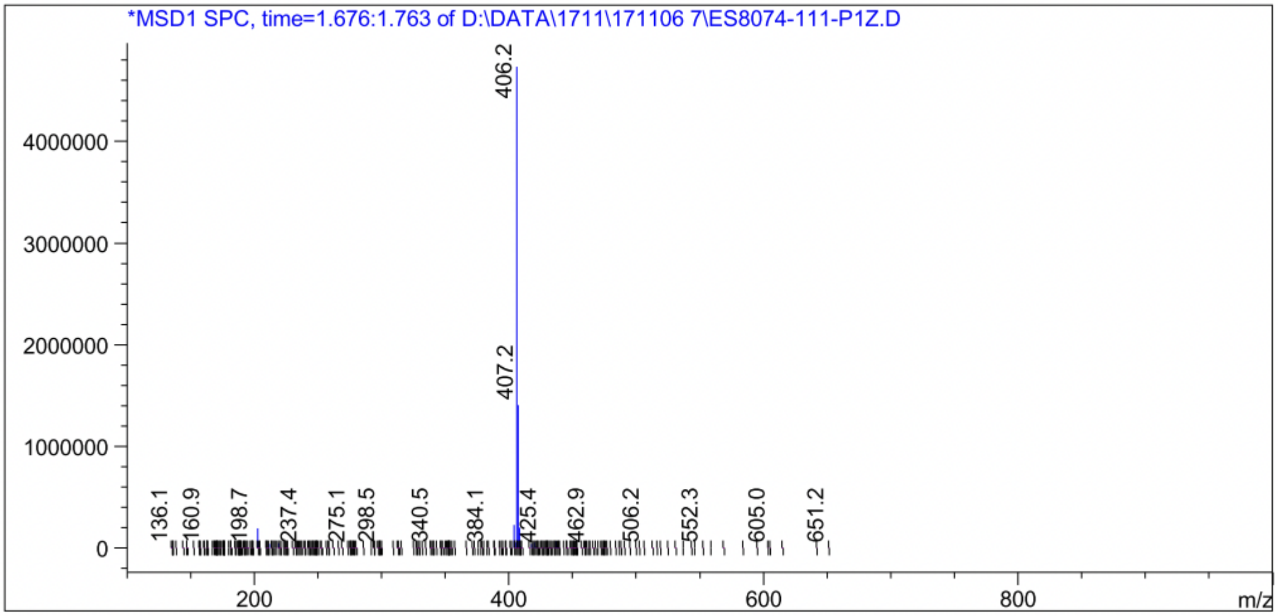
*MSD1 SPC, time=1.676:1.763 of D:\DATA\1711\171106 7\ES8074-111-P1Z.D
406.2
407.2
136.1
160.9
198.7
237.4
275.1
298.5
340.5
384.1
425.4
462.9
506.2
552.3
605.0
651.2
4000000
3000000
2000000
1000000
0
200
400
600
800
m/z

Method :Column: Chiralpak AD-3 50*4.6mm I.D., 3um
Mobile phase: A:CO2 B:ethanol (0.05% DEA)
Gradient: hold 5% for 0.2 min,then from 5% to 40% of B in 1.4 min and hold 40% for 1.05 min, then 5% of B for 0.35 min
Flow rate: 4mL/min Column temp:40 C

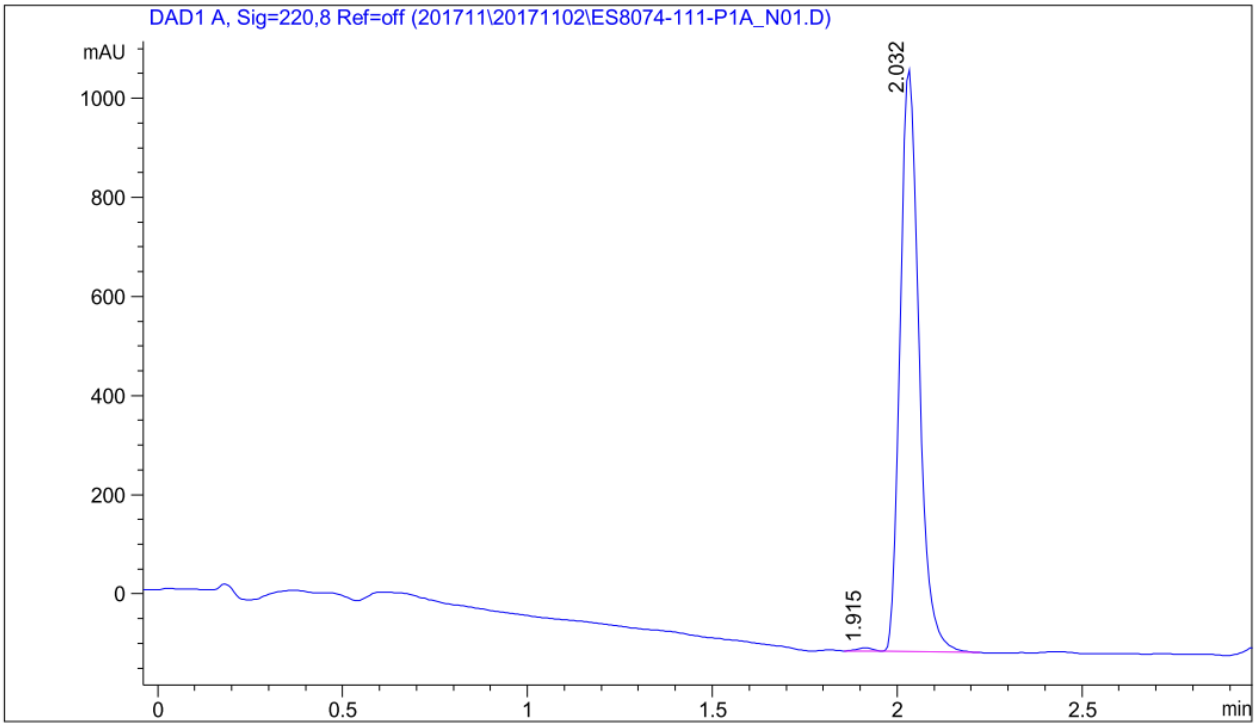

Signal 1 : DAD1 A, Sig=220,8 Ref=off

Peak	Meas. Ret. Time	Height	Height %	Width	Area	Area %
1	1.915	6.804	0.573	0.051	20.800	0.495
2	2.032	1181.353	99.427	0.059	4184.395	99.505

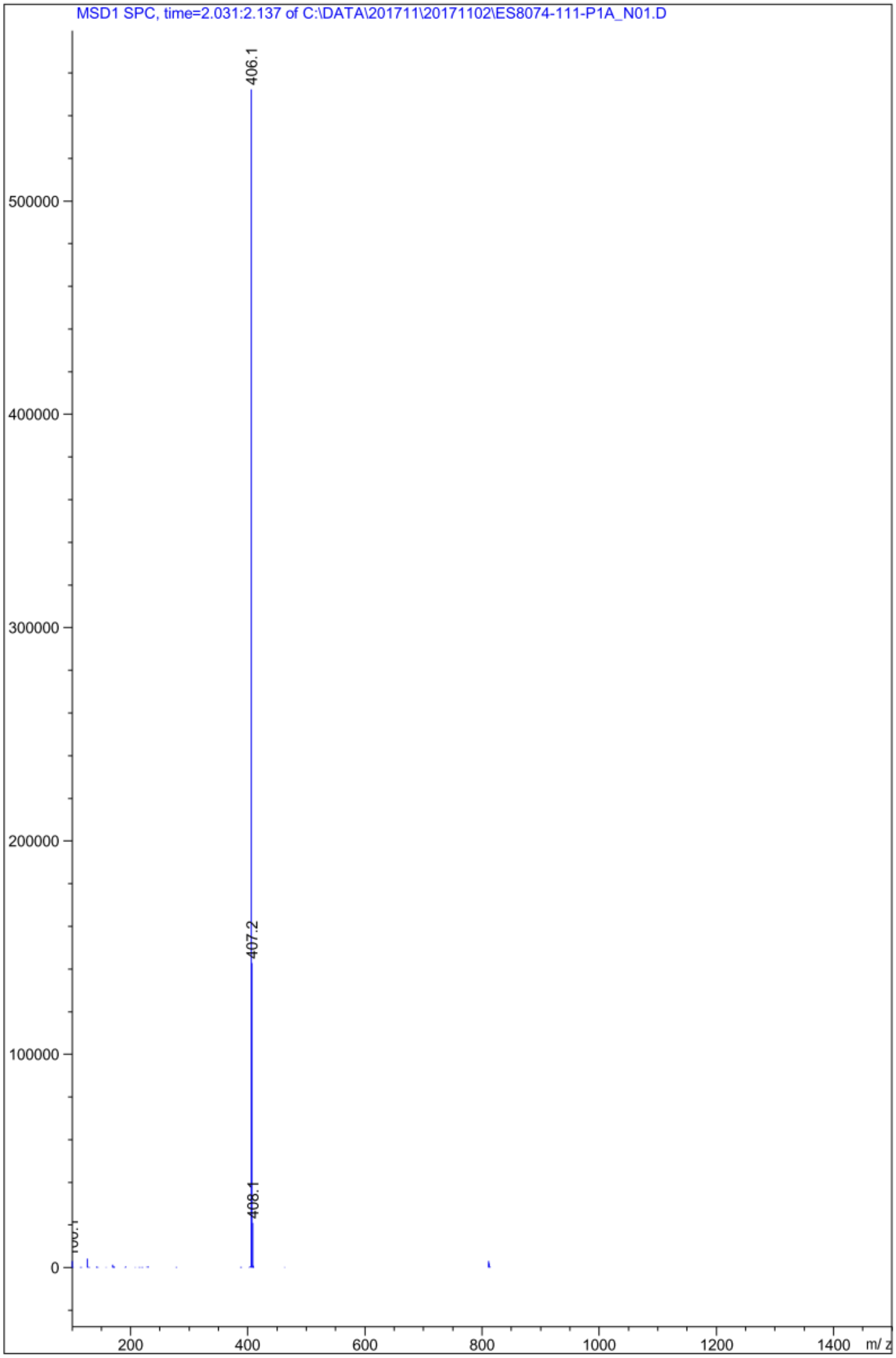
MSD1 SPC, time=2.031:2.137 of C:\DATA\201711\20171102\ES8074-111-P1A_N01.D
406.1
407.2
408.1
500000
400000
300000
200000
100000
0
200
400
600
800
1000
1200
1400
m/z

Compound 7

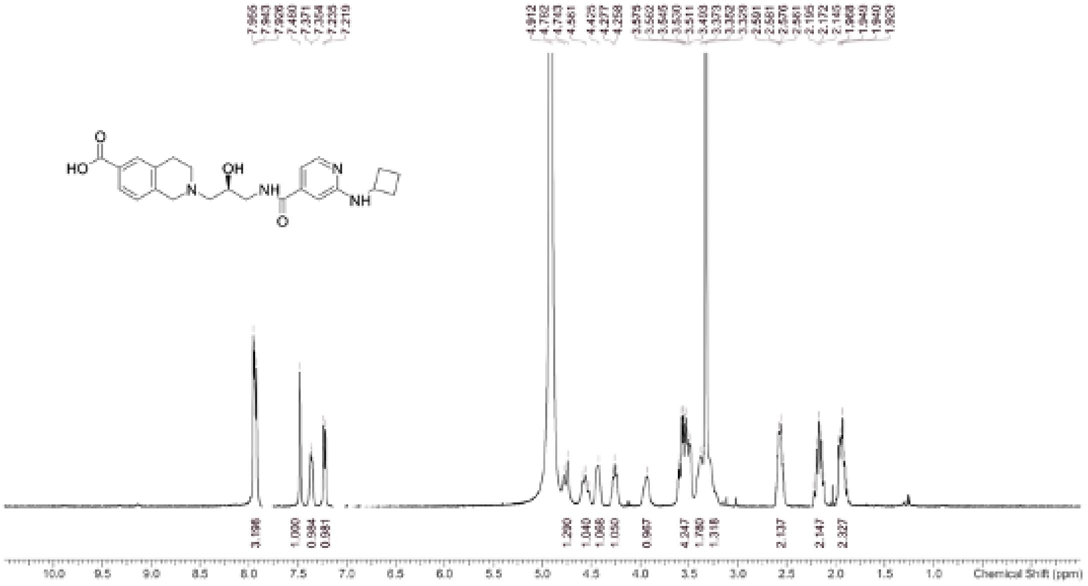

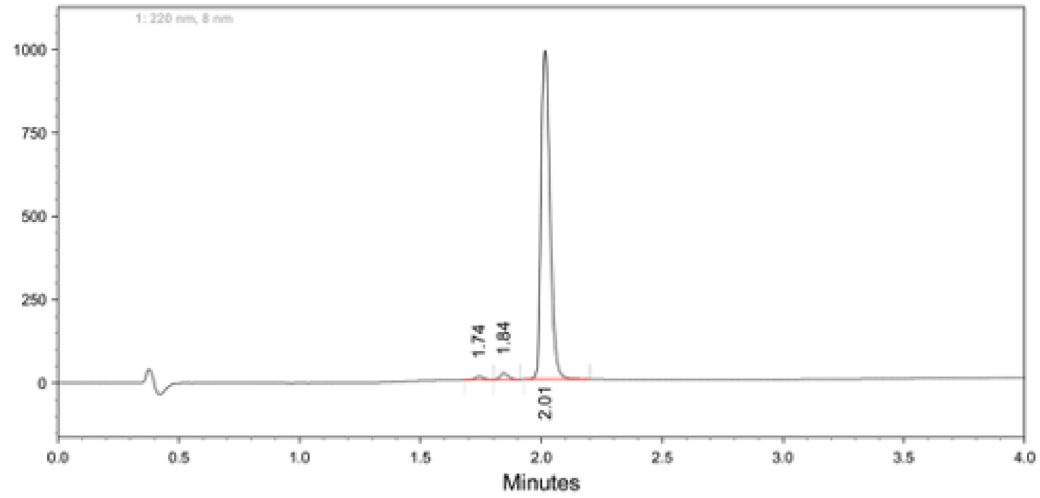

1: 220 nm, 8 nm

Retention Time	***Height***	***Area***	***Area Percent***
1.74	10390	26019	1.00
1.84	19534	46090	1.77
2.01	970886	2533299	97.23

Instrument : LCMS AR
A:,Xtimate,2.1*30mm,3um
B:XBridge Shield, 2.1*50mm,5um
Confidential. For research only NOT for regulatory filing

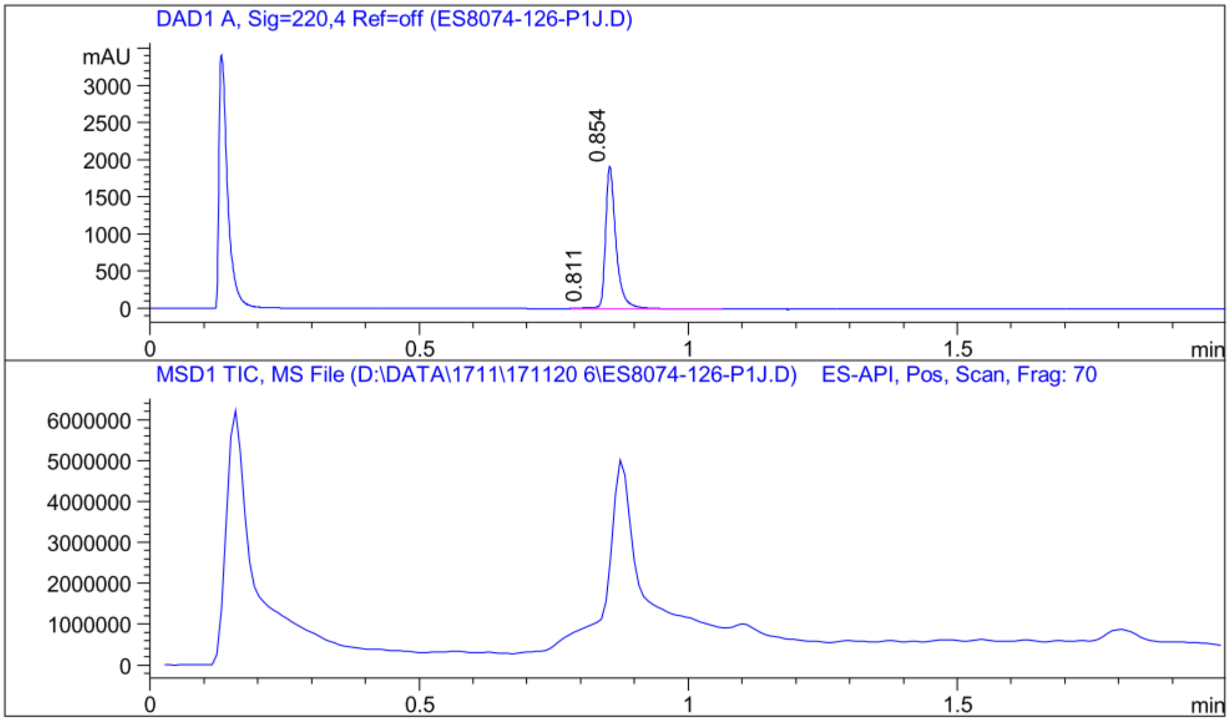

Report

===

Signal ->: DAD1 A, Sig=220,4 Ref=off

#	Meas. Ret.	Height	Width	Area	Area %
1	0.811	17.572	0.022	29.356	1.115
2	0.854	1902.941	0.021	2604.505	98.885

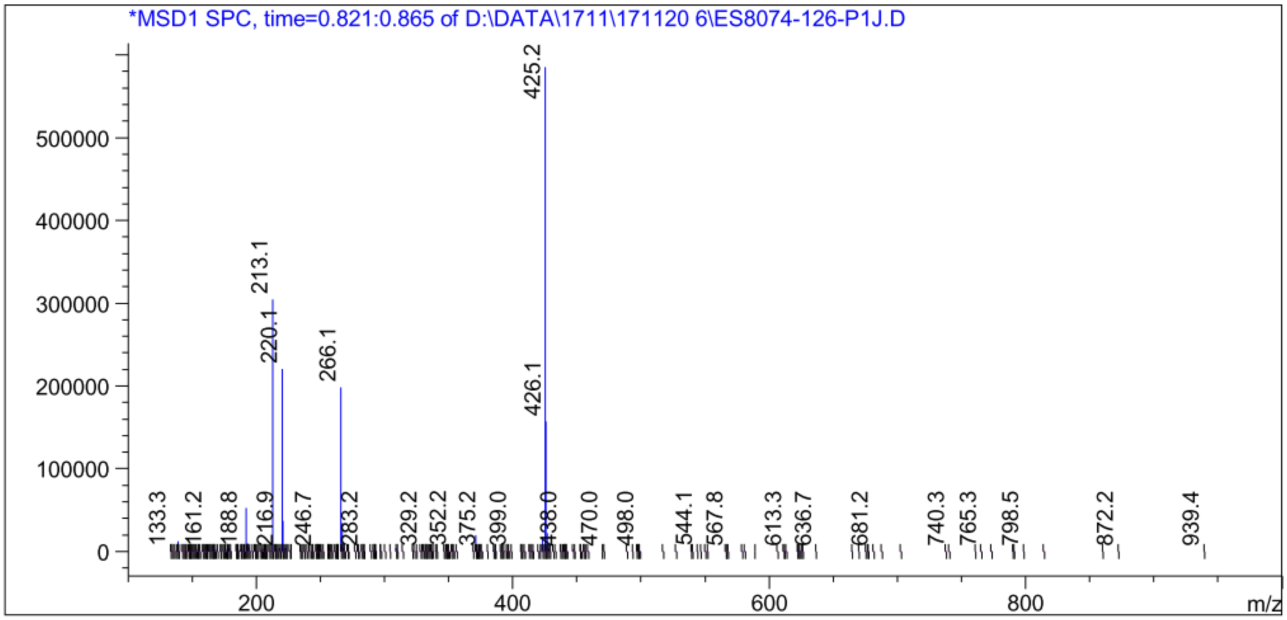
*MSD1 SPC, time=0.821:0.865 of D:\DATA\1711\171120 6\ES8074-126-P1J.D
425.2
213.1
220.1
266.1
426.1
m/z

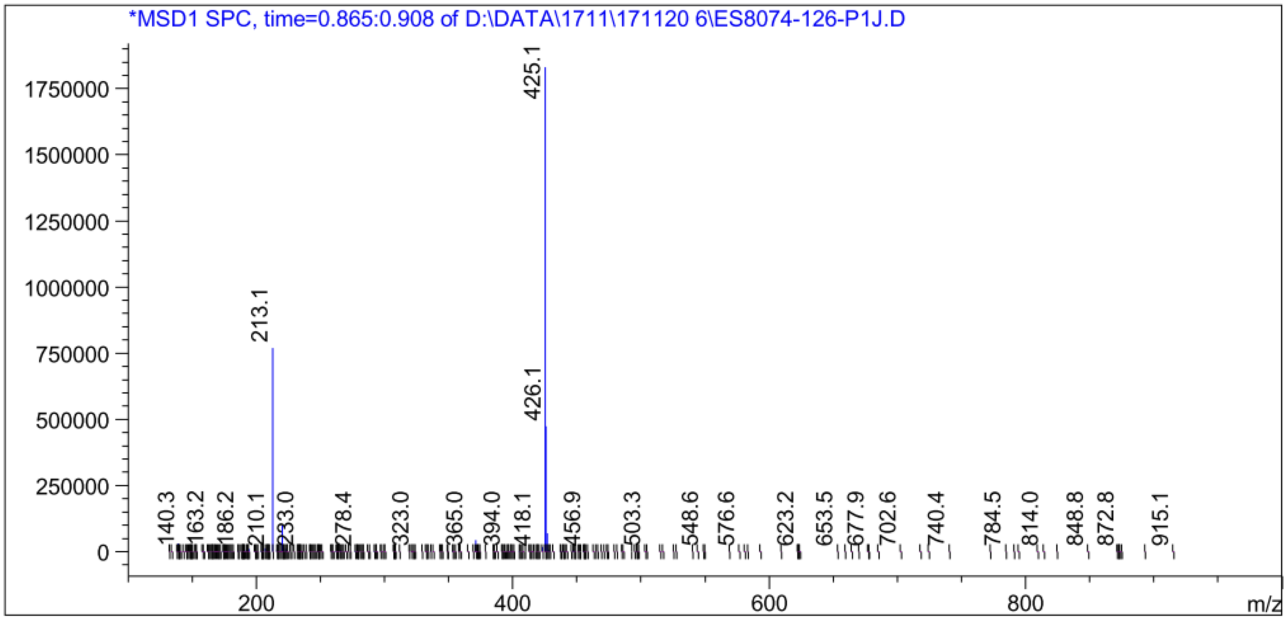
*MSD1 SPC, time=0.865:0.908 of D:\DATA\1711\171120 6\ES8074-126-P1J.D
425.1
213.1
426.1
m/z

Method :Column: Chiralpak AS-H 150*4.6mm I.D., 5um
Mobile phase: A:CO2 B:ethanol (0.05% DEA)
Gradient: hold 5% for 0.5 min,then from 5% to 40% of B in 3.5 min and hold 40% for 2.5 min, then 5% of B for 1.5 min
Flow rate: 3mL/min Column temp:40 C

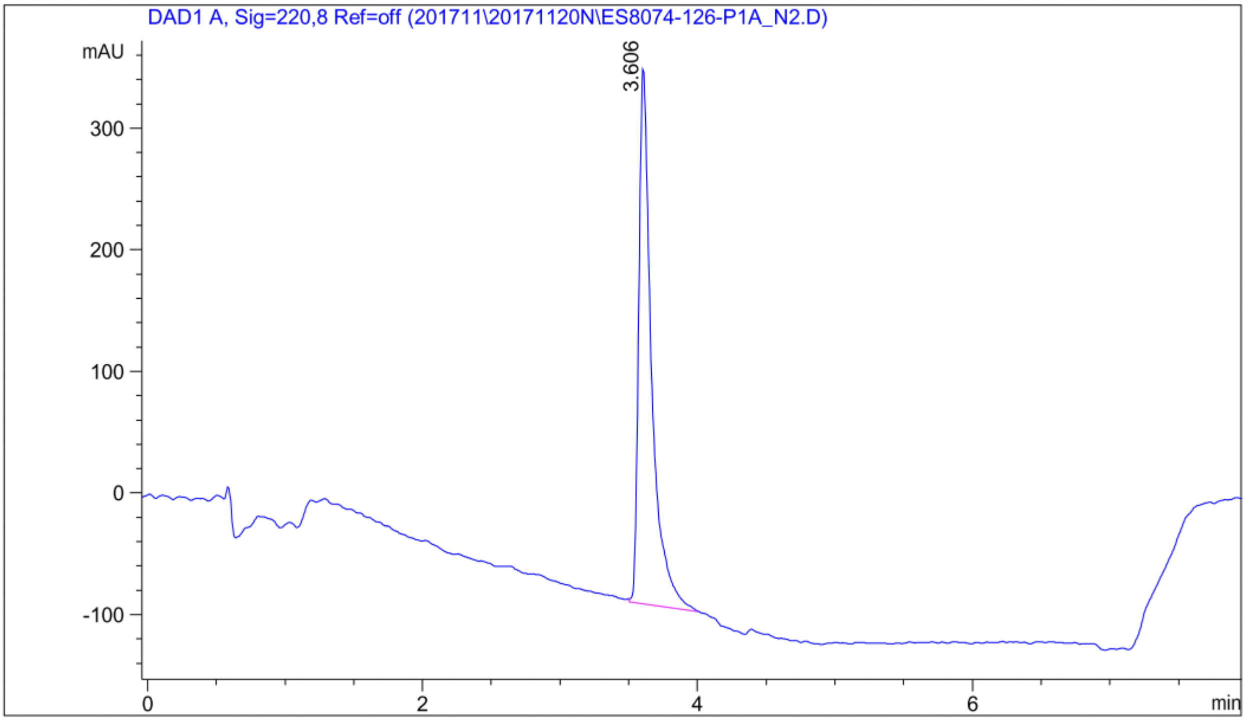

Signal 1 : DAD1 A, Sig=220,8 Ref=off

Peak	Meas. Ret. Time	Height	Height %	Width	Area	Area %
1	3.606	441.159	100.000	0.108	2861.488	100.000

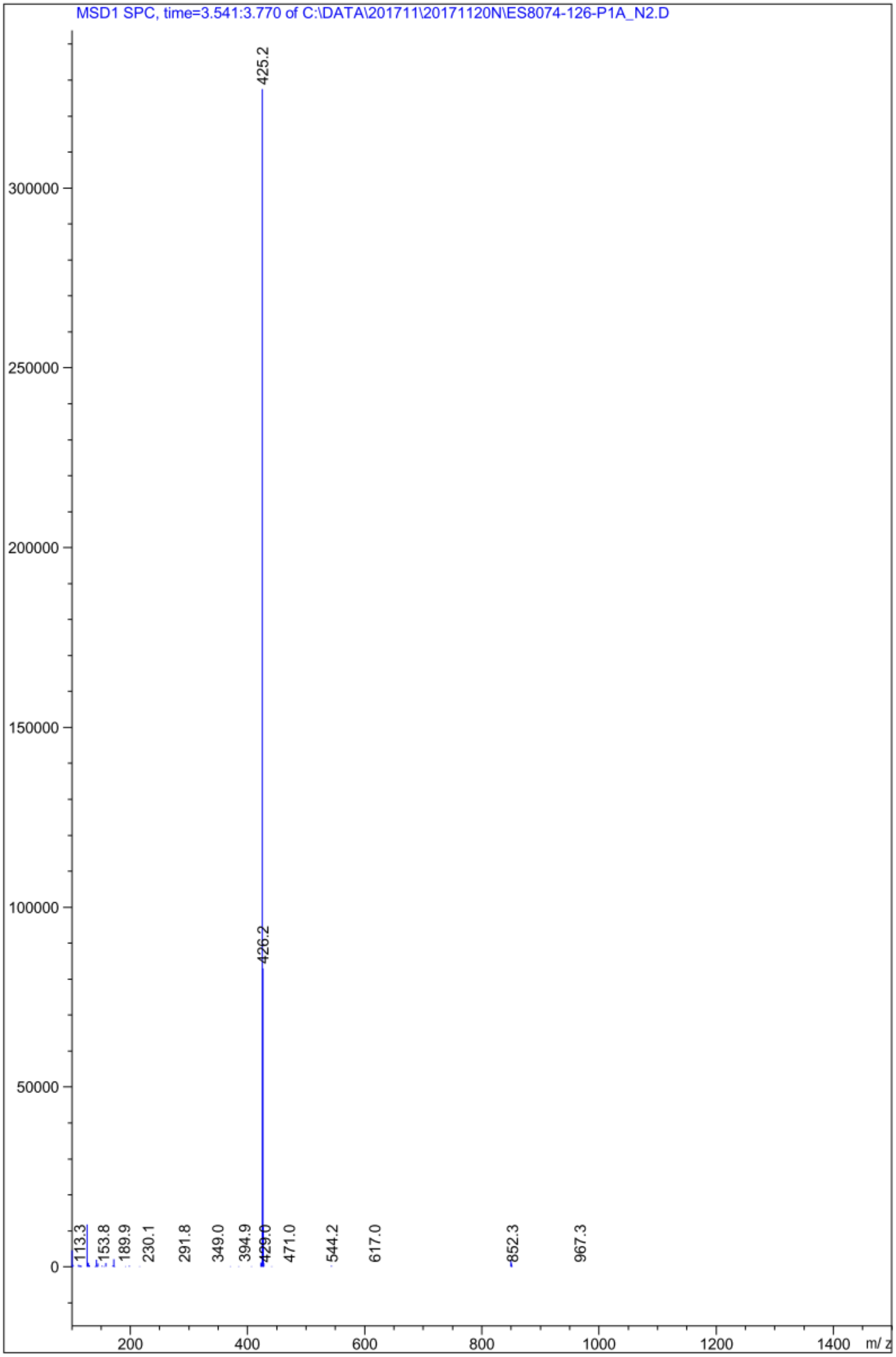

MSD1 SPC, time=3.541:3.770 of C:\DATA\201711\20171120N\ES8074-126-P1A_N2.D
425.2
426.2
113.3
153.8
189.9
230.1
291.8
349.0
394.9
429.0
471.0
544.2
617.0
852.3
967.3
300000
250000
200000
150000
100000
50000
0
200
400
600
800
1000
1200
1400
m/z

Compound 8

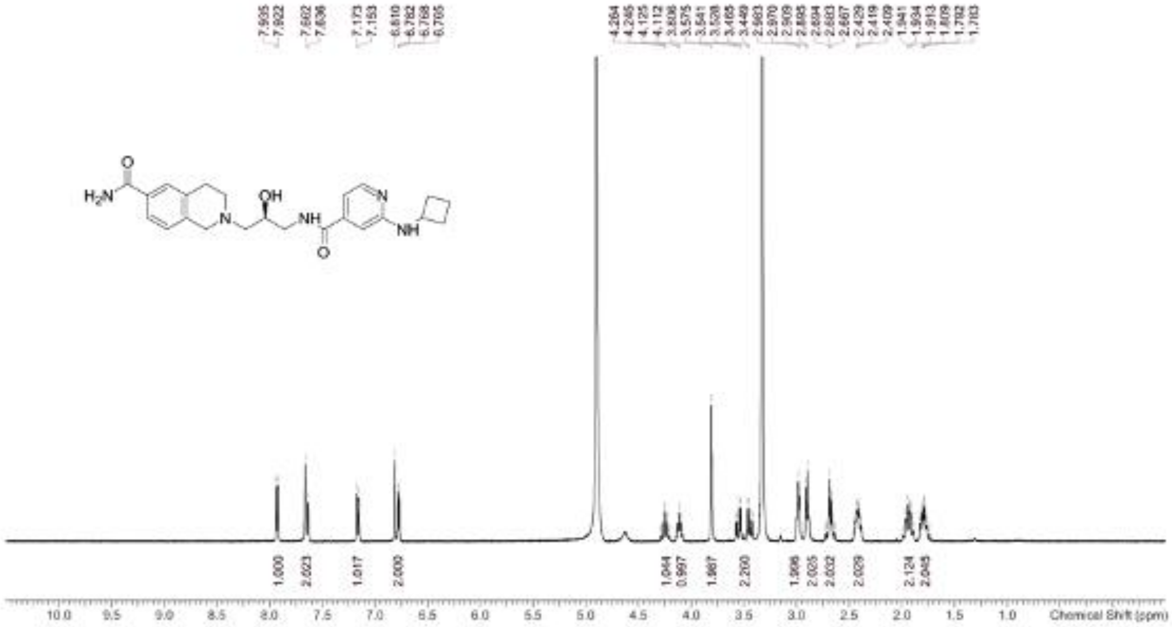

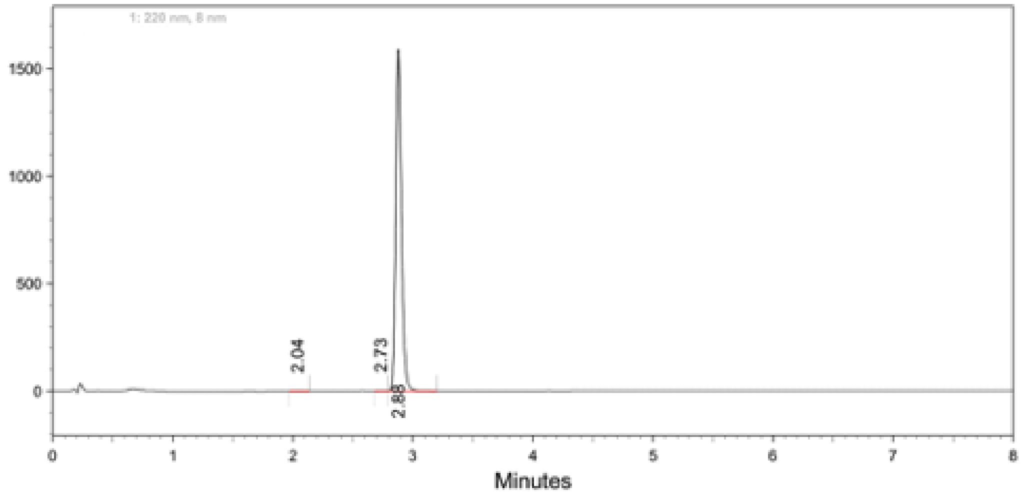

1: 220 nm, 8 nm

Retention Time	***Height***	***Area***	***Area Percent***
2.04	1709	7005	0.13
2.73	2790	9021	0.17
2.88	1578250	5180554	99.69

```
Instrument          : LCMS AR
                      A:,Xtimate,2.1*30mm,3um
                      B:XBridge Shield,  2.1*50mm,5um
                      Confidential. For research only  NOT for regulatory fili
```

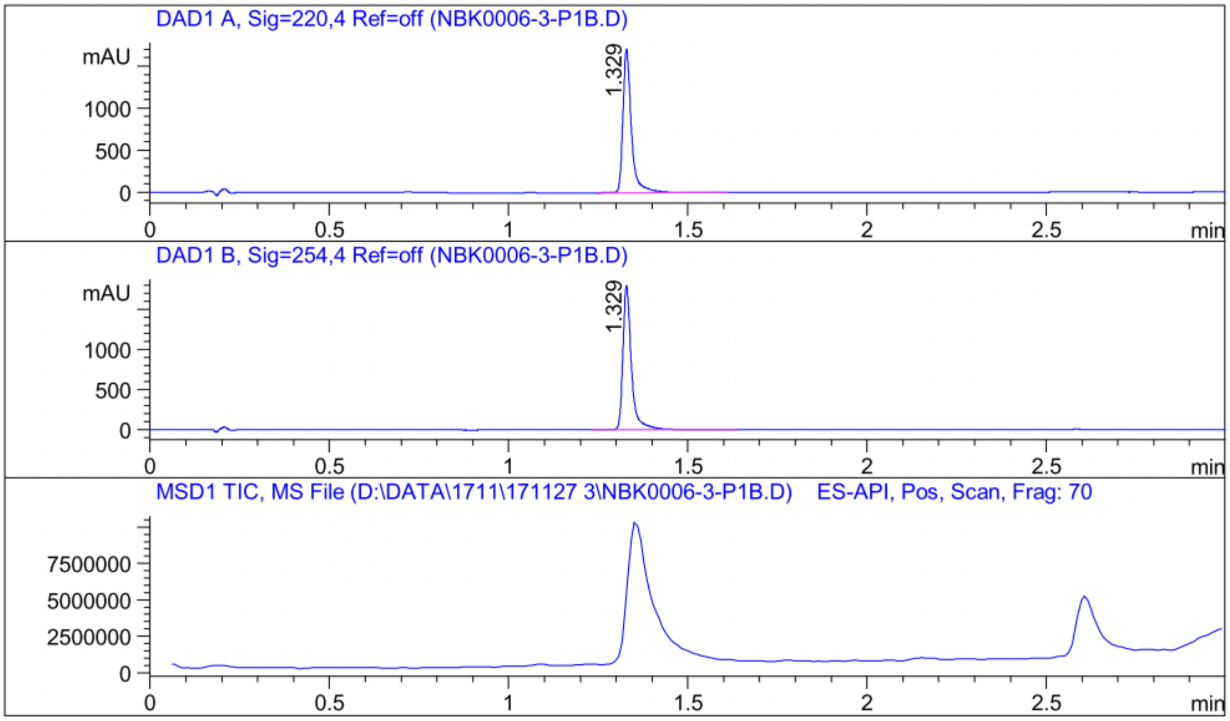

Report

```
======================================================================
   Signal ->: DAD1 A, Sig=220,4 Ref=off
    #  Meas. Ret.   Height    Width        Area        Area %
   ----------------------------------------------------------
     1       1.329   1692.987   0.025        2828.224 100.000
   ----------------------------------------------------------


   Signal ->: DAD1 B, Sig=254,4 Ref=off
    #  Meas. Ret.   Height    Width        Area        Area %
   ----------------------------------------------------------
     1       1.329   1785.727   0.026        2999.818 100.000
   ----------------------------------------------------------
```

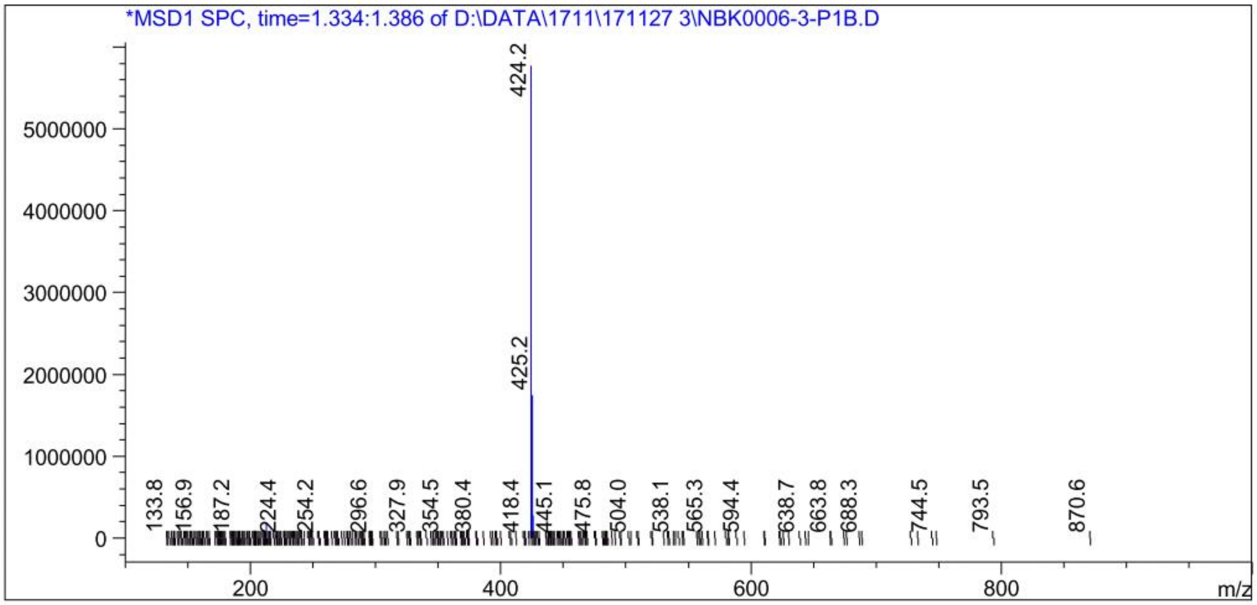
*MSD1 SPC, time=1.334:1.386 of D:\DATA\1711\171127 3\NBK0006-3-P1B.D
424.2
425.2
133.8
156.9
187.2
224.4
254.2
296.6
327.9
354.5
380.4
418.4
445.1
475.8
504.0
538.1
565.3
594.4
638.7
663.8
688.3
744.5
793.5
870.6
5000000
4000000
3000000
2000000
1000000
0
200
400
600
800
m/z

```
Acq Method          : D:\DATA\201712\20171201N\OD_ETOH(DEA)_5_40_2,8ML_8MIN.M
Data Filename       : D:\DATA\201712\20171201N\NBK0006-3-P1B_D3.D
Instrument          : SFC-D (12-102)
Method              : Column: Chiralcel OD-3 100×4.6mm I.D., 3um
                      Mobile phase: A:CO2  B:ethanol (0.05% DEA)
                      Gradient: from 5% to 40% of B in 4.5min and hold 40%
                      for 2.5min,  then 5% of B for 1min
                      Flow rate: 2.8mL/min    Column temperature:40 C
```

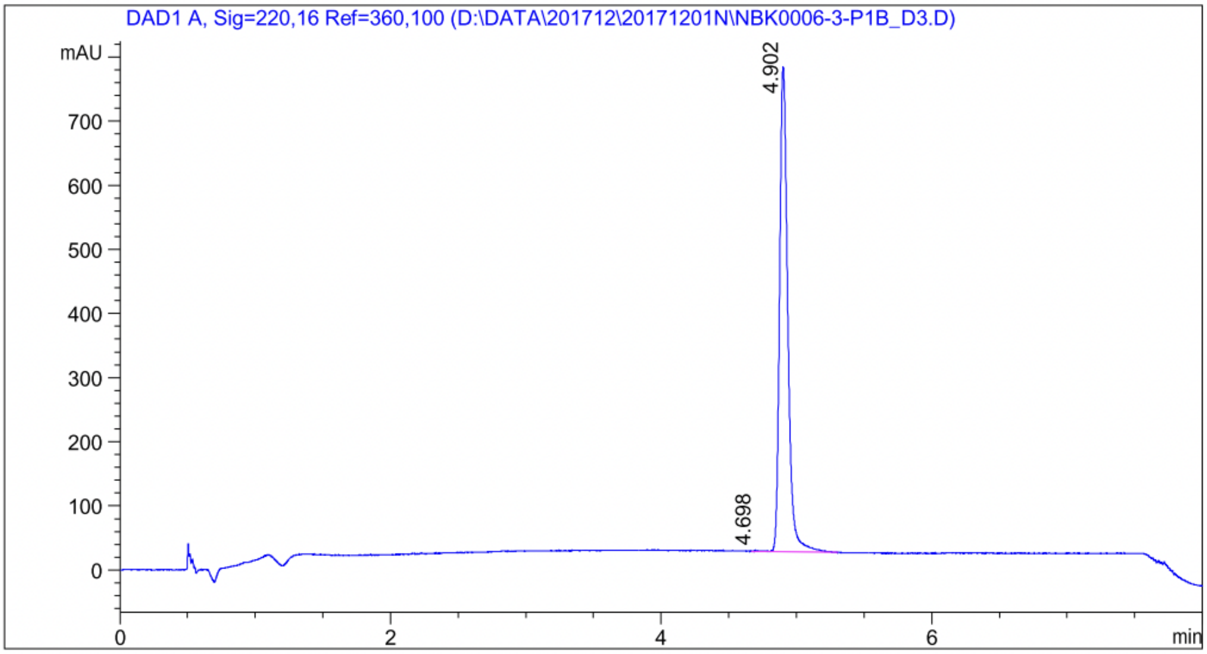

DAD1 A, Sig=220,16 Ref=360,100

#	Meas. Ret. Time	Height	Height %	Width	Area	Area %
1	4.698	1.445	0.191	0.076	6.581	0.206
2	4.902	757.001	99.809	0.070	3189.239	99.794

Compound 9

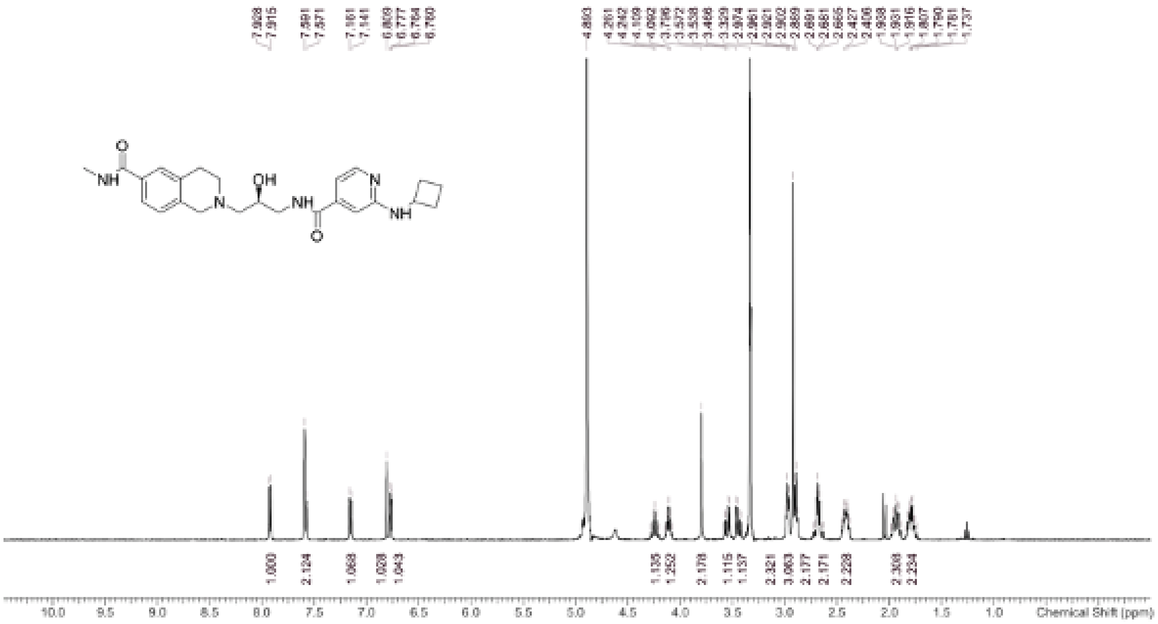

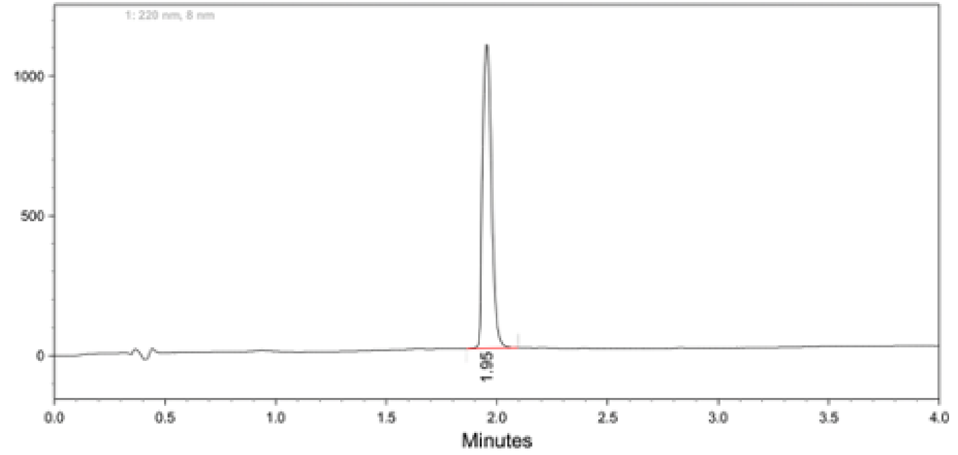

1: 220 nm, 8 nm

Retention Time	***Height***	***Area***	***Area Percent***
1.95	1073857	3070374	100.00

```
Instrument         : LCMS AR
                     A:,Xtimate,2.1*30mm,3um
                     B:XBridge Shield,  2.1*50mm,5um
                     Confidential. For research only  NOT for regulatory fili
```

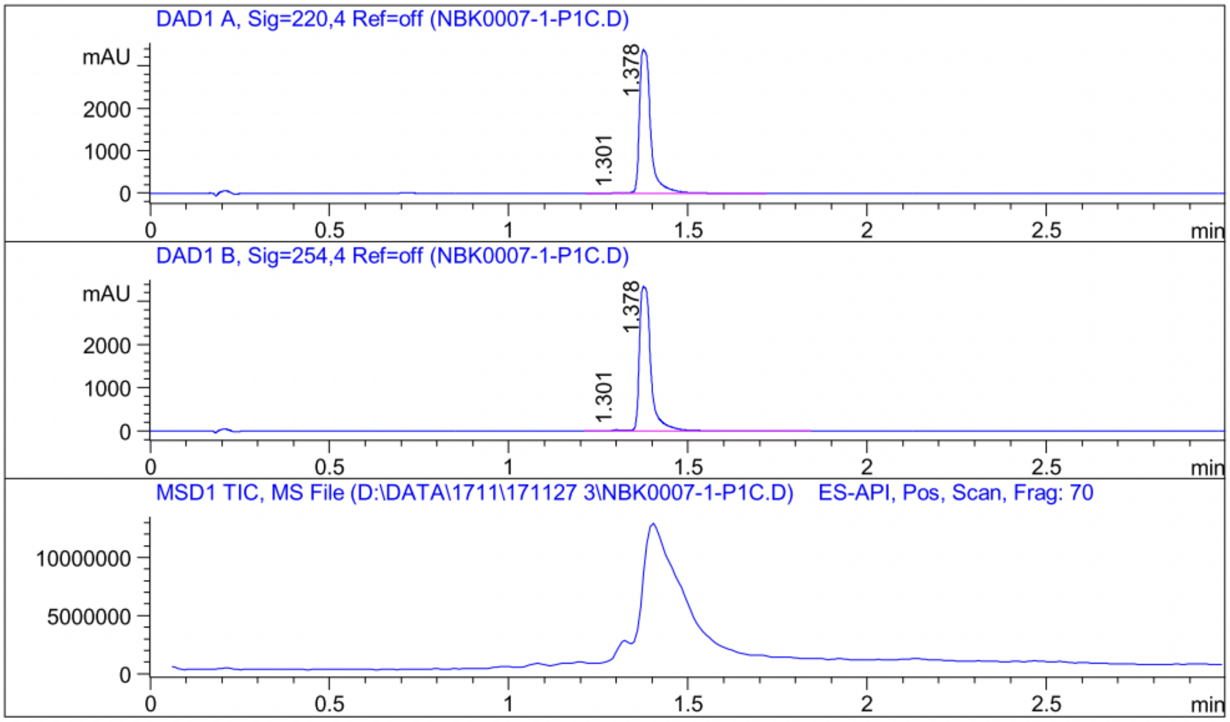

Report

Signal ->: DAD1 A, Sig=220,4 Ref=off

#	Meas. Ret.	Height	Width	Area	Area %
1	1.301	17.143	0.027	30.386	0.409
2	1.378	3358.684	0.034	7399.476	99.591

Signal ->: DAD1 B, Sig=254,4 Ref=off

#	Meas. Ret.	Height	Width	Area	Area %
1	1.301	29.294	0.026	50.798	0.673
2	1.378	3342.430	0.034	7494.796	99.327

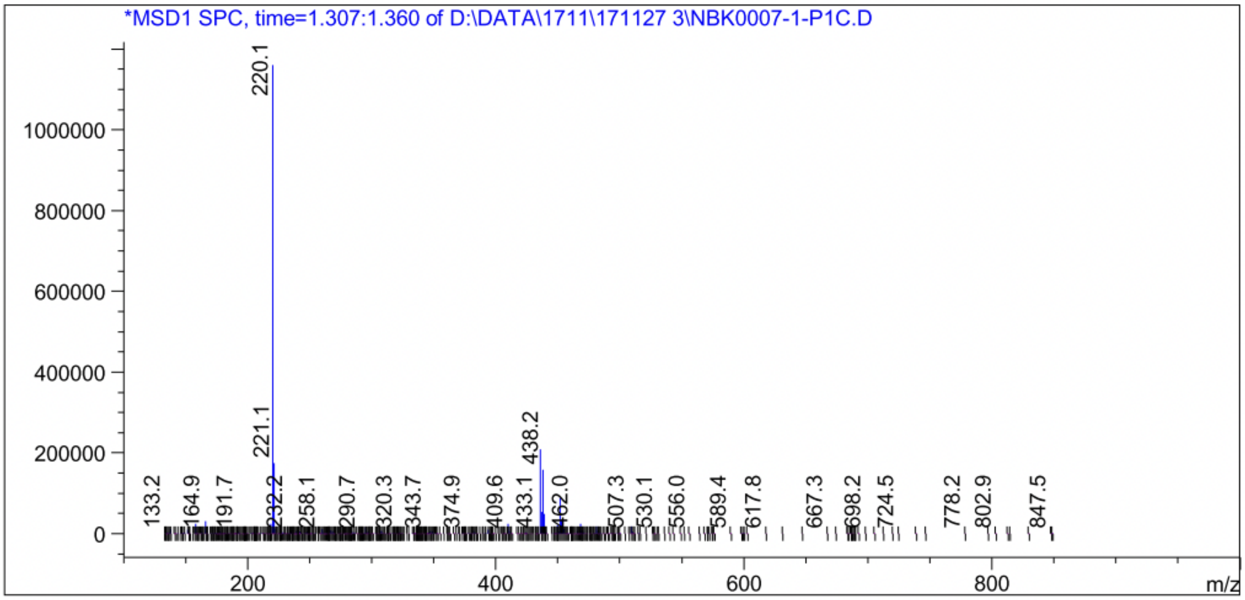
*MSD1 SPC, time=1.307:1.360 of D:\DATA\1711\171127 3\NBK0007-1-P1C.D
220.1
221.1
438.2
m/z

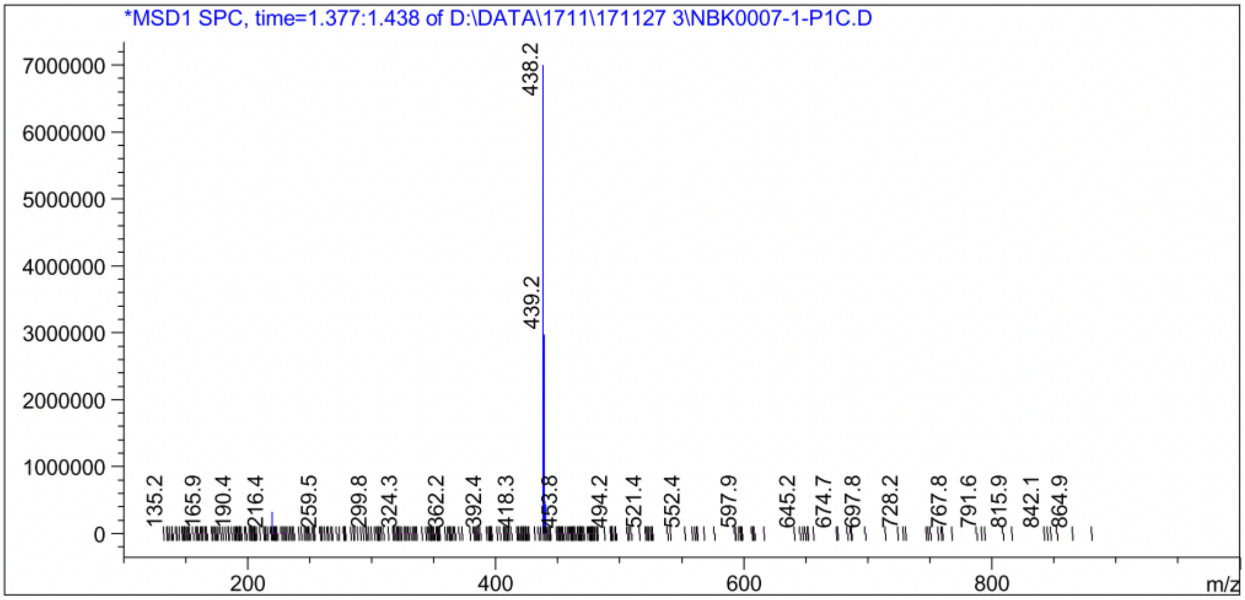
*MSD1 SPC, time=1.377:1.438 of D:\DATA\1711\171127 3\NBK0007-1-P1C.D
438.2
439.2
m/z

```
Method              : Column: Chiralcel OD-3 100×4.6mm I.D., 3um
                      Mobile phase: A:CO2  B:ethanol (0.05% DEA)
                      Gradient: from 5% to 40% of B in 4.5min and hold 40%
                      for 2.5min,  then 5% of B for 1min
                      Flow rate: 2.8mL/min    Column temperature:40 C
```

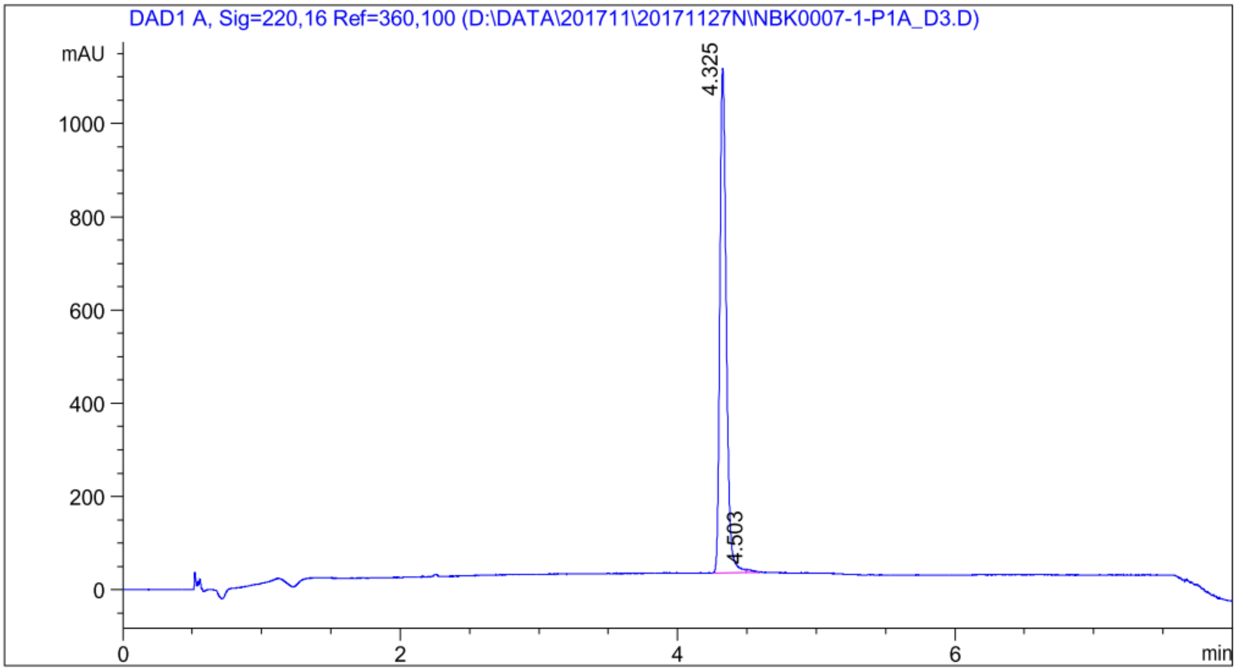

DAD1 A, Sig=220,16 Ref=360,100

#	Meas. Ret. Time	Height	Height %	Width	Area	Area %
1	4.325	1083.911	99.412	0.054	3488.175	99.428
2	4.503	6.415	0.588	0.052	20.069	0.572

Compound 10

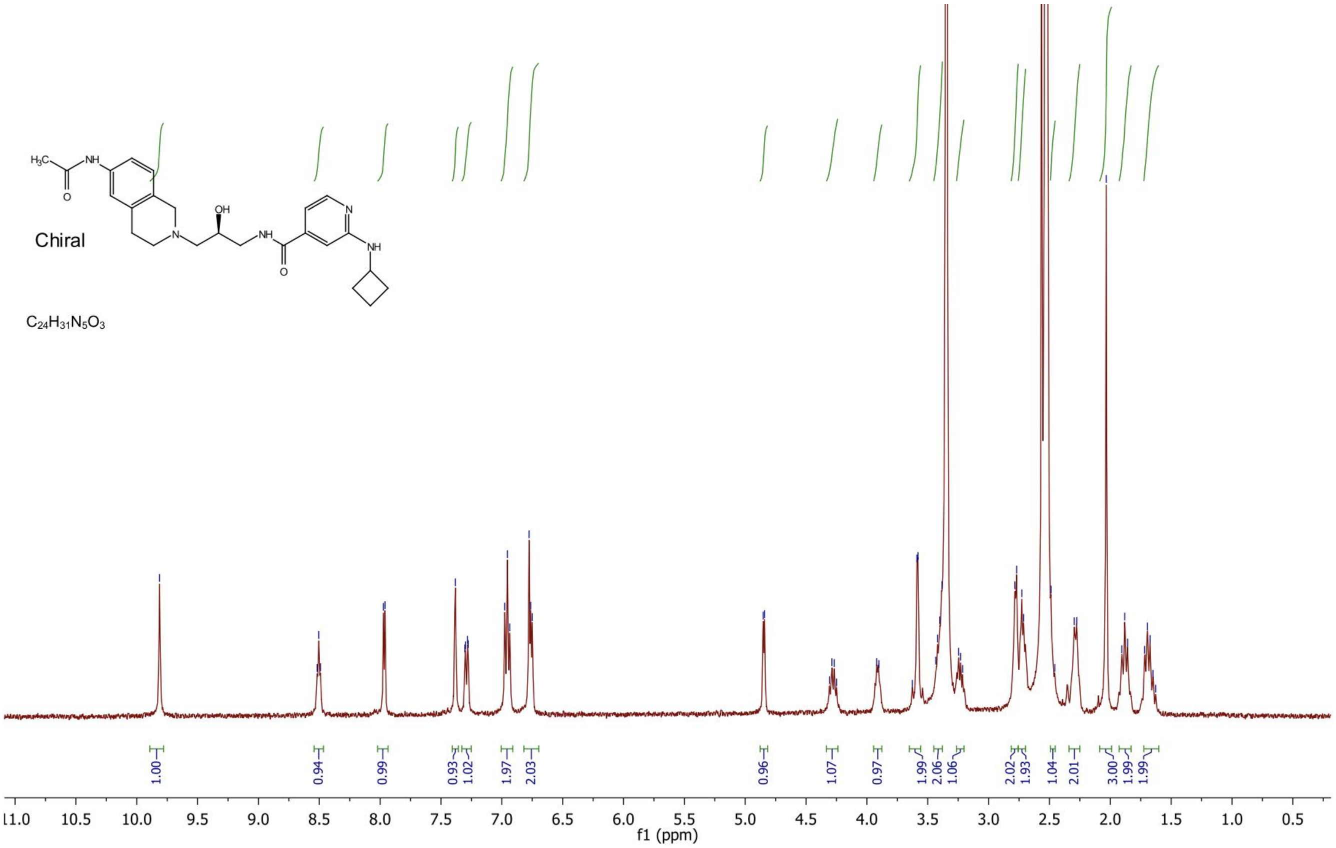

H3C
NH
O
OH
N
NH
O
N
NH
Chiral
C24H31N5O3
1.00
0.94
0.99
0.93
1.02
1.97
2.03
0.96
1.07
0.97
1.99
2.06
1.06
2.02
1.93
1.04
2.01
3.00
1.99
1.99
11.0
10.5
10.0
9.5
9.0
8.5
8.0
7.5
7.0
6.5
6.0
5.5
5.0
4.5
4.0
3.5
3.0
2.5
2.0
1.5
1.0
0.5
f1 (ppm)

MaxPeak: 96.84%
Ret_Time: 0.673 min

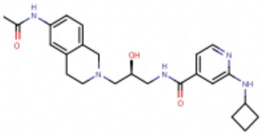

Mol Wt 437.54
Exact Mass 437.28

#	Time	Area%
1	0.673	96.84
2	0.696	3.16

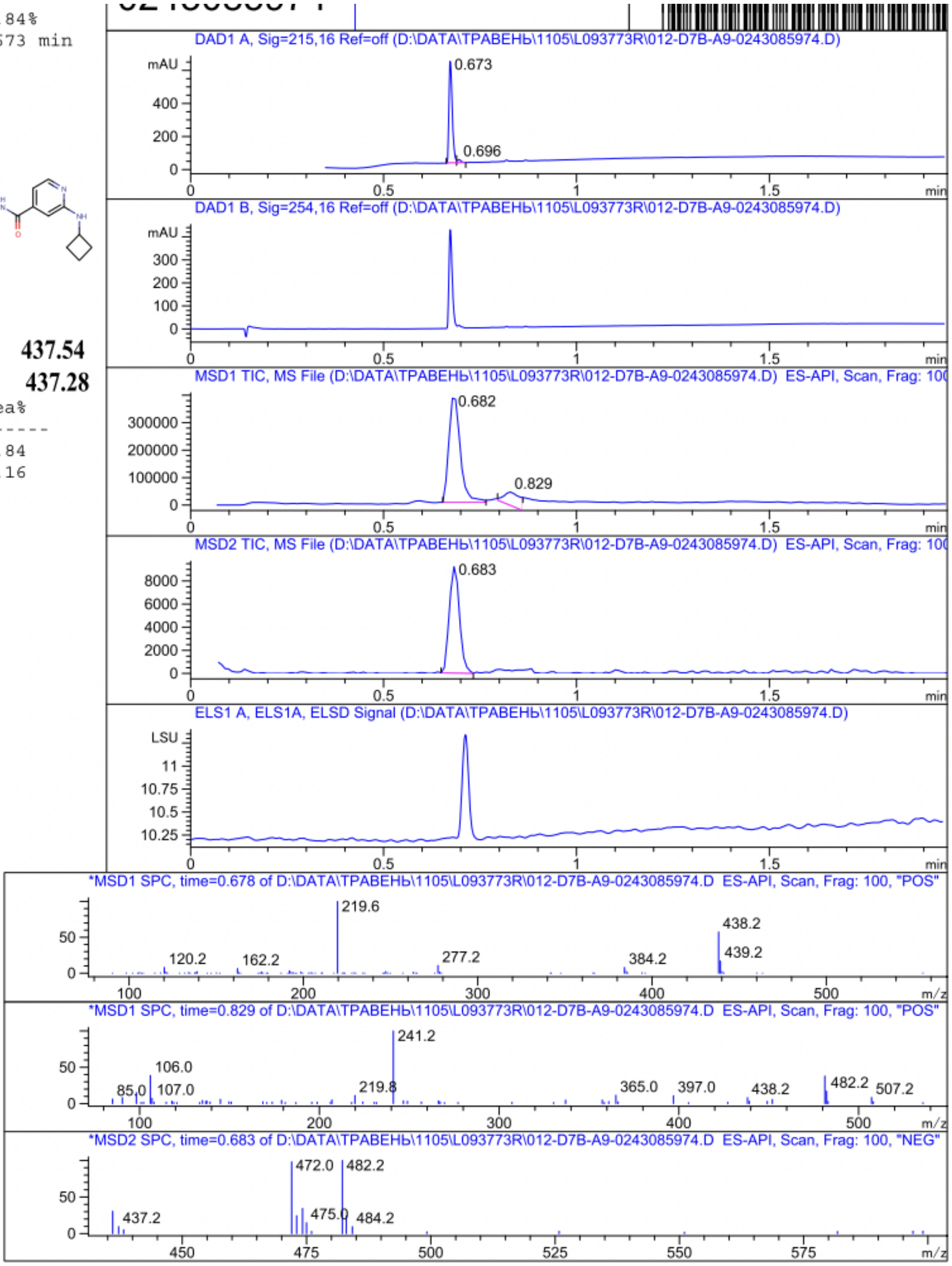

RT 0.682

RT 0.829

RT 0.683

Compound 11

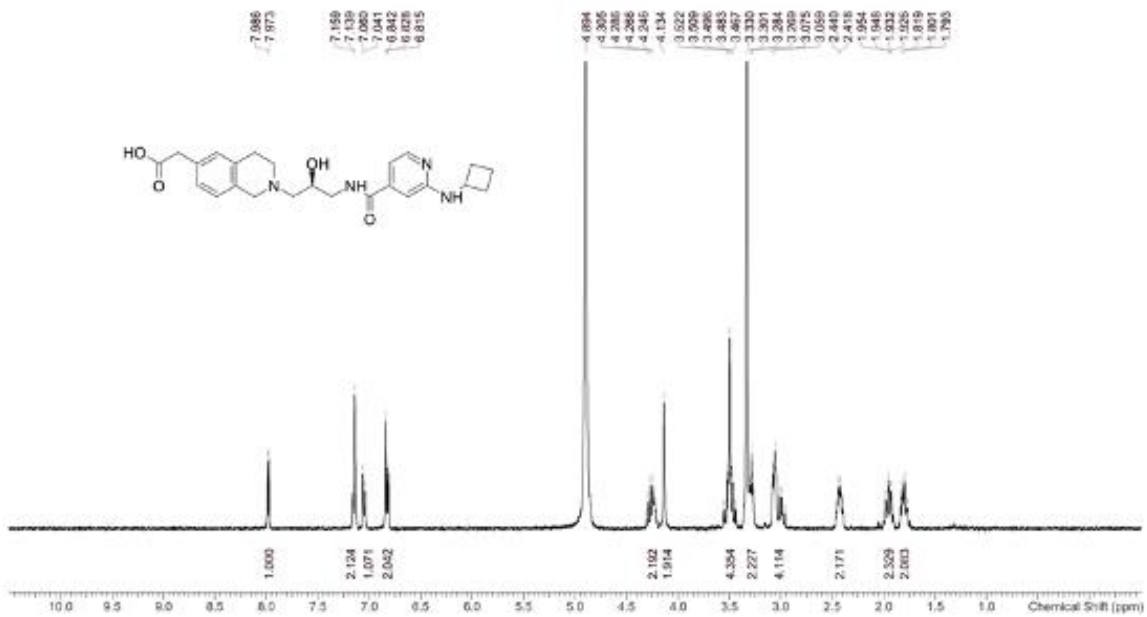

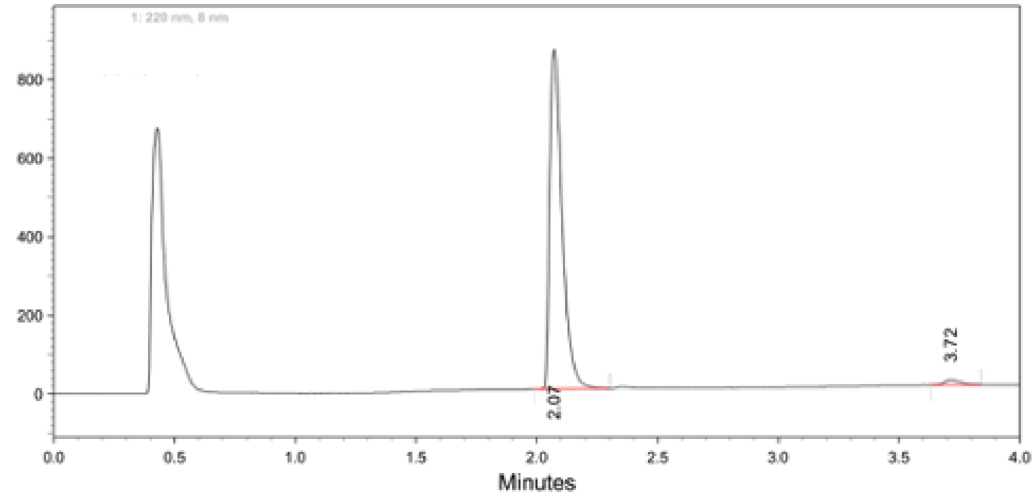

1: 220 nm, 8 nm

Retention Time	*Height*	*Area*	*Area Percent*
2.07	853196	3172574	98.22
3.72	12302	57511	1.78

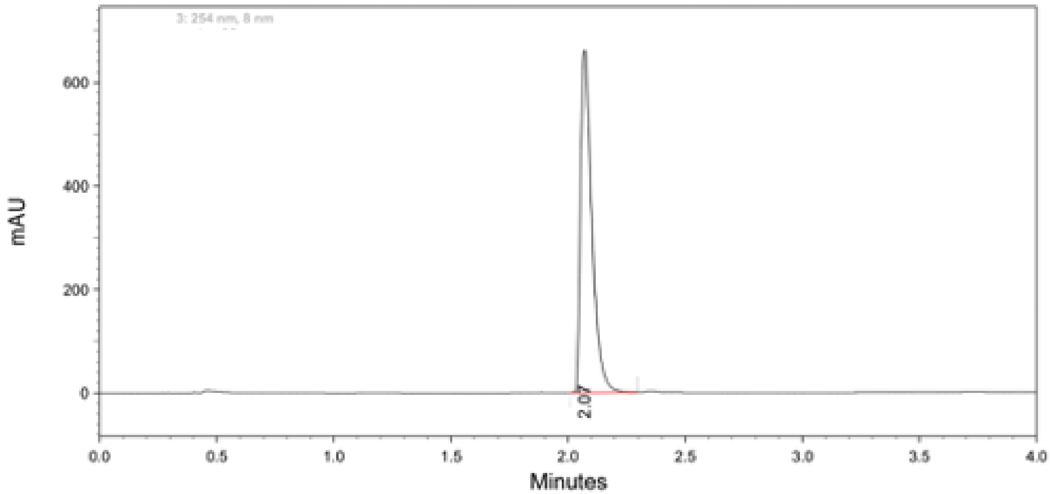

3: 254 nm, 8 nm

Retention Time	Height	Area	Area Percent
2.07	656844	2238328	100.00

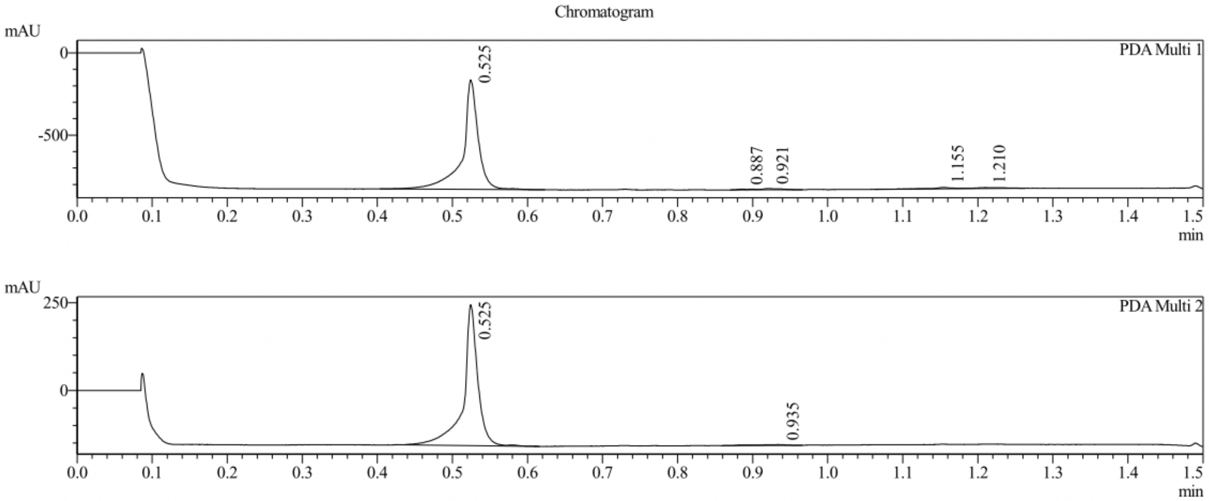

1 PDA Multi 1 / 220nm 4nm
2 PDA Multi 2 / 254nm 4nm

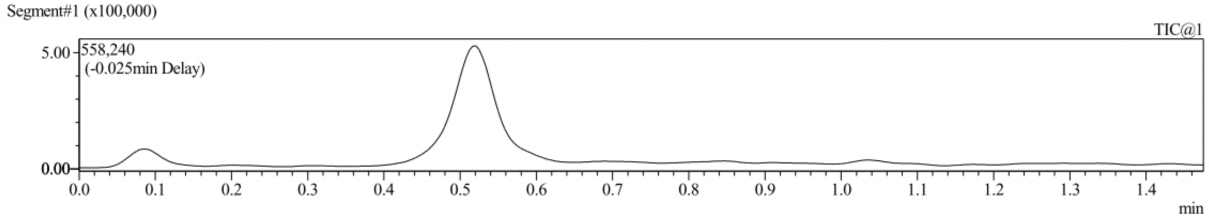

Integration Result

SPD-M20A Ch1 220nm 4nm

Peak#	Ret. Time	Height	Height %	USP Width	Area	Area %
1	0.525	655202	96.305	0.028	971457	95.042
2	0.887	4074	0.599	0.064	7205	0.705
3	0.921	7677	1.128	0.045	12665	1.239
4	1.155	7862	1.156	0.031	16838	1.647
5	1.210	5525	0.812	0.066	13968	1.367

SPD-M20A Ch2 254nm 4nm

Peak#	Ret. Time	Height	Height %	USP Width	Area	Area %
1	0.525	393783	99.433	0.026	539729	98.628
2	0.935	2247	0.567	0.045	7510	1.372

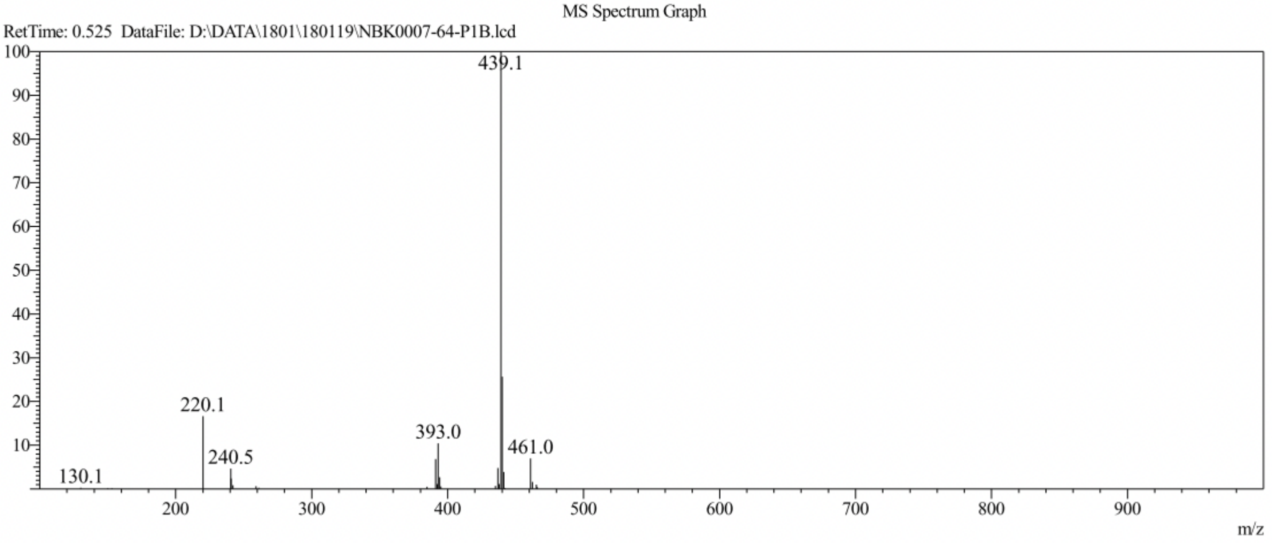

Method :Column: Chiralpak AD-3 50*4.6mm I.D., 3um
Mobile phase: A:CO2 B:ethanol (0.05% DEA)
Gradient: hold 5% for 0.2 min,then from 5% to 40% of B in 1.4 min and hold 40% for 1.05 min, then 5% of B for 0.35 min
Flow rate: 4mL/min Column temp:40 C

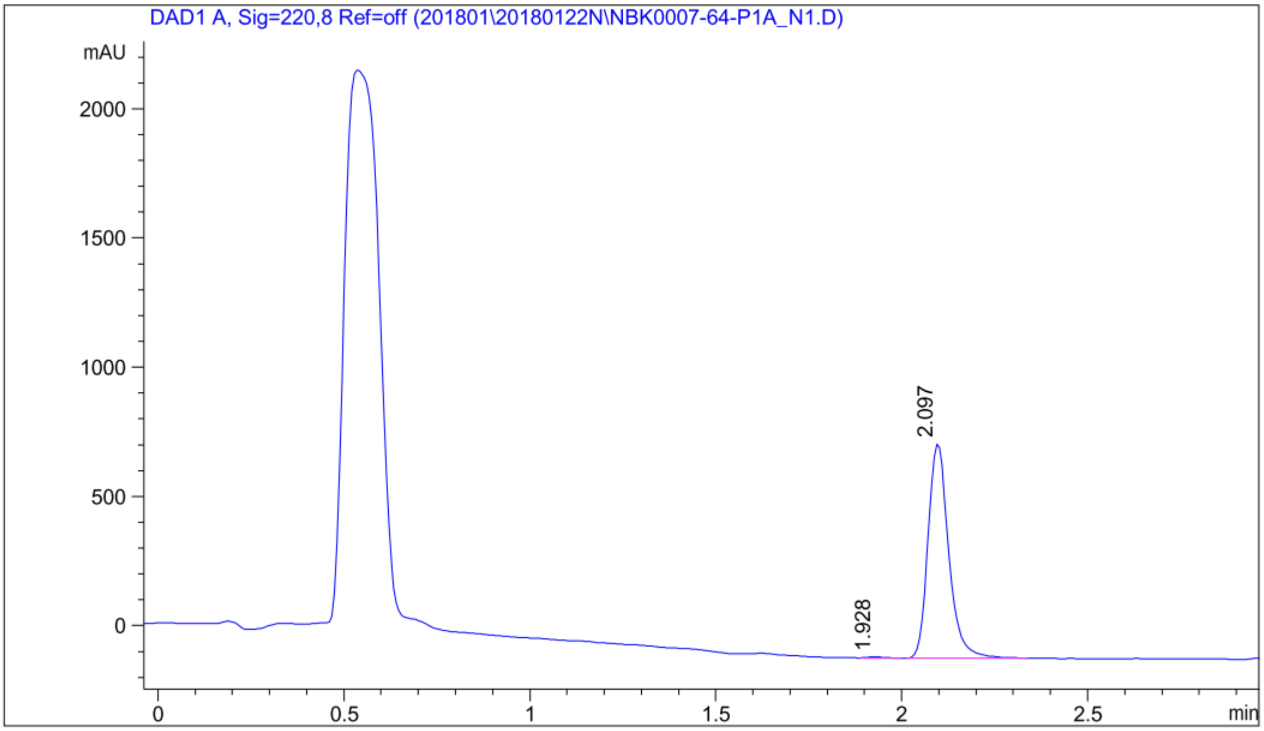

Signal 1 : DAD1 A, Sig=220,8 Ref=off

Peak	Meas. Ret. Time	Height	Height %	Width	Area	Area %
1	1.928	4.782	0.571	0.058	16.586	0.515
2	2.097	833.374	99.429	0.064	3202.912	99.485

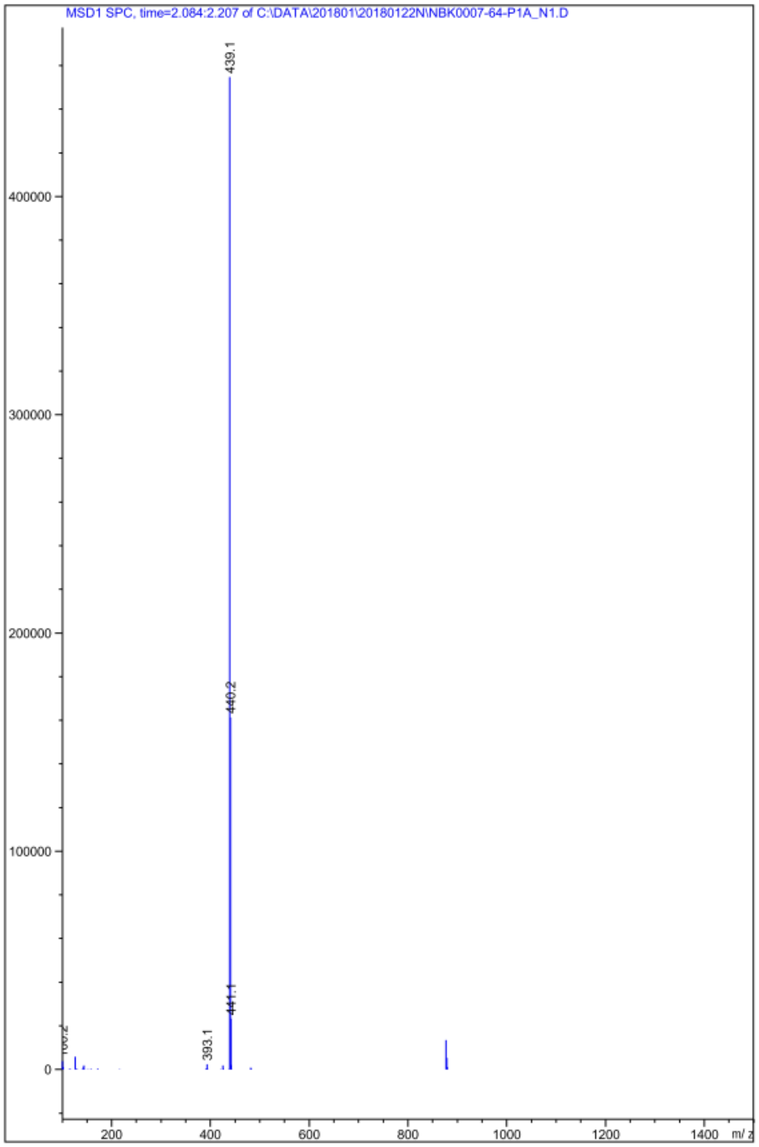

MSD1 SPC, time=2.084:2.207 of C:\DATA\201801\20180122N\NBK0007-64-P1A_N1.D
439.1
440.2
441.1
393.1
400000
300000
200000
100000
0
200
400
600
800
1000
1200
1400
m/z

Compound 12

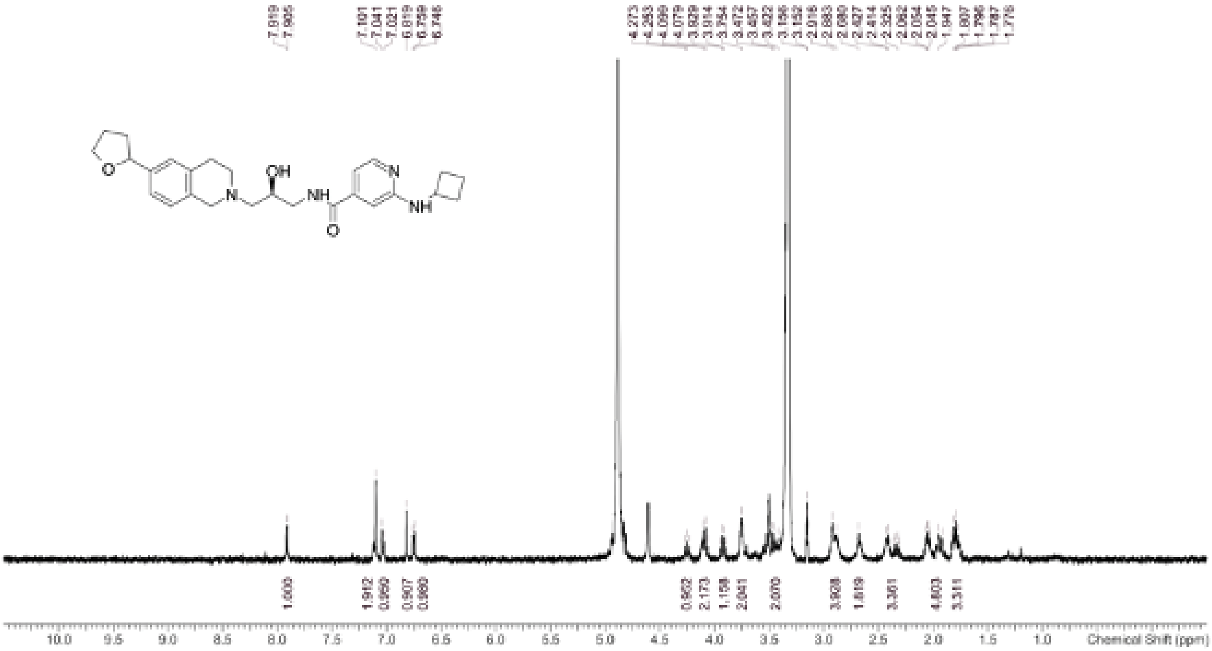

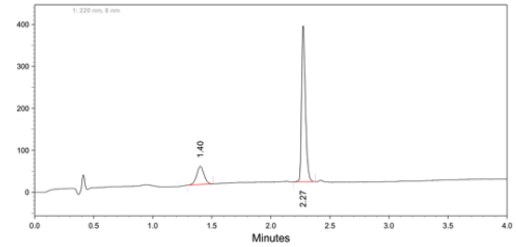

1: 220 nm, 8 nm

Retention Time	Height	Area	Area Percent
1.40	42824	185335	17.97
2.27	357098	845996	82.03

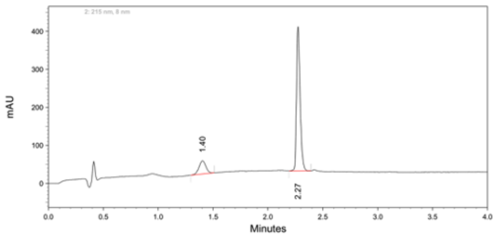

2: 215 nm, 8 nm

Retention Time	Height	Area	Area Percent
1.40	34457	151334	14.70
2.27	366281	878326	85.30

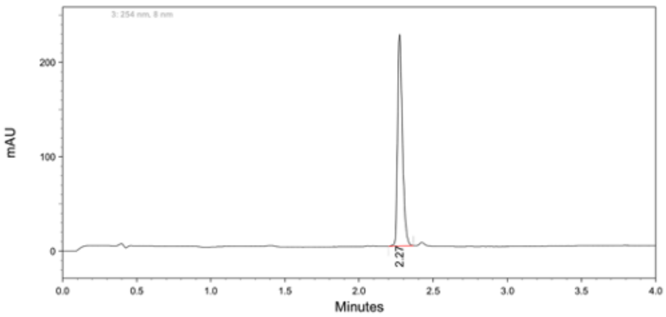

3: 254 nm, 8 nm

Retention Time	Height	Area	Area Percent
2.27	214288	502863	100.00

Instrument & column:LCMS-BG 28-106
Agilent Pursit 5 C18 20*2.0mm

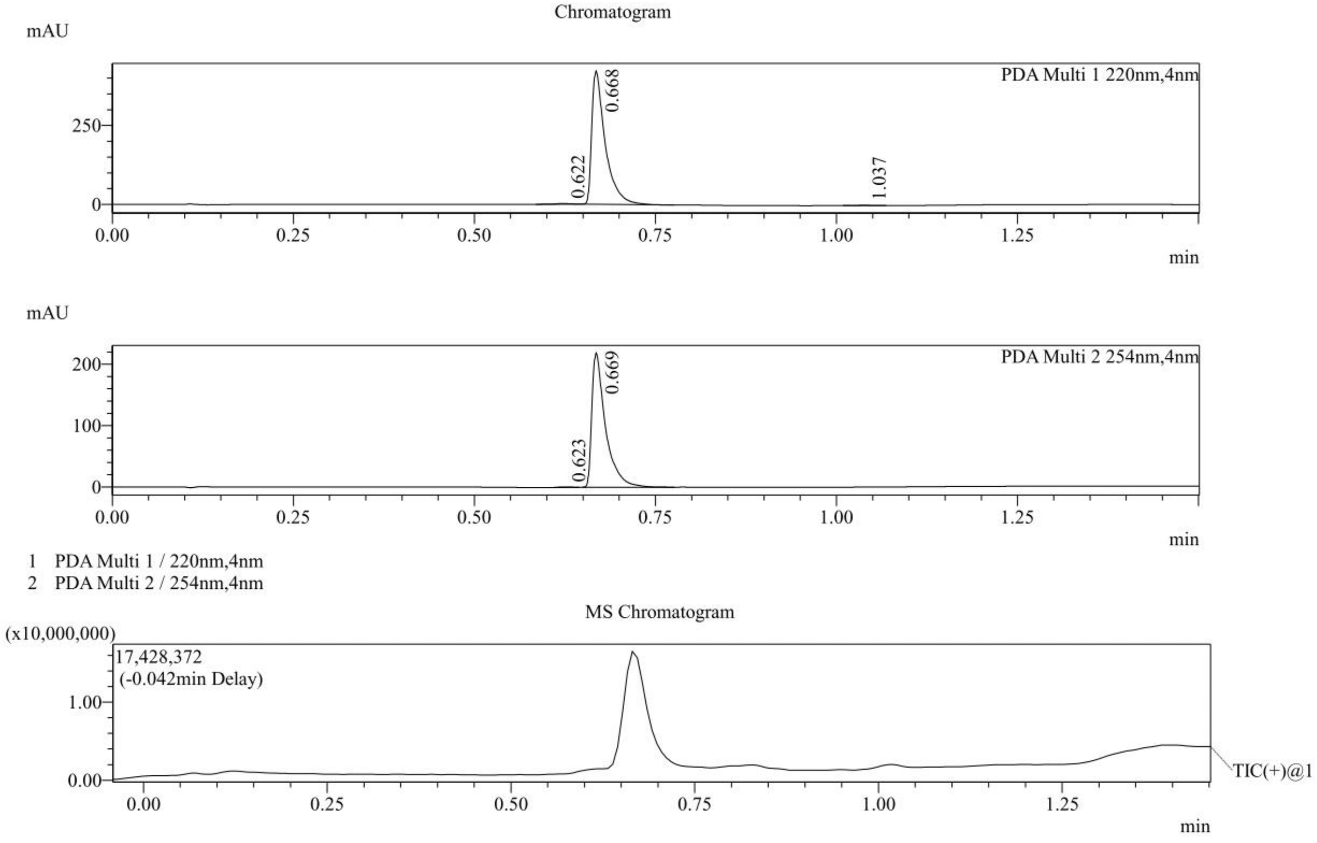

Integration Result

Peak Table

PDA Ch1 220nm

Peak#	Ret. Time	Height	Height%	USP Width	Area	Area%
1	0.622	2175	0.529	0.038	3129	0.567
2	0.668	407845	99.244	0.031	546981	99.186
3	1.037	931	0.227	0.037	1358	0.246

Peak Table

PDA Ch2 254nm

Peak#	Ret. Time	Height	Height%	USP Width	Area	Area%
1	0.623	818	0.384	0.031	936	0.324
2	0.669	212319	99.616	0.031	288099	99.676

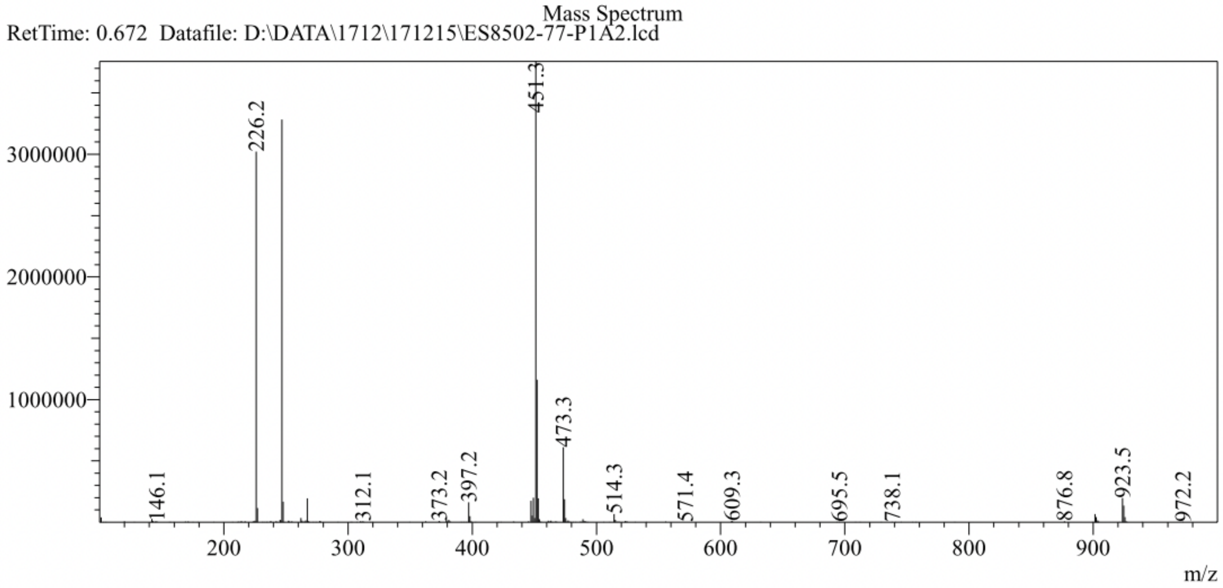
Mass Spectrum
RetTime: 0.672 Datafile: D:\DATA\1712\171215\ES8502-77-P1A2.lcd
3000000
2000000
1000000
226.2
451.3
473.3
146.1
312.1
373.2
397.2
514.3
571.4
609.3
695.5
738.1
876.8
923.5
972.2
200
300
400
500
600
700
800
900
m/z

Method :Column: Chiralcel OD-3 50*4.6mm I.D., 3um
Mobile phase: A: CO2 B:methanol (0.05% DEA)
Gradient: hold 5% for 0.2 min,then from 5% to 40% of B in 1.4 min and hold 40% for 1.05 min, then 5% of B for 0.35 min
Flow rate: 4mL/min Column temp:40 C

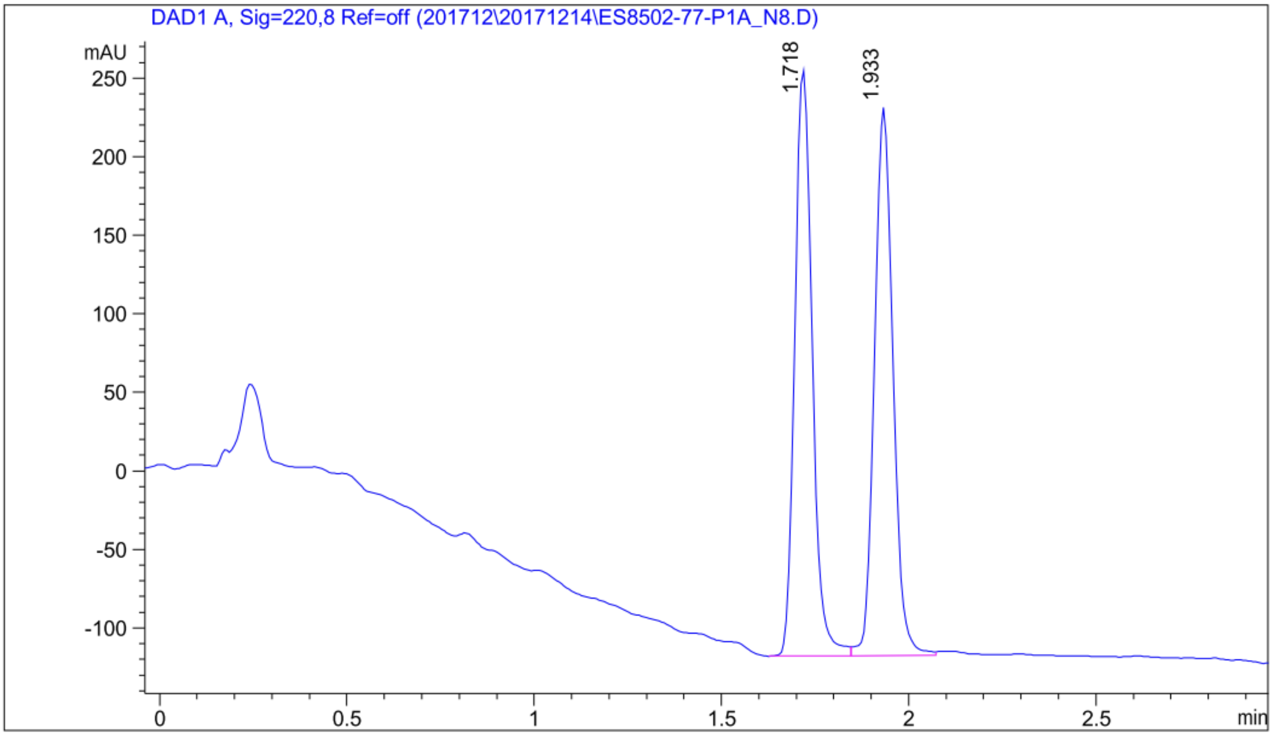

Signal 1 : DAD1 A, Sig=220,8 Ref=off

Peak	Meas. Ret. Time	Height	Height %	Width	Area	Area %
1	1.718	373.890	51.717	0.050	1211.644	50.248
2	1.933	349.058	48.283	0.054	1199.695	49.752

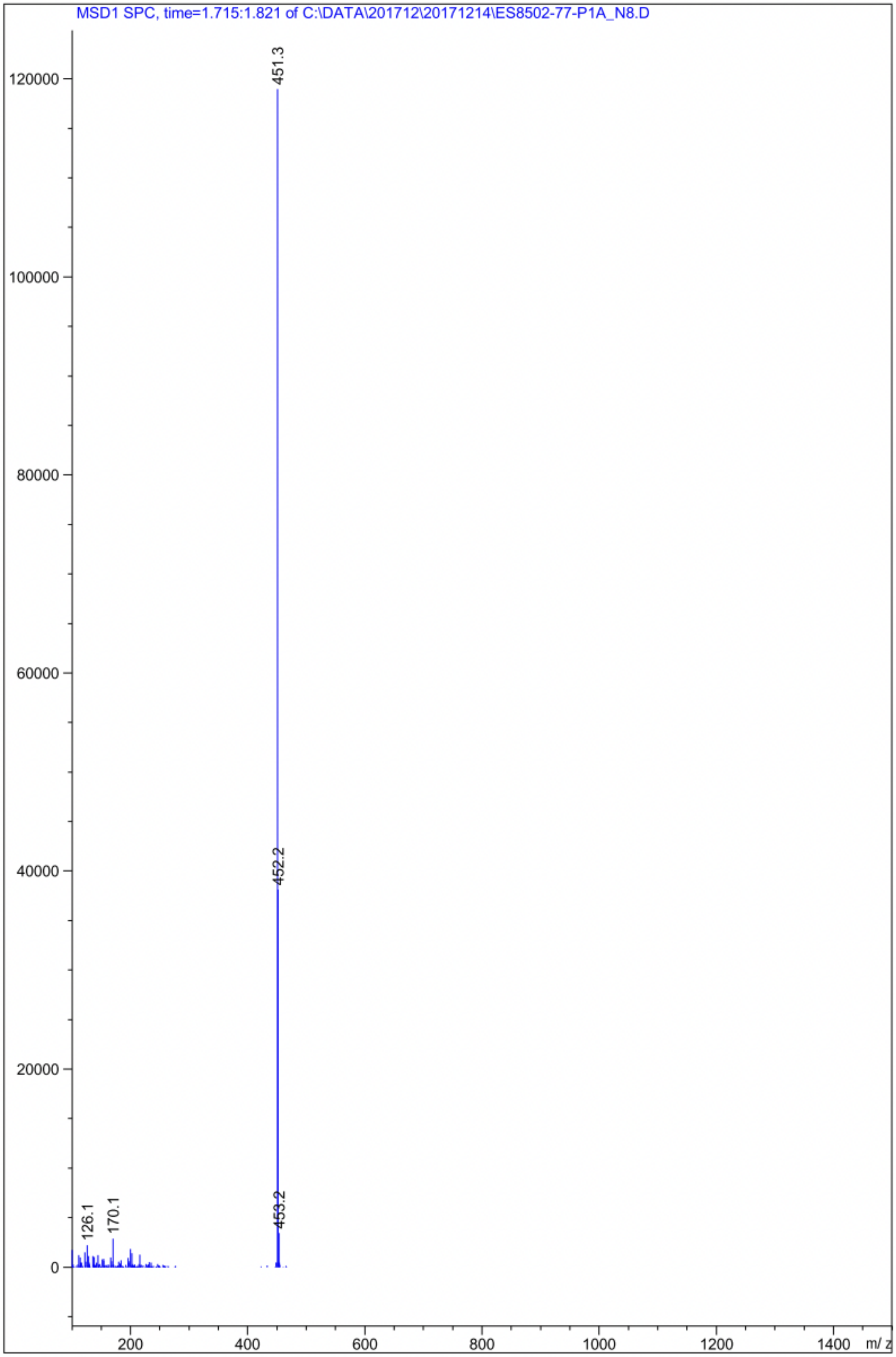

MSD1 SPC, time=1.715:1.821 of C:\DATA\201712\20171214\ES8502-77-P1A_N8.D
120000
100000
80000
60000
40000
20000
0
200
400
600
800
1000
1200
1400
m/z
451.3
452.2
453.2
126.1
170.1

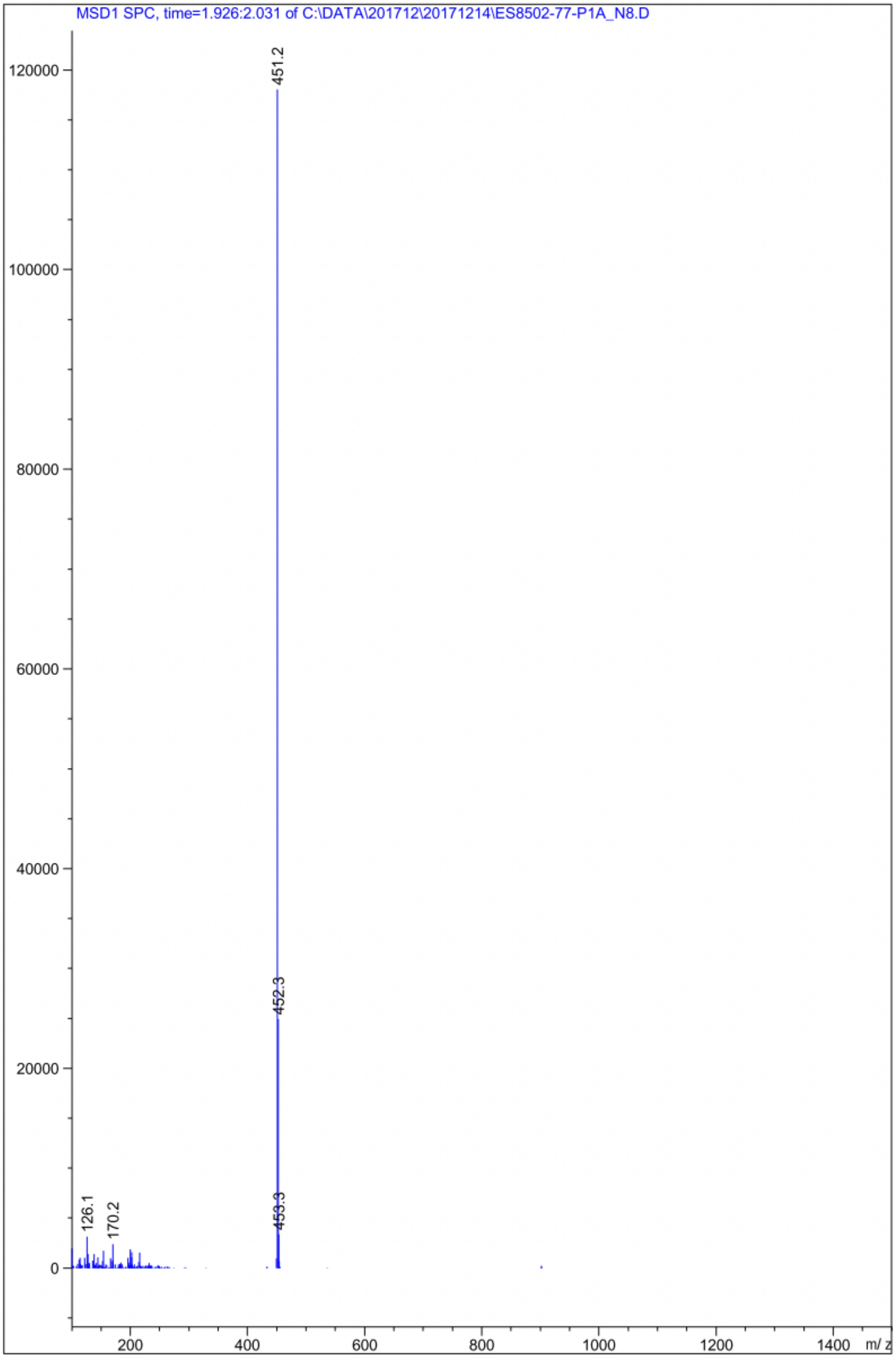
MSD1 SPC, time=1.926:2.031 of C:\DATA\201712\20171214\ES8502-77-P1A_N8.D
451.2
452.3
453.3
126.1
170.2
120000
100000
80000
60000
40000
20000
0
200
400
600
800
1000
1200
1400
m/z

Compound 13

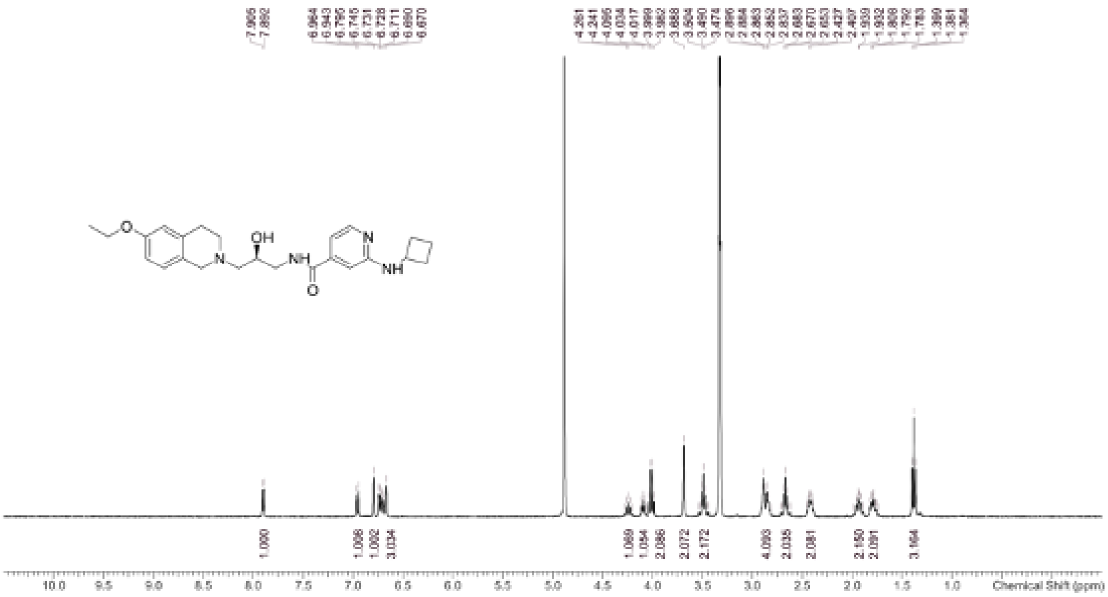

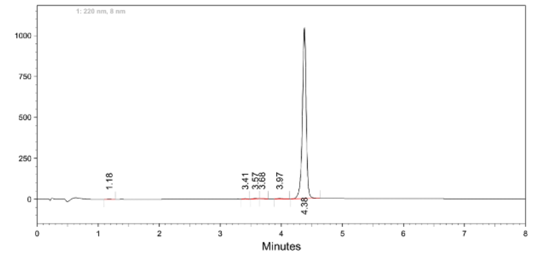

1: 220 nm, 8 nm

Retention Time	*Height*	*Area*	*Area Percent*
1.18	1739	8071	0.18
3.41	2251	7354	0.16
3.57	3200	12043	0.26
3.68	1685	5365	0.12
3.97	3417	16110	0.35
4.38	1038190	4512012	98.93

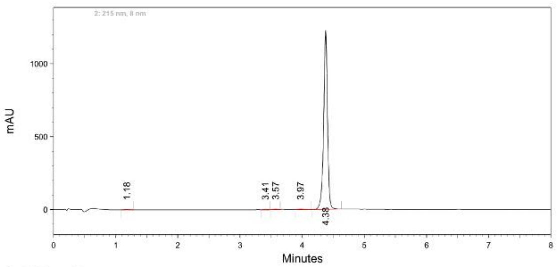

2: 215 nm, 8 nm

Retention Time	*Height*	*Area*	*Area Percent*
1.18	2543	11949	0.22
3.41	2487	8106	0.15
3.57	3625	14244	0.27
3.97	3753	17898	0.34
4.38	1217587	5260555	99.02

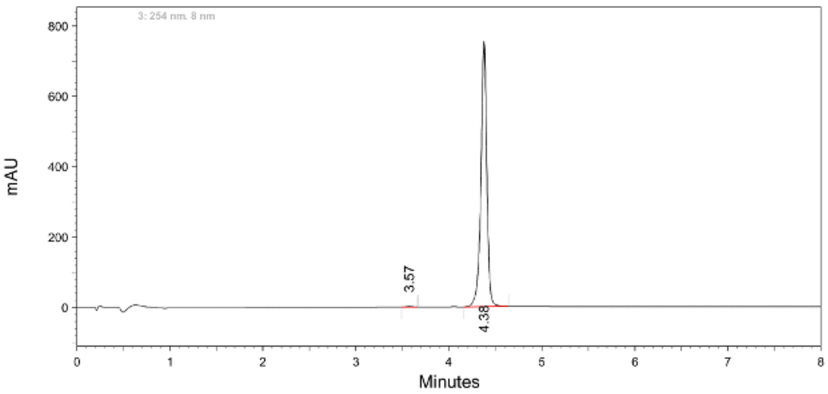

3: 254 nm, 8 nm

Retention Time	***Height***	***Area***	***Area Percent***
3.57	1948	8150	0.24
4.38	749633	3325233	99.76

```
Instrument          : LCMS AR
                      A:,Xtimate,2.1*30mm,3um
                      B:XBridge Shield,  2.1*50mm,5um
                      Confidential. For research only  NOT for regulatory fil
```

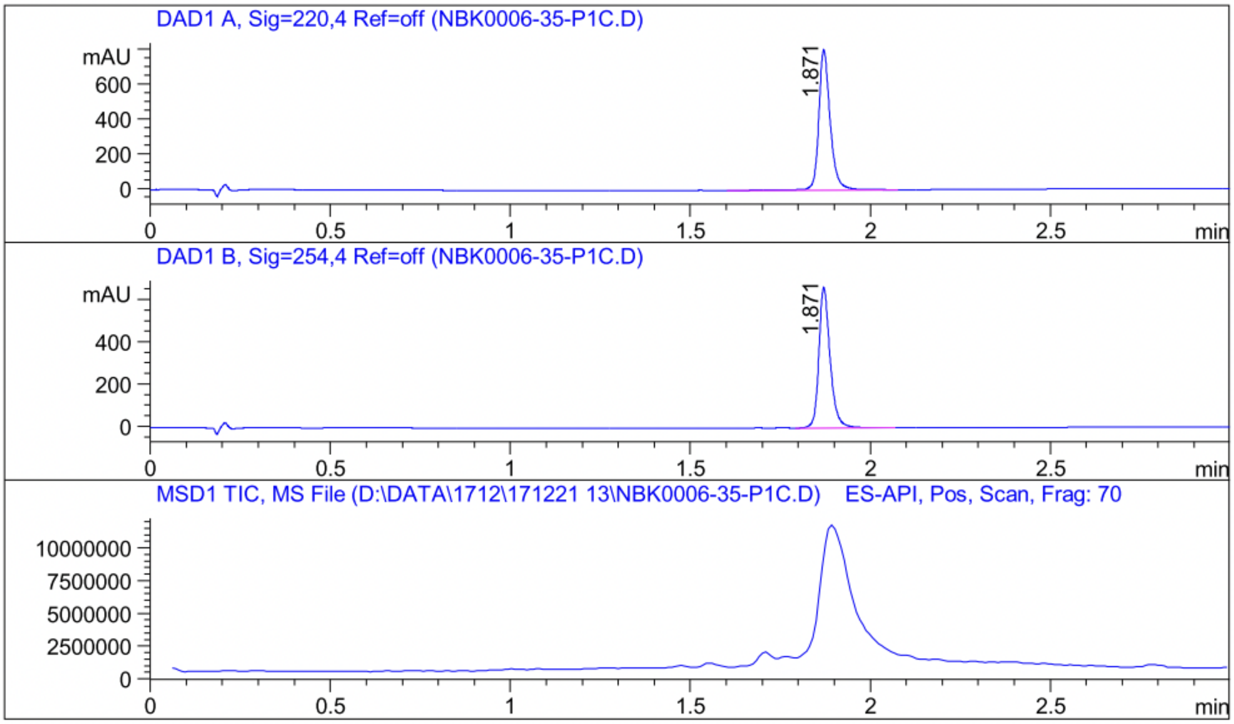

Report

===

Signal ->: DAD1 A, Sig=220,4 Ref=off

#	Meas. Ret.	Height	Width	Area	Area %
1	1.871	802.075	0.033	1785.370	100.000

Signal ->: DAD1 B, Sig=254,4 Ref=off

#	Meas. Ret.	Height	Width	Area	Area %
1	1.871	662.631	0.033	1450.208	100.000

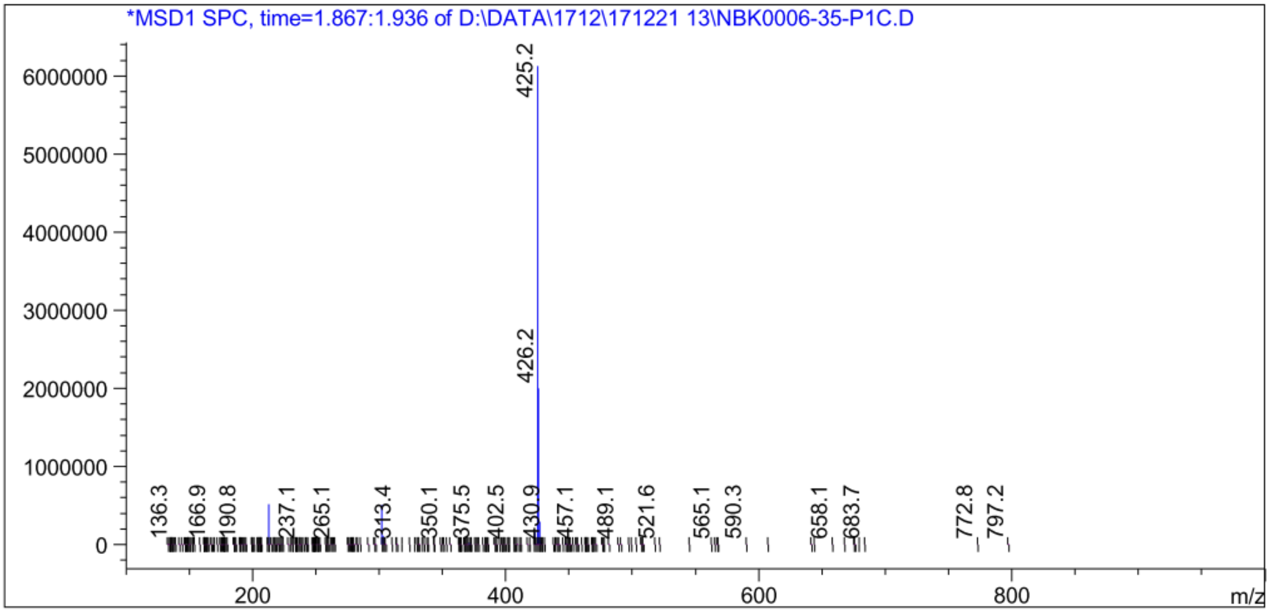


```
Method                 : Column: Chiralpak AD-3 50×3mm I.D., 3um
                         Mobile phase: A: CO2  B:ethanol (0.05% DEA)
                         Gradient:from 5% to 40% of B in 2.5 min and hold 40%
                         for 0.35 min, then from 40% to 5% of B for 0.15 min
                         Flow rate: 2.5mL/min    Column temperature:40 C
```

==

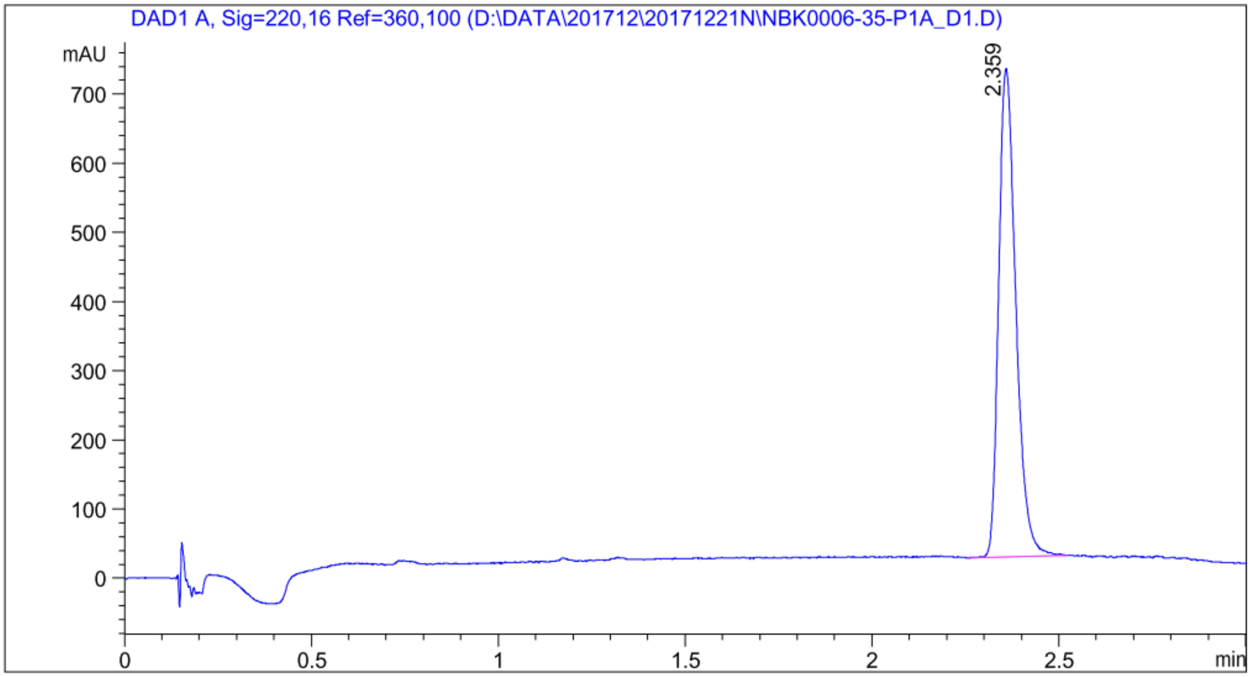

==

DAD1 A, Sig=220,16 Ref=360,100

#	Meas. Ret. Time	Height	Height %	Width	Area	Area %
1	2.359	705.637	100.000	0.049	2260.755	100.000

Compound 14

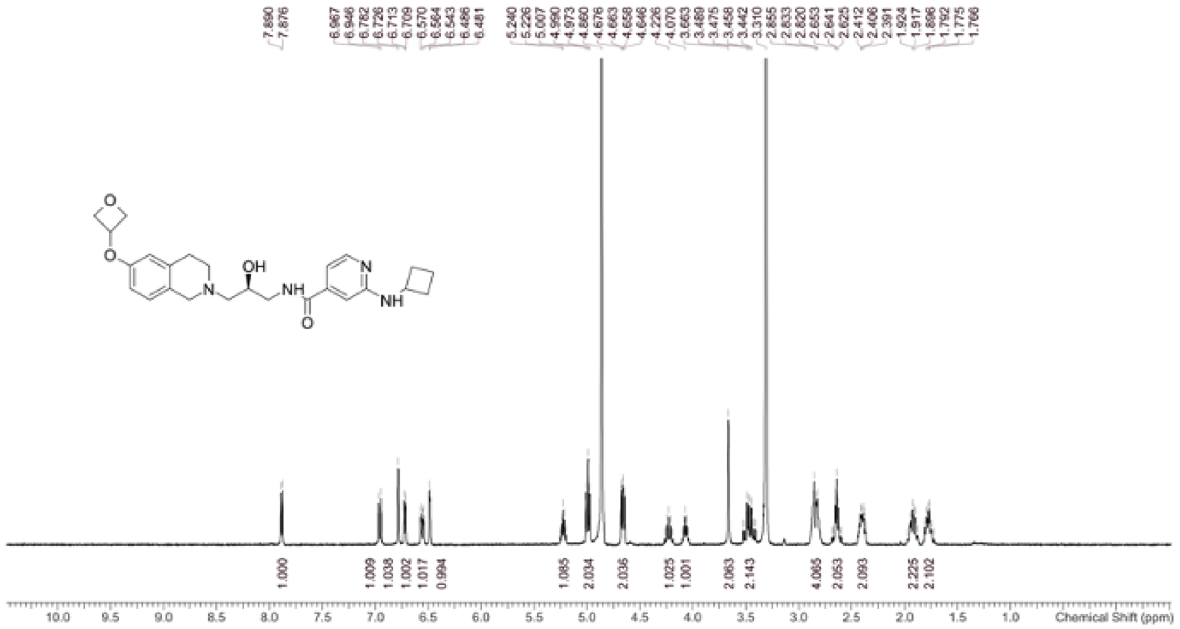

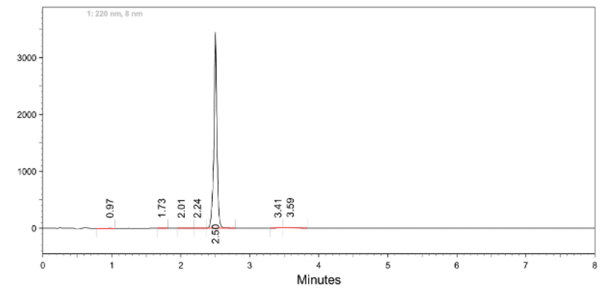

1: 220 nm, 8 nm

Retention Time	Height	Area	Area Percent
0.97	2274	19499	0.19
1.73	1270	5235	0.05
2.01	918	6037	0.06
2.24	1465	7835	0.07
2.50	3360347	10398534	99.20
3.41	722	5208	0.05
3.59	3913	39801	0.38

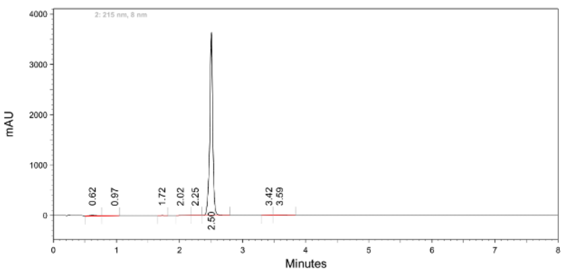

2: 215 nm, 8 nm

Retention Time	Height	Area	Area Percent
0.62	19046	178934	1.45
0.97	3259	54557	0.44
1.72	1400	5761	0.05
2.02	1169	7427	0.06
2.25	2031	10750	0.09
2.50	3544644	12048142	97.51
3.42	817	6263	0.05
3.59	4362	43836	0.35

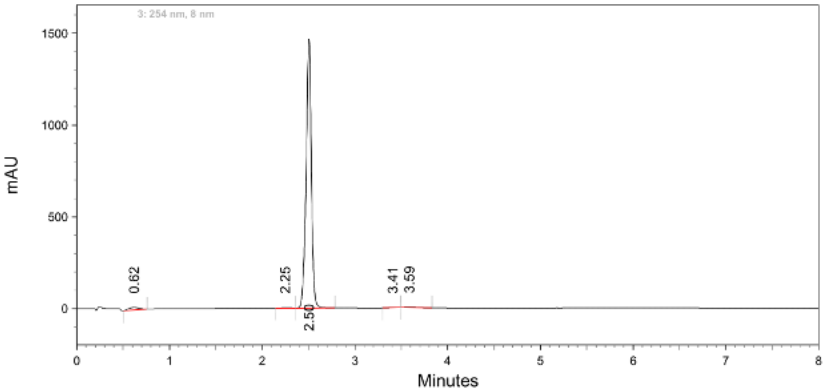

3: 254 nm, 8 nm

Retention Time	Height	Area	Area Percent
0.62	13787	115346	1.95
2.25	2819	16418	0.28
2.50	1448074	5750218	97.17
3.41	957	6439	0.11
3.59	3053	29389	0.50

```
Instrument          : LCMS AR
                      A:,Xtimate,2.1*30mm,3um
                      B:XBridge Shield,  2.1*50mm,5um
                      Confidential. For research only  NOT for regulatory fil:
```

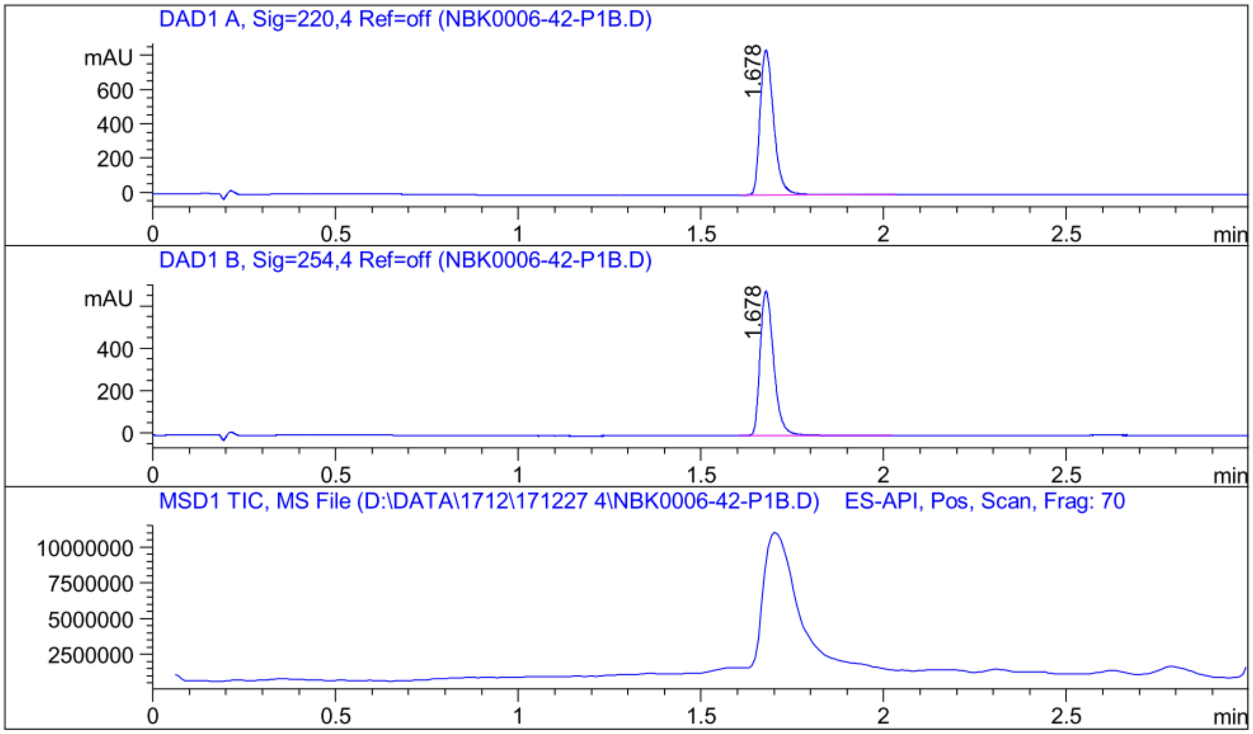

Report

==

Signal ->: DAD1 A, Sig=220,4 Ref=off

#	Meas. Ret.	Height	Width	Area	Area %
1	1.678	843.397	0.041	2203.375	100.000

Signal ->: DAD1 B, Sig=254,4 Ref=off

#	Meas. Ret.	Height	Width	Area	Area %
1	1.678	679.746	0.041	1770.988	100.000

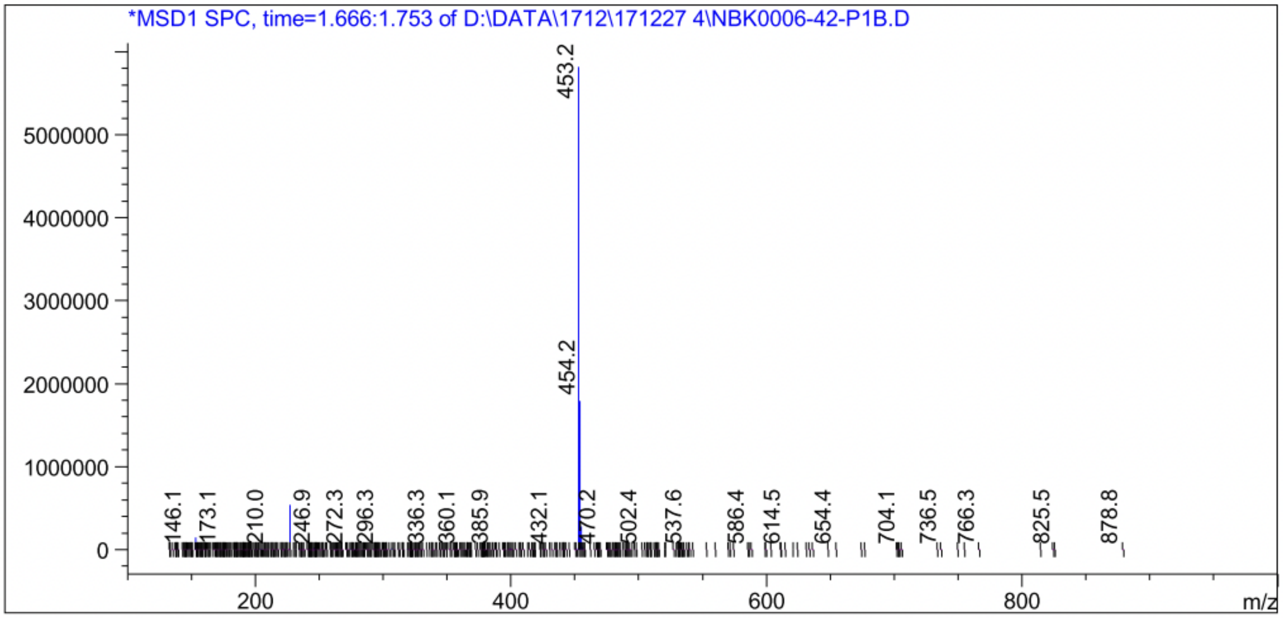
*MSD1 SPC, time=1.666:1.753 of D:\DATA\1712\171227 4\NBK0006-42-P1B.D
453.2
454.2
5000000
4000000
3000000
2000000
1000000
0
146.1
173.1
210.0
246.9
272.3
296.3
336.3
360.1
385.9
432.1
470.2
502.4
537.6
586.4
614.5
654.4
704.1
736.5
766.3
825.5
878.8
200
400
600
800
m/z

Method :Column: Chiralcel OJ-H 150*4.6mm I.D., 5um
Mobile phase: A:CO2 B:ethanol (0.05% DEA)
Gradient: hold 5% for 0.5 min,then from 5% to 40% of B in 3.5 min and hold 40% for 2.5 min, then 5% of B for 1.5 min
Flow rate: 3mL/min Column temp:40 C

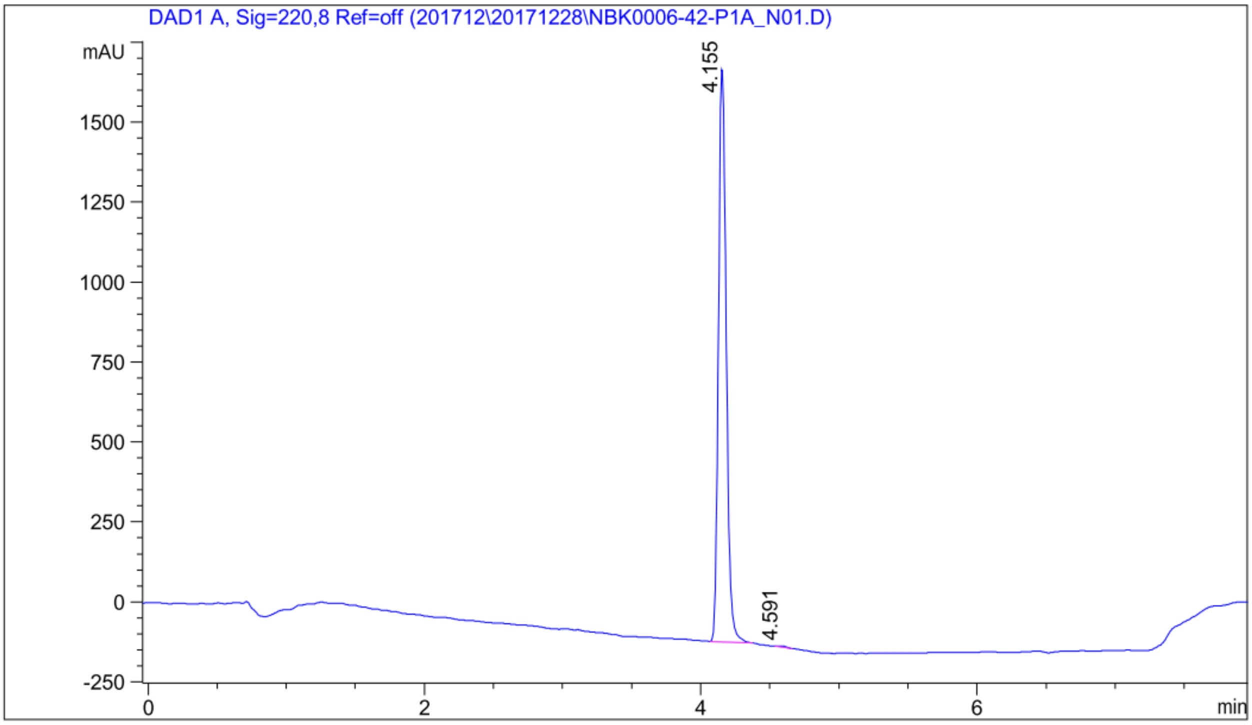

Signal 1 : DAD1 A, Sig=220,8 Ref=off

Peak	Meas. Ret. Time	Height	Height %	Width	Area	Area %
1	4.155	1806.886	99.754	0.065	7048.172	99.755
2	4.591	4.448	0.246	0.065	17.305	0.245

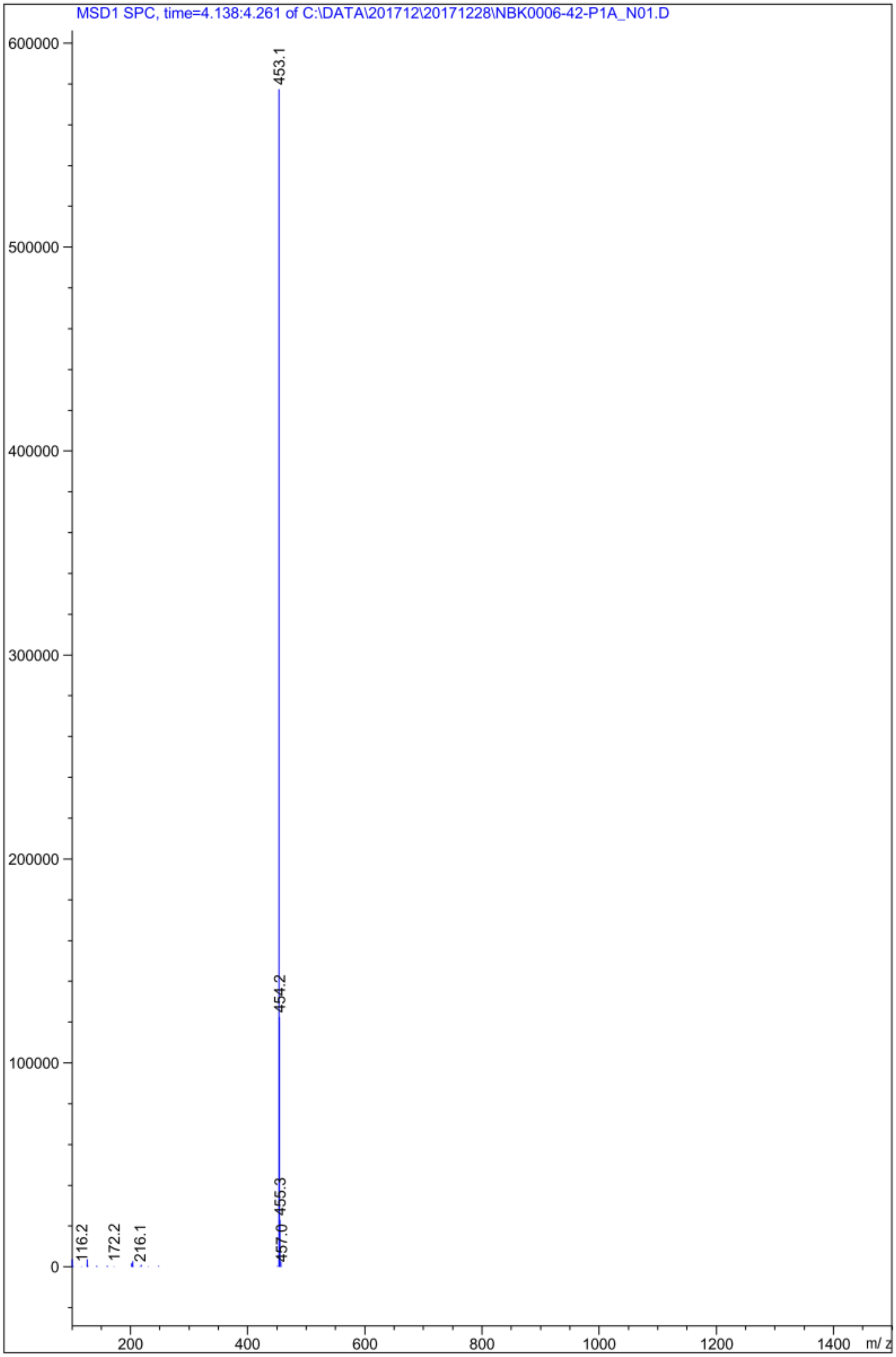
MSD1 SPC, time=4.138:4.261 of C:\DATA\201712\20171228\NBK0006-42-P1A_N01.D
600000
500000
400000
300000
200000
100000
0
453.1
454.2
455.3
457.0
116.2
172.2
216.1
200
400
600
800
1000
1200
1400
m/z

Compound 15

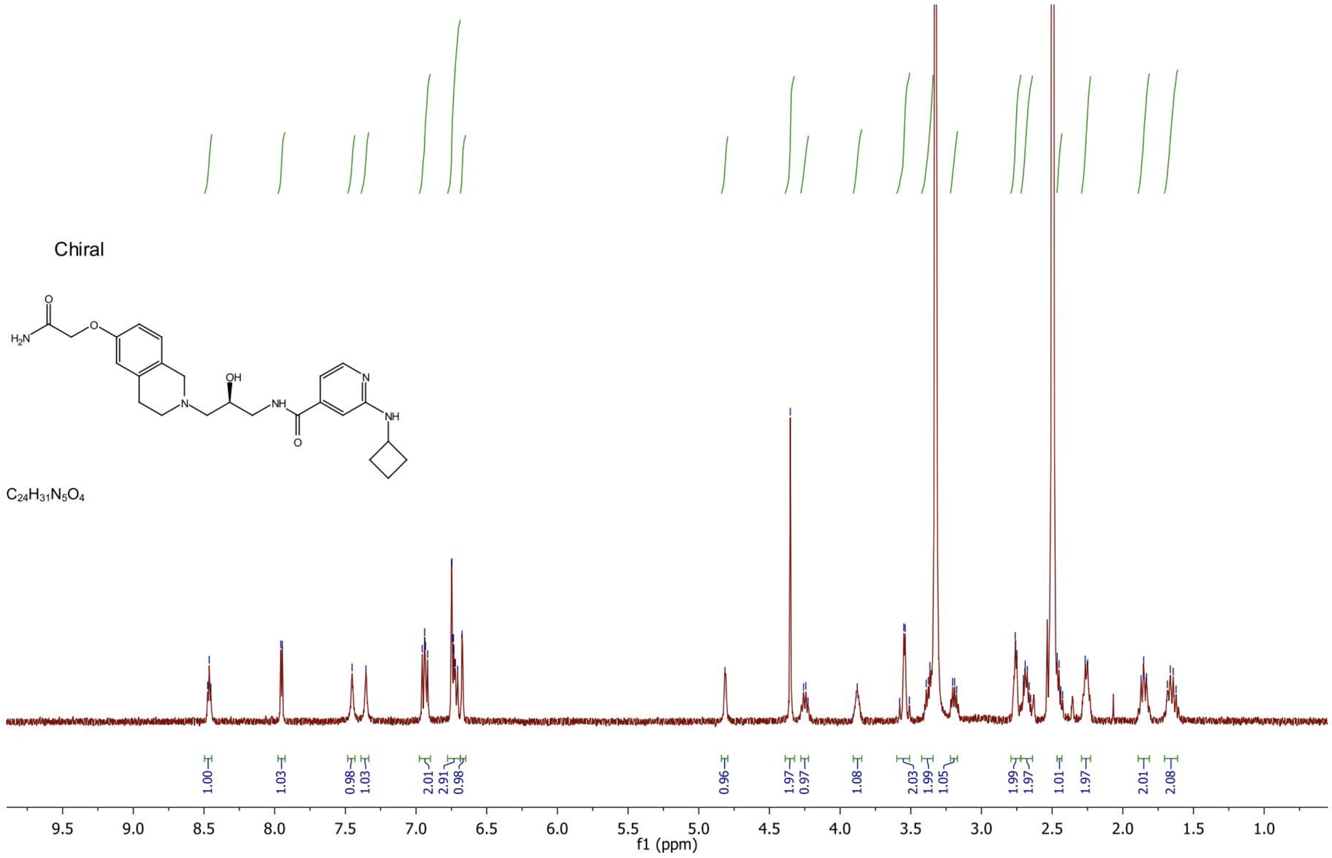

Chiral
C24H31N5O4
f1 (ppm)

MaxPeak: 100.00%
Ret_Time: 0.695 min

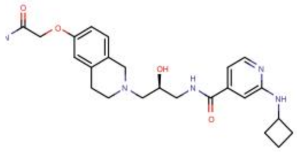

Mol Wt 453.53
Exact Mass 453.27

#	Time	Area%
1	0.695	100.00

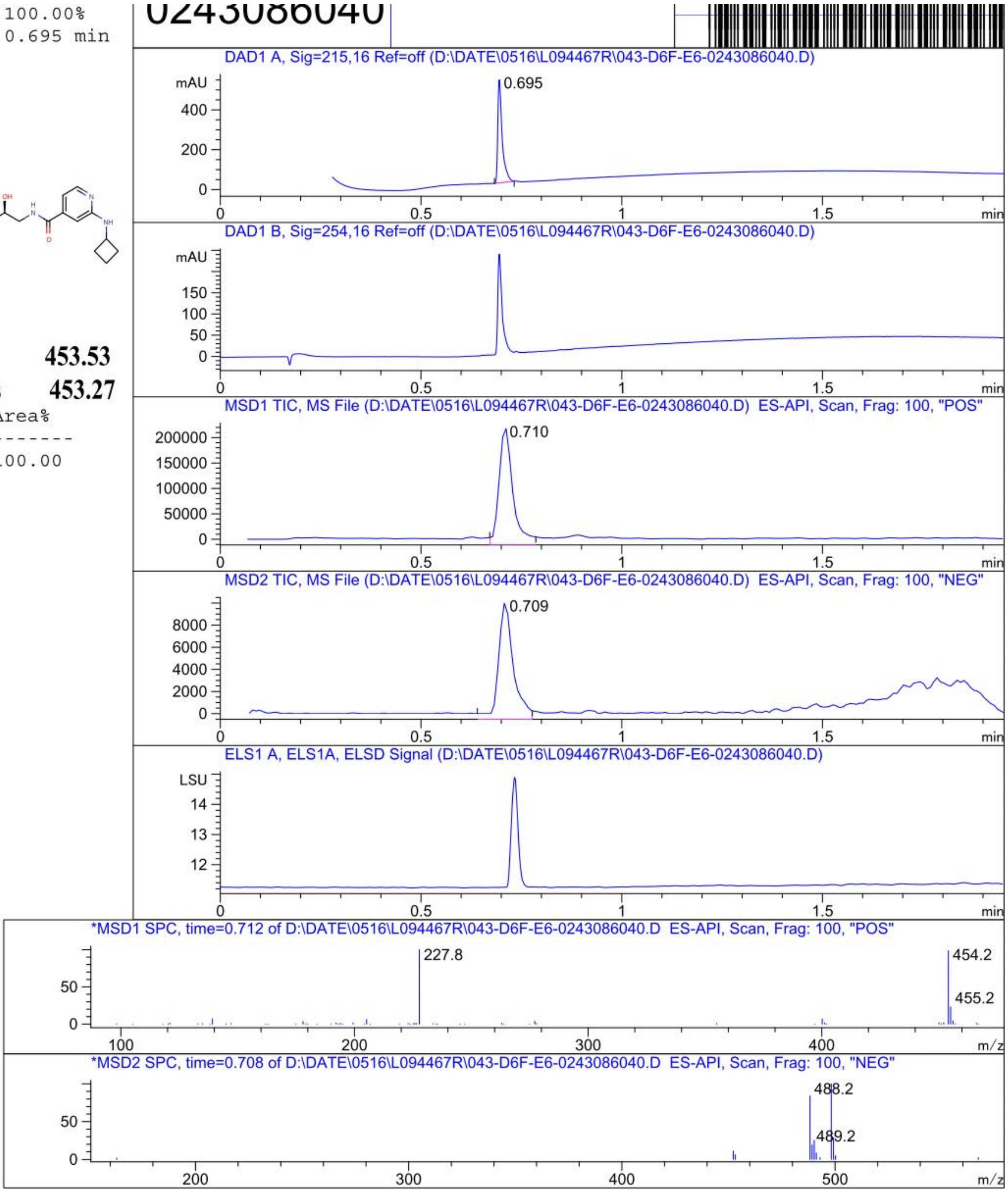

RT 0.710

RT 0.709

Compound 16

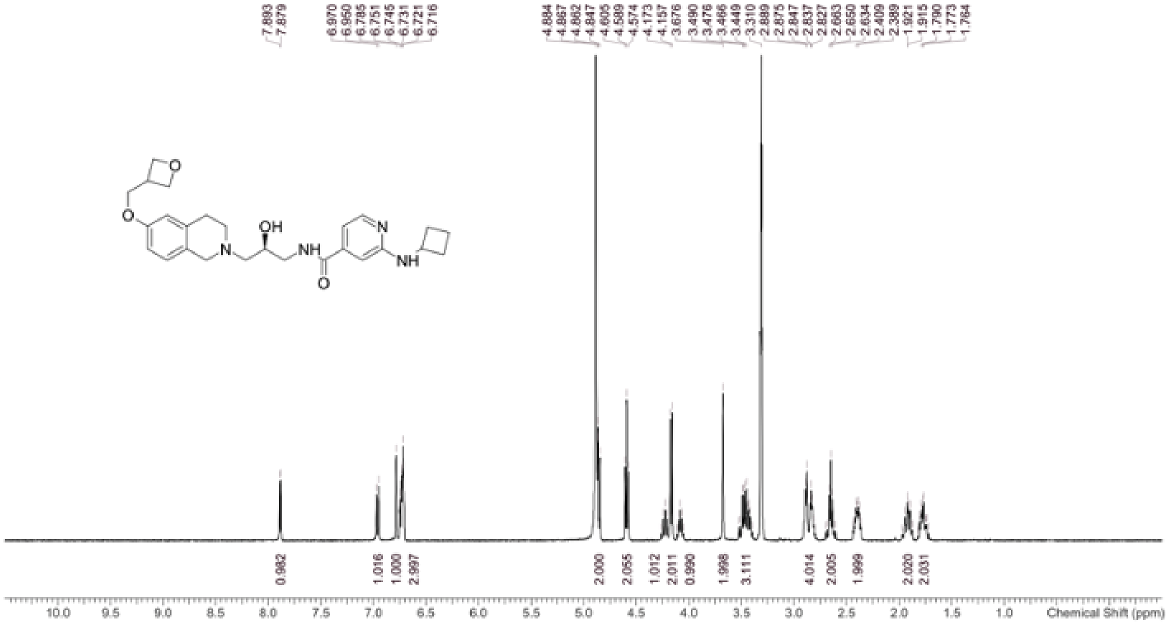

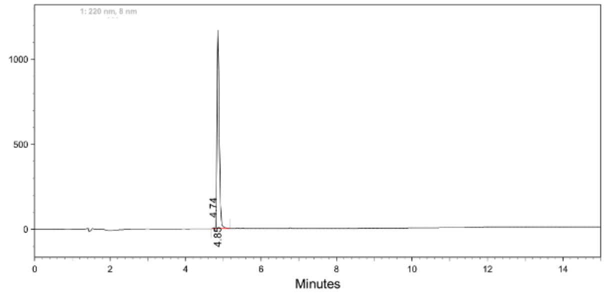

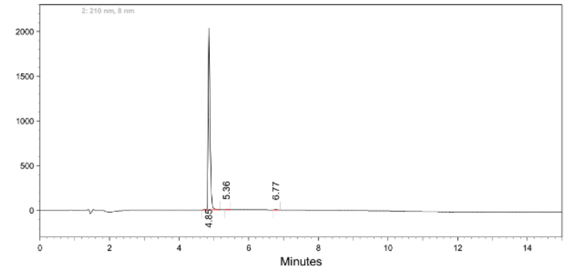

1: 220 nm, 8 nm

Retention Time	*Height*	*Area*	*Area Percent*
4.74	4135	14167	0.29
4.85	1168643	4817350	99.71

2: 210 nm, 8 nm

Retention Time	*Height*	*Area*	*Area Percent*
4.85	2034549	7554235	99.52
5.36	4550	19073	0.25
6.77	4979	17633	0.23

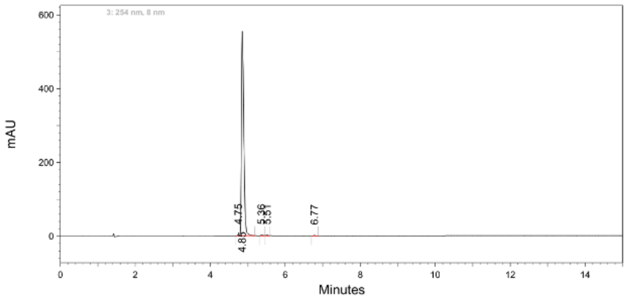

3: 254 nm, 8 nm

Retention Time	*Height*	*Area*	*Area Percent*
4.75	2619	8173	0.34
4.85	554004	2343002	98.87
5.36	1899	8040	0.34
5.51	1761	5305	0.22
6.77	1558	5220	0.22

```
Instrument          : LCMS AR
                      A:,Xtimate,2.1*30mm,3um
                      B:XBridge Shield,  2.1*50mm,5um
                      Confidential. For research only  NOT for regulatory filin
```

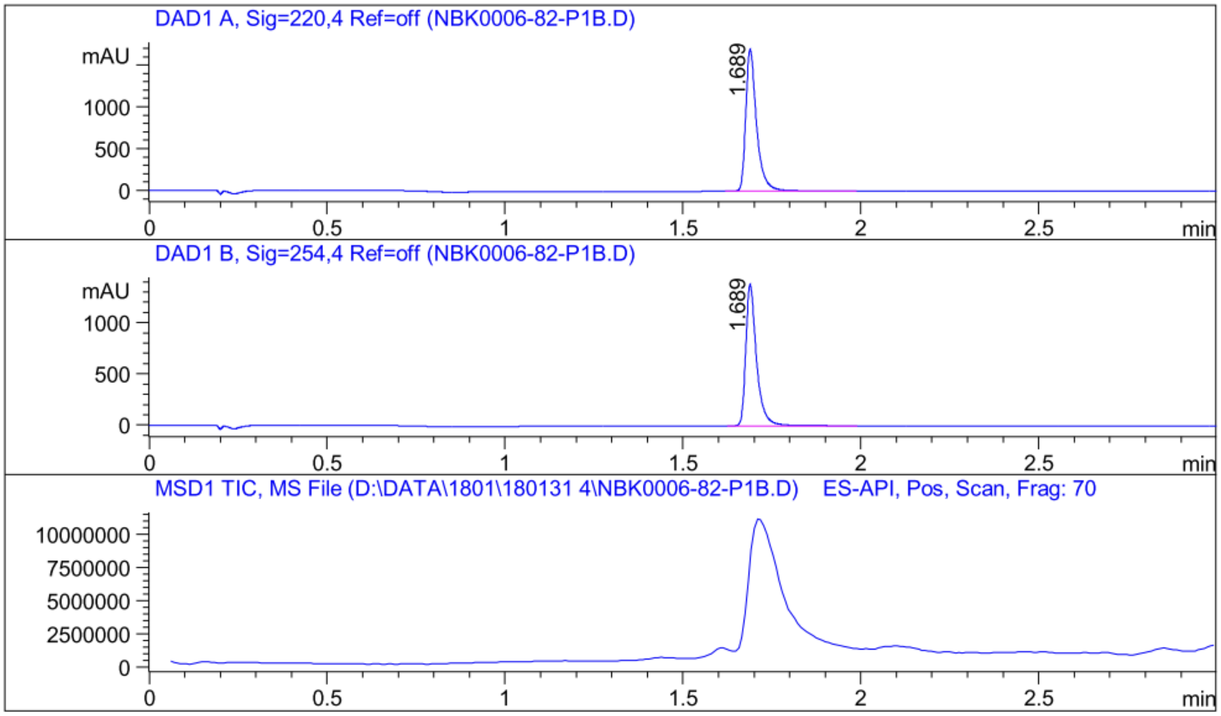


```
                              Report
=================================================================
   Signal ->: DAD1 A, Sig=220,4 Ref=off
    #  Meas. Ret.   Height    Width        Area        Area %
   --------------------------------------------------------
     1      1.689   1697.337   0.032       3610.857 100.000
   --------------------------------------------------------


   Signal ->: DAD1 B, Sig=254,4 Ref=off
    #  Meas. Ret.   Height    Width        Area        Area %
   --------------------------------------------------------
     1      1.689   1381.815   0.032       2923.837 100.000
   --------------------------------------------------------
```

√

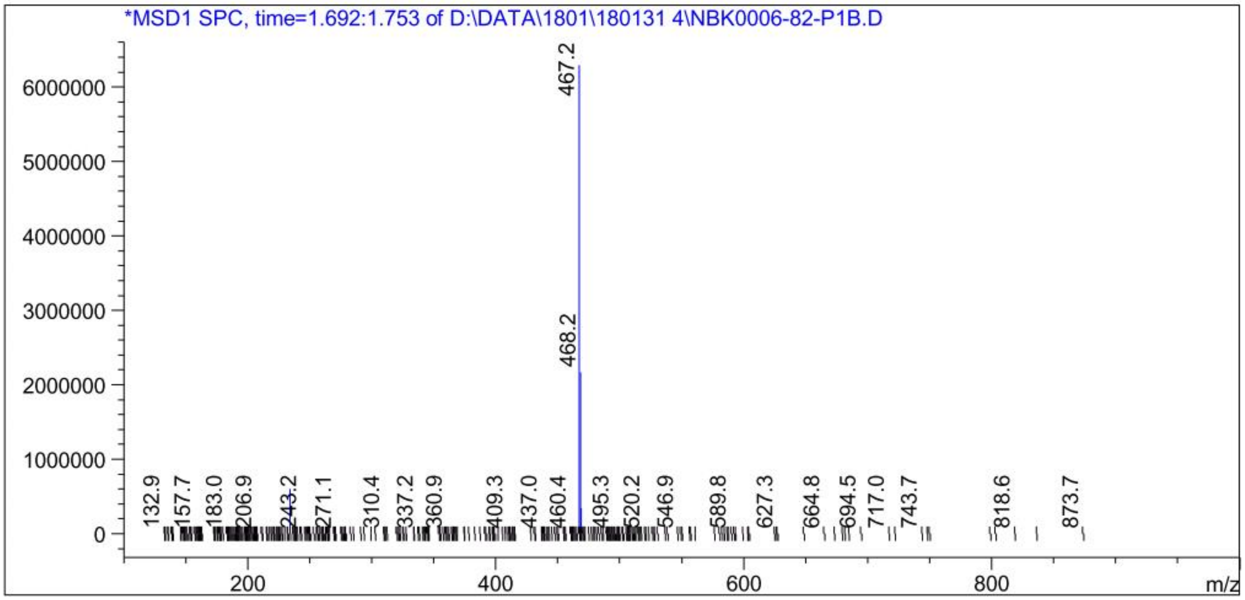
*MSD1 SPC, time=1.692:1.753 of D:\DATA\1801\180131 4\NBK0006-82-P1B.D
467.2
468.2
6000000
5000000
4000000
3000000
2000000
1000000
0
132.9
157.7
183.0
206.9
243.2
271.1
310.4
337.2
360.9
409.3
437.0
460.4
495.3
520.2
546.9
589.8
627.3
664.8
694.5
717.0
743.7
818.6
873.7
200
400
600
800
m/z

Method :Column: Chiralcel OJ-H 150*4.6mm I.D., 5um
Mobile phase: A:CO2 B:methanol(0.05% DEA)
Gradient: hold 5% for 0.5 min,then from 5% to 40% of B in 3.5 min and hold 40% for 2.5 min, then 5% of B for 1.5 min
Flow rate: 3mL/min Column temp:40 C

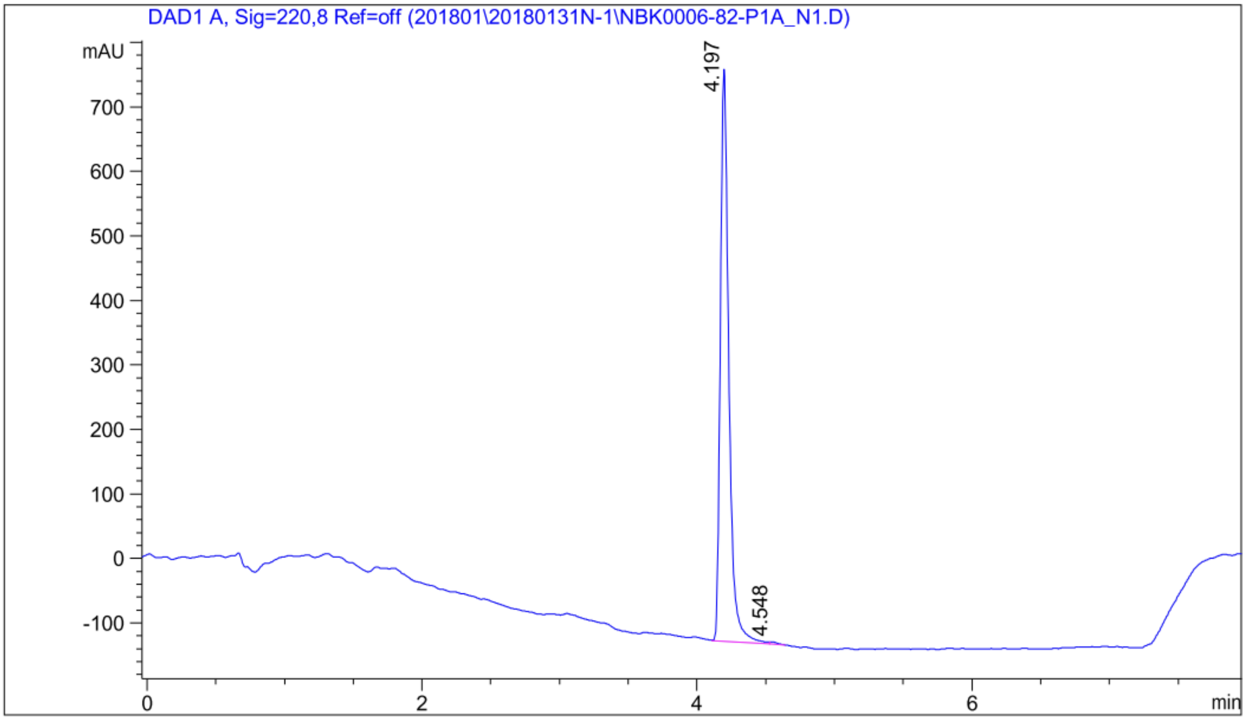

Signal 1 : DAD1 A, Sig=220,8 Ref=off

Peak	Meas. Ret. Time	Height	Height %	Width	Area	Area %
1	4.197	892.593	99.642	0.071	3801.493	99.710
2	4.548	3.206	0.358	0.057	11.045	0.290

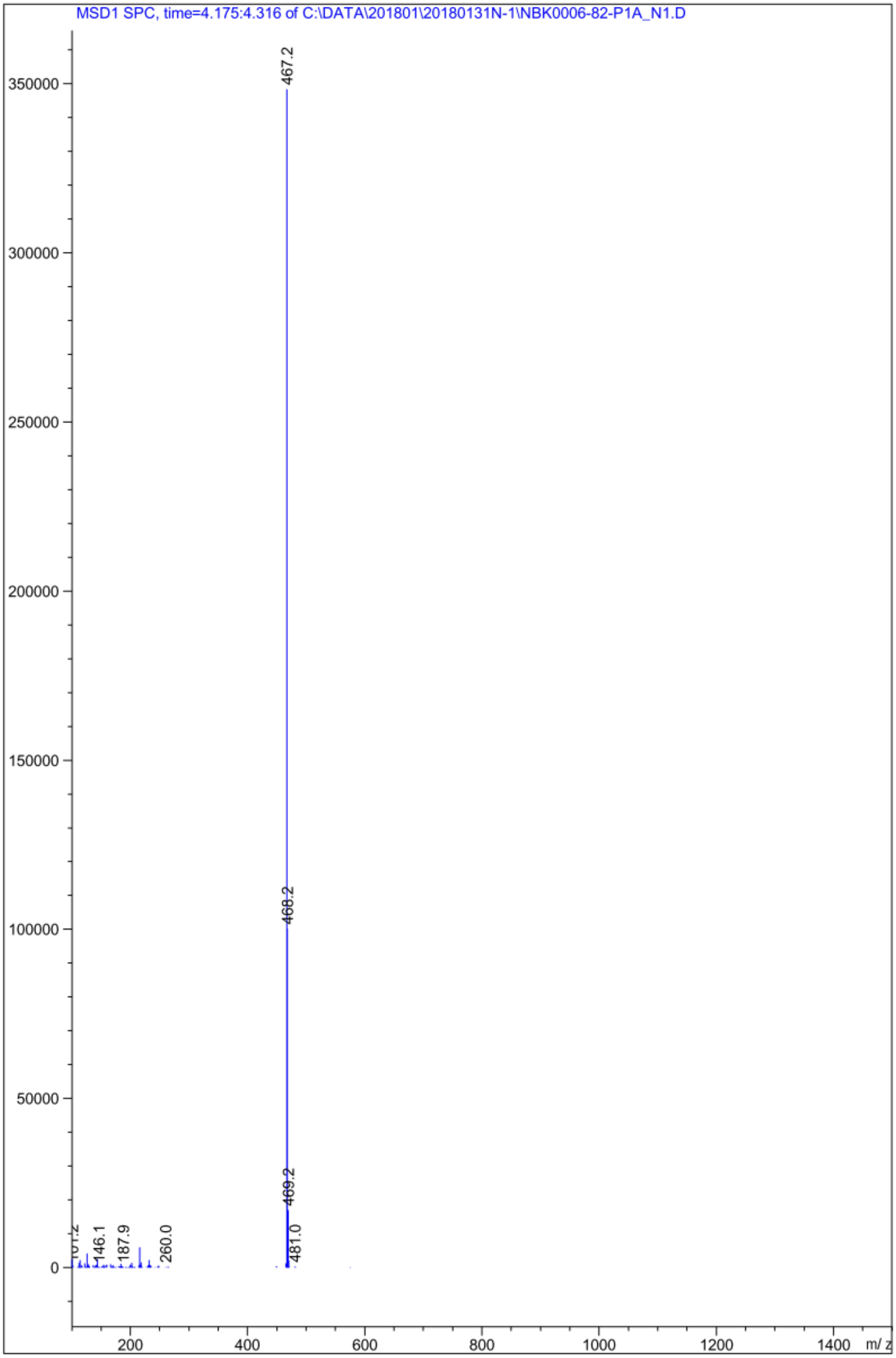

MSD1 SPC, time=4.175:4.316 of C:\DATA\201801\20180131N-1\NBK0006-82-P1A_N1.D
467.2
468.2
469.2
481.0
146.1
187.9
260.0
350000
300000
250000
200000
150000
100000
50000
0
200
400
600
800
1000
1200
1400
m/z

Compound 17

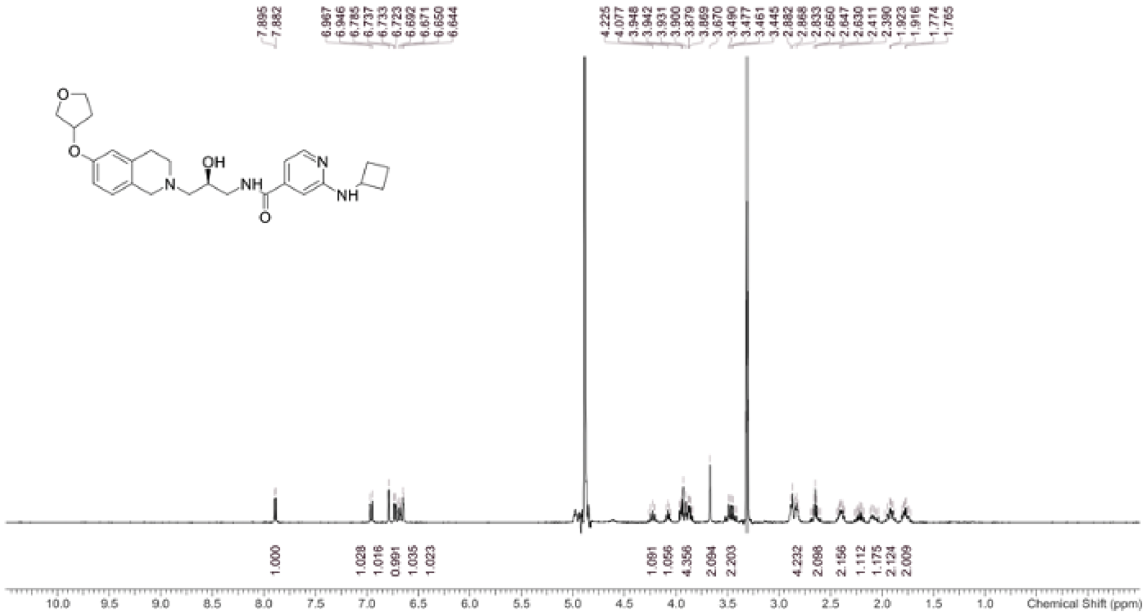

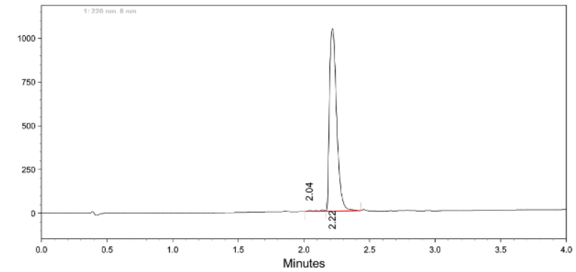

1: 220 nm, 8 nm

Retention Time	***Height***	***Area***	***Area Percent***
2.04	5102	36572	0.91
2.22	1032582	3973984	99.09

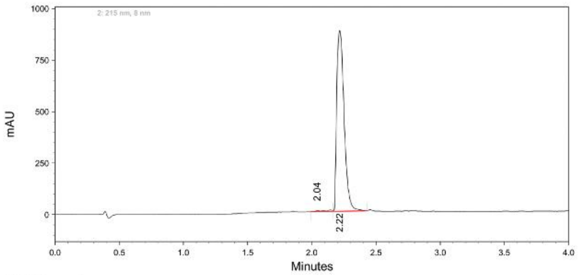

2: 215 nm, 8 nm

Retention Time	***Height***	***Area***	***Area Percent***
2.04	4705	35409	1.00
2.22	872338	3523096	99.00

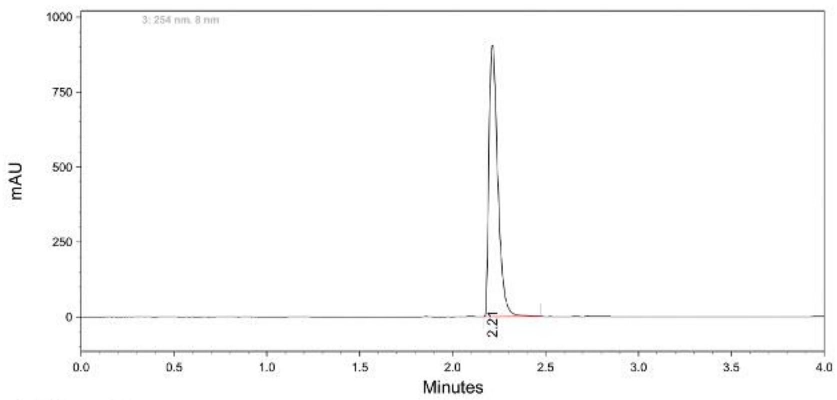

3: 254 nm, 8 nm

Retention Time	***Height***	***Area***	***Area Percent***
2.21	901751	3010612	100.00

```
Instrument          : LCMS AR
                      A:,Xtimate,2.1*30mm,3um
                      B:XBridge Shield,  2.1*50mm,5um
                      Confidential. For research only  NOT for regulatory fil
```

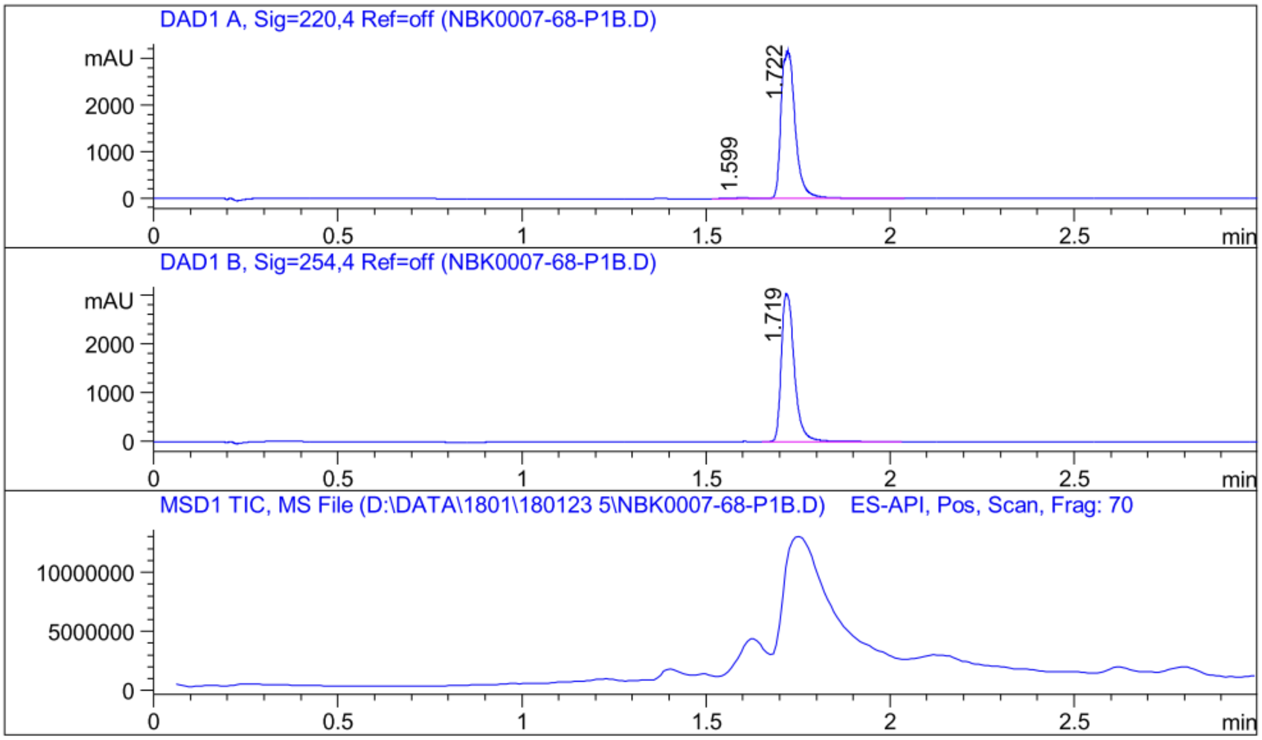

Report

Signal ->: DAD1 A, Sig=220,4 Ref=off

#	Meas. Ret.	Height	Width	Area	Area %
1	1.599	20.465	0.047	65.177	0.761
2	1.722	3146.111	0.043	8504.273	99.239

Signal ->: DAD1 B, Sig=254,4 Ref=off

#	Meas. Ret.	Height	Width	Area	Area %
1	1.719	3020.836	0.038	7464.604	100.000

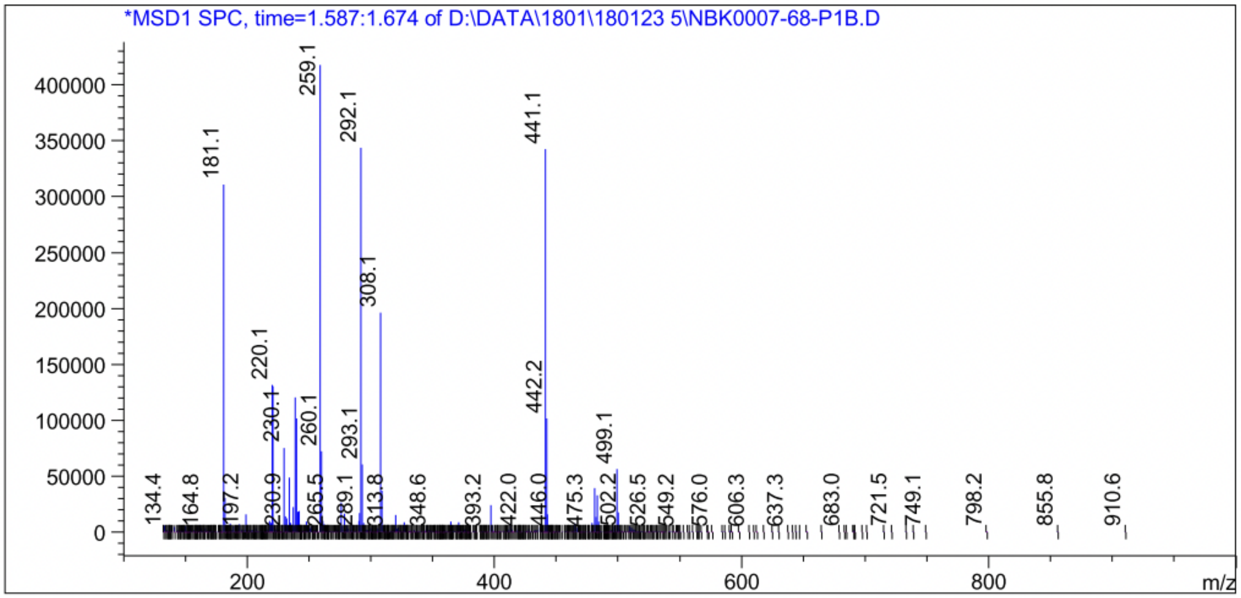
*MSD1 SPC, time=1.587:1.674 of D:\DATA\1801\180123 5\NBK0007-68-P1B.D
181.1
259.1
292.1
308.1
441.1
442.2
220.1
230.1
260.1
293.1
499.1
m/z

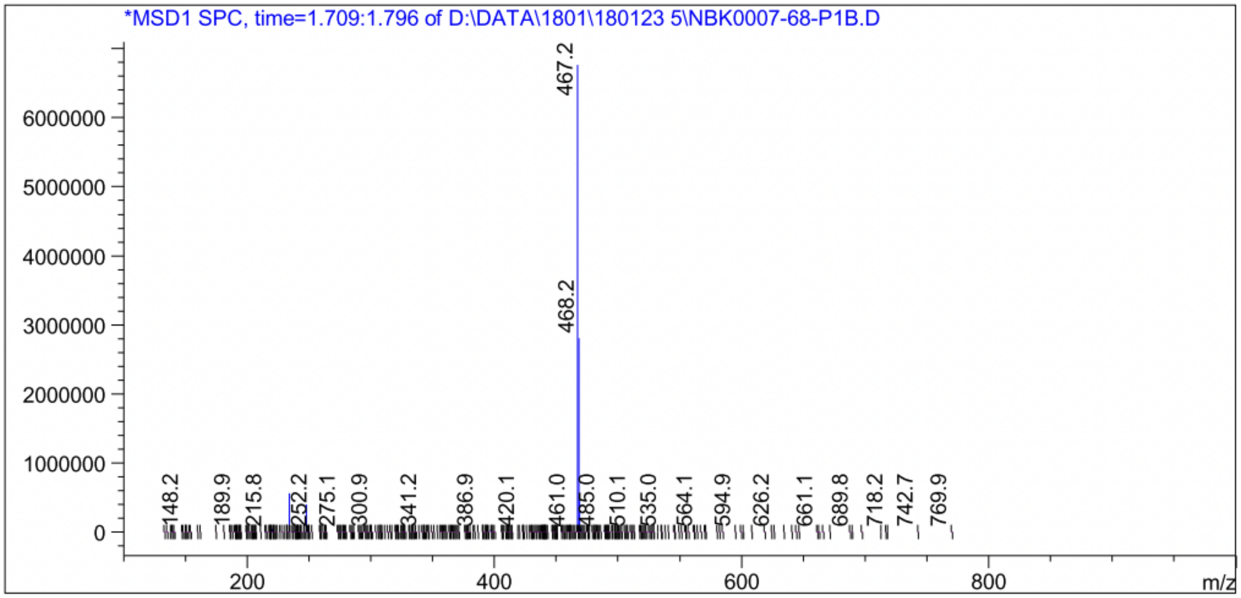
*MSD1 SPC, time=1.709:1.796 of D:\DATA\1801\180123 5\NBK0007-68-P1B.D
467.2
468.2
m/z

Method :Column: Chiralpak AY 100*4.6mm I.D., 3um
Mobile phase: 40% of ethanol (0.05% DEA) in CO2
Flow rate: 3mL/min
Column temp:40 C

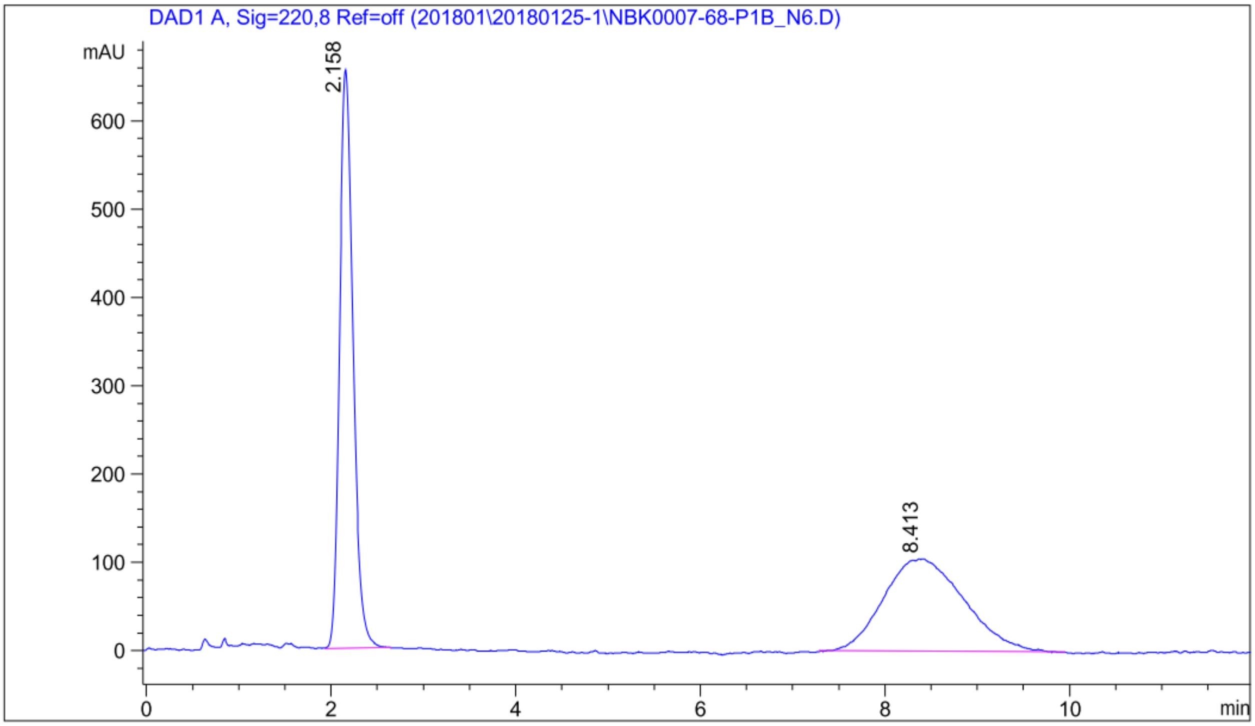

Signal 1 : DAD1 A, Sig=220,8 Ref=off

Peak	Meas. Ret. Time	Height	Height %	Width	Area	Area %
1	2.158	655.030	86.224	0.169	6640.981	51.221
2	8.413	104.657	13.776	1.007	6324.361	48.779

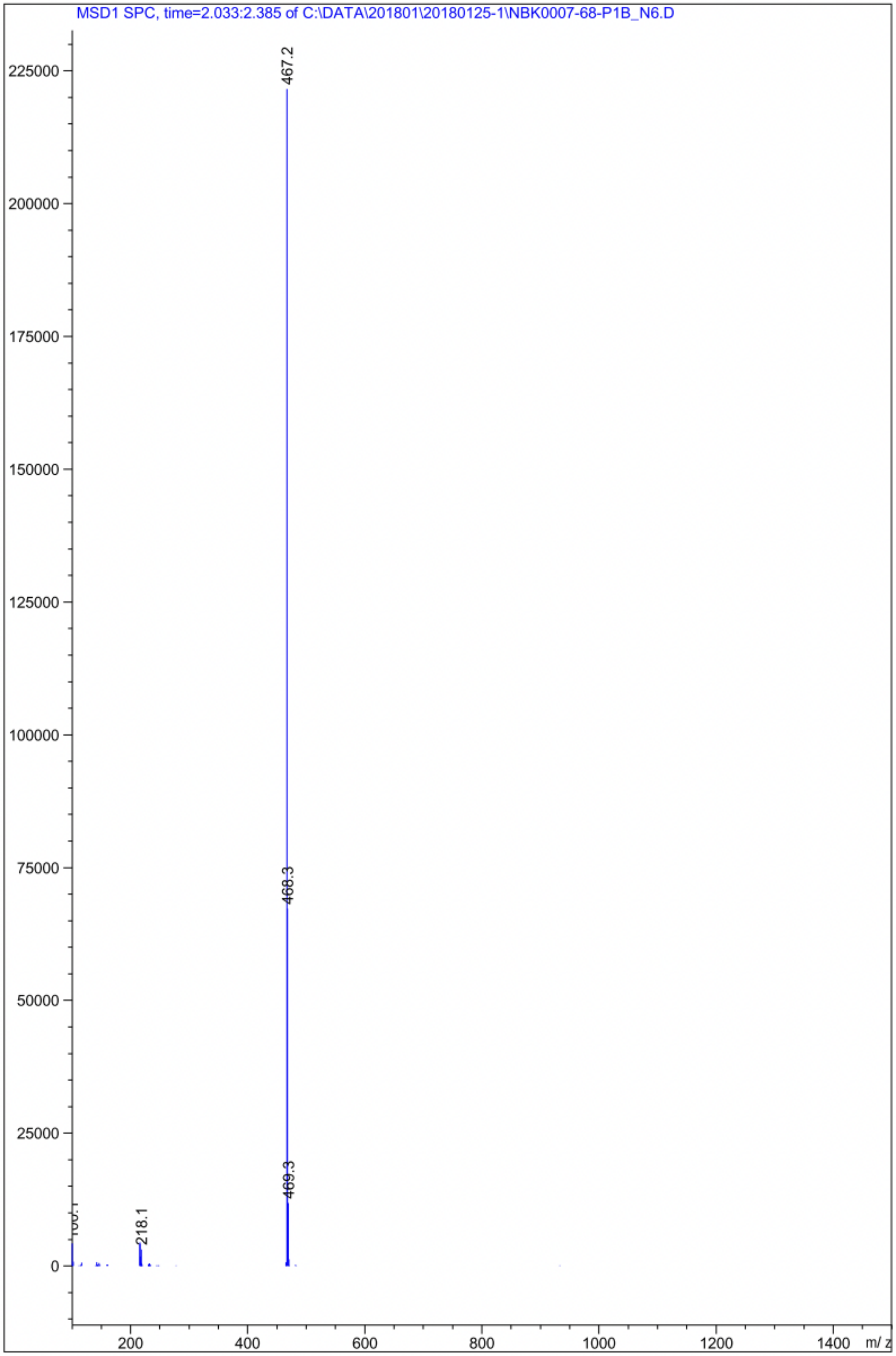
MSD1 SPC, time=2.033:2.385 of C:\DATA\201801\20180125-1\NBK0007-68-P1B_N6.D
467.2
468.3
469.3
218.1
225000
200000
175000
150000
125000
100000
75000
50000
25000
0
200
400
600
800
1000
1200
1400
m/z

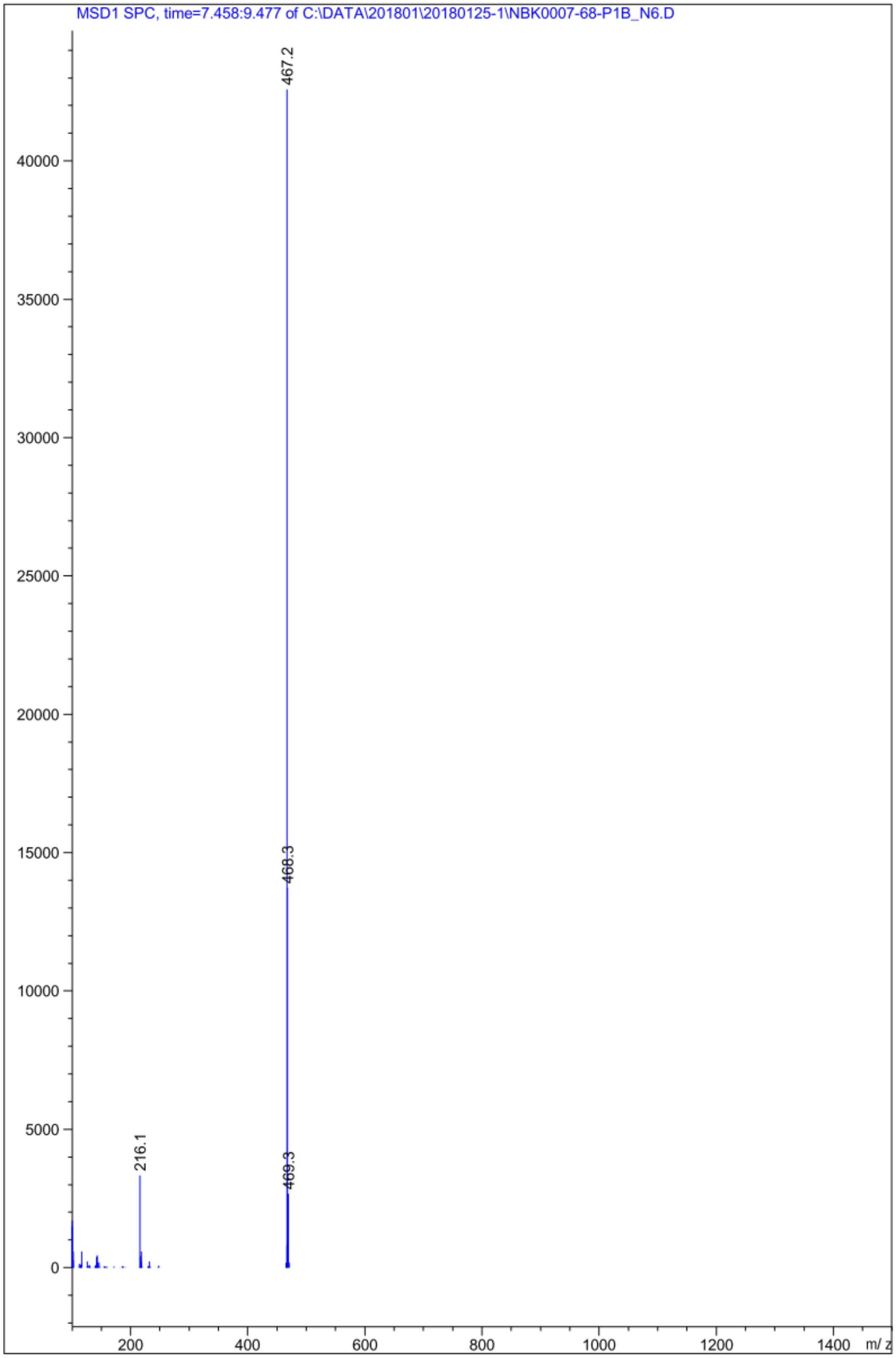
MSD1 SPC, time=7.458:9.477 of C:\DATA\201801\20180125-1\NBK0007-68-P1B_N6.D
467.2
468.3
469.3
216.1
40000
35000
30000
25000
20000
15000
10000
5000
0
200
400
600
800
1000
1200
1400
m/z

Compound 18

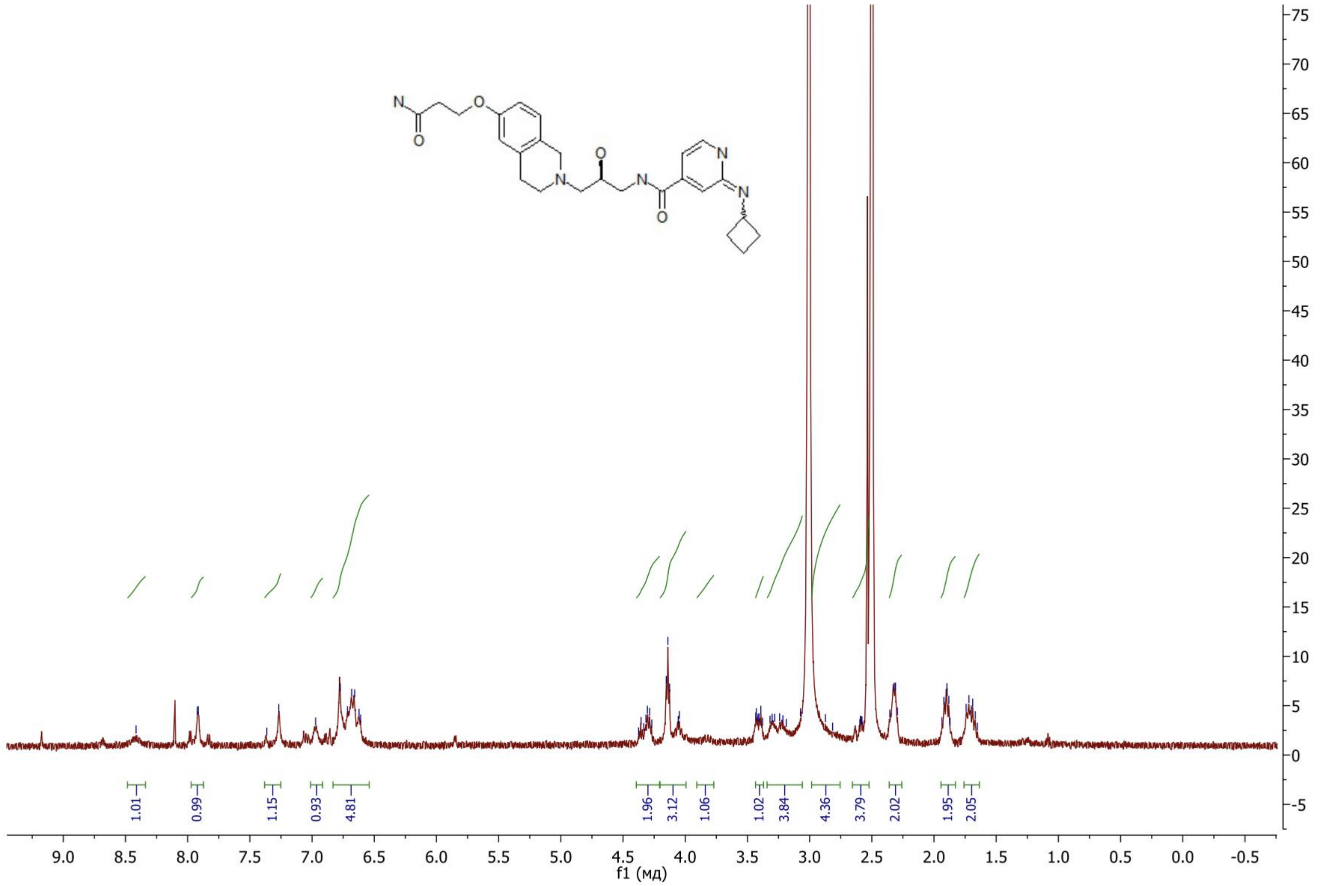

f1 (мд)

MaxPeak: 97.50%
Ret_Time: 0.673 min

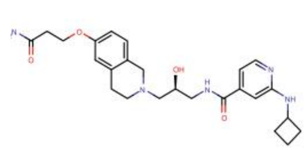

Mol Wt 467.56
Exact Mass 467.29

#	Time	Area%
1	0.673	97.50
2	0.695	2.50

RT 0.681

RT 0.684

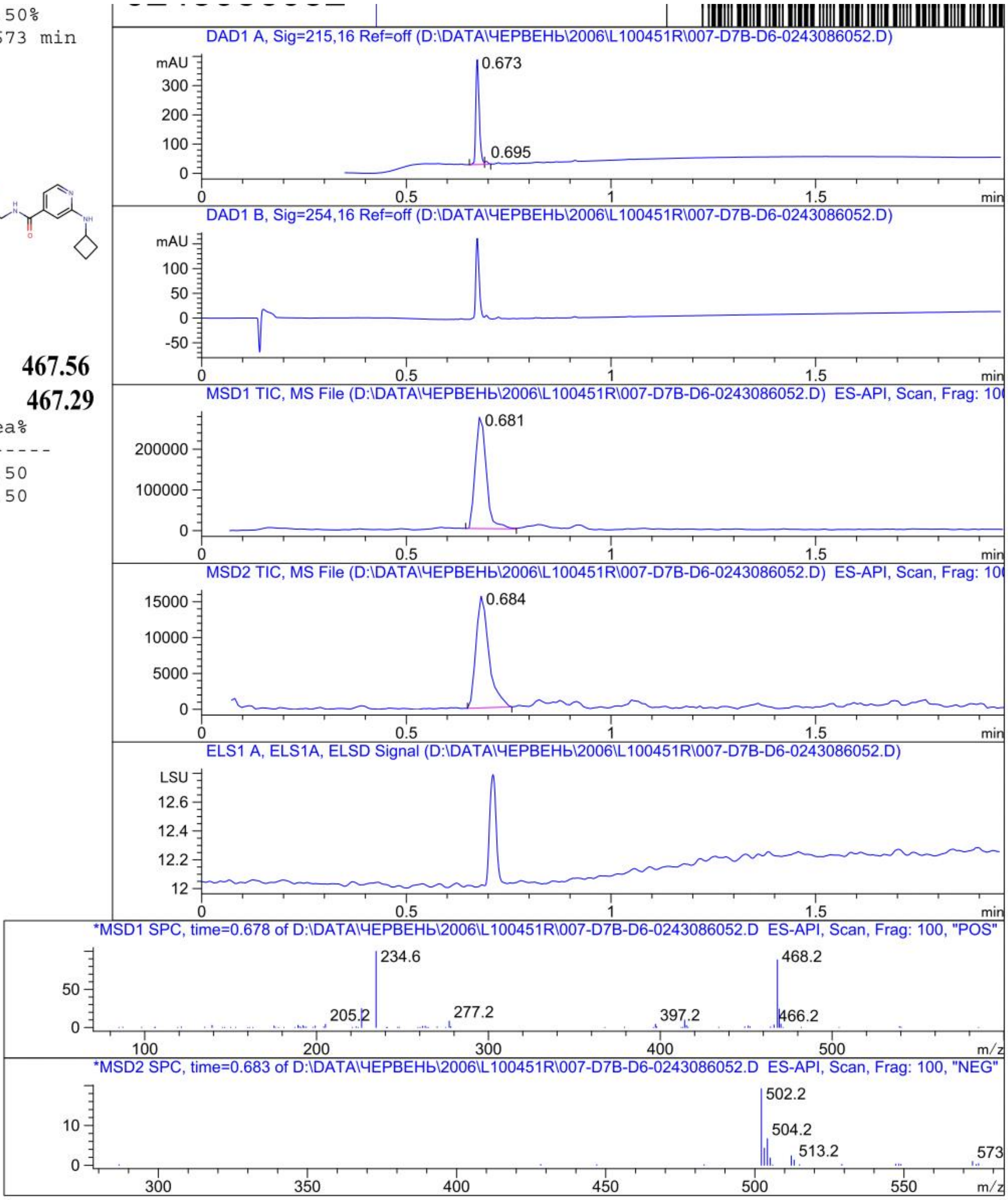

Compound 19

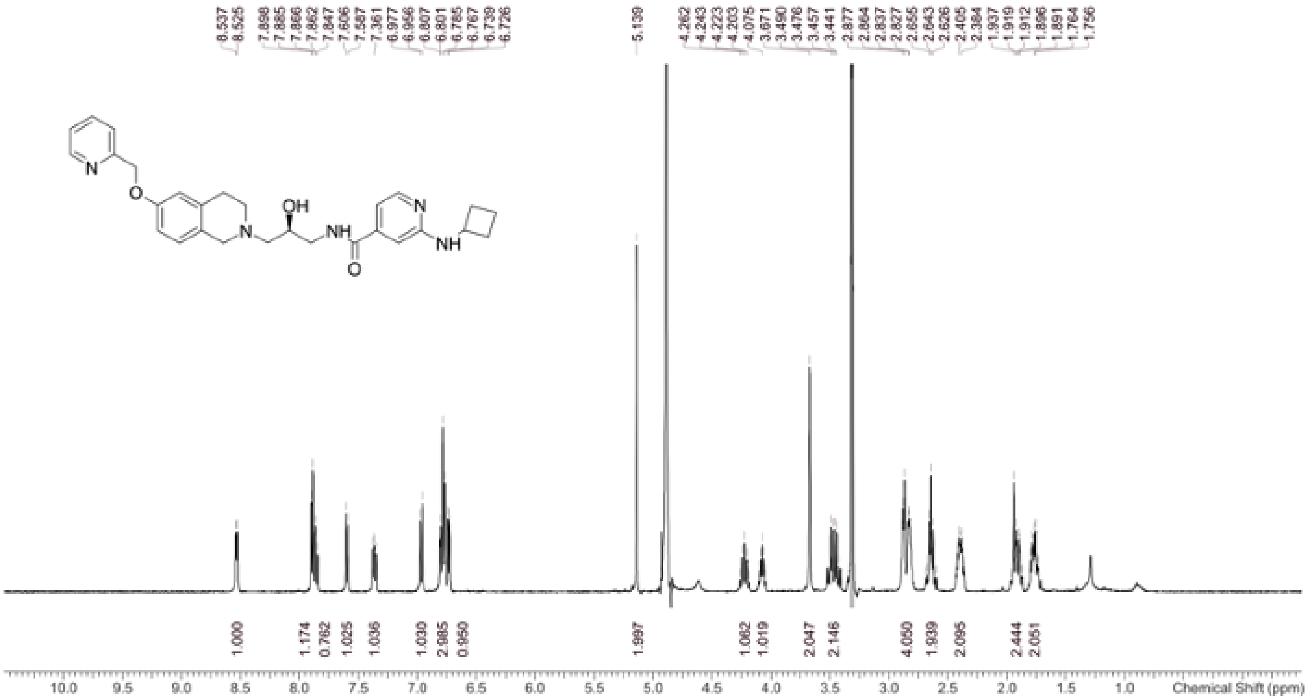

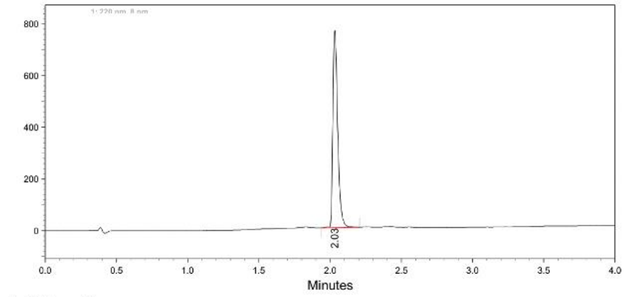

1: 220 nm, 8 nm

Retention Time	Height	Area	Area Percent
2.03	733288	1825663	100.00

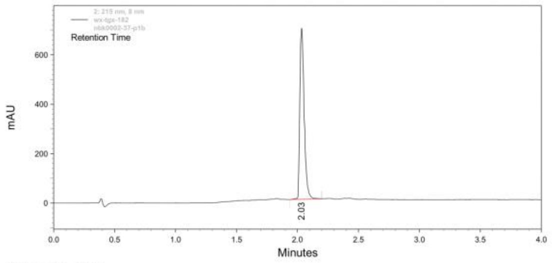

2: 215 nm, 8 nm

Retention Time	Height	Area	Area Percent
2.03	673576	1729929	100.00

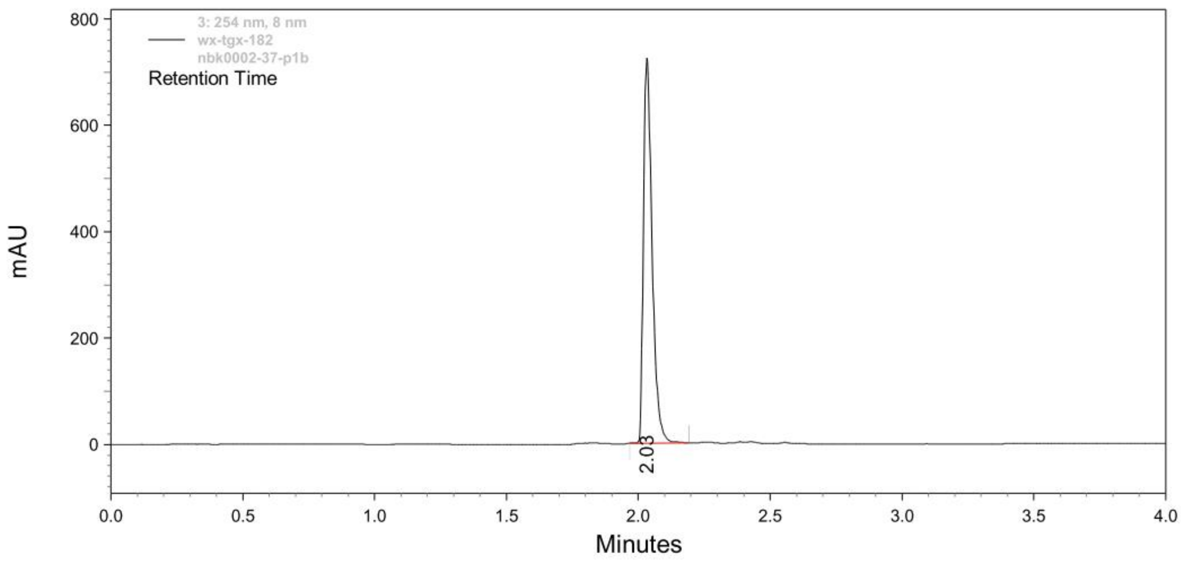

3: 254 nm, 8 nm

Retention Time	***Height***	***Area***	***Area Percent***
2.03	697223	1660360	100.00

Acq Method :D:\method\5-95AB_1.5MIN_220&254.lcm
Org DateFile :D:\DATA\1801\180108\NBK0002-37-P1B.lcd
Instrument & column:LCMS-BG 28-106
Agilent Pursit 5 C18 20*2.0mm

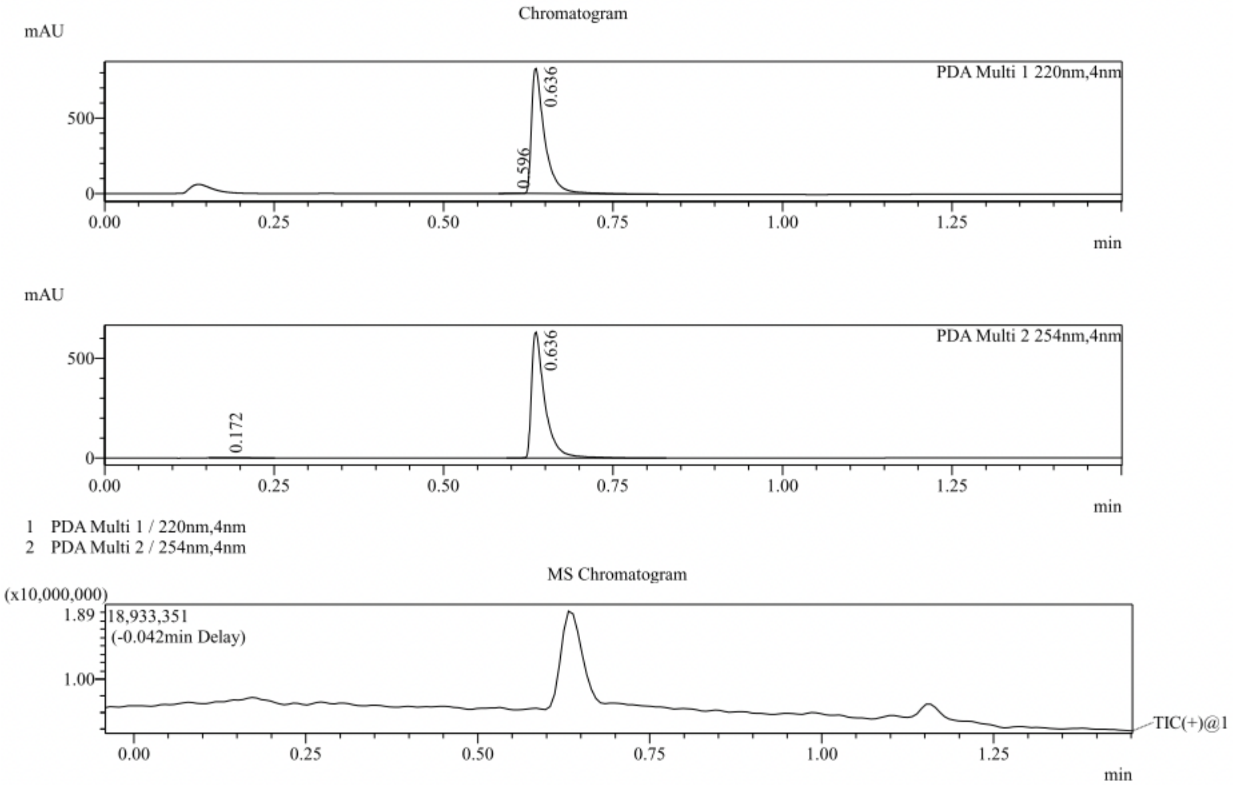

Integration Result

Peak Table

PDA Ch1 220nm

Peak#	Ret. Time	Height	Height%	USP Width	Area	Area%
1	0.596	504	0.061	0.024	525	0.047
2	0.636	818972	99.939	0.032	1116913	99.953

Peak Table

PDA Ch2 254nm

Peak#	Ret. Time	Height	Height%	USP Width	Area	Area%
1	0.172	1148	0.184	0.060	2700	0.312
2	0.636	622717	99.816	0.032	861686	99.688

Mass Spectrum
RetTime: 0.638 Datafile: D:\DATA\1801\180108\NBK0002-37-P1B.lcd

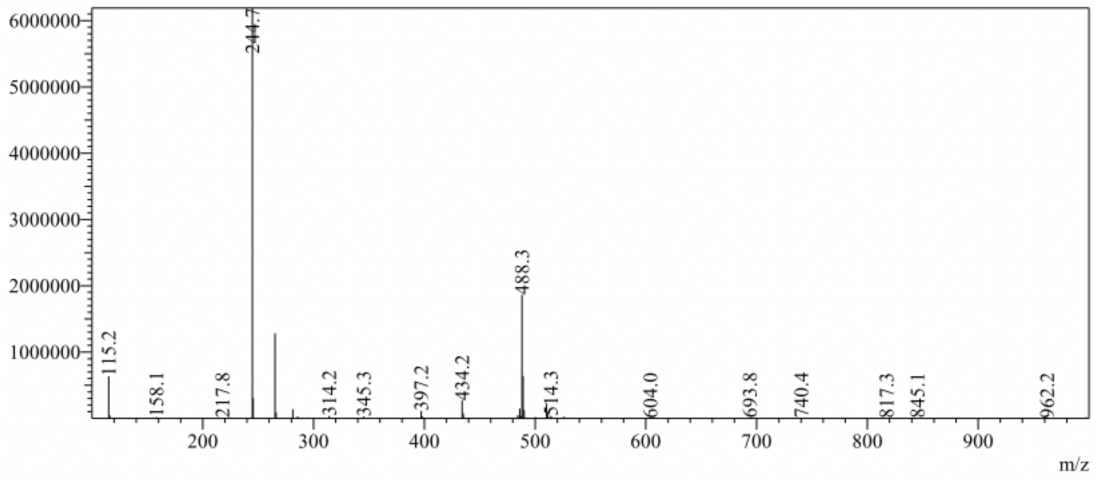


```
Method              :Column: Chiralpak AD-3 50*4.6mm I.D., 3um
                     Mobile phase: 40% of ethanol(0.1% ethanolamine) in CO2
                     Flow rate: 4mL/min
                     Column temp:40 C
```

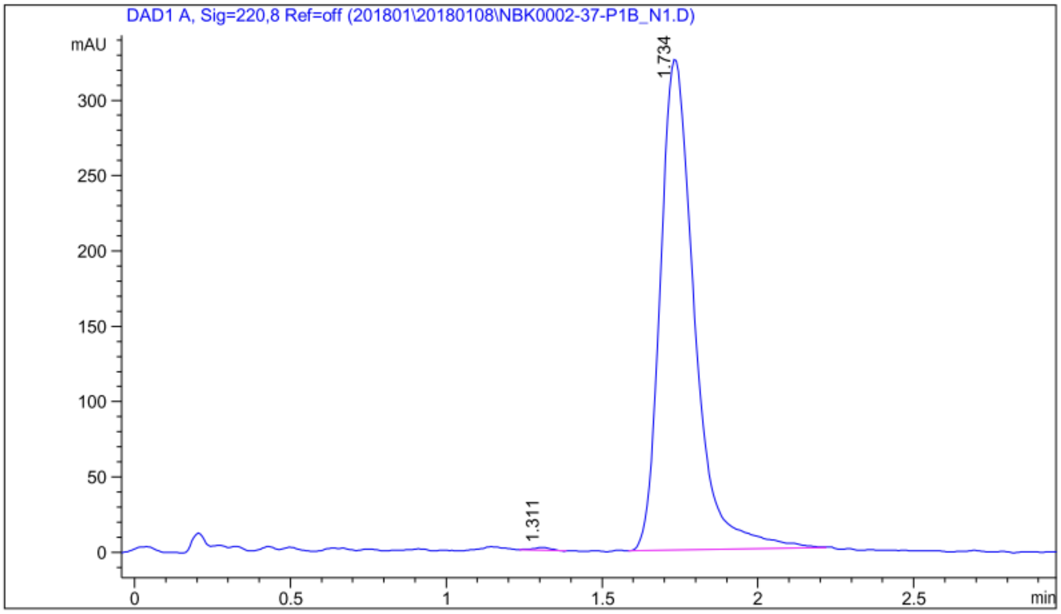

Signal 1 : DAD1 A, Sig=220,8 Ref=off

Peak	Meas. Ret. Time	Height	Height %	Width	Area	Area %
1	1.311	1.749	0.533	0.061	6.356	0.255
2	1.734	326.282	99.467	0.127	2483.069	99.745

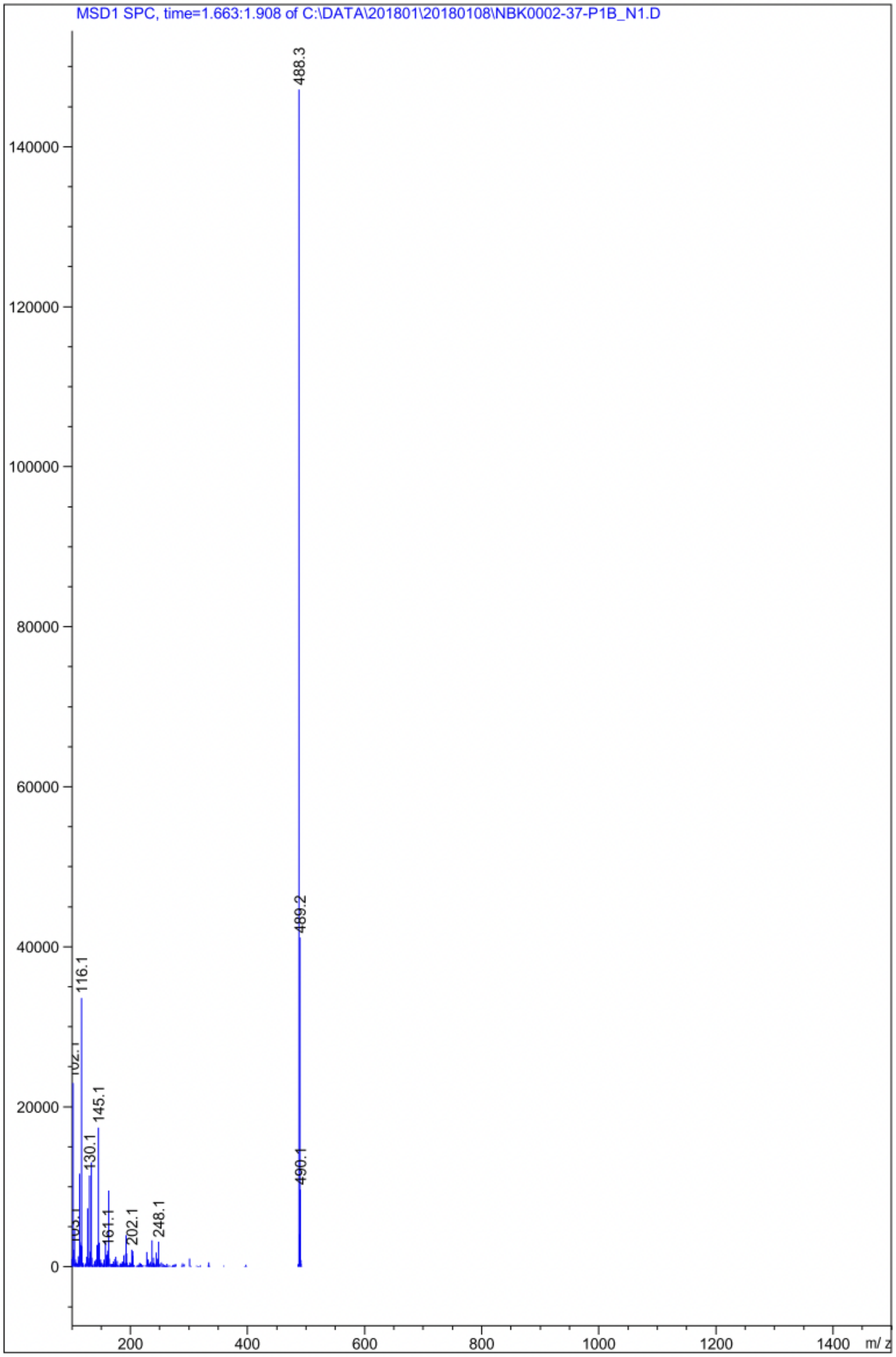
MSD1 SPC, time=1.663:1.908 of C:\DATA\201801\20180108\NBK0002-37-P1B_N1.D
488.3
489.2
490.1
116.1
145.1
130.1
161.1
202.1
248.1
140000
120000
100000
80000
60000
40000
20000
0
200
400
600
800
1000
1200
1400
m/z

Compound 20

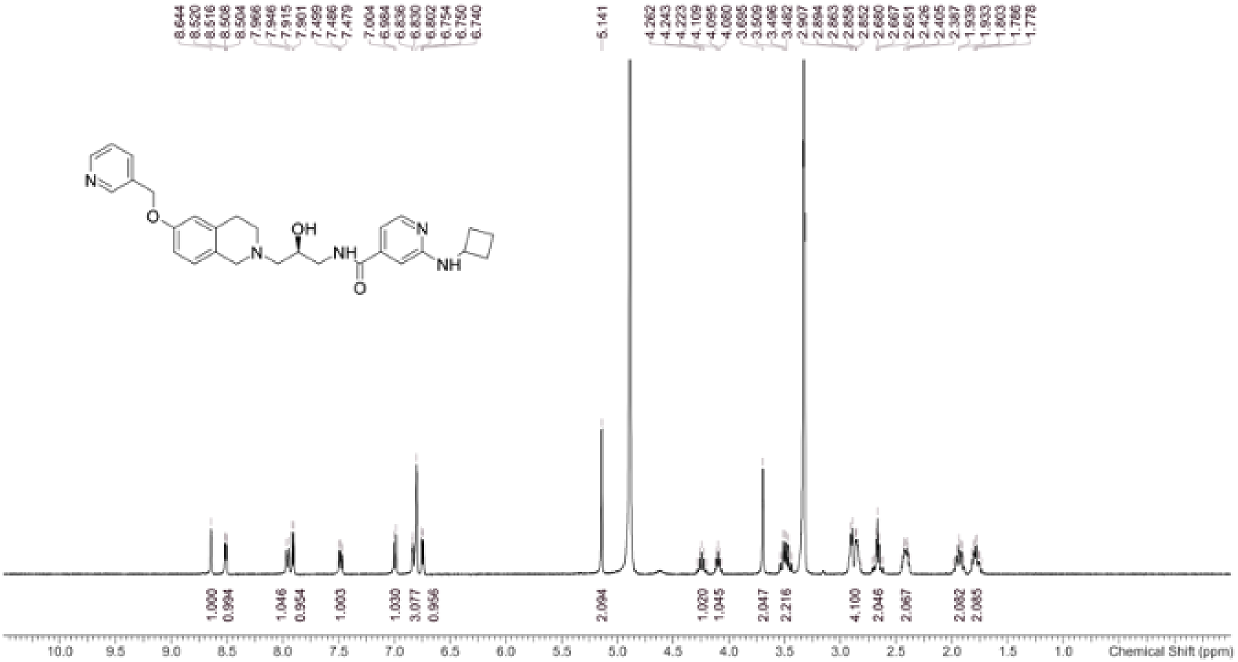

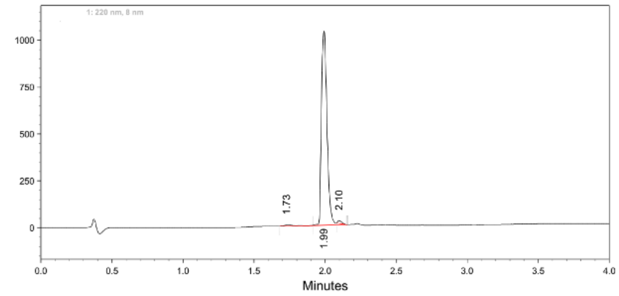

1: 220 nm, 8 nm

Retention Time	*Height*	*Area*	*Area Percent*
1.73	4212	13758	0.48
1.99	1020150	2830163	98.47
2.10	14563	30196	1.05

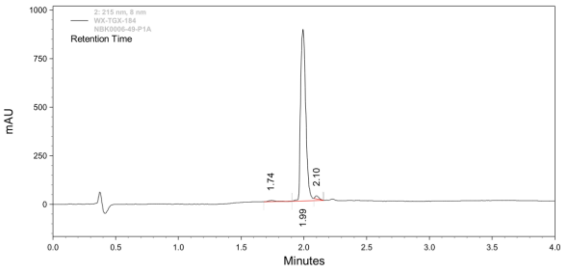

2: 215 nm, 8 nm

Retention Time	*Height*	*Area*	*Area Percent*
1.74	4983	18701	0.72
1.99	871170	2545835	98.03
2.10	15802	32336	1.25

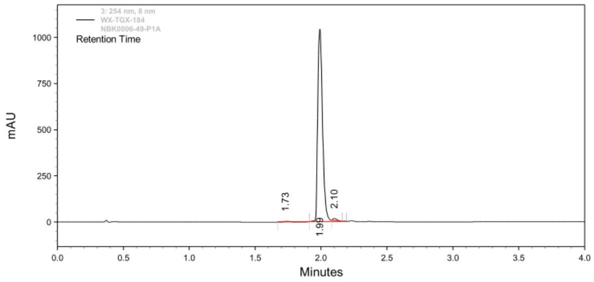

3: 254 nm, 8 nm

Retention Time	*Height*	*Area*	*Area Percent*
1.73	3119	10468	0.39
1.99	1033648	2646984	98.77
2.10	10336	22582	0.84

```
Instrument        : LCMS AR
                    A:,Xtimate,2.1*30mm,3um
                    B:XBridge Shield,  2.1*50mm,5um
                    Confidential. For research only  NOT for regulatory fili
```

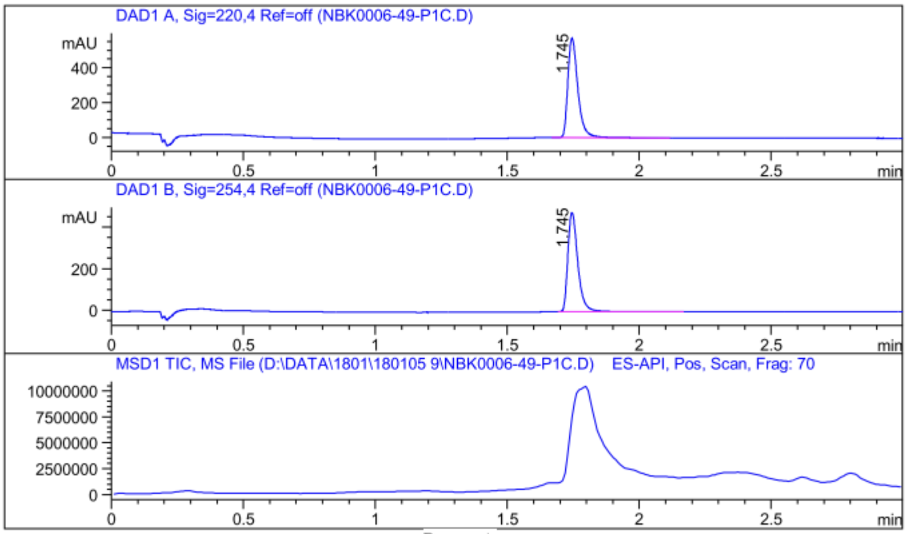

Report

```
===================================================================
   Signal ->: DAD1 A, Sig=220,4 Ref=off
    #  Meas. Ret.   Height    Width        Area        Area %
   ---------------------------------------------------------
     1       1.745     569.276    0.041         1513.587 100.000
   ---------------------------------------------------------


   Signal ->: DAD1 B, Sig=254,4 Ref=off
    #  Meas. Ret.   Height    Width        Area        Area %
   ---------------------------------------------------------
     1       1.745     477.184    0.041         1263.405 100.000
   ---------------------------------------------------------
```

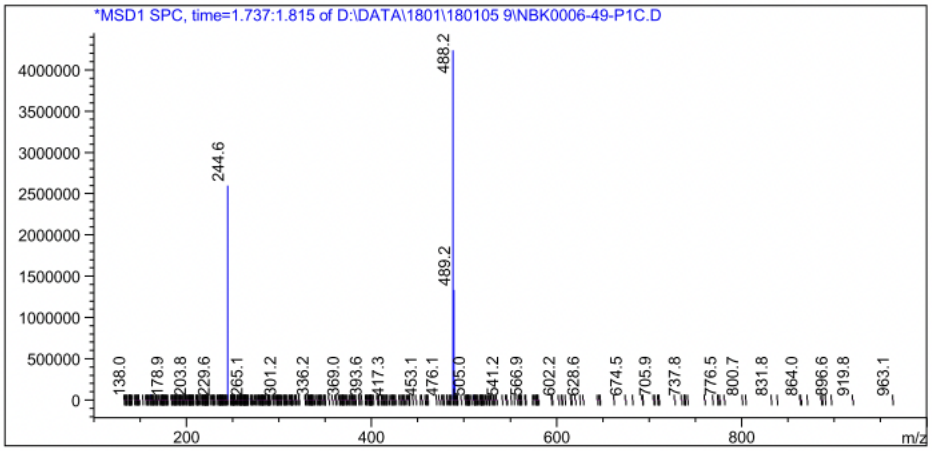

Method : Column: Chiralpak AD-3 100×4.6mm I.D., 3um
Mobile phase: 40% of iso-propanol (0.05% DEA) in CO2
Flow rate: 2.8mL/min Column temperature:40 C

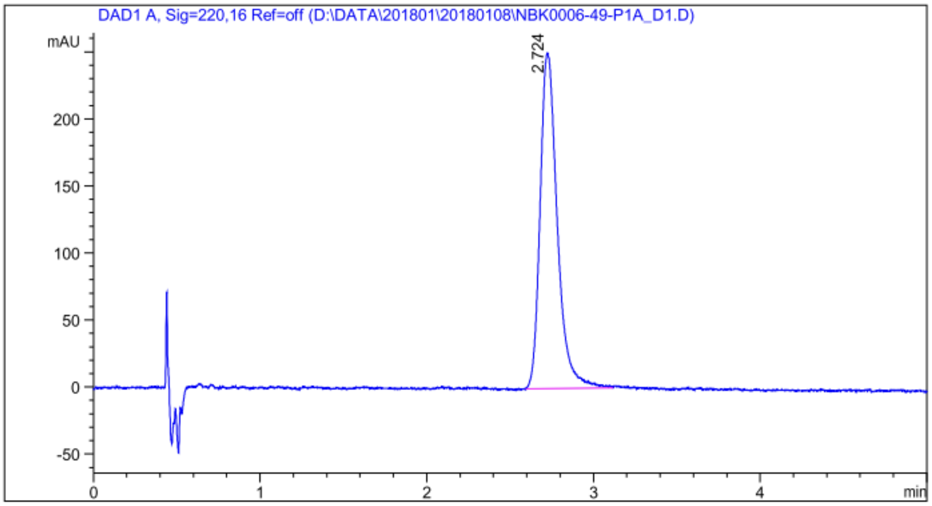

DAD1 A, Sig=220,16 Ref=off

#	Meas. Ret. Time	Height	Height %	Width	Area	Area %
1	2.724	250.988	100.000	0.119	1796.851	100.000

Compound 21

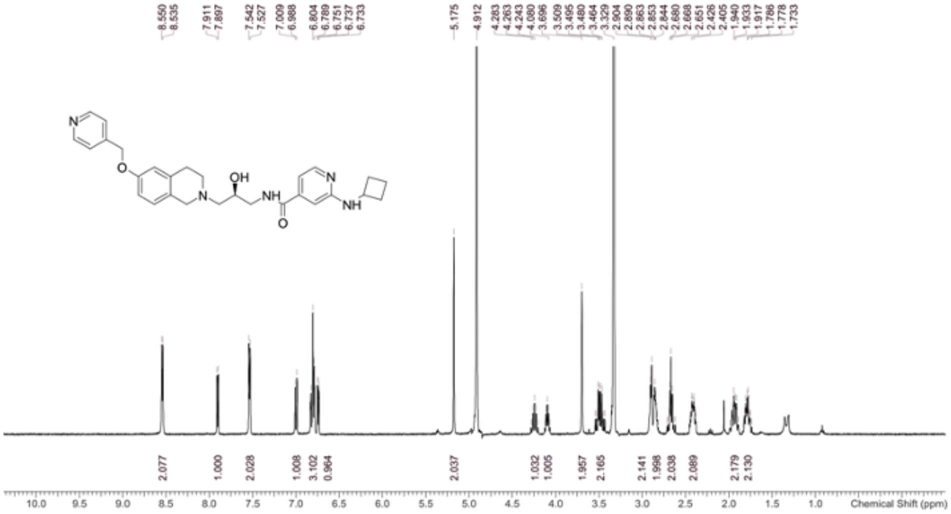

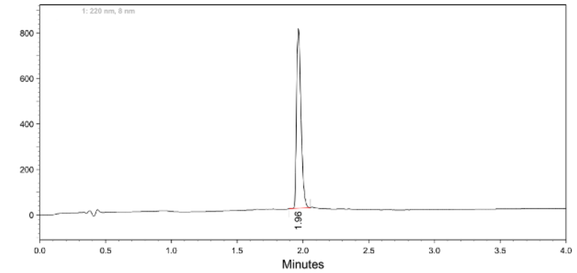

1: 220 nm, 8 nm

Retention Time	*Height*	*Area*	*Area Percent*
1.96	783770	1861168	100.00

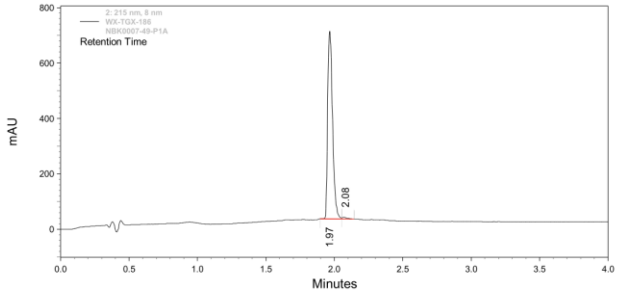

2: 215 nm, 8 nm

Retention Time	*Height*	*Area*	*Area Percent*
1.97	672821	1677936	99.08
2.08	5521	15614	0.92

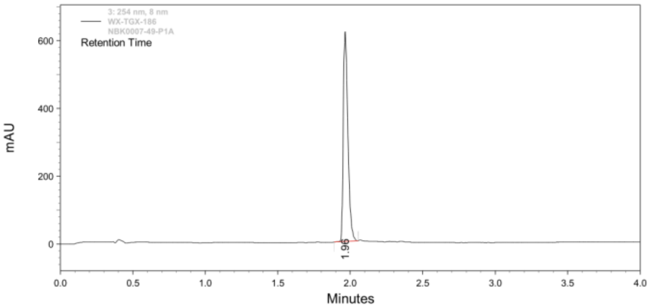

3: 254 nm, 8 nm

Retention Time	*Height*	*Area*	*Area Percent*
1.96	612928	1371847	100.00

Instrument : LCMS AR

A:,Xtimate,2.1*30mm,3um

B:XBridge Shield, 2.1*50mm,5um

Confidential. For research only NOT for regulatory filir

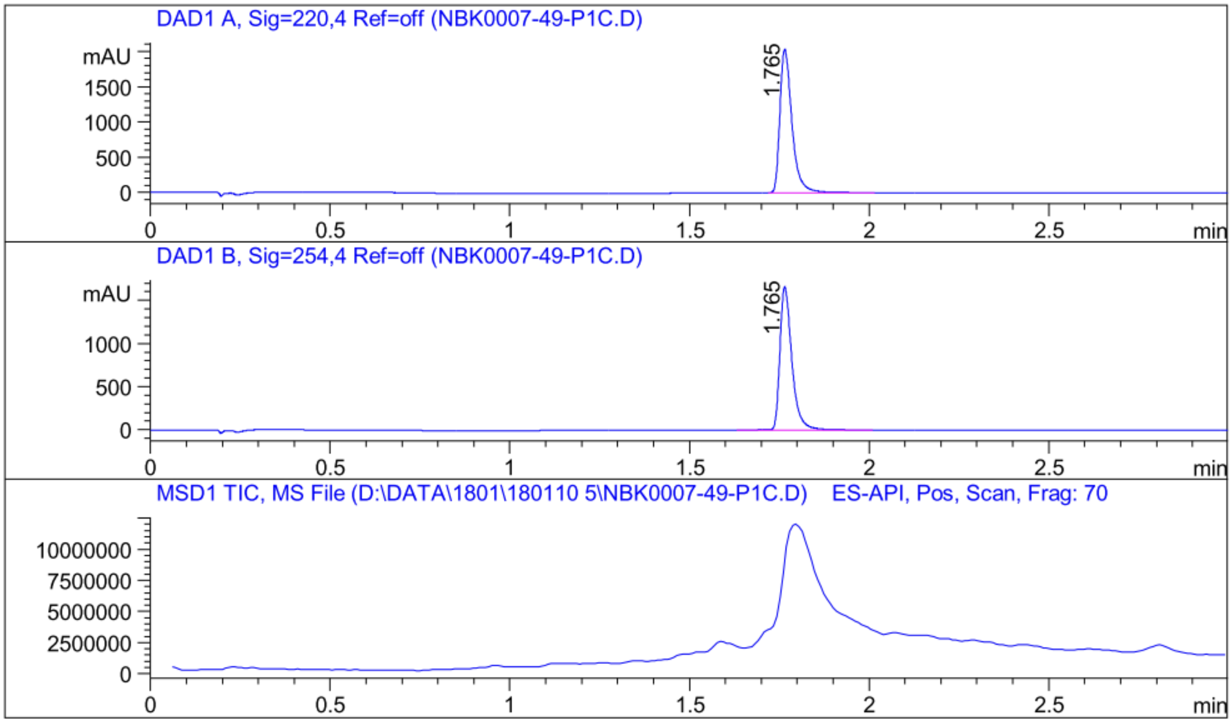

Report

==

Signal ->: DAD1 A, Sig=220,4 Ref=off

#	Meas. Ret.	Height	Width	Area	Area %
1	1.765	2027.346	0.035	4753.665	100.000

Signal ->: DAD1 B, Sig=254,4 Ref=off

#	Meas. Ret.	Height	Width	Area	Area %
1	1.765	1658.967	0.035	3869.469	100.000

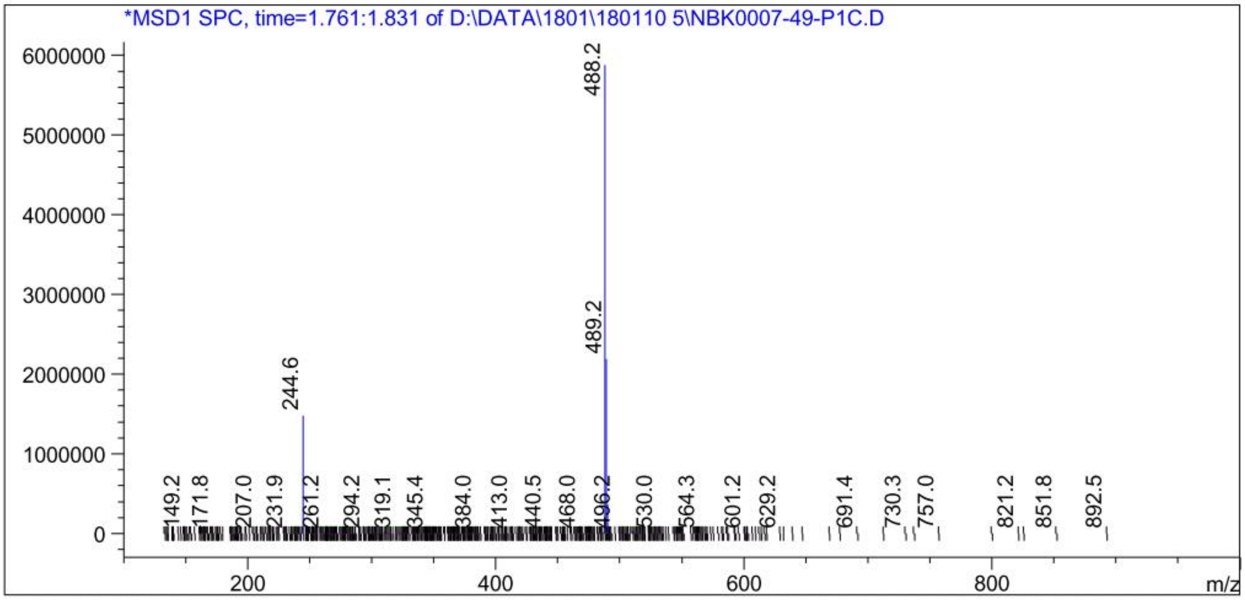


```
Method              :Column: Chiralpak AD-3 50*4.6mm I.D., 3um
                     Mobile phase: 40% of ethanol(0.05% DEA) in CO2
                     Flow rate: 4mL/min
                     Column temp:40 C
```

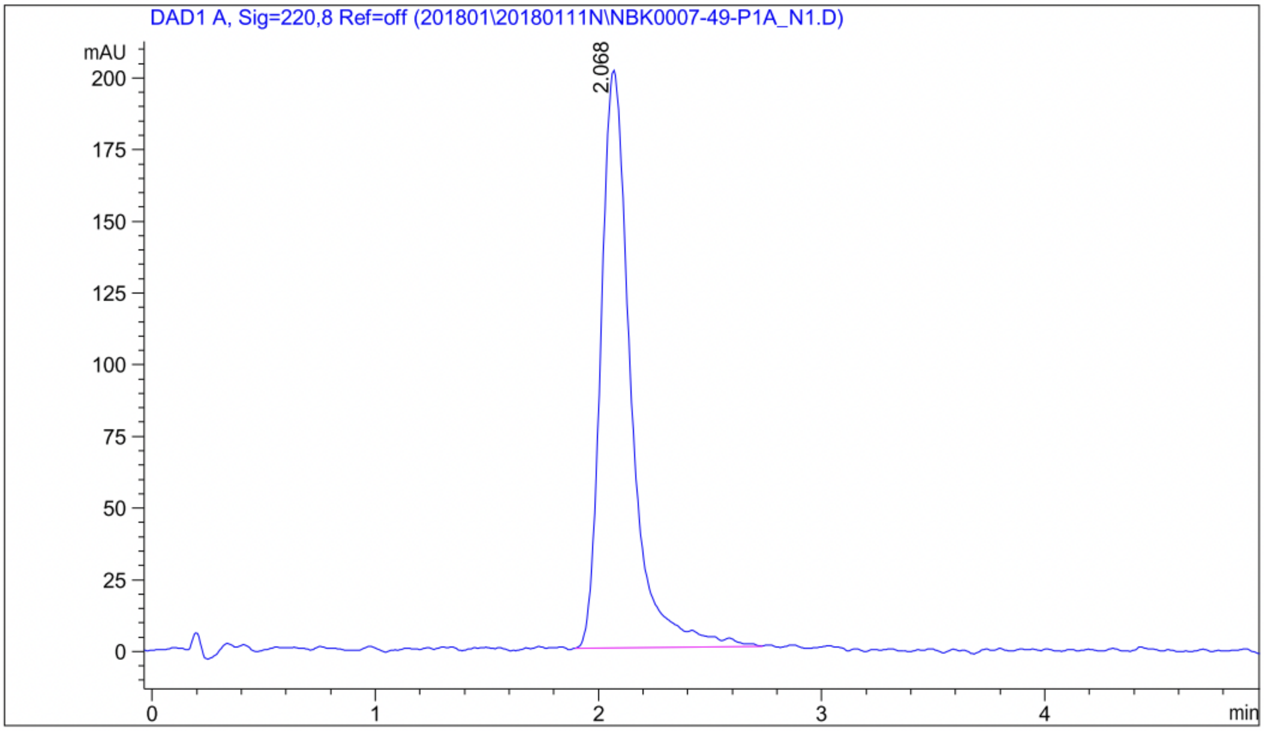


```
=================================================================================
Signal 1 :     DAD1 A, Sig=220,8 Ref=off
  Peak  Meas. Ret. Time   Height    Height %     Width      Area      Area %
---------------------------------------------------------------------------------
      1            2.068    201.517    100.000      0.156   1880.388    100.000
------- -------------- ---------- ---------- ---------- ---------- ----------
```

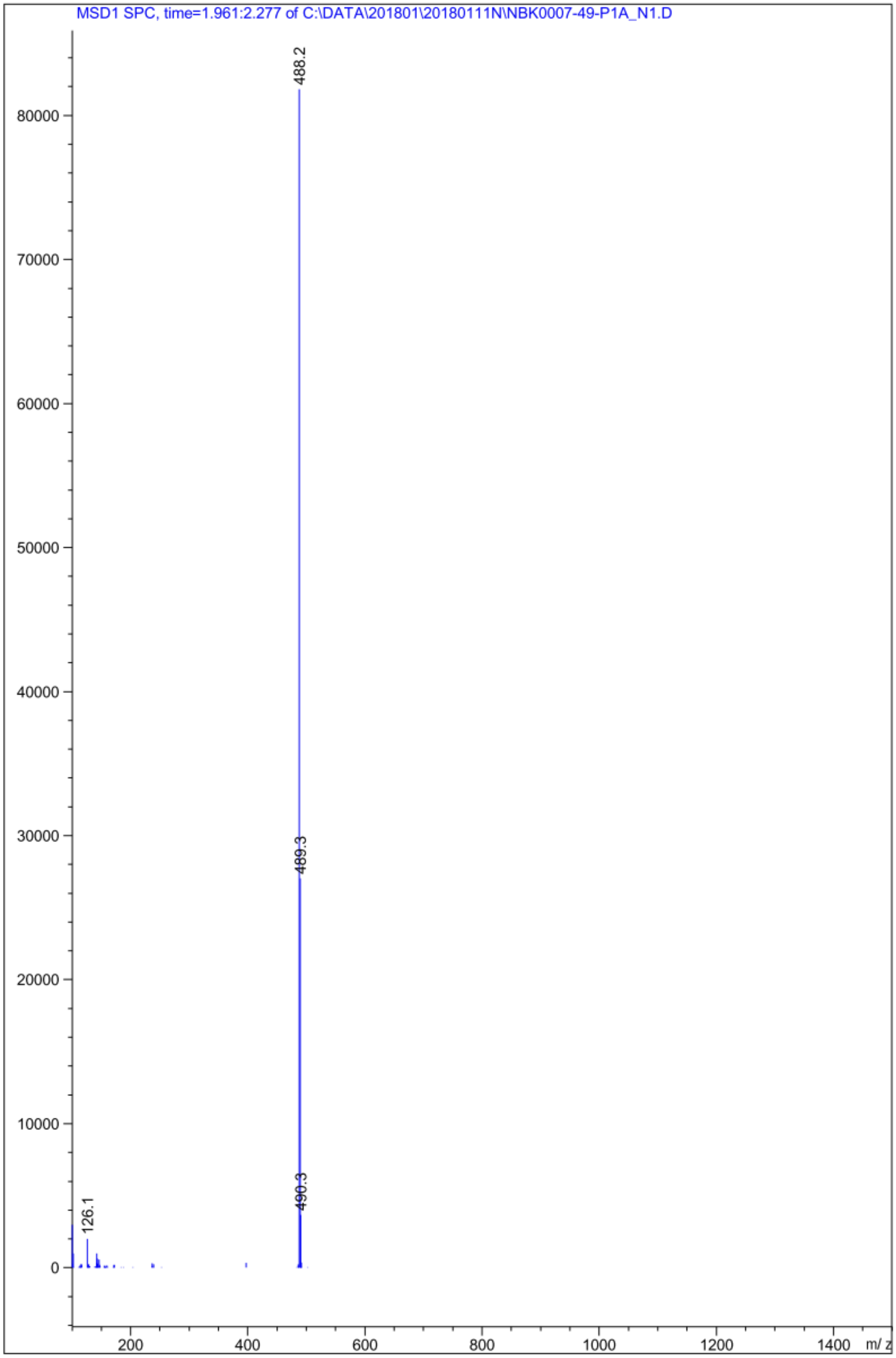

MSD1 SPC, time=1.961:2.277 of C:\DATA\201801\20180111N\NBK0007-49-P1A_N1.D
488.2
489.3
490.3
126.1
80000
70000
60000
50000
40000
30000
20000
10000
0
200
400
600
800
1000
1200
1400
m/z

Compound 22

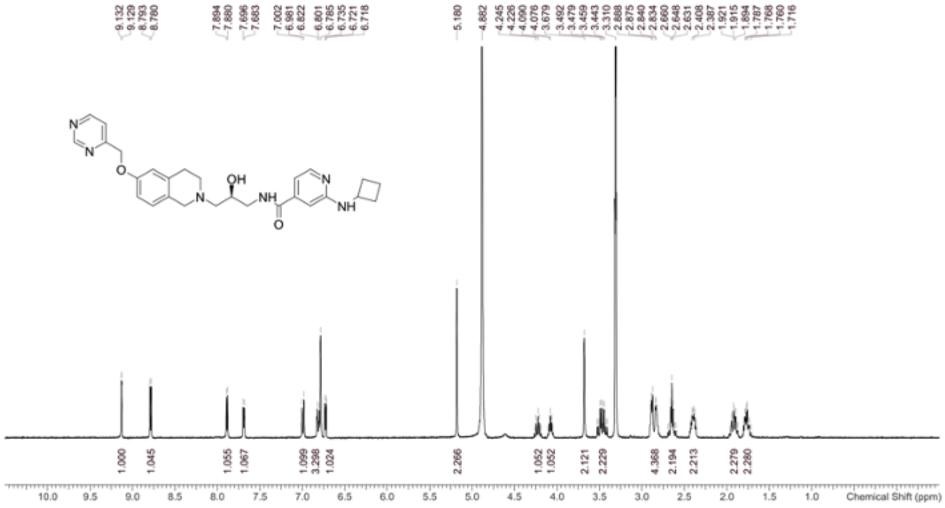

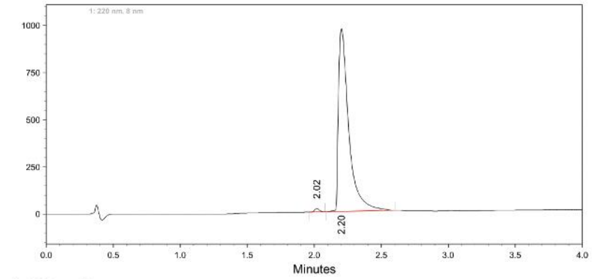

1: 220 nm, 8 nm

Retention Time	*Height*	*Area*	*Area Percent*
2.02	17778	45677	0.88
2.20	965508	5164819	99.12

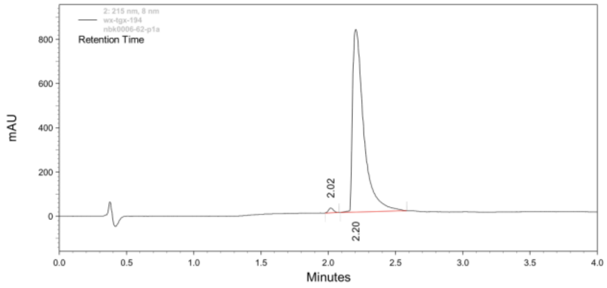

2: 215 nm, 8 nm

Retention Time	*Height*	*Area*	*Area Percent*
2.02	21171	54865	1.13
2.20	826110	4795727	98.87

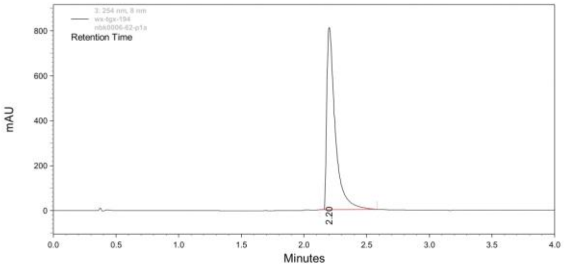

3: 254 nm, 8 nm

Retention Time	*Height*	*Area*	*Area Percent*
2.20	799737	3808378	100.00

```
Instrument         : LCMS AR
                     A:,Xtimate,2.1*30mm,3um
                     B:XBridge Shield,  2.1*50mm,5um
                     Confidential. For research only  NOT for regulatory filin
```

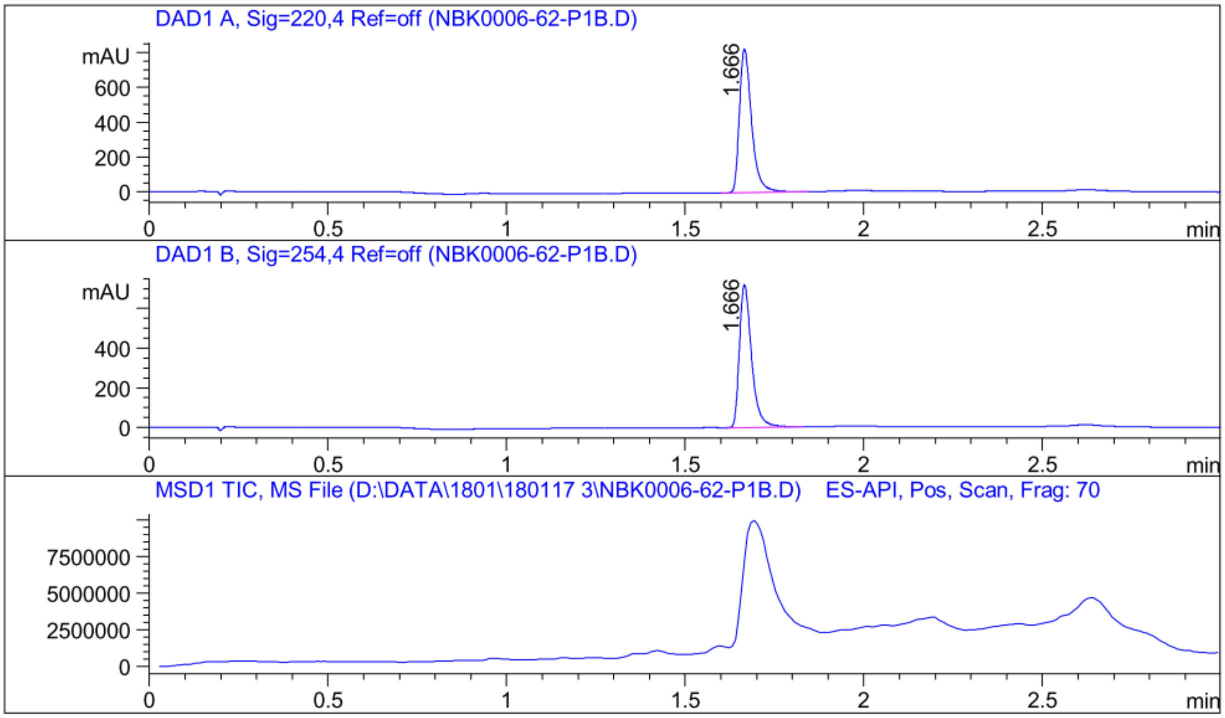

Report

Signal ->: DAD1 A, Sig=220,4 Ref=off

#	Meas. Ret.	Height	Width	Area	Area %
1	1.666	822.429	0.036	1966.456	100.000

Signal ->: DAD1 B, Sig=254,4 Ref=off

#	Meas. Ret.	Height	Width	Area	Area %
1	1.666	716.872	0.036	1710.133	100.000

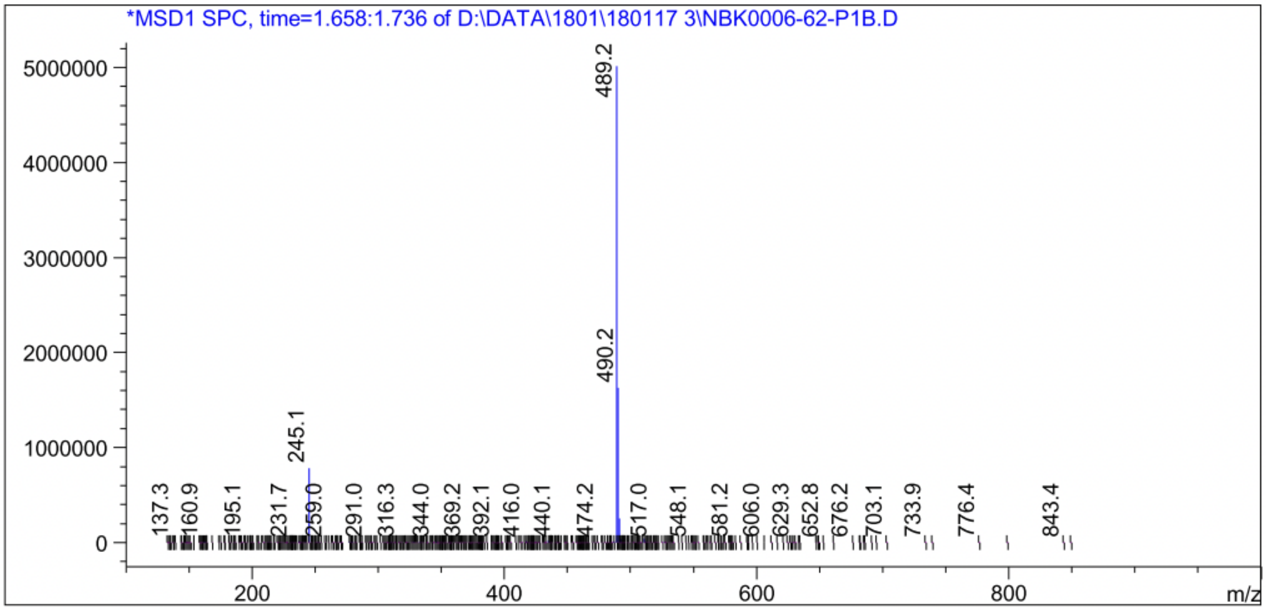

*MSD1 SPC, time=1.658:1.736 of D:\DATA\1801\180117 3\NBK0006-62-P1B.D
5000000
4000000
3000000
2000000
1000000
0
489.2
490.2
245.1
137.3
160.9
195.1
231.7
259.0
291.0
316.3
344.0
369.2
392.1
416.0
440.1
474.2
517.0
548.1
581.2
606.0
629.3
652.8
676.2
703.1
733.9
776.4
843.4
200
400
600
800
m/z

Method :Column: Chiralpak AD-3 50*4.6mm I.D., 3um
Mobile phase: 40% of ethanol(0.05% DEA) in CO2
Flow rate: 4mL/min
Column temp:40 C

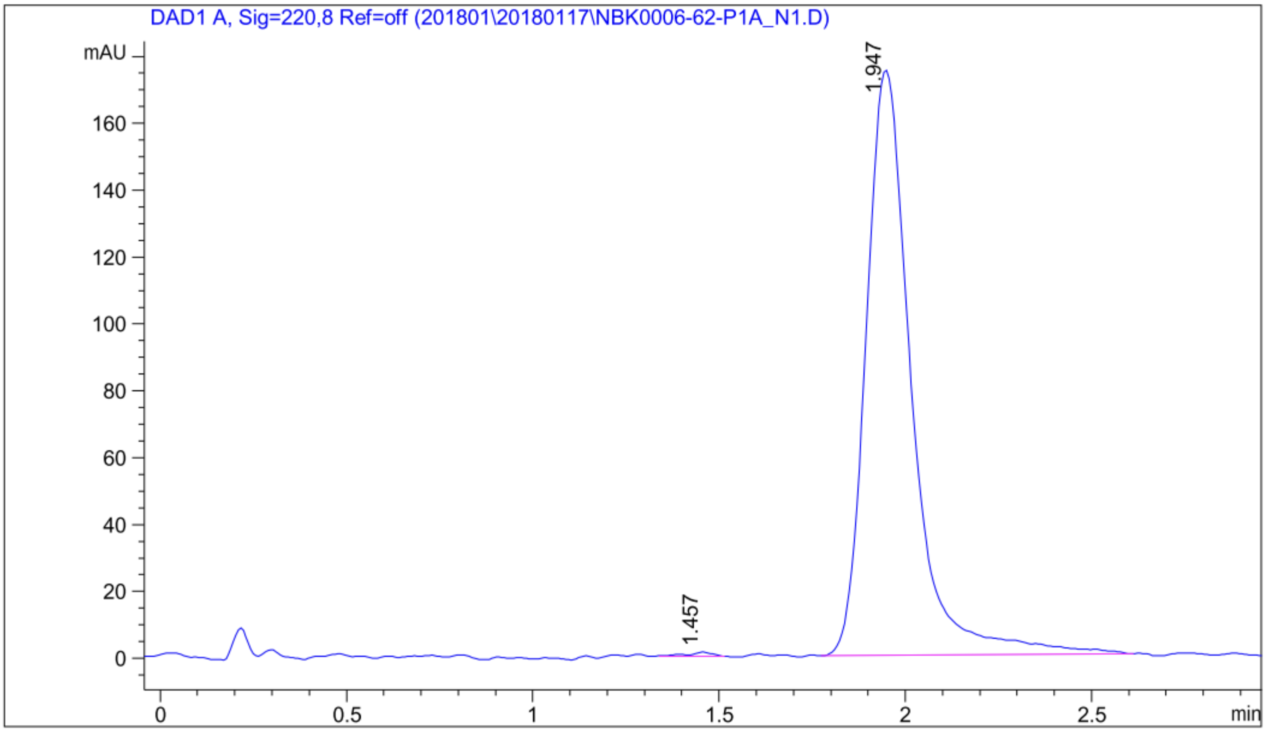

Signal 1 : DAD1 A, Sig=220,8 Ref=off

Peak	Meas. Ret. Time	Height	Height %	Width	Area	Area %
1	1.457	1.266	0.718	0.079	5.990	0.400
2	1.947	175.168	99.282	0.142	1490.041	99.600

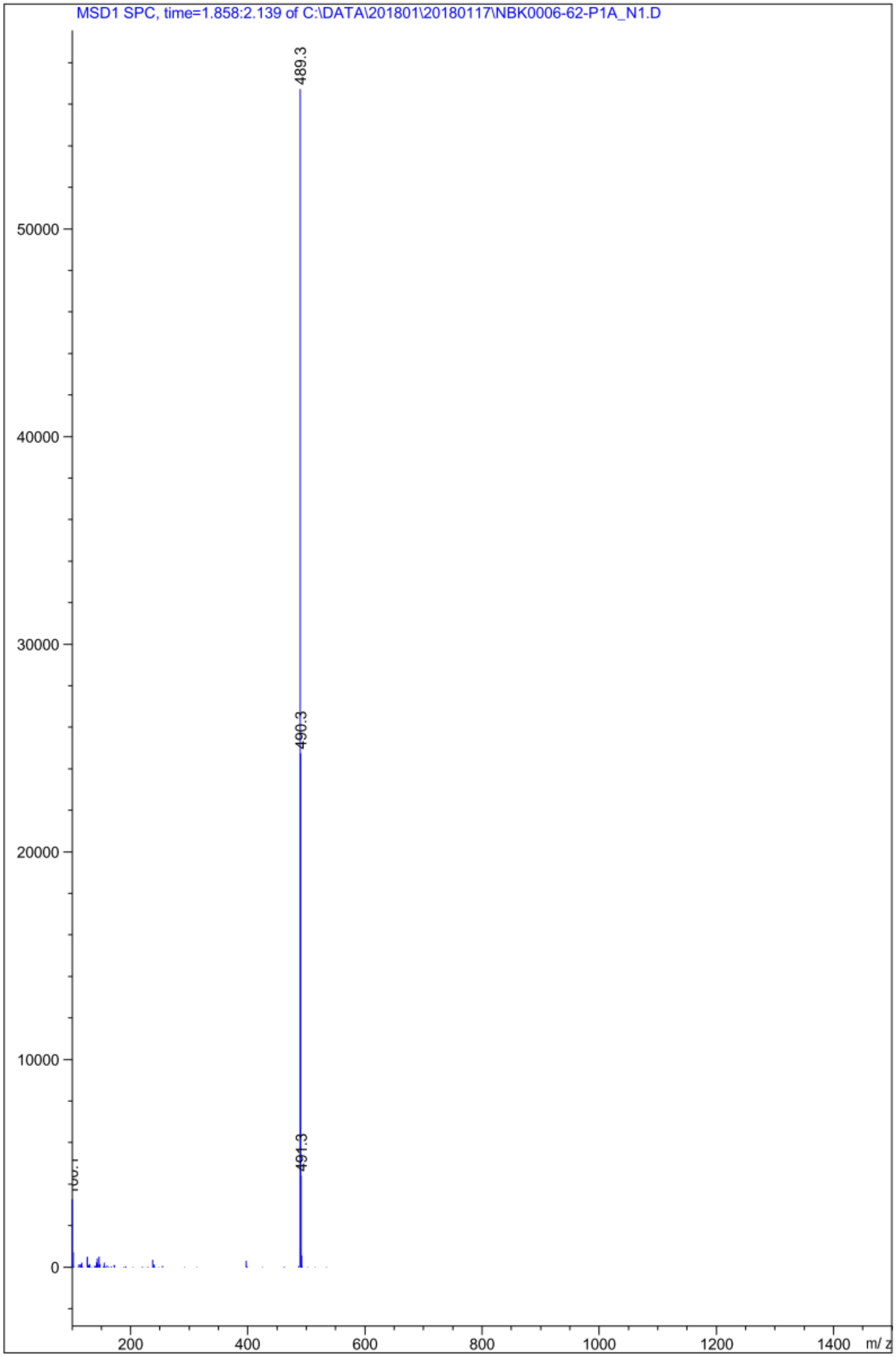
MSD1 SPC, time=1.858:2.139 of C:\DATA\201801\20180117\NBK0006-62-P1A_N1.D
489.3
490.3
491.3
50000
40000
30000
20000
10000
0
200
400
600
800
1000
1200
1400
m/z

Compound 23

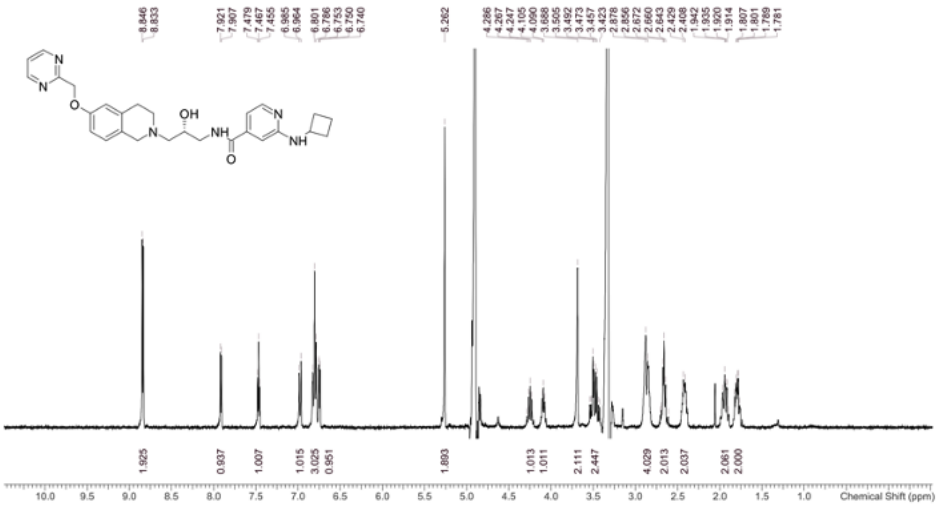

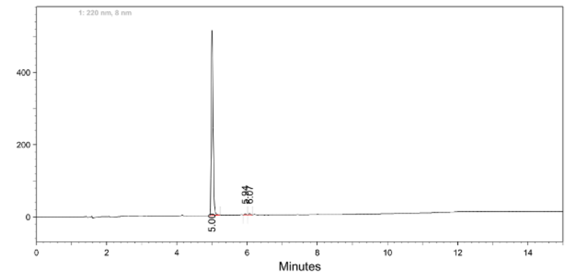

1: 220 nm, 8 nm

Retention Time	Height	Area	Area Percent
5.00	513387	1780731	98.76
5.94	2923	10089	0.56
6.07	3645	12200	0.68

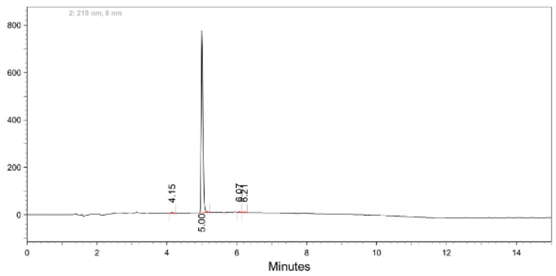

2: 210 nm, 8 nm

Retention Time	Height	Area	Area Percent
4.15	3232	10863	0.40
5.00	770216	2666652	98.70
6.07	3750	12511	0.46
6.21	3465	11654	0.43

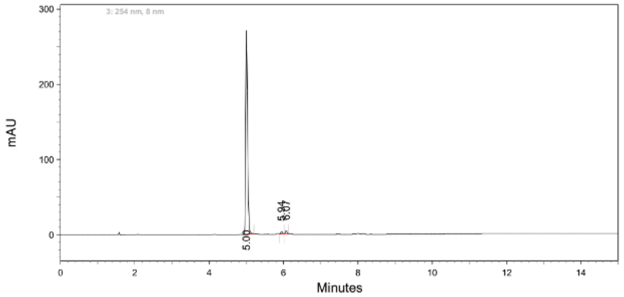

3: 254 nm, 8 nm

Retention Time	Height	Area	Area Percent
5.00	271147	944689	97.47
5.94	2980	10271	1.06
6.07	4246	14221	1.47

```
Instrument          : LCMS AR
                      A:,Xtimate,2.1*30mm,3um
                      B:XBridge Shield,  2.1*50mm,5um
                      Confidential. For research only  NOT for regulatory filin
```

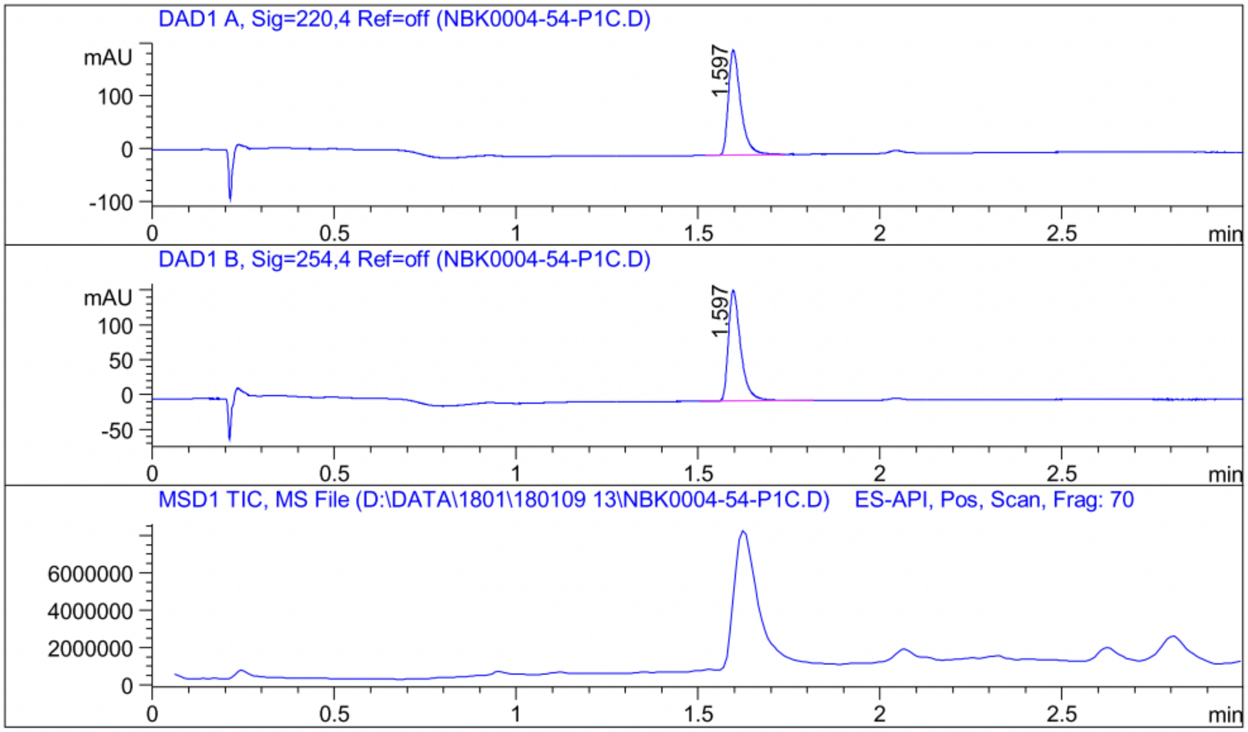

Report

==

Signal ->: DAD1 A, Sig=220,4 Ref=off

#	Meas. Ret.	Height	Width	Area	Area %
1	1.597	197.969	0.036	470.798	100.000

Signal ->: DAD1 B, Sig=254,4 Ref=off

#	Meas. Ret.	Height	Width	Area	Area %
1	1.597	158.009	0.036	377.688	100.000

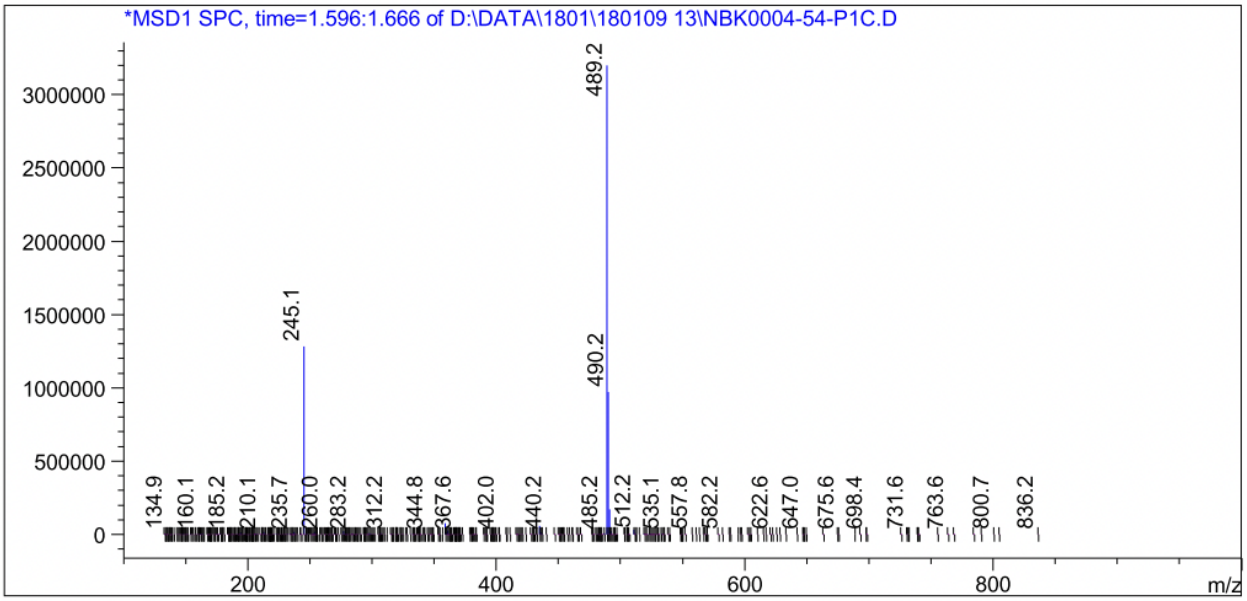


```
Method              : Column: Chiralcel OJ-3 100×4.6mm I.D., 3um
                      Mobile phase: A: CO2  B:Methanol (0.1%ethanolamine)
                      Gradient: from 5% to 40% of B  in 4.5min and hold 40%
                      for 2.5 min,  then 5% of B for  1 min
                      Flow rate: 2.8mL/min    Column temperature:40 C
=====================================================================
```

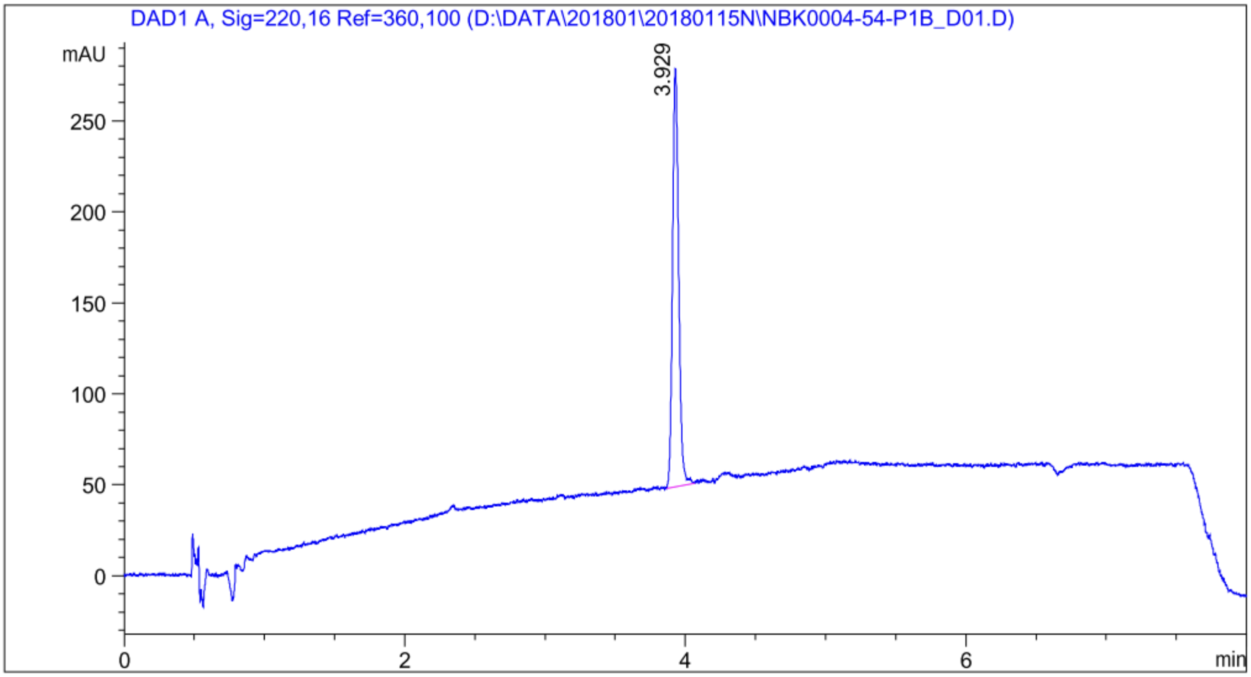

===

DAD1 A, Sig=220,16 Ref=360,100

#	Meas. Ret. Time	Height	Height %	Width	Area	Area %
1	3.929	229.760	100.000	0.051	697.439	100.000

Compound 24

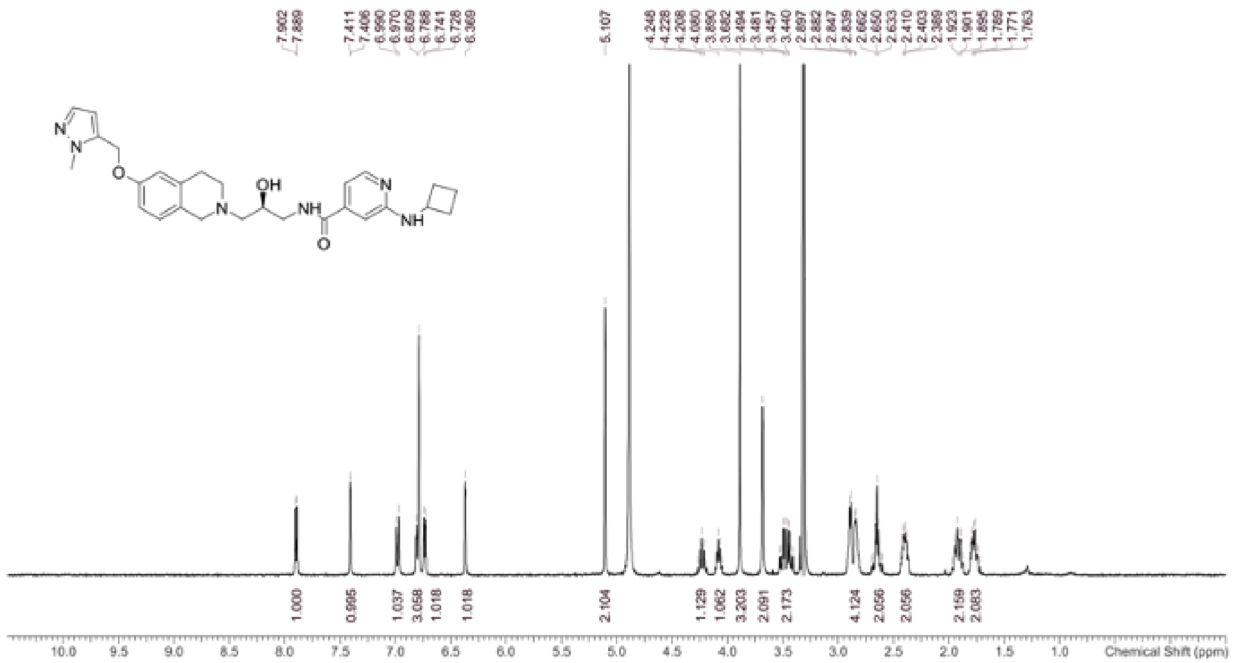

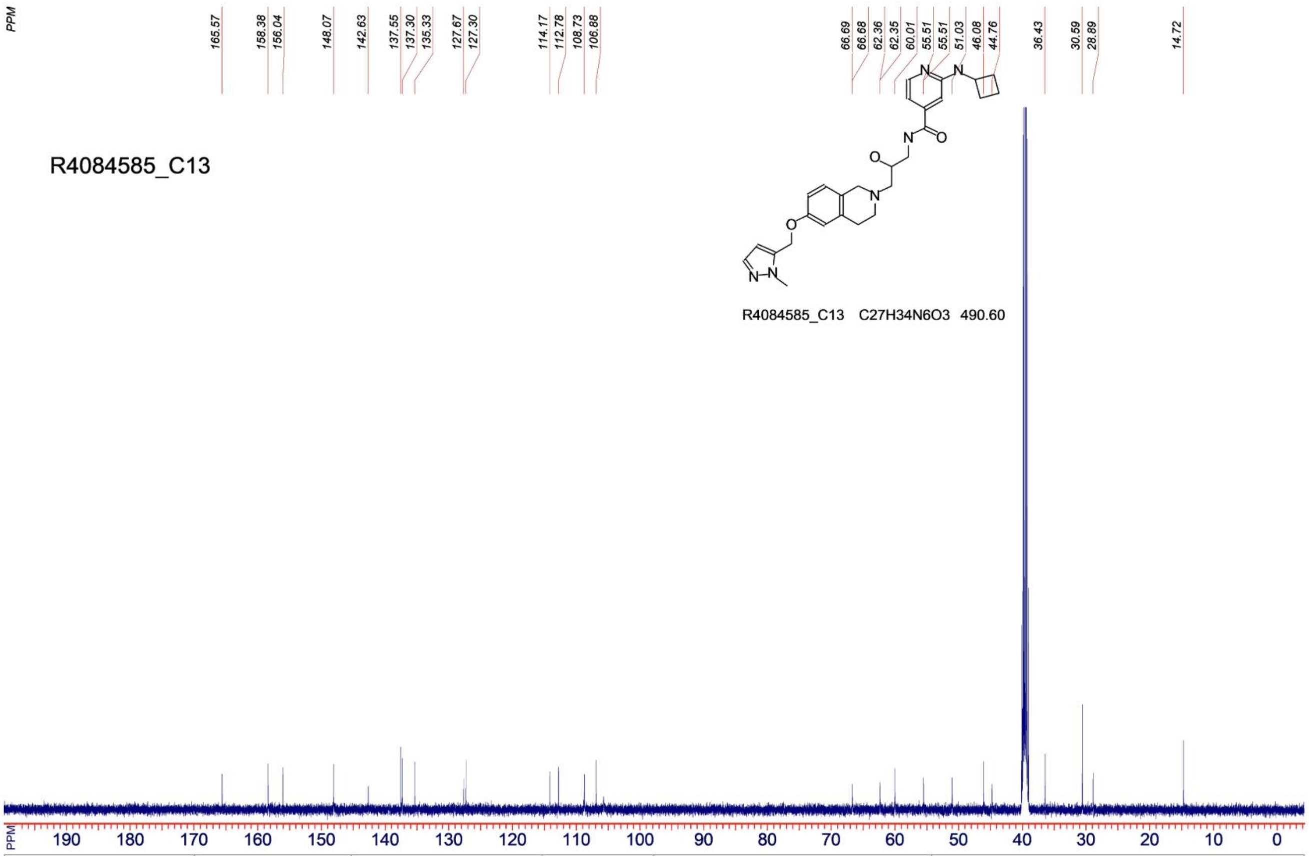

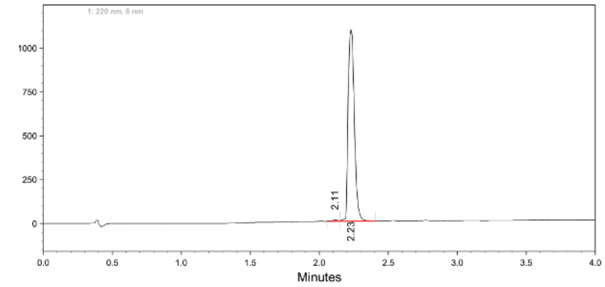

1: 220 nm, 8 nm

Retention Time	*Height*	*Area*	*Area Percent*
2.11	7504	22648	0.66
2.23	1089154	3430256	99.34

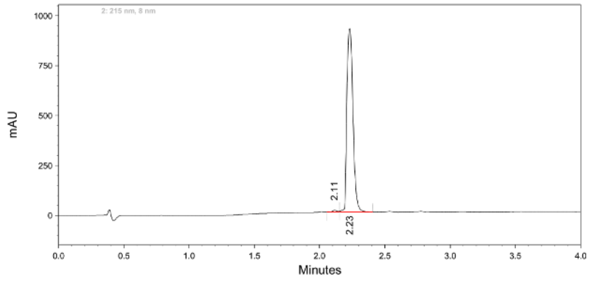

2: 215 nm, 8 nm

Retention Time	*Height*	*Area*	*Area Percent*
2.11	7546	21668	0.72
2.23	915450	2977504	99.28

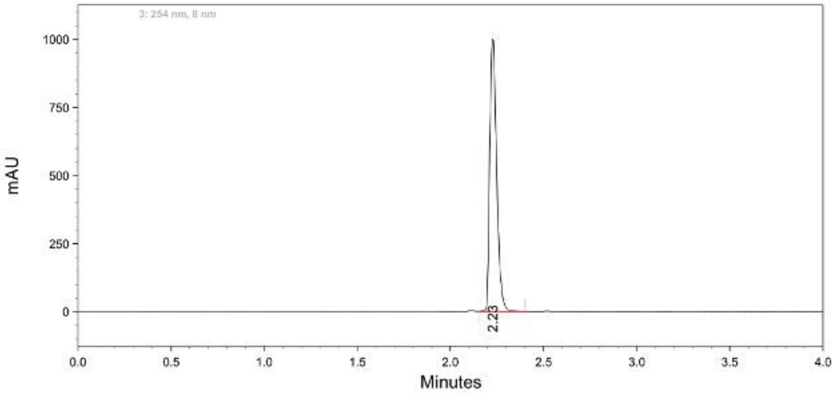

3: 254 nm, 8 nm

Retention Time	*Height*	*Area*	*Area Percent*
2.23	987158	2596706	100.00

A:,Xtimate,2.1*30mm,3um
B:XBridge Shield, 2.1*50mm,5um
Confidential. For research only NOT for regulatory fili

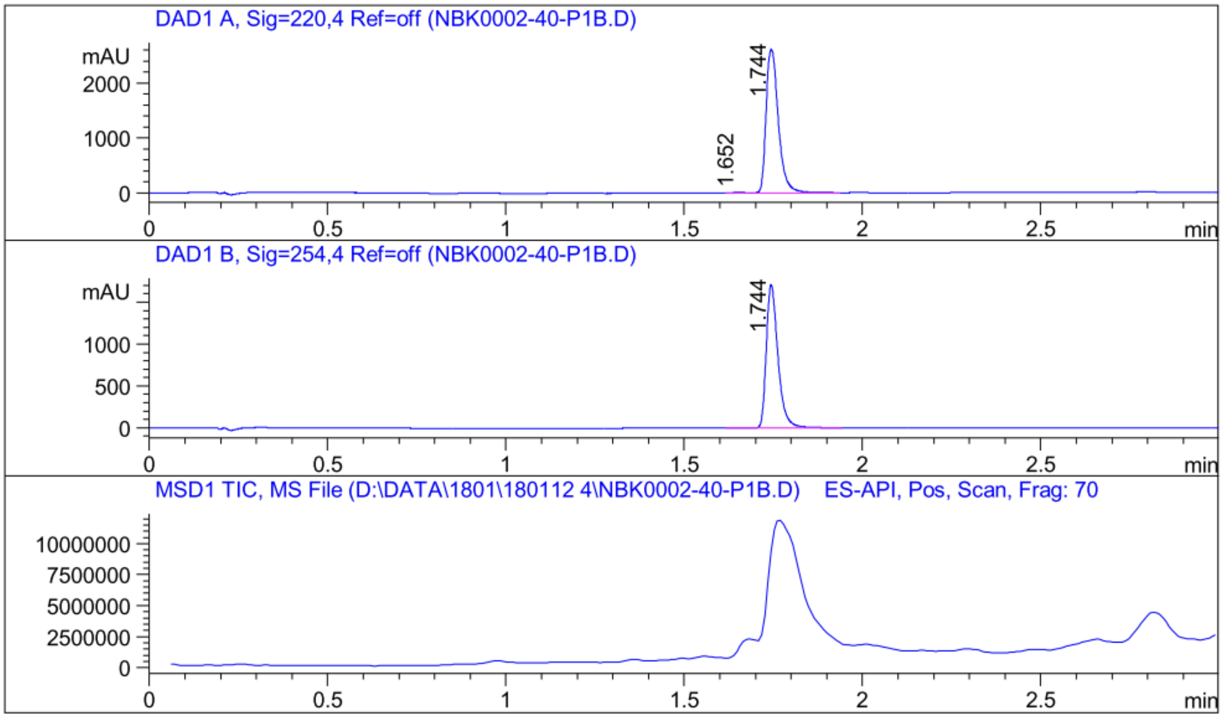

Report

===

Signal ->: DAD1 A, Sig=220,4 Ref=off

#	Meas. Ret.	Height	Width	Area	Area %
1	1.652	11.966	0.036	28.113	0.446
2	1.744	2607.486	0.037	6271.941	99.554

Signal ->: DAD1 B, Sig=254,4 Ref=off

#	Meas. Ret.	Height	Width	Area	Area %
1	1.744	1701.812	0.036	3904.715	100.000

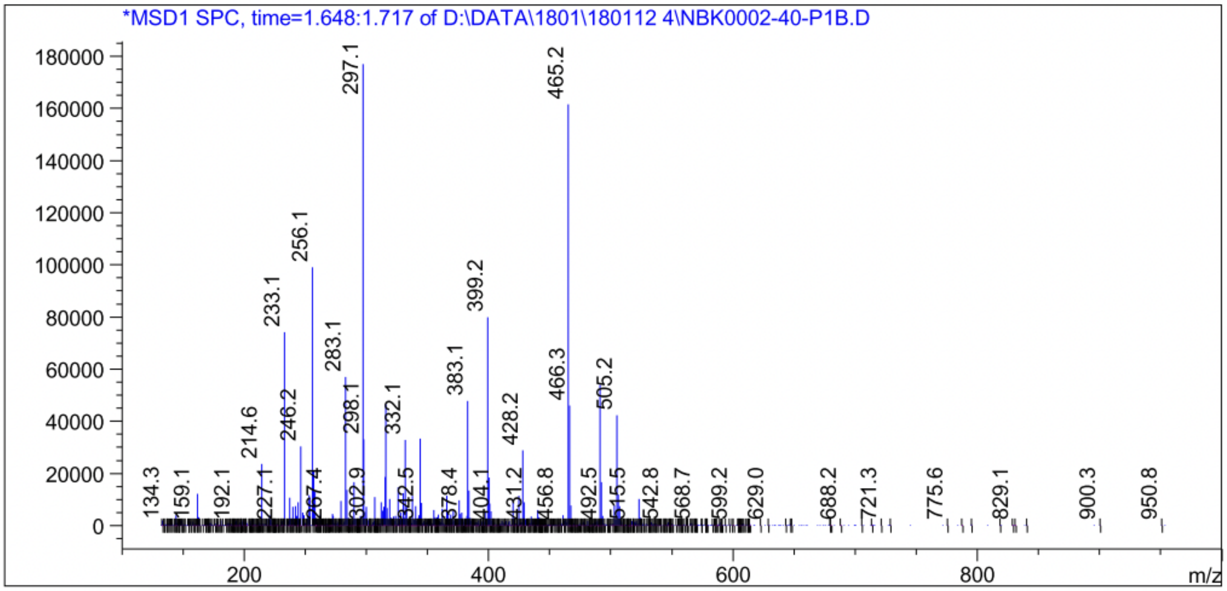
*MSD1 SPC, time=1.648:1.717 of D:\DATA\1801\180112 4\NBK0002-40-P1B.D
297.1
465.2
256.1
399.2
233.1
283.1
383.1
466.3
505.2
428.2
m/z

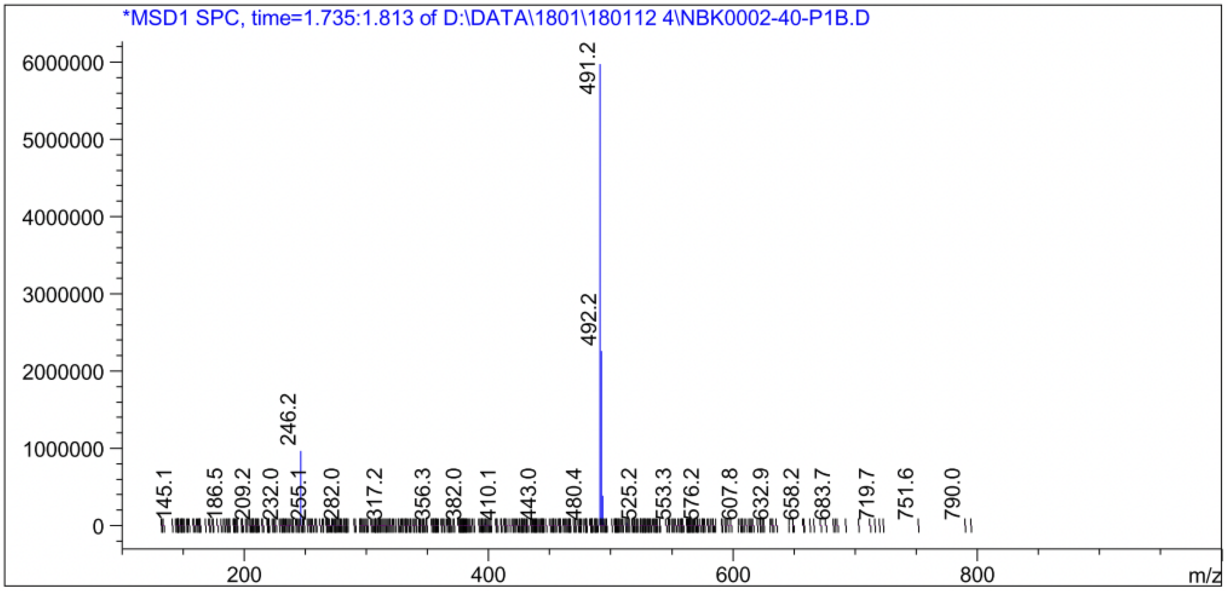
*MSD1 SPC, time=1.735:1.813 of D:\DATA\1801\180112 4\NBK0002-40-P1B.D
491.2
492.2
246.2
m/z

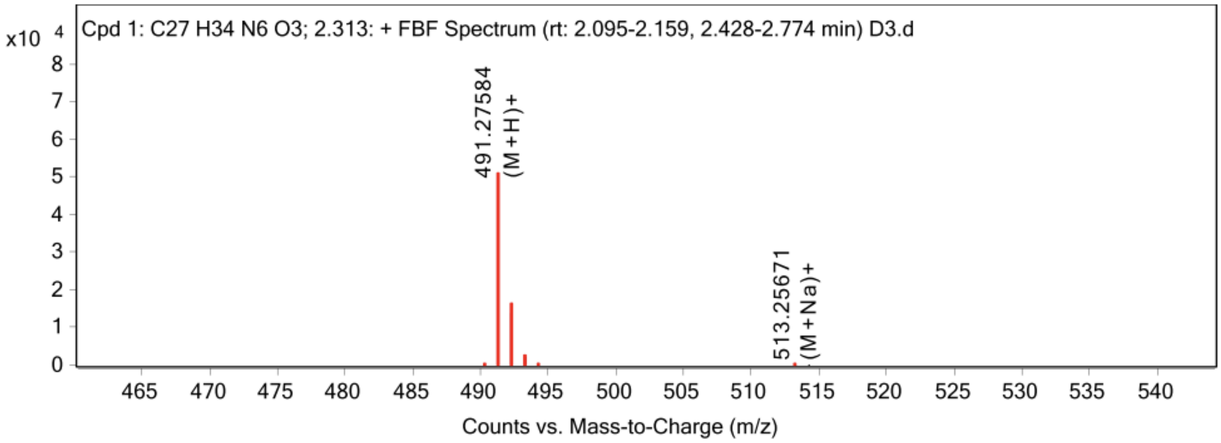

MS Spectrum Peak List

Obs. *m/z*	Charge	Abund	Ion/Isotope
490.26386	1	369.57	M+
491.27584	1	51171.16	(M+H)+
492.27864	1	13655.8	(M+H)+
493.28148	1	2128.12	(M+H)+
494.28302	1	261.42	(M+H)+
513.25671	1	210.98	(M+Na)+
514.26371	1	94.45	(M+Na)+

MS Zoomed Spectrum

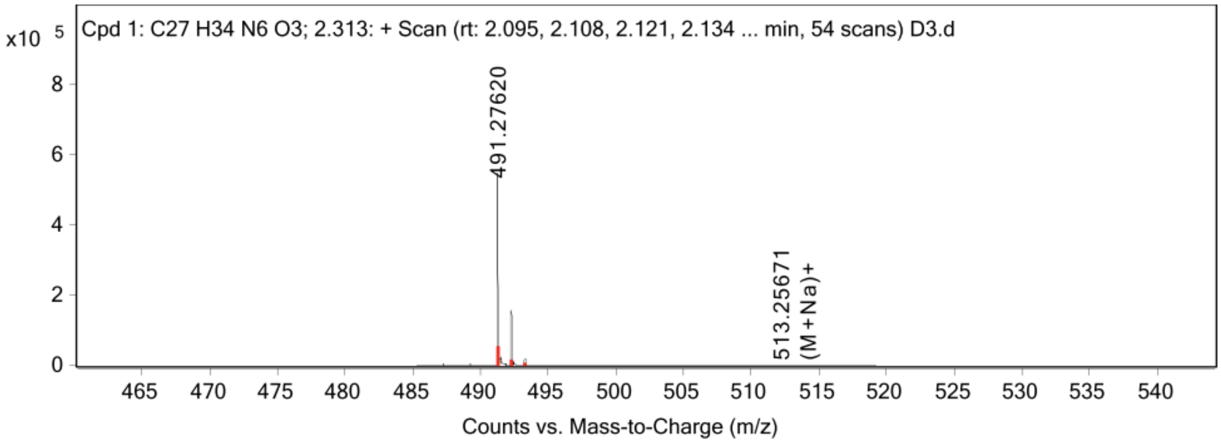

MS Spectrum Peak List

Obs. *m/z*	Charge	Abund	Ion/Isotope	Tgt Mass Error (ppm)
490.26386	1	369.57	M+	9.86
491.27584	1	51171.16	(M+H)+	1.38
491.2762		553875.9		
492.27864	1	13655.8	(M+H)+	1.7
493.28148	1	2128.12	(M+H)+	1.54
494.28302	1	261.42	(M+H)+	3.78
513.25671	1	210.98	(M+Na)+	3.42
514.26371	1	94.45	(M+Na)+	-4.45

--- End Of Report ---

```
Method                  : Column: Chiralcel OJ-3 100×4.6mm I.D., 3um
                          Mobile phase: A: CO2  B:ethanol (0.05% DEA)
                          Gradient: from 5% to 40% of B  in 4.5min and hold 40%
                          for 2.5 min,  then 5% of B for   1 min
                          Flow rate: 2.8mL/min    Column temperature:40 C
===============================================================================
```

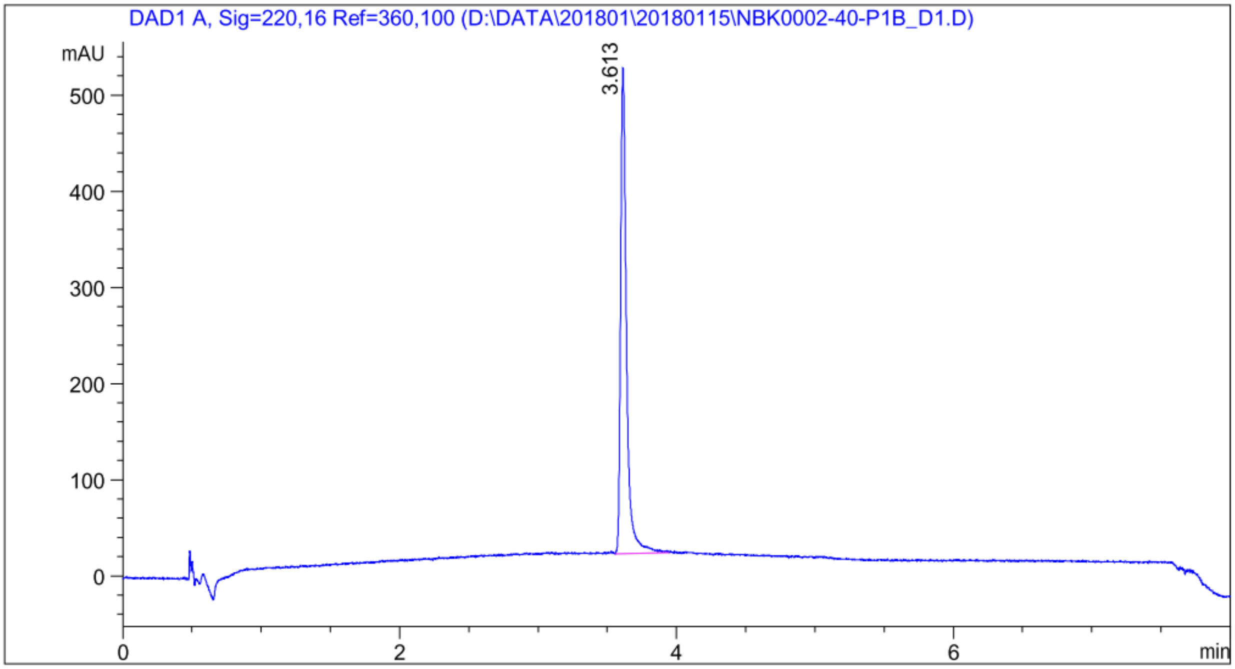


```
===============================================================================
DAD1 A, Sig=220,16 Ref=360,100
```

#	Meas. Ret. Time	Height	Height %	Width	Area	Area %
1	3.613	505.926	100.000	0.049	1500.462	100.000

Compound 25

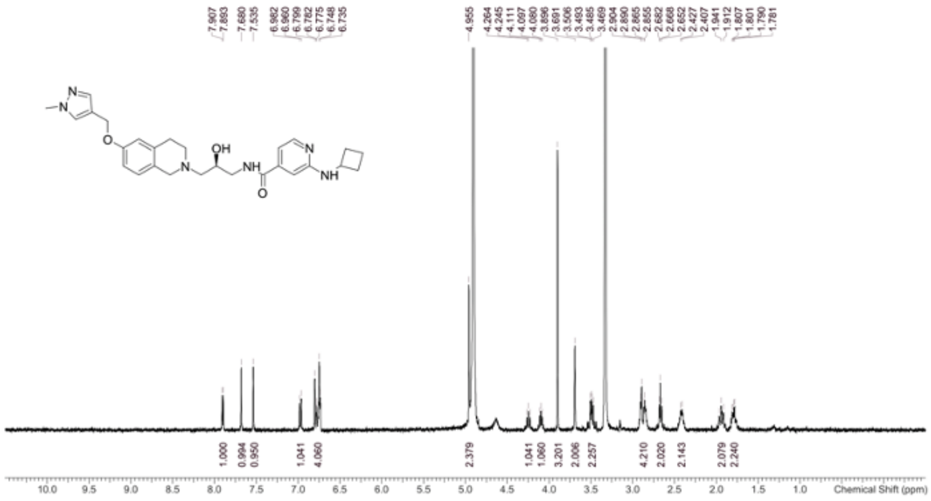

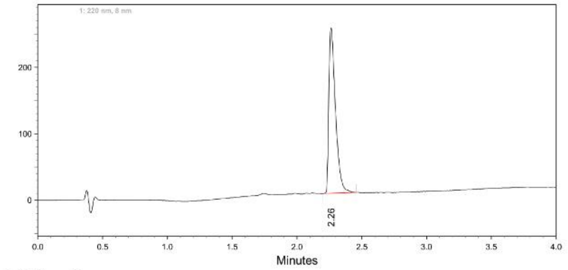

1: 220 nm, 8 nm

Retention Time	Height	Area	Area Percent
2.26	246881	892409	100.00

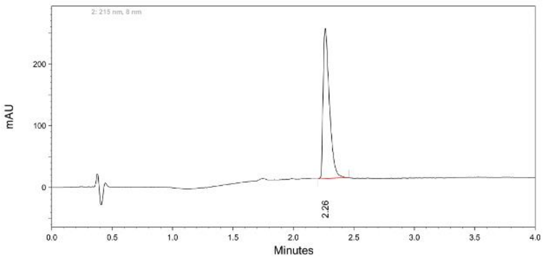

2: 215 nm, 8 nm

Retention Time	Height	Area	Area Percent
2.26	241411	880097	100.00

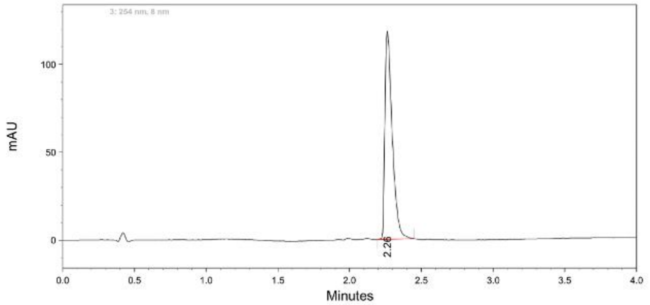

3: 254 nm, 8 nm

Retention Time	Height	Area	Area Percent
2.26	117741	421336	100.00

Instrument : LCMS AR
A:,Xtimate,2.1*30mm,3um
B:XBridge Shield, 2.1*50mm,5um
Confidential. For research only NOT for regulatory filing

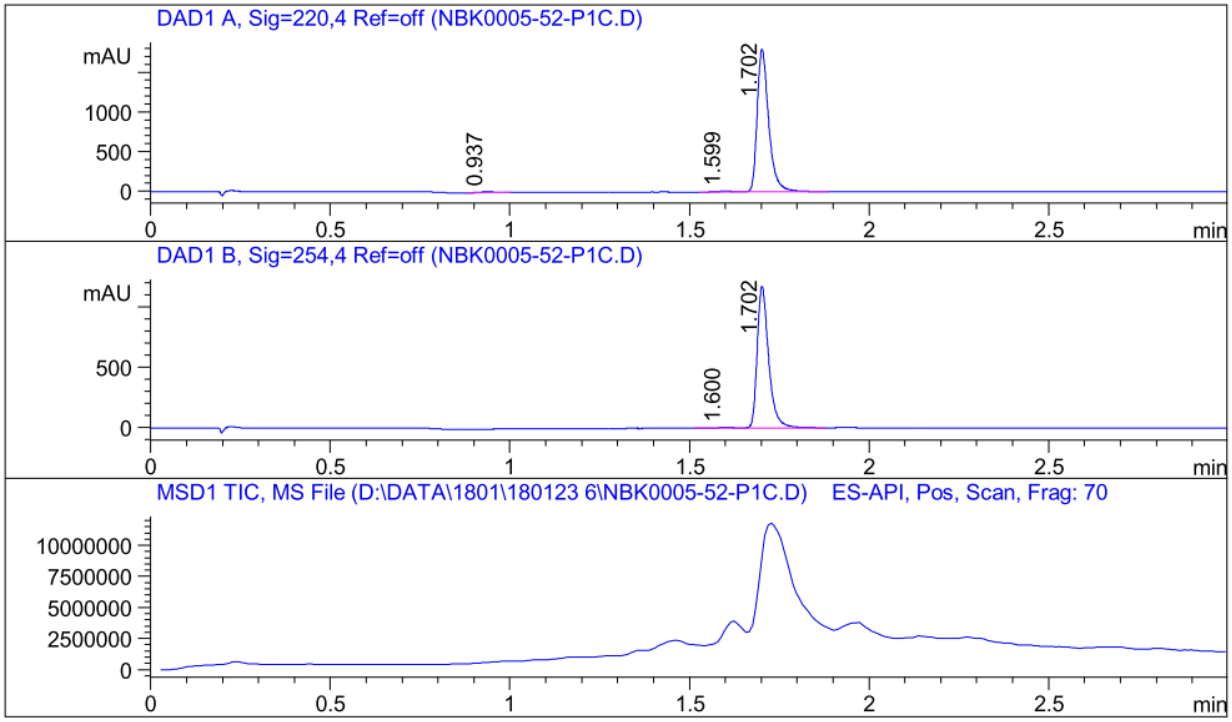

Report

==

Signal ->: DAD1 A, Sig=220,4 Ref=off

#	Meas. Ret.	Height	Width	Area	Area %
1	0.937	10.073	0.057	35.670	0.870
2	1.599	11.973	0.043	34.697	0.846
3	1.702	1793.106	0.034	4028.644	98.283

Signal ->: DAD1 B, Sig=254,4 Ref=off

#	Meas. Ret.	Height	Width	Area	Area %
1	1.600	7.728	0.042	22.315	0.843
2	1.702	1175.482	0.034	2623.948	99.157

Method :Column: Chiralcel OJ-H 150*4.6mm I.D., 5um
Mobile phase: A:CO2 B:methanol(0.05% DEA)
Gradient: hold 5% for 0.5 min,then from 5% to 40% of B in 3.5 min and hold 40% for 2.5 min, then 5% of B for 1.5 min
Flow rate: 3mL/min Column temp:40 C

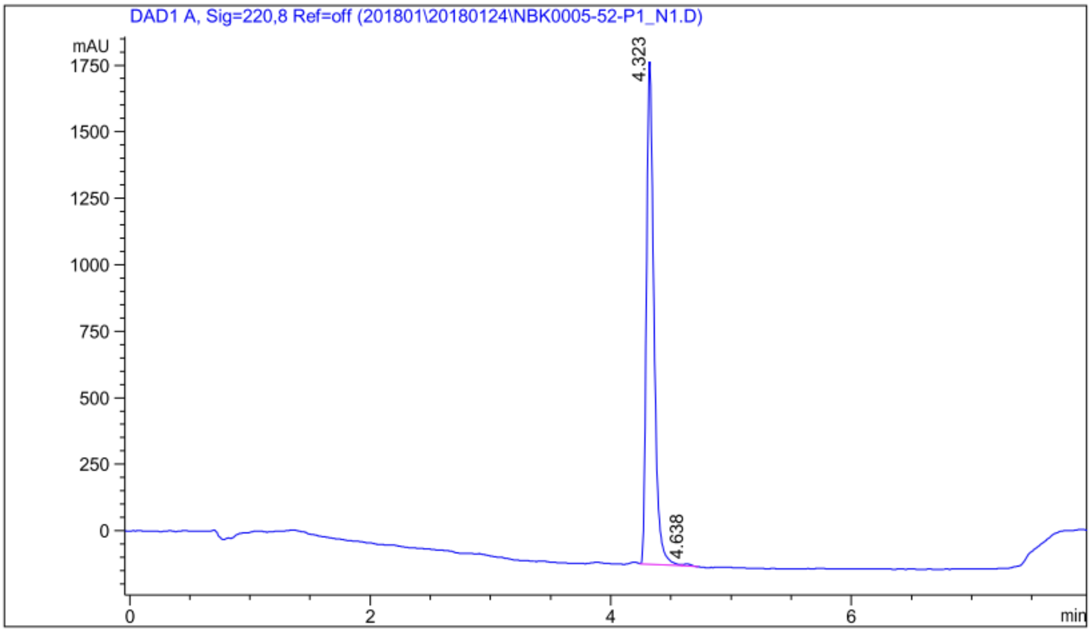

Signal 1 : DAD1 A, Sig=220,8 Ref=off

Peak	Meas. Ret. Time	Height	Height %	Width	Area	Area %
1	4.323	1898.586	99.564	0.072	8202.471	99.560
2	4.638	8.307	0.436	0.073	36.275	0.440

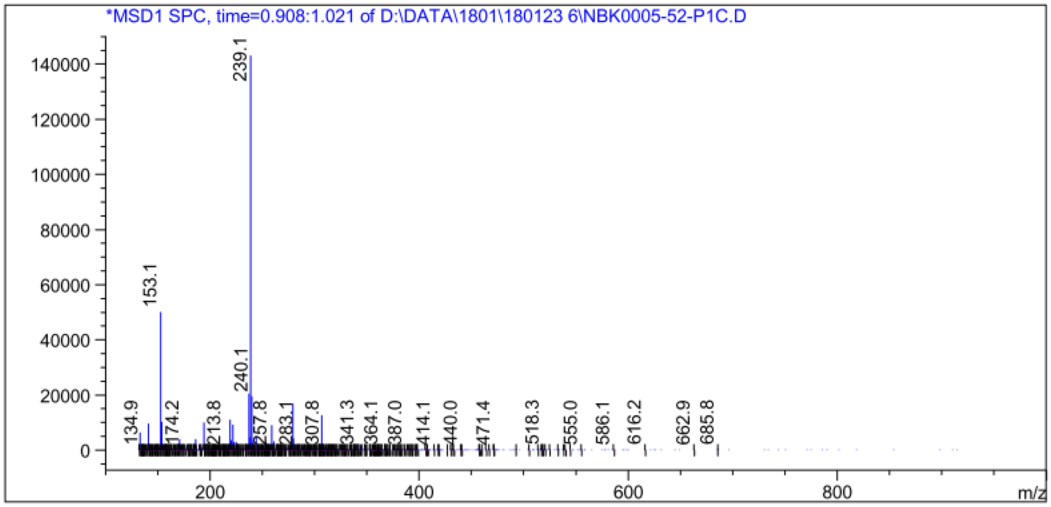
*MSD1 SPC, time=0.908:1.021 of D:\DATA\1801\180123 6\NBK0005-52-P1C.D
239.1
153.1
240.1
m/z

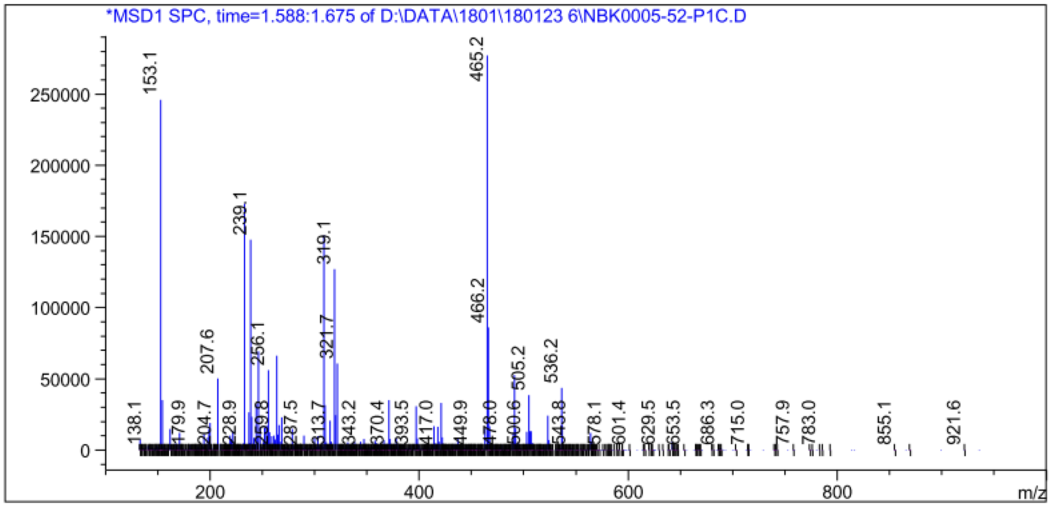
*MSD1 SPC, time=1.588:1.675 of D:\DATA\1801\180123 6\NBK0005-52-P1C.D
465.2
153.1
239.1
319.1
466.2
m/z

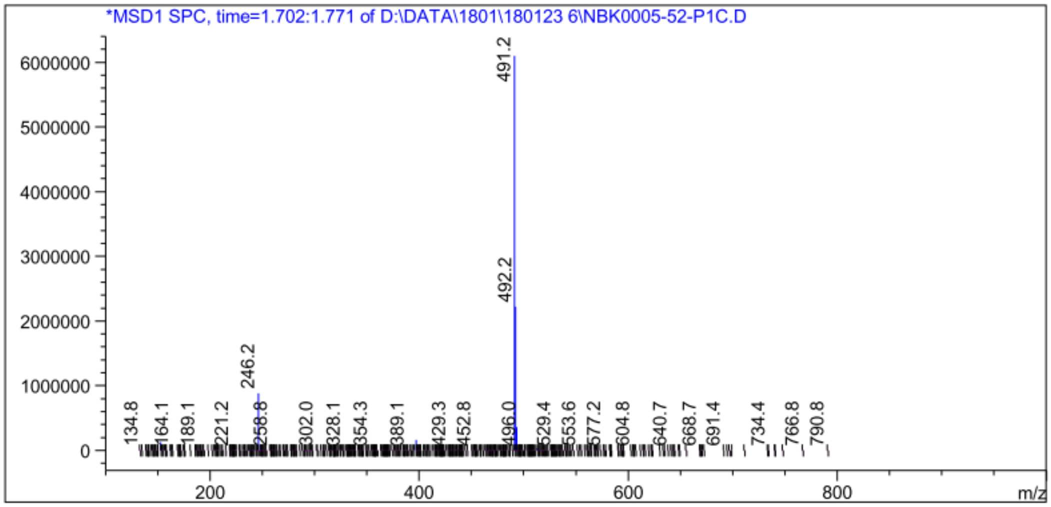
*MSD1 SPC, time=1.702:1.771 of D:\DATA\1801\180123 6\NBK0005-52-P1C.D
491.2
492.2
246.2
m/z

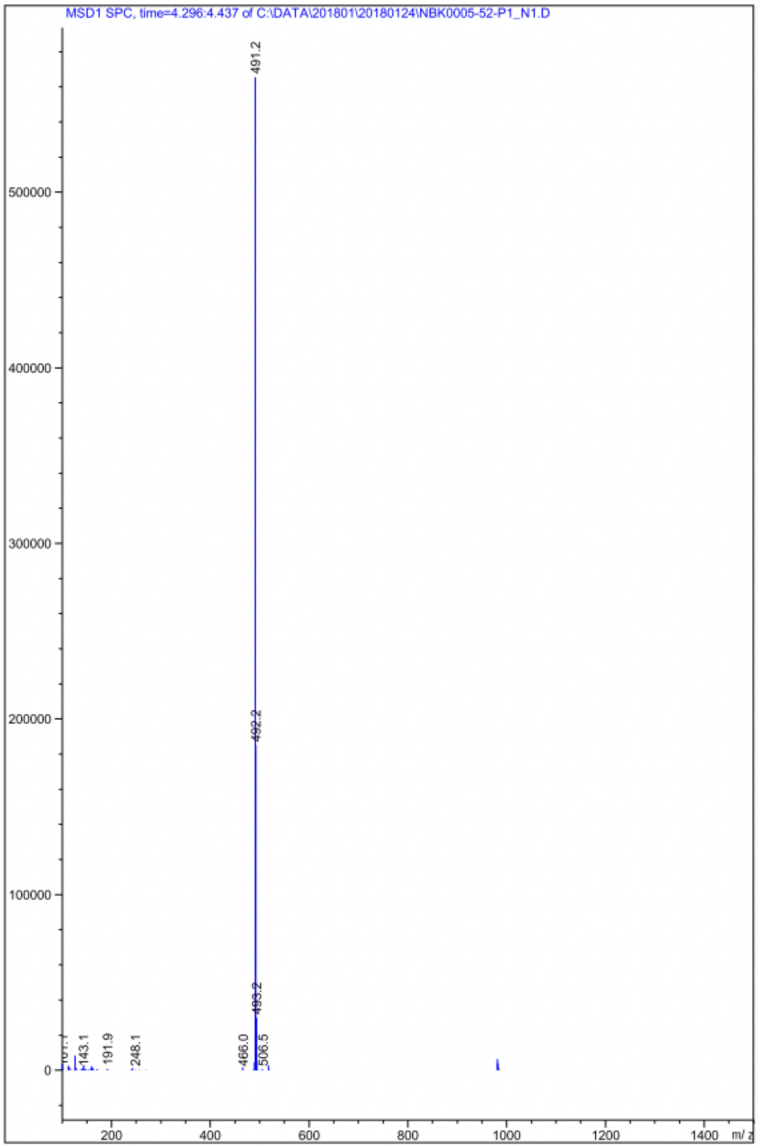
MSD1 SPC, time=4.296:4.437 of C:\DATA\201801\20180124\NBK0005-52-P1_N1.D
491.2
492.2
493.2
143.1
191.9
248.1
466.0
506.5
500000
400000
300000
200000
100000
0
200
400
600
800
1000
1200
1400
m/z

Compound 26

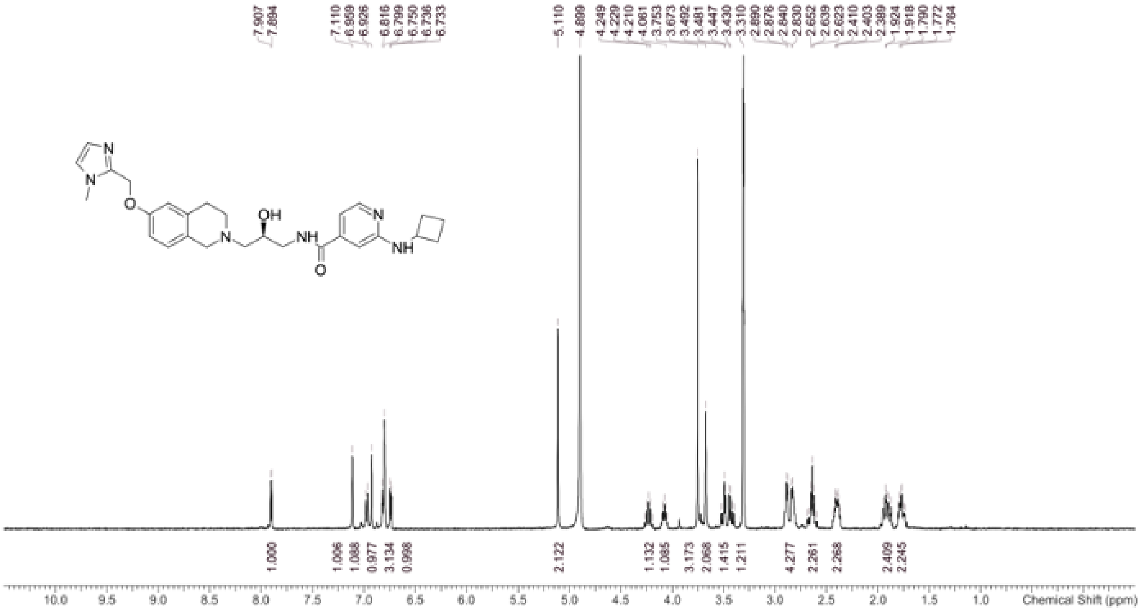

Compound 27

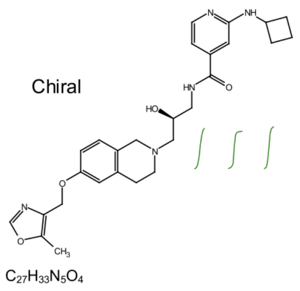

Chiral
N
NH
HN
O
HO
N
O
N
O
CH3
C27H33N5O4

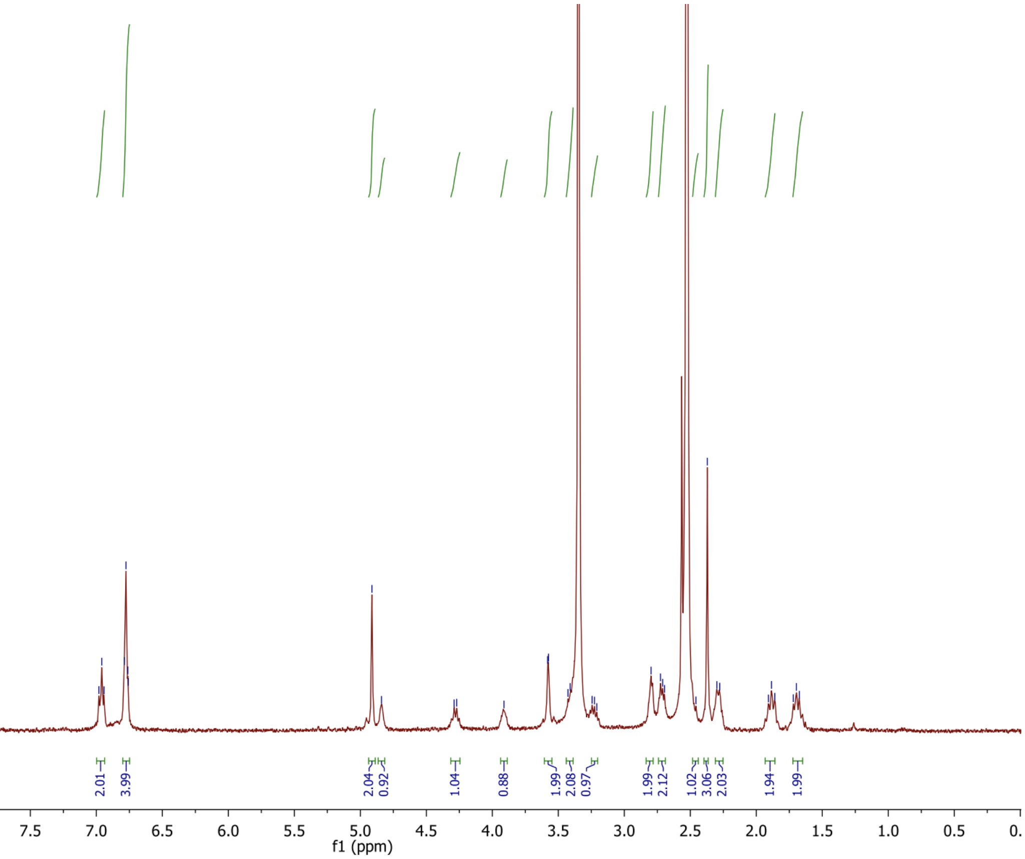

0.93
0.86
0.97
2.01
3.99
2.04
0.92
1.04
0.88
1.99
2.08
0.97
1.99
2.12
1.02
3.06
2.03
1.94
1.99
9.5
9.0
8.5
8.0
7.5
7.0
6.5
6.0
5.5
5.0
4.5
4.0
3.5
3.0
2.5
2.0
1.5
1.0
0.5
f1 (ppm)

Ret_Time: 0.843 min

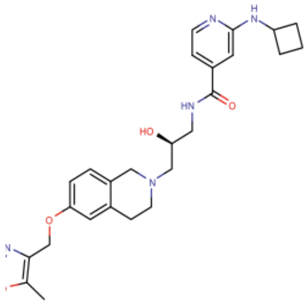

Mol Wt **491.58**

Exact Mass **491.29**

#	Time	Area%
1	0.843	100.00

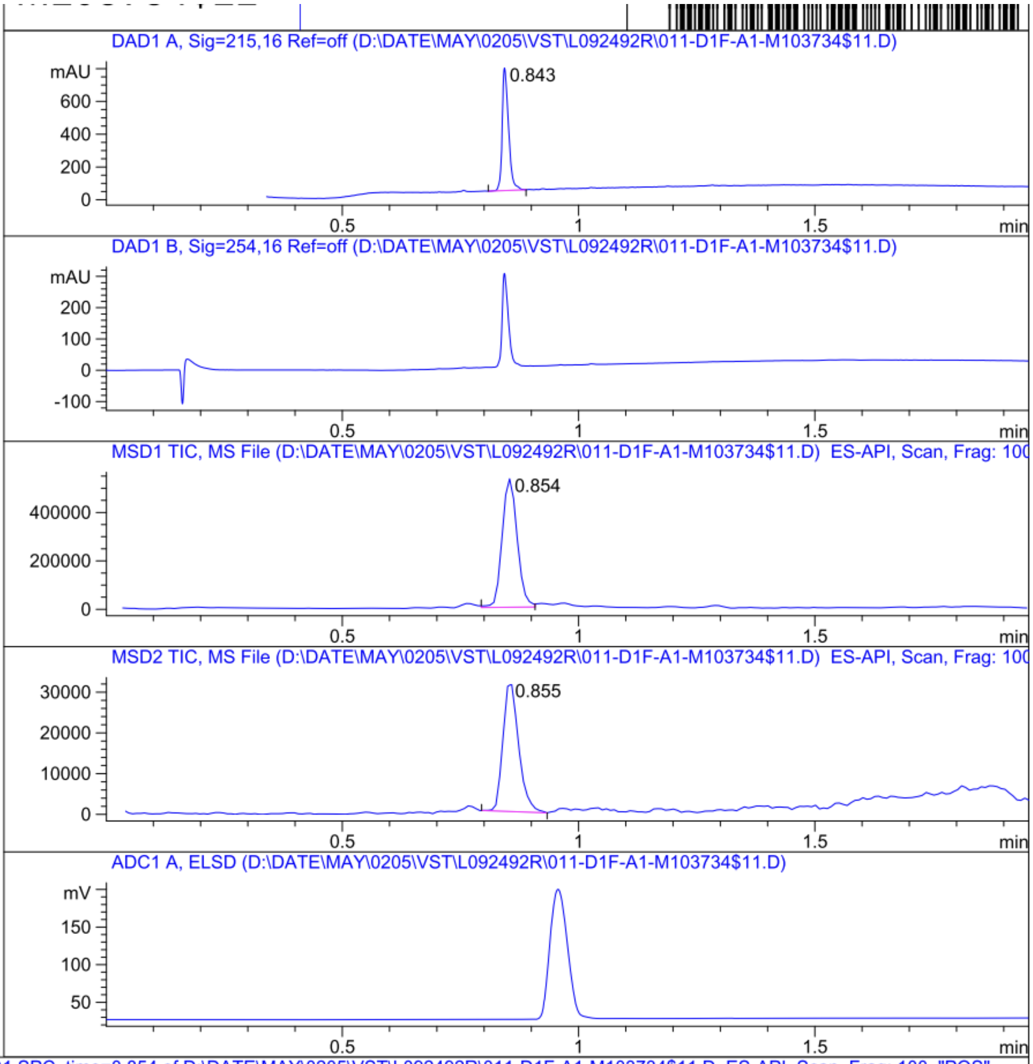

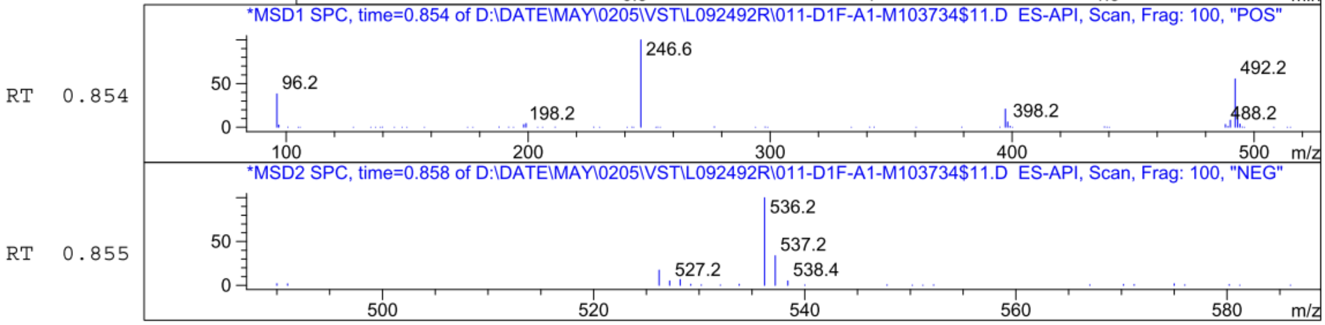

Compound 28

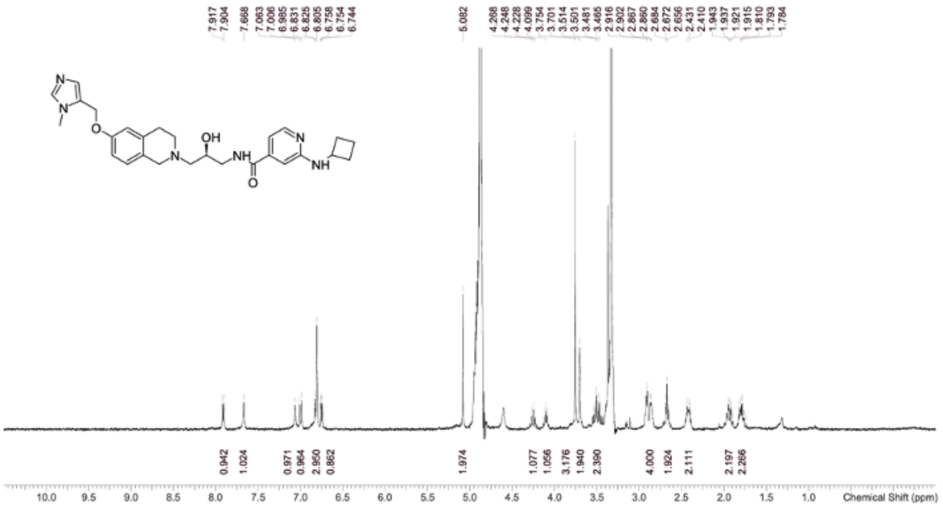

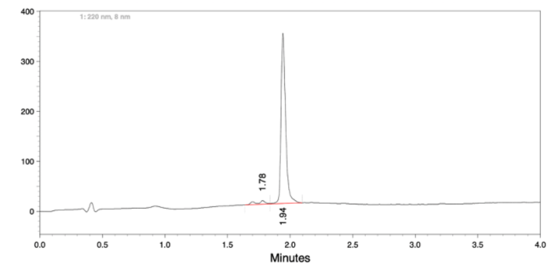

1: 220 nm, 8 nm

Retention Time	Height	Area	Area Percent
1.78	7125	34833	3.86
1.94	337080	867335	96.14

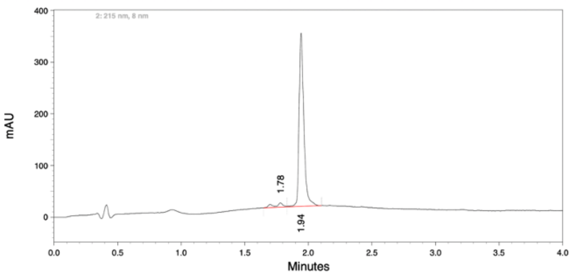

2: 215 nm, 8 nm

Retention Time	Height	Area	Area Percent
1.78	8122	40105	4.38
1.94	331691	874735	95.62

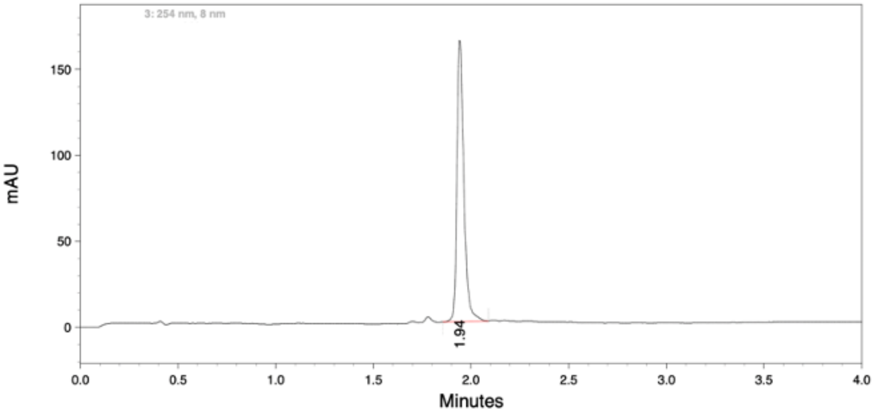

3: 254 nm, 8 nm

Retention Time	Height	Area	Area Percent
1.94	161687	404477	100.00

```
Method              :Column: Chiralcel OJ-H 150*4.6mm I.D., 5um
                     Mobile phase: A:CO2  B:methanol(0.05% DEA)
                     Gradient: hold 5% for 0.5 min,then from 5% to 40% of B
                     in 3.5 min and hold 40% for 2.5 min,  then 5% of B for
                     1.5 min
                     Flow rate: 3mL/min   Column temp:40 C
```

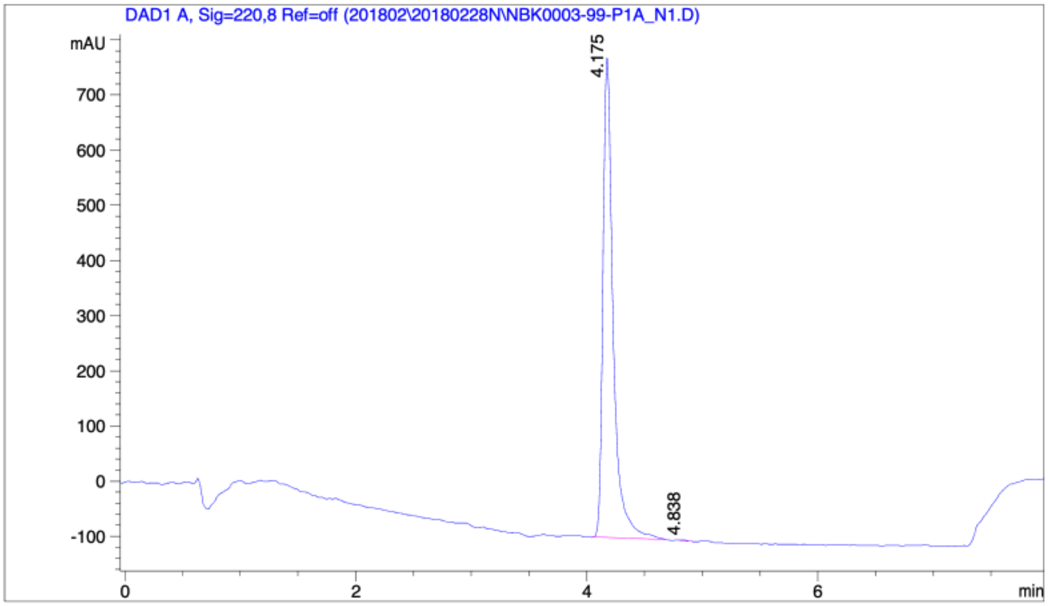

Signal 1 :   DAD1 A, Sig=220,8 Ref=off

Peak	Meas. Ret. Time	Height	Height %	Width	Area	Area %
1	4.175	873.051	99.825	0.102	5362.419	99.898
2	4.838	1.531	0.175	0.059	5.461	0.102

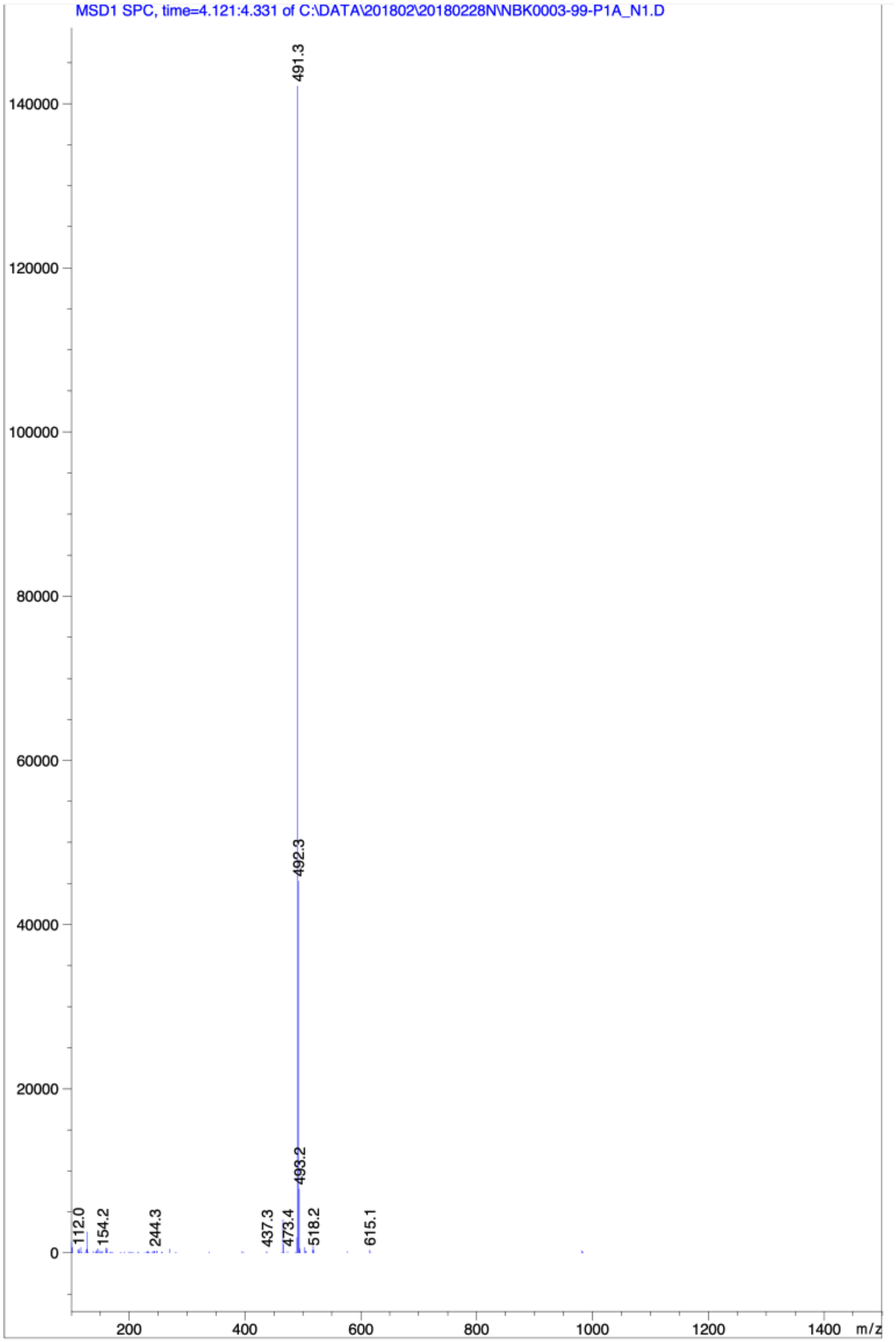

MSD1 SPC, time=4.121:4.331 of C:\DATA\201802\20180228N\NBK0003-99-P1A_N1.D
491.3
492.3
493.2
112.0
154.2
244.3
437.3
473.4
518.2
615.1
140000
120000
100000
80000
60000
40000
20000
0
200
400
600
800
1000
1200
1400
m/z

Compound 29

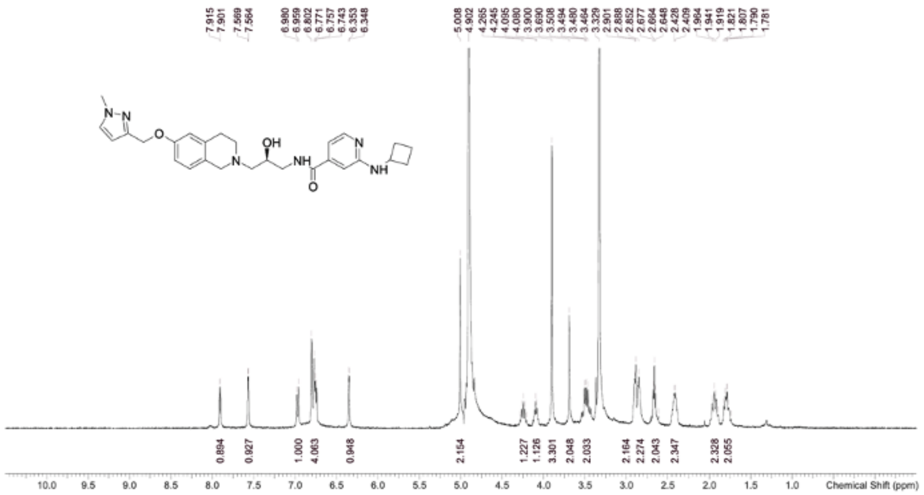

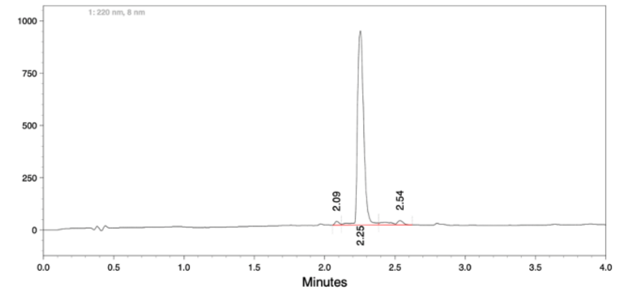

1: 220 nm, 8 nm

Retention Time	*Height*	*Area*	*Area Percent*
2.09	17529	38114	1.27
2.25	925359	2828556	94.53
2.54	19898	125426	4.19

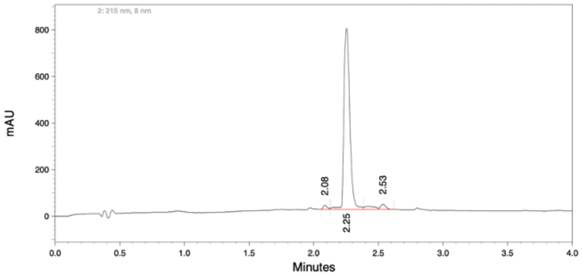

2: 215 nm, 8 nm

Retention Time	*Height*	*Area*	*Area Percent*
2.08	16895	38930	1.48
2.25	773955	2454079	93.45
2.53	22211	133097	5.07

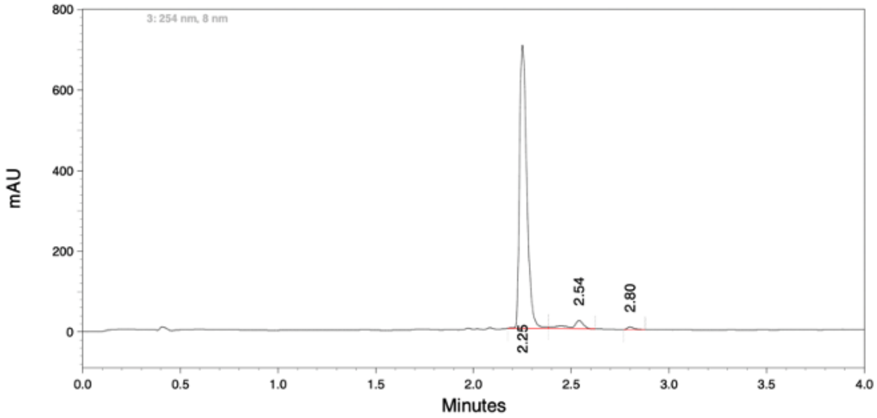

3: 254 nm, 8 nm

Retention Time	*Height*	*Area*	*Area Percent*
2.25	692393	1819828	94.61
2.54	20057	88228	4.59
2.80	5976	15518	0.81

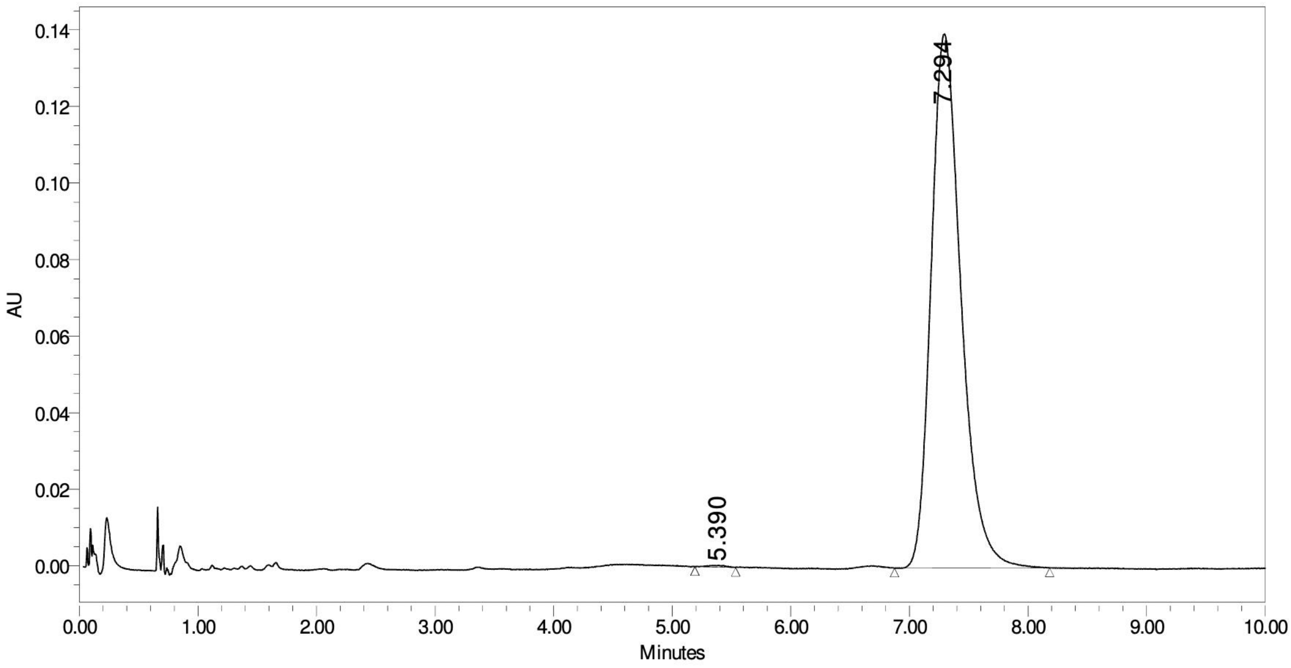

	RT	Area	% Area
1	5.390	4877	0.20
2	7.294	2446826	99.80

Instrument : LCMS AR
A:,Xtimate,2.1*30mm,3um
B:XBridge Shield, 2.1*50mm,5um
Confidential. For research only NOT for regulatory filing

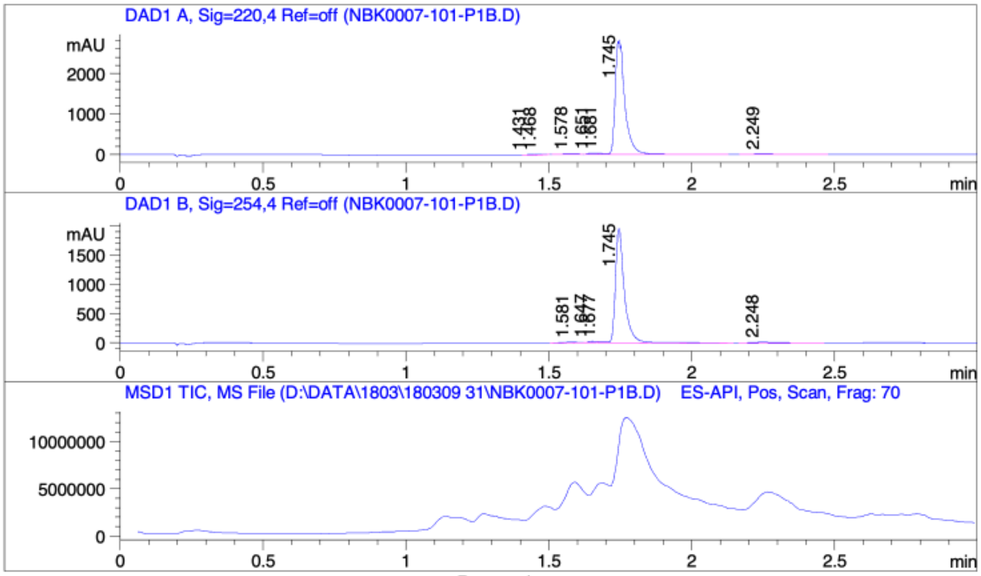

Report

===

Signal ->: DAD1 A, Sig=220,4 Ref=off

#	Meas. Ret.	Height	Width	Area	Area %
1	1.431	4.929	0.022	7.059	0.104
2	1.468	9.528	0.026	16.675	0.246
3	1.578	17.126	0.053	57.202	0.843
4	1.651	28.471	0.026	48.034	0.708
5	1.681	19.277	0.028	35.613	0.525
6	1.745	2772.809	0.035	6517.568	96.071
7	2.249	17.037	0.081	101.971	1.503

Signal ->: DAD1 B, Sig=254,4 Ref=off

#	Meas. Ret.	Height	Width	Area	Area %
1	1.581	13.185	0.049	40.461	0.916
2	1.647	24.227	0.025	42.518	0.962
3	1.677	23.864	0.024	33.787	0.765
4	1.745	1949.610	0.032	4225.659	95.651
5	2.248	12.740	0.084	75.377	1.706

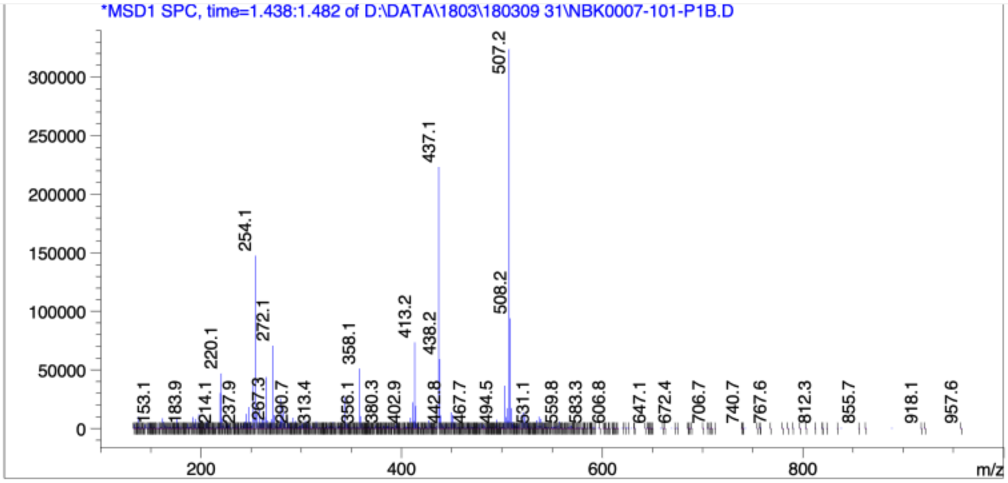
*MSD1 SPC, time=1.438:1.482 of D:\DATA\1803\180309 31\NBK0007-101-P1B.D
507.2
437.1
254.1
508.2
413.2
272.1
220.1
358.1
438.2
m/z

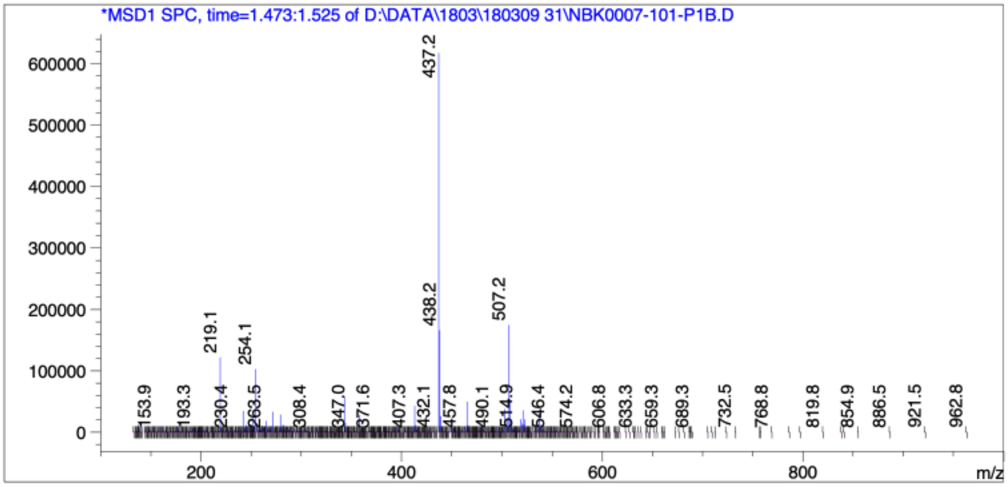
*MSD1 SPC, time=1.473:1.525 of D:\DATA\1803\180309 31\NBK0007-101-P1B.D
437.2
438.2
507.2
219.1
254.1
m/z

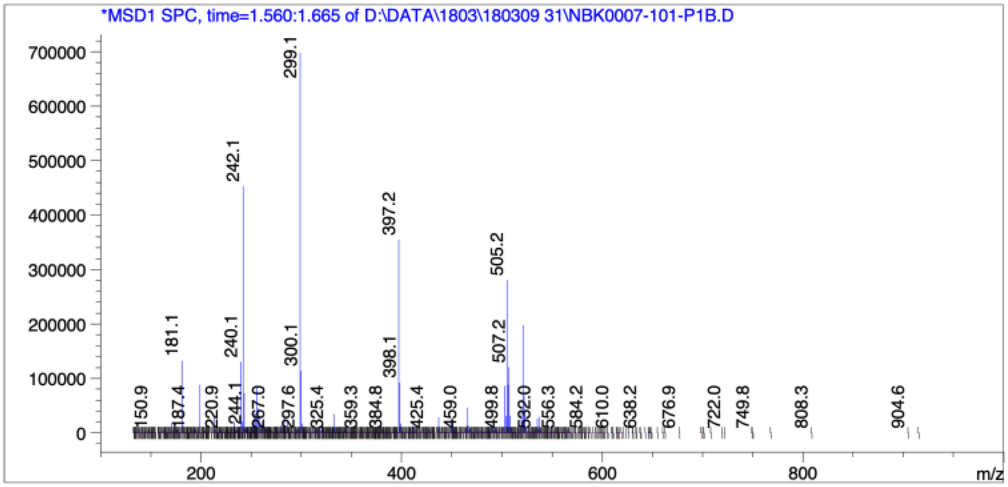
*MSD1 SPC, time=1.560:1.665 of D:\DATA\1803\180309 31\NBK0007-101-P1B.D
299.1
242.1
397.2
505.2
181.1
240.1
300.1
507.2
398.1
m/z

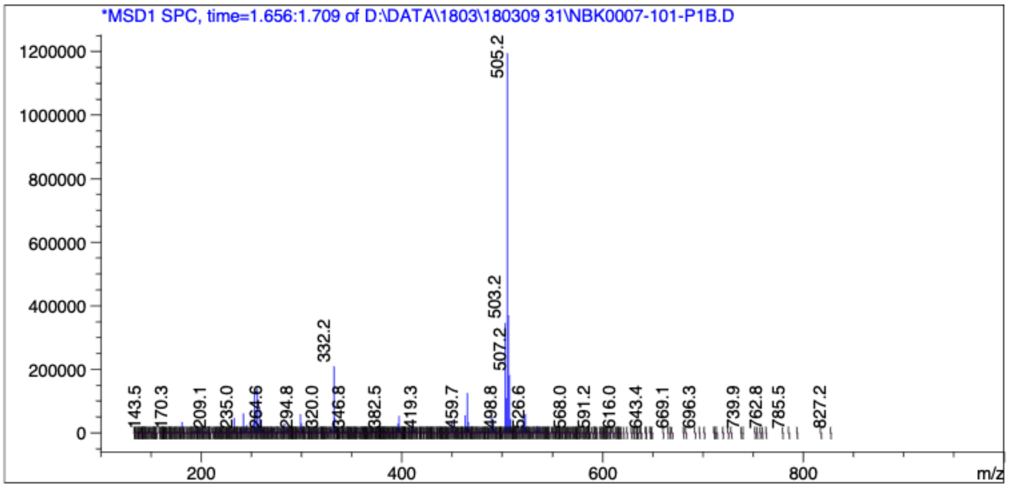
*MSD1 SPC, time=1.656:1.709 of D:\DATA\1803\180309 31\NBK0007-101-P1B.D
505.2
503.2
332.2
507.2
m/z

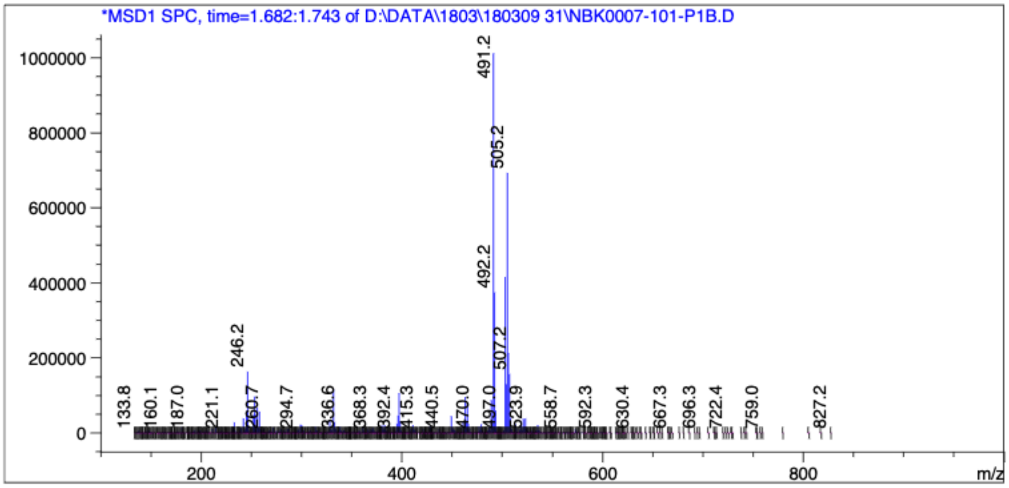
*MSD1 SPC, time=1.682:1.743 of D:\DATA\1803\180309 31\NBK0007-101-P1B.D
491.2
505.2
492.2
246.2
507.2
m/z

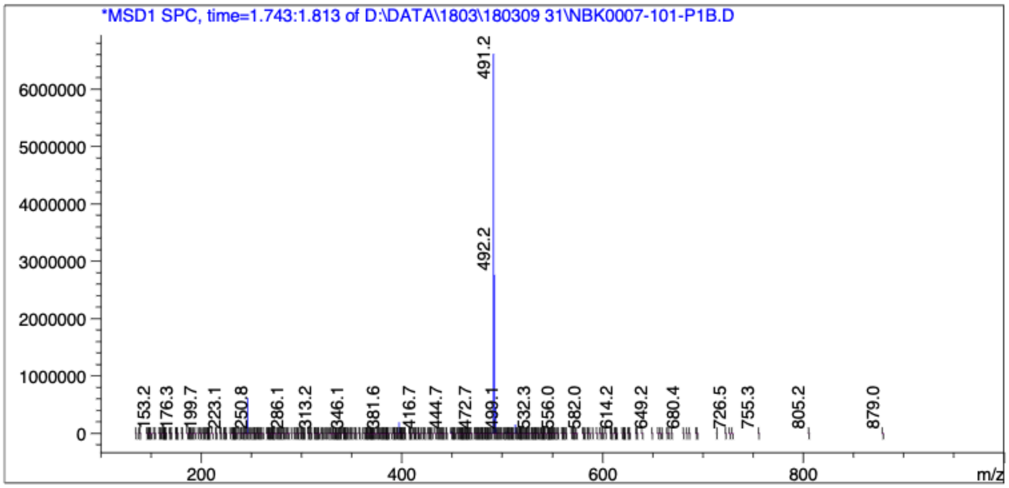
*MSD1 SPC, time=1.743:1.813 of D:\DATA\1803\180309 31\NBK0007-101-P1B.D
491.2
492.2
m/z

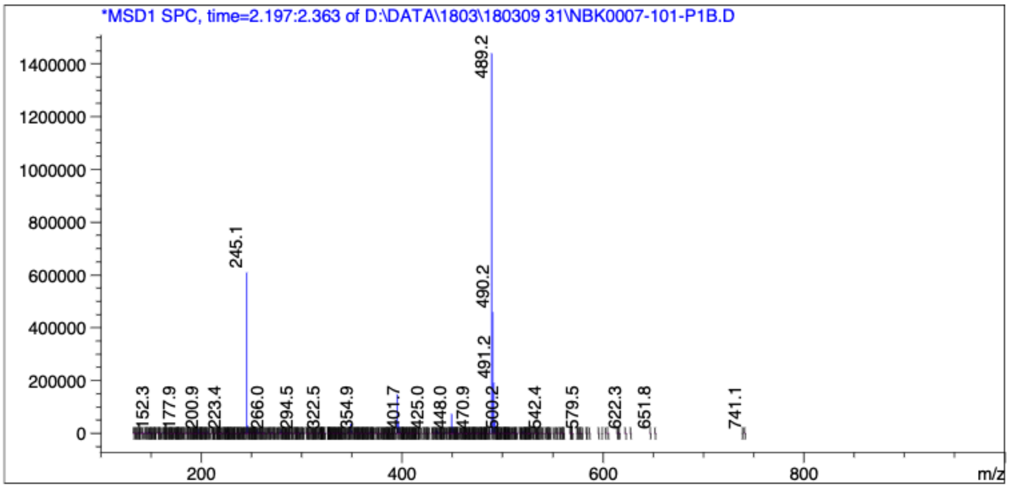
*MSD1 SPC, time=2.197:2.363 of D:\DATA\1803\180309 31\NBK0007-101-P1B.D
1400000
1200000
1000000
800000
600000
400000
200000
0
489.2
245.1
490.2
491.2
152.3
177.9
200.9
223.4
266.0
294.5
322.5
354.9
401.7
425.0
448.0
470.9
500.2
542.4
579.5
622.3
651.8
741.1
200
400
600
800
m/z

Compound 30

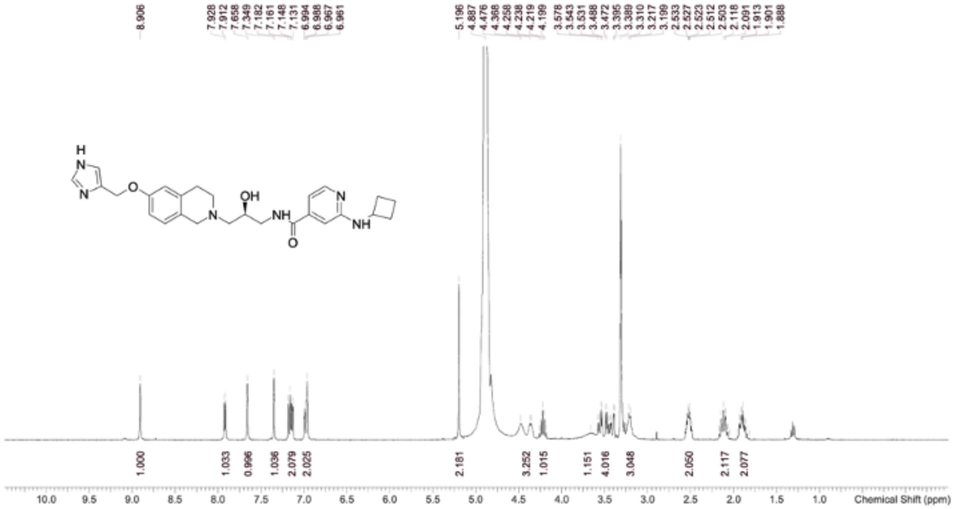

Method : Column: ChiralPak IG-3 100×4.6mm I.D., 3um
Mobile phase: A: CO2 B:ethanol (0.05% DEA)
Isocratic: 40% B
Flow rate: 3mL/min
Column temp.:40℃
ABPR: 100 bar

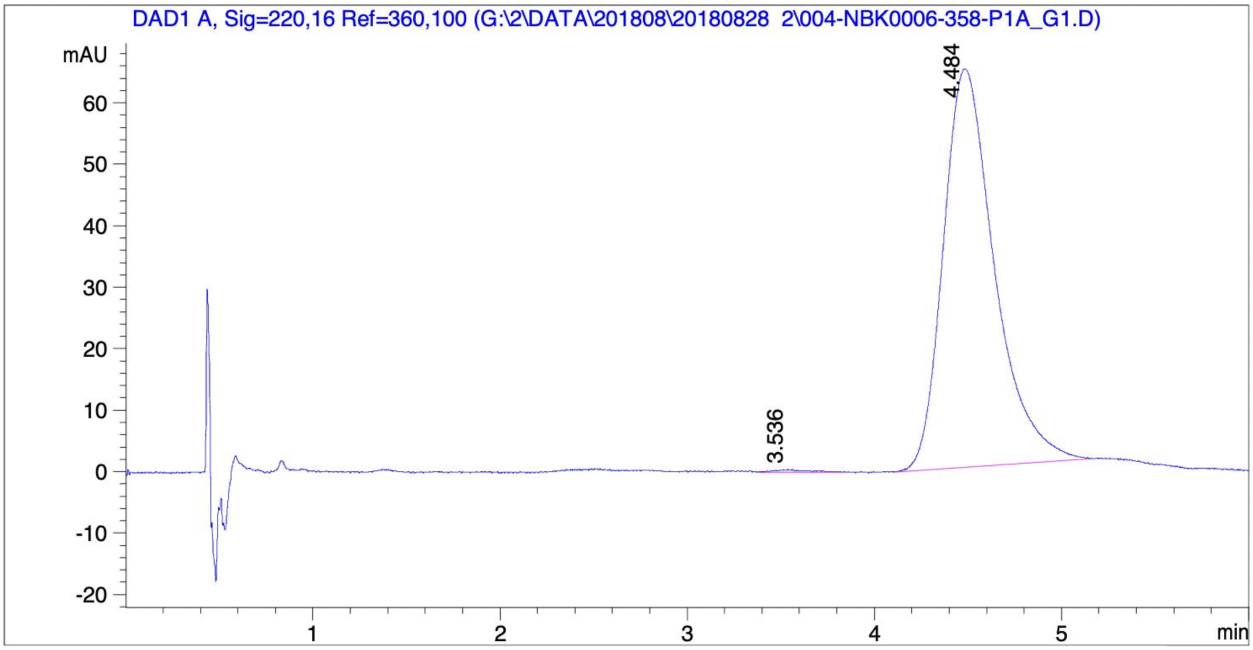

DAD1 A, Sig=220,16 Ref=360,100

#	Meas. Ret. Time	Height	Height %	Width	Area	Area %
1	3.536	0.417	0.640	0.228	5.715	0.462
2	4.484	64.799	99.360	0.226	1230.208	99.538

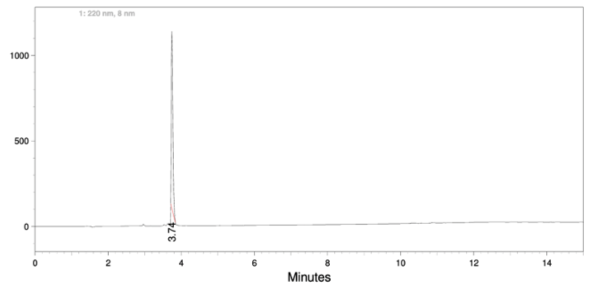

1: 220 nm, 8 nm

Retention Time	*Height*	*Area*	*Area Percent*
3.74	1028185	2986267	100.00

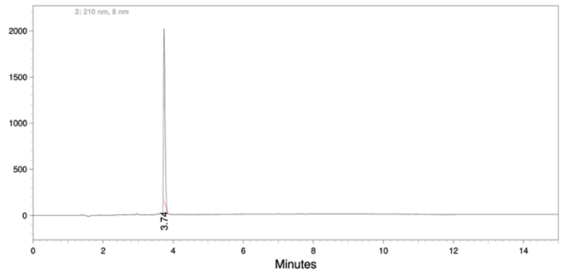

2: 210 nm, 8 nm

Retention Time	*Height*	*Area*	*Area Percent*
3.74	1860858	4917307	100.00

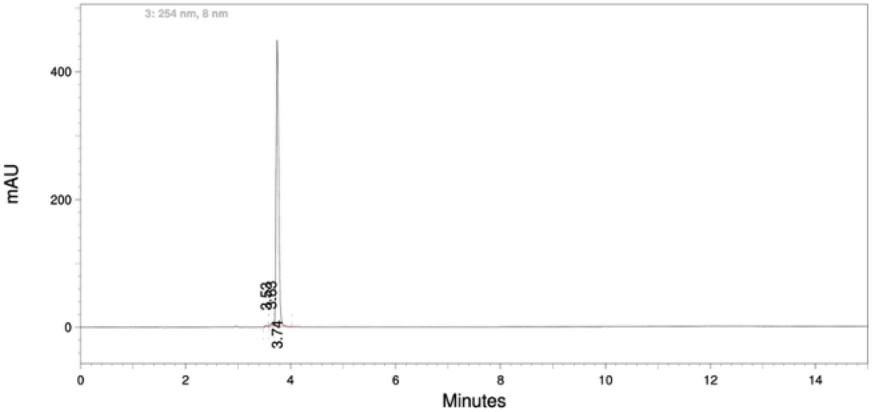

3: 254 nm, 8 nm

Retention Time	*Height*	*Area*	*Area Percent*
3.53	2549	7876	0.53
3.63	3843	10982	0.74
3.74	447129	1463745	98.73

Compound 31

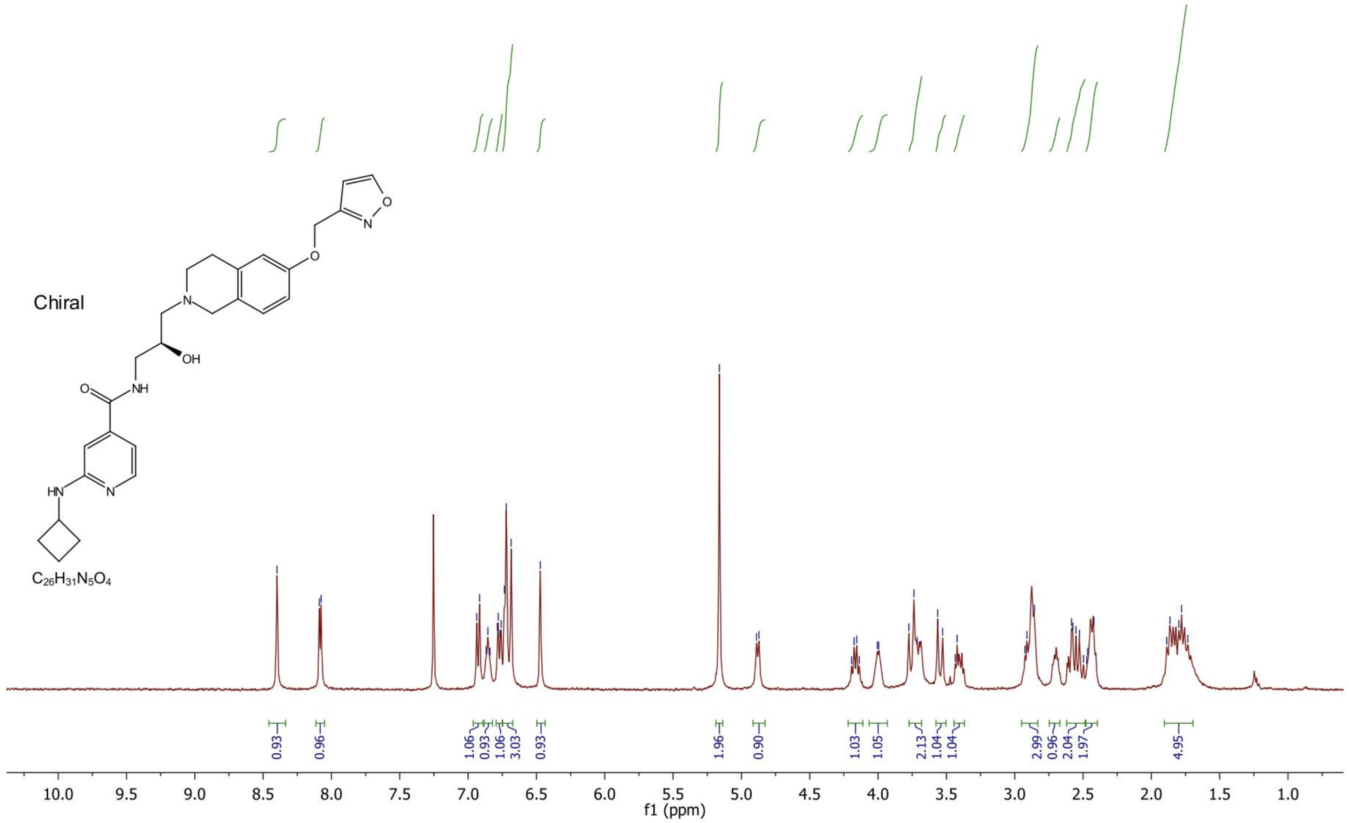

Chiral
$C_{26}H_{31}N_5O_4$
f1 (ppm)

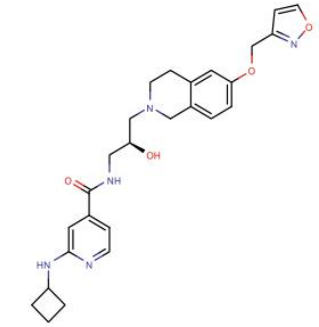

Mol Wt **477.56**

Exact Mass **477.27**

```
#  Time   Area%
-----------------
1  0.816  100.00
```

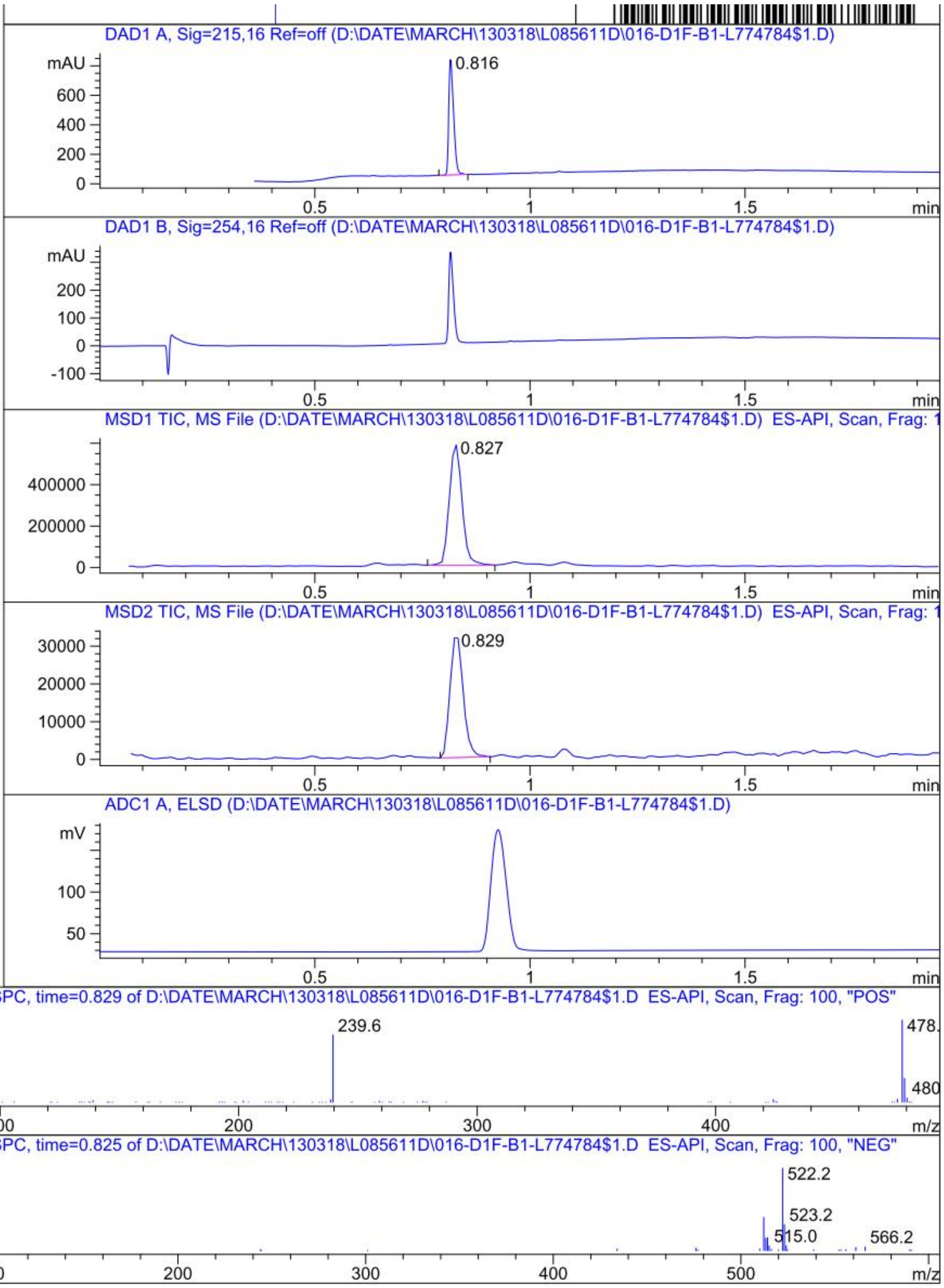

RT 0.827

RT 0.829

Compound 32

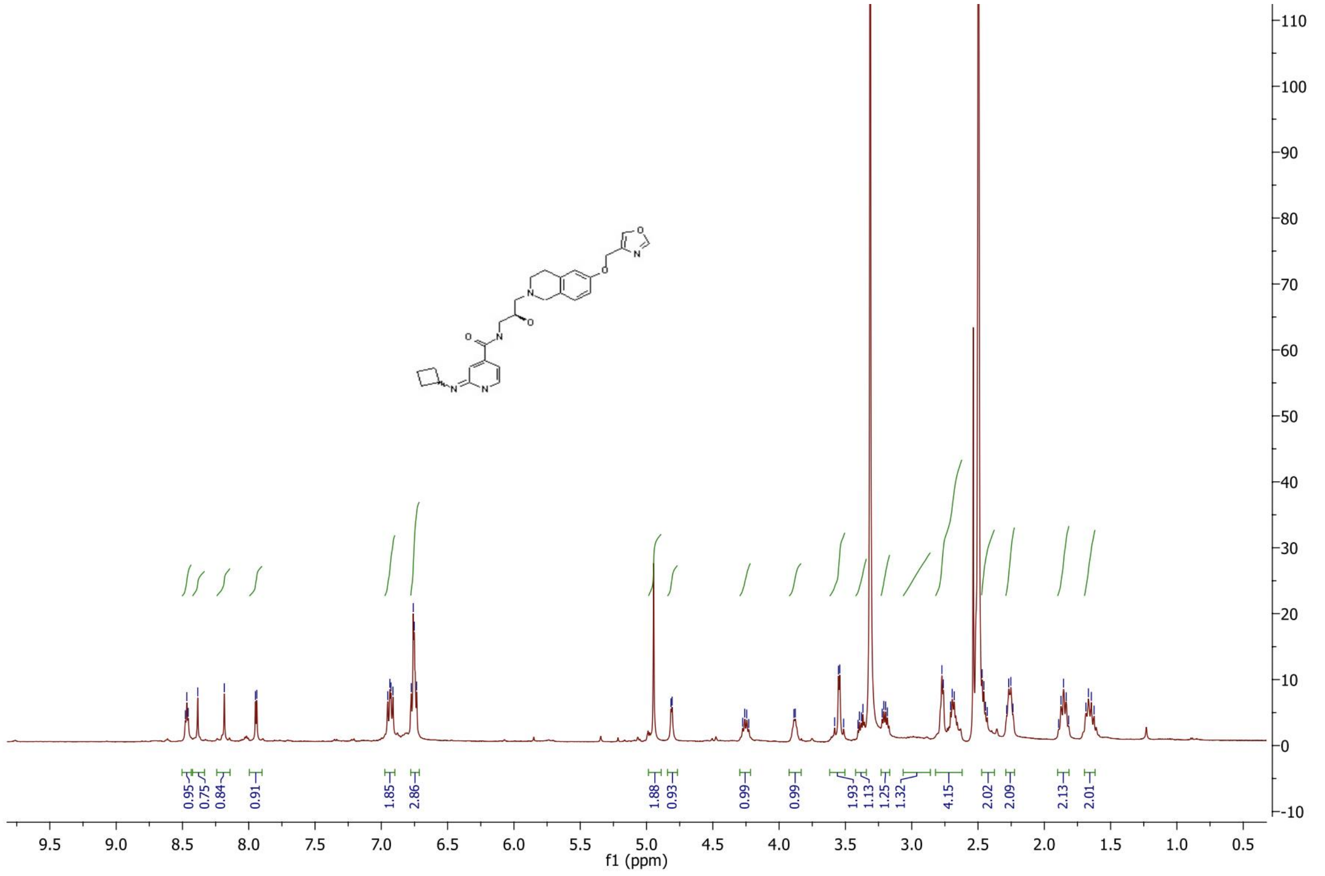

0.95
0.75
0.84
0.91
1.85
2.86
1.88
0.93
0.99
0.99
1.93
1.13
1.25
1.32
4.15
2.02
2.09
2.13
2.01
f1 (ppm)

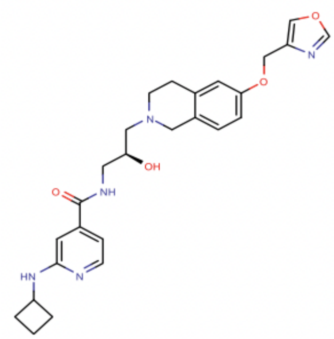

Mol Wt 477.56

Exact Mass 477.27

#	Time	Area%
1	0.607	2.19
2	0.698	95.63
3	0.822	2.18

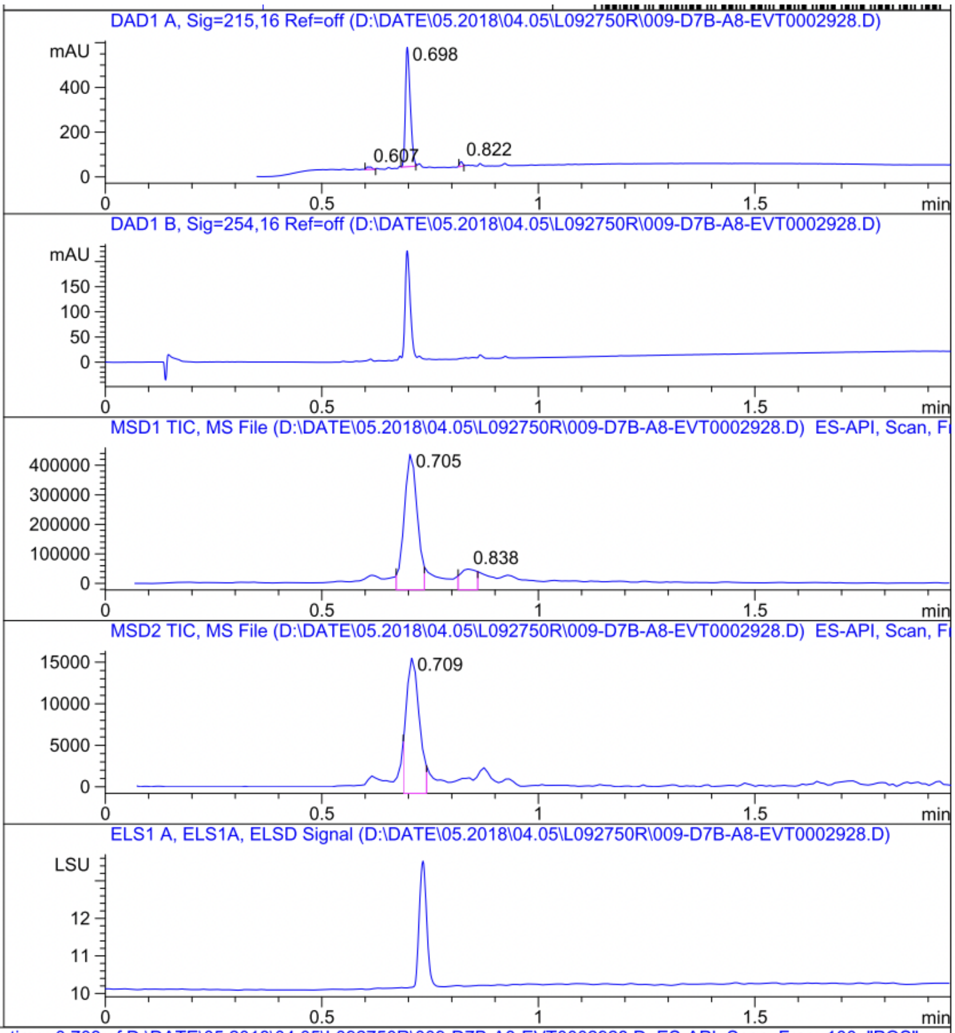

RT 0.705

RT 0.838

RT 0.709

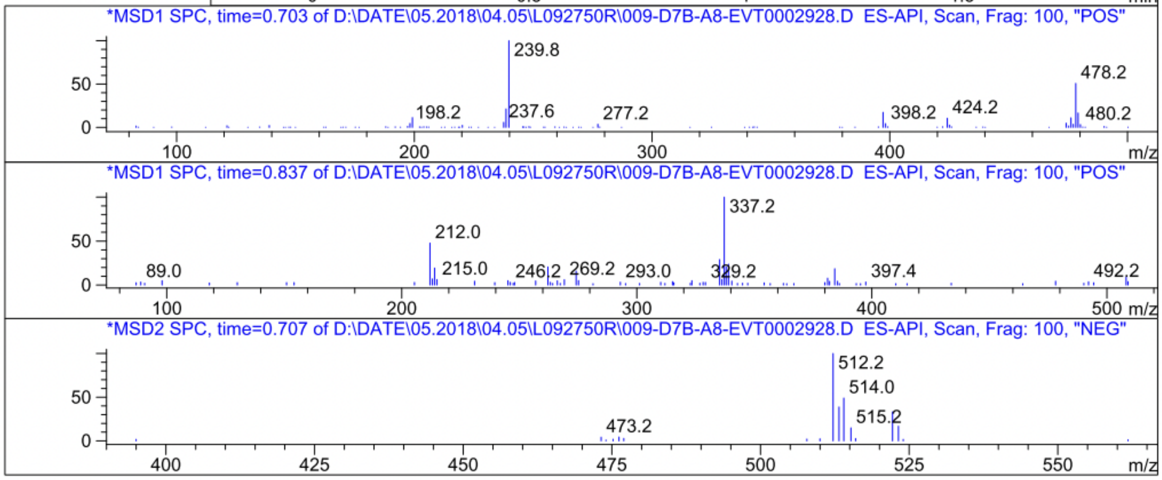

Compound 33

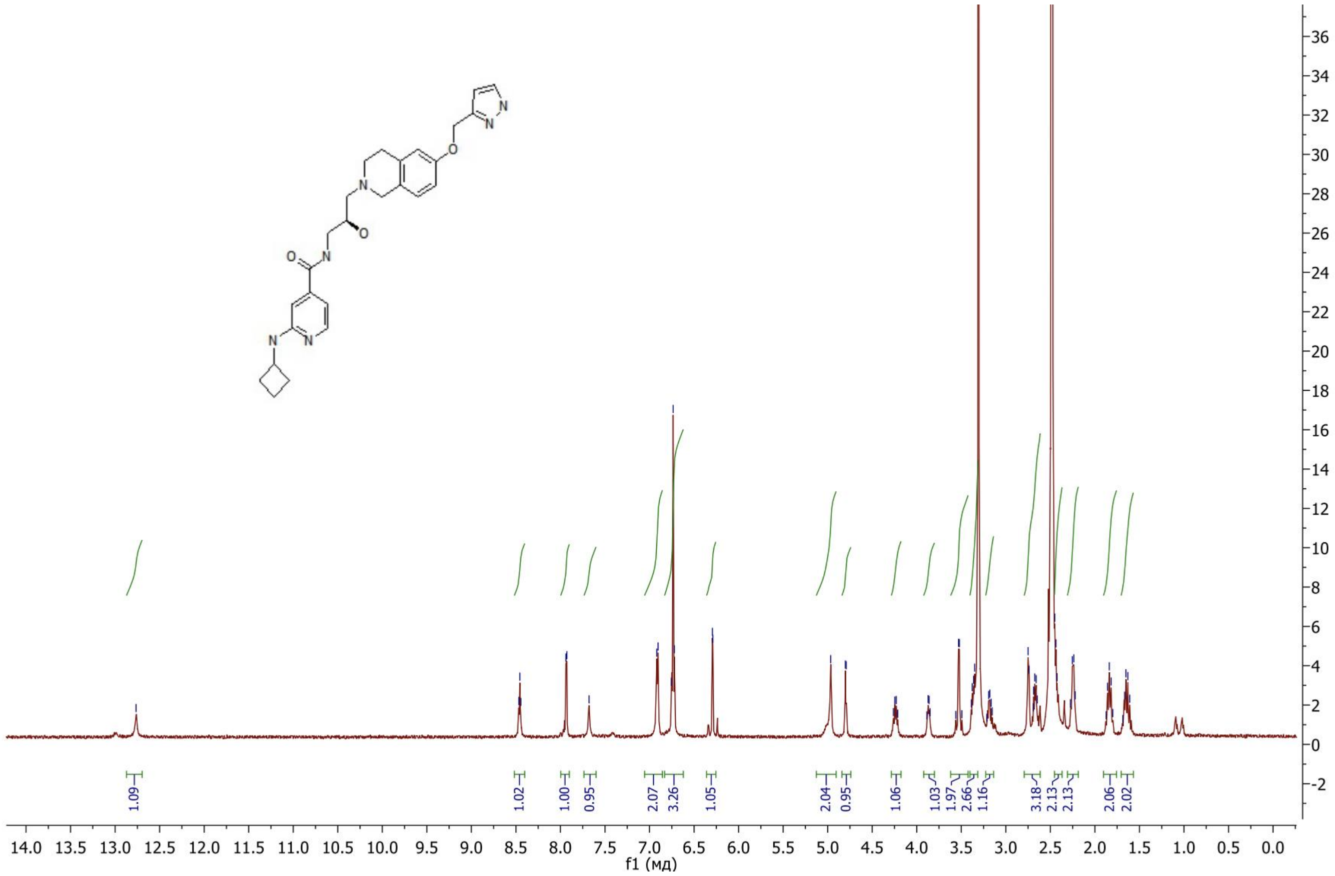
f1 (мд)

```
MaxPeak:  95.98%
Ret_Time: 0.791 min
```

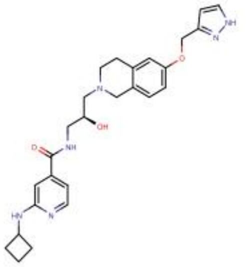

Mol Wt 476.57

Exact Mass 476.29

#	Time	Area%
1	0.791	95.98
2	0.877	2.95
3	1.011	1.07

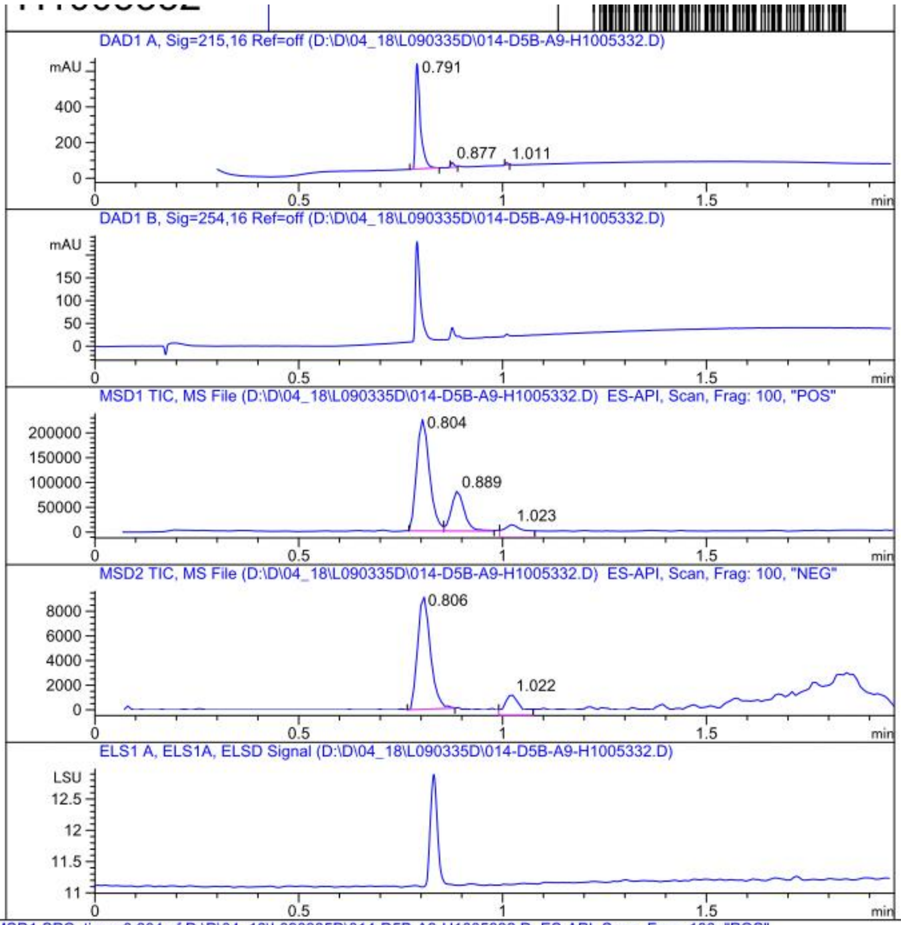

RT 0.804

RT 0.889

RT 1.023

RT 0.806

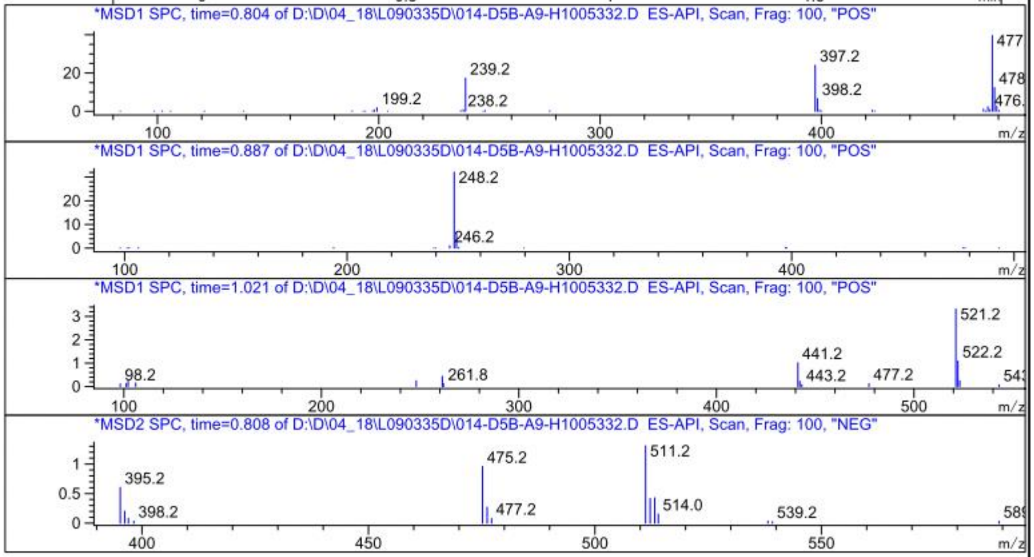

RT 1.022

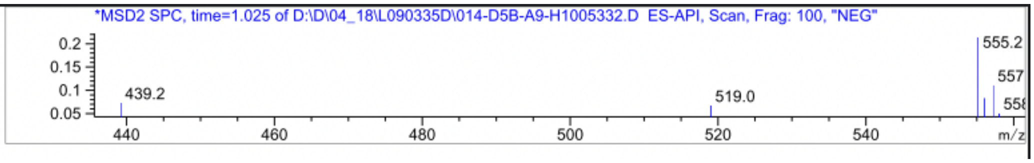

Compound 34

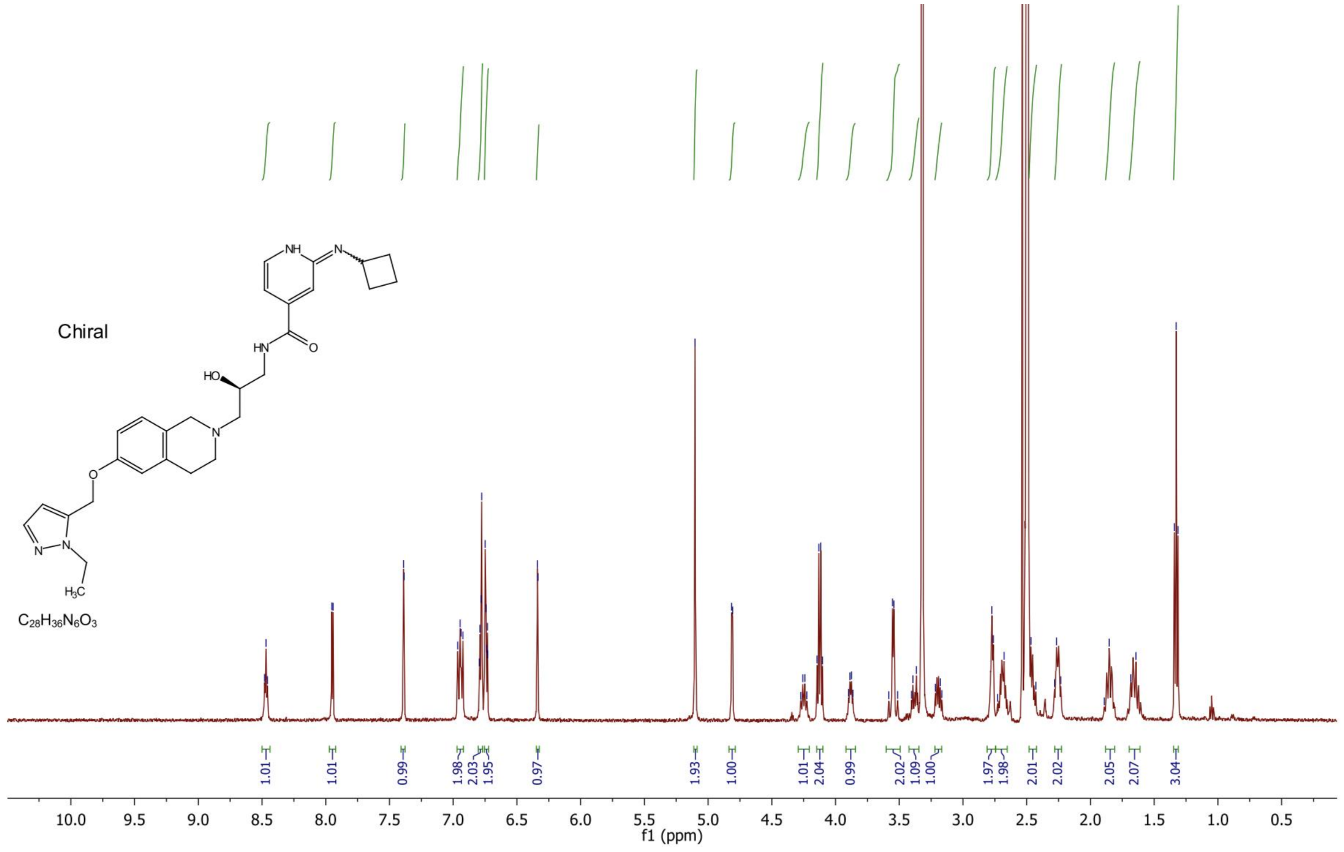
Chiral
$C_{28}H_{36}N_6O_3$
1.01
1.01
0.99
1.98
2.03
1.95
0.97
1.93
1.00
1.01
2.04
0.99
2.02
1.09
1.00
1.97
1.98
2.01
2.02
2.05
2.07
3.04
10.0 9.5 9.0 8.5 8.0 7.5 7.0 6.5 6.0 5.5 5.0 4.5 4.0 3.5 3.0 2.5 2.0 1.5 1.0 0.5
f1 (ppm)

MaxPeak: 100.00%
Ret_Time: 0.904 min

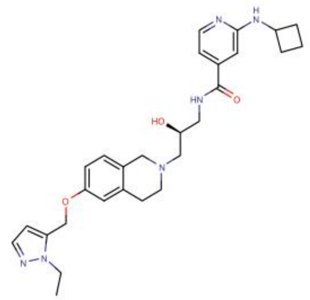

Mol Wt 504.62
Exact Mass 504.33

#	Time	Area%
1	0.904	100.00

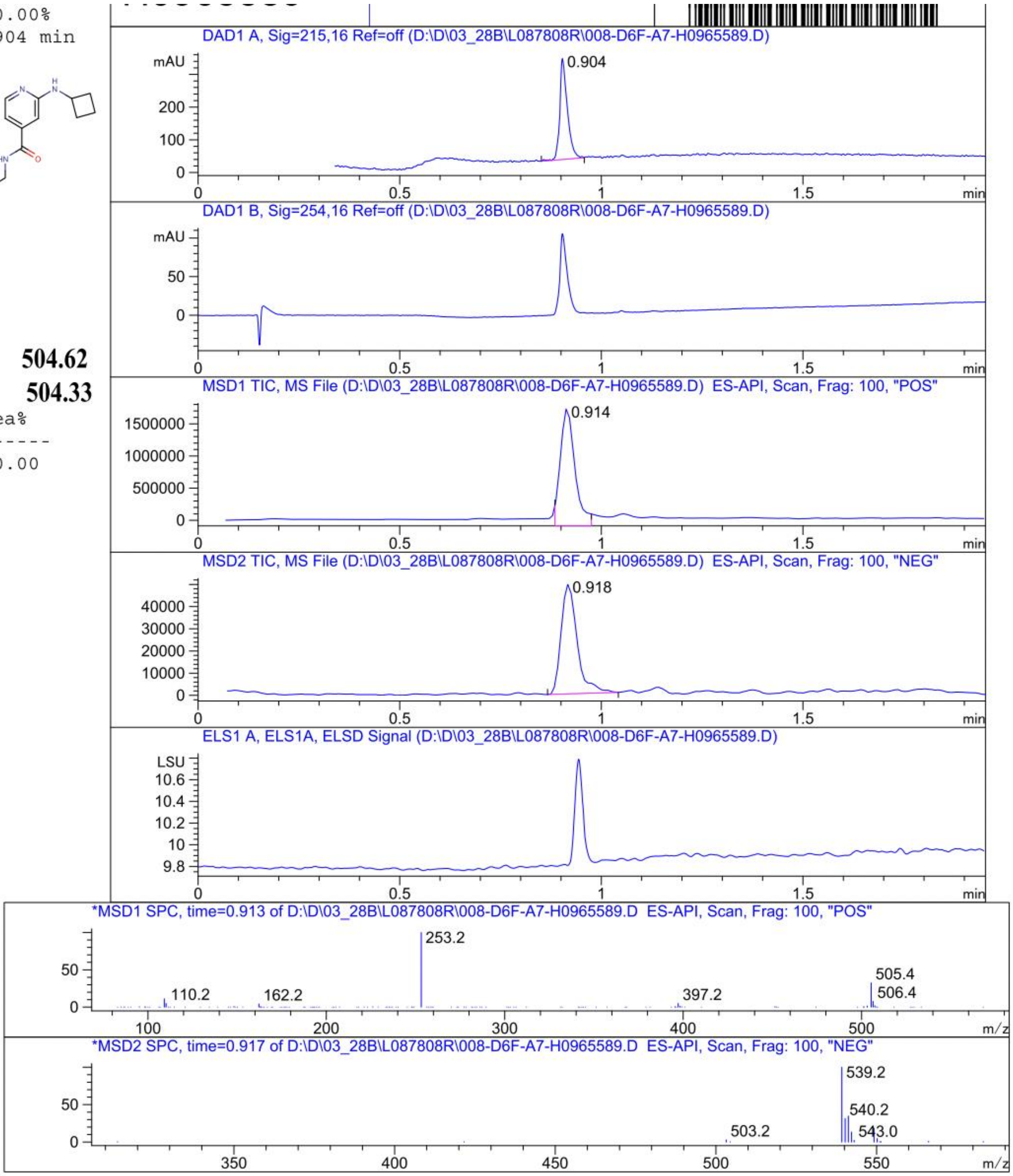

Compound 35

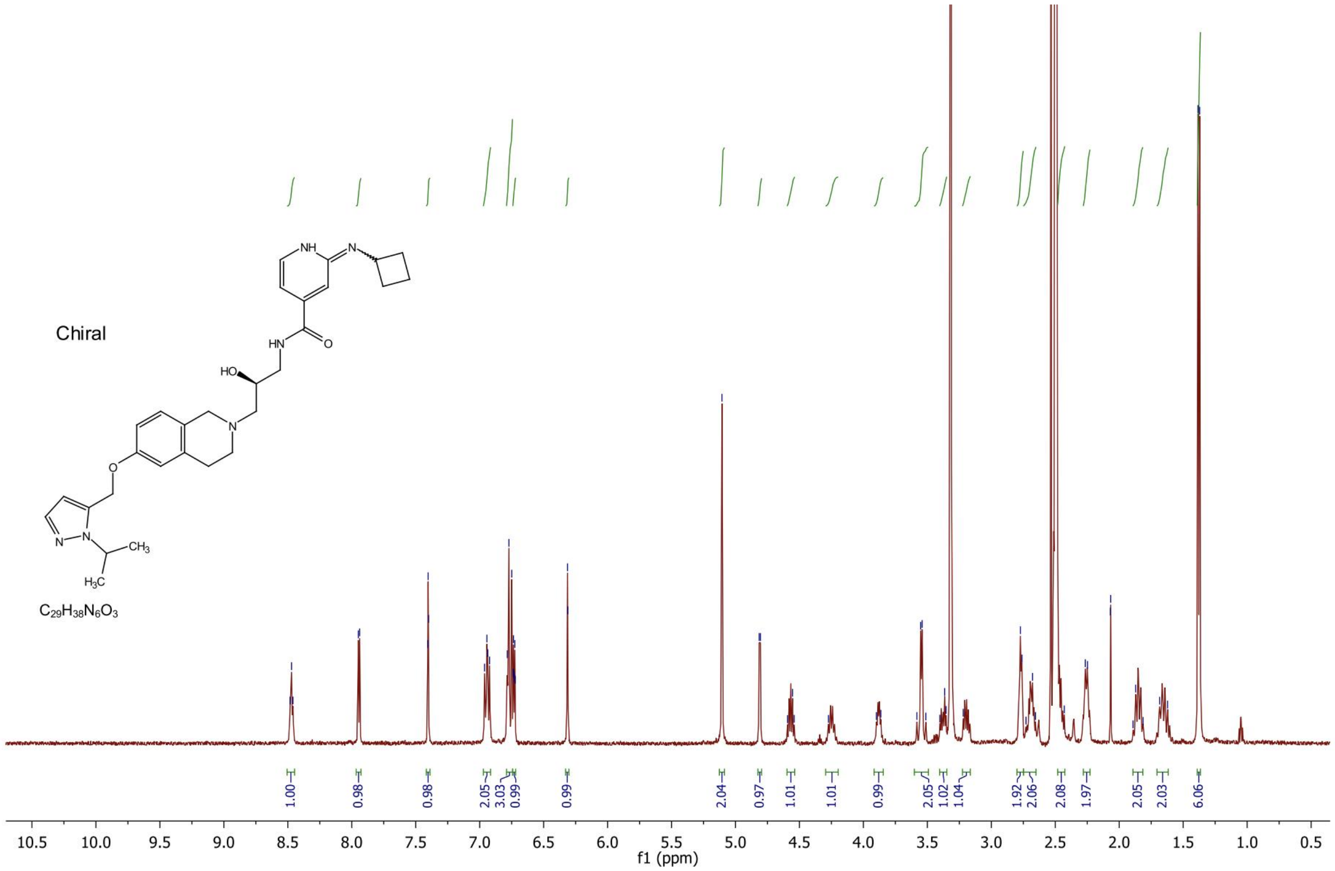

Chiral
NH
N
HN
O
HO
N
O
N
N
CH3
H3C
C29H38N6O3
1.00
0.98
0.98
2.05
3.03
0.99
0.99
2.04
0.97
1.01
1.01
0.99
2.05
1.02
1.04
1.92
2.06
2.08
1.97
2.05
2.03
6.06
10.5
10.0
9.5
9.0
8.5
8.0
7.5
7.0
6.5
6.0
5.5
5.0
4.5
4.0
3.5
3.0
2.5
2.0
1.5
1.0
0.5
f1 (ppm)

MaxPeak: 98.47%
Ret_Time: 0.933 min

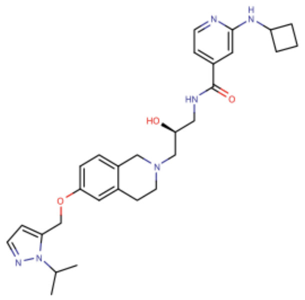

Mol Wt 518.65
Exact Mass 518.35

#	Time	Area%
1	0.933	98.47
2	1.166	1.53

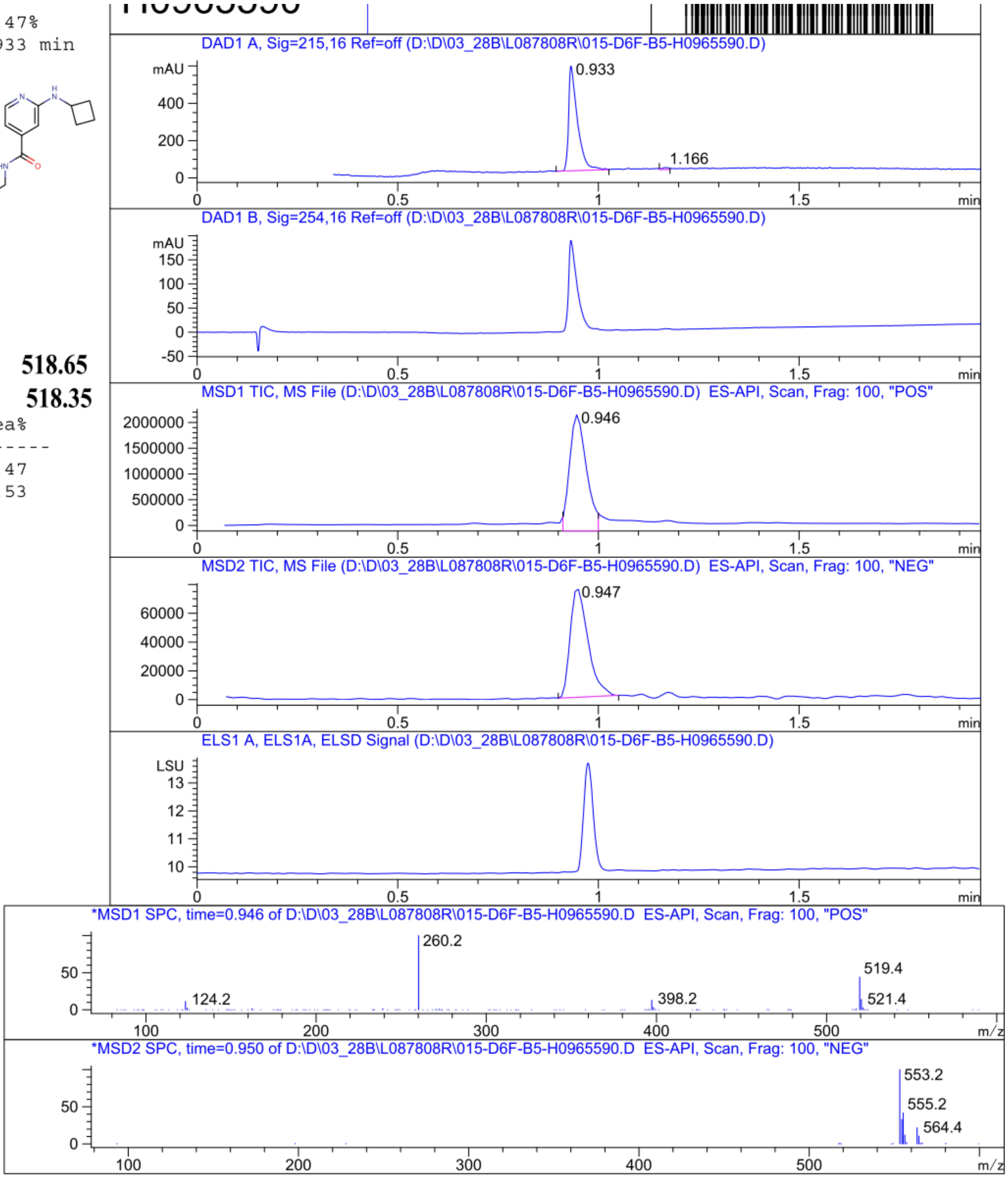

Compound 36

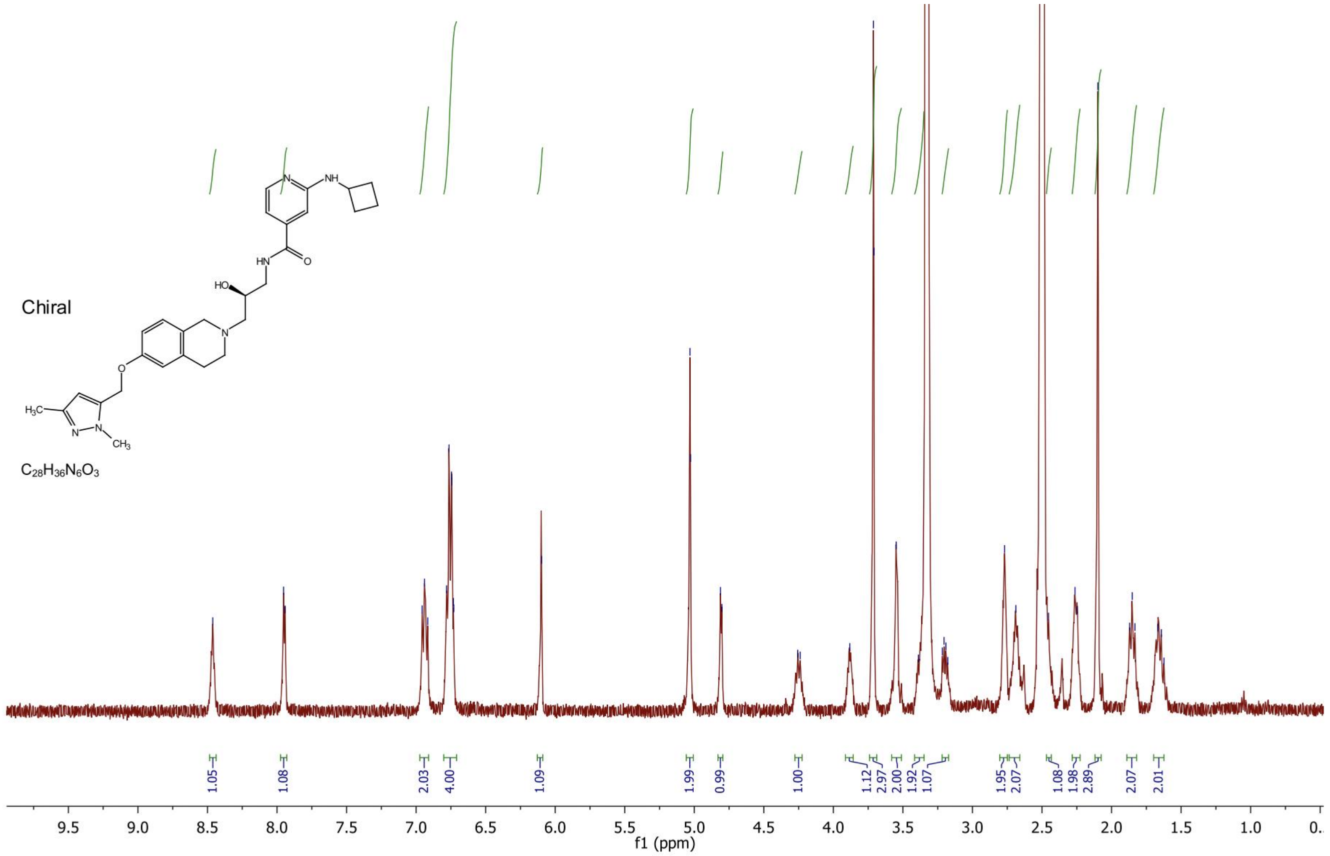

Chiral
HO
HN
O
N
NH
O
H3C
N
N
CH3
C28H36N6O3
1.05
1.08
2.03
4.00
1.09
1.99
0.99
1.00
1.12
2.97
2.00
1.92
1.07
1.95
2.07
1.08
1.98
2.89
2.07
2.01
9.5
9.0
8.5
8.0
7.5
7.0
6.5
6.0
5.5
5.0
4.5
4.0
3.5
3.0
2.5
2.0
1.5
1.0
f1 (ppm)

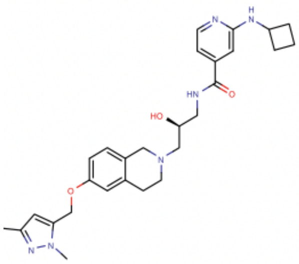

Mol Wt 504.62
Exact Mass 504.33

#	Time	Area%
1	0.866	100.00

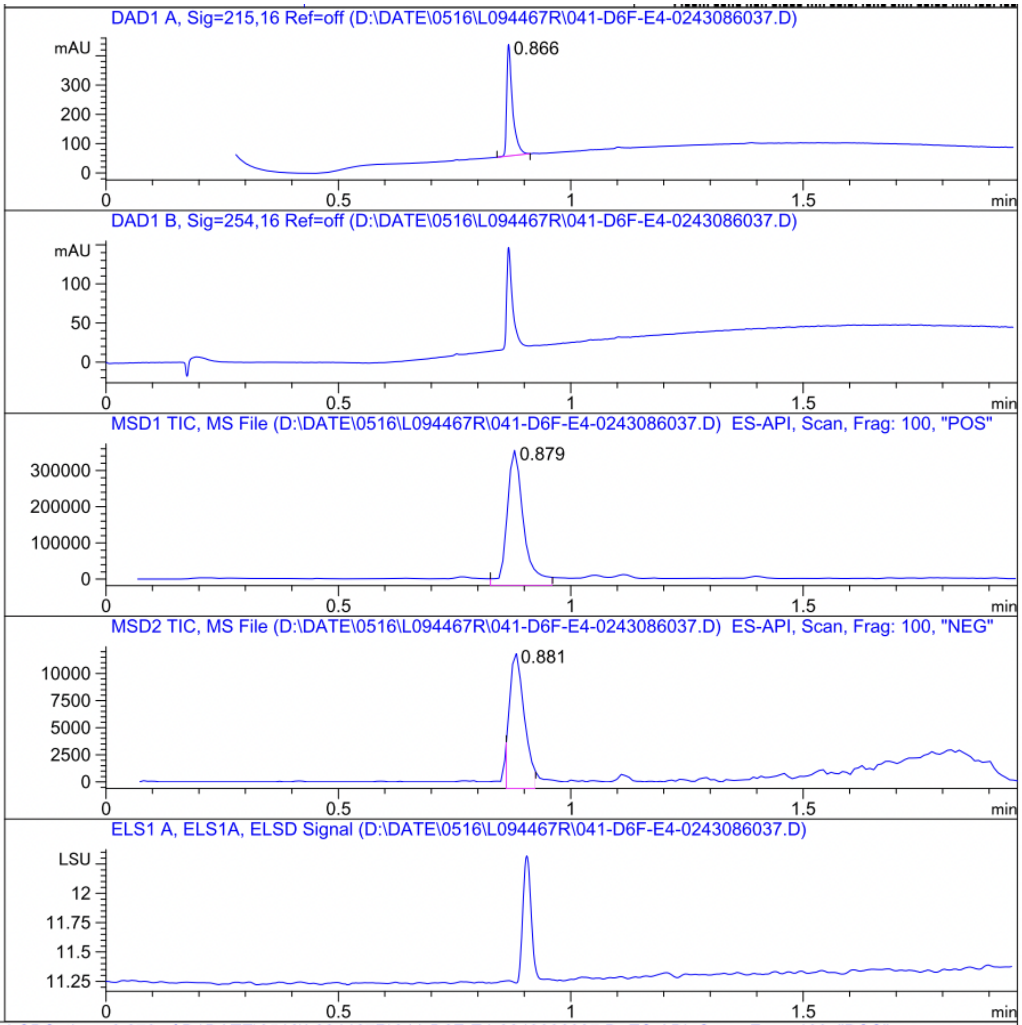

RT 0.879

RT 0.881

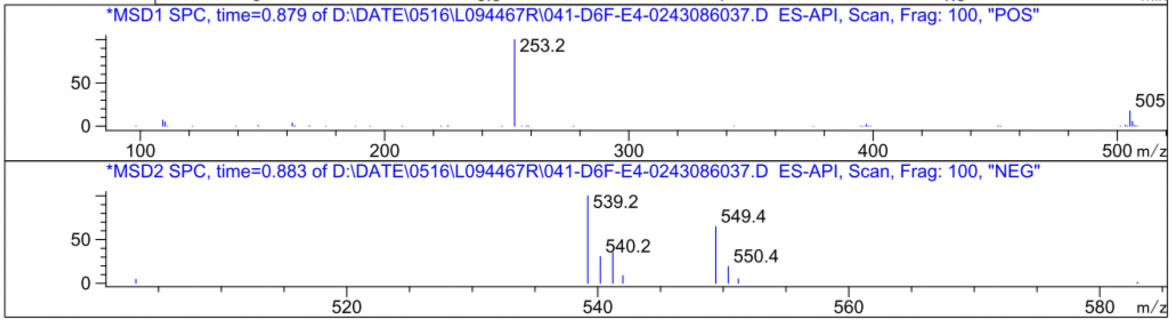

Compound 37

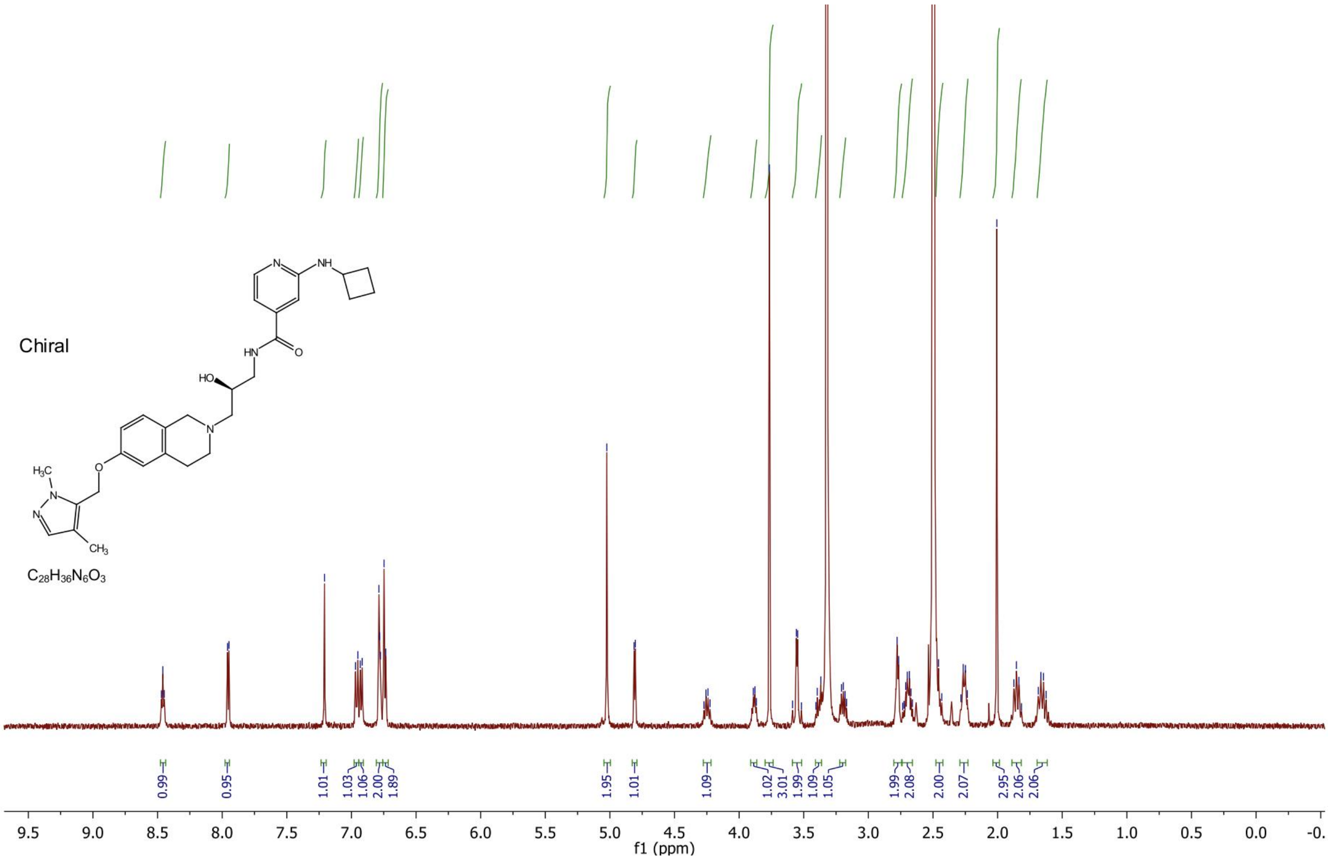

Chiral
$C_{28}H_{36}N_6O_3$
0.99
0.95
1.01
1.03
1.06
2.00
1.89
1.95
1.01
1.09
1.02
3.01
1.99
1.09
1.05
1.99
2.08
2.00
2.07
2.95
2.06
2.06
9.5 9.0 8.5 8.0 7.5 7.0 6.5 6.0 5.5 5.0 4.5 4.0 3.5 3.0 2.5 2.0 1.5 1.0 0.5 0.0 -0.
f1 (ppm)

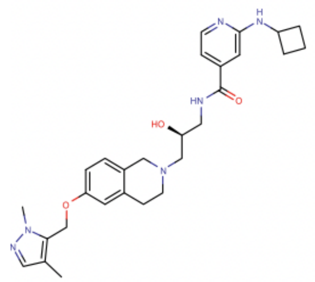

Mol Wt 504.62

Exact Mass 504.33

```
#  Time   Area%
-----------------
1  0.869  100.00
```

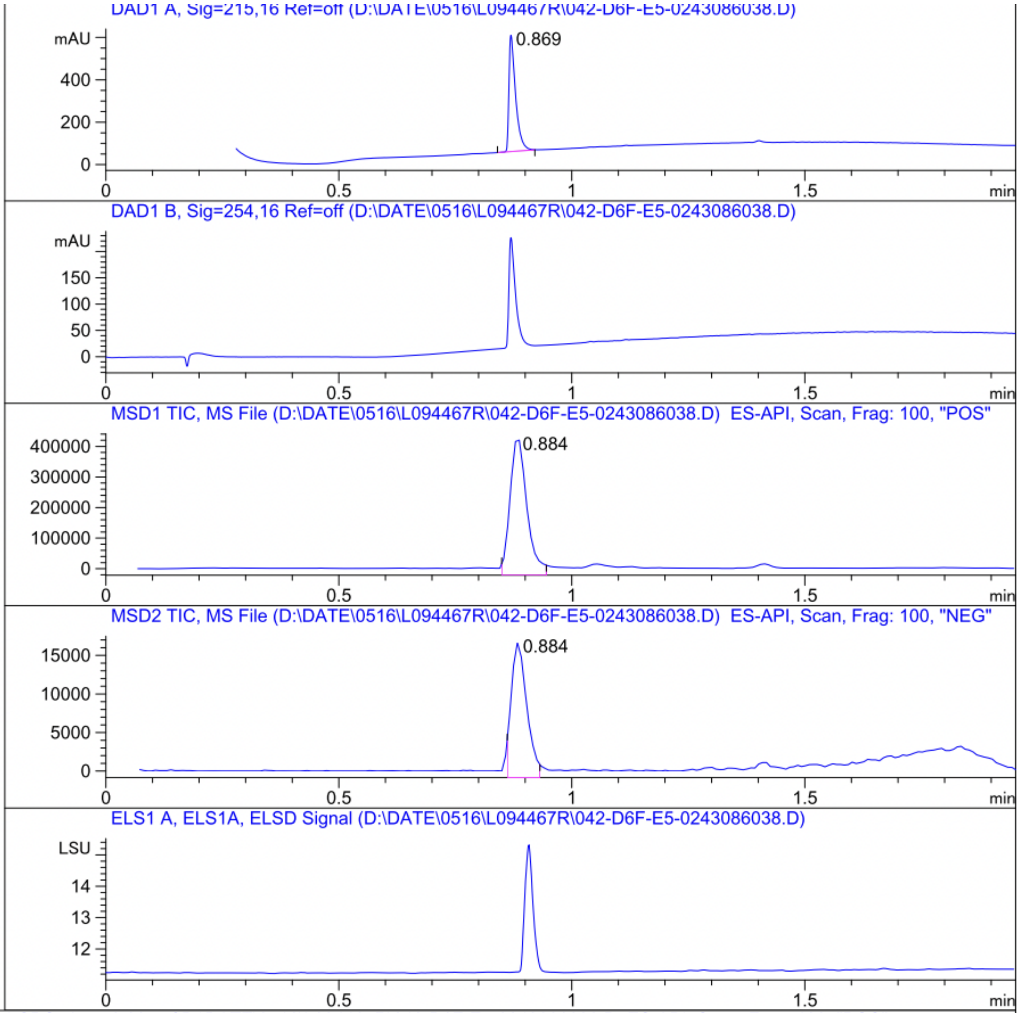

RT 0.884

RT 0.884

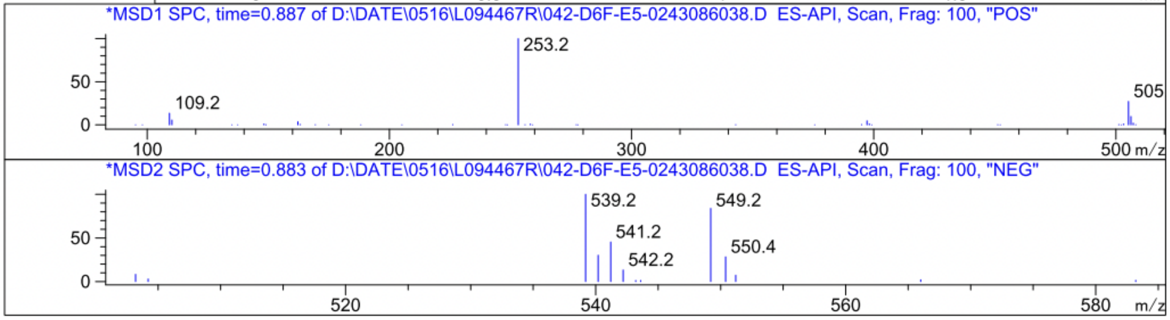

Compound 38

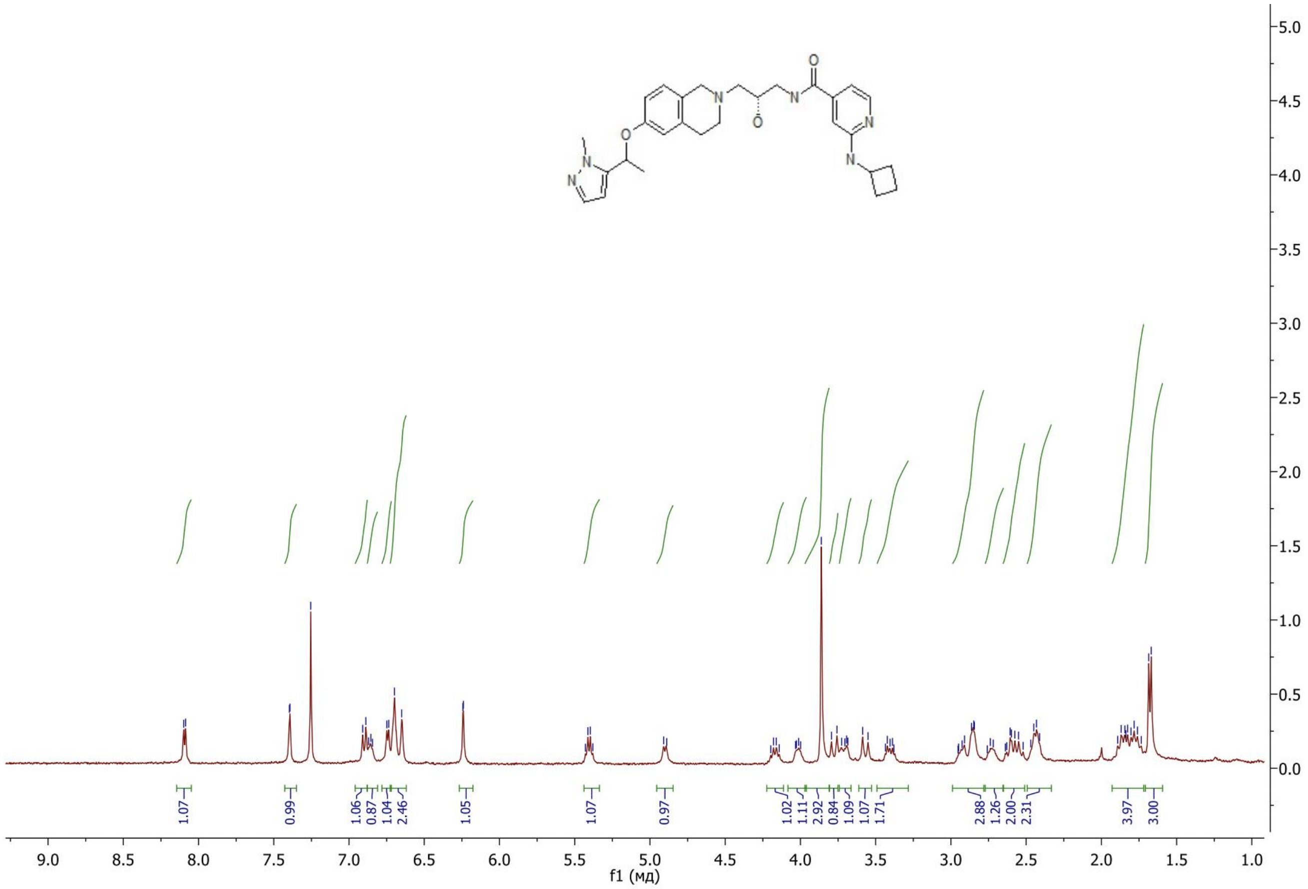
f1 (мд)
1.07
0.99
1.06
0.87
1.04
2.46
1.05
1.07
0.97
1.02
1.11
2.92
0.84
1.09
1.07
1.71
2.88
1.26
2.00
2.31
3.97
3.00

MaxPeak: 98.85%
Ret_Time: 0.824 min

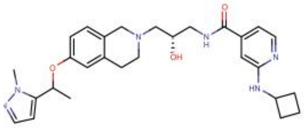

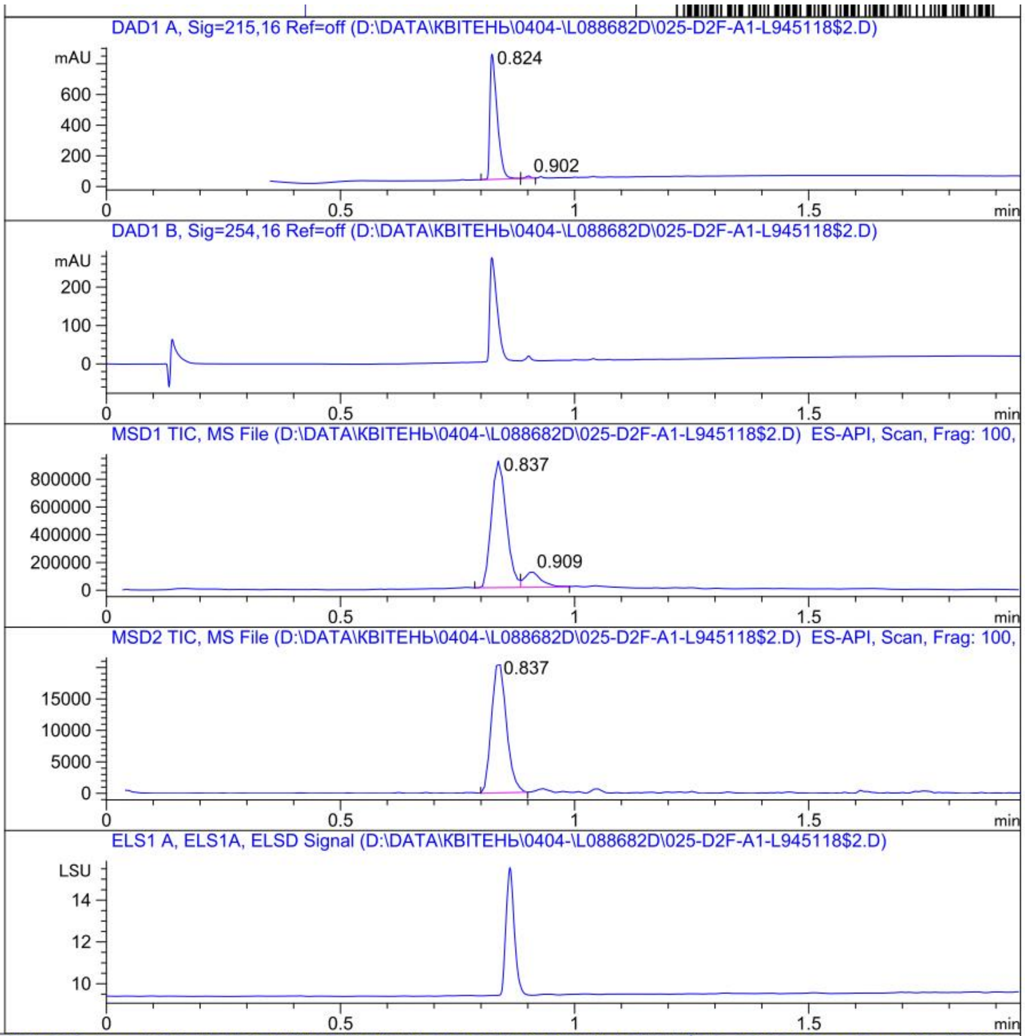

Mol Wt 504.62

Exact Mass 504.33

#	Time	Area%
1	0.824	98.85
2	0.902	1.15

RT 0.837

RT 0.909

RT 0.837

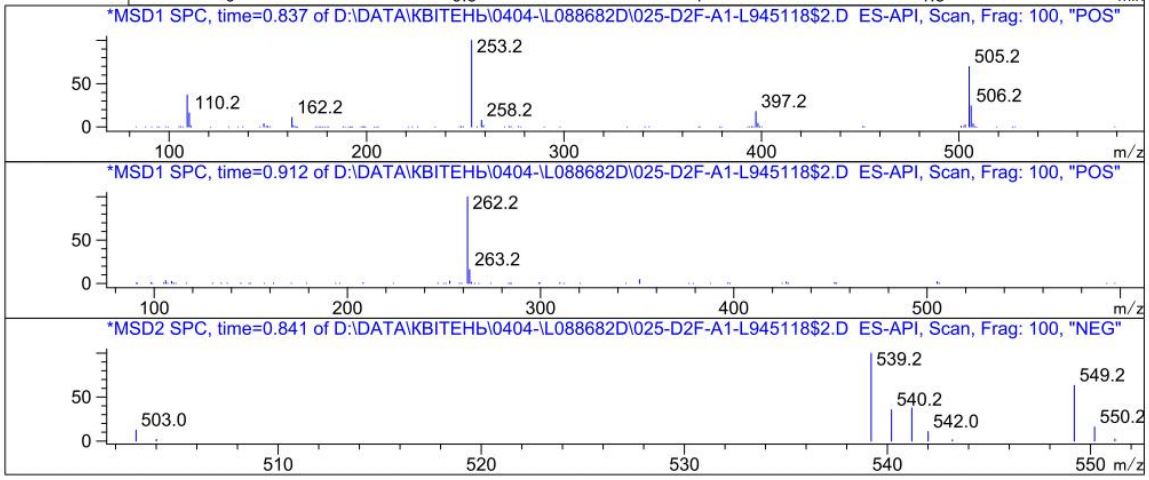

Compound 39

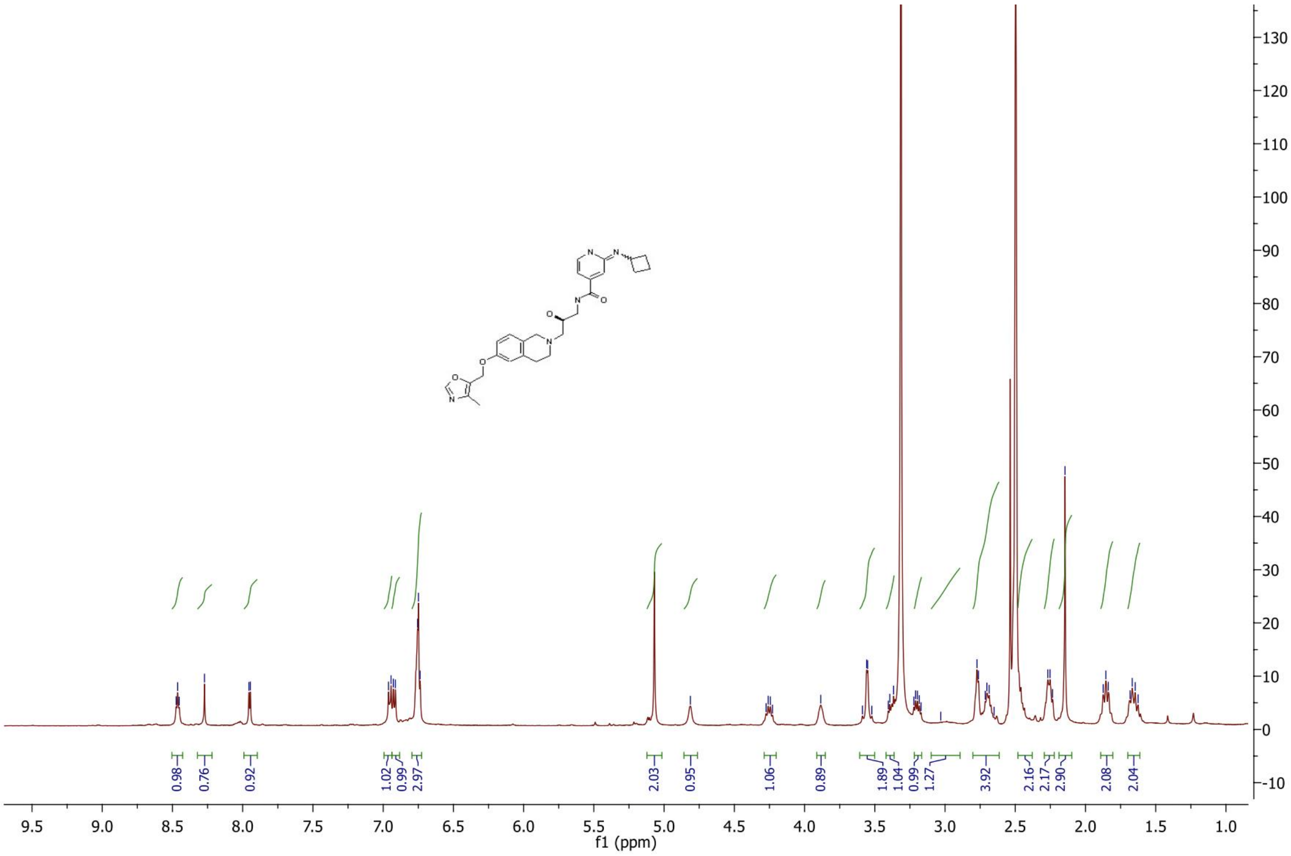
0.98
0.76
0.92
1.02
0.99
2.97
2.03
0.95
1.06
0.89
1.89
1.04
0.99
1.27
3.92
2.16
2.17
2.90
2.08
2.04
f1 (ppm)

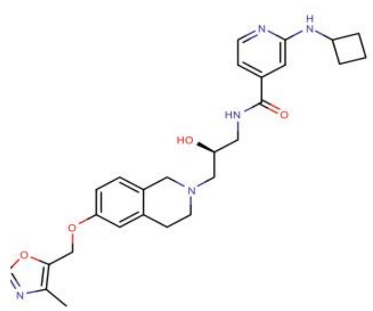

Mol Wt **491.58**

Exact Mass **491.29**

#	Time	Area%
1	0.725	95.80
2	0.752	2.51
3	0.948	1.69

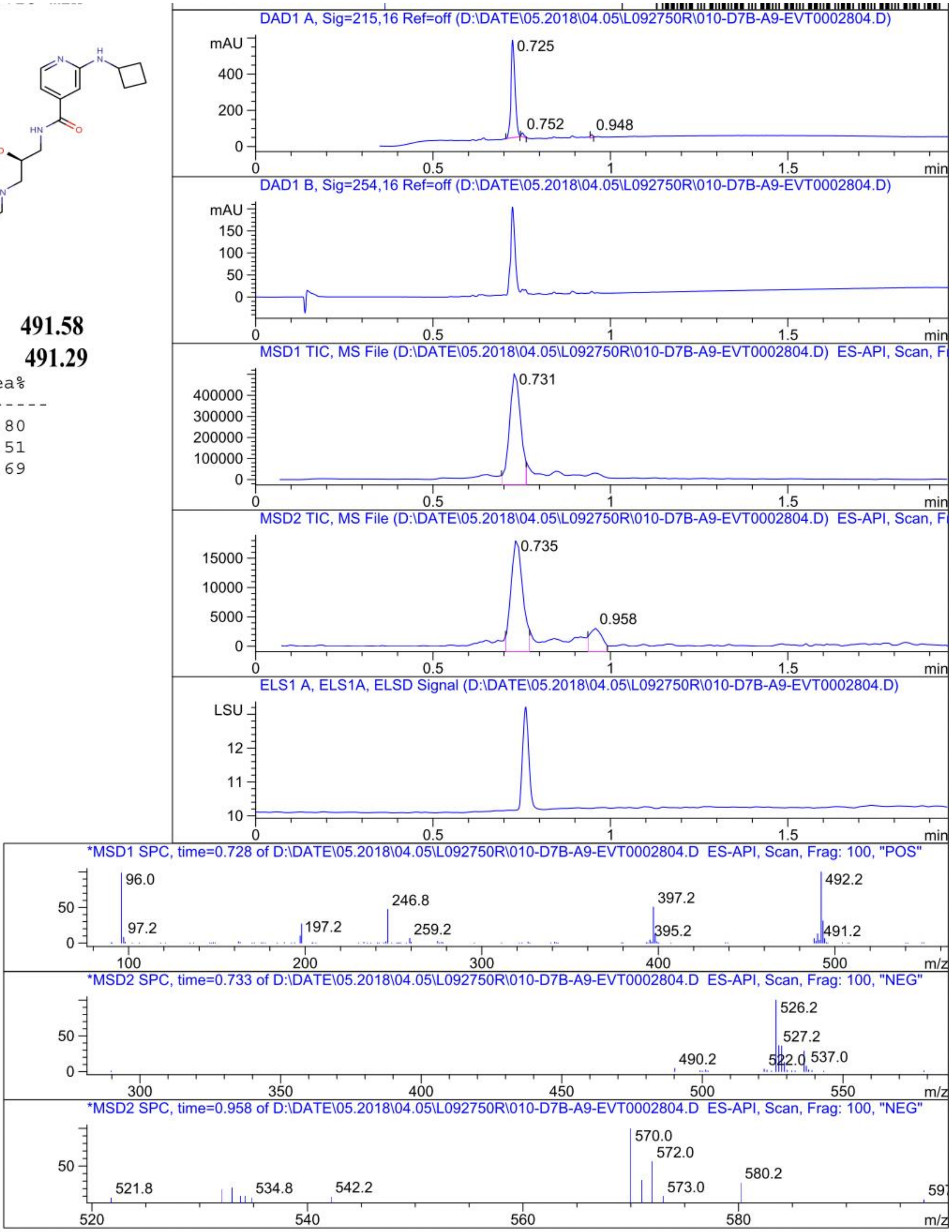

RT 0.731

RT 0.735

RT 0.958

Compound 40

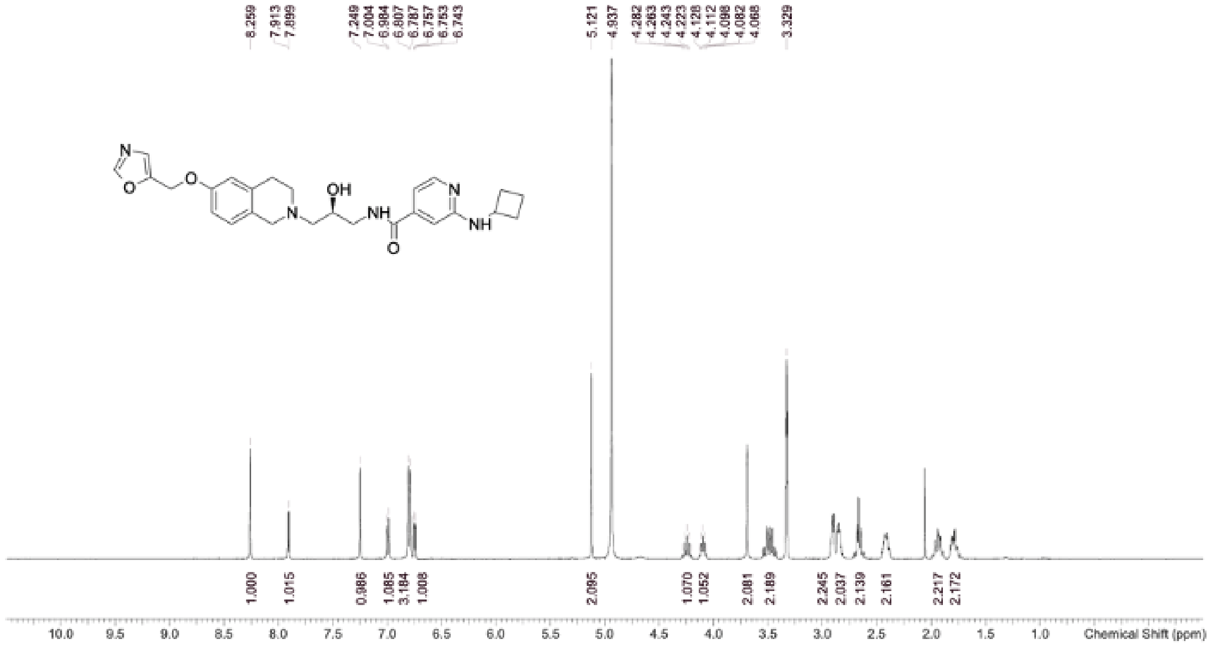

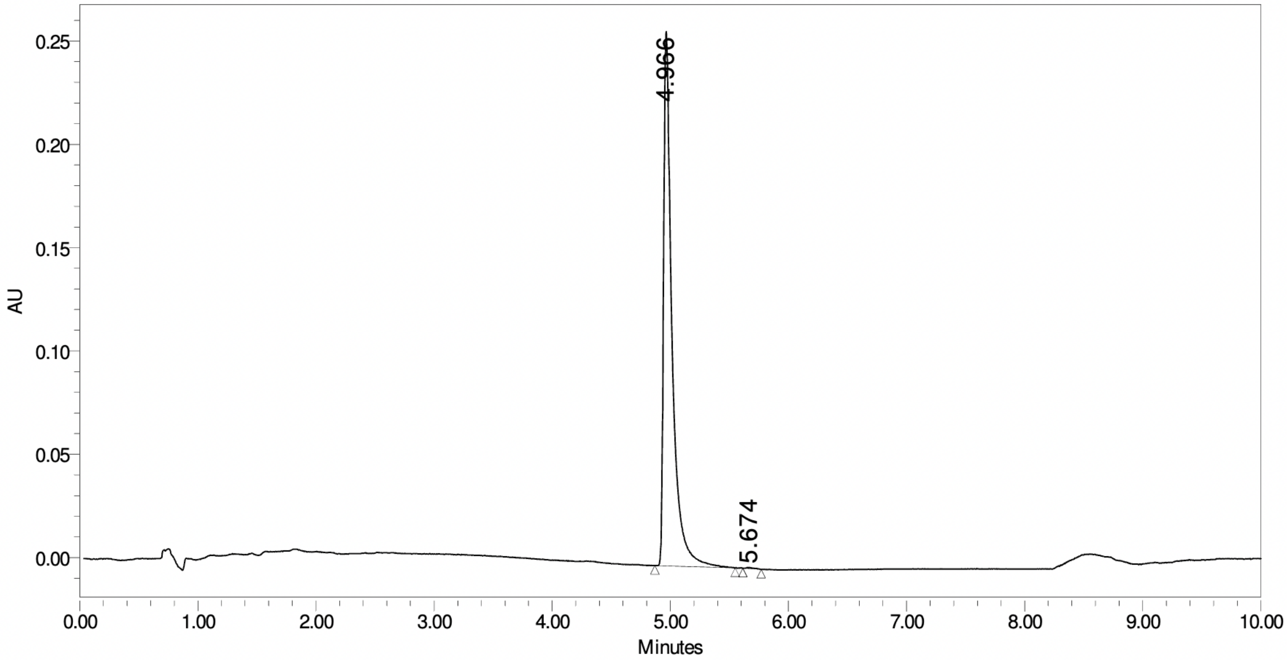

	RT	Area	% Area
1	4.966	1280805	99.85
2	5.674	1885	0.15

Instrument Method: OJ_3_MeOH_DEA_5_40_25ML

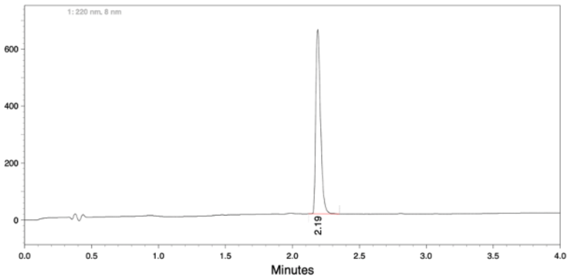

1: 220 nm, 8 nm

Retention Time	*Height*	*Area*	*Area Percent*
2.19	641881	1615324	100.00

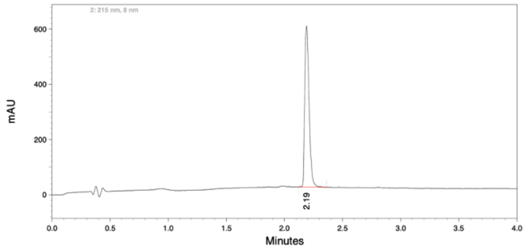

2: 215 nm, 8 nm

Retention Time	*Height*	*Area*	*Area Percent*
2.19	580332	1518970	100.00

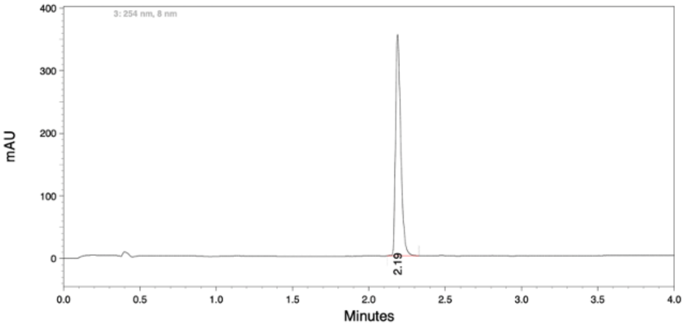

3: 254 nm, 8 nm

Retention Time	*Height*	*Area*	*Area Percent*
2.19	350136	839385	100.00

```
Instrument        : LCMS AR
                    A:,Xtimate,2.1*30mm,3um
                    B:XBridge Shield,  2.1*50mm,5um
                    Confidential. For research only  NOT for regulatory fili
```

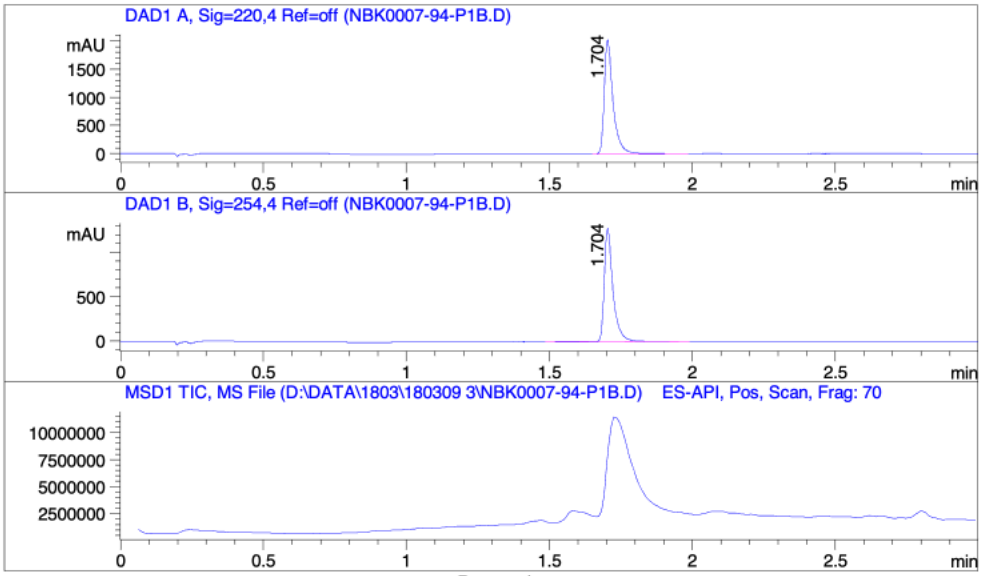


```
                              Report
==================================================================
   Signal ->: DAD1 A, Sig=220,4 Ref=off
    #  Meas. Ret.   Height   Width        Area       Area %
   --------------------------------------------------------
     1      1.704   2008.634   0.032        4330.393 100.000
   --------------------------------------------------------


   Signal ->: DAD1 B, Sig=254,4 Ref=off
    #  Meas. Ret.   Height   Width        Area       Area %
   --------------------------------------------------------
     1      1.704   1268.110   0.032        2726.039 100.000
   --------------------------------------------------------
```

Compound 41

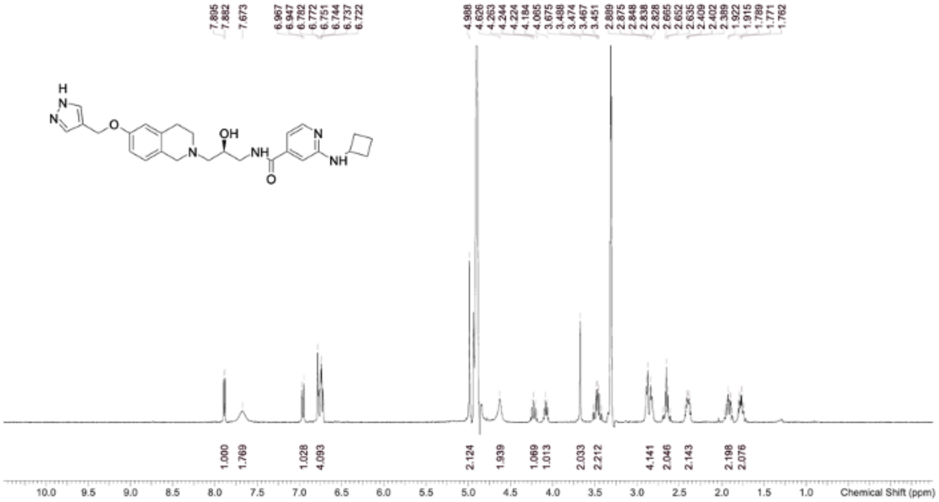

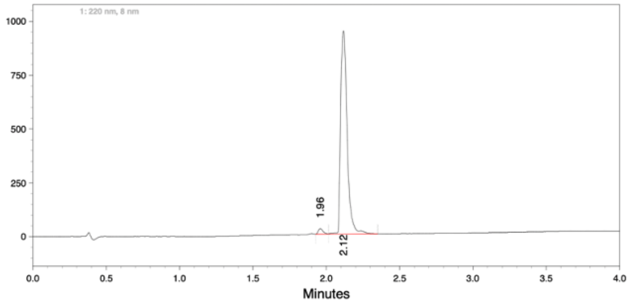

1: 220 nm, 8 nm

Retention Time	Height	Area	Area Percent
1.96	25158	57534	1.94
2.12	940692	2914236	98.06

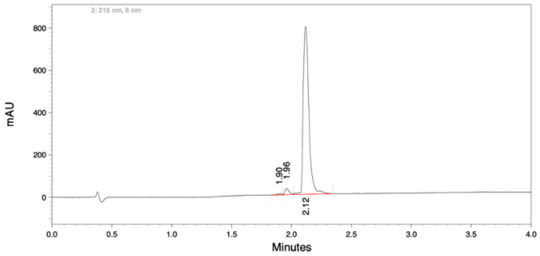

2: 215 nm, 8 nm

Retention Time	Height	Area	Area Percent
1.90	4945	13851	0.52
1.96	28650	71741	2.68
2.12	790335	2590015	96.80

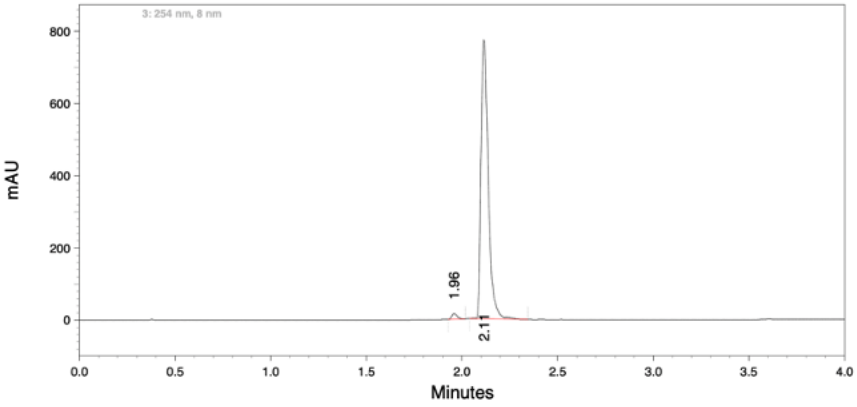

3: 254 nm, 8 nm

Retention Time	Height	Area	Area Percent
1.96	15219	32834	1.58
2.11	760911	2048339	98.42

```
Instrument         : LCMS AR
                     A:,Xtimate,2.1*30mm,3um
                     B:XBridge Shield,  2.1*50mm,5um
                     Confidential. For research only  NOT for regulatory filing
```

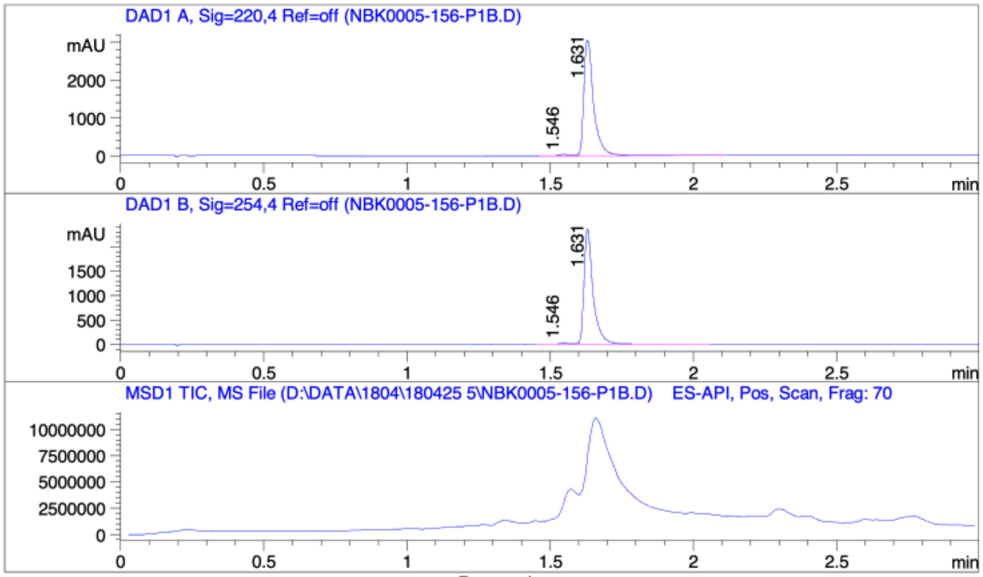


```
                          Report
=================================================================
   Signal ->: DAD1 A, Sig=220,4 Ref=off
    #  Meas. Ret.   Height    Width         Area       Area %
   ----------------------------------------------------------
     1      1.546    39.663   0.034         91.640    1.232
     2      1.631  3037.392   0.036       7346.099   98.768
   ----------------------------------------------------------

   Signal ->: DAD1 B, Sig=254,4 Ref=off
    #  Meas. Ret.   Height    Width         Area       Area %
   ----------------------------------------------------------
     1      1.546    32.950   0.034         76.701    1.427
     2      1.631  2356.907   0.033       5297.644   98.573
   ----------------------------------------------------------
```

Method :Column: Chiralpak AD-3 50*4.6mm I.D., 3um
Mobile phase: 40% of ethanol(0.1% ethanolamine) in CO2
Flow rate: 4mL/min
Column temp:40 C

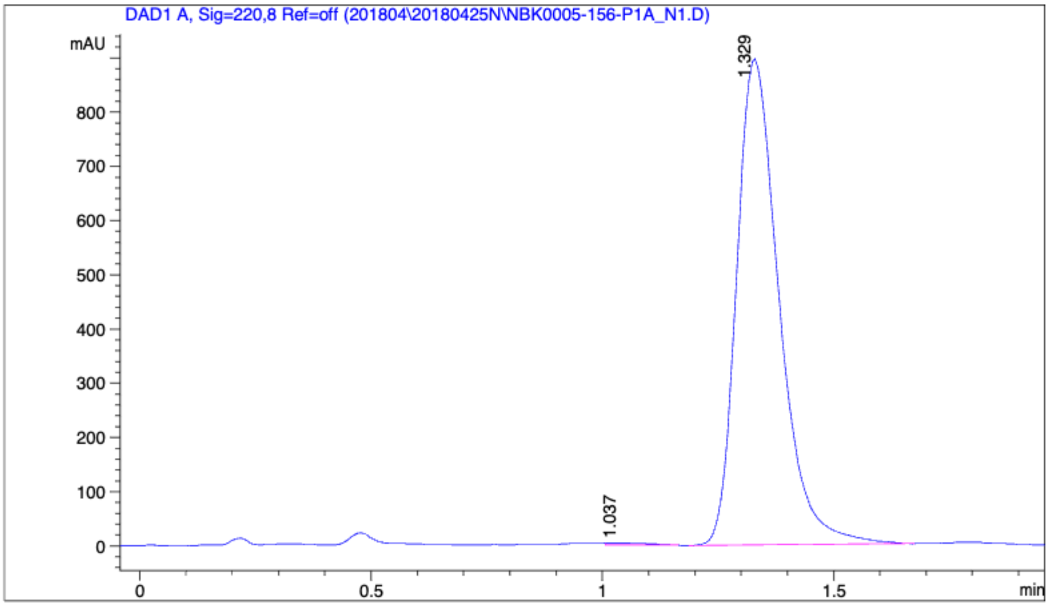

Signal 1 : DAD1 A, Sig=220,8 Ref=off

Peak	Meas. Ret. Time	Height	Height %	Width	Area	Area %
1	1.037	4.272	0.473	0.093	23.964	0.417
2	1.329	898.212	99.527	0.106	5718.140	99.583

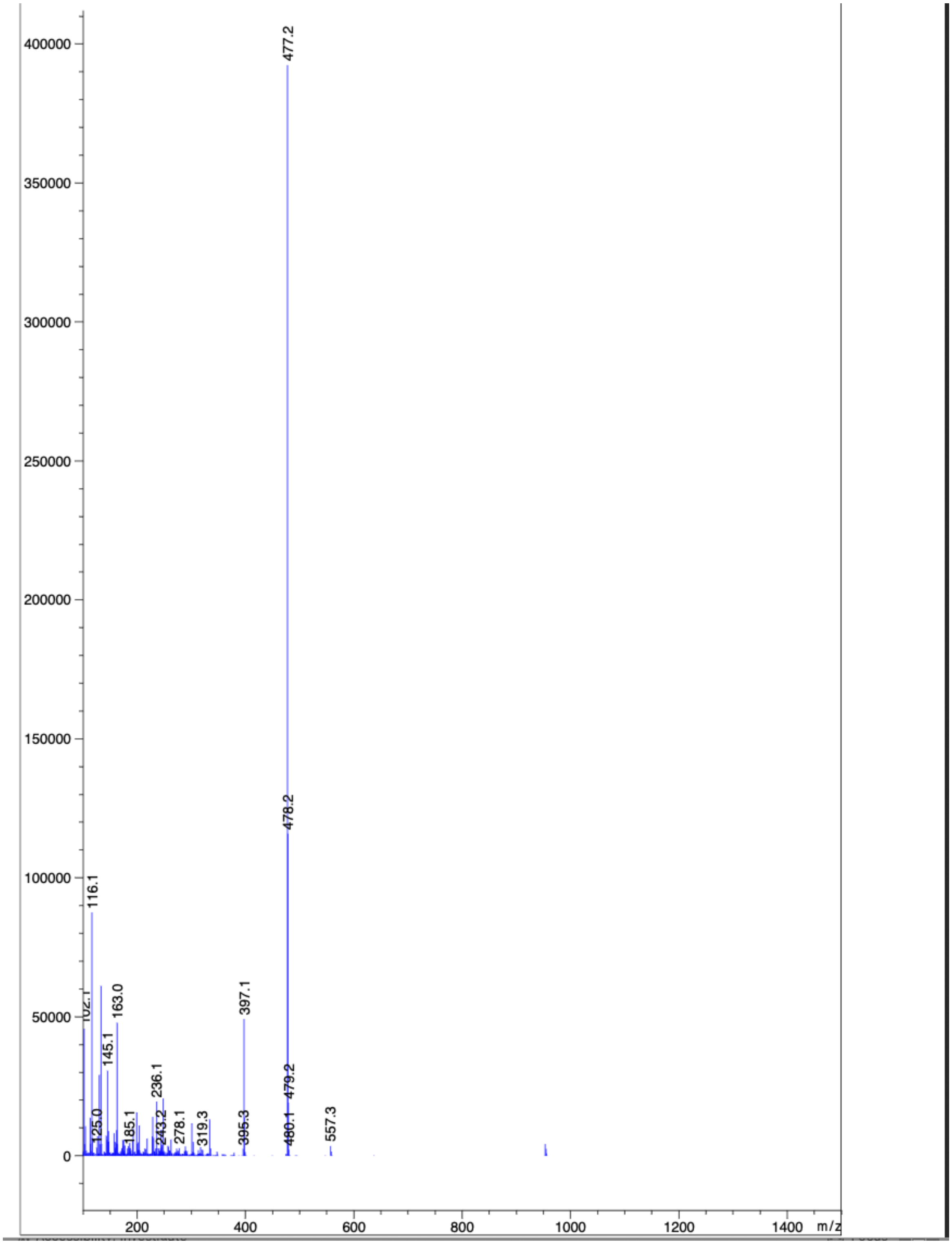

400000
350000
300000
250000
200000
150000
100000
50000
0
200
400
600
800
1000
1200
1400
m/z
477.2
478.2
116.1
163.0
397.1
145.1
236.1
479.2
185.1
125.0
243.2
278.1
319.3
395.3
480.1
557.3

Compound 42

MaxPeak: 95.20%
Ret_Time: 0.794 min

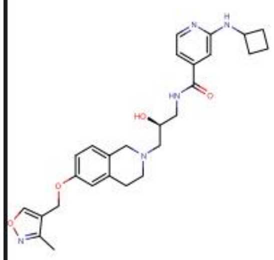

Mol Wt 491.58
Exact Mass 491.29

#	Time	Area%
1	0.692	4.80
2	0.794	95.20

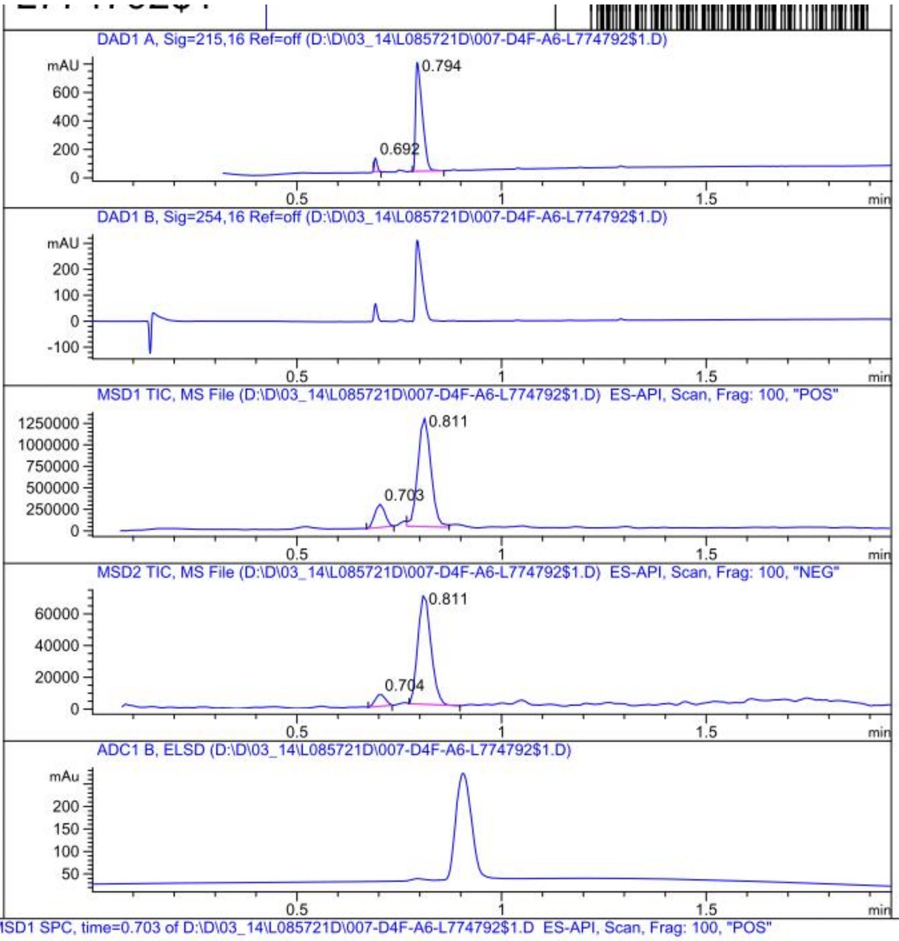

RT 0.703

RT 0.811

RT 0.704

RT 0.811

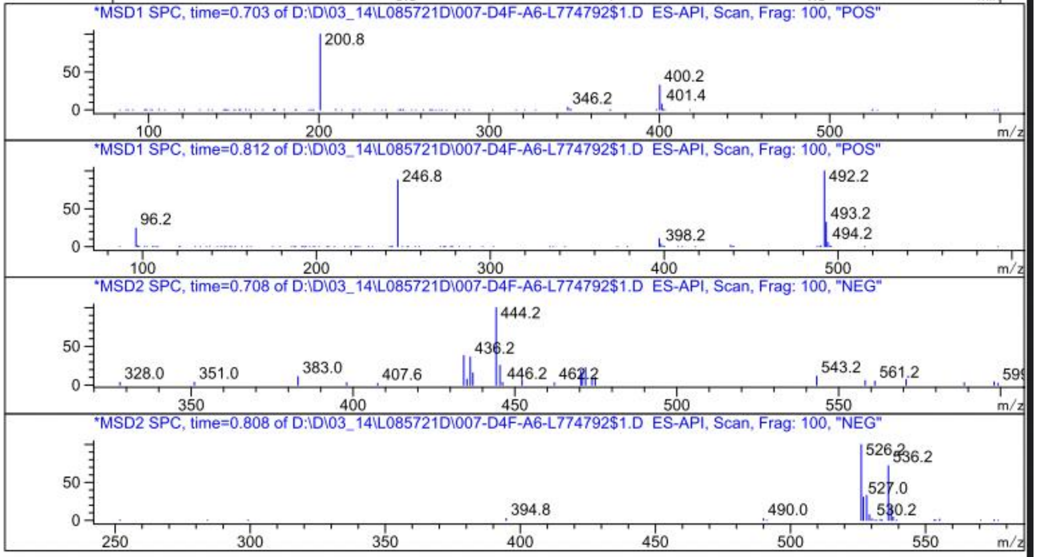

Compound 43

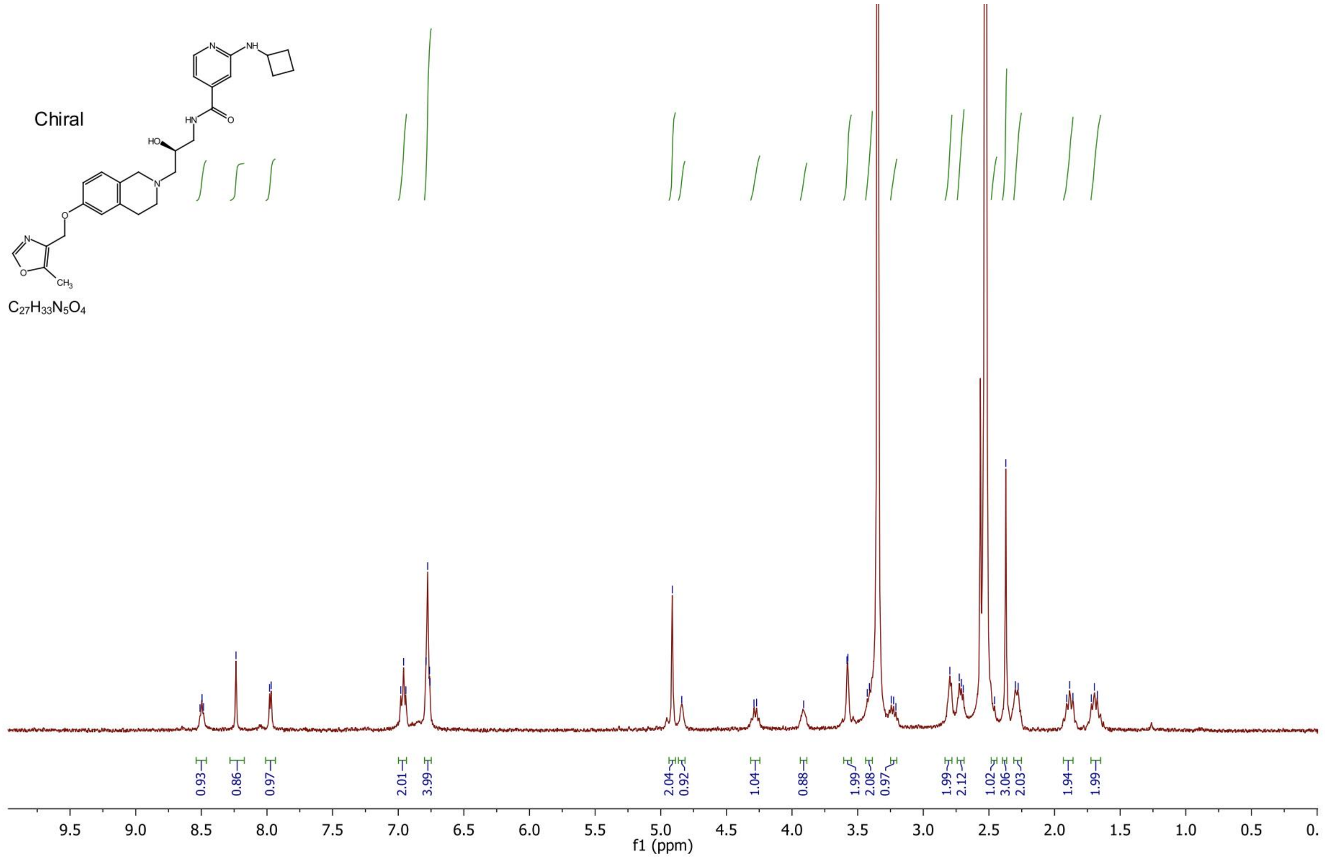
Chiral
$C_{27}H_{33}N_5O_4$
0.93
0.86
0.97
2.01
3.99
2.04
0.92
1.04
0.88
1.99
2.08
0.97
1.99
2.12
1.02
3.06
2.03
1.94
1.99
9.5 9.0 8.5 8.0 7.5 7.0 6.5 6.0 5.5 5.0 4.5 4.0 3.5 3.0 2.5 2.0 1.5 1.0 0.5 0.
f1 (ppm)

MaxPeak: 100.00%
Ret_Time: 0.843 min

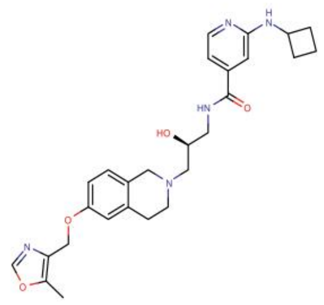

Mol Wt 491.58

Exact Mass 491.29

#	Time	Area%
1	0.843	100.00

RT 0.854

RT 0.855

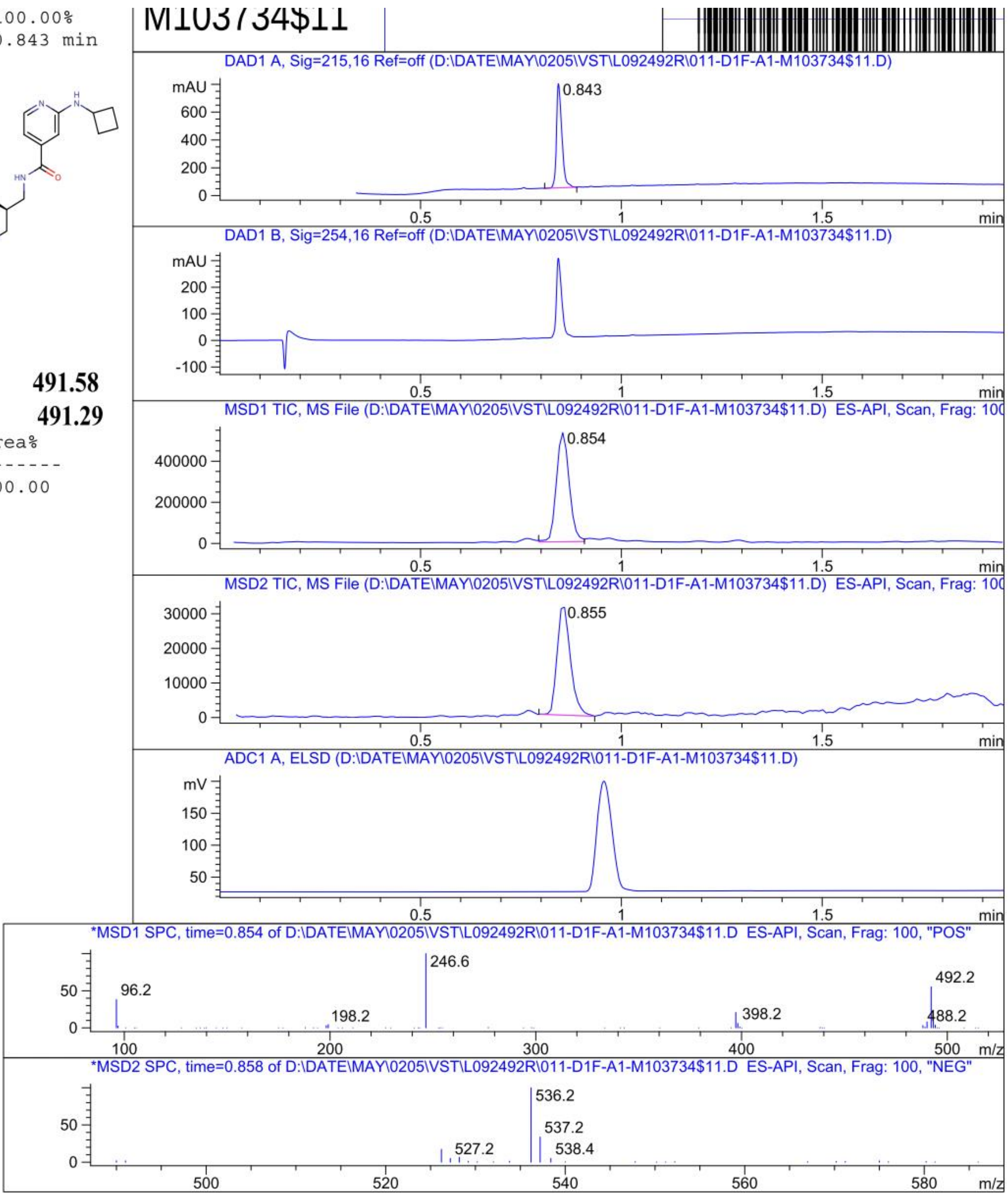

Compound 44

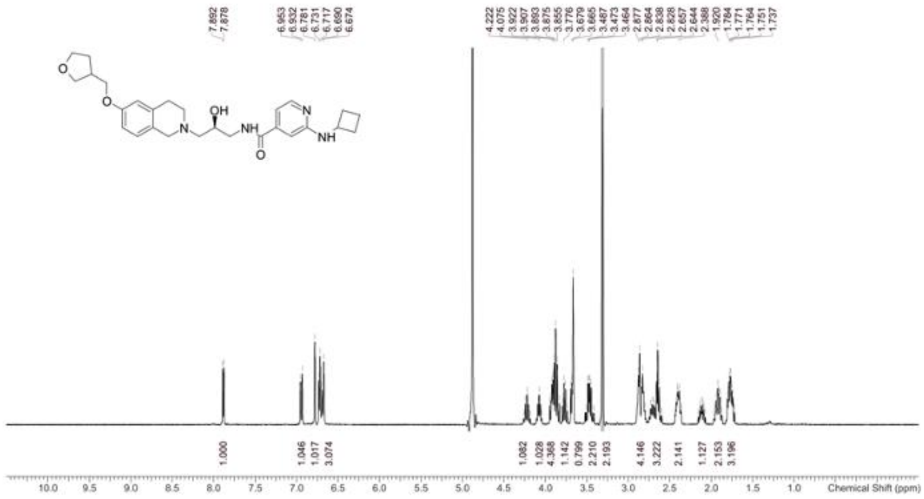

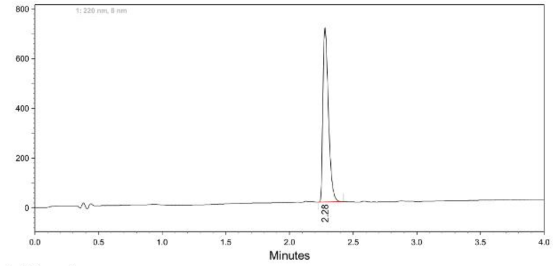

1: 220 nm, 8 nm

Retention Time	*Height*	*Area*	*Area Percent*
2.28	694344	2119713	100.00

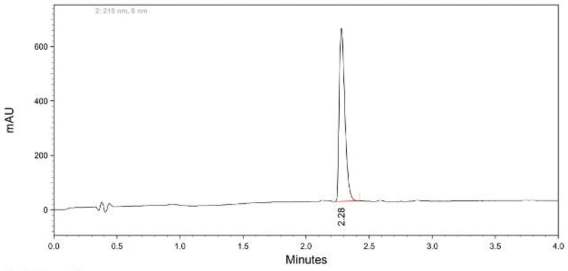

2: 215 nm, 8 nm

Retention Time	*Height*	*Area*	*Area Percent*
2.28	628885	1991351	100.00

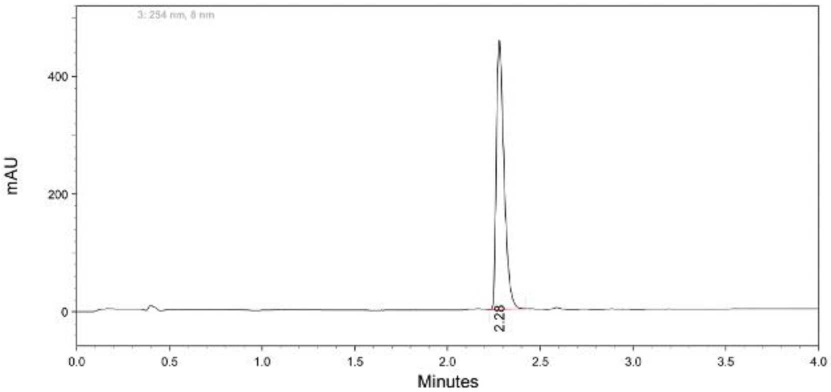

3: 254 nm, 8 nm

Retention Time	***Height***	***Area***	***Area Percent***
2.28	454737	1326680	100.00

Instrument : LCMS AR

A:,Xtimate,2.1*30mm,3um

B:XBridge Shield, 2.1*50mm,5um

Confidential. For research only NOT for regulatory filir

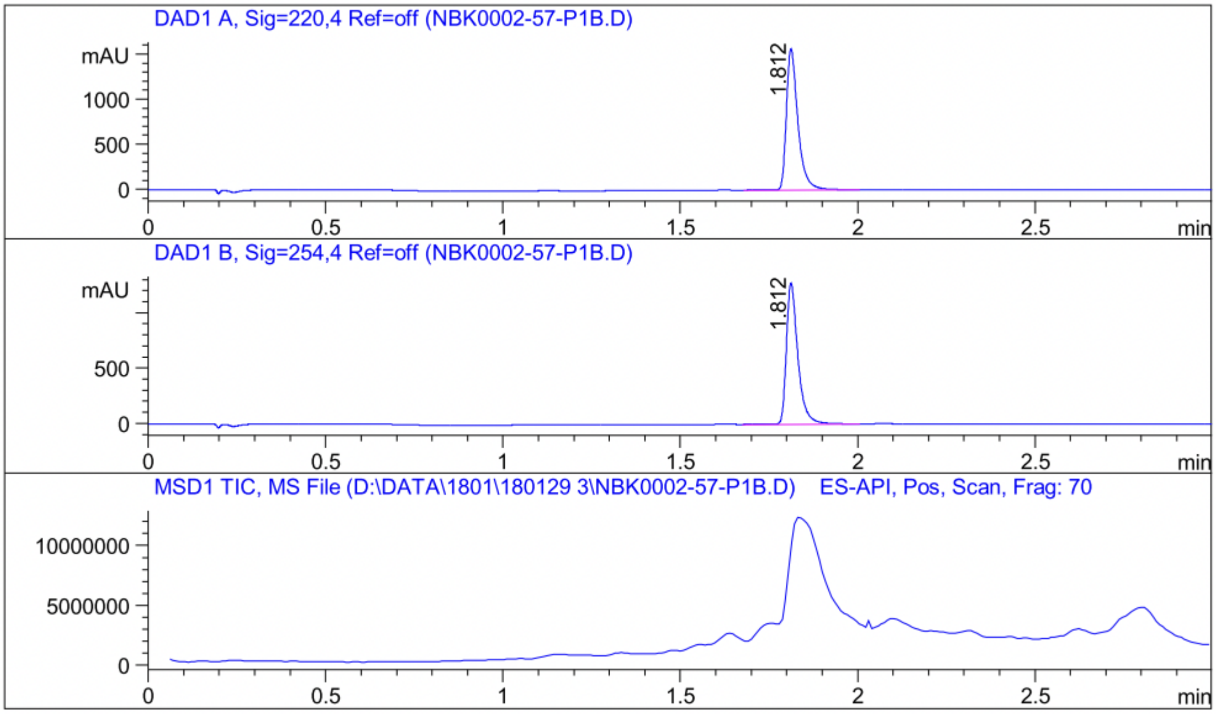

Report

==

Signal ->: DAD1 A, Sig=220,4 Ref=off

#	Meas. Ret.	Height	Width	Area	Area %
1	1.812	1563.669	0.035	3573.288	100.000

Signal ->: DAD1 B, Sig=254,4 Ref=off

#	Meas. Ret.	Height	Width	Area	Area %
1	1.812	1272.161	0.035	2893.496	100.000

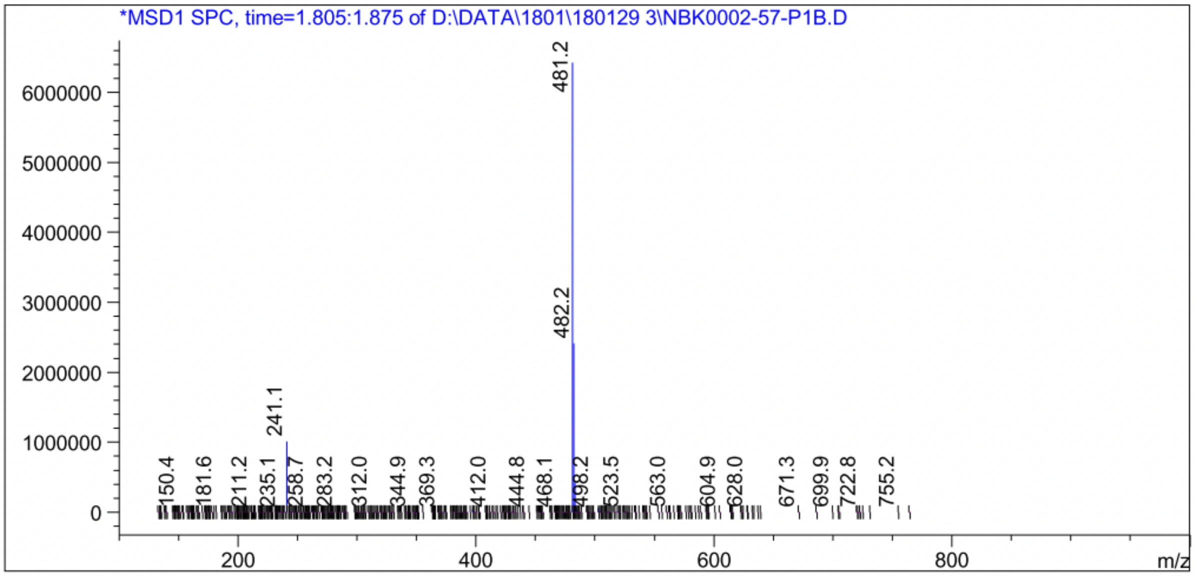
*MSD1 SPC, time=1.805:1.875 of D:\DATA\1801\180129 3\NBK0002-57-P1B.D
481.2
482.2
241.1
150.4
181.6
211.2
235.1
258.7
283.2
312.0
344.9
369.3
412.0
444.8
468.1
498.2
523.5
563.0
604.9
628.0
671.3
699.9
722.8
755.2
6000000
5000000
4000000
3000000
2000000
1000000
0
200
400
600
800
m/z

```
Method              :Column: Chiralpak AD-3 50*4.6mm I.D., 3um
                     Mobile phase: 40% of ethanol(0.05% DEA) in CO2
                     Flow rate: 4mL/min
                     Column temp:40 C
```

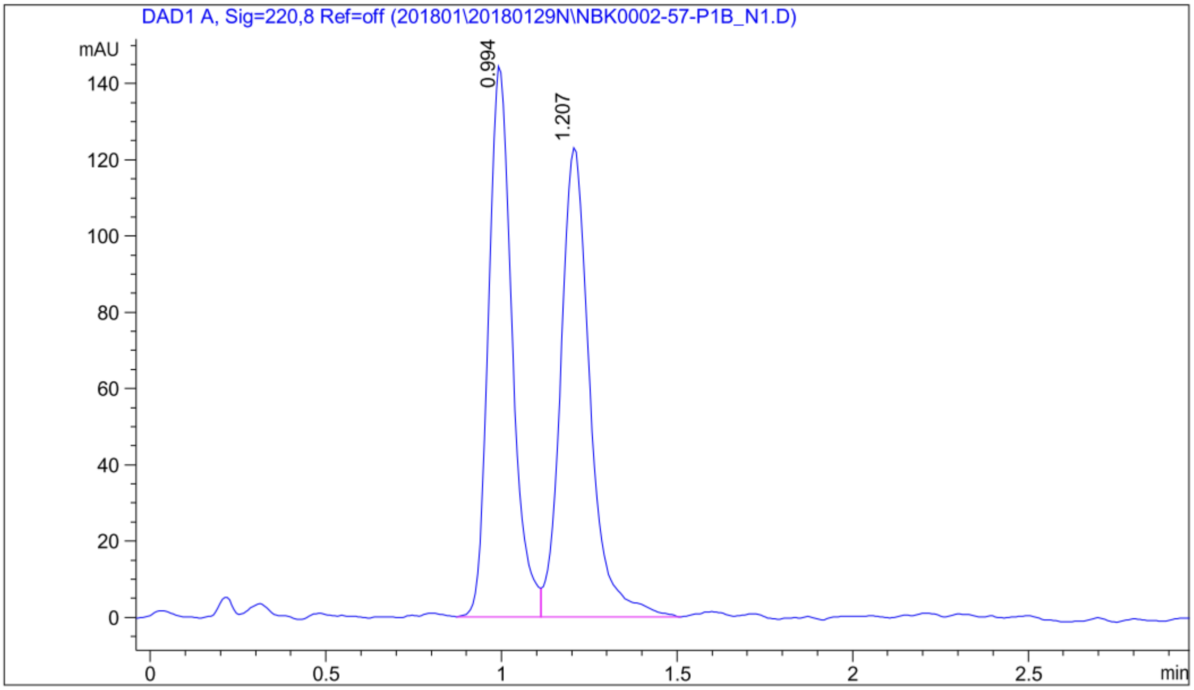

Signal 1 : DAD1 A, Sig=220,8 Ref=off

Peak	Meas. Ret. Time	Height	Height %	Width	Area	Area %
1	0.994	144.629	54.003	0.073	677.166	48.718
2	1.207	123.187	45.997	0.088	712.817	51.282

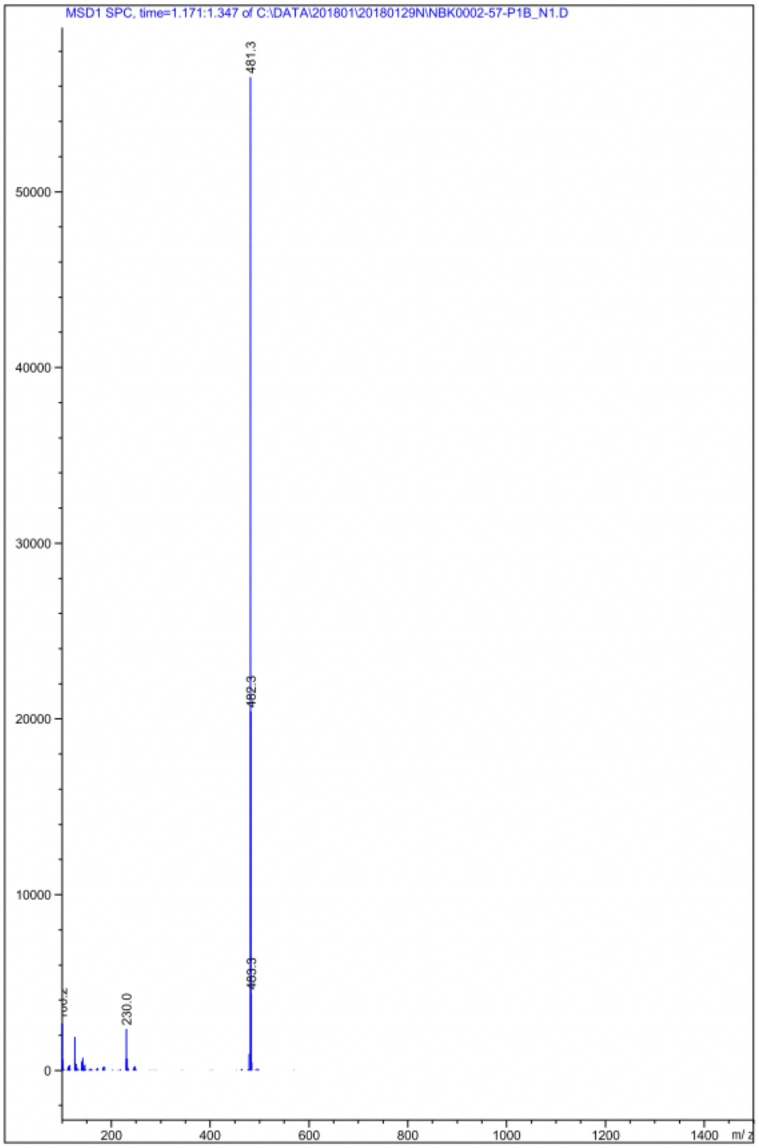

MSD1 SPC, time=1.171:1.347 of C:\DATA\201801\20180129N\NBK0002-57-P1B_N1.D
481.3
482.3
483.3
230.0
50000
40000
30000
20000
10000
0
200
400
600
800
1000
1200
1400
m/z

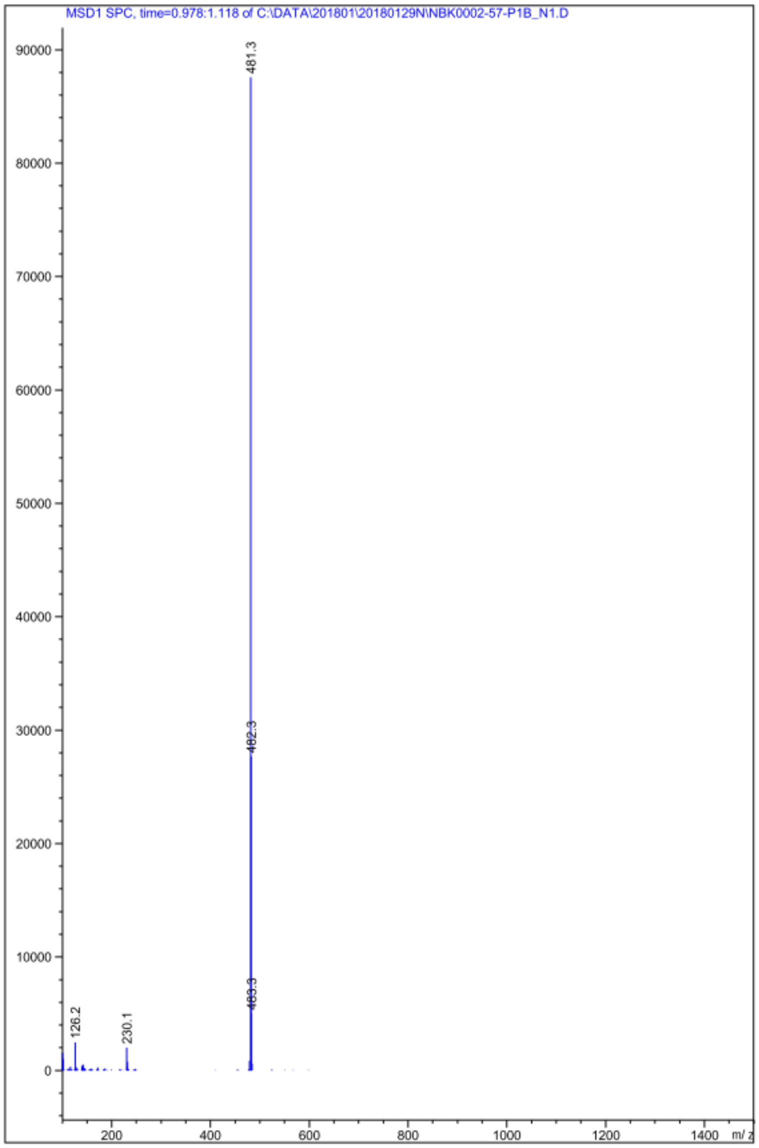
MSD1 SPC, time=0.978:1.118 of C:\DATA\201801\20180129N\NBK0002-57-P1B_N1.D
481.3
482.3
483.3
126.2
230.1
m/z

Compound 45

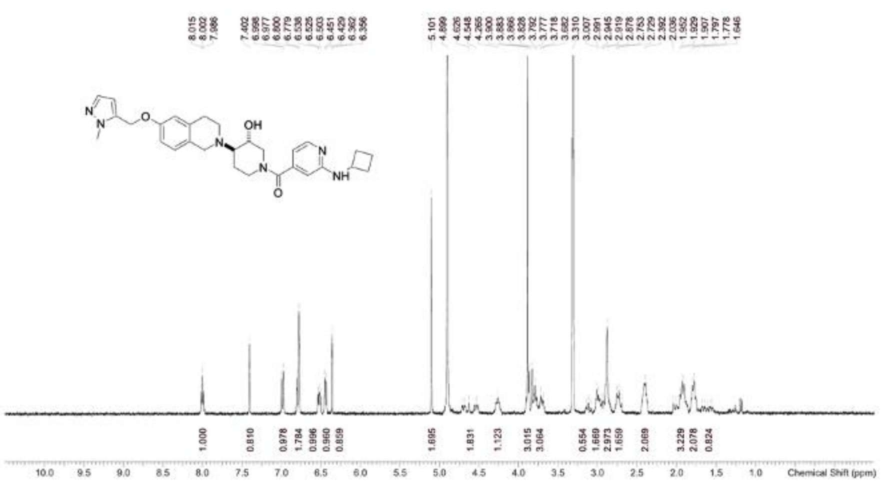

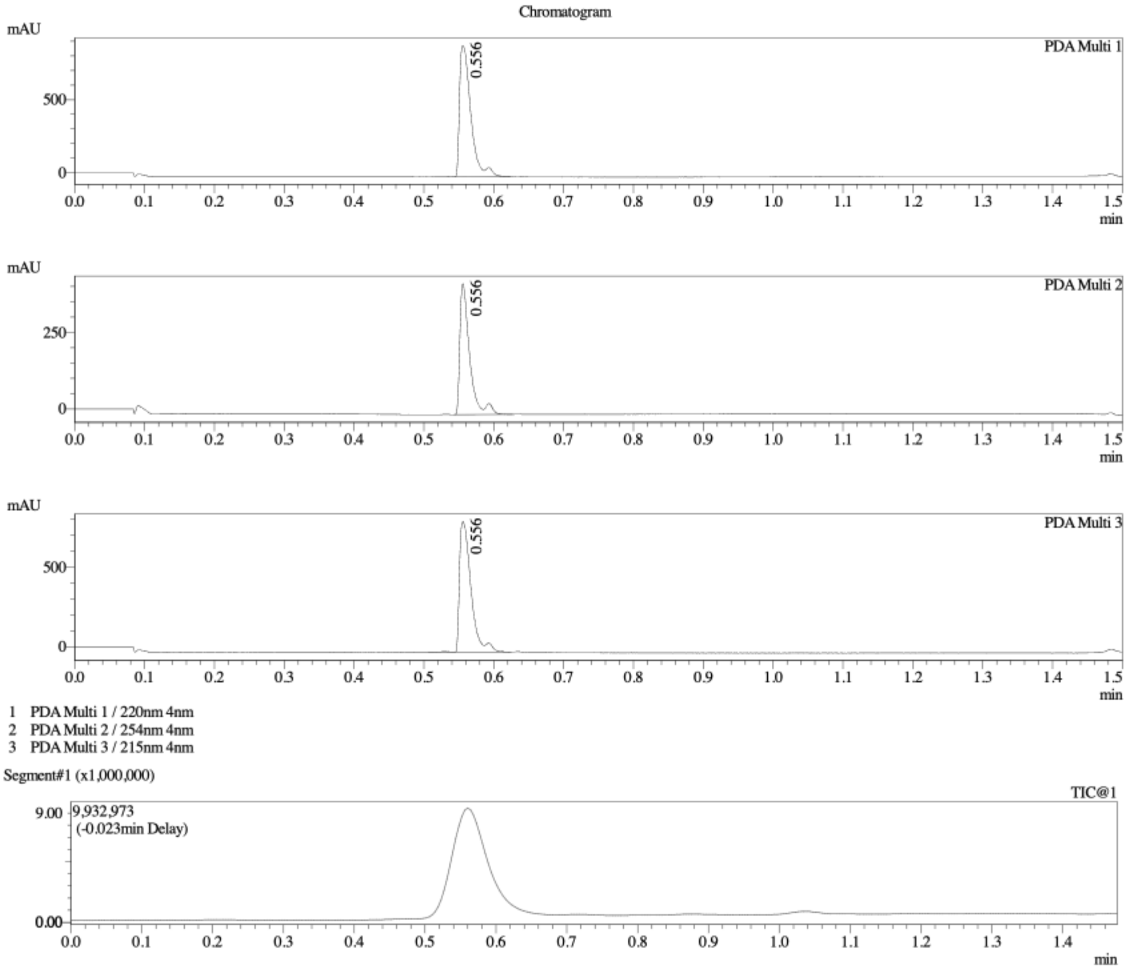

Integration Result

PDA Ch1 220nm 4nm

Peak#	Ret. Time	Height	Height %	USP Width	Area	Area %
1	0.556	886486	100.000	0.027	1006938	100.000

PDA Ch2 254nm 4nm

Peak#	Ret. Time	Height	Height %	USP Width	Area	Area %
1	0.556	416113	100.000	0.024	417140	100.000

PDA Ch3 215nm 4nm

Peak#	Ret. Time	Height	Height %	USP Width	Area	Area %
1	0.556	807391	100.000	0.028	938410	100.000

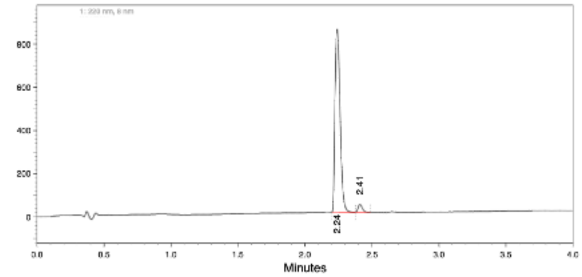

1: 220 nm, 8 nm

Retention Time	*Height*	*Area*	*Area Percent*
2.24	827136	2263534	96.56
2.41	35429	80642	3.44

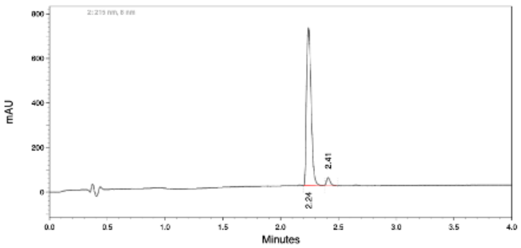

2: 215 nm, 8 nm

Retention Time	*Height*	*Area*	*Area Percent*
2.24	693053	1952748	96.08
2.41	35093	79742	3.92

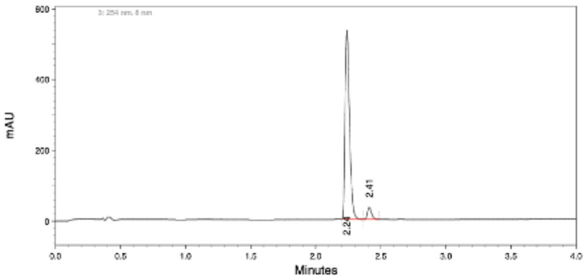

3: 254 nm, 8 nm

Retention Time	*Height*	*Area*	*Area Percent*
2.24	527426	1292764	94.54
2.41	32773	74710	5.46

Compound 46

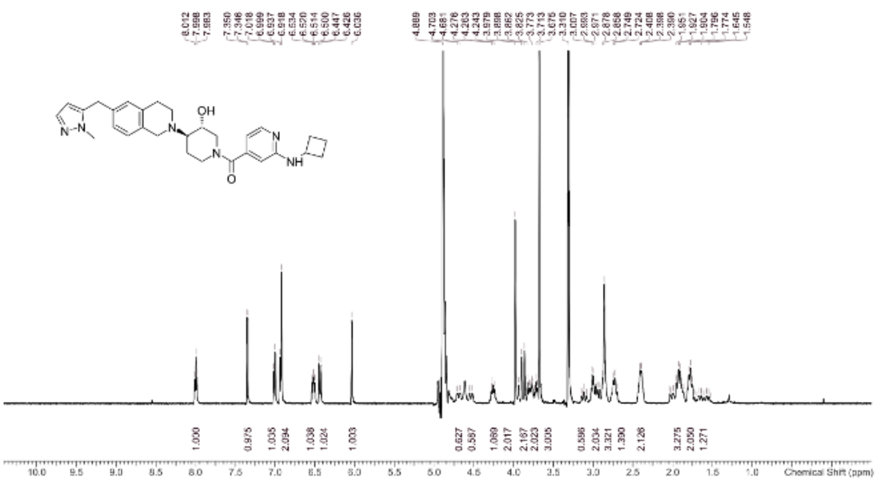

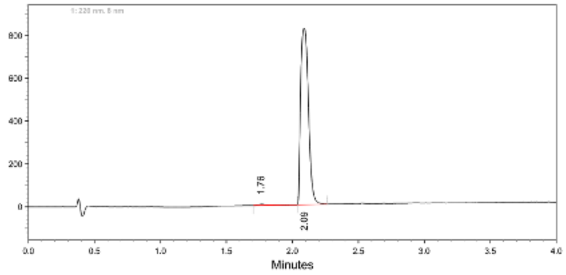

1: 220 nm, 8 nm

Retention Time	*Height*	*Area*	*Area Percent*
1.76	4484	16693	0.49
2.09	820149	3382358	99.51

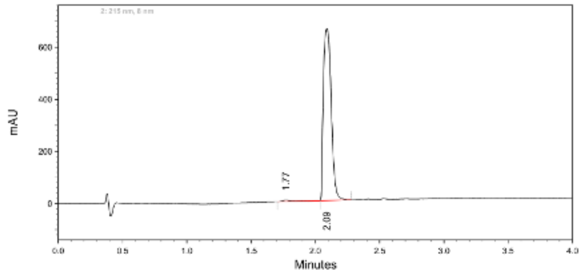

2: 215 nm, 8 nm

Retention Time	*Height*	*Area*	*Area Percent*
1.77	4945	17996	0.64
2.09	657091	2799233	99.36

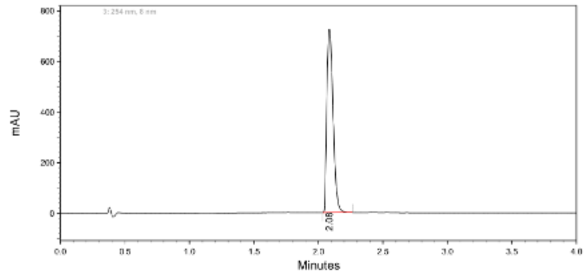

3: 254 nm, 8 nm

Retention Time	*Height*	*Area*	*Area Percent*
2.08	723376	2472905	100.00

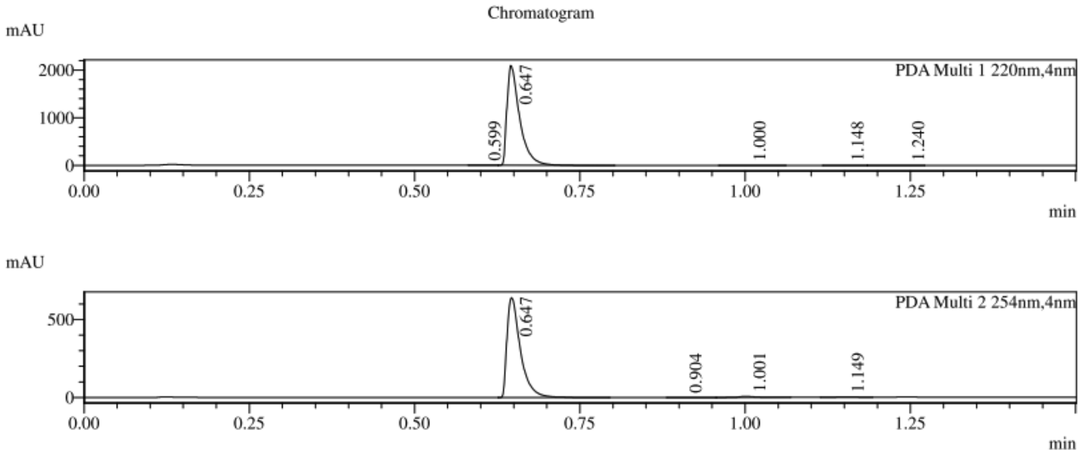

1 PDA Multi 1 / 220nm,4nm
2 PDA Multi 2 / 254nm,4nm

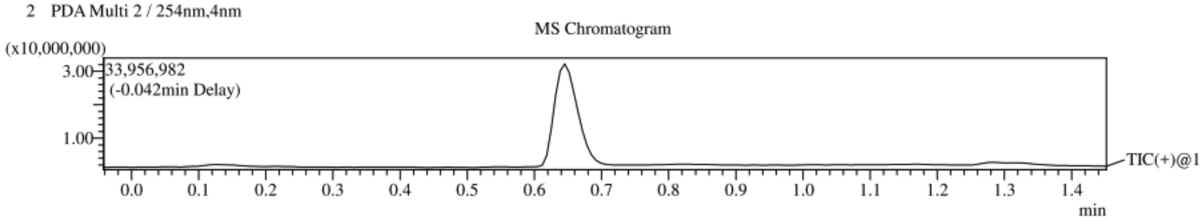

Integration Result

Peak Table

PDA Ch1 220nm

Peak#	Ret. Time	Height	Height%	USP Width	Area	Area%
1	0.599	4109	0.199	0.032	4857	0.172
2	0.647	2052197	99.346	0.033	2801564	99.259
3	1.000	4852	0.235	0.037	7464	0.264
4	1.148	3559	0.172	0.042	6093	0.216
5	1.240	989	0.048	0.044	2496	0.088

Peak Table

PDA Ch2 254nm

Peak#	Ret. Time	Height	Height%	USP Width	Area	Area%
1	0.647	635436	98.528	0.035	908710	98.323
2	0.904	562	0.087	0.032	689	0.075
3	1.001	6738	1.045	0.039	11448	1.239
4	1.149	2194	0.340	0.039	3361	0.364

Mass Spectrum

RetTime: 0.645 Datafile: D:\DATA\1904\190416\NBK0007-702-P1B.lcd

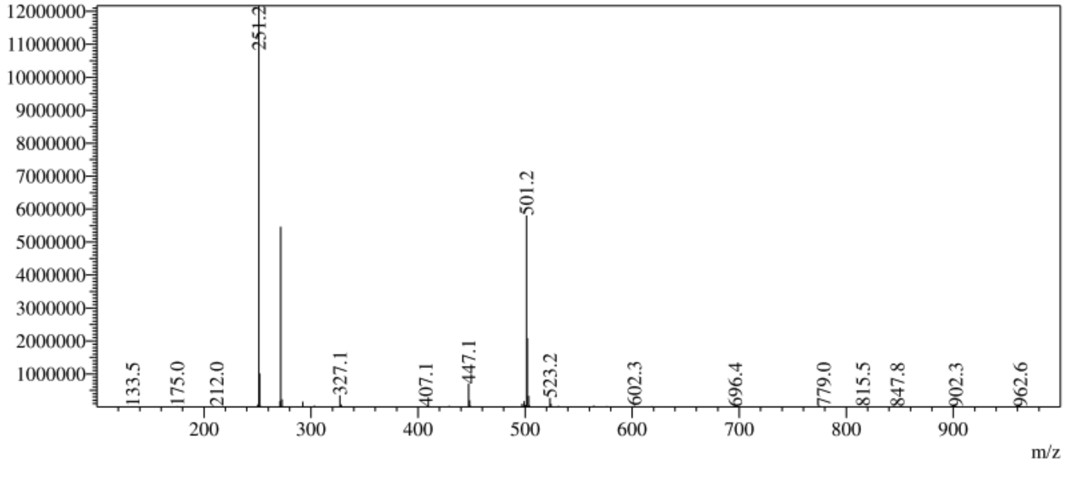

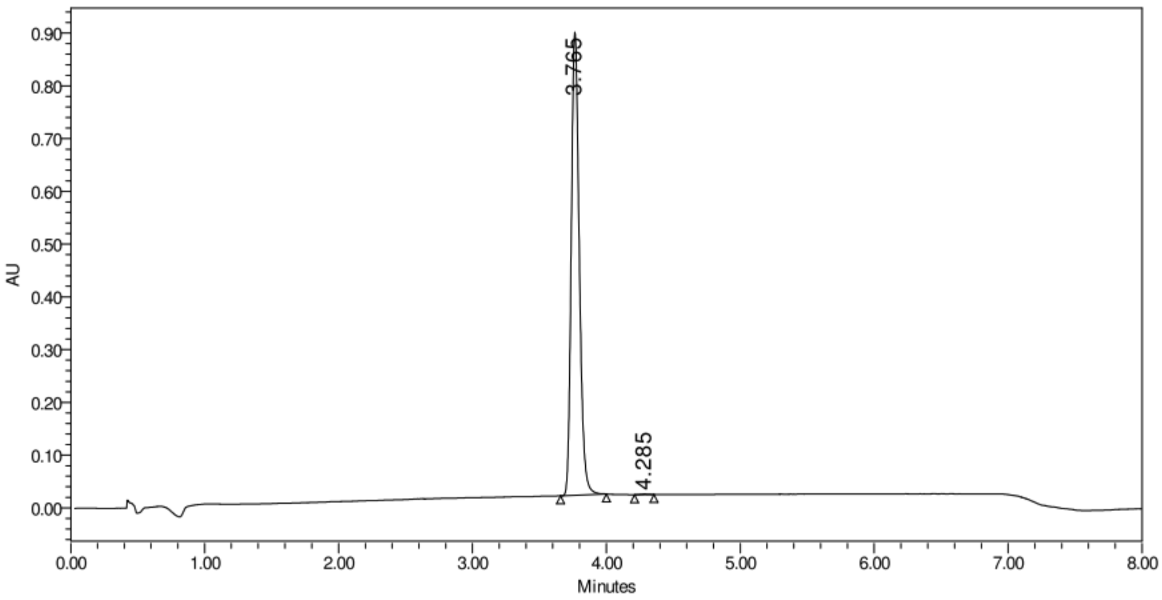

	RT	Area	% Area
1	3.765	3654049	99.85
2	4.285	5638	0.15

Instrument Method: AS_ETOH_DEA_5_40_28ML_8MIN

Compound 47

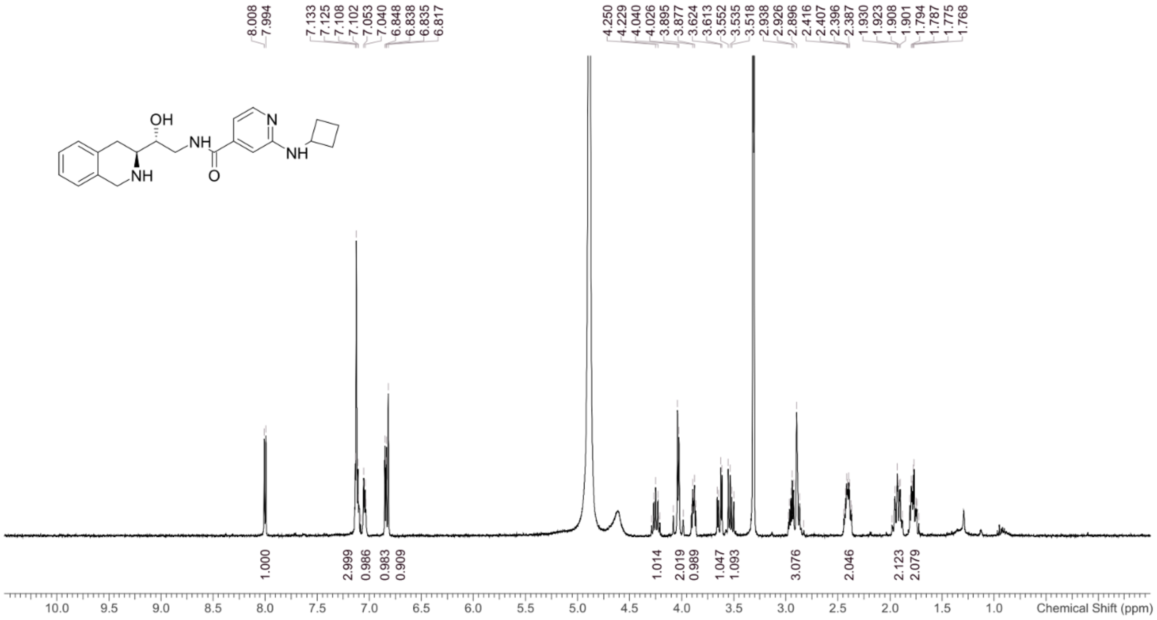

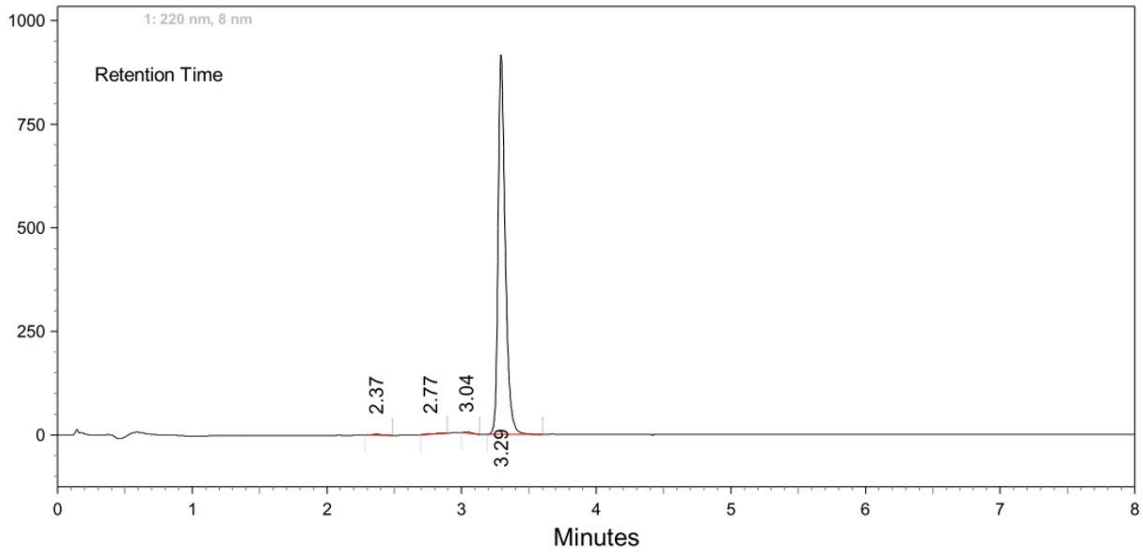

1: 220 nm, 8 nm

Retention Time	*Height*	*Area*	*Area Percent*
2.37	2897	12673	0.37
2.77	1479	9473	0.28
3.04	2886	10664	0.31
3.29	912215	3353586	99.03

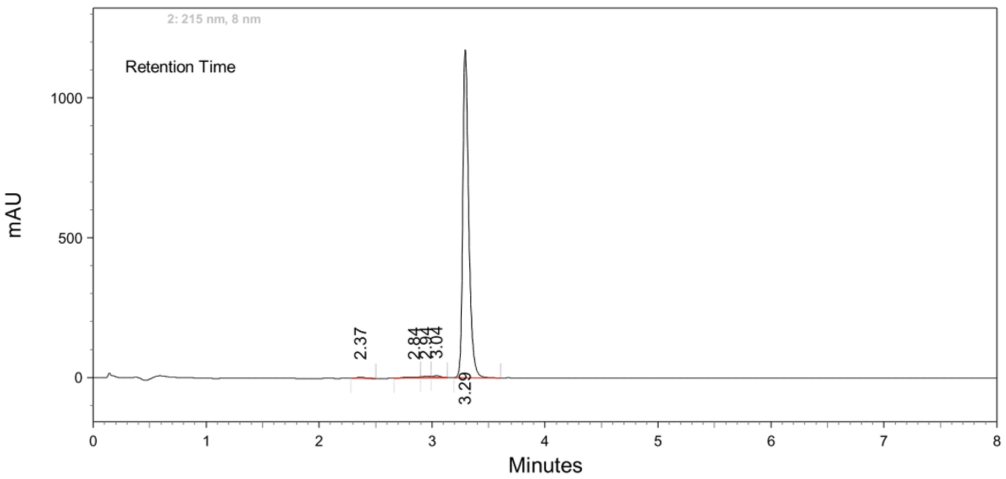

2: 215 nm, 8 nm

Retention Time	***Height***	***Area***	***Area Percent***
2.37	5143	22869	0.52
2.84	4146	39178	0.88
2.94	6149	32213	0.73
3.04	7918	39942	0.90
3.29	1168698	4297207	96.97

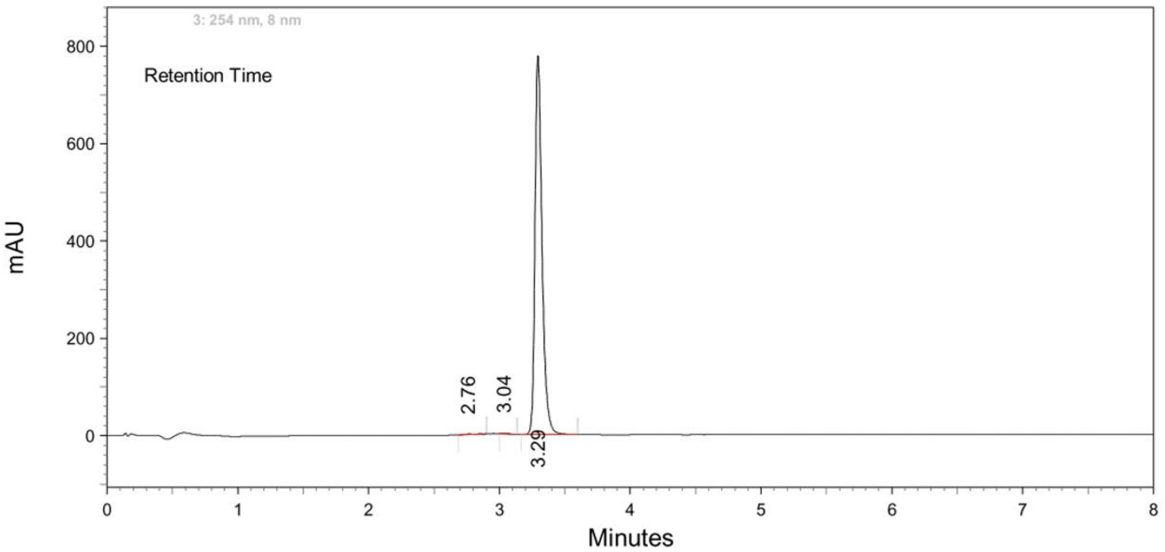

3: 254 nm, 8 nm

Retention Time	***Height***	***Area***	***Area Percent***
2.76	1176	6355	0.22
3.04	1701	5996	0.21
3.29	775983	2908808	99.58

Instrument & column:LCMS-BG 28-106
Agilent Pursit 5 C18 20*2.0mm

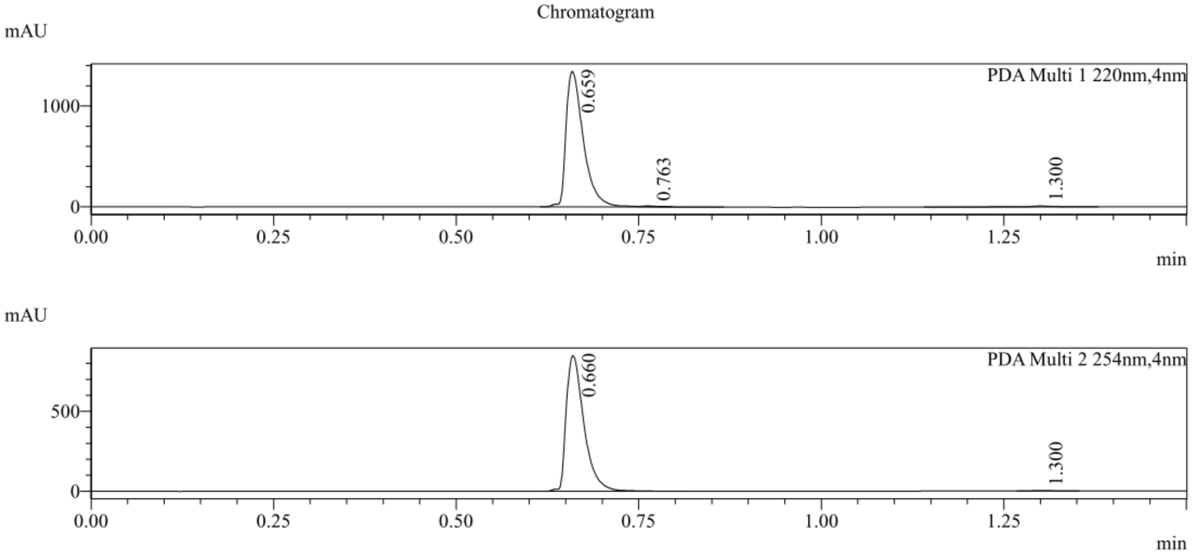

1 PDA Multi 1 / 220nm,4nm
2 PDA Multi 2 / 254nm,4nm

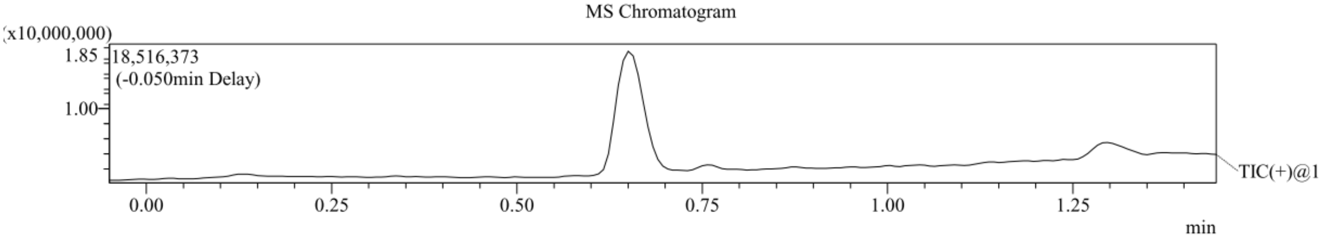

Integration Result

Peak Table

PDA Ch1 220nm

Peak#	Ret. Time	Height	Height%	USP Width	Area	Area%
1	0.659	1334182	98.849	0.041	2234156	98.599
2	0.763	5494	0.407	0.027	5515	0.243
3	1.300	10039	0.744	0.044	26241	1.158

Peak Table

PDA Ch2 254nm

Peak#	Ret. Time	Height	Height%	USP Width	Area	Area%
1	0.660	835241	99.829	0.042	1438567	99.844
2	1.300	1429	0.171	0.039	2245	0.156

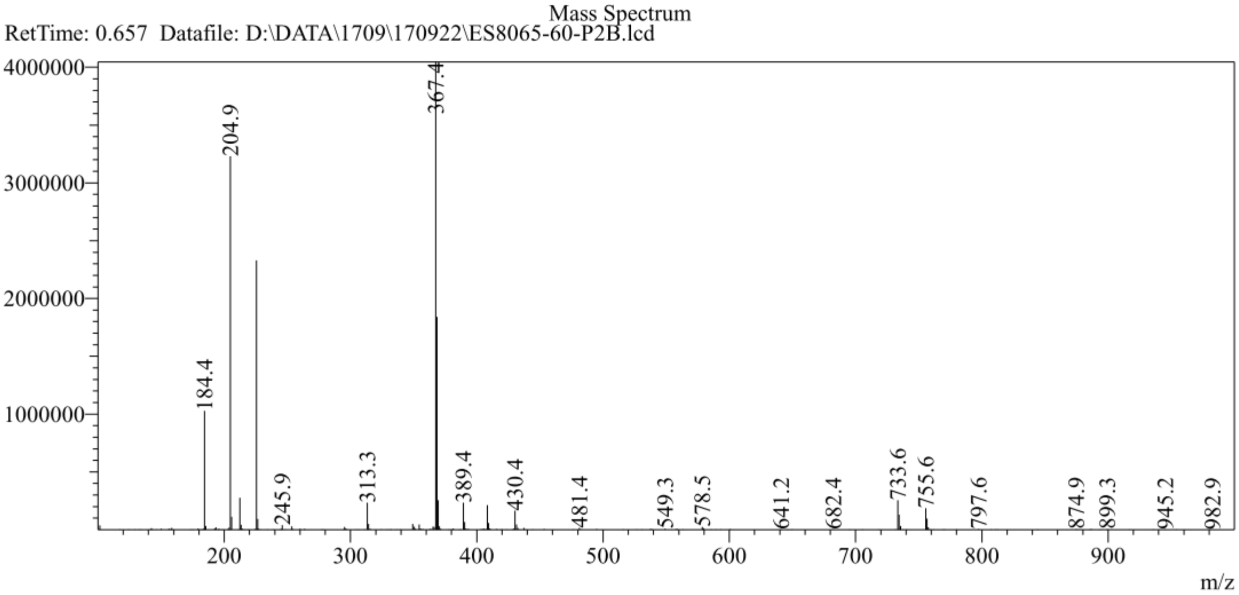

Method :Column: Chiralcel OD-3 50*4.6mm I.D., 3um
Mobile phase: A: CO2 B:methanol (0.05% DEA)
Gradient: hold 5% for 0.2 min,then from 5% to 40% of B in 1.4 min and hold 40% for 1.05 min, then 5% of B for 0.35 min
Flow rate: 4mL/min Column temp:40 C

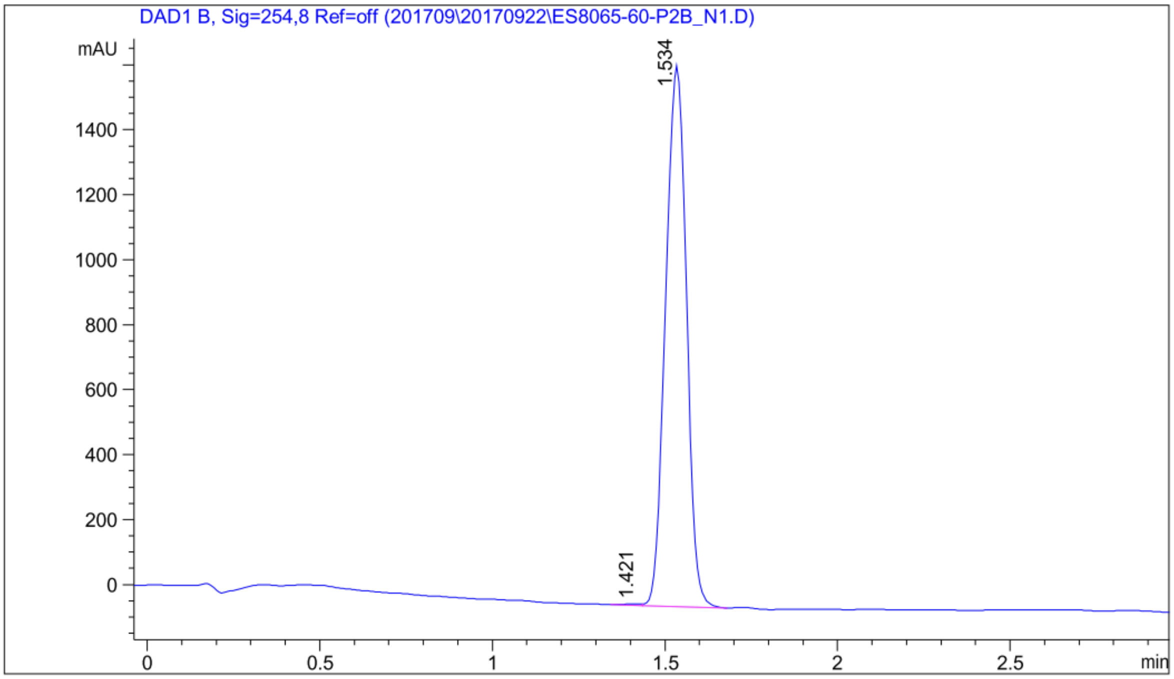

Signal 1 : DAD1 B, Sig=254,8 Ref=off

Peak	Meas. Ret. Time	Height	Height %	Width	Area	Area %
1	1.421	5.572	0.334	0.049	17.633	0.251
2	1.534	1664.913	99.666	0.067	7006.638	99.749

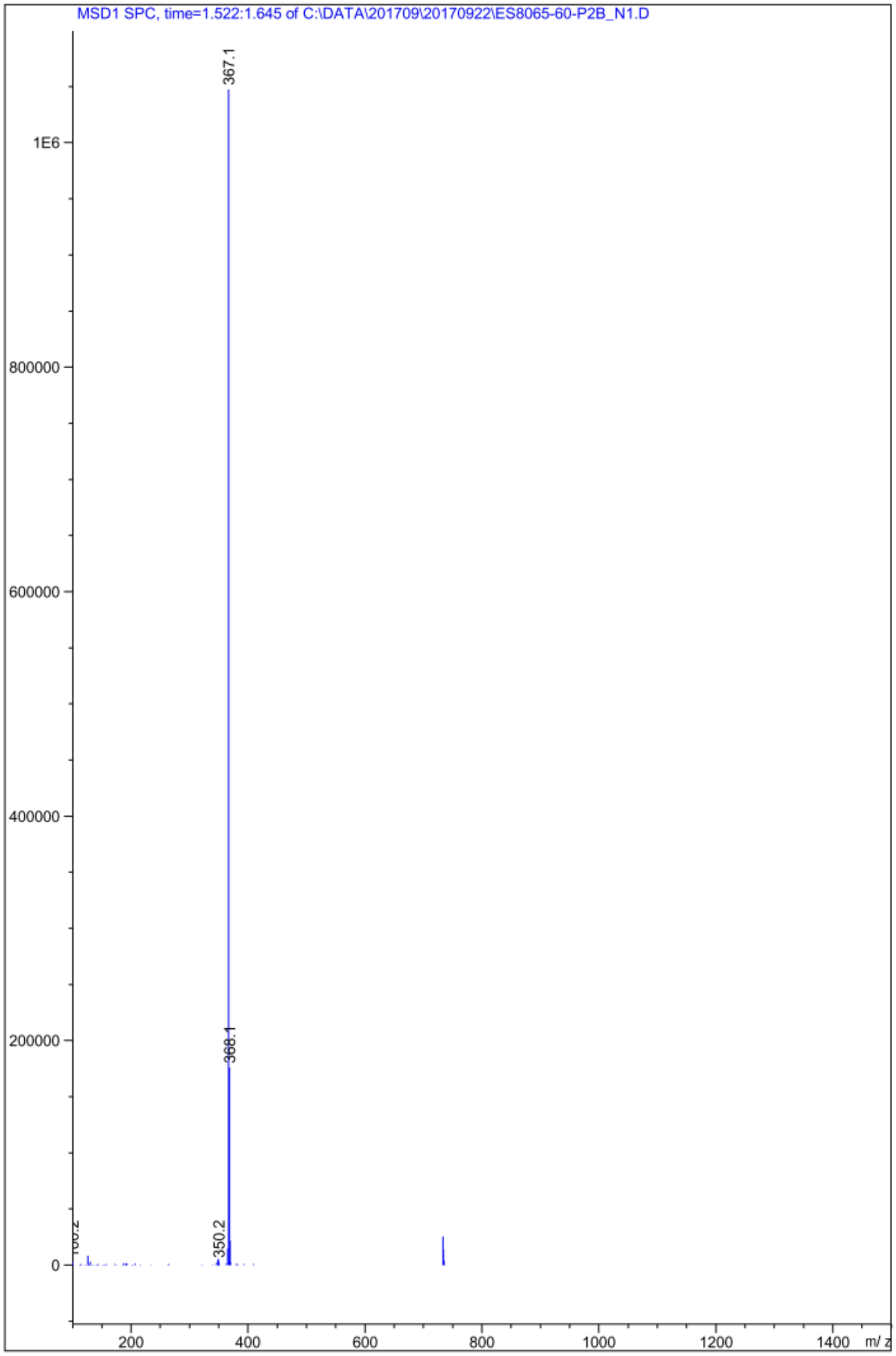

MSD1 SPC, time=1.522:1.645 of C:\DATA\201709\20170922\ES8065-60-P2B_N1.D
367.1
368.1
350.2
1E6
800000
600000
400000
200000
0
200
400
600
800
1000
1200
1400
m/z

Compound 48

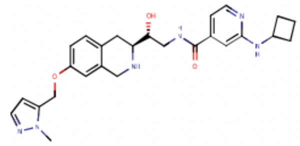

Mol Wt 476.57
Exact Mass 476.29

```
#  Time   Area%
-----------------
1  0.848  100.00
```

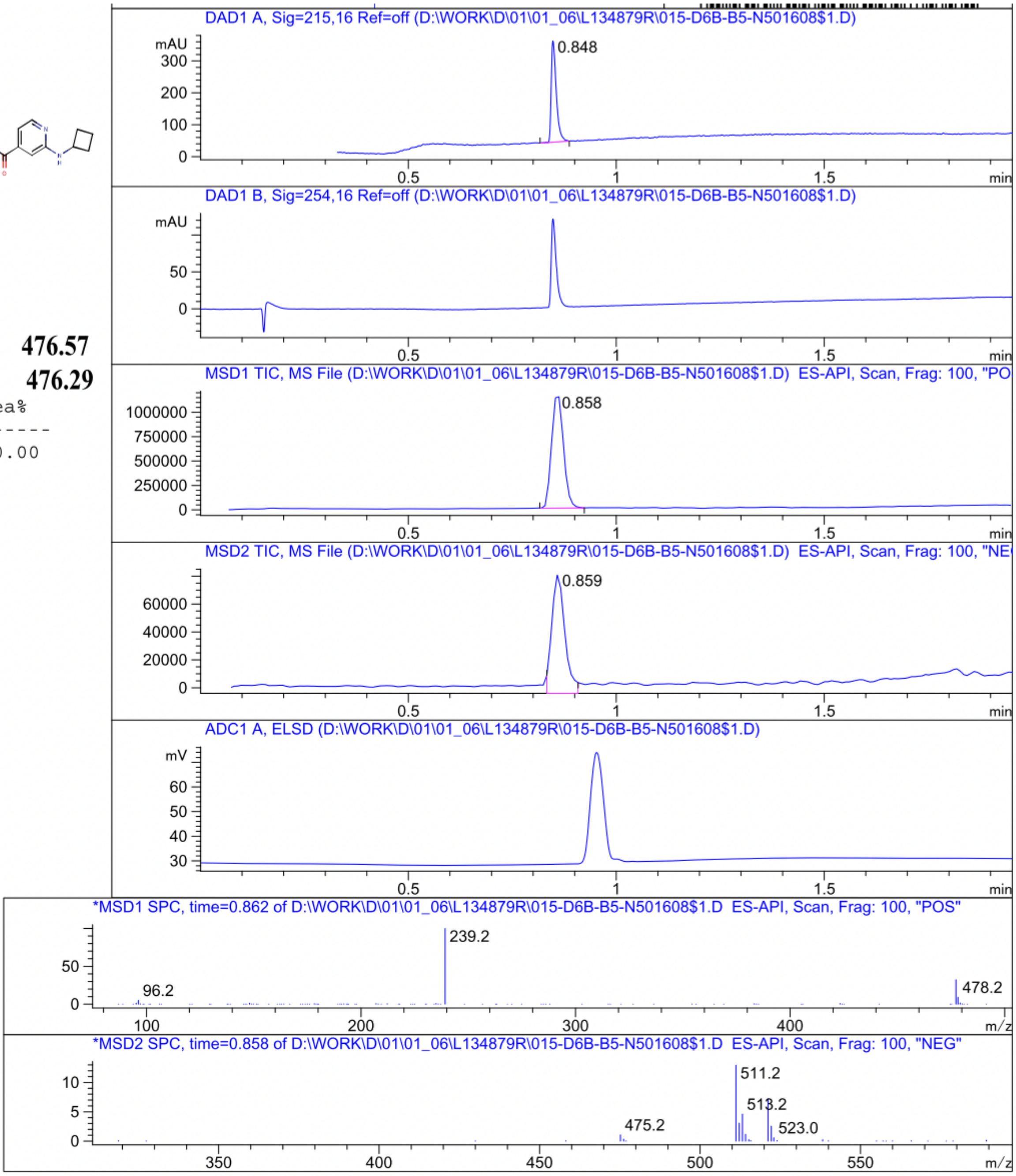

RT 0.858

RT 0.859

Compound 49

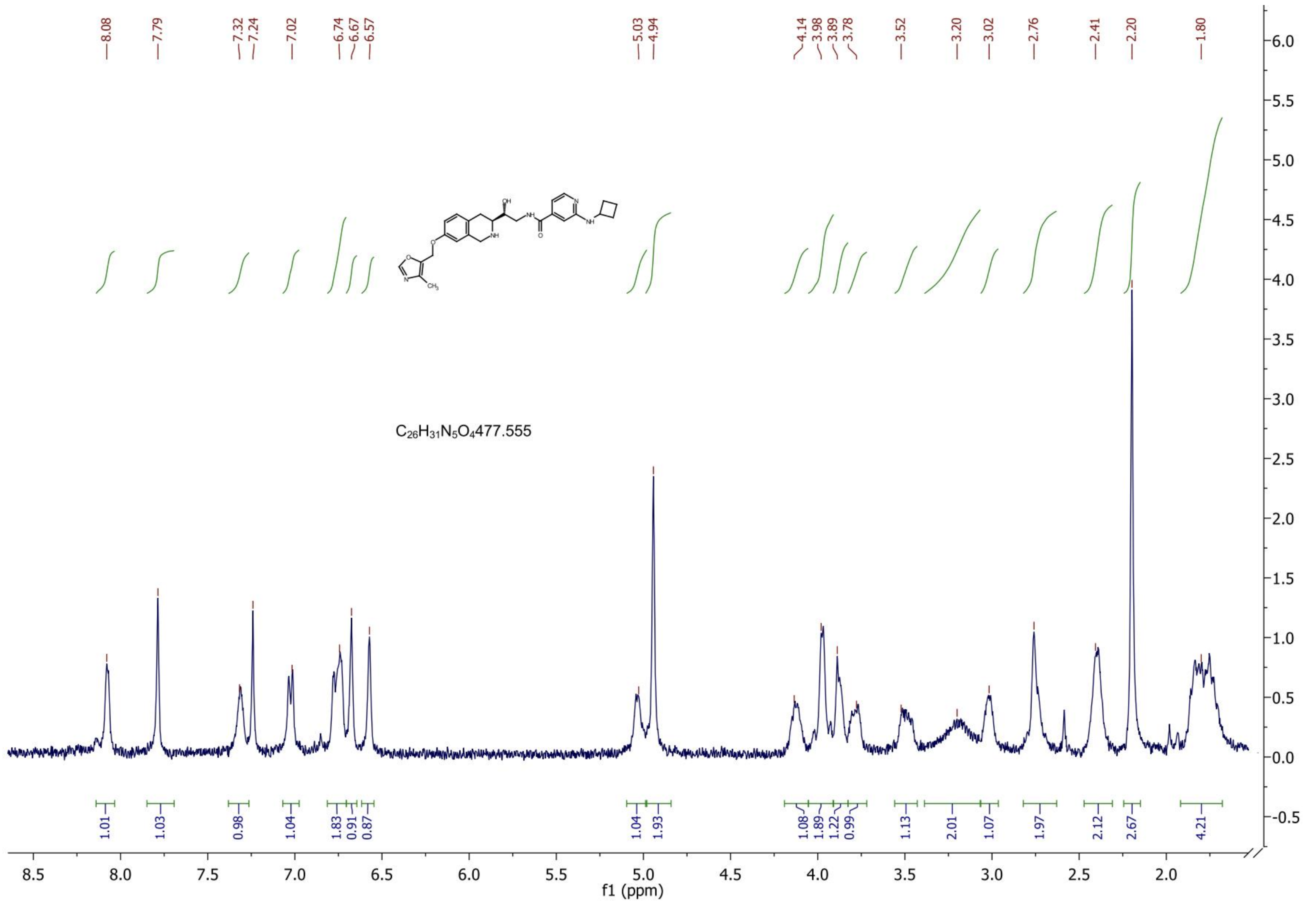

8.08
7.79
7.32
7.24
7.02
6.74
6.67
6.57
5.03
4.94
4.14
3.98
3.89
3.78
3.52
3.20
3.02
2.76
2.41
2.20
1.80
C26H31N5O4477.555
1.01
1.03
0.98
1.04
1.83
0.91
0.87
1.04
1.93
1.08
1.89
1.22
0.99
1.13
2.01
1.07
1.97
2.12
2.67
4.21
f1 (ppm)

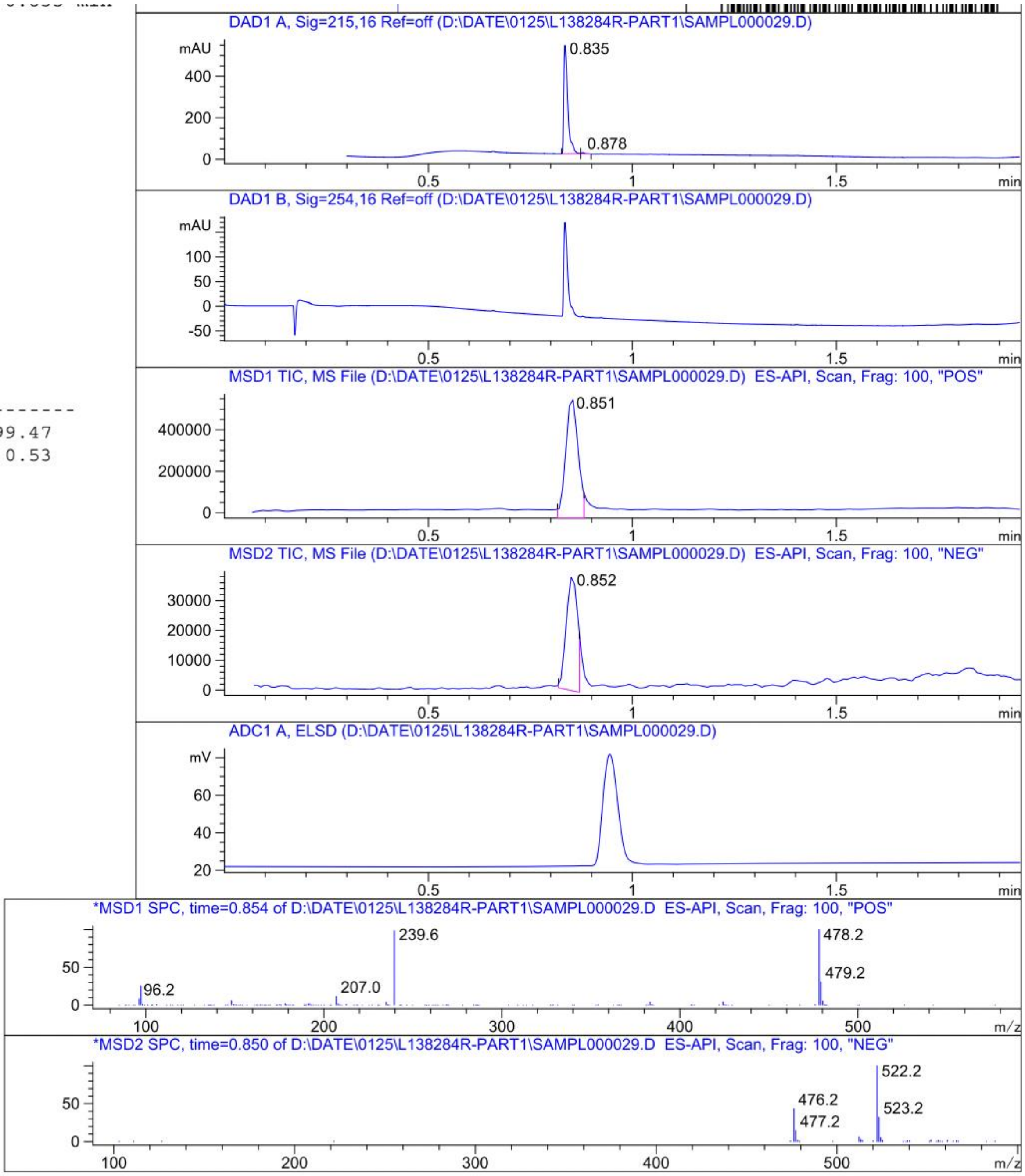

----
1 0.835 99.47
2 0.878 0.53

RT 0.851

RT 0.852

Compound 50

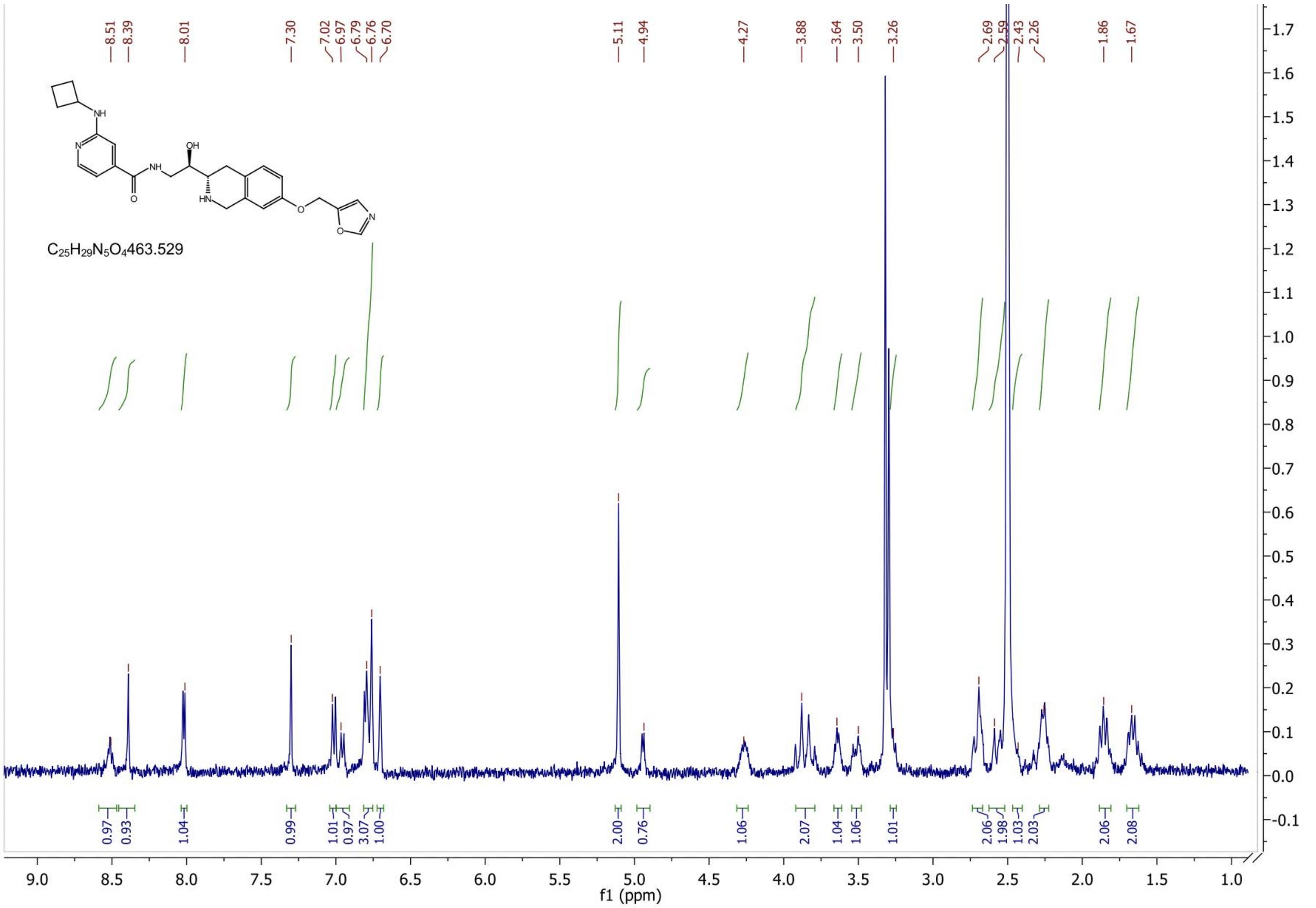

8.51
8.39
8.01
7.30
7.02
6.97
6.79
6.76
6.70
5.11
4.94
4.27
3.88
3.64
3.50
3.26
2.69
2.59
2.43
2.26
1.86
1.67
NH
N
OH
NH
O
HN
O
N
O
C25H29N5O4463.529
0.97
0.93
1.04
0.99
1.01
0.97
3.07
1.00
2.00
0.76
1.06
2.07
1.04
1.06
1.01
2.06
1.98
1.03
2.03
2.06
2.08
f1 (ppm)

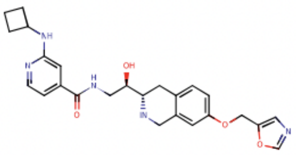

Mol Wt 463.53

Exact Mass 463.25

#	Time	Area%
1	0.769	100.00

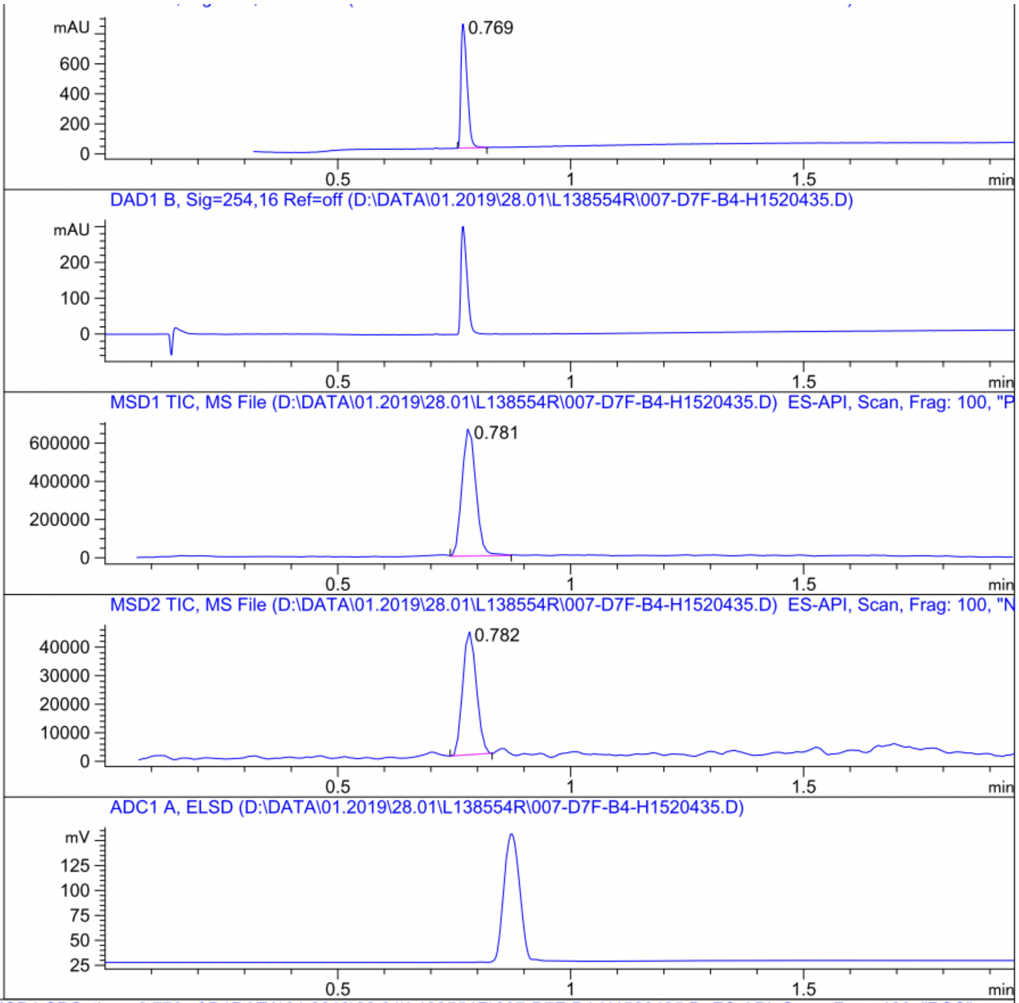

RT 0.781

RT 0.782

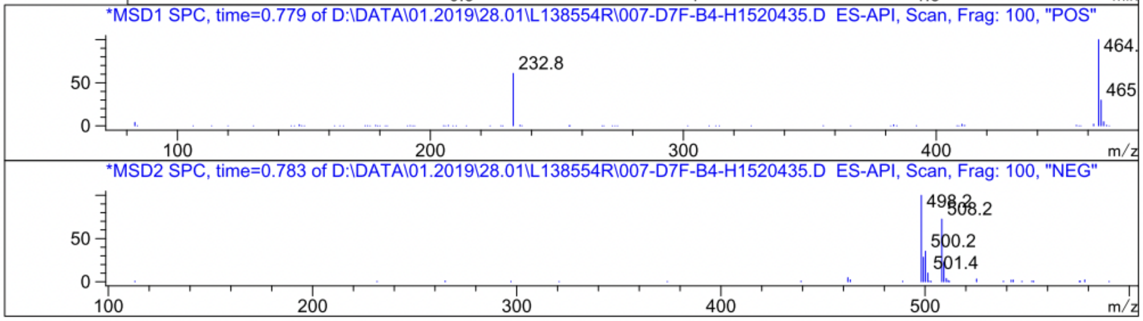

Compound 51

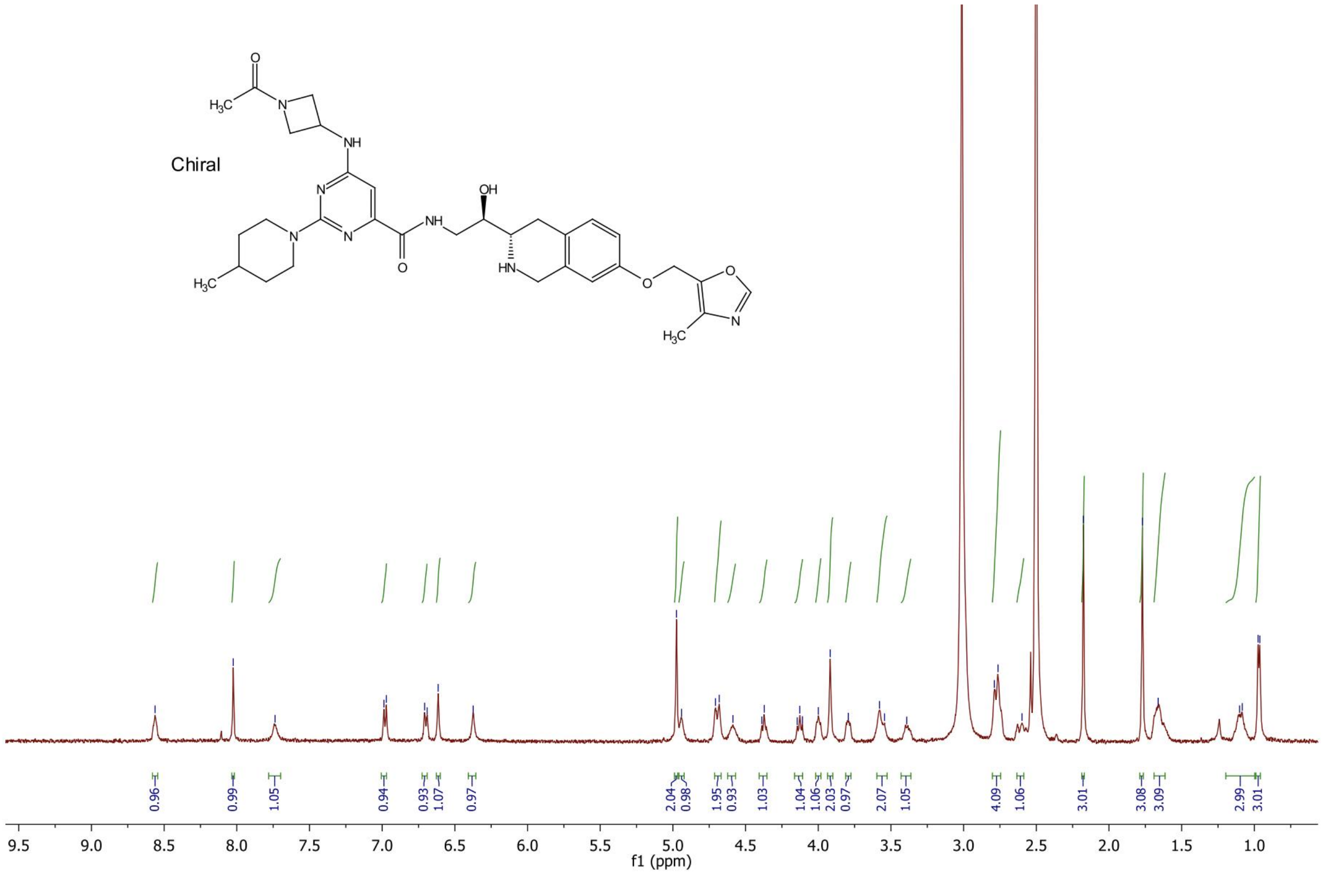

Chiral
O
H3C
N
NH
N
N
N
OH
NH
O
HN
O
O
N
H3C
H3C
0.96
0.99
1.05
0.94
0.93
1.07
0.97
2.04
0.98
1.95
0.93
1.03
1.04
1.06
2.03
0.97
2.07
1.05
4.09
1.06
3.01
3.08
3.09
2.99
3.01
9.5
9.0
8.5
8.0
7.5
7.0
6.5
6.0
5.5
5.0
4.5
4.0
3.5
3.0
2.5
2.0
1.5
1.0
f1 (ppm)

MaxPeak: 100.00%
Ret_Time: 1.072 min

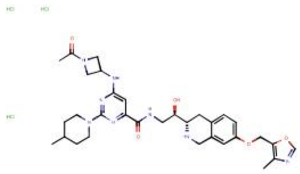

Mol Wt 728.11

Exact Mass 618.37

#	Time	Area%
1	1.072	100.00

RT 1.083

RT 1.084

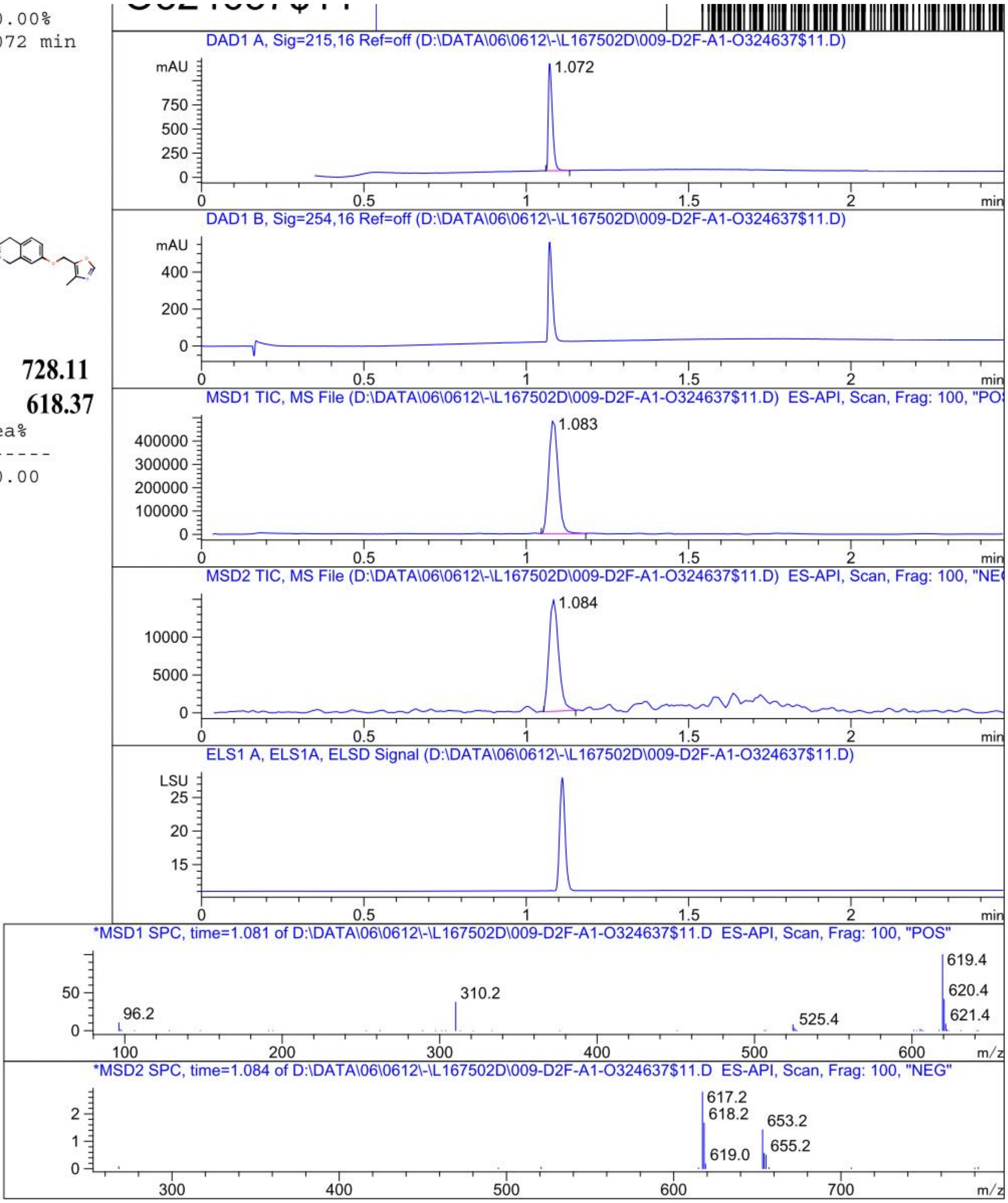

Compound 52

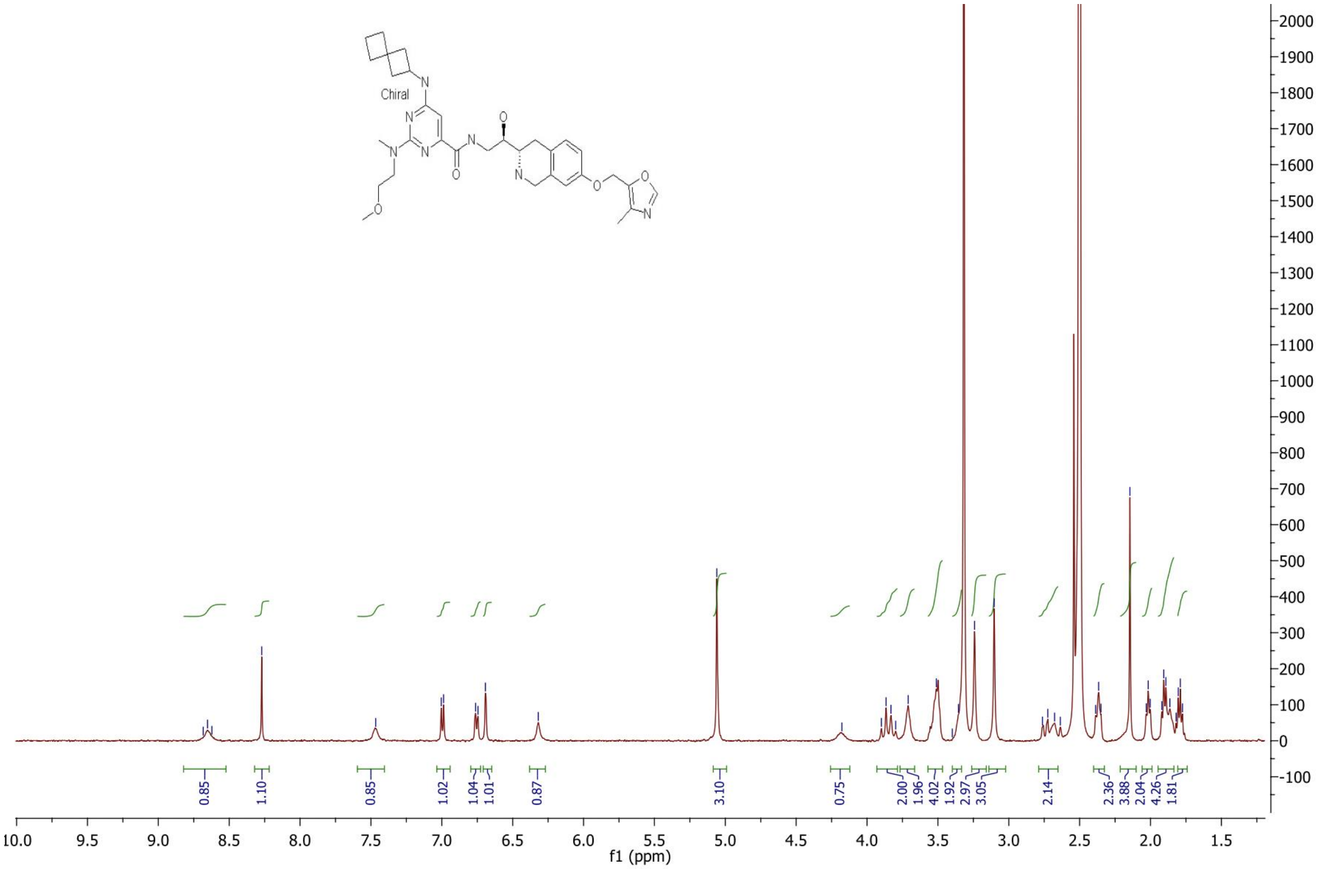
Chiral
0.85
1.10
0.85
1.02
1.04
1.01
0.87
3.10
0.75
2.00
1.96
4.02
1.92
2.97
3.05
2.14
2.36
3.88
2.04
4.26
1.81
f1 (ppm)

Ret_Time: 1.240 min

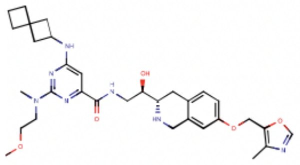

Mol Wt 605.73
Exact Mass 605.38

#	Time	Area%
1	1.240	100.00

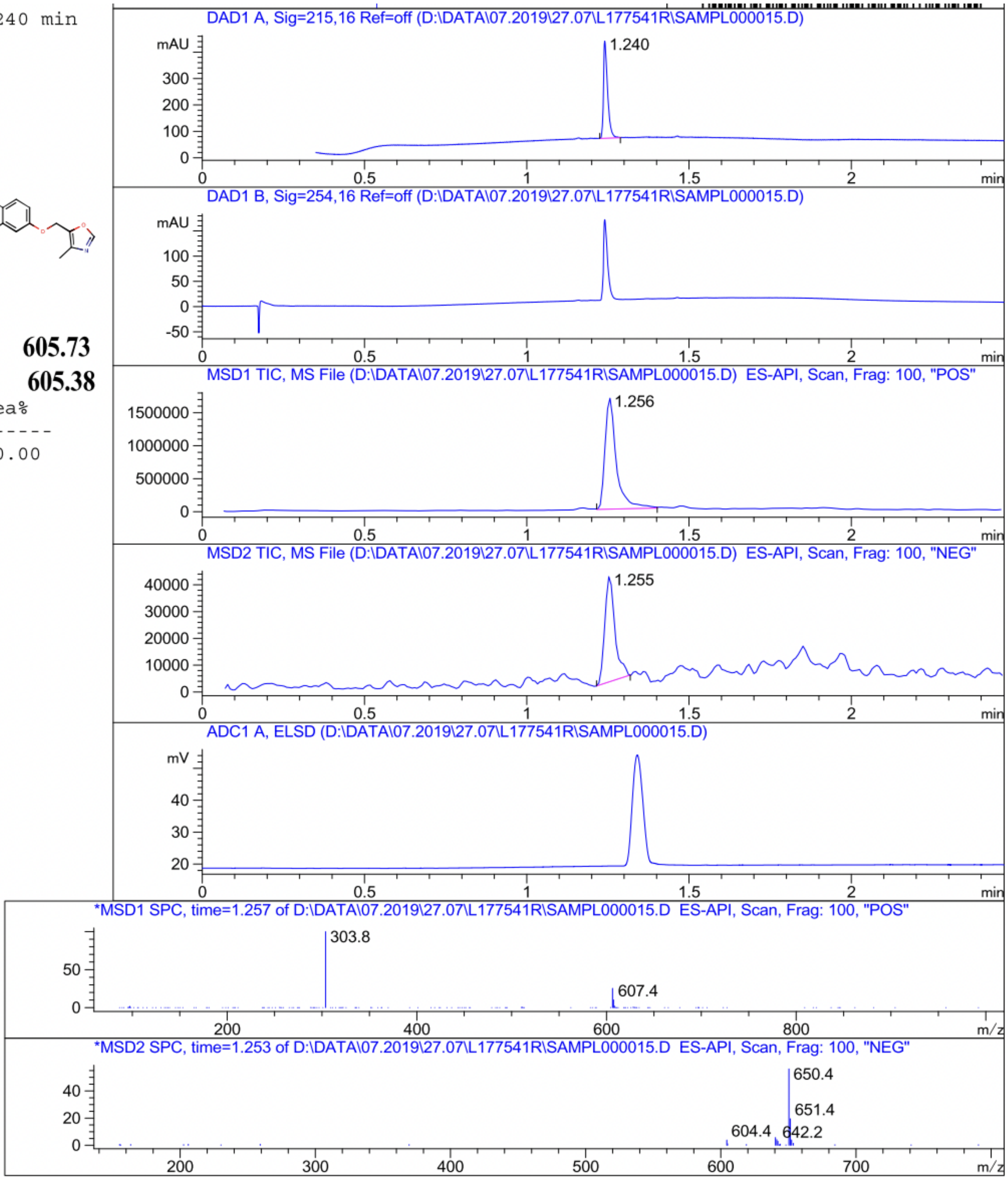

RT 1.256

RT 1.255

Compound 53

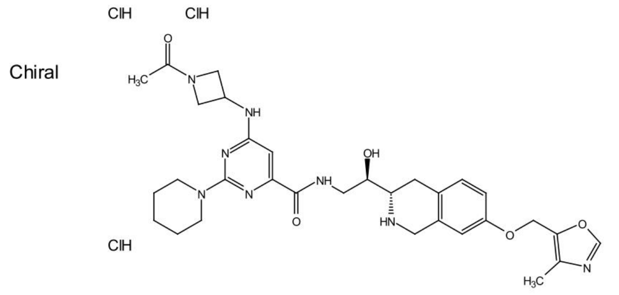
Chiral
ClH
ClH
ClH
OH
NH
HN

$C_{31}H_{40}N_8O_5$

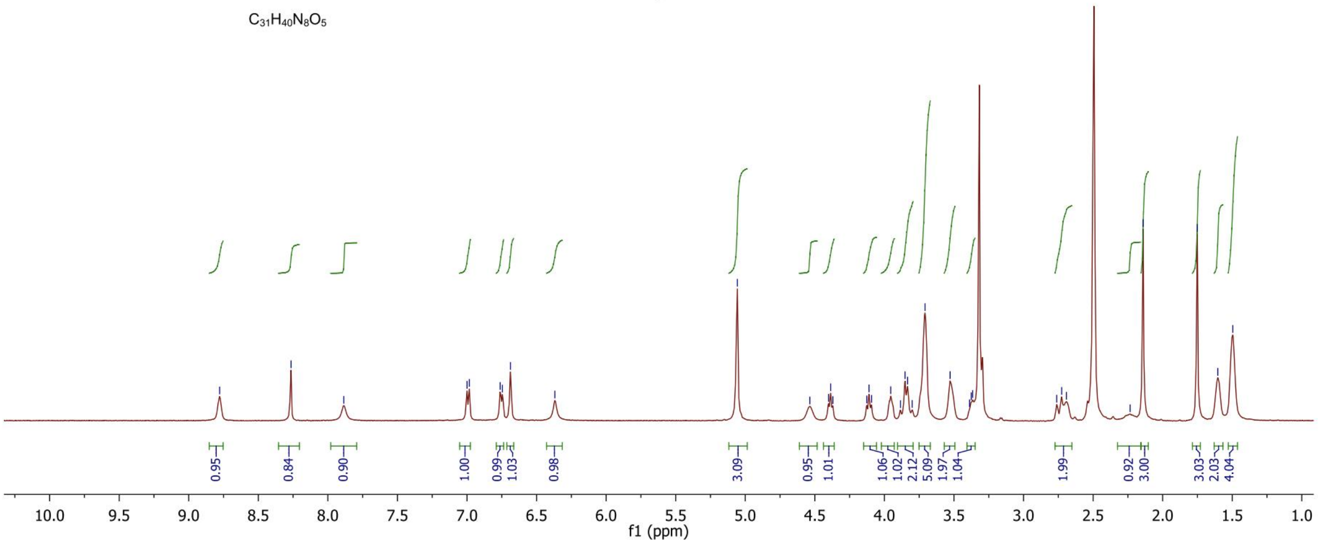
0.95
0.84
0.90
1.00
0.99
1.03
0.98
3.09
0.95
1.01
1.06
1.02
2.12
5.09
1.97
1.04
1.99
0.92
3.00
3.03
2.03
4.04
f1 (ppm)

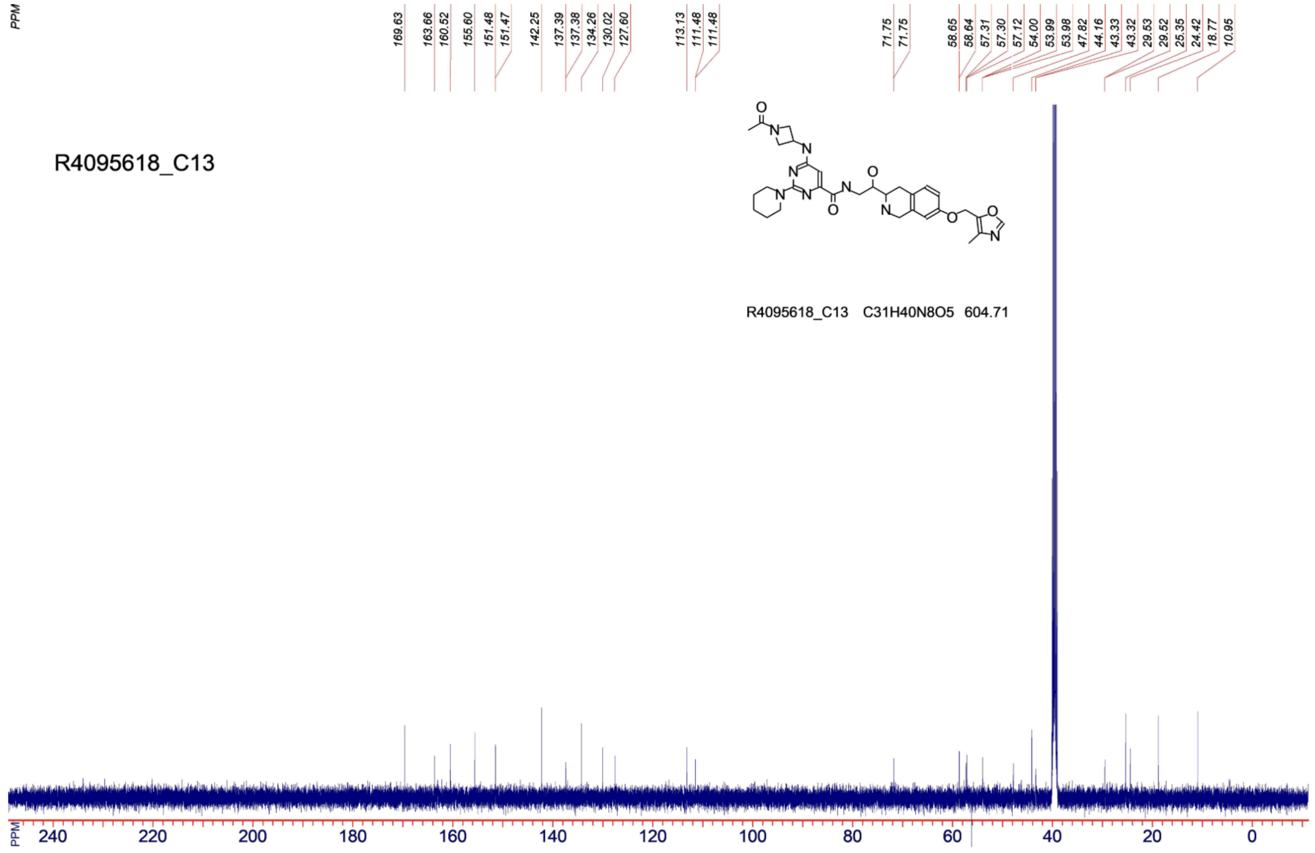
PPM
169.63
163.66
160.52
155.60
151.48
151.47
142.25
137.39
137.38
134.26
130.02
127.60
113.13
111.48
111.48
71.75
71.75
58.65
58.64
57.31
57.30
57.12
54.00
53.99
53.98
47.82
44.16
43.33
43.32
29.53
29.52
25.35
24.42
18.77
10.95
R4095618_C13
R4095618_C13 C31H40N8O5 604.71

MaxPeak: 95.89%
Ret_Time: 1.039 min

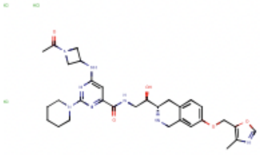

Mol Wt 714.08

Exact Mass 604.35

#	Time	Area%
1	0.901	1.51
2	0.964	2.59
3	1.039	95.89

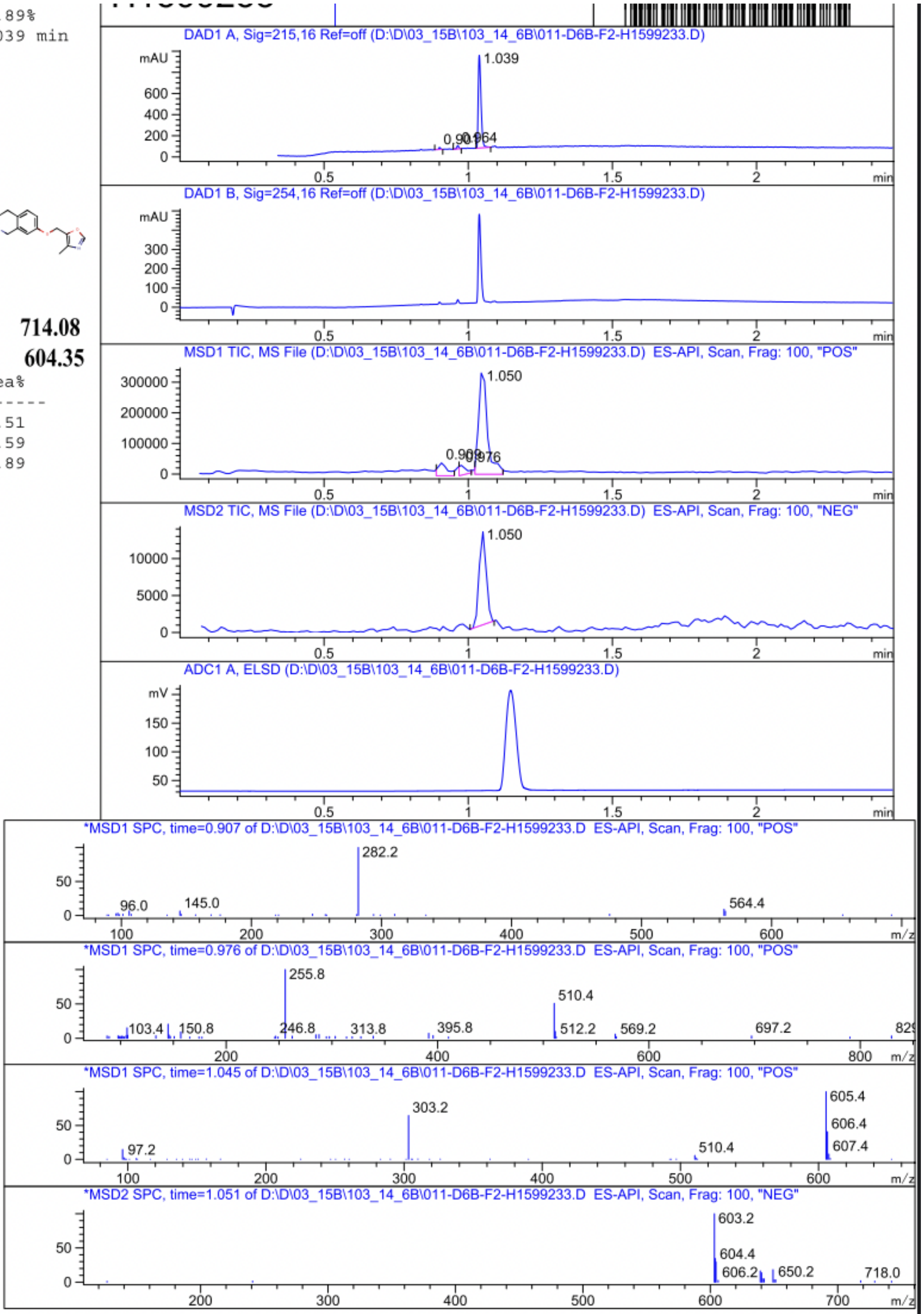

RT 0.909

RT 0.976

RT 1.050

RT 1.050

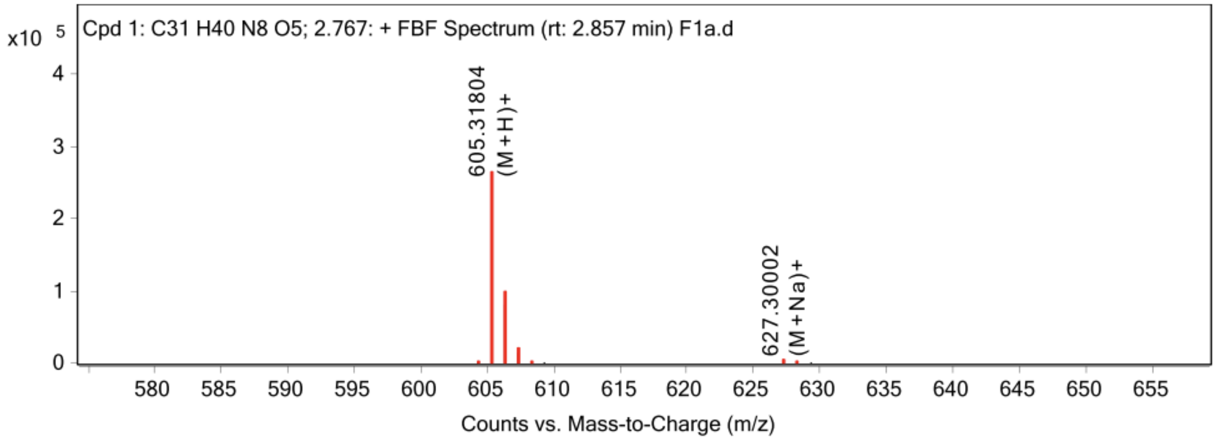

MS Spectrum Peak List

Obs. *m/z*	Charge	Abund	Ion/Isotope
604.30683	1	1288.95	M+
605.31804	1	265424.97	(M+H)+
606.32147	1	80913.59	(M+H)+
607.32345	1	14247.96	(M+H)+
608.32595	1	2149.34	(M+H)+
609.32618	1	356.2	(M+H)+
627.30002	1	4166.96	(M+Na)+
628.30097	1	1390.58	(M+Na)+
629.30327	1	424.08	(M+Na)+

MS Zoomed Spectrum

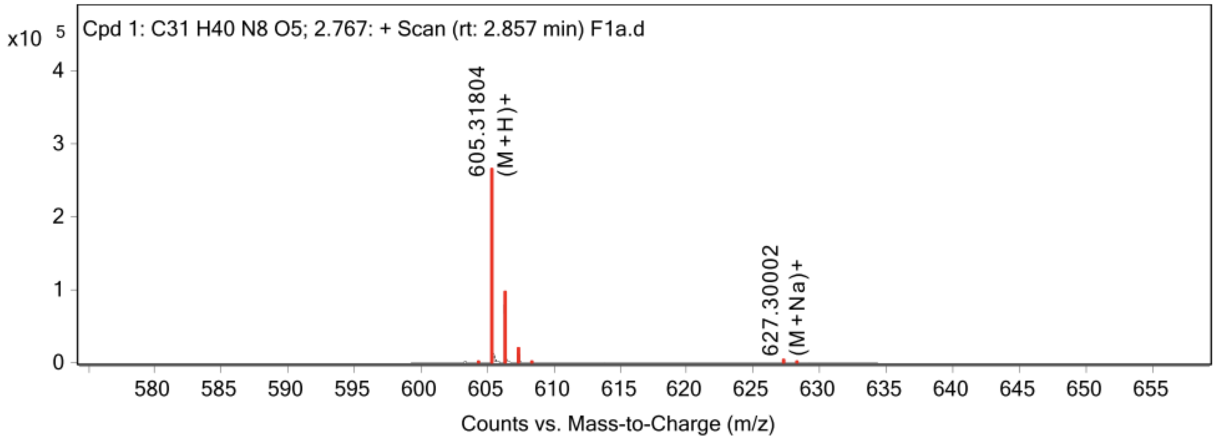

MS Spectrum Peak List

Obs. *m/z*	Charge	Abund	Ion/Isotope	Tgt Mass Error (ppm)
604.30683	1	1288.95	M+	7.92
605.31804	1	265424.98	(M+H)+	2.32
605.31804		265424.98		
606.32147	1	80913.59	(M+H)+	1.44
607.32345	1	14247.96	(M+H)+	2.59
608.32595	1	2149.34	(M+H)+	2.72
609.32618	1	356.2	(M+H)+	6.48
627.30002	1	4166.96	(M+Na)+	2.18
628.30097	1	1390.58	(M+Na)+	5.28
629.30327	1	424.08	(M+Na)+	5.87

--- End Of Report ---

Compound 54

Chiral

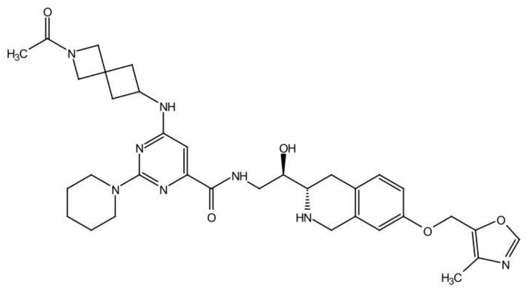

O
H3C
N
NH
N
N
N
OH
NH
O
HN
O
O
N
H3C

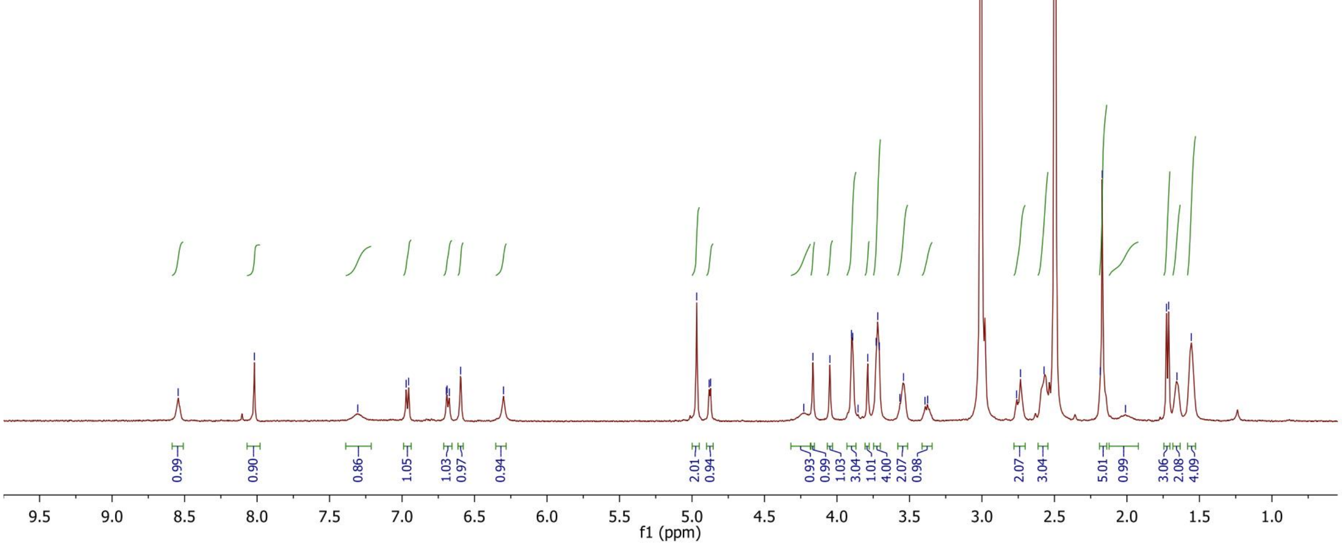

0.99
0.90
0.86
1.05
1.03
0.97
0.94
2.01
0.94
0.93
0.99
1.03
3.04
1.01
4.00
2.07
0.98
2.07
3.04
5.01
0.99
3.06
2.08
4.09
9.5 9.0 8.5 8.0 7.5 7.0 6.5 6.0 5.5 5.0 4.5 4.0 3.5 3.0 2.5 2.0 1.5 1.0
f1 (ppm)

Ret_Time: 1.048 min

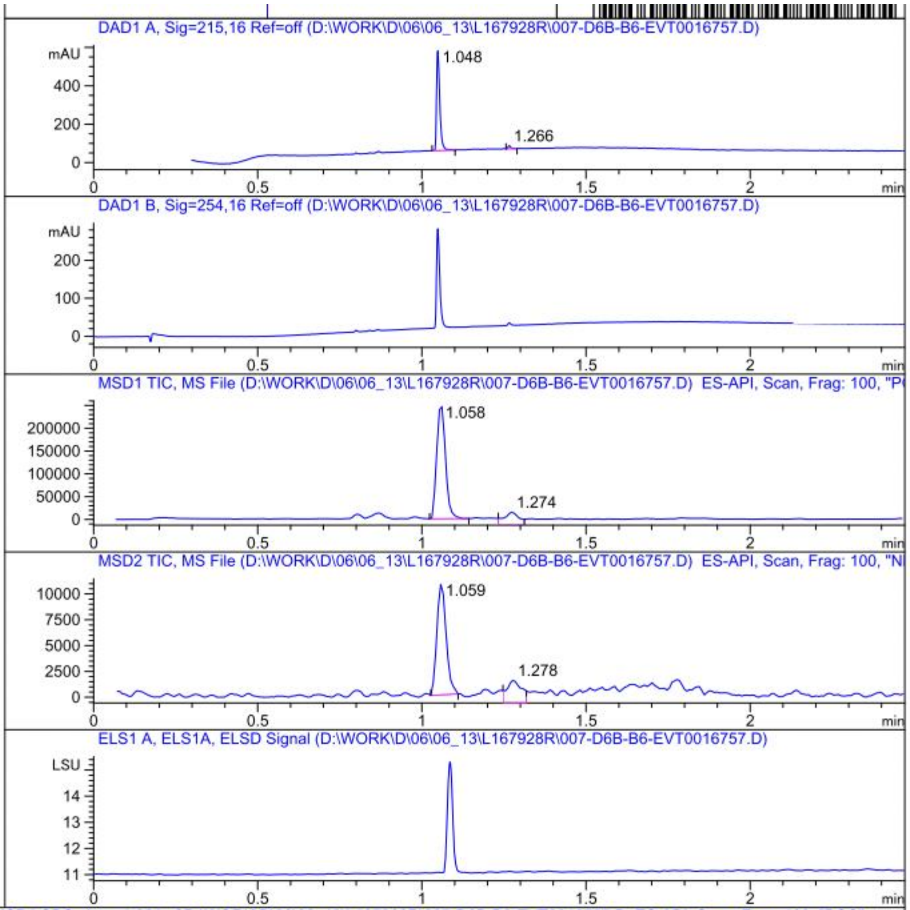

#	Time	Area%
1	1.048	97.15
2	1.266	2.85

RT 1.058

RT 1.274

RT 1.059

RT 1.278

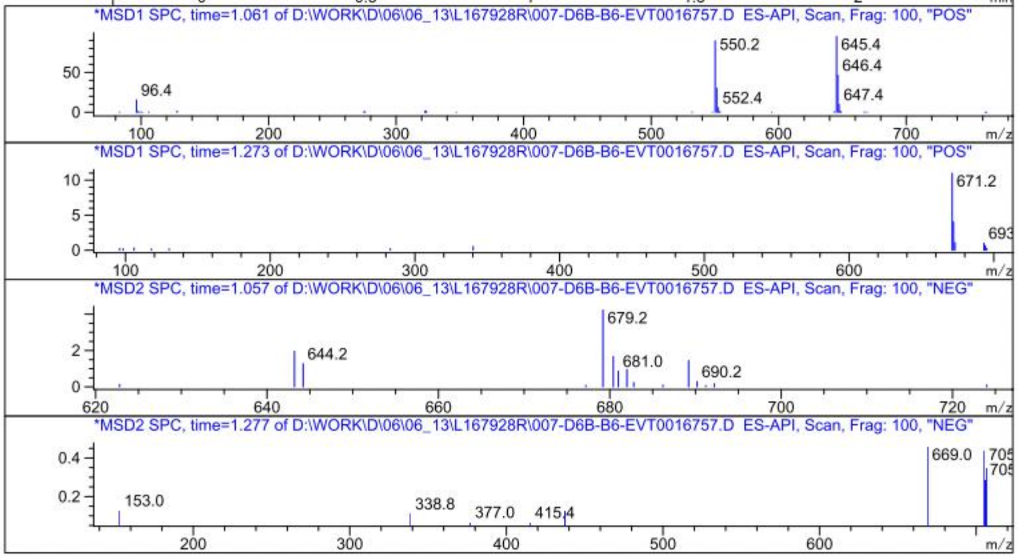

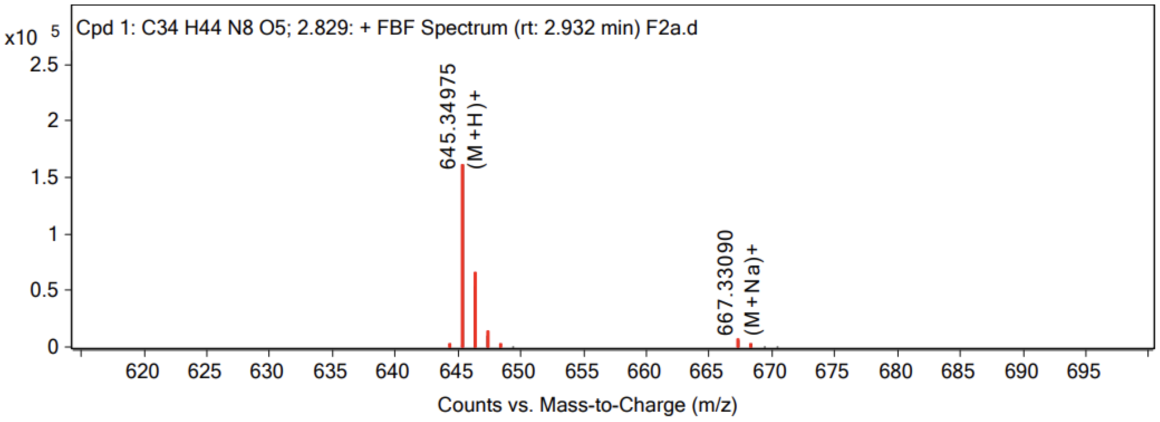

MS Spectrum Peak List

Obs. *m/z*	Charge	Abund	Ion/Isotope
644.33642	1	2450.03	M+
645.34975	1	161693.83	(M+H)+
646.35283	1	53602.52	(M+H)+
647.35588	1	10307.8	(M+H)+
648.35662	1	1389.39	(M+H)+
649.35167	1	241.08	(M+H)+
667.3309	1	6337.04	(M+Na)+
668.3336	1	2594.01	(M+Na)+
669.33307	1	662.04	(M+Na)+
670.33344	1	137.18	(M+Na)+

MS Zoomed Spectrum

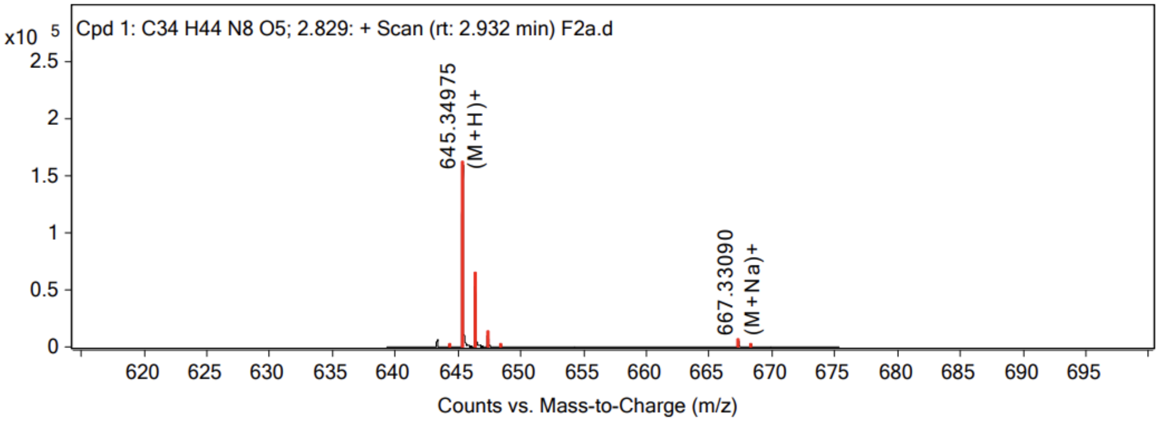

MS Spectrum Peak List

Obs. *m/z*	Charge	Abund	Ion/Isotope	Tgt Mass Error (ppm)
644.33642	1	2450.03	M+	10.08
645.34975	1	161693.83	(M+H)+	1.54
646.35283	1	53602.52	(M+H)+	1.32
647.35588	1	10307.8	(M+H)+	0.84
648.35662	1	1389.39	(M+H)+	3.77
649.35167	1	241.08	(M+H)+	15.35
667.3309	1	6337.04	(M+Na)+	2.68
668.3336	1	2594.01	(M+Na)+	3.03
669.33307	1	662.04	(M+Na)+	7.91
670.33344	1	137.18	(M+Na)+	11.28

--- End Of Report ---

BIOCHEMICAL FLUORESCENCE ANISOTROPY PEPTIDE DISPLACEMENT ASSAY.

A fluorescence anisotropy (FA) assay was established to measure binding of C-terminal 5'-TAMRA labeled histone H4 peptide (1-21) with PRMT5/MEP50. The test compound competes with the peptide to bind to PRMT5/MEP50 protein and thus would act to disrupt binding by the labeled histone H4 peptide. The assay buffer consisted of 30 mM Bicine (pH 8.0), 150 mM NaCl, 1.5 mM DTT, 0.003% Tween-20. The two peptides were utilized for these studies were Me_0: Ac-SGRGKGGKGLGKGGAKRHRKV-K(5-TAMRA)-NH_2 and Me_2: Ac-SGR(Sym Me_2)GKGGKGLGKGGAKRHRKV-K(5-TAMRA)-NH_2. Me_0 peptide is not methylated and used to determine the compound potency in the presence of 50 µM 5'-methylthioadenosine (MTA). Its K_D with PRMT5•MTA = 4.6 nM. Me_2 peptide is symmetrically methylated at Arginine 3 and used to determine the compound potency in the presence of 50 µM S-adenosyl methionine (SAM). Its K_D with PRMT5•SAM = 79 nM. Inhibitor potency was assessed at equilibrium by measuring the dose dependent displacement of a fixed concentration of the peptide from PRMT5/MEP50. Following incubation at room temperature for 30 minutes, the plate was read on an Envision plate reader. For data analysis, fluorescence anisotropy (FA) detected equals 1000 * (S - G * P) / (S + G * 2 * P) where: S = detector 2 or channel 2 signal, P = detector 1 or channel 1 signal, G = G-factor. Fluorescence anisotropy is normalized to %inhibition using: %inhibition=(Signal-MinAVG)/(MaxAVG-MinAVG)*100, where MinAVG = the average value of Min value and MaxAVG = the average value of Max value. Curves are fitted by XL-Fit as %inhibition vs. log [compound concentration] using a 4-parameter logistic equation with fixed 0% and 100% inhibition limits.

The Cheng-Prusoff equation[1] for a competitive inhibitor $K_{i,app} = IC_{50}/(1+ [\text{Peptide probe}]/K_D)$ was applied, where [Peptide probe] = the concentration of the H4 peptide probe used in the assay. K_D = the equilibrium dissociation constant of peptide probe, representing the peptide probe concentration at which 50% of the proteins are bound to the probes at equilibrium.

HAP1 MTAP WT AND MTAP-NULL IN-CELL WESTERN ASSAY.

A HAP1 *MTAP*-isogenic cell line pair was acquired from Horizon Discovery (HZGHC004894c005) and maintained in DMEM (high glucose) + 10% FBS in a humidified, 10% CO_2 tissue culture incubator. The SAM-cooperative PRMT5 inhibitor, GSK3326595, was sourced from Selleck Chemicals and maintained as a 10 mM DMSO stock. All test compounds are maintained as 10 mM DMSO stocks.
On Day 0, MTAP WT or MTAP-null cells are seeded in a 384-well plate, and incubated in a humidified, 5% CO_2 tissue culture incubator for 16-24h. On Day 1, the test compounds are dispensed to wells at defined concentrations using a Tecan D300e digital dispenser (n=4), and the volume of DMSO is normalized to highest class volume. Each plate includes wells dosed with defined concentrations of GSK3326595 as a plate control. The compounds are incubated with cells for 24h in a humidified, 5% CO_2 tissue culture incubator.
On Day 2, the compound-treated cells are fixed with a final concentration of 4% formaldehyde. The cells are then washed/permeabilized with 1X PBS + 0.1% Triton X-100, and then blocked with 5% goat serum/1X TBS. The fixed cells are then incubated overnight at 4ºC with a primary SDMA antibody cocktail (Cell Signaling 13222).
On Day 3, the cells are washed with 1X PBS + 0.1% Triton X-100, and then incubated at room temperature for 1 h with a NIR fluorescent secondary antibody cocktail that also contains DRAQ5 (LiCor 926-32211 and VWR 10761-508). The cells are washed with 1X PBS + 0.1% Triton X-100, and then washed again with ddH_2O. The plates are then imaged using a NIR fluorescent imager (LiCor Odyssey).
For data analysis, the SDMA signal is normalized to the DRAQ5 signal. Assay background is determined by the signal from wells treated with 1 µM GSK3326595 and subtracted from every well. The data are plotted as % of the DMSO control wells for the MTAP WT and the MTAP-null cell lines independently and fitted to the 4-parameter logistic (4-PL) Hill equation with maximal effect constrained to 0. The fit was performed using GraphPad Prism or the default IC_{50} fitting procedure in Dotmatics Studies 5.4 as part of a customized data analysis protocol.

HAP1 MTAP WT AND MTAP-NULL VIABILITY ASSAY.

A HAP1 *MTAP*-isogenic cell line pair was acquired from Horizon Discovery (HZGHC004894c005) and maintained in DMEM (high glucose) + 10% FBS in a humidified, 5 or 10% CO_2 tissue culture incubator. All test compounds are maintained as 10 mM DMSO stocks. On Day 0, MTAP WT and MTAP-null cells are seeded in a 96-well plate, and incubated in a humidified, 5 or 10% CO_2 tissue culture incubator for 16-24h. On Day 1, the test compounds are dispensed to wells at defined concentrations using a Tecan D300e digital dispenser (n=3), and the volume of DMSO is normalized to highest class volume (0.2%). The compound-treated plates are incubated for 7 days in a humidified, 5 or 10% CO_2 tissue culture incubator. On Day 7, the plates are removed from the tissue culture incubator and allowed to equilibrate to room temperature. Then either a ½ volume CellTiter-Glo® Luminescent

Cell Viability Assay reagent (Promega G7572) is added to each well, or the media is removed from every well and a 1:3 dilution of CellTiter-Glo® 2.0 Cell Viability Assay reagent (Promega G9241) in 1X PBS is added. Ten minutes after addition, the luminescent signal is detected by an Envision plate reader. The data are plotted as % of the DMSO control wells for the MTAP WT and the MTAP-null cell lines independently and fitted to the 4-parameter logistic (4-PL) Hill equation with maximal effect constrained to 0. The fit was performed using GraphPad Prism or the default IC_{50} fitting procedure in Dotmatics Studies 5.4 as part of a customized data analysis protocol.

CELLULAR LUCIFERASE-BASED THERMOSTABILITY ASSAY.

HAP1 *MTAP*-deleted cells were engineered to express exogenous PRMT5 fused to an N-terminal NanoLuc® luciferase construct and the endogenous PRMT5 gene was knocked out using CRISPR-based editing. The stable cell line was harvested by trypsinization, washed with 1x PBS and resuspended in PRMT5 Activity Buffer (Bicine at 30mM, Tween-20 at 0.003%, NaCl at 150mM, Q.S. w/Water) supplemented with 1X Halt protease inhibitor cocktail (ThermoFisher #78446). Cells were counted and aliquoted into separated PCR tubes at 1.0×10^6 cells/mL and were lysed by repeated freeze thaw cycles (liquid nitrogen for 3 min followed by 15 min in a room temperature water bath x 3 cycles). Lysates were centrifuged for 20 min and the supernatants were transferred to PCR tubes and compounds were dosed as indicated for 30 min. Lysates were supplemented with 10 μM MTA when profiling MTA-cooperative PRMT5 inhibitors. Supplementation with exogenous SAM was deemed unnecessary for GSK3326595 as PRMT5 stability was ~300% at 10 μM GSK3326595 + 10 μM SAM in an independent study, which is the equivalent maximum effect determined with the conditions used here. Samples were then heated for 3 min and cooled to RT using a Bio-Rad T100 PCR thermal cycler. Individual wells were transferred to a 96 well white clear bottom plate at 25 µL per well and mixed with 25 µL of RT equilibrated Nano-Glo Luciferase substrate (#N1120) for 5 min. Plates were read on Envision luminometer.

MDCKII AND MDR1-MDCKII ASSAYS.

Wild type (WT) MDCKII cells or MDR1-MDCKII cells (both obtained from Piet Borst at the Netherlands Cancer Institute) were seeded onto the polycarbonate membranes in the 96- well insert system at 4.44×10^5 cells/mL and cultured for 4-7 days until confluence before being used for the transport studies. Test compounds were diluted with the transport buffer (HPSS with 10 mM HEPES, pH 7.4) from DMSO stock solution to a concentration of 2 µM (DMSO < 1%) and applied to the apical or basolateral side of the cell monolayer. The plate was incubated for 2.5 h in CO_2 incubator at 37±1 °C, with 5% CO_2 at saturated humidity without shaking. Permeation of the test compounds from A to B or B to A direction was determined in duplicate. In addition, the efflux ratio of each compound was also calculated. For each transport assay, digoxin (P-gp efflux substrate) was tested at 10.0 µM bidirectionally, while nadolol (low permeability marker) and metoprolol (high permeability marker) were tested at 2.00 µM in A to B direction in duplicate. Test and reference compounds were quantified by LC-MS/MS analysis based on the peak area ratio of analyte/internal standard (IS). After transport assay, Lucifer yellow fluorescence rejection assay was performed to confirm the integrity of the cell monolayer.

HUMAN LIVER MICROSOME METABOLIC STABILITY ASSAY.

Test and reference compounds (testosterone, diclofenac and propafenone) at 1 µM were incubated individually in human liver microsome (0.5 mg protein/mL, from mixed-gender donors) supplemented with 1 mM NADPH at 37 °C for 60 minutes while shaking. Aliquots of 60 µl were taken at 5, 15, 30, 45 and 60 minutes of incubation and reactions were stopped by adding 180 ul of quenching solution. After which all sampling plates were shaken for 10 minutes, then centrifuged at 4000 rpm for 20 minutes at 4 °C. Supernatants were transferred to HPLC water (1:3) and mixed for 10 minutes prior to the LC-MS/MS analysis. Test and reference compounds were quantified by LC-MS/MS analysis based on the peak area ratio of analyte/internal standard (IS).

KINETIC SOLUBILITY ASSAY.

Medium:
The preparation of 50 mM phosphate buffer (PB) with pH 7.4:
The preparation of 50 mM NaH_2PO_4: Dissolved 3.000 g of NaH_2PO_4 in 500 mL H_2O, and the pH measured was about 4.5.
The preparation of 50 mM NaH_2PO_4: Dissolved 3.549 g of NaH_2PO_4 in 500 mL H_2O, and the pH measured was about 9.4.
The preparation of 50 mM PB (pH 7.4): 15 mL of 50 mM NaH_2PO_4 was added to a 50 mL tube and then adjusted to pH 7.4 +/- 0.05 with 50 mM NaH_2PO_4.

Procedure:

10μL of 10mM DMSO stock solution of test and control compounds was added into each well of a 96-well plate, respectively.
Added 490 μL of medium into the well of the 96-well plate, respectively.
Vortexed the solubility samples for at least 2 minutes.
Shook the 96-well plate on a shaker at room temperature at the speed of 800 rpm for 24h.
Centrifuged at 25 °C for 10 minutes (eq 4000 rpm).
Transferred the supernatant into a filter plate, and then collected the filtrates into a new 96-well plate by centrifuging for at least 5 minutes.
The concentrations of the filtrates were quantified by LC-UV system.

PRMT5:MEP50 EXPRESSION AND PURIFICATION.

Recombinant protein production of the human PRMT5:MEP50 complex was performed in the Hi5 insect cell expression system using protein constructs that were described previously2. Purification of the PRMT5:MEP50 complex was also performed as described previously.3 Purified protein was concentrated to >15 mg/mL and flash-frozen before storage at -80 °C.

PRMT5:MEP50 SPR STUDIES.

Compounds were tested for binding to the PRMT5:MEP50 complex via surface plasmon resonance using a Biacore S200 and Biacore 8K equipped with a SA sensor chip (Cytiva). Biotinylated PRMT5:MEP50 diluted to 10 µg/mL in a buffer consisting of 25 mM Bicine (pH 7.6), 100 mM sodium chloride, 1 mM TCEP and 0.05% P20 was loaded onto a streptavidin-coated biosensor chip at a flow rate of 10 mL/min until a capture level of ~2500 RUs was achieved. Compound binding studies were performed at 25 °C using the same buffer as used for immobilization but with 2% DMSO and with the addition of no cofactor, 10 µM MTA, or 25 µM SAM. MTA, SAM, and/or GSK591 were used as control molecules to test the activity of surfaces before each run. For apo studies on the Biacore S200, compound binding was tested in 11-point dose response and 2x dilution factor, using a top concentration of 50 µM and a flow rate of 50 µL/min, an association time of 60 s, and a dissociation time of 200 s, in MCK mode. For MTA/SAM cooperative binding studies on the Biacore 8K, compound binding was tested in 9-point dose response and 2x dilution factor, using a top concentration of 1 µM and a flow rate of 50 µL/min, an association time of 120 s, and a dissociation time of 4000 s, in SCK mode. Top concentrations and kinetic off-rate windows were adjusted as needed to accommodate tighter binding when observed.

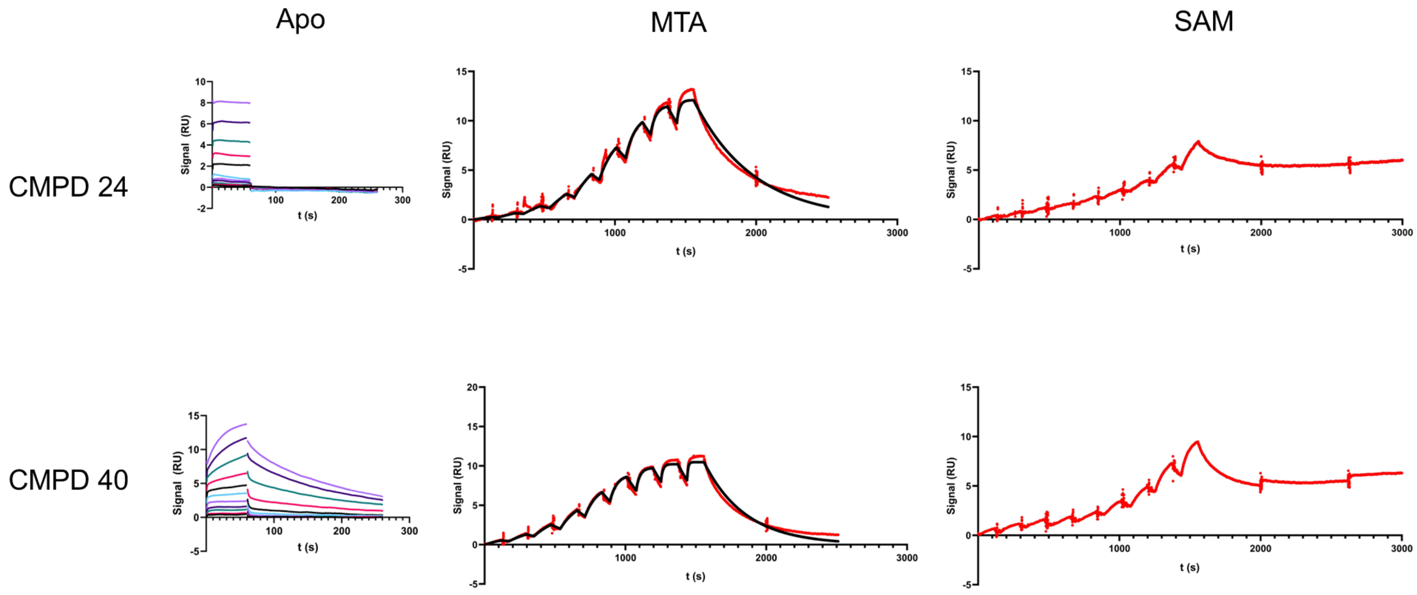

Figure SI-1. Binding of compounds 24 and 40 to PRMT5:MEP50 as demonstrated by SPR using either multi-cycle kinetics (apo) or single cycle kinetics (MTA and SAM conditions). Both compounds show MTA-cooperative binding; apo and SAM conditions show weaker binding and/or worse data quality.

Table SI-1. Fitted parameters from SPR kinetic data obtained for compounds 24 and 40.

Compound	k_a (1/Ms), MTA	k_d (1/s), MTA	K_D Kinetic (M), MTA	K_D Steady State (M), apo	Percent Binding - MTA	Percent Binding - Apo
**24**	4.80E4 ± 1.2E3	2.40E-3 ± 6.1E-05	5.0E-08 ± 2.49E-09	1.97E-05 ± 5.52E-06	111.3 ± 1.5	79.1 ± 2.7
**40**	1.41E5 ± 1.1E4	2.97E-3 ± 6.0E-04	2.12E-08 ± 5.88E-09	9.91E-06 ± 1.26E-06	131.0 ± 25.3	140.3 ± 6.3

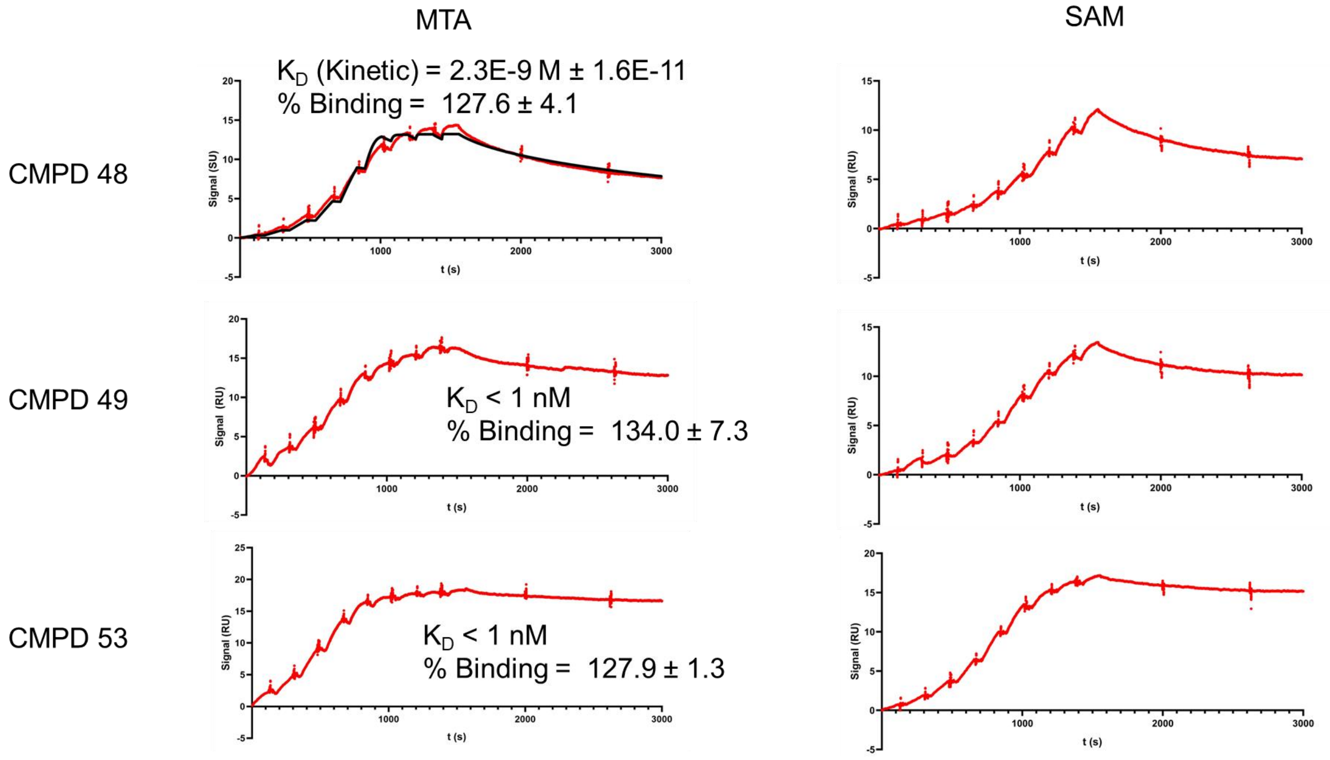

Figure SI-2. Binding of compounds 48, 49, and 53 to PRMT5:MEP50 by SPR using single-cycle kinetics. The images allow qualitative comparisons of binding between the two conditions. K_D and percent binding values are provided for the MTA condition; percent binding values for the SAM condition are not shown due to lack of confident saturation and non-ideal baseline drift.

PRMT5:MEP50 CRYSTALLOGRAPHY.

Crystals of the MTA-bound PRMT5:MEP50 complex were grown routinely from 15-17 mg/mL protein samples as described previously[3]. The structures of PRMT5:MEP50 + MTA containing compounds 41 or 46a or 51 were obtained from these crystals soaked with 1mM inhibitor for 2 – 5 h at room temperature. Crystals of PRMT5:MEP50 with compounds 24 or 47 or pamrametostat were obtained by cocrystallization of samples containing 1 mM cofactor and 1 mM inhibitor. These crystals were grown by vapor diffusion at 10 °C from conditions consisting of 15-35% PEG 3350 and 0.2 M ammonium sulfate, sometimes with added buffer. Crystals were transferred into a cryo solution consisting of mother liquor plus 20 % glycerol prior to flash-cooling in liquid nitrogen. Data collection occurred at multiple synchrotron sources as noted in Table SI-2. Data reduction and scaling were performed with XDS and AIMLESS[4,5] and structures were refined using either BUSTER (Global Phasing) or REFMAC from the CCP4 software suite[6]. Model building was performed using COOT[7]. Initial phases were obtained from previously determined structures of PRMT5:MEP50. All final models exhibited good geometry. Final coordinates and structure factors are deposited in the PDB with the accession codes noted in Table SI-2.

Table SI-2. Data collection and refinement statistics

	PRMT5/MEP50 MTA + GSK595	PRMT5/MEP50 MTA + compound 24	PRMT5/MEP50 MTA + compound 41	PRMT5/MEP50 MTA + compound 46a	PRMT5/MEP50 SFG + compound 47	PRMT5/MEP50 MTA + compound 51
**Data Collection**						
Synchrotron source	DLS	DLS	APS	SSRF	ALBA	SSRF
Beamline	I04	I04	21-ID-D	BL18U	XALOC	BL02U1
Space group	I222	I222	I222	I222	I222	I222
Cell dimensions						
a, *b*, *c* (Å)	99.7,137.0,178.9	100.3,137.1,178.6	101.7,137.8,179.1	99.1, 137.7, 178.4	101.7,137.7,178.0	102.9,137.9,178.6

Resolution (Å)	2.25	2.25	2.82	2.67	1.85	2.07
Unique reflections	24898	58619	30319	35013	74746	36863
Redundancy	6.5 (6.0)	6.6 (6.5)	13.5 (14.1)	6.7 (6.4)	6.6 (6.7)	13.5 (11.1)
Completeness (%)†	94.3 (81.0)	100 (100)	98.9 (98.4)	99.9 (99.9)	94.4 (44.4)	93.5 (81.5)
R_{merge}†	0.116 (1.028)	0.132 (0.896)	0.119 (1.115)	0.085 (0.707)	0.095 (1.595)	0.160 (1.343)
I/σ(I)†	11.3 (1.8)	7.5 (1.4)	14.2 (2.2)	14.9 (2.2)	4.1 (1.5)	12.9 (1.9)
$CC_{1/2}$	0.998 (0.668)	0.997 (0.611)	0.998 (0.924)	0.999 (0.934)	0.999 (0.057)	0.998 (0.758)
**Refinement**						
Reflections used	24874	56959	28805	34913	74731	35038
R_{work}/R_{free}	0.192/0.268	0.246/0.289	0.229/281	0.220/0.271	0.199/0.242	0.222/0.273
Average B-value ($Å^2$)	44.0	62.3	86.3	80.0	37.6	42.5
Number of atoms						
Protein	7358	7353	7391	7321	7364	7463
Cofactor/Inhibitor	53	56	55	57	54	65
Solvent/Other	234	321	20	188	506	192
R.m.s. deviations						
Bond lengths (Å)	0.013	0.002	0.002	0.013	0.008	0.002
Bond angles (°)	1.25	0.52	1.29	1.68	1.01	1.01
PDB ID code	9MGL	9MGM	9MGN	9MGP	9MGQ	9MGR

† Values in parentheses are for the highest resolution shell.

REFERENCES

(1) Yung-Chi, C.; Prusoff, W. H. Relationship between the Inhibition Constant (KI) and the Concentration of Inhibitor Which Causes 50 per Cent Inhibition (I50) of an Enzymatic Reaction. *Biochem. Pharmacol.* **1973**, *22* (23), 3099–3108. https://doi.org/10.1016/0006-2952(73)90196-2.

(2) Chan-Penebre, E.; Kuplast, K. G.; Majer, C. R.; Boriack-Sjodin, P. A.; Wigle, T. J.; Johnston, L. D.; Rioux, N.; Munchhof, M. J.; Jin, L.; Jacques, S. L.; West, K. A.; Lingaraj, T.; Stickland, K.; Ribich, S. A.; Raimondi, A.; Scott, M. P.; Waters, N. J.; Pollock, R. M.; Smith, J. J.; Barbash, O.; Pappalardi, M.; Ho, T. F.; Nurse, K.; Oza, K. P.; Gallagher, K. T.; Kruger, R.; Moyer, M. P.; Copeland, R. A.; Chesworth, R.; Duncan, K. W. A Selective Inhibitor of PRMT5 with in Vivo and in Vitro Potency in MCL Models. *Nat. Chem. Biol.* **2015**, *11* (6), 432–437. https://doi.org/10.1038/nchembio.1810.

(3) Cottrell, K. M.; Briggs, K. J.; Davis, C. B.; Gotur, D.; Huang, A.; Jahic, H.; Tonini, M. R.; Tsai, A.; Wilker, E. W.; Whittington, D. A.; Zhang, M.; Maxwell, J. P. Discovery of TNG908: A Selective, Brain Penetrant MTA-Cooperative PRMT5 Inhibitor That Is Synthetic Lethal with MTAP-Deleted Cancers. *Journal of Medicinal Chemistry* **2024**.

(4) Kabsch. XDS. *Acta Crystallographica* **2010**, No. d66, 125–132. https://doi.org/10.1107/s0907444909047337.

(5) Evans, P. R.; Murshudov, G. N. How Good Are My Data and What Is the Resolution? *Acta Crystallogr. Sect. D: Biol. Crystallogr.* **2013**, *69* (7), 1204–1214. https://doi.org/10.1107/s0907444913000061.

(6) Murshudov, G. N.; Vagin, A. A.; Dodson, E. J. Refinement of Macromolecular Structures by the Maximum-Likelihood Method. *Acta Crystallogr. Sect. D: Biol. Crystallogr.* **1997**, *53* (3), 240–255. https://doi.org/10.1107/s0907444996012255.

(7) Emsley, P.; Cowtan, K. Coot: Model-Building Tools for Molecular Graphics. *Acta Crystallogr. Sect. D: Biol. Crystallogr.* **2004**, *60* (12), 2126–2132. https://doi.org/10.1107/s0907444904019158.

AUTHOR INFORMATION

Corresponding Author

* **Kevin M. Cottrell** – *Tango Therapeutics, Boston, MA 02215, United States;* Phone: (+1) 857-320-4900; Email: kcottrell@tangotx.com

Authors

Douglas A. Whittington – Tango Therapeutics, Boston, MA 02215, United States
Kimberly J. Briggs – Tango Therapeutics, Boston, MA 02215, United States
Haris Jahic – Tango Therapeutics, Boston, MA 02215, United States
Janid A. Ali – Tango Therapeutics, Boston, MA 02215, United States
Alvaro J. Amor – Tango Therapeutics, Boston, MA 02215, United States
Deepali Gotur – Tango Therapeutics, Boston, MA 02215, United States
Matthew R. Tonini – Tango Therapeutics, Boston, MA 02215, United States
Wenhai Zhang – Tango Therapeutics, Boston, MA 02215, United States
Alan Huang – Tango Therapeutics, Boston, MA 02215, United States
John P. Maxwell – Tango Therapeutics, Boston, MA 02215, United States

Author Contributions

All authors have given approval to the final version of the manuscript.
